# Supplementary material for: Crown-hydroxylamines are pH-dependent chelating N,O-ligands with a potential for aerobic oxidation catalysis
Source: Nat Commun. 2023 Nov 23;14:7673. doi: 10.1038/s41467-023-43530-6 (PMC10667252; doi:10.1038/s41467-023-43530-6)
Supplement: Supplementary file 1 — Supplementary information [file 41467_2023_43530_MOESM1_ESM.pdf]

Supplementary information

Crown-hydroxylamines are pH-dependent chelating *N*,*O*-ligands with a potential for aerobic oxidation catalysis

Vladislav K. Lesnikov[1], Ivan S. Golovanov[1], Yulia V. Nelyubina[2,3], Svetlana A. Aksenova[2,3], Alexey Yu. Sukhorukov[1,*]

[1] N. D. Zelinsky Institute of Organic Chemistry, Russian Academy of Sciences, 119991, Leninsky prospect, 47, Moscow, Russian Federation

[2] A. N. Nesmeyanov Institute of Organoelement Compounds, Russian Academy of Sciences, 119991, Vavilova str. 28, Moscow, Russian Federation

[3] Moscow Institute of Physics and Technology (National Research University), 141700, Institutskiy per. 9, Dolgoprudny, Moscow Region, Russian Federation

* Corresponding author: sukhorukov@ioc.ac.ru

Table of contents

1. Supplementary methods **S3**

1.1 General experimental S3

1.2 Synthesis of starting macrocyclic polyamines S4

1.3 Synthesis of starting mono-*N*-protected macrocyclic polyamines S15

1.4 Synthesis of macrocyclic polyhydroxylamines and their derivatives S21

1.5 Deprotection of cyclam$(OBz)_4$: optimization study S32

1.6 Synthesis and characterization of d-metal complexes of macrocyclic polyhydroxylamines S33

1.7 Synthesis and characterization of nickel(II)-tacn complexes S40

1.8 Catalytic aerobic oxidation studies S41

1.9 X-ray crystallographic data and refinement details S49

1.10 Copies of NMR, FT-IR and UV-Vis spectra S63

1.11 Cyclic voltammetry analysis S213

1.12 HRMS studies of metal–cyclam$(OH)_4$ complexes S227

1.13 Competitive complexation of cyclam and cyclam$(OH)_4$ study S230

1.14 Potentiometric pH titration study S233

1.15 Quantum chemical calculations S235

2. Supplementary references **S257**

1. Supplementary methods

1.1 General experimental

All reactions were carried out in oven-dried (150°C) glassware. CH_2Cl_2, $CHCl_3$, and Et_3N were distilled over CaH_2; THF was distilled over $LiAlH_4$. Hexanes (Hex), ethyl acetate, CH_3CN, Et_2O, isopropanol and methanol were distilled without drying agents. 1,4,7-triazacyclononane (tacn) trihydrochloride, 1,5,9-triazacyclotridecane (tacd) trihydrobromide, cyclam, 1,4,8,12-tetraazacyclopentadecane, 1,4,7,10-tetraazacyclododecane (cyclen), diethylenetriamine, phthalimide, 1,2-ethanediol, 1,3-propanediol, 1,3-dibromopropane, hydrazine hydrate, methanesulfonyl chloride, propionic anhydride, phenol, Boc_2O, TsCl, $NiCl_2$•$6H_2O$, $Ni(NO_3)_2$•$6H_2O$, $Ni(ClO_4)_2$•$6H_2O$, $ZnCl_2$, $Zn(NO_3)_2$•$6H_2O$, $CuCl_2$•$2H_2O$, $MnCl_2$•$4H_2O$, $MnBr_2$, and other inorganic salts and acids were commercial grade and used as received.

NMR spectra were recorded at room temperature with residual solvent peaks as internal standards.[1] Multiplicities are indicated by s (singlet), d (doublet), m (multiplet), and br (broad).

Melting points were determined on a Kofler heating stage and were not corrected. HRMS experiments were performed on a mass-spectrometer with electrospray ionization and a time-of-flight (TOF) detector. HRMS data are given for major isotopes. Peaks in FT-IR spectra data are reported in cm^{-1} with the following relative intensities: s (strong), m (medium), w (weak), br (broad), sh (shoulder). UV-Vis spectra were recorded with the use of SF2000 spectrophotometer for the solutions of the investigated compounds. The peaks in the UV-Vis spectra data are reported in nm. Cyclic voltammetry (CV) experiments were performed for dimethylsulfoxide solutions with 0.1 M tetrabutylammonium hexafluorophosphate as a supporting electrolyte using a Metrohm Autolab PGSTAT128N potentiostat with a conventional one-compartment three-electrode cell (5 mL of solution) and 100mV/s scan rate. Quantum chemical calculations were performed with the Gaussian 16 Rev C.01[2] or ORCA 4.2.1[3] quantum chemistry programs.

For details of X-ray diffraction studies, CV measurements, potentiometric pH titration, and quantum chemical calculations see corresponding sections.

1.2 Synthesis of starting macrocyclic polyamines

General procedure for the synthesis of bis-tosylates of diols

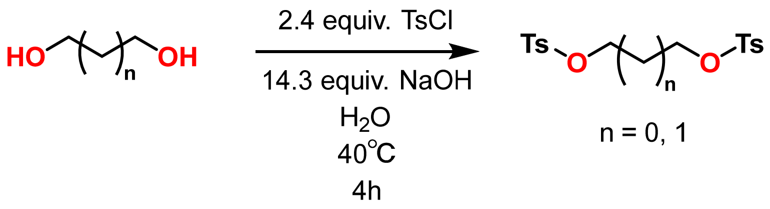

To a well-stirred mixture of TsCl (2.4 equiv.) and the corresponding diol (1 equiv.) heated to 40°C was added a solution of NaOH in water (40% w/w, 14.3 equiv.) dropwise within 30 minutes. The reaction mixture was stirred for 4 hours at 40°C. The, the resulting mixture was poured into an excess of water (ca. twice of the volume of the reaction mixture). If needed, more water was added to ensure complete dissolution of a precipitate. The product was extracted with CH_2Cl_2 (3×30 ml). Organic phase was dried with Na_2SO_4, and the solvent was removed under reduced pressure. The residue was dried under reduced pressure (c.a. 0.5 Torr) to give bis-tosylate as a white solid product.

1,2-Ethanediol ditosylate[4]

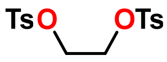

Prepared according to the general procedure from 22.1 g (116 mmol) of TsCl, 3.0 g (48.3 mmol) of 1,2-ethandiol and 69.1 g (691.2 mmol) of NaOH. Yield: 6.0 g (16.3 mmol, 34%). White solid. Mp = 124–125°C.

^{1}H NMR (300 MHz, $CDCl_3$) δ 7.73 (d, J = 8.2 Hz, 4H, 4 CH_{Ar}), 7.33 (d, J = 8.2 Hz, 4H, 4 CH_{Ar}), 4.18 (s, 4H, 2 CH_2), 2.45 (s, 6H, 2 CH_3).

^{13}C NMR (76 MHz, $CDCl_3$) δ 145.4 (2 C_{Ar}), 132.5 (2 C_{Ar}), 130.1 (4 CH_{Ar}), 128.1 (4 CH_{Ar}), 66.8 (2 CH_2), 21.8 (2 CH_3).

ESI-HRMS m/z: $[M+NH_4]^+$ Calcd for $[C_{16}H_{18}O_6S_2+NH_4]^+$ 388.0883; Found 388.0873.

Physical properties and NMR spectral data are in agreement with to those given in the literature.[4]

1,3-Propanediol ditosylate[4]

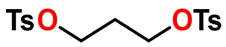

Prepared according to the general procedure from 6.0 g (31.5 mmol) of TsCl, 1.0 g (13.1 mmol) of 1,3-propanediol and 18.8 g (188 mmol) of NaOH. Yield: 3.1 g (8.1 mmol, 61%). White solid. Mp = 92–93°C.

^{1}H NMR (300 MHz, $CDCl_3$) δ 7.71 (d, J = 8.2 Hz, 4H, 4 CH_{Ar}), 7.32 (d, J = 8.2 Hz, 4H, 4 CH_{Ar}), 4.04 (t, J = 6.0 Hz, 4H, 2 CH_2), 2.42 (s, 6H, 2 CH_3), 1.97 (p, J = 6.0 Hz, 2H, CH_2).

^{13}C NMR (76 MHz, $CDCl_3$) δ 145.2 (2 C_{Ar}), 132.7 (2 C_{Ar}), 130.1 (4 CH_{Ar}), 128.0 (4 CH_{Ar}), 66.0 (2 CH_2), 28.7 (CH_2), 21.7 (2 CH_3).

Anal. Calcd. for $C_{17}H_{20}O_6S_2$: C, 53.11; H, 5.24. Found: C, 53.05; H, 5.12.

Physical properties and NMR spectral data are in agreement with to those given in the literature.[4]

General procedure for the synthesis of *N*,*N'*,*N''*-tritosylates of linear triamines

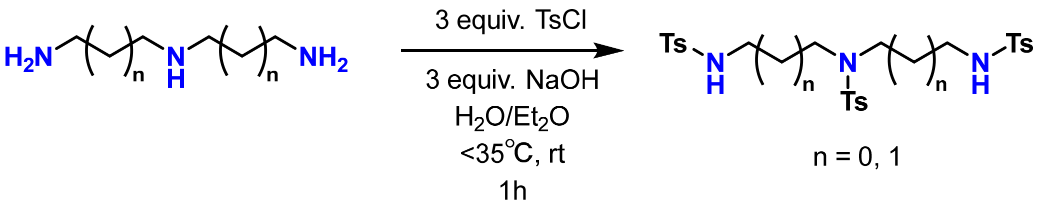

To a cooled solution of the corresponding triamine (1.0 equiv.) in water (1.25 M) was added NaOH (3.0 equiv.) portionwise keeping temperature of reaction mixture below 35°C. To this solution was added a solution of TsCl (3.0 equiv.) in Et_2O (ca. 2 M) dropwise. The resulting mixture was stirred under ambient temperature in 1 hour. The white precipitate formed was filtered, washed with water several times and dried under reduced pressure (c.a. 0.5 Torr) with $CaCl_2$. The resulting tris-tosylate was obtained as white foam.

***N*,*N'*,*N''*-Tri-tosyl-diethylenetriamine**[4]

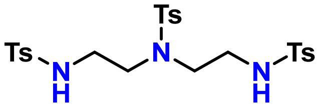

Prepared according to the general procedure from 5.5 g (29.1 mmol) of TsCl, 1.0 g (9.7 mmol) of diethylenetriamine and 1.16 g (29.1 mmol) of NaOH. Yield: 5.0 g (8.8 mmol, 91%). White solid. Mp = 174–175°C.

^{1}H NMR (300 MHz, DMSO-d_6, HSQC) δ 7.67 (d and br, J = 8.1 Hz, 6H, 4 CH_{Ar} and 2 NH), 7.56 (d, J = 8.0 Hz, 2H, 2 CH_{Ar}), 7.39 (d, J = 8.2 Hz, 4H, 4 CH_{Ar}), 7.36 (d, J = 8.0 Hz, 2H, 2 CH_{Ar}), 3.10 – 2.97 (m, 4H, 2 CH_2), 2.91 – 2.77 (m, 4H, 2 CH_2), 2.38 (s, 9H, 3 CH_3).

^{13}C NMR (76 MHz, DMSO-d_6, HSQC) δ 143.5 (C_{Ar}), 142.8 (2 C_{Ar}), 137.4 (2 C_{Ar}), 135.4 (C_{Ar}), 129.9 (2 CH_{Ar}), 129.7 (4 CH_{Ar}), 126.8 (2 CH_{Ar}), 126.6 (4 CH_{Ar}), 48.4 (2 CH_2), 41.6 (2 CH_2), 21.0 (3 CH_3).

Anal. Calcd. for $C_{25}H_{31}N_3O_6S_3$: C, 53.08; H, 5.52; N, 7.43. Found: C, 53.21; H, 5.65; N, 7.59.

Physical properties and NMR spectral data are in agreement with to those given in the literature.[4]

1,5,9-Tri-tosyl-1,5,9-triazanonane[5]

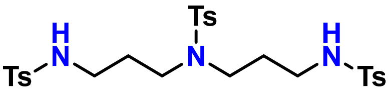

Prepared according to the general procedure from 4.36 g (22.9 mmol) of TsCl, 1.0 g (7.6 mmol) of 1,5,9-triazanonane and 0.91 g (22.9 mmol) of NaOH. Yield: 4.0 g (6.8 mmol, 89%). White solid. Mp = 116–117°C.

^{1}H NMR (300 MHz, DMSO-d_6, HSQC) δ 7.68 (d, J = 8.2 Hz, 4H, 4 CH_{Ar}), 7.62 (d, J = 8.1 Hz, 2H, 2 CH_{Ar}), 7.58 – 7.47 (br m, 2H, NH), 7.37 (d, J = 7.6 Hz, 6H, 6 CH_{Ar}), 2.95 (t, J = 7.4 Hz, 4H, 2 CH_2), 2.69 (br t, J = 7.1 Hz, 4H, 2 CH_2), 2.37 (s, 3H, CH_3), 2.36 (s, 6H, 2 CH_3), 1.64 – 1.46 (m, 4H, 2 CH_2).

^{13}C NMR (76 MHz, DMSO-d_6, HSQC) δ 143.1 (C_{Ar}), 142.6 (2 C_{Ar}), 137.5 (2 C_{Ar}), 136.0 (C_{Ar}), 129.8 (2 CH_{Ar}), 129.7 (4 CH_{Ar}), 126.9 (2 CH_{Ar}), 126.6 (4 CH_{Ar}), 46.0 (2 CH_2), 40.2 (2 CH_2), 28.5 (2 CH_2), 21.0 (3 CH_3).

Anal. Calcd. for $C_{27}H_{35}N_3O_6S_3$: C, 54.62; H, 5.94; N, 7.08. Found: C, 54.58; H, 5.74; N, 7.16.

Physical properties and NMR spectral data are in agreement with to those given in the literature.[5]

Synthesis of 1,5,9,13,17-pentatosyl-1,5,9,13,17-pentaazaeicosane

The three-step synthetic route used for the synthesis of 1,5,9,13,17-pentatosyl-1,5,9,13,17-pentaazaeicosane is depicted below:

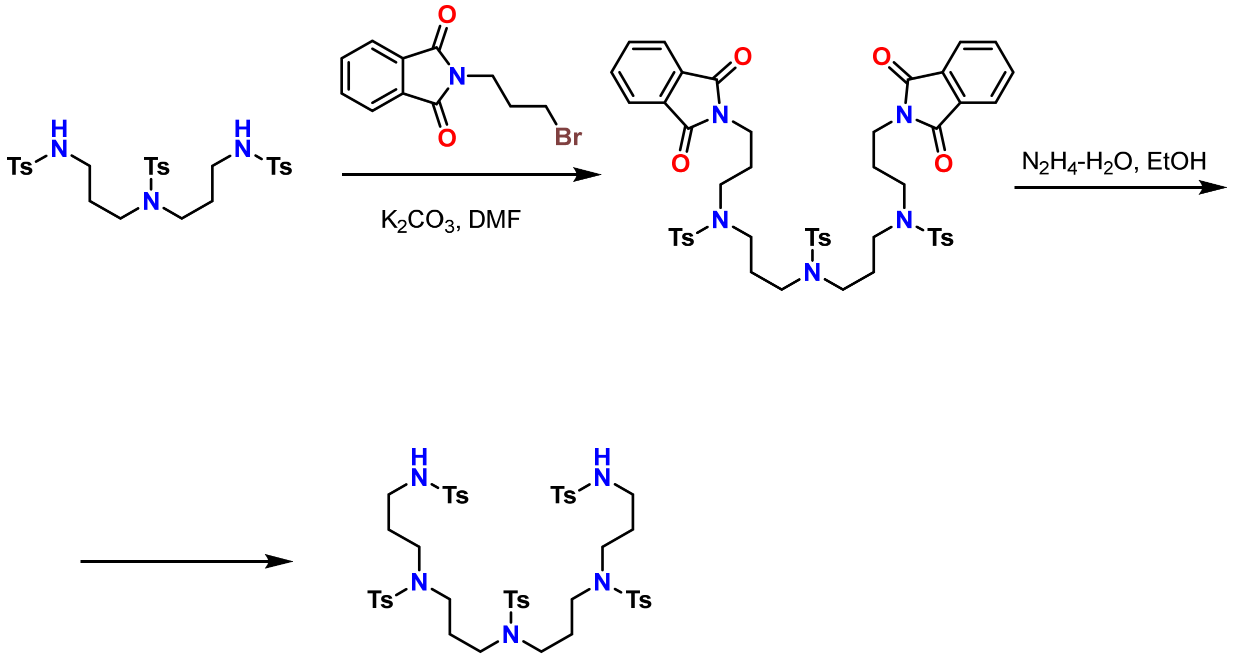

Step 1: synthesis of *N*-(3-bromopropyl)phthalimide[6]

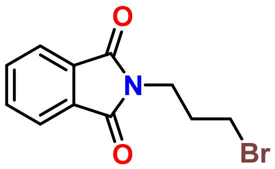

A mixture of phthalimide (491 mg, 3.34 mmol, 0.67 equiv.), 1,3-dibromopropane (1 g, 4.95 mmol, 1 equiv.) and K_2CO_3 (922 mg, 6.68 mmol, 1.35 equiv.) in DMF (6 ml) was stirred at room temperature for 36 h (light green color disappears). Then, 10 ml of water was added and the product was extracted with CH_2Cl_2 (3×25 ml). The organic phase was washed with saturated NaCl solution, dried over Na_2SO_4. The volatiles were removed under reduced pressure. The residue was subjected to a column chromatography on silica gel (Hex:EtOAc, 9:1) to give 518 mg (57%) of *N*-(3-bromopropyl)phthalimide as a white solid.

^{1}H NMR (300 MHz, $CDCl_3$, HSQC) δ 7.83 (dd, J = 5.4, 3.1 Hz, 2H, 2 CH_{Ar}), 7.71 (dd, J = 5.5, 3.1 Hz, 2H, 2 CH_{Ar}), 3.82 (t, J = 6.8 Hz, 2H, CH_2), 3.40 (t, J = 6.7 Hz, 2H, CH_2), 2.24 (p, J = 6.8 Hz, 2H, CH_2).

^{13}C NMR (76 MHz, $CDCl_3$, HSQC) δ 168.3 (2 C=O), 134.2 (2 CH_{Ar}), 132.1 (2 C_{Ar}), 123.4 (2 CH_{Ar}), 36.8 (CH_2), 31.7 (CH_2), 29.9 (CH_2).

ESI-HRMS m/z: $[M+Na]^+$ Calcd for $[C_{11}H_{10}BrNO_2+Na]^+$ 289.9787 and 291.9767; Found 289.9793 and 291.9770.

Physical properties and NMR spectral data are in agreement with to those given in the literature.[6]

Step 2: Synthesis of 1,17-phthalimido-5,9,13-tritosyl-5,9,13-triazaheptadecane[6]

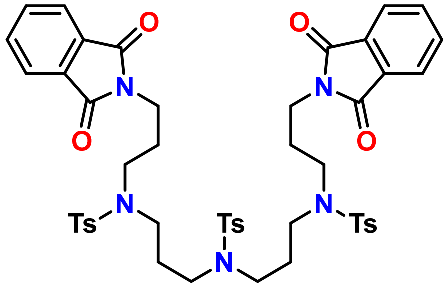

A solution of *N*-(3-bromopropyl)phthalimide (2.2 g, 8.0 mmol) in DMF (0.37 M) was added to mixture of K_2CO_3 (5.5 g, 40 mmol, 15 equiv.) and 1,5,9-tritosyl-1,5,9-triazanonane (1.5 g, 2.5 mmol, 1 equiv.) in DMF (0.17 M) at room temperature. The mixture turned light green in color. After 36 hours, the reaction mixture was filtered, and the filtrate was concentrated under reduced pressure. The residue was subjected to a column chromatography on silica gel (Hex:EtOAc 1:1, R_f ~ 0.5) to give 1.8 g (1.86 mmol, 74%) of 1,17-phthalimido-5,9,13-tritosyl-5,9,13-triazaheptadecane as an amorphous solid.

^{1}H NMR (300 MHz, $CDCl_3$, HSQC) δ 7.80 (dd, J = 5.5, 3.0 Hz, 4H, 4 CH_{Ar}), 7.69 (dd, J = 5.5, 3.1 Hz, 4H, 4 CH_{Ar}), 7.65 (d, J = 8.2 Hz, 2H, 2 CH_{Ar}), 7.63 (d, J = 8.3 Hz, 4H, 4 CH_{Ar}), 7.30 (d, J = 8.2 Hz, 2H, 2 CH_{Ar}), 7.27 (d, J = 8.3 Hz, 4H, 4 CH_{Ar}), 3.67 (t, J = 7.2 Hz, 4H, 2 CH_2), 3.26 – 3.06 (m, 12H, 6 CH_2), 2.41 (s, 3H, CH_3), 2.40 (s, 6H, 2 CH_3), 1.99 – 1.82 (m, 8H, 4 CH_2).

^{13}C NMR (76 MHz, $CDCl_3$, HSQC) δ 168.1 (4 C=O), 143.3 (3 C_{Ar}), 135.9 (3 C_{Ar}), 134.0 (4 CH_{Ar}), 131.9 (4 C_{Ar}), 129.7 (6 CH_{Ar}), 127.1 (6 CH_{Ar}), 123.1 (4 CH_{Ar}), 59.1 (2 CH_2), 46.7 (2 CH_2), 46.5 (2 CH_2), 35.5 (2 CH_2), 28.6 (2 CH_2), 27.7 (2 CH_2), 21.4 (3 CH_3).

ESI-HRMS m/z: $[M+Na]^+$ Calcd for $[C_{49}H_{53}N_5O_{10}S_3Na]^+$ 990.2847; Found 990.2831.

Physical properties and NMR spectral data are in agreement with to those given in the literature.[6]

Step 3: Synthesis of 1,5,9,13,17-pentatosyl-1,5,9,13,17-pentaazaeicosane[6]

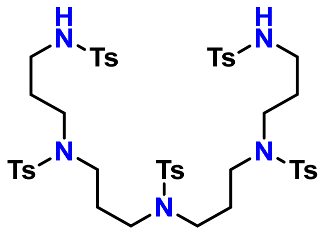

A mixture of 1,17-phthalimido-5,9,13-tritosyl-5,9,13-triazaheptadecane (1.8 g, 1.88 mmol, 1 equiv.) and hydrazine monohydrate (0.75 g, 15 mmol, 8 equiv.) in EtOH (45 ml) was refluxed for 48 h. After consumption of the starting material (TLC control, Hex:EtOAc 1:1), the reaction mixture was filtered and the solvent was removed under reduced pressure. The residue was suspended in a saturated NaCl solution and the product was extracted with CH_2Cl_2 (2×40 ml). The organic phase was dried under Na_2SO_4 and concentrated under reduced pressure. The residue was dissolved in diethyl ether (4 ml) with the addition of $CHCl_3$ until complete dissolution. To the resulting solution was added TsCl (0.68 g, 3.6 mmol, 2 equiv.) followed by a 40 wt% solution of NaOH (0.14 g, 3.6 mmol, 2 equiv.) in water (dropwise). After 4 hours, the mixture was poured into cold water (20 ml) and the product was extracted with CH_2Cl_2 (3×15 ml). The organic phase was washed with a saturated NaCl solution, dried under Na_2SO_4 and concentrated under reduced pressure. The residue was subjected to a column chromatography on silica gel (Hex:EtOAc 1:1→EtOAc) to give 1.39 g (1.37 mmol, 77%) of 1,5,9,13,17-pentatosyl-1,5,9,13,17-pentaazaeicosane as an amorphous solid.

^{1}H NMR (300 MHz, $CDCl_3$, HSQC) δ 7.72 (d, J = 8.0 Hz, 4H, 4 CH_{Ar}), 7.64 (d, J = 8.2 Hz, 6H, 6 CH_{Ar}), 7.33 – 7.22 (m, 10H, 10 CH_{Ar}), 5.44 (t, J = 6.4 Hz, 2H, 2 NH), 3.20 – 3.03 (m, 12H, 6 CH_2), 3.02 – 2.89 (m, 4H, 2 CH_2), 2.41 (s, 9H, 3 CH_3), 2.39 (s, 6H, 2 CH_3), 1.93 – 1.70 (m, 8H, 4).

^{13}C NMR (76 MHz, $CDCl_3$, HSQC) δ 143.8 (2 C_{Ar}), 143.7 (C_{Ar}), 143.4 (2 C_{Ar}), 136.9 (2 C_{Ar}), 135.7 (C_{Ar}), 135.5 (2 C_{Ar}), 130.0 (6 CH_{Ar}), 129.8 (4 CH_{Ar}), 127.2 (6 CH_{Ar}), 127.1 (4 CH_{Ar}), 47.6 (2 CH_2), 47.3 (2 CH_2), 46.9 (2 CH_2), 40.3 (2 CH_2), 29.4 (2 CH_2), 29.2 (2 CH_2), 21.6 (5 CH_3).

ESI-HRMS m/z: $[M+Na]^+$ Calcd for $[C_{47}H_{61}N_5O_{10}S_5Na]^+$ 1038.2914; Found 1038.2904.

Physical properties and NMR spectral data are in agreement with to those given in the literature.[6]

General procedure for the synthesis of symmetrical macrocyclic *N*-tosylamines via macrocyclization

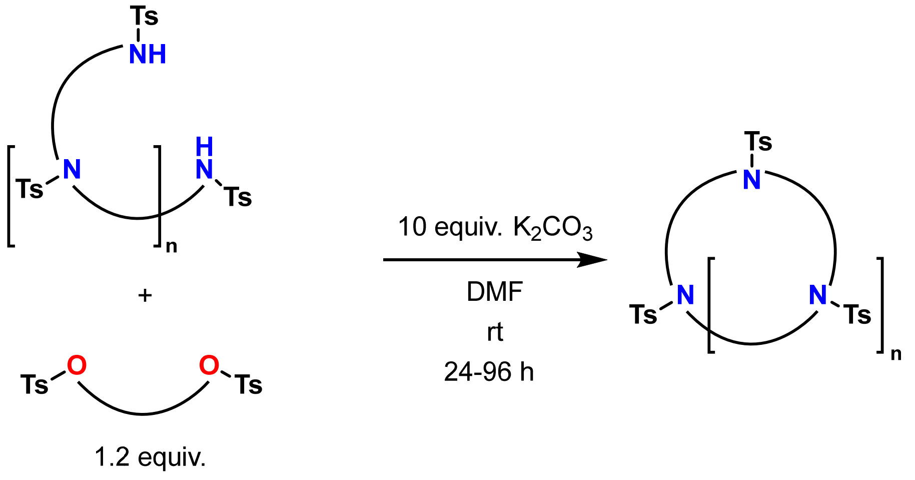

A mixture of finely ground and dried K_2CO_3 (10 equiv.), *N*-tosylated polyamine (1 equiv.) and di-tosylated diol (1.2 equiv.) was stirred under ambient temperature in DMF (0.02 M with respect to K_2CO_3) for 24–96 h (TLC control, CH_2Cl_2). After completion of reaction, the precipitate was filtered and discarded. The filtrate was concentrated under reduced pressure to ca. 1/10 of initial volume. The residue was diluted with water (ca. 5 volumes of the residue) and left at 0–5°C until the formation of precipitate. Water was decanted, the precipitate was washed 2 times with water (ca. ½ of the volume of decanted water). Water phase was washed with CH_2Cl_2 twice (1:1 volume). The organic phase was combined with the precipitate, and, if needed, diluted with additional CH_2Cl_2 for complete dissolving, dried with Na_2SO_4, and concentrated under reduced pressure. The residue was dried under reduced pressure (ca. 0.5 Torr) to give the crude product as a white foam. For analytical purposes, the products were recrystallized from a large amount of MeOH.

1,5,9-Tritosyl-1,5,9-triazacyclododecane[7]

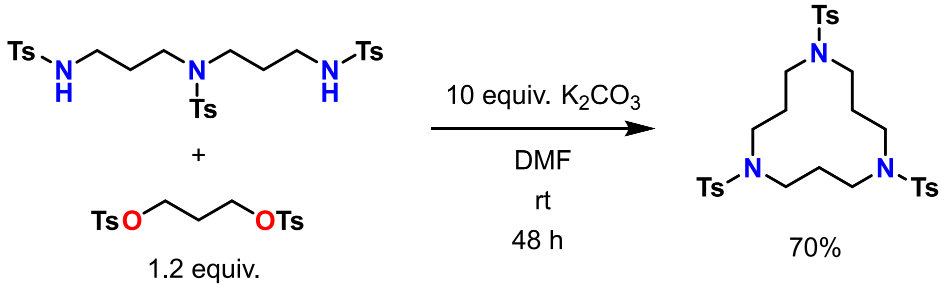

Prepared according to the general procedure from 0.6 g (1.01 mmol) of 1,4,8-tri-tosyl-1,4,8-triazanonane, 0.47 g (1.2 mmol) of 1,3-propanediol ditosylate and 1.4 g (10.1 mmol) of K_2CO_3 (reaction time – 48 h). Yield: 0.45 g (0.7 mmol, 70%). White solid. Mp = 170–171°C.

^{1}H NMR (300 MHz, $CDCl_3$) δ 7.65 (d, J = 7.9 Hz, 6H, 6 CH_{Ar}), 7.31 (d, J = 7.9 Hz, 6H, 6 CH_{Ar}), 3.21 (t, J = 6.7 Hz, 12H, 6 CH_2), 2.43 (s, 9H, 3 CH_3), 1.91 (p, J = 6.8 Hz, 6H, 3 CH_2).

^{13}C NMR (75 MHz, $CDCl_3$) δ 143.7 (3 C_{Ar}), 135.2 (3 C_{Ar}), 129.9 (6 CH_{Ar}), 127.3 (6 CH_{Ar}), 45.6 (6 CH_2), 26.4 (3 CH_2), 21.6 (3 CH_3).

ESI-HRMS m/z: $[M+H]^+$ Calcd for $[C_{30}H_{40}N_3O_6S_3]^+$ 634.2074; Found 634.2065. m/z: $[M+NH_4]^+$ Calcd for $[C_{30}H_{39}N_3O_6S_3+NH_4]^+$ 651.2339; Found 651.2336.

Physical properties and NMR spectral data are in agreement with to those given in the literature.[7]

1,5,9,13,17-Pentatosyl-1,5,9,13,17-pentaazacycloeicosane[8]

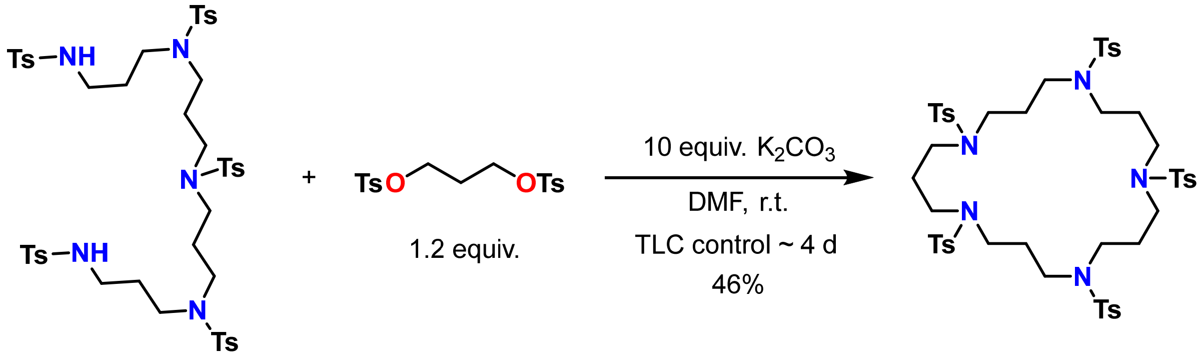

Prepared according to the general procedure from 0.23 g (0.22 mmol) of 1,5,9,13,17-pentatosyl-1,5,9,13,17-pentaazaeicosane, 0.1 g (0.27 mmol) of 1,3-propanediol ditosylate and 0.3 g (2.2 mmol) of K_2CO_3. Yield: 0.11 g (0.1 mmol, 46%). White solid. Mp = 211–213°C.

^1H NMR (300 MHz, $CDCl_3$) δ 7.66 (d, J = 8.1 Hz, 10H, 10 CH_{Ar}), 7.31 (d, J = 8.1 Hz, 10H, 10 CH_{Ar}), 3.16 (t, J = 7.1 Hz, 20H, 10 CH_2), 2.42 (s, 15H, 5 CH_3), 1.94 (p, J = 7.1 Hz, 10H, 5 CH_2).

Physical properties and NMR spectral data are in agreement with to those given in the literature.[8]

General procedure for the synthesis of unsymmetrical macrocyclic *N*-tosylamines via macrocyclization

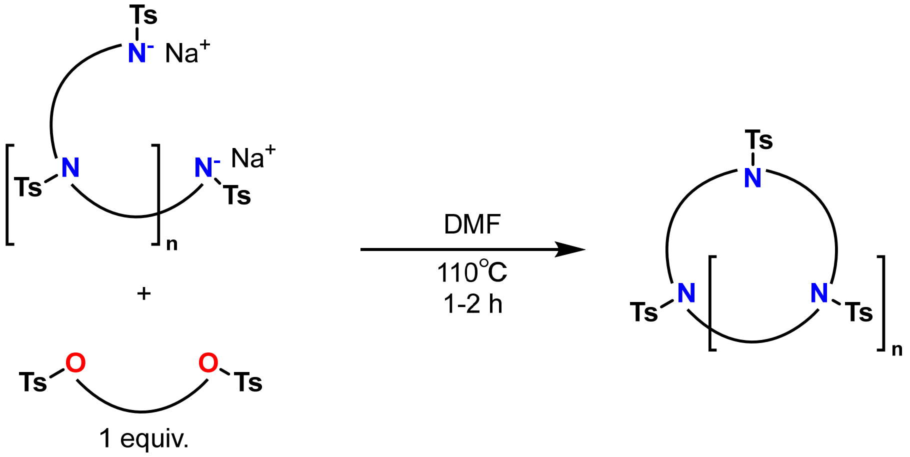

Generation of disodium salts of tris-tosylated triamines: a freshly prepared sodium ethoxide (2 equiv.) was rapidly added to a suspension of tri-tosylated triamine (1 equiv.) in boiling EtOH (0.75 M). After the addition, the solvent was removed under reduced pressure and residue was

dried in a vacuum (ca. 0.5 Torr). The resulting disodium salt of tris-tosylated triamine was used without further purification.

Macrocyclization: The disodium salt of tri-tosylated triamine (1 equiv.) from the previous stage was heated in DMF (0.1 M) to 110°C. Then, a solution of di-tosidated diol (1 equiv.) in DMF (0.2 M) was added dropwise. After addition was completed, the reaction mixture was stirred for 2 h at 110°C. After consumption of the starting material (TLC control, CH_2Cl_2), the mixture was cooled to rt and concentrated under reduced pressure to ca. ¼ of its initial volume. Cold distilled water (ca. 3 volumes of the initial solution) was added, and the mixture was left at 0–5°C until the precipitation was complete. The water phase was decanted, and the residual solid was dissolved in CH_2Cl_2. The solution was dried under Na_2SO_4 and concentrated under reduced pressure. The residue was dried in a vacuum (ca. 0.5 Torr) until a glassy substance formed. Then, EtOH was added to the residue (ca. 3 ml per 500 mg) and heated to boiling until a white crystalline powder of the product was formed. After cooling the precipitate was filtered, dried in a vacuum (c.a. 0.5 Torr) to give the product as a white solid.

1,4,7-Tri-tosyl-1,4,7-triazacyclodecane[5]

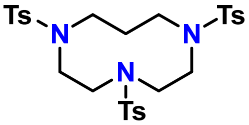

Prepared according to the general procedure from 0.5 g (0.88 mmol) of 1,4,7-tri-tosyl-diethylenetriamine, 0.34 g (0.88 mmol) of 1,3-propanediol ditosylate and 0.12 g (1.77 mmol) of EtONa. Yield: 0.37 g (0.61 mmol, 69%). White solid. Mp = 222–224°C.

^{1}H NMR (300 MHz, $CDCl_3$, HSQC) δ 7.72 (d, J = 8.3 Hz, 2H, 2 CH_{Ar}), 7.69 (d, J = 8.2 Hz, 4H, 4 CH_{Ar}), 7.32 (d, J = 8.3 Hz, 2H, 2 CH_{Ar}), 7.31 (d, J = 8.2 Hz, 4H, 4 CH_{Ar}), 3.37 (s, 8H, 4 CH_2), 3.20 (br t, J = 5.5 Hz, 4H, 2 CH_2), 2.42 (s, 9H, 3 CH_3), 2.24 – 2.13 (m, 2H, CH_2).

^{13}C NMR (76 MHz, $CDCl_3$, HSQC) δ 143.94 (C_{Ar}), 143.86 (2 C_{Ar}), 134.7 (3 C_{Ar}), 129.9 (6 CH_{Ar}), 127.8 (2 CH_{Ar}), 127.7 (4 CH_{Ar}), 52.1 (2 CH_2), 52.0 (2 CH_2), 49.6 (2 CH_2), 29.4 (CH_2), 21.6 (3 CH_3).

ESI-HRMS m/z: $[M+NH_4]^+$ Calcd for $[C_{28}H_{35}N_3O_6S_3+NH_4]^+$ 623.2026; Found 623.2003.

Physical properties and NMR spectral data are in agreement with to those given in the literature.[5]

1,4,8-Tri-tosyl-1,4,8-triazacycloundecane[9]

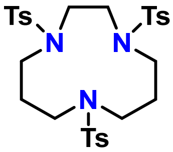

Prepared according to general procedure from 0.5 g (0.84 mmol) of 1,4,8-tri-tosyl-1,4,8-triazanonane, 0.31 g (0.84 mmol) of 1,2-ethanediol ditosylate and 0.11 g (1.68 mmol) of EtONa. Yield: 0.38 g (0.61 mmol, 72%). White solid. Mp = 211–213°C.

^1H NMR (300 MHz, $CDCl_3$, HSQC) δ 7.68 (d, J = 8.1 Hz, 2H, 2 CH_{Ar}), 7.63 (d, J = 8.0 Hz, 4H, 4 CH_{Ar}), 7.31 (d, J = 8.0 Hz, 4H, 4 CH_{Ar}), 7.30 (d, J = 8.1 Hz, 2H, 2 CH_{Ar}), 3.42 (t, J = 6.9 Hz, 4H, 2 CH_2), 3.27 (s, 4H, 2 CH_2), 2.99 (t, J = 5.6 Hz, 4H, 2 CH_2), 2.42 (s, 9H, 3 CH_3), 1.90 (p, J = 6.3 Hz, 4H, 2 CH_2).

^{13}C NMR (76 MHz, $CDCl_3$, HSQC) δ 144.1 (2 C_{Ar}), 143.5 (C_{Ar}), 135.8 (C_{Ar}), 133.9 (2 C_{Ar}), 130.0 (4 CH_{Ar}), 129.9 (2 CH_{Ar}), 127.7 (4 CH_{Ar}), 127.4 (2 CH_{Ar}), 52.6 (2 CH_2), 49.0 (2 CH_2), 43.4 (2 CH_2), 24.1 (2 CH_2), 21.6 (3 CH_3).

ESI-HRMS m/z: $[M+NH_4]^+$ Calcd for $[C_{29}H_{37}N_3O_6S_3+NH_4]^+$ 637.2183; Found 637.2178.

Physical properties and NMR spectral data are in agreement with to those given in the literature.[9]

General procedure for deprotection of macrocyclic *N*-tosylamines

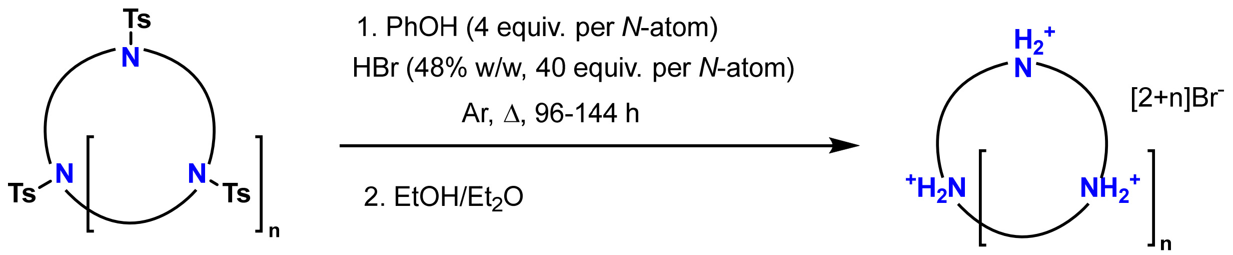

A mixture of *N*-tosylated macrocyclic polyamine (1 eq.), phenol (4 eq. per one *N*-atom) and hydrobromic acid (48% w/w, 40 eq. per one *N*-atom) was refluxed in a stream of argon for 96–144 h (TLC control). After consumption of the starting material, the reaction mixture was transferred in separating funnel and washed with diethyl ether three times (5 volumes of hydrobromic acid each). The aqueous phase was collected and concentrated under reduced pressure to ca. $^1/_5$ of the initial volume. Ethanol (ca. $^1/_2$ volume of hydrobromic acid) was added to the residue and the resulting mixture was poured into a cooled diethyl ether (ca. 3 volumes of hydrobromic acid). The precipitate was filtered, washed with Et_2O and dried in a vacuum (ca. 0.5 Torr) to give the pure hydrobromide salt of macrocyclic polyamine.

1,4,7-Triazacyclodecane trihydrobromide[5]

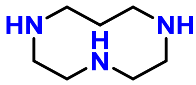

Prepared according to the general procedure from 0.5 g (0.8 mmol) of 1,4,7-tritosyl-1,4,7-triazacyclodecane, 1.01 g (10.7 mmol) of phenol and 16.7 g (11 ml, 99.1 mmol) of 48% HBr. Yield: 0.16 g (0.42 mmol, 51%). Pale yellow solid.

^{1}H NMR (300 MHz, HSQC, D_2O) δ 3.39 – 3.33 (m, 4H, 2 CH_2), 3.33 – 3.26 (m, 8H, 4 CH_2), 2.07 (p, J = 6.0 Hz, 2H, CH_2) (NH hydrogens were not observed due to exchange with D_2O).

^{13}C NMR (76 MHz, HSQC, D_2O) δ 45.0 (2 CH_2), 43.7 (4 CH_2), 21.3 (CH_2).

ESI-HRMS m/z: $[M+H]^+$ Calcd for $[C_7H_{18}N_3]^+$ 144.1495; Found 144.1495.

NMR spectral data are in agreement with to those given in the literature.[5]

1,4,8-Triazacycloundecane trihydrobromide[9]

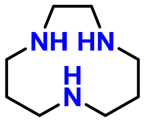

Prepared according to the general procedure from 0.43 g (0.69 mmol) of 1,4,8-tritosyl-1,4,8-triazacycloundecane, 0.85 g (9 mmol) of phenol and 13.9 g (9.5 ml, 82.7 mmol) of 48% HBr. Yield: 0.2 g (0.5 mmol, 72%). Pale yellow solid.

^{1}H NMR (300 MHz, HSQC, D_2O) δ 3.34 – 3.23 (m, 8H, 4 CH_2), 3.25 (s, 4H, 2 CH_2), 2.05 (p, J = 6.1 Hz, 4H, 2 CH_2) (NH hydrogens were not observed due to exchange with D_2O).

^{13}C NMR (76 MHz, HSQC, D_2O) δ 45.5 (2 CH_2), 45.3 (2 CH_2), 43.7 (2 CH_2), 21.0 (2 CH_2).

ESI-HRMS m/z: $[M+H]^+$ Calcd for $[C_8H_{20}N_3]^+$ 158.1652; Found 158.1653.

NMR spectral data are in agreement with to those given in the literature.[9]

1,5,9-Triazacyclododecane trihydrobromide[7]

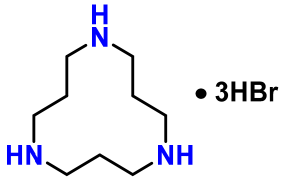

Prepared according to the general procedure from 0.29 g (0.45 mmol) of 1,5,9-tritosyl-1,5,9-triazacyclododecane, 0.56 g (5.9 mmol) of phenol and 9.2 g (6.0 ml, 54.5 mmol) of 48% HBr. Yield: 0.12 g (0.29 mmol, 63%). Pale yellow solid.

^{1}H NMR (300 MHz, HSQC, D_2O) δ 3.41 (t, J = 6.7 Hz, 12H, 6 CH_2), 2.28 (p, J = 6.7 Hz, 6H, 3 CH_2).

^{13}C NMR (76 MHz, HSQC, D_2O) δ 41.0 (6 CH_2), 19.1 (3 CH_2).

ESI-HRMS m/z: $[M+H]^+$ Calcd for $[C_9H_{22}N_3]^+$ 172.1808; Found 172.1814.

Physical properties and ^{1}H NMR spectral data are in agreement with to those given in the literature.[7]

1,5,9,13,17-Pentaazacycloeicosane pentahydrobromide[8]

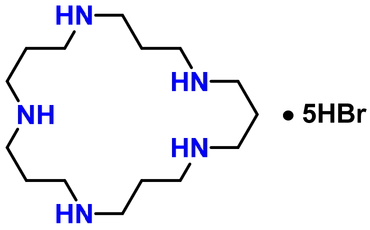

Prepared according to the general procedure from 0.85 g (0.8 mmol) of 1,5,9,13,17-pentatosyl-1,5,9,13,17-pentaazacycloeicosane, 1.5 g (16 mmol) of phenol and 27 g (18 ml, 160 mmol) of 48% HBr. Yield: 0.37 g (0.53 mmol, 66%). Pale yellow solid.

^{1}H NMR (300 MHz, D_2O) δ 3.41 (t, J = 7.4 Hz, 20H, 10 CH_2), 2.30 (p, J = 7.4 Hz, 10H, 5 CH_2).

NMR spectral data are in agreement with to those given in the literature.[8]

1.3 Synthesis of starting mono-*N*-protected macrocyclic polyamines

General procedure for the synthesis of tris-Boc-protected macrocyclic tetraamines

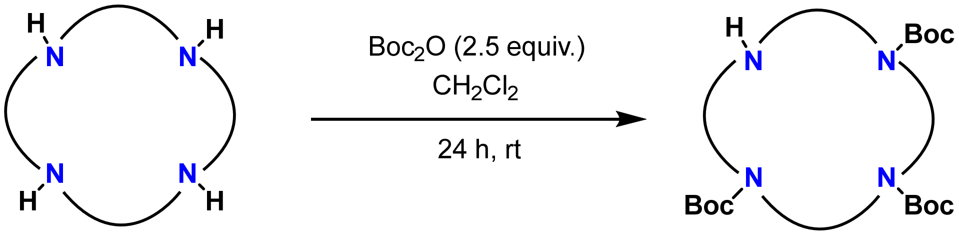

To a stirred solution of a macrocyclic polyamine (1 equiv.) in CH_2Cl_2 (16 mM) was added dropwise a solution of Boc_2O (2.5 equiv.) in CH_2Cl_2 (80 mM) at rt. The reaction mixture was kept for ca. 24 h. Then, the solvent was removed under reduced pressure and the residue was subjected to a column chromatography on silica gel (Hex:EtOAc = 9:1→1:1→EtOAc).

Tris-tert-butyl 1,4,8,11-tetraazacyclotetradecane-1,4,8-tricarboxylate[10]

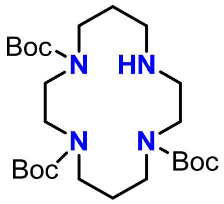

Prepared according to the general procedure from 0.1 g (0.5 mmol) of cyclam and 0.27 g (1.25 mmol) of Boc_2O. Yield: 0.14 g (0.27 mmol, 66%). Amorphous solid.

^{1}H NMR (300 MHz, $CDCl_3$, HSQC, COSY, rotamers) δ 3.44 – 3.32 (br m, 4H, 2 CH_2), 3.27 (t, *J* = 6.3 Hz, 8H, 4 CH_2), 2.76 (t, *J* = 5.2 Hz, 2H, CH_2), 2.59 (br t, *J* = 5.5 Hz, 2H, CH_2), 2.00 – 1.79 (br m, 2H, CH_2), 1.74 – 1.60 (br m, 2H, CH_2), 1.43 (s, 27H, 3 ^{t}Bu), 1.35 – 0.71 (br m, 1H, NH).

^{13}C NMR (76 MHz, $CDCl_3$) δ 156.4 (C=O), 155.6 (2 C=O), 79.6 and 79.3 (2 br, 3 *C*(Me)$_3$), 50.7, 50.1, 47.8, 46.8, 46.0 and 44.2 (6 br, 8 CH_2), 30.1 (br, 2 CH_2), 28.6 (3 C*(Me)$_3$*).

ESI-HRMS m/z: $[M+H]^+$ Calcd for $[C_{25}H_{49}N_4O_6]^+$ 501.3647; Found 501.3644.

NMR spectral data are in agreement with the literature.[10]

Tris-tert-butyl 1,4,7,10-tetraazacyclododecane-1,4,7-tricarboxylate[10]

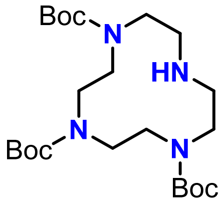

Prepared according to the general procedure from 0.11 g (0.64 mmol) of cyclen and 0.35 g (1.6 mmol) of Boc_2O. Yield: 0.25 g (0.52 mmol, 98%). Amorphous solid.

^{1}H NMR (300 MHz, $CDCl_3$, HSQC, COSY, rotamers) δ 3.69 – 3.52 (br m, 4H, 2 CH_2), 3.45 – 3.14 (br m, 8H, 4 CH_2), 2.91 – 2.78 (br m, 4H, 2 CH_2), 1.45 (s, 9H, ^{t}Bu), 1.43 (s, 18H, 2 ^{t}Bu), 1.21 – 0.71 (br m, 1H, NH).

^{13}C NMR (76 MHz, $CDCl_3$, HSQC, COSY, rotamers) δ 155.9 and 155.6 (2 br, 3 C=O), 79.6 and 79.3 (2 br, 3 *C*(Me)$_3$), 51.1, 49.6 and 46.1 (3 br, 8 CH_2), 28.8 (C*(Me)$_3$*), 28.6 (2 C*(Me)$_3$*).

ESI-HRMS m/z: $[M+H]^+$ Calcd for $[C_{23}H_{45}N_4O_6]^+$ 473.3334; Found 473.3324.

NMR spectral data are in agreement with the literature.[10]

General procedure for acylation/sulfonylation of tris-Boc-protected macrocyclic tetraamines

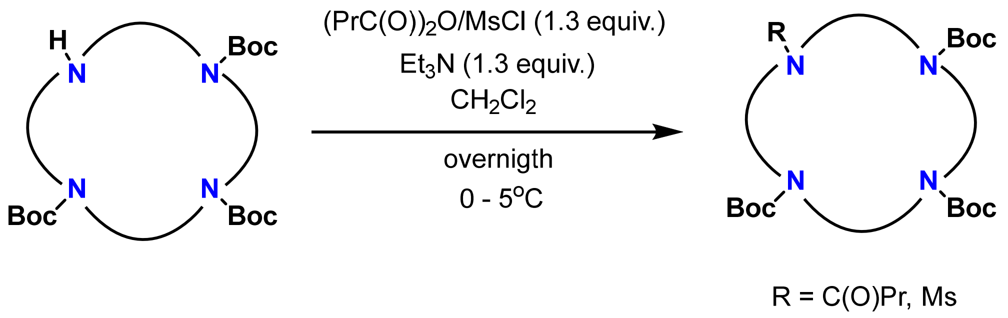

To a solution of a tris-Boc protected macrocyclic polyamine (1 equiv.) in CH_2Cl_2 (0.3 M) was added Et_3N (1.3 equiv.). Then, the corresponding anhydride/acyl chloride (1.3 equiv.) was added dropwise keeping the temperature of the reaction mixture below 5°C (ice bath). After the addition, the reaction mixture was allowed to warm to rt and kept overnight. The volatiles were removed under reduced pressure and the residue was subjected to a column chromatography on silica gel (Hex:EtOAc = 9:1→6:1→1:1→EtOAc).

Tris-*tert*-butyl 11-propionyl-1,4,8,11-tetraazacyclotetradecane-1,4,8-tricarboxylate

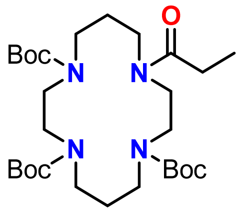

Prepared according to the general procedure from 57 mg (0.11 mmol) of tris-Boc-cyclam, 15 mg (21 μl, 0.15 mmol) of Et_3N and 19 mg (19 μl, 0.15 mmol) of propionic anhydride. Yield: 63 mg (0.11 mmol, 98%). Amorphous solid.

^{1}H NMR (300 MHz, $CDCl_3$, HSQC, rotamers) δ, ppm: 1.08 (t, *J* = 7.4 Hz, 3H, CH_2*CH_3*), 1.36 – 1.48 (m, 27H, 3 C(Me)$_3$), 1.60 – 1.80 (m, 4H, 2 CH_2), 2.28 (q, *J* = 7.4 Hz, 2H, *CH_2*CH_3), 3.20 – 3.51 (br m, 16H, 8 CH_2N).

^{13}C NMR (76 MHz, $CDCl_3$, HSQC, rotamers) δ, ppm: 9.6 ($COCH_2$*CH_3*), 26.1 (*CH_2*CH_3), 28.4 (2 CH_2), 28.5 (3 C*(Me)$_3$*), 46.2–49.5 (br, 8 CH_2N), 79.8 (3 *C*(Me)$_3$), 155.4 (C(O)O), 155.8 (C(O)O), 156.0 (C(O)O), 173.5 (*C*(O)Et).

ESI-HRMS m/z: $[M+H]^+$ Calcd for $[C_{28}H_{53}N_4O_7]^+$ 557.3909; Found 557.3905.

Tris-*tert*-butyl 11-(methylsulfonyl)-1,4,8,11-tetraazacyclotetradecane-1,4,8-tricarboxylate

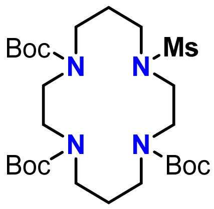

Prepared according to the general procedure from 57 mg (0.11 mmol) of tris-Boc-cyclam, 15 mg (21 µl, 0.15 mmol) of Et_3N and 17 mg (12 µl, 0.15 mmol) of methanesulfonyl chloride. Yield: 63 mg (0.11 mmol, 96%). Amorphous solid.

^1H NMR (300 MHz, $CDCl_3$, HSQC, rotamers) δ, ppm: 1.45 (s, 27H, 3 C*(Me)$_3$*), 1.68 – 1.92 (m, 4H, 2 CH_2), 2.78 (s, 3H, S(O)$_2$*Me*), 3.12 – 3.46 (br m, 16H, 8 CH_2N).

^{13}C NMR (76 MHz, $CDCl_3$, HSQC, rotamers) δ, ppm: 28.5 (2 CH_2), 28.6 (3 C*(Me)$_3$*), 35.4 (br, S(O)$_2$Me), 45.5–49.5 (br, 8 CH_2N), 79.8 (*C*(Me)$_3$), 80.0 (*C*(Me)$_3$), 80.0 (*C*(Me)$_3$), 155.8 (3 C=O).

ESI-HRMS m/z: $[M+H]^+$ Calcd for $[C_{26}H_{51}N_4O_8S]^+$ 579.3422; Found 579.3409.

Tris-*tert*-butyl 10-propionyl-1,4,7,10-tetraazacyclododecane-1,4,7-tricarboxylate

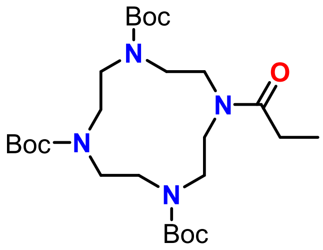

Prepared according to general procedure from 240 mg (0.51 mmol) of tris-Boc-cyclen, 67 mg (92 µl, 0.66 mmol) of Et_3N and 86 mg (85 µl, 0.66 mmol) of propionic anhydride. Yield: 246 mg (0.47 mmol, 92%). Amorphous solid.

^1H NMR (300 MHz, $CDCl_3$, HSQC, rotamers) δ, ppm: 1.10 (t, $J = 7.4$ Hz, 3H, CH_2*CH_3*), 1.43 (s, 18H, C(Me)$_3$), 1.44 (s, 9H, C(Me)$_3$), 2.29 (q, $J = 7.4$ Hz, 2H, *CH_2*CH_3), 3.18 – 3.65 (br m, 16H, 8 CH_2N).

^{13}C NMR (76 MHz, $CDCl_3$, HSQC, rotamers) δ, ppm: 9.6 (CH_2*CH_3*), 26.6 (C*H_2*CH_3), 28.5 (2 C*(Me)$_3$*), 28.6 (C*(Me)$_3$*), 49.7, 50.3, and 51.5 (3 br, 8 CH_2N), 80.2 (*C*(Me)$_3$), 80.3 (*C*(Me)$_3$), 80.4 (*C*(Me)$_3$), 155.5 (3 C(O)O) (amide carbon C(O)N was not observed due to broadening).

ESI-HRMS m/z: $[M+H]^+$ Calcd for $[C_{26}H_{49}N_4O_7]^+$ 529.3596; Found 529.3588.

General procedure for deprotection of Boc-groups

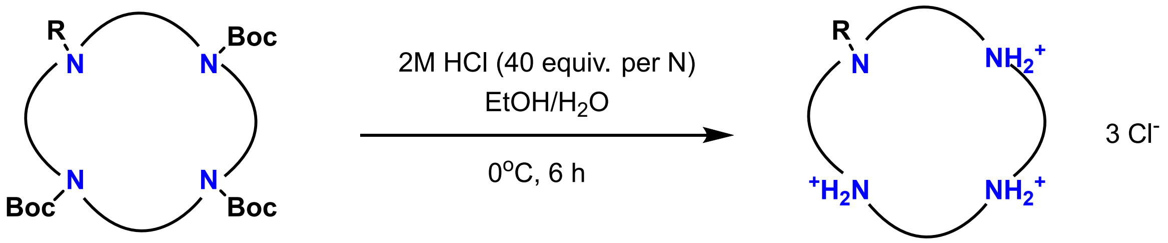

To a solution of a tris-Boc protected macrocyclic polyamine (1 equiv.) in EtOH (1 M) was added a 2M aqueous solution of HCl (excess, 50 equiv.) dropwise keeping the reaction temperature near 0°C. After the addition, the reaction mixture allowed to warm to rt and kept for 6 h at rt. The volatiles (*Caution! Dangerous vapors of HCl.*) were removed under reduced pressure. The residue was dried in a vacuum (ca. 0.5 Torr) until constant weight to give a tris-hydrochloride salt.

1-Propionyl-1,4,8,11-tetraazacyclotetradecane trihydrochloride

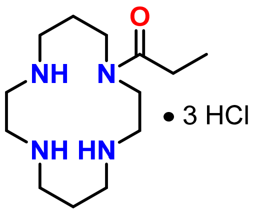

Prepared according to the general procedure from 63 mg (0.11 mmol) of tris-*tert*-butyl 11-propionyl-1,4,8,11-tetraazacyclotetradecane-1,4,8-tricarboxylate and 2.8 ml (50 equiv., 5.7 mmol) of 2M hydrochloric acid. Yield: 41 mg (0.11 mmol, 99%). Pale pink solid. Mp > 250 °C.

^{1}H NMR (300 MHz, D_2O, HSQC) δ, ppm: 0.97 (t, *J* = 7.4 Hz, 3H, $CH_2\mathit{CH_3}$), 1.77 – 2.10 (br m, 2H, CH_2), 2.10 – 2.28 (m, 2H, CH_2), 2.36 (q, *J* = 7.4 Hz, 2H, $\mathit{CH_2}CH_3$), 3.13 – 3.42 (br m, 8H, CH_2N), 3.42 – 3.62 (br m, 6H, CH_2N), 3.61 – 3.86 (m, 2H, $\mathit{CH_2}$NC(O)Et) (NH protons are not observed due to exchange with D_2O).

^{13}C NMR (76 MHz, D_2O, HSQC) δ, ppm: 8.6 ($CH_2\mathit{CH_3}$), 18.8 (CH_2), 23.8 (CH_2), 26.4 ($\mathit{CH_2}CH_3$), 37.9 (CH_2N), 38.7 (CH_2N), 41.3 (CH_2N), 41.6 (CH_2N), 41.9 (CH_2N), 42.7 (CH_2N), 43.0 (CH_2N), 46.4 (CH_2N), 178.6 (C=O).

FT-IR (KBr): 3481 (s, br), 2842 (s, br), 2098 (w), 2043 (w), 1963 (m), 1638 (s, sh), 1484 (s, sh), 1375 (s), 1338 (w), 1312 (s), 1252 (s), 1210 (s), 1186 (m), 1126 (m), 1066 (s), 1020 (m, sh), 926 (w), 890 (w, sh), 820 (w), 797 (w), 772 (m, sh), 613 (w, sh), 550 (m), 516 (w), 460 (w) cm^{-1}.

ESI-HRMS m/z: $[M–2HCl–Cl^-]^+$ Calcd for $[C_{13}H_{29}N_4O]^+$ 257.2336; Found 253.2337.

1-(Methylsulfonyl)-1,4,8,11-tetraazacyclotetradecane trihydrochloride

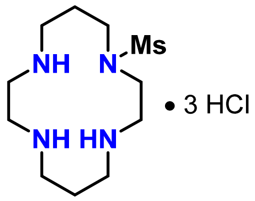

Prepared according to the general procedure from 156 mg (0.28 mmol) tris-*tert*-butyl 11-(methylsulfonyl)-1,4,8,11-tetraazacyclotetradecane-1,4,8-tricarboxylate and 6.7 ml (50 equiv., 13.5 mmol) of 2M hydrochloric acid. Yield: 99 mg (0.27 mmol, 97%). Pale pink solid. Decomp. temp. 245°C.

^{1}H NMR (300 MHz, D_2O, HSQC) δ, ppm: 2.06 – 2.20 (m, 2H, CH_2), 2.23 – 2.34 (m, 2H, CH_2), 3.11 (s, 3H, $S(O)_2Me$), 3.27 – 3.51 (m, 10H, 5 CH_2N), 3.57 (s, 4H, 2 CH_2N), 3.67 (t, J = 5.7 Hz, 2H, $\mathit{CH_2}$NMs) (NH protons are not observed due to exchange with D_2O).

^{13}C NMR (76 MHz, D_2O, HSQC) δ, ppm: 19.7 (CH_2), 24.7 (CH_2), 34.3 ($S(O)_2Me$), 38.9 (N$\mathit{CH_2}$$CH_2$N), 39.4 (N$CH_2$$\mathit{CH_2}$N), 42.2 ($CH_2N$), 42.7 ($CH_2N$), 43.6 ($CH_2N$), 45.5 ($CH_2N$), 46.9 ($\mathit{CH_2}$NMs), 48.3 ($CH_2N$).

FT-IR (KBr): 3420 (s, br), 3059 (s, br), 1947 (w), 1583 (s, sh), 1459 (s, sh), 1370 (s), 1340 (s, sh), 1213 (s, sh), 1147 (s, sh), 1097 (w), 1041 (s, sh), 967 (s), 914 (s, sh), 834 (w), 779 (s, sh), 735 (m), 697 (w), 661 (m), 525 (s, sh), 487 (w), 456 (w) cm^{-1}.

ESI-HRMS m/z: $[M–2HCl–Cl^-]^+$ Calcd for $[C_{11}H_{27}N_4O_2S]^+$ 279.1849; Found 279.1850.

1-Propionyl-1,4,7,10-tetraazacyclododecane trihydrochloride

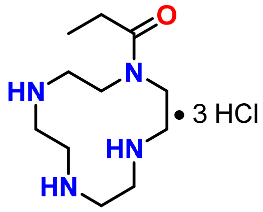

Prepared according to the general procedure from 248 mg (0.47 mmol) tris-*tert*-butyl 10-propionyl-1,4,7,10-tetraazacyclododecane-1,4,7-tricarboxylate and 11.8 ml (50 equiv., 23.5 mmol) of 2M hydrochloric acid. Yield: 157 mg (0.47 mmol, 99%). Pale pink solid. Mp > 250 °C.

^{1}H NMR (300 MHz, D_2O, HSQC) δ, ppm: 1.09 (t, J = 7.4 Hz, 3H, $CH_2\mathit{CH_3}$), 2.50 (q, J = 7.4 Hz, 2H, $\mathit{CH_2}CH_3$), 3.12 – 3.27 (m, 8H, 4 CH_2N), 3.27 – 3.39 (m, 4H, CH_2N), 3.57 – 3.84 (br m, 4H, $\mathit{CH_2}$NC(O)Et).

^{13}C NMR (76 MHz, D_2O, HSQC) δ, ppm: 8.5 ($CH_2\mathit{CH_3}$), 27.1 ($\mathit{CH_2}CH_3$), 43.5 ($\mathit{CH_2}CH_2$NC(O)Et, br), 43.9 ($\mathit{CH_2}CH_2$NC(O)Et), br), 44.5 (2 CH_2N), 45.7 ($\mathit{CH_2}$NC(O)Et and 2 CH_2N), 47.4 ($\mathit{CH_2}$NC(O)Et, br), 179.5 (C(O)).

FT-IR (KBr): 3433 (m, br), 3050 (s, br), 2068 (w), 1653 (s, sh), 1610 (s, sh), 1361 (s, sh), 1296 (s, sh), 1273 (m), 1211 (s, sh), 1131 (s), 1107 (m), 1078 (s, sh), 1006 (m), 956 (s), 910 (m), 859 (m), 815 (s), 786 (s), 759 (s), 594 (s, sh), 546 (s), 489 (s), 444 (w) cm^{-1}.

ESI-HRMS m/z: $[M–2HCl–Cl^-]^+$ Calcd for $[C_{11}H_{25}N_4O]^+$ 229.2023; Found 229.2017.

1.4 Synthesis of macrocyclic polyhydroxylamines and their derivatives

Dibenzoyl peroxide[11]

Into a round-bottom flask were placed a solution of a benzoyl chloride (1.62 ml, 1.96 g, 13.95 mmol, 1 equiv.) in 30 ml of methyl *tert*-butyl ether. To the resulting ice-bath cooled stirred solution was added 18.5% aqueous solution of H_2O_2 (1.92 g, 10.46 mmol, 0.75 equiv.) drop by drop, followed by pyridine (1.35 ml, 16.73 mmol, 1.2 equiv.). The reaction mixture was stirred at 0°C over 1 hour. After reaction completion, methyl *tert*-butyl ether (volume equal to the reaction mixture volume) was added. The organic phase was washed with 20 ml of 3% HCl aqueous solution, 3×20 ml saturated $NaHCO_3$ solution then with water till pH = 7. The organic phase was dried (Na_2SO_4) and evaporated under reduced pressure. The product was purified by column chromatography (Hex:EtOAc, 9:1) to give 1.42 g (84%, 5.87 mmol) of dibenzoyl peroxide as a white solid. R_f = 0.9 (Hex:EtOAc, 5:1). Mp 103−105°C.

^{1}H NMR (300 MHz, $CDCl_3$) δ 8.08 (d, *J* = 7.6 Hz, 4H, 4 *o*-Ph), 7.65 (t, *J* = 7.6 Hz, 2H, 2 *p*-Ph), 7.50 (t, *J* = 7.6 Hz, 4H, 4 *m*-Ph).

^{13}C NMR (76 MHz, $CDCl_3$) δ 163.1 (2 C=O), 134.4 (2 *p*-Ph), 129.8 and 128.9 (4 *o*-Ph and 4 *m*-Ph), 125.6 (2 *i*-Ph).

Physical properties and NMR spectral data are in agreement with to those given in the literature.[11]

General procedure for the synthesis of *N*-benzoyloxylated macrocyclic polyamines (method A). Into a round-bottom flask were placed a solution (25 mM) of a cyclic polyamine or its salt (1 equiv.) and Cs_2CO_3 (3×n equiv., n − number of NH units in the starting polyamine). To the resulting stirred suspension was added $(PhCOO)_2$ (2×n equiv.) followed by water (9×n equiv.). The reaction mixture was stirred at room temperature overnight. After reaction completion (TLC control), water (volume equal to the reaction mixture volume) was added. The crude product was extracted with CH_2Cl_2. The organic phase was washed with saturated NaCl solution, dried (Na_2SO_4) and evaporated under reduced pressure. The product was purified by column chromatography.

General procedure for the synthesis of *N*-benzoyloxylated macrocyclic polyamines (method B). Into a round-bottom flask were placed a solution (25 mM) of a cyclic polyamine or its salt (1 equiv.), Cs_2CO_3 (1 equiv.) and finely ground K_2CO_3 ([3×n−1], n − number of NH units in the starting polyamine). To the resulting stirred suspension was added $(PhCOO)_2$ (2×n equiv.) followed by water (20×n equiv., final concentration of M_2CO_3 – ca. 8 M). The reaction mixture was stirred at room temperature overnight. After reaction completion (TLC control), water (volume equal to the reaction mixture volume) was added. The crude product was extracted with CH_2Cl_2. The organic phase was washed with saturated NaCl solution, dried (Na_2SO_4) and evaporated under reduced pressure. The product was purified by column chromatography.

1,4,7-Tribenzoyloxy-1,4,7-triazacyclononane (tacn(OBz)$_3$)

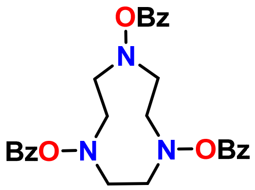

Prepared according to method A from 75 mg (0.31 mmol) of 1,4,7-triazacyclononane trihydrochloride (tacn•3HCl), 0.46 g (1.89 mmol) of dibenzoylperoxide, 1.23 g (3.77 mmol, 12 equiv.) of Cs_2CO_3 and 0.5 ml of water. The product was purified by column chromatography on silica gel (Hex:EtOAc, 9:1→3:1) to give 74 mg (0.15 mmol, 48%) of tacn(OBz)$_3$ as a white solid. R_f = 0.5 (Hex:EtOAc, 1:1). Mp 76–82°C (with decomp.).

Also, prepared according to method B from 50 mg (0.21 mmol) of 1,4,7-triazacyclononane trihydrochloride (tacn•3HCl), 0.31 g (1.26 mmol) of dibenzoylperoxide, 70 mg (0.21 mmol) Cs_2CO_3, 0.32 g (2.3 mmol) K_2CO_3 and 0.3 ml of water. The product was purified by column chromatography on silica gel (Hex:EtOAc, 9:1→3:1) to give 21 mg (21%, 0.043 mmol) of tacn(OBz)$_3$ as a white solid.

^{1}H NMR (300 MHz, $CDCl_3$) δ, ppm: 3.81 (s, 12H, CH_2), 7.46 (t, J = 7.6Hz, 6H, m-Ar), 7.59 (t, J = 7.3Hz, 3H, p-Ar), 8.05 (d, J = 7.2Hz, 6H, o-Ar).

^{13}C NMR (75 MHz, DEPT135, $CDCl_3$) δ, ppm: 57.0 (CH_2), 128.5 (CH, m-Ar), 129.4 (C, Ar), 129.6 (CH, o-Ar), 133.2 (CH, p-Ar), 164.8 (C=O).

ESI-HRMS m/z: $[M+H]^+$ Calcd for $[C_{27}H_{28}N_3O_6]^+$ 490.1966; Found 490.1973.

Crystals for X-Ray diffraction analysis were obtained by recrystallization from MeOH (CCDC 2265473).

1,4,7-Tribenzoyloxy-1,4,7-triazacyclodecane ([10]-ane[NOBz]$_3$)

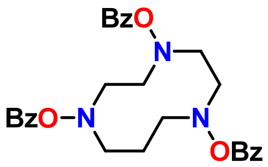

Prepared according to method A from 20 mg (0.079 mmol) of 1,4,7-triazacyclodecane trihydrochloride, 0.12 g (0.48 mmol) of dibenzoylperoxide, 0.31 g (0.71 mmol, 12 equiv.) of Cs_2CO_3 and 40 μl of water. The product was purified by column chromatography on silica gel (Hex:EtOAc, 9:1→3:1) to give 7 mg (0.014 mmol, 18%) of [10]-ane[NOBz]$_3$ as a white solid. R_f = 0.58 (Hex:EtOAc, 1:1). Mp 132–134°C (with decomp.).

^{1}H NMR (300 MHz, $CDCl_3$) δ, ppm: 1.66 – 1.74 (m, 2H, CH_2), 2.97 – 4.16 (br m, 12H, CH_2N), 7.39 – 7.51 (m, 6H, m-Ar), 7.57 – 7.64 (m, 3H, p-Ar), 8.02 – 8.07 (m, 6H, o-Ar).

^{13}C NMR (75 MHz, $CDCl_3$) δ, ppm: 24.4 (CH_2), 52.9 (CH_2N), 53.3 (CH_2N), 56.6 (CH_2N), 128.4 (m-Ar), 128.5 (m-Ar), 129.2 (o-Ar), 129.5 (o-Ar), 129.7 (C, Ar), 132.9 (p-Ar), 133.2 (p-Ar), 164.8 (C=O), 165.8 (C=O).

ESI-HRMS m/z: $[M+H]^+$ Calcd for $[C_{28}H_{30}N_3O_6]^+$ 504.2129; Found 504.2123.

1,4,8-Tribenzoyloxy-1,4,8-triazacycloundecane ([11]-ane[NOBz]$_3$)

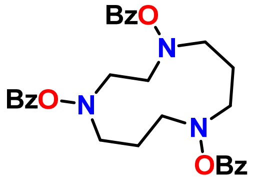

Prepared according to method A from 20 mg (0.075 mmol) of 1,4,8-triazacycloundecane trihydrochloride, 0.11 g (0.45 mmol) of dibenzoylperoxide, 0.29 g (0.9 mmol, 12 equiv.) of Cs_2CO_3 and 40 μl of water. The product was purified by column chromatography on silica gel (Hex:EtOAc, 9:1→3:1) to give 16 mg (0.031 mmol, 41%) of [11]-ane[NOBz]$_3$ as a white solid. R_f = 0.48 (Hex:EtOAc, 1:1). Mp 126–127°C (with decomp.).

^{1}H NMR (300 MHz, $CDCl_3$) δ, ppm: 1.97 (m, 4H, CH_2), 3.46 – 3.59 (br m, 12H, CH_2N), 7.41 – 7.49 (m, 6H, m-Ar), 7.53-7.61 (m, 3H, p-Ar), 7.99 – 8.11 (m, 6H, o-Ar).

^{13}C NMR (75 MHz, $CDCl_3$) δ, ppm: 24.4 (CH_2), 54.9 (CH_2N), 55.4 (CH_2N), 55.7 (CH_2N), 128.3 (CH, m-Ar), 128.4 (CH, m-Ar), 129.5 (C, CH, o-Ar), 129.6 (CH, o-Ar), 132.9 (CH, p-Ar), 133.0 (CH, p-Ar), 165.3 (C=O), 165.9 (C=O).

ESI-HRMS m/z: $[M+H]^+$ Calcd for $[C_{29}H_{32}N_3O_6]^+$ 518.2286; Found 518.2288.

1,5,9-Tribenzoyloxy-1,5,9-triazacyclododecane (tacd(OBz)$_3$)

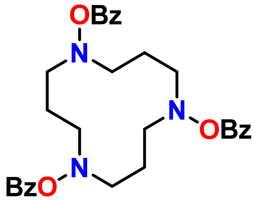

Prepared according to method B from 75 mg (0.27 mmol) of 1,5,9-triaazacyclododecane trihydrochloride (tacd•3HCl), 0.39 g (1.6 mmol) of dibenzoylperoxide, 0.3 g (2.1 mmol) of K_2CO_3, 0.09 g (0.27 mmol) of Cs_2CO_3 and 0.3 ml of water. The product was purified by column chromatography on silica gel (Hex:EtOAc, 9:1→4:1) to give 78 mg (0.15 mmol, 55%) of tacd(OBz)$_3$ as a solid white product. R_f = 0.45 (Hex:EtOAc, 1:1). Mp 62–64°C.

Also, prepared according to method A from 50 mg (0.18 mmol) of 1,5,9-triaazacyclododecane trihydrochloride (tacd•3HCl), 0.26 g (1.0 mmol) of dibenzoylperoxide, 0.7 g (2.1 mmol) of Cs_2CO_3 and 0.1 ml of water. The product was purified by column chromatography to give 49 mg (52%, 0.09 mmol) of tacd(OBz)$_3$ as a solid white product.

^{1}H NMR (300 MHz, $CDCl_3$) δ, ppm: 1.91 (m, 6H, CH_2), 3.02 – 3.83 (br m, 12H, CH_2N), 7.46 (t, J = 7.6 Hz, 6H, m-Ar), 7.58 (t, J = 7.3 Hz, 3H, p-Ar), 8.03 (d, J = 7.2 Hz, 6H, o-Ar).

^{13}C NMR (75 MHz, $CDCl_3$) δ, ppm: 22.6 (CH_2), 53.3 (CH_2N), 128.4 (CH, m-Ar), 129.4 (C and CH, o-Ar), 133.0 (CH, p-Ar), 165.3 (C=O).

ESI-HRMS m/z: $[M+H]^+$ Calcd for $[C_{30}H_{34}N_3O_6]^+$ 532.2442; Found 532.2445.

1,5,9-Tribenzoyloxy-1,5,9-triazacyclotridecane ([13]-ane[NOBz]$_3$)

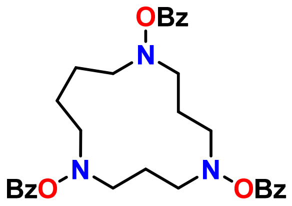

Prepared according to method A from 50 mg (0.12 mmol) of 1,5,9-triazacyclotridecane trihydrobromide, 0.17 g (0.7 mmol) of dibenzoylperoxide, 0.46 g (1.4 mmol, 12 equiv.) of Cs_2CO_3 and 55 µl of water. The product was purified by column chromatography on silica gel (Hex:EtOAc, 9:1→3:1) to give 36 mg (0.07 mmol, 57%) of [13]-ane[NOBz]$_3$ as a vitreous pale-yellow material. R_f = 0.46 (Hex:EtOAc, 1:1).

^{1}H NMR (300 MHz, HSQC, $CDCl_3$) δ, ppm: 1.63 (m, 4H, CH_2), 1.95 (m, 4H, CH_2), 3.04 – 3.72 (m, 12H, CH_2N), 7.38 – 7.49 (m, 6H, m-Ar), 7.49 – 7.61 (m, 3H, p-Ar), 7.97 – 8.06 (m, 6H, o-Ar).

^{13}C NMR (76 MHz, HSQC, $CDCl_3$) δ, ppm: 24.9 (CH_2), 25.5 (CH_2), 54.1 (CH_2N), 55.8 (CH_2N), 58.7 (CH_2N), 128.5 (m-Ar), 128.6 (m-Ar), 129.3 (C, i-Ar), 129.5 (o-Ar), 129.6 (o-Ar), 129.8 (C, i-Ar), 132.9 (p-Ar), 133.2 (p-Ar), 165.4 (C=O), 166.3 (C=O).

ESI-HRMS m/z: $[M+H]^+$ Calcd for $[C_{35}H_{36}N_3O_6]^+$ 546.2599; Found 546.2600.

1,4,8,11-Tetrabenzoyloxy-1,4,8,11-tetraazacyclotetradecane (cyclam(OBz)$_4$)

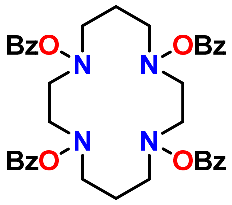

Prepared according to method B from 100 mg (0.5 mmol) of 1,4,8,11-tetraazacyclotetradecane (cyclam), 0.97 g (4 mmol) of dibenzoylperoxide, 0.76 g (5.5 mmol) of K_2CO_3, 0.16 g (0.5 mmol) of Cs_2CO_3 and 0.75 ml of water. The product was purified by column chromatography on silica gel (Hex:EtOAc, 9:1→1:1) to give 256 mg (0.38 mmol, 75%) of cyclam(OBz)$_4$ as a solid white product. For analytical purposes the product was recrystallized from MeOH. R_f = 0.3 (Hex:EtOAc, 1:1). Mp 150–153°C.

Also, prepared according to method A from 100 mg (0.5 mmol) of 1,4,8,11-tetraazacyclotetradecane (cyclam), 0.97 g (8.0 mmol) of dibenzoylperoxide, 1.95 g (12.0 mmol) of Cs_2CO_3 and 0.32 ml of water. The product was purified by column chromatography on silica gel (Hex:EtOAc 9:1→1:1) to give 180 mg (53%, 0.26 mmol) of cyclam(OBz)$_4$ as a white solid.

^{1}H NMR (300 MHz, HSQC, $CDCl_3$) δ, ppm: 1.67 (p, J = 5.6 Hz, 4H, CH_2), 3.04 – 4.00 (br m, 16H, CH_2N), 7.42 (t, J = 7.6 Hz, 8H, m-Ar), 7.54 (t, J = 7.4 Hz, 4H, p-Ar), 8.03 (d, J = 7.2 Hz, 8H, o-Ar).

^{13}C NMR (75 MHz, HSQC, $CDCl_3$) δ, ppm: 25.1 (CH_2), 56.3 ($\mathit{CH_2}CH_2\mathit{CH_2}N$), 57.3 ($CH_2N$), 128.4 (m-Ar), 129.5 (i-Ar), 129.6 (o-Ar), 132.9 (p-Ar), 165.2 (CO).

ESI-HRMS m/z: $[M+H]^+$ Calcd for $[C_{38}H_{41}N_4O_8]^+$ 681.2919; Found 681.2913.

Crystals for X-Ray diffraction analysis were obtained by recrystallization from MeOH (CCDC 2257251).

1-Benzoyl-4,8,11-tribenzoyloxy-1,4,8,11-tetraazacyclotetradecane (Bz-cyclam(OBz)$_3$)

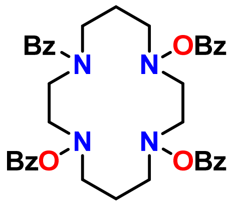

Bz-cyclam(OBz)$_3$ was obtained as a side product in the synthesis of cyclam(OBz)$_4$ by method A in the second fraction of column chromatography (EtOAc). Yield: 25 mg (7%, 0.04 mmol). Pale-yellow oil. R_f = 0.72 (Hex:EtOAc, 1:1).

^{1}H NMR (300 MHz, HSQC, $CDCl_3$) δ, ppm: 1.74 (m, 2H, CH_2), 1.79 – 2.24 (m, 2H, *CH_2*CH_2NCO), 2.72 – 3.61 (m, 12H, 6 CH_2NOCO), 3.62 – 4.05 (m, 4H, 2 CH_2NCO), 7.28 – 7.45 (m, 10H, 8 CH m-Ar and 2 CH o-Ar (NCO)), 7.52 (t, *J* = 7.5 Hz, 4H, 4 CH p-Ar), 7.73 – 8.16 (m, 6H, 3 CH o-Ar (OCO)).

^{13}C NMR (76 MHz, DEPT135, HSQC, $CDCl_3$) δ, ppm: 24.3 (CH_2), 26.7 (*CH_2*CH_2NCO), 43.6 (CH_2NCO), 48.7 (CH_2NCO), 54.6 (CH_2NOCO), 55.5 (CH_2NOCO), 56.5 (CH_2NOCO), 57.1 (CH_2NOCO), 60.7 (CH_2NOCO), 61.3 (CH_2NOCO), 126.4 (NC(O)Ph o-Ar), 128.3 (NOC(O)Ph m-Ar), 128.3 (NOC(O)Ph and NC(O)Ph m-Ar), 128.4 (NOC(O)Ph m-Ar), 129.0 (NOC(O)Ph i-Ar), 129.1 (NC(O)Ph i-Ar), 129.3 (NOC(O)Ph o-Ar), 129.4 (NOC(O)Ph o-Ar and NC(O)Ph p-Ar), 129.4 (NOC(O)Ph o-Ar), 132.9 (NOC(O)Ph p-Ar), 133.0 (NOC(O)Ph o-Ar), 133.1 (NOC(O)Ph o-Ar), 137.1 (NOC(O)Ph i-Ar), 165.0 (2 OC=O), 165.2 (OC=O), 172.1 (NC=O).

ESI-HRMS m/z: $[M+Na]^+$ Calcd for $[C_{38}H_{40}N_4O_7Na]^+$ 687.2795; Found 687.2792.

1,4,8,12-Tetrabenzoyloxy-1,4,8,12-tetraazacyclopentadecane ([15]-ane[NOBz]$_4$)

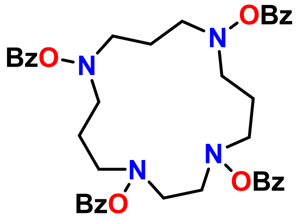

Prepared according to method A from 25 mg (0.12 mmol) of 1,4,8,12-tetraazacyclopentadecane, 0.23 g (0.9 mmol) of dibenzoylperoxide, 0.46 g (1.4 mmol, 12 equiv.) of Cs_2CO_3 and 75 µl of water. The product was purified by column chromatography on silica gel (Hex:EtOAc, 9:1→3:1) to give 35 mg (0.05 mmol, 43%) of [15]-ane[NOBz]$_4$ as a vitreous pale-yellow material. R_f = 0.49 (Hex:EtOAc, 1:1).

^{1}H NMR (300 MHz, $CDCl_3$) δ, ppm: 1.61 (p, *J* = 5.5 Hz, 2H, CH_2), 1.70 (p, *J* = 5.4 Hz, 4H, 2 CH_2), 2.81 – 3.95 (m, 16H, 4 CH_2N), 7.37 – 7.52 (m, 8H, m-Ar), 7.52 – 7.62 (m, 4H, p-Ar), 7.93 – 8.13 (m, 8H, o-Ar).

^{13}C NMR (76 MHz, $CDCl_3$) δ, ppm: 24.8 (CH_2), 25.2 (CH_2), 55.9 (CH_2N), 56.5 (CH_2N), 57.1 (CH_2N), 58.1 (CH_2N), 128.5 (m-Ar), 129.4 (i-Ar), 129.5 (i-Ar), 129.6 (o-Ar), 129.6 (o-Ar), 133.0 (p-Ar), 133.1 (p-Ar), 165.5 (C=O), 166.0 (C=O).

ESI-HRMS m/z: $[M+H]^+$ Calcd for $[C_{39}H_{43}N_4O_8]^+$ 695.3075; Found 695.3072.

1,5,9,13,17-Pentabenzoyloxy-1,5,9,13,17-pentaazacycloicosane ([20]-*ane*[NOBz]$_5$)

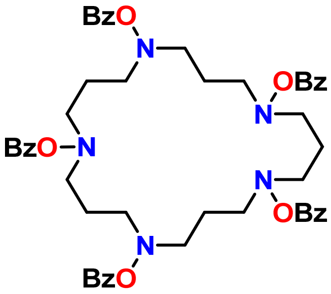

Prepared according to method B from 50 mg (0.07 mmol) of 1,5,9,13,17-pentaazacycloicosane pentahydrobromide, 0.18 g (0.73 mmol) of dibenzoylperoxide, 0.19 g (1.4 mmol) of K_2CO_3, 24 mg (0.07 mmol) of Cs_2CO_3 and 1.85 ml of water. The product was purified by column chromatography on silica gel (Hex:EtOAc, 9:1→1:1) to give 36 mg (0.04 mmol, 56%) of [20]-ane[NOBz]$_5$ as a vitreous pale-yellow material. R_f = 0.68 (Hex:EtOAc, 1:1).

Also, prepared according to method A from 100 mg (0.15 mmol) of 1,5,9,13,17-pentaazacycloicosane pentahydrobromide, 0.35 g of dibenzoylperoxide (1.45 mmol), 0.94 g of Cs_2CO_3 (2.9 mmol) and 0.12 ml of water. The product was purified by column chromatography on silica gel (Hex:EtOAc, 1:1) to give 60 mg (47%, 0.068 mmol) of [20]-ane[NOBz]$_5$ as a vitreous pale-yellow material.

^1H NMR (300 MHz, $CDCl_3$) δ, ppm: 1.84 (p, J = 6.5 Hz, 10H, CH_2), 3.29 (t, J = 6.6 Hz, 20H, CH_2N), 7.37 (t, J = 7.7 Hz, 10H, m-Ar), 7.47 – 7.59 (m, 5H, p-Ar), 7.87 – 8.08 (m, 10H, o-Ar).

^{13}C NMR (75 MHz, $CDCl_3$) δ, ppm: 24.4 (CH_2), 56.8 (CH_2N), 128.4 (m-Ar), 129.3 (i-Ar), 129.5 (o-Ar), 132.9 (p-Ar), 165.5 (CO).

ESI-HRMS m/z: $[M+H]^+$ Calcd for $[C_{50}H_{56}N_5O_{10}]^+$ 886.4022; Found 886.4021.

11-Methylsulfonyl-1,4,8-tribenzoyloxy-1,4,8,11-tetraazacyclotetradecane (Ms-cyclam(OBz)$_3$)

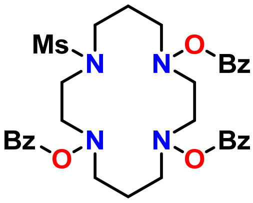

Prepared according to method B from 50 mg (0.13 mmol) of 1-methylsulfonyl-1,4,8,11-tetraazacyclotetradecane trihydrochloride, 0.19 g (0.77 mmol) of dibenzoylperoxide, 0.2 g (1.4 mmol) of K_2CO_3, 42 mg (0.13 mmol) of Cs_2CO_3 and 0.2 ml of water. The product was purified

by column chromatography on silica gel (Hex:EtOAc, 9:1→2:1) to give 50 mg (0.08 mmol, 61%) of Ms-cyclam(OBz)$_3$ as a vitreous pale-yellow material. R_f = 0.67 (Hex:EtOAc, 1:1).

^{1}H NMR (300 MHz, HSQC, $CDCl_3$) δ, ppm: 1.54 – 1.88 (m, 2H, CH_2), 2.06 (m, 2H, CH_2), 2.96 (s, 3H, CH_3(Ms)), 3.17 – 3.90 (m, 16H, 4 CH_2N), 7.29 – 7.53 (m, 6H, m-Ar), 7.53 – 7.69 (m, 3H, p-Ar), 7.93 – 8.17 (m, 6H, o-Ar).

^{13}C NMR (76 MHz, HSQC, $CDCl_3$) δ, ppm: 24.4 (CH_2), 27.5 (CH_2), 38.7 (CH_3), 46.9 (CH_2N), 47.1 (CH_2N), 55.3 (CH_2N), 55.6 (CH_2N), 55.9 (CH_2N), 56.4 (CH_2N), 57.1 (CH_2N), 60.5 (CH_2N), 128.5 (m-Ar), 128.5 (m-Ar), 129.1 (i-Ar), 129.1 (i-Ar), 129.2 (i-Ar), 129.5 (o-Ar), 129.5 (o-Ar), 129.5 (o-Ar), 133.1 (p-Ar), 133.2 (p-Ar), 165.1 (C=O), 165.5 (C=O), 165.6 (C=O).

ESI-HRMS m/z: $[M+H]^+$ Calcd for $[C_{32}H_{39}N_4O_8S]^+$ 639.2483; Found 639.2488.

11-Propionyl-1,4,8-tribenzoyloxy-1,4,8,11-tetraazacyclotetradecane (EtC(O)-*cyclam*(OBz)$_3$)

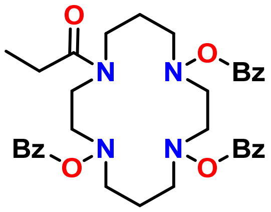

Prepared according to method B from 122 mg (0.34 mmol) of 1-propionyl-1,4,8,11-tetraazacyclotetradecane trihydrochloride, 0.49 g (2 mmol) of dibenzoylperoxide, 0.51 g (3.7 mmol) of K_2CO_3, 110 mg (0.34 mmol) of Cs_2CO_3 and 0.5 ml of water. The product was purified by column chromatography on silica gel (Hex:EtOAc, 9:1→3:1) to give 140 mg (0.23 mmol, 68%) of EtC(O)-cyclam(OBz)$_3$ as a vitreous pale-yellow material. R_f = 0.58 (Hex:EtOAc, 1:1).

^{1}H NMR (300 MHz, HSQC, $CDCl_3$) δ, ppm: 1.17 (t, J = 7.4 Hz, 3H, CH_3 (Et)), 1.64 – 1.78 (m, 2H, CH_2), 1.87 – 2.03 (m, 2H, CH_2), 2.42 (q, J = 7.4 Hz, 2H, CH_2 (Et)), 3.17 – 4.05 (m, 16H, 8 CH_2N), 7.44 (q, J = 7.3 Hz, 6H, CH (*m*-Ar)), 7.52 – 7.63 (m, 3H, CH (*p*-Ar)), 7.91 – 8.08 (m, 6H, CH (*o*-Ar).

^{13}C NMR (76 MHz, HSQC, $CDCl_3$) δ, ppm: 9.6 (CH_3 (Et)), 24.4 (CH_2), 26.2 (CH_2), 27.0 (CH_2 (Et)), 44.2 (CH_2N), 47.3 (CH_2N), 54.5 (CH_2N), 55.8 (CH_2N), 55.8 (CH_2N), 56.7 (CH_2N), 57.5 (CH_2N), 61.8 (CH_2N), 128.4 (CH, *m*-Ar), 128.5 (CH, *m*-Ar), 128.5 (CH, *m*-Ar), 129.1 (C, *i*-Ar), 129.3 (C, *i*-Ar), 129.4 (C, *i*-Ar), 129.4 (CH, *o*-Ar), 129.5 (CH, *o*-Ar), 129.5 (CH, *o*-Ar), 132.9 (CH, *p*-Ar), 133.1 (CH, *p*-Ar), 133.1 (CH, *p*-Ar), 164.9 (*C*(O)Ar), 165.1 (*C*(O)Ar), 165.3 (*C*(O)Ar), 174.1 (*C*(O)Et).

ESI-HRMS m/z: $[M+H]^+$ Calcd for $[C_{34}H_{41}N_4O_7]^+$ 617.2970; Found 617.2966.

11-Propionyl-1,4,7-tribenzoyloxy-1,4,7,10-tetraazacyclododecane (EtC(O)-cyclen(OBz)$_3$)

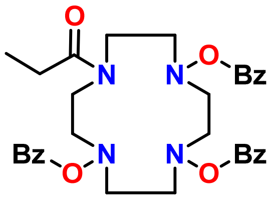

Prepared according to method A from 50 mg (0.15 mmol) 1-propionyl-1,4,7,10-tetraazacyclododecane trihydrochloride, 0.22 g (0.89 mmol) of dibenzoylperoxide, 580 mg (1.8 mmol) of Cs_2CO_3 and 0.2 ml of water. The product was purified by column chromatography on silica gel (Hex:EtOAc, 9:1→3:1) to give 52 mg (0.09 mmol, 60%) of EtC(O)-cyclen$(OBz)_3$ as a vitreous pale-yellow material. $R_f = 0.71$ (Hex:EtOAc, 1:1).

^{1}H NMR (300 MHz, HSQC, $CDCl_3$) δ, ppm: 0.94 (t, J = 7.3 Hz, 3H, CH_3 (Et)), 2.21 (q, J = 7.3 Hz, 2H, CH_2 (Et)), 3.08 – 4.29 (m, 16H, 4 CH_2N), 7.31 – 7.50 (m, 6H, m-Ar), 7.50 – 7.66 (m, 3H, p-Ar), 7.83 – 8.16 (m, 6H, o-Ar).

^{13}C NMR (76 MHz, HSQC, $CDCl_3$) δ, ppm: 9.3 (CH_3(COEt)), 26.2 (CH_2(COEt)), 45.0 (CH_2N), 46.2 (CH_2N), 53.6 (CH_2N), 54.5 (CH_2N), 56.8 (CH_2N), 57.2 (CH_2N), 58.2 (CH_2N), 59.0 (CH_2N), 128.5 (m-Ar), 128.6 (m-Ar), 128.7 (m-Ar), 129.3 (i-Ar), 129.3 (i-Ar), 129.3 (i-Ar), 129.5 (o-Ar), 129.6 (o-Ar), 129.6 (o-Ar), 133.2 (p-Ar), 133.3 (p-Ar), 133.4 (p-Ar), 164.9 (*C*(O)Ar), 164.9 (*C*(O)Ar), 165.9 (*C*(O)Ar), 174.2 (*C*(O)Et).

ESI-HRMS m/z: $[M+H]^+$ Calcd for $[C_{32}H_{37}N_4O_7]^+$ 589.2657; Found 589.2653.

1,4,7-Trihydroxy-1,4,7-triazacyclononane (tacn$(OH)_3$)

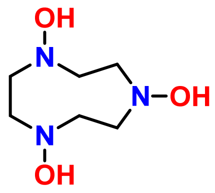

To a stirred solution of 1,4,7-tribenzoyloxy-1,4,7-triazacyclononane tacn$(OBz)_3$ (107 mg, 0.22 mmol, 1 equiv.) in $CHCl_3$ (3 ml) was added hydrazine hydrate (160 µl, 3.3 mmol, 15 equiv.) (*Caution: Hydrazine is both highly toxic and reactive and must be handled using appropriate protective equipment to prevent physical contact with either vapor or liquid*). The reaction mixture was stirred at ambient temperature under argon until full consumption of the starting material (overnight, TLC control). Then, the volatiles were removed under reduced pressure and the crude product was well-dried from water and residual hydrazine at ca. 0.5 Torr. The residue was purified by column chromatography on silica gel ($CHCl_3$:MeOH 20:1→5:1) to give 19 mg of mixture of benzohydrazide and product in 1.3:1 ratio. Yield of tacn$(OH)_3$: 24%.

^{1}H NMR (300 MHz, HSQC, D_2O) δ, ppm: 3.27 (s, 12H).

^{13}C NMR (76 MHz, HSQC, DEPT135, D_2O) δ, ppm: 57.8 (CH_2N).

ESI-HRMS m/z: $[M+H]^+$ Calcd for $[C_6H_{16}N_3O_3]^+$ 178.1186; Found 178.1182.

1,4,8,11-Tetrahydroxy-1,4,8,11-tetraazacyclotetradecane (cyclam$(OH)_4$)

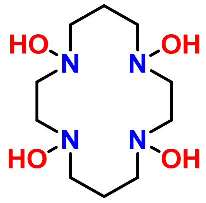

To a stirred solution of 1,4,8,11-tetrabenzoyloxy-1,4,8,11-tetraazacyclotetradecane cyclam(OBz)$_4$ (670 mg, 0.98 mmol, 1 equiv.) in $CHCl_3$ (17 ml) was added hydrazine hydrate (1.0 g, 20 mmol, 20 equiv.) (*Caution: Hydrazine is both highly toxic and reactive and must be handled using appropriate protective equipment to prevent physical contact with either vapor or liquid*). The reaction mixture was refluxed until full consumption of the starting material (4 h, TLC control) and formation of precipitate. The mixture was cooled to rt, and the volatiles removed under reduced pressure. The crude product was well dried from water and residual hydrazine at ca. 0.5 Torr. The residue was suspended in $CHCl_3$ (5 ml), filtered, washed with $CHCl_3$ (3×2 ml), and dried (0.5 Torr) until constant weight to give 150 mg (0.57 mmol, 58%) of cyclam(OH)$_4$ as a white solid. Mp = 210–213°C (with decomp.).

^{1}H NMR (300 MHz, D_2O, HSQC, COSY) δ, ppm: 1.62 – 1.84 (m, 2H, CH_2), 2.44 – 2.69 (m, 2H, CH_2), 2.80 – 2.97 (m, 4H, $\mathit{CH_2}CH_2\mathit{CH_2}N$), 3.13 – 3.36 (m, 8H, CH_2N), 3.39 – 3.62 (m, 4H, $\mathit{CH_2}CH_2\mathit{CH_2}N$).

^{13}C NMR (75 MHz, HSQC, D_2O) δ, ppm: 19.8 (CH_2), 55.9 ($\mathit{CH_2}CH_2\mathit{CH_2}N$), 61.0 ($CH_2N$).

FT-IR (KBr): 3450 (s, sh), 3152 (m, br), 2975 (s), 2929 (m, sh), 2849 (s, br), 1640 (w, br), 1450 (s, sh), 1353 (s, sh), 1310 (m), 1278 (m), 1248 (w, sh), 1159 (s), 1091 (s, sh), 985 (m), 943 (s), 913 (s), 875 (m), 843 (s), 758 (w), 720 (s), 616 (m), 566 (m), 533 (m), 484 (s, sh), 434 (s, sh) cm^{-1}.

Anal. Calcd for $C_{10}H_{24}N_4O_4$: C, 45.44; H, 9.15; N, 21.20. Found: C, 45.28; H, 8.98; N, 21.22.

ESI-HRMS m/z: $[M+H]^+$ Calcd for $[C_{10}H_{25}N_4O_4]^+$ 265.1870; Found 265.1866.

1,4,8,11-Tetrahydroxy-1,4,8,11-tetraazacyclotetradecane monohydrochloride (cyclam(OH)$_4$•HCl)

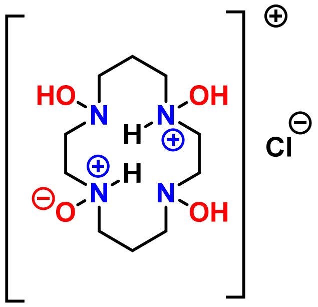

Mono-hydrochloride of cyclam(OH)$_4$ was prepared in the same manner as mentioned above with an exception that CH_2Cl_2 (24 ml) was used as a solvent and the reaction time was 8 h. Yield of cyclam(OH)$_4$•HCl: 178 mg (0.59 mmol, 42%) from 958 mg (1.41 mmol, 1 equiv.) of cyclam(OBz)$_4$ and 1.41 g of hydrazine hydrate (28 mmol, 20 equiv.). White solid. The product can be additionally purified by recrystallization from MeOH. Mp = 110°C (loss of methanol), 198–200°C.

^{1}H NMR (300 MHz, HSQC, D_2O) δ, ppm: 1.54 – 1.79 (m, 2H, CH_2), 2.42 – 2.64 (m, 2H, CH_2), 2.74 – 2.93 (m, 4H, $\mathit{CH_2}CH_2\mathit{CH_2}N$), 3.02 – 3.33 (m, 8H, CH_2N), 3.38 – 3.59 (m, 4H, $\mathit{CH_2}CH_2\mathit{CH_2}N$).

^{13}C NMR (75 MHz, HSQC, D_2O) δ, ppm: 19.4 (CH_2), 55.9 ($\mathit{CH_2}CH_2\mathit{CH_2}N$), 61.1 ($CH_2N$).

FT-IR (KBr): 3017 (s, br), 2339 (m, sh), 2116 (w, sh), 2047 (w), 1982 (w, sh), 1919 (w, br), 1734 (w, sh), 1574 (s, sh), 1505 (s), 1409 (s), 1298 (w), 1239 (s), 1118 (s), 1075 (s), 967 (s, sh), 509 (s), 471 (m), 442 (w) cm^{-1}.

Crystals of cyclam$(OH)_4$•HCl•MeOH for X-ray diffraction analysis crystals were obtained by slow vapor diffusion of Et_2O into the solution of cyclam$(OH)_4$•HCl in MeOH (CCDC 2265480).

1,4,8,11-Tetrahydroxy-1,4,8,11-tetraazacyclotetradecane dihydrobromide (cyclam(OH)$_4$•2HBr)

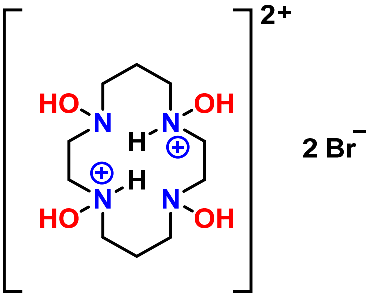

Methanol (2 ml) was added to cyclam$(OH)_4$ (10 mg, 0.038 mmol, 1 equiv.) and the suspension was heated until dissolution. To the resulting solution was added 60 µL (0.4 mmol, 10 equiv.) of aqueous HBr (36% w/w). The precipitate formed was filtered off and dried till constant weight under reduced pressure (0.5 Torr) to give 15 mg (0.035 mmol, 93%) of cyclam$(OH)_4$•2HBr as colorless crystals. Mp = 230–232°C (with decomp.).

Anal. Calcd for $C_{10}H_{26}Br_2N_4O_4$: C, 28.18; H, 6.15; N, 13.15. Found: C, 28.4; H, 5.9; N, 12.8.

Crystals for X-Ray diffraction analysis were obtained by recrystallization from MeOH (CCDC 2257250).

1,5,9,13,17-Pentahydroxy-1,5,9,13,17-pentaazacycloicosane ([20]-ane[NOH]$_5$)

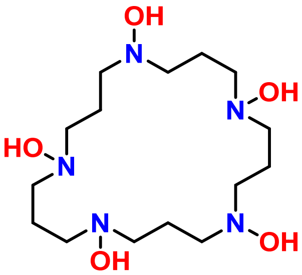

To a stirred solution of 1,5,9,13,17-pentabenzoyloxy-1,5,9,13,17-pentaazacycloicosane (65 mg, 0.07 mmol, 1 equiv.) in $CHCl_3$ (3 ml) was added hydrazine hydrate (90 mg, 1.8 mmol, 25 equiv.) (*Caution: Hydrazine is both highly toxic and reactive and must be handled using appropriate protective equipment to prevent physical contact with either vapor or liquid*). The reaction mixture was refluxed until full consumption of the starting material (4 h, TLC control) and the formation of precipitate. The mixture was cooled to rt, and volatiles removed under reduced pressure. Crude product was well-dried from water and residual hydrazine at ca. 0.5 Torr. The residue was suspended in $CHCl_3$ (2 ml), filtered, washed with $CHCl_3$ (3×1 ml), and dried (0.5

Torr) until constant weight to give 19 mg (0.05 mmol, 71%) of [20]-ane[NOH]$_5$ as a white solid product. Mp = 141−143°C.

^{1}H NMR (300 MHz, HSQC, D_2O) δ, ppm: 1.95 (br m, 10H, CH_2), 2.89 (br m, 20H, CH_2N).

^{13}C NMR (76 MHz, HSQC, D_2O) δ, ppm: 22.7 (CH_2), 23.0 (CH_2), 23.3 (CH_2), 23.5 (CH_2), 24.2 (CH_2), 58.0 (CH_2N).

FT-IR (KBr): 3245 (s), 3141 (m), 3044 (br m), 2958 (w), 2925 (m), 2851 (m sh), 2697 (br m), 2579 (m), 1633 (m sh), 1577 (m sh), 1496 (s), 1377 (m sh), 1241 (m), 1104 (s sh), 965 (s), 835 (w), 503 (m sh) cm^{-1}.

ESI-HRMS m/z: $[M+H]^+$ Calcd for $[C_{15}H_{36}N_5O_5]^+$ 366.2711; Found 366.2701.

1.5 Deprotection of cyclam(OBz)$_4$: optimization study

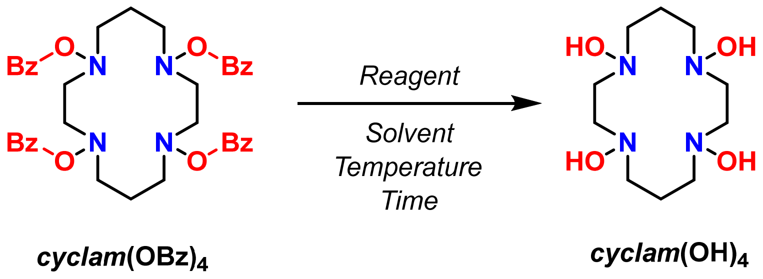

Typical procedure: To a solution of cyclam(OBz)$_4$ in the solvent indicated in Table S1 was added the corresponding basic reagent and stirred at the indicated temperature until full consumption of the starting material (TLC control). The mixture was cooled to room temperature (if needed), and volatiles were removed in a vacuum. The residue was washed with a cold MeOH–MeCN mixture (1:6) to remove most of inorganic impurities. Unless stated otherwise, the residue was analyzed by ^{1}H NMR with 1,4-benzoquinone as an internal standard in D_2O. Results are summarized in Supplementary Table 1.

Supplementary Table 1. Deprotection of cyclam(OBz)$_4$: optimization study

Entry	Reagent	Equiv.	Solvent	Time, h	Temp.	Yield, %
1	$LiOH•H_2O$	4	MeOH	8	reflux	20[a]
2	K_2CO_3	8	MeOH	8	rt	18[a]
3	NH_3 (23% aqueous w/w)	120	MeOH	24	rt	traces[a]
4	$NH_2NH_2•H_2O$	20	$CHCl_3$	6	reflux	58[b]
5	$NH_2NH_2•H_2O$	20	CH_2Cl_2	6	reflux	42[b,c]

[a] Yield was determined by ^{1}H NMR with internal standard (1,4-benzoquinone) in D_2O. [b] Yield determined for isolated product. [c] Yield for mono-hydrochloride of cyclam(OBz)$_4$.

1.6 Synthesis and characterization of d-metal complexes of macrocyclic polyhydroxylamines

Synthesis of [Ni_2(μ-Cl)(μ-O_2CPh)(tacn$(OH)_3)_2Cl_2$]

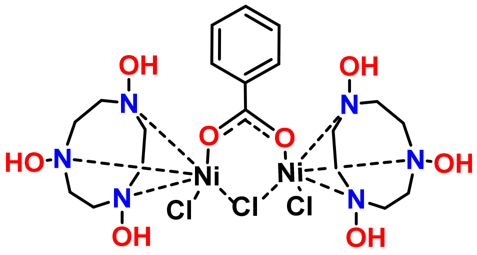

To a methanol suspension of tacn$(OBz)_3$ (17 mg, 0.034 mmol, 1 equiv.) was added a solution of $NiCl_2$•$6H_2O$ (8 mg, 0.034 mmol, 1 equiv.) in MeOH (1 ml). The mixture was stirred for 1 hour at rt. Solvent was removed under reduced pressure and the residue was dried in a vacuum. The product was isolated by recrystallization from MeOH to give 10 mg (0.014 mmol, 84%) of [Ni_2(μ-Cl)(μ-O_2CPh)(tacn$(OH)_3)_2Cl_2$] as a marine-blue diamond-shaped crystals. Decomp. temp. > 250°C.

FT-IR (KBr): 3381 (s, sh), 3287 (s), 2929 (s, sh), 2876 (s), 1599 (s), 1564 (s), 1458 (s), 1401 (s, sh), 1263 (s, sh), 1147 (s, sh), 1086 (m), 1014 (s), 963 (s), 914 (s), 806 (s, sh), 727 (s), 676 (m), 584 (s), 459 (m, sh) cm^{-1}.

Anal. Calcd for $C_{20}H_{38}Cl_3N_6Ni_2O_8$: C, 32.64; H, 5.05; N, 12.02. Found: C, 32.49; H, 5.09; N, 12.10.

Crystals for X-ray diffraction analysis were obtained by recrystallization from MeOH (CCDC 2257253).

Synthesis of [Zn(tacn$(OH)_3)_2$]$(NO_3)_2$

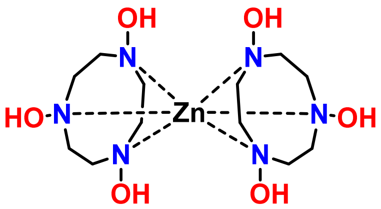

To a suspension of tacn$(OBz)_3$ (86 mg, 0.18 mmol, 1 equiv.) in methanol/isopropanol (1:1, 6 ml) was added a solution of $Zn(NO_3)_2$•$6H_2O$ (52 mg, 0.18 mmol, 1 equiv.) in MeOH (2 ml). The mixture was stirred at rt ca. 14 hours to become a clear solution. The volatiles were removed under reduced pressure. The resulting crude complex was triturated with *i*-PrOH (3×4 ml) and dried in a vacuum (0.5 Torr) until constant weight to give 26 mg (0.04 mmol, 48%) of [Zn(tacn$(OH)_3)_2$]$(NO_3)_2$•$2H_2O$•$^1/_6$$PhCO_2{}^iPr$ as yellowish crystals.

^1H NMR (300 MHz, HSQC, D_2O) δ, ppm: 3.15 – 3.33 (m, 6H, CH_2N), 3.36 (s, 2H, CH_2N), 3.38 – 3.56 (m, 10H, CH_2N), 3.69 – 3.86 (m, 6H, CH_2N).

^{13}C NMR (76 MHz, HSQC, D_2O) δ, ppm: 55.5 (CH_2N), 56.3 (CH_2N).

FT-IR (KBr): 3296 (m, br), 3118 (w), 2929 (w), 2888 (w), 2400 (w, br), 1766 (w), 1641 (m, sh), 1564 (w), 1480 (s), 1378 (s, sh), 1314 (m), 1269 (m, sh), 1147 (m), 1085 (m), 1041 (w), 1011 (m), 966 (m), 912 (m), 830 (m), 791 (s), 724 (w), 674 (w, br), 584 (s, sh), 455 (w) cm^{-1}.

ESI-HRMS m/z: $[M{-}2NO_3]^{2+}$ Calcd for $[C_{12}H_{30}N_6O_6Zn]^{2+}$ 209.0754; Found 209.0773.

Anal. Calcd for $[Zn(tacn(OH)_3)_2](NO_3)_2{\bullet}2H_2O{\bullet}{}^1/_6PhCO_2{}^iPr$: C, 27.03; H, 5.98; N, 18.45. Found: C, 26.65; H, 5.46; N, 18.31.

Crystals of $[Zn(tacn(OH)_3)_2](NO_3)_2$ for X-ray diffraction analysis were obtained by a slow vapor diffusion of diethyl ether into the methanol solution (CCDC 2257254).

Synthesis of $Cu(cyclam(OH)_4)Cl_2$

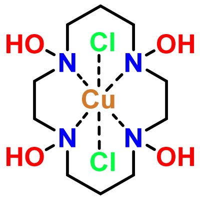

To a solution of $cyclam(OH)_4$ (20 mg, 76 μmol, 1 equiv.) in boiling *i*-PrOH (ca. 4 ml) was added a solution of $CuCl_2{\bullet}2H_2O$ (13 mg, 76 μmol, 1 equiv.) in *i*-PrOH (1 ml). The resulting hot solution was transferred into a Petri dish and the solvent was slowly evaporated under gentle heating (45°C) until crystals were formed. Then, heating plate was removed and the rest of the solvent was allowed to evaporate at ambient temperature. The residual solid was washed twice with *i*-PrOH (1.5 ml) and centrifuged. The resulting purple crystalline complex was dried under reduced pressure (c.a. 0.5 Torr) until constant weight. Yield: 24 mg (60 μmol, 80%). Decomp. temp. 212°C.

FT-IR (KBr): 3447 (w, br), 3176 (s, br, sh), 2935 (s), 2847 (s), 2709 (w, br), 1633 (w, br), 1441 (s, sh), 1391 (m), 1365 (w), 1299 (w), 1240 (w), 1142 (w), 1092 (m), 1038 (s), 999 (w), 924 (s), 874 (m), 821 (m), 751 (w), 666 (m), 546 (s), 508 (m, sh), 431 (w) cm^{-1}.

UV-Vis (H_2O, c = 5 mM) λ, nm: 294 (ε = 2560 $M^{-1}cm^{-1}$), 524 (ε = 170 $M^{-1}cm^{-1}$).

ESI-HRMS m/z: $[M{-}2Cl{-}H]^+$ Calcd for $[C_{10}H_{23}N_4O_4Cu]^+$ 326.1010; Found 326.1022.

Anal. Calcd for $C_{10}H_{24}Cl_2N_4O_4Cu$: C, 30.12; H, 6.07; N, 14.05. Found: C, 30.22; H, 6.18; N, 14.07.

Crystals for X-ray diffraction analysis were obtained by simple recrystallization from H_2O (CCDC 2257248).

Synthesis of $Zn(cyclam(OH)_4)Cl_2$

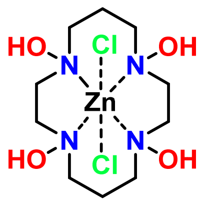

To a solution of $cyclam(OH)_4$ (10 mg, 38 μmol, 1 equiv.) in boiling *i*-PrOH (ca. 2 ml) was added a solution of $ZnCl_2$ (5 mg, 38 μmol, 1 equiv.) in *i*-PrOH (1 ml). The resulting hot solution was transferred into a Petri dish and the solvent was slowly evaporated under gentle heating (45°C) until crystals were formed. Then, heating plate was removed and the rest of the solvent was allowed to evaporate at ambient temperature. The residual solid was washed twice with cooled (ca. 15°C) *i*-PrOH (1.5 ml) and centrifuged. The resulting white crystalline complex was dried under reduced pressure (c.a. 0.5 Torr) until constant weight. Yield: 8 mg (20 μmol, 53%). Decomp. temp. 242°C.

^1H NMR (300 MHz, D_2O, 315K) δ, ppm: 2.03 – 2.46 (m, 4H, CH_2), 2.94 – 3.14 (m, 2H, $CH_2CH_2\mathit{CH_2}N$), 3.14 – 3.33 (m, 2H, $CH_2CH_2\mathit{CH_2}N$), 3.33 – 3.67 (m, 8H, $N\mathit{CH_2CH_2}N$), 3.67 – 3.96 (m, 4H, $CH_2CH_2\mathit{CH_2}N$). ^{13}C NMR could not be recorded due to a low solubility of the complex.

FT-IR (KBr): 3251 (br, s, sh), 2933 (m), 2840 (br, m), 1611 (w), 1456 (s, sh), 1389 (w), 1346 (w), 1293 (w), 1241 (w), 1172 (w), 1141 (w), 1090 (m), 1041 (s), 920 (s), 893 (w), 856 (w), 816 (m), 744 (w), 636 (m, sh), 538 (s, sh), 490 (m), 431 (w) cm^{-1}.

ESI-HRMS m/z: $[M–2Cl–H]^+$ Calcd for $[C_{10}H_{23}N_4O_4Zn]^+$ 327.1005; Found 327.1012.

Anal. Calcd for $C_{10}H_{24}Cl_2N_4O_4Zn$: C, 29.98; H, 6.04; N, 13.99. Found: C, 29.65; H, 6.21; N, 13.70.

Crystals for X-ray diffraction analysis were obtained by the following procedure: to a solution of $cyclam(OH)_4$ (20 mg, 0.076 mmol, 1 equiv.) in EtOH (4 ml) was added a suspension of $ZnCl_2$ (10 mg, 0.075 mmol, 1 equiv.) in EtOH (1 ml). The mixture was stirred for 1 hour at rt. The resulting solution was filtered, concentrated in a vacuum, and the residue was dissolved in MeOH (2 ml). Vapor diffusion of diethyl ether into this solution gave pale-yellow needle crystals of $Zn(cyclam(OH)_4)Cl_2$, which were used for X-ray analysis (CCDC 2257252).

Synthesis of $Mn(cyclam(OH)_4)Cl_2$

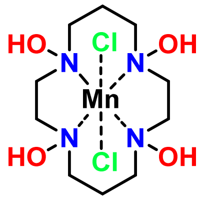

Methanol (3 ml) was added to $cyclam(OH)_4$ (15 mg, 57 μmol, 1 equiv.) and the suspension was heated until dissolution. To the resulting solution was added a solution of $MnCl_2$•$4H_2O$ (11 mg, 0.057 mmol, 1 equiv.) in MeOH (1 ml) and the mixture was stirred for 1 hour at rt. The resulting solution was filtered and concentrated under reduced pressure. The residue was dried in a vacuum (ca. 0.5 Torr). Crystallization of the crude complex by a slow vapor diffusion of diethyl

ether into the methanol solution produced pale-yellow cubic crystals, which were collected and dried in a vacuum (0.5 Torr) until constant weight. Yield: 8 mg (0.02 mmol, 36%). Decomp. temp. 209-211°C.

FT-IR (KBr): 3281 (s), 2972 (w), 2927 (s), 2828 (m, sh), 2677 (w), 1633 (w, br), 1451 (s), 1383 (m), 1342 (m), 1291 (m), 1242 (m), 1137 (m), 1079 (m), 1038 (s), 964 (w), 915 (s), 883 (w), 839 (m), 810 (s), 736 (w), 624 (s), 530 (s, sh), 479 (m), 426 (m) cm^{-1}.

ESI-HRMS m/z: $[M–2Cl–H]^+$ Calcd for $[C_{10}H_{23}N_4O_4Mn]^+$ 318.1094; Found 318.1084.

Anal. Calcd for $C_{10}H_{24}Cl_2N_4O_4Mn$: C, 30.78; H, 6.20; N, 14.36. Found: C, 30.46; H, 5.99; N, 14.55.

Pale-yellow cubic crystals for X-ray diffraction analysis were obtained by slow vapor diffusion of diethyl ether into a methanol solution (CCDC 2265474).

Synthesis of Mn(cyclam(OH)$_4$)Br$_2$•1.33 cyclam(OH)$_4$

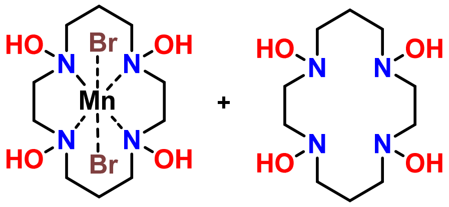

Methanol (2 ml) was added to cyclam(OH)$_4$ (10 mg, 38 μmol, 1 equiv.) and the suspension was heated until dissolution. To the resulting solution was added a solution of $MnBr_2$ (8 mg, 38 μmol, 1 equiv.) in methanol (1 ml). The mixture was stirred for 1 hour at rt. Pale-yellow cubic crystals of Mn(cyclam(OH)$_4$)Br$_2$•1.33 cyclam(OH)$_4$ were obtained by a slow diffusion of diethyl ether into the resulting solution. Yield: 13 mg (96%).

FT-IR (KBr): 3271 (br, s, sh), 2927 (s, sh), 2795 (br, s), 2708 (br, s), 2636 (br, s), 1606 (s), 1572 (s, sh), 1501 (s), 1449 (s, sh), 1393 (s), 1345 (w), 1297 (m), 1215 (w, sh), 1158 (s, sh), 1080 (s, sh), 962 (s), 915 (s), 878 (w), 840 (w), 811 (w), 738 (w, sh), 586 (m, sh), 521 (s, sh), 480 (m), 427 (m) cm^{-1}.

Crystals of Mn(cyclam(OH)$_4$)Br$_2$•1.33 cyclam(OH)$_4$ for X-ray diffraction analysis were obtained by slow vapor diffusion of diethyl ether into a methanol solution (CCDC 2257249).

Synthesis of Ni(cyclam(OH)$_4$)(NO$_3$)$_2$

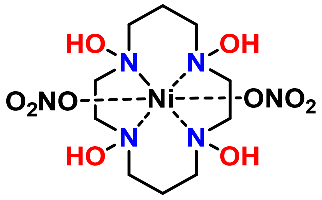

To a solution of cyclam(OH)$_4$ (10 mg, 38 μmol, 1 equiv.) in boiling *i*-PrOH (ca. 4 ml) was added a solution of $Ni(NO_3)_2•6H_2O$ (11 mg, 38 μmol, 1 equiv.) in *i*-PrOH (1 ml). The resulting hot solution was transferred into a Petri dish and the solvent was slowly evaporated under gentle heating (45°C) until orange crystals were formed. Then, heating plate was removed and rest of

the solvent was allowed to evaporate at ambient temperature. The residual solid was washed twice with a cooled (15°C) *i*-PrOH (1 ml) and centrifuged. The resulting orange crystalline complex was dried under reduced pressure (c.a. 0.5 Torr) until constant weight. Yield: 5 mg (11 µmol, 30%).

NMR spectra show the presence of paramagnetic and diamagnetic species in solution (D_2O) attributed to octahedral Ni(cyclam(OH)$_4$)(NO$_3$)$_2$ complexes (several conformers) and square-planar Ni(cyclam(O$^-$)(OH)$_3$)(NO$_3$) complex. Upon addition of KOH (c.a. 40 equiv.) disappearing of paramagnetic signals is observed with an increase of signals related to diamagnetic Ni(cyclam(O$^-$)(OH)$_3$)(NO$_3$).

^{1}H NMR (300 MHz, D_2O) δ -2.05 – -0.31 (br, Ni(cyclam(OH)$_4$)(NO$_3$)$_2$), 0.96 – 3.94 (m, Ni(cyclam(O$^-$)(OH)$_3$)(NO$_3$)), 5.25 – 7.26 (br, Ni(cyclam(OH)$_4$)(NO$_3$)$_2$), 12.28 – 14.11 (br, Ni(cyclam(OH)$_4$)(NO$_3$)$_2$), 14.33 – 16.08 (br), 37.35 – 45.49 (br, Ni(cyclam(OH)$_4$)(NO$_3$)$_2$).

UV-Vis (H_2O, c = 5 mM) λ, nm: 243 (ε = 2900 $M^{-1}cm^{-1}$), 297 (ε = 1600 $M^{-1}cm^{-1}$), 441 (ε = 100 $M^{-1}cm^{-1}$).

FT-IR (KBr): 3502 (w, br), 3287 (m, sh), 2938 (w, sh), 2801 (w), 1633 (w, br), 1503 (m), 1453 (m, sh), 1385 (s), 1283 (m, sh), 1141 (w), 1097 (w), 1040 (m, sh), 919 (w), 894 (w), 820 (w), 627 (w), 541 (w, sh), 489 (w) cm^{-1}.

ESI-HRMS m/z: $[M{-}2NO_3{-}H]^+$ Calcd for $[C_{10}H_{23}N_4O_4Ni]^+$ 321.1067; Found 321.1067.

Anal. Calc for Ni(cyclam(OH)$_4$)(NO$_3$)$_2$•1.25H_2O: C, 25.58; H, 5.69, N, 17.9. Found: C, 25.18; H, 5.29; N, 18.10.

Orange diamond-shaped crystals for X-ray diffraction analysis were obtained by slow vapor diffusion of diethyl ether into a solution of Ni(cyclam(OH)$_4$)(NO$_3$)$_2$ in MeOH (CCDC 2265481).

Synthesis of Ni(cyclam(OH)$_4$)(ClO$_4$)$_2$

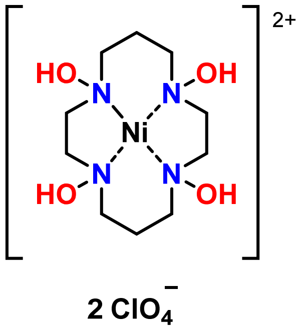

Methanol (2 ml) was added to cyclam(OH)$_4$ (10 mg, 38 µmol, 1 equiv.) and the suspension was heated until dissolution. To the resulting solution was added a solution of Ni(ClO$_4$)$_2$•6H_2O (14 mg, 0.038 mmol, 1 equiv.) in MeOH (1 ml). The mixture was stirred for 15 minutes at rt. Solvent was removed under reduced pressure and the residue was dried in a vacuum. The resulting crude complex was triturated with EtOH/CH_3CN (1:15) mixture and then with CH_2Cl_2, and dried in a vacuum (0.5 Torr) until constant mass to give 12 mg (0.023 mmol, 60%) of Ni(cyclam(OH)$_4$)(ClO$_4$)$_2$ as a orange crystals. ^{1}H NMR spectra of Ni(cyclam(OH)$_4$)(ClO$_4$)$_2$ exhibit a similar paramagnetic/diamagnetic pattern as Ni(cyclam(OH)$_4$)(NO$_3$)$_2$.

UV-Vis (H_2O, *c* = 10 mM) λ, nm: 304 (*ε* = 1400 $M^{-1}cm^{-1}$), 453 (*ε* = 108 $M^{-1}cm^{-1}$).

FT-IR (KBr): 3405 (s, br), 2951 (m, sh), 2796 (m, sh), 2018 (w, br), 1604 (m, sh), 1446 (m), 1389 (w), 1293 (w, sh), 1152 (s, sh), 1074 (s, sh), 942 (m), 876 (w, sh), 631 (s), 548 (w), 518 (w), 480 (w) cm^{-1}.

ESI-HRMS m/z: $[M–H–2ClO_4]^+$ Calcd for $[C_{10}H_{23}N_4O_4Ni]^+$ 321.1067; Found 321.1058.

Synthesis of $Ni(cyclam(O^-)(OH)_3)(ClO_4)$•$Ni(cyclam(OH)_4)(ClO_4)_2$•MeOH

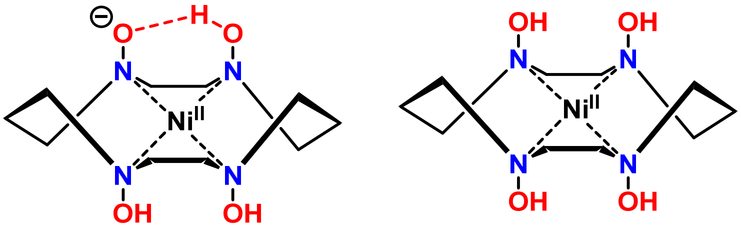

ClO4- MeOH 2 ClO4-

Methanol (2 ml) was added to $cyclam(OH)_4$ (10 mg, 0.038 mmol, 1 equiv.) and the suspension was heated until dissolution. To the resulting solution was added a solution of $Ni(ClO_4)_2$•$6H_2O$ (14 mg, 0.038 mmol, 1 equiv.) in MeOH (1 ml). The resulting yellow-colored mixture was stirred for 15 minutes at rt. Then, KOH pillet (4 mg, 0.076 mmol, 2 equiv.) was added resulting in the appearance of dark-orange color and the formation of white precipitate. The mixture was cooled to ca. 5°C and kept for 15 minutes. The precipitate was removed by filtration; solution was centrifuged to remove the rest of the precipitate. Upon evaporation of solvent, crystals were formed on the top of flask in a thin layer of solvent, which were taken for X-ray analysis. The solution was concentrated under reduced pressure. The residue was dissolved in 1 ml of MeOH and subjected to a slow vapor diffusion of Et_2O to this solution. The resulting crystalline material was collected and dried in a vacuum (0.5 Torr) to give 5 mg (0.023 mmol, 27%) of $Ni(cyclam(OH)_4)(ClO_4)_2$•$Ni(cyclam(OH)_3(O^-))(ClO_4)$•MeOH as an orange solid.

FT-IR (KBr): 3459 (s, br), 3116 (m, br), 2933 (m, sh), 2857 (w), 1711 (m), 1600 (m, sh), 1448 (s), 1362 (w, sh), 1286 (m, sh), 1148 (m), 1086 (s, br), 1009 (m, sh), 950 (w), 918 (m), 881 (w), 849 (w), 783 (w), 746 (w), 626 (s), 521 (w, sh), 475 (w), 449 (w) cm^{-1}.

ESI-HRMS m/z: $[M–ClO_4]^+$ Calcd for $[C_{10}H_{23}N_4O_4Ni]^+$ 321.1067; Found 321.1064.

Orange crystals of $Ni(cyclam(OH)_4)(ClO_4)_2$•$Ni(cyclam(OH)_3(O^-))(ClO_4)$•MeOH for X-ray diffraction analysis were obtained from the reaction mixture as described above (CCDC 2271901).

Synthesis of Ni(*cyclam(O$^-$)*(OH)$_3$)(NO$_3$)•Ni(*cyclam*(OH)$_4$)(NO$_3$)$_2$

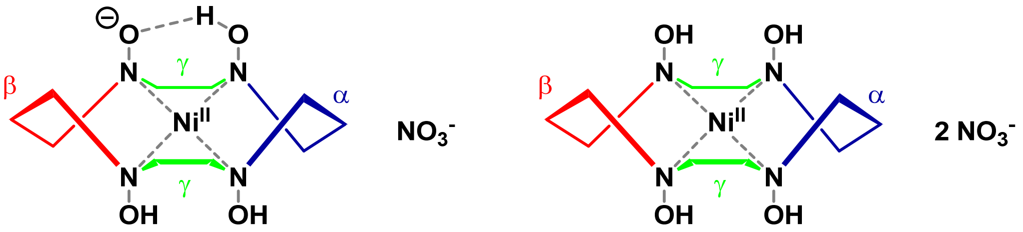

Methanol (3 ml) was added to cyclam(OH)$_4$ (15 mg, 0.057 mmol, 1 equiv.) and the suspension was heated until dissolution. To the resulting solution was added a solution of $Ni(NO_3)_2 \cdot 6H_2O$ (17 mg, 0.057 mmol, 1 equiv.) in MeOH (1 ml) followed by KOH (13 mg, 0.23 mmol, 4 equiv.). The mixture was stirred for 1 hour at rt and solvent was removed under reduced pressure to dryness. The residue was dissolved in MeOH (2 ml), filtered from insoluble impurities. Solvent was evaporated in a vacuum, and the crude complex was washed with CH_2Cl_2/CH_3CN (7:1) mixture and dried in a vacuum (0.5 Torr) until constant weight. Yield: 15 mg (69%). For elemental analysis, the complex was triturated several times with isopropanol.

^{1}H NMR (300 MHz, D_2O, HSQC, COSY) δ, ppm: 1.55 – 1.82 (m, 2H, CH_2 α), 1.82 – 2.09 (m, 2H, CH_2 β), 2.14 – 2.26 (m, 2H, CH_2N α), 2.26 – 2.37 (m, 2H, CH_2N γ), 2.37 – 2.48 (m, 1H, CH_2N β), 2.48 – 2.62 (m, 2H, CH_2N γ), 2.97 – 3.14 (m, 2H, CH_2N α), 3.14 – 3.25 (m, 2H, CH_2N β), 3.27 – 3.52 (m, 3H, CH_2N γ), 3.52 – 3.71 (m, 1H, CH_2N γ), 3.74 – 3.91 (m, 1H, CH_2N β).

^{13}C NMR (76 MHz, D_2O, HSQC) δ, ppm: 20.9 (CH_2 α), 21.3 (CH_2 β), 54.7 (CH_2N α), 55.0 (CH_2N α), 59.4 (CH_2N β), 61.2 (CH_2N γ), 62.5 (CH_2N γ), 63.5 (CH_2N β), 63.6 (CH_2N γ), 64.3 (CH_2N γ).

FT-IR (KBr): 3453 (s, br), 2930 (m, sh, br), 2854 (w), 2789 (w), 2394 (m), 2350 (w), 2283 (w, br), 1765 (w), 1707 (m), 1664 (w, sh), 1591 (s), 1418 (s, sh), 1375 (s, sh), 1281 (w), 1084 (w, sh), 1010 (w), 920 (m), 826 (m), 764 (w, sh), 658 (w, sh), 569 (w, sh), 457 (w, sh) cm^{-1}.

UV-Vis (H_2O, c = 1 mM) λ, nm: 306 (ε = 8400 $M^{-1}cm^{-1}$), 453 (ε = 1300 $M^{-1}cm^{-1}$).

ESI-HRMS m/z: $[M–NO_3]^+$ Calcd for $[C_{10}H_{23}N_4O_4Ni]^+$ 321.1067; Found 321.1070.

Anal. Calc for Ni(cyclam(O$^-$)(OH)$_3$)(NO$_3$)•Ni(cyclam(OH)$_4$)(NO$_3$)$_2$•6^{i}PrOH: C, 38.27; H, 8.11, N, 12.92. Found: C, 38.99; H, 7.91; N, 13.47.

1.7 Synthesis and characterization of nickel(II)-*tacn* complexes

Synthesis of $[Ni_2(\mu\text{-}Cl)_2(tacn)_2Cl_2]$ and $[Ni(tacn)_2]Cl_2$ for X-ray diffraction analysis

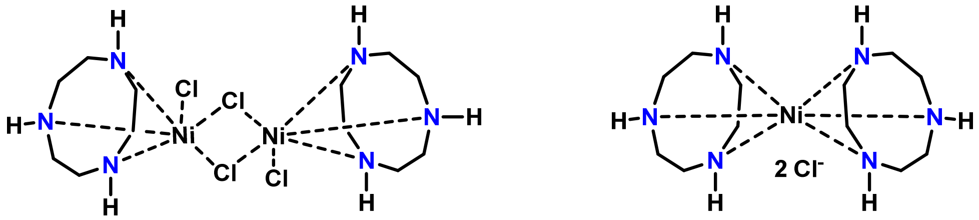

$NiCl_2$•$6H_2O$ (28 mg, 0.12 mmol, 1 equiv.), 2,4,9-triazaadamantane-2,4,7,9-tetraol[12] (24 mg, 0.12 mmol, 1 equiv.) and tacn•3HCl (30 mg, 0.12 mmol, 1 equiv.) were solved in 1 ml of MeOH. Triethylamine (0.11 ml, 0.62 mmol) was added and mixture was stirred at room temperature under air for 1 hour. Subsequent vapor diffusion of Et_2O into this solution gave pink crystals of $[Ni(tacn)_2]Cl_2$ and green of $[Ni_2(\mu\text{-}Cl)_2(tacn)_2Cl_2]$, which were collected and separated mechanically to give 15 mg (23%) of $[Ni_2(\mu\text{-}Cl)_2(tacn)_2Cl_2]$ (CCDC 2259686) and 7 mg (11%) of $[Ni(tacn)_2]Cl_2$ (CCDC 2259687), which were used for X-ray diffraction analysis.

Synthesis of $[Ni_2(\mu\text{-}Cl)_2(tacn)_2Cl_2]$. $NiCl_2$•$6H_2O$ (28 mg, 0.12 mmol, 1 equiv.) and tacn•3HCl (30 mg, 0.12 mmol, 1 equiv.) were dissolved in MeOH (1 ml) and triethylamine (0.11 ml, 0.62 mmol) was added. The mixture was stirred at room temperature for 1 hour. Subsequent vapor diffusion of Et_2O into the resulting solution produced green crystals of $[Ni_2(\mu\text{-}Cl)_2(tacn)_2Cl_2]$, which were collected from mother liquor and dried. Yield: 14 mg (32%). Mp = 233°C (with decomp.).

FT-IR (KBr): 3292 (s), 1672 (m), 1651 (m), 1416 (m), 1359 (m), 1233 (m), 1097 (m), 1021 (w), 952 (m), 752 (s), 569 (w), 474 (w), 416 (w).

Anal. Calcd for $C_{12}H_{30}Cl_4N_6Ni_2$: C, 27.85; H, 5.84; N, 16.24. Found: C, 27.45; H, 6.01; N, 16.20.

Synthesis of $[Ni(tacn)_2]Cl_2$. To a stirred solution of tacn•3HCl (48 mg, 0.2 mmol, 2 equiv.) in EtOH (2 ml) was added KOH (34 mg, 0.6 mmol, 6 equiv.). After 30 min, a solution of $NiCl_2$ (13 mg, 0.1 mmol, 1 equiv.) in EtOH (3 ml) was added. The resulting mixture was kept at rt for 1 hour and centrifuged. A clear solution was separated from an undissolved material, kept overnight at rt and concentrated in a vacuum. The residue was washed with a small portion of wet EtOH (0.6 ml). The resulting pink crystals were collected and dried until constant weight to give 8 mg (17%) of $[Ni(tacn)_2]Cl_2$•$5H_2O$.

ESI-HRMS m/z: $[M–2Cl]^{2+}$ Calcd for $[C_{12}H_{30}N_6Ni]^{2+}$ 158.0937; Found 158.0944.

Anal. Calcd for $C_{12}H_{30}Cl_2N_6Ni$•$5H_2O$: C, 30.15; H, 8.43; N, 17.58. Found: C, 29.63; H, 8.27; N, 17.18.

1.8 Catalytic aerobic oxidation studies

Synthesis of *N'*-phenylpropionohydrazide (NPPH)[13]

To a stirred solution of $PhNHNH_2$ (4.3 g, 39.7 mmol, 1 equiv.) in pyridine (24 ml) was added propionyl chloride (4.05 g, 43.7 mmol, 1.1 equiv.) dropwise at 0°C. The mixture was allowed to warm to room temperature and stirred overnight. Then, water (25 ml) was added to the resulting mixture. The formed precipitate was filtered, washed with water, dried and then recrystallized from MeOH to give 4.5 g (27.4 mmol, 96%) of *N'*-phenylpropionohydrazide as white crystals. Mp = 158 – 160°C. Two conformers in DMSO-d_6, ratio 7 : 1.

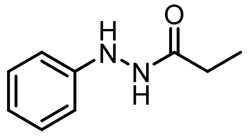

^{1}H NMR (300 MHz, DMSO-d_6, HSQC, major conformer) δ 9.58 (d, J = 3.0 Hz, 1H, NH), 7.64 (d, J = 3.0 Hz, 1H, NH), 7.13 (dd, J = 8.6, 7.0 Hz, 2H, 2 m-CH_{Ph}), 6.71 (d and m, J = 7.0 Hz, 3H, 2 o-CH_{Ph} and p-CH_{Ph}), 2.18 (q, J = 7.6 Hz, 2H, CH_2), 1.07 (t, J = 7.6 Hz, 3H, CH_3).

^{1}H NMR (300 MHz, DMSO-d_6, HSQC, minor conformer) δ 8.89 (s, 1H), 7.90 (s, 1H), 7.18 (t, J = 7.5 Hz, 2H, 2 m-CH_{Ph}), 6.71 (m, 3H, 2 o-CH_{Ph} and p-CH_{Ph}), 2.24 (q, J = 7.5 Hz, 2H, CH_2), 0.96 (t, J = 7.5 Hz, 3H, CH_3).

^{13}C NMR (76 MHz, DMSO-d_6, HSQC, major conformer) δ 172.8 (C=O), 149.5 (C_{Ph}), 128.7 (2 m-CH_{Ph}), 118.4 (p-CH_{Ph}), 112.1 (2 o-CH_{Ph}), 26.7 (CH_2), 9.9 (CH_3).

^{13}C NMR (76 MHz, DMSO-d_6, HSQC, minor conformer) δ 178.2 (C=O), 148.9 (C_{Ph}), 129.0 (2 m-CH_{Ph}), 118.8 (p-CH_{Ph}), 111.7 (2 o-CH_{Ph}), 24.0 (CH_2), 8.8 (CH_3).

ESI-HRMS m/z: $[M+H]^+$ Calcd for $[C_9H_{13}N_2O]^+$ 165.1022; Found 165.1024.

Physical properties and NMR spectral data are in agreement with to those given in the literature.[13]

General procedure for catalytic aerobic homo-coupling of NPPH to *N*',*N*'-diphenylpropionohydrazide DPPH

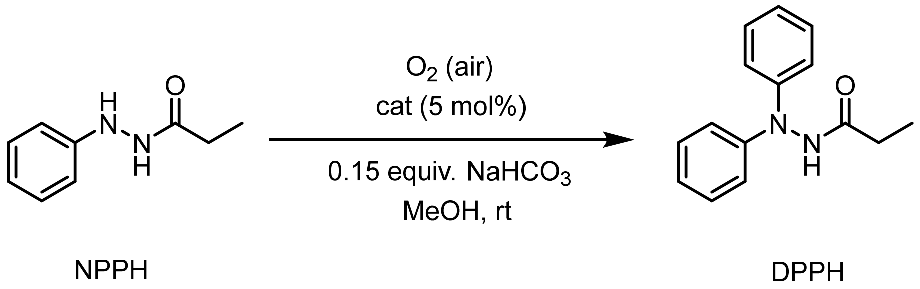

Into a round-bottom flask connected with an air-filled balloon were placed a solution of *N*'-phenylpropionohydrazide NPPH (100 mg, 0.61 mmol, 1 equiv.) in MeOH (4 ml), $NaHCO_3$ (8 mg, 0.09 mmol, 0.15 equiv.) and the corresponding complex (0.03 mmol, 0.05 equiv.). The mixture was stirred under air for 24 hours and periodic sampling was performed (by removing small portions of the solution). The samples were analyzed by ^{1}H NMR with internal standard (1,1,2,2-tetrachloroethane) to determine the product yield. The results are summarized in Supplementary Table 2.

Supplementary Table 2. Monitoring of catalytic aerobic oxidation of *N*'-phenylpropionohydrazide NPPH

Time of reaction, h	NMR yield of *N*',*N*'-diphenylpropionohydrazide (DPPH), %	
	Cu(cyclam)Cl_2	Cu(cyclam(OH)$_4$)Cl_2
0	0	0
1	0.5	16
2	4	44
3,5	9	56
6	11	68
9	17	81
16	17	83
24	24	90

N',N'-Diphenylpropionohydrazide DPPH (preparative synthesis from NPPH)

Into a round-bottom flask connected with an air-filled balloon were placed a solution of *N'*-phenylpropionohydrazide NPPH (25 mg, 0.15 mmol, 1 equiv.) in MeOH (1 ml), $NaHCO_3$ (2 mg, 0.025 mmol, 0.15 equiv.) and Cu(cyclam(OH)$_4$)Cl_2 (3 mg, 0.0075 mmol, 0.05 equiv.). After stirring for 24 hours, the solution was concentrated under reduced pressure. The residue was dissolved in $CHCl_3$, filtered through Celite and concentrated under reduced pressure. The residue was subjected to a standard aqueous work-up with brine (10 ml) and CH_2Cl_2 (3×15 ml). The combined organic extracts were dried with anhydrous Na_2SO_4 and evaporated under reduced pressure. The resulting crude product was purified by a column chromatography on silica gel (Hex:EtOAc 4:1 as eluent) to give 16 mg (0.066 mmol, 87%) of DPPH as a white solid. Mp = 215°C. Two conformers: ratio 6 : 1 (in DMSO-d_6), ratio 1.5 : 1 (in $CDCl_3$).

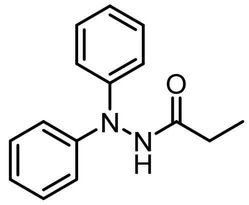

^{1}H NMR (300 MHz, DMSO-d_6, HSQC, major conformer) δ 10.45 (s, 1H, NH), 7.34 – 7.19 (m, 2H, 4 m-CH_{Ph}), 7.06 (d, J = 7.6 Hz, 2H, 4 o-CH_{Ph}), 6.97 (t, J = 7.3 Hz, 1H, 2 p-CH_{Ph}), 2.22 (q, J = 7.6 Hz, 2H, CH_2), 1.07 (t, J = 7.6 Hz, 3H, CH_3).

^{1}H NMR (300 MHz, DMSO-d_6, HSQC, minor conformer) δ 9.92 (s, 1H, NH), 7.40 – 7.29 (m, 2H, 4 m-CH_{Ph}), 7.09 (d, J = 7.6 Hz, 2H, 4 o-CH_{Ph}), 6.97 (t, J = 7.3 Hz, 1H, 2 p-CH_{Ph}), 2.25 (q, J = 7.4 Hz, 2H, CH_2), 0.90 (t, J = 7.4 Hz, 3H, CH_3).

^{13}C NMR (76 MHz, DMSO-d_6, HSQC, major conformer) δ 172.4 (C=O), 145.8 (2 C_{Ph}), 129.0 (4 m-CH_{Ph}), 122.0 (2 p-CH_{Ph}), 118.6 (4 o-CH_{Ph}), 26.5 (CH_2), 9.5 (CH_3).

^{13}C NMR (76 MHz, D DMSO-d_6, HSQC, minor conformer characteristic signals) δ 129.3 (4 m-CH_{Ph}), 122.9 (2 p-CH_{Ph}), 119.0 (4 o-CH_{Ph}), 24.4 (CH_2), 8.4 (CH_3).

ESI-HRMS m/z: $[M+H]^+$ Calcd for $[C_{15}H_{17}N_2O]^+$ 241.1335; Found 241.1341.

Physical properties and NMR spectral data are in agreement with to those given in the literature.[14]

General procedure for aerobic catalytic thiol oxidation

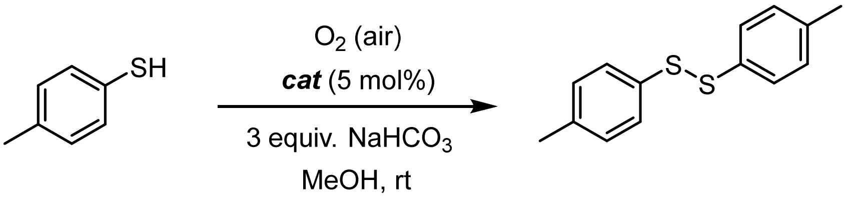

Into a round-bottom flask connected with an air-filled balloon were placed a solution of *p*-thiocresol (50 mg, 0.040 mmol, 1 equiv.) in MeOH (5 ml, 75 mM), $NaHCO_3$ (101 mg, 1.2 mmol, 3 equiv.) and the corresponding complex (0.02 mmol, 0.05 equiv.). The mixture was stirred under air for 25 hours and periodic sampling was performed (by removing small portions of the solution). The sample solution was decanted from insoluble material; the required amount of internal standard (1,1,2-trichloroethylene) was added and ^{1}H NMR spectra was recorded to determine the product yield. The results are summarized in Supplementary Table 3.

Supplementary Table 3. Monitoring of catalytic aerobic oxidation of *p*-thiocresol.

	**NMR yield of *p,p'*-ditolyl disulfide, %**	
**Time of reaction, h**	**Cu(cyclam)Cl$_2$**	**Cu(cyclam(OH)$_4$)Cl$_2$**
0	0	0
1	9	67
2	11	78
3	13	82
5	15	91
9	23	93
15	60	100
25	100	100

1,2-Di-*p*-tolyldisulfane (*p,p'*-ditolyl disulfide). For characterization purposes the title compound was purified by crystallization of crude product from hexane. Mp = 43–46°C.

^{1}H NMR (300 MHz, $CDCl_3$) δ 7.41 (d, J = 8.1 Hz, 4H, 4 CH_{Ar}), 7.12 (d, J = 8.1 Hz, 4H, 4 CH_{Ar}), 2.34 (s, 6H, 2 CH_3).

^{13}C NMR (76 MHz, $CDCl_3$) δ 137.6 (2 C_{Ar}), 134.0 (2 C_{Ar}), 129.9 (4 CH_{Ar}), 128.7 (4 CH_{Ar}), 21.2 (2 CH_3).

MS (EI): m/z = 246 ($[M]^{+\bullet}$).

Physical properties and NMR spectra are in accordance with lit. data.[15]

Dioxygen reactivity of deprotonated Cu(cyclam(OH)$_4$)Cl$_2$ complex (UV-Vis monitoring)

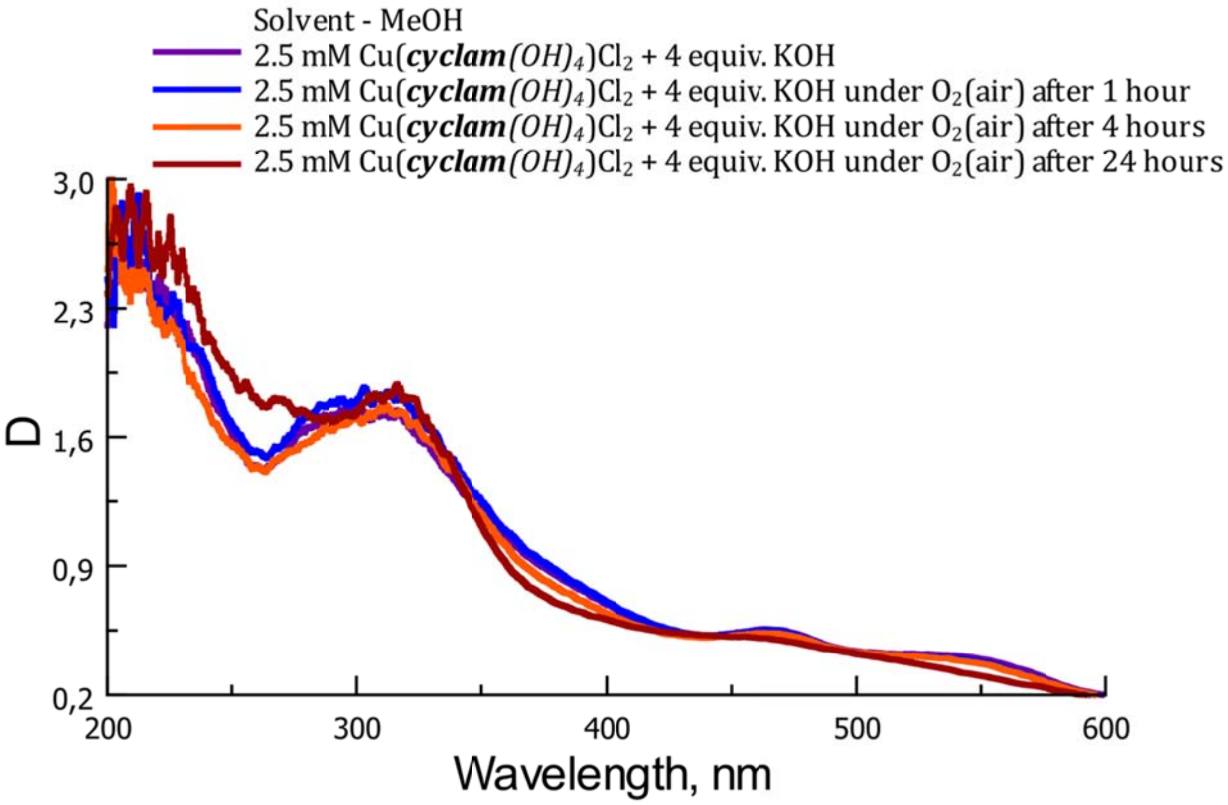

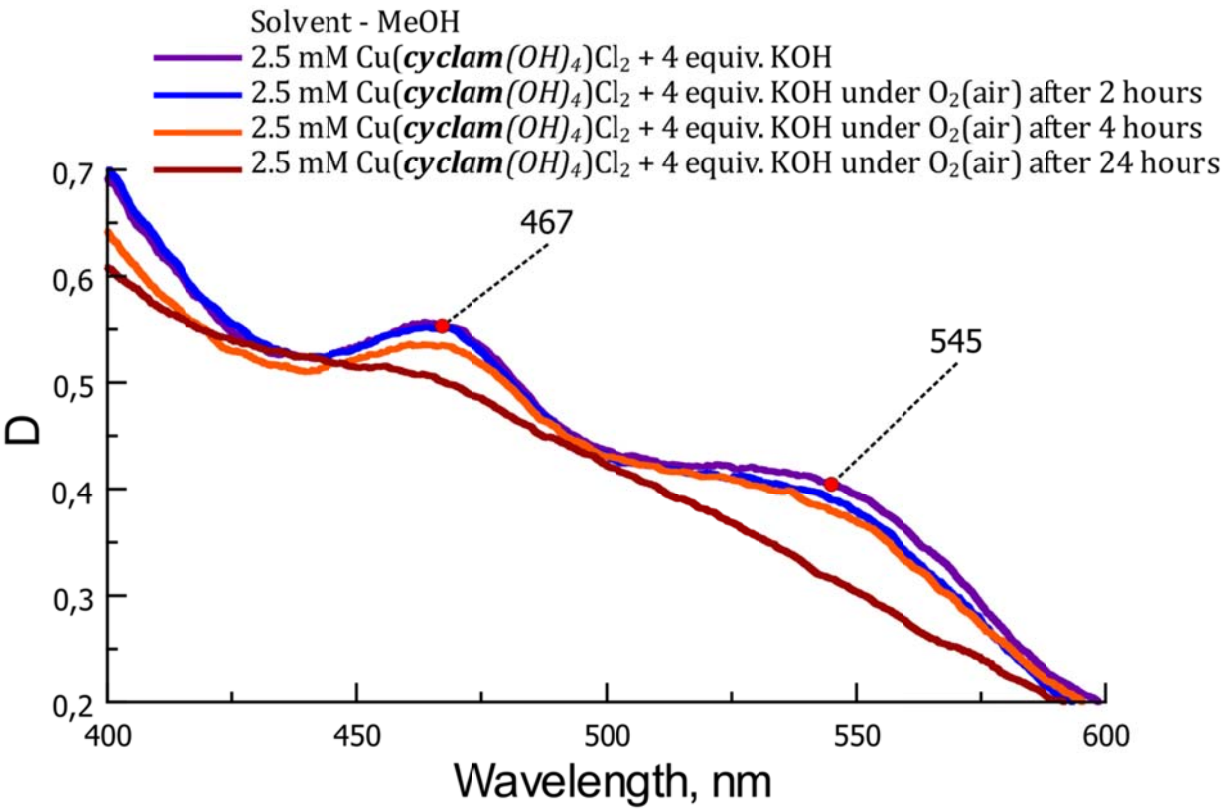

Reaction of deprotonated Cu(cyclam(OH)$_4$)Cl$_2$ complex with *p*-thiocresol (UV-Vis monitoring)

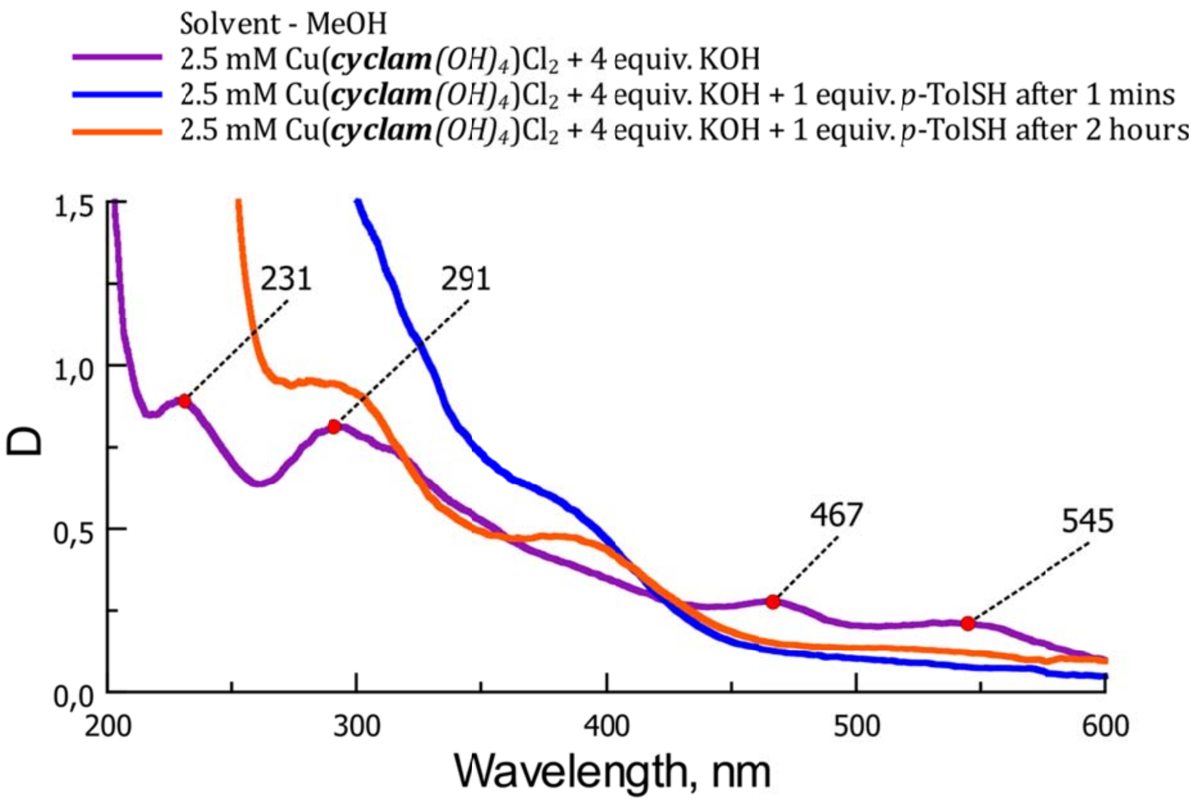

Reaction of deprotonated Cu(cyclam(OH)$_4$)Cl$_2$ complex with *N*'-phenylpropionohydrazide NPPH (UV-Vis monitoring)

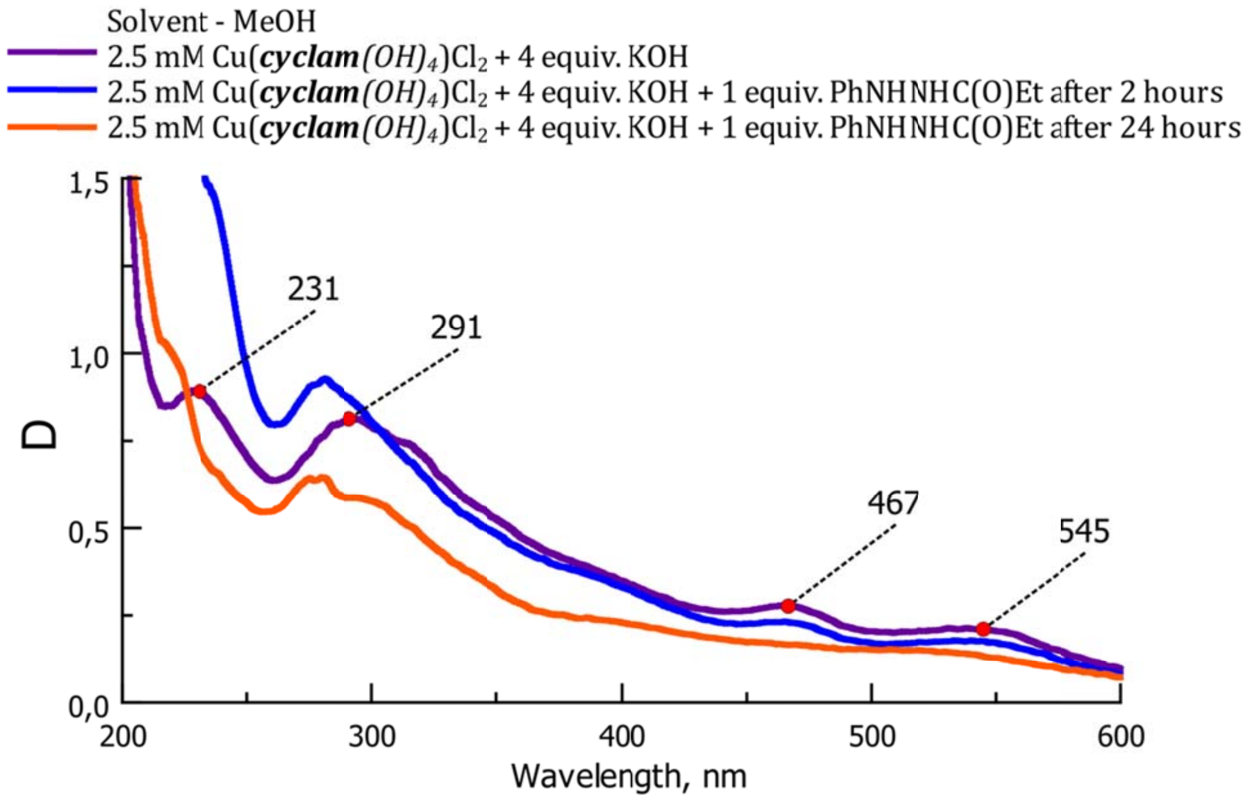

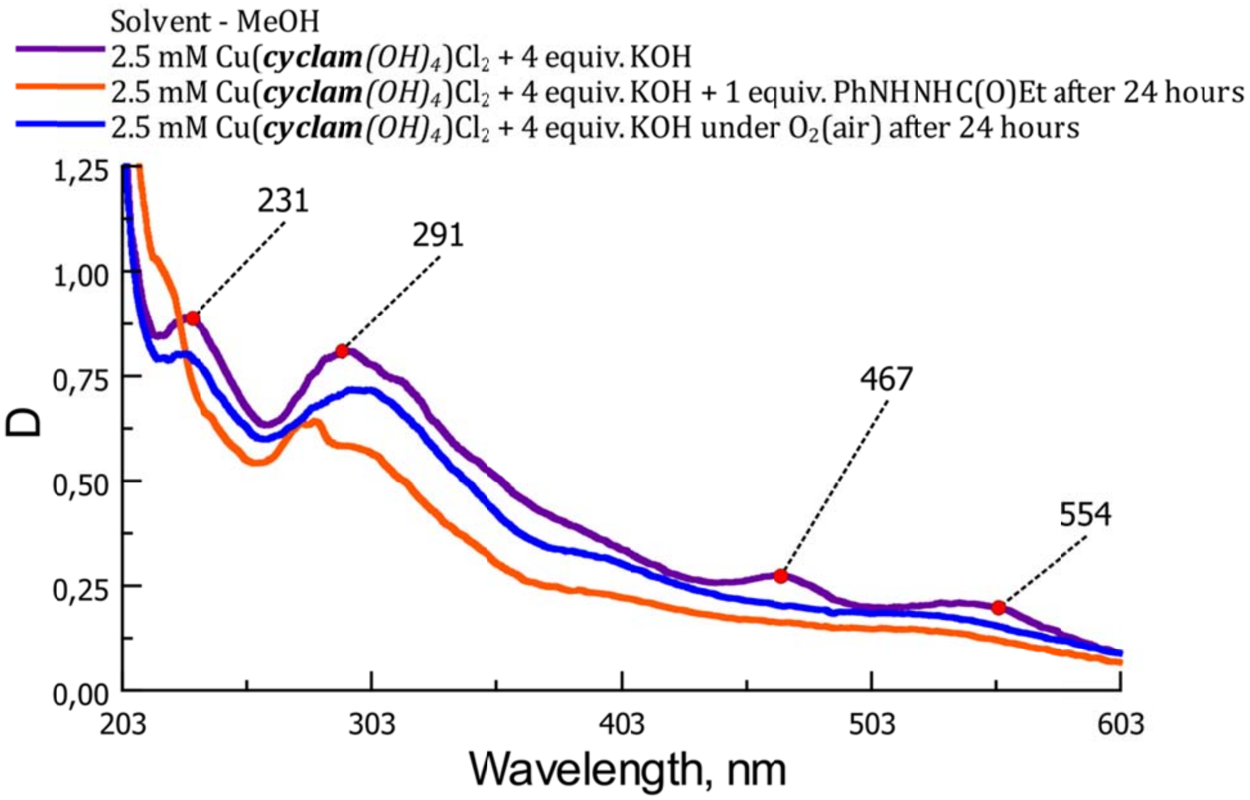

Catalytic activity of preoxidized Cu(cyclam(OH)$_4$)Cl$_2$ complex in aerobic oxidation of *N*'-phenylpropionohydrazide NPPH

Into a round-bottom flask connected with an air-filled balloon were placed Cu(cyclam(OH)$_4$)Cl$_2$ (11 mg, 0.03 mmol, 0.05 equiv.), $NaHCO_3$ (8 mg, 0.09 mmol, 0.15 equiv.) and MeOH (4 ml). The mixture was stirred under air for 24 hours and then *N*'-phenylpropionohydrazide NPPH (100 mg, 0.61 mmol, 1 equiv.) was added. The mixture was stirred under air for another 24 hours during which periodic sampling was performed (by removing small portions of the solution). The samples were analyzed by ^{1}H NMR with internal standard (1,1,2,2-tetrachloroethane) to determine the product yield. Results are given in Supplementary Table 4.

Supplementary Table 4. Monitoring of catalytic aerobic oxidation of *N*'-phenylpropionohydrazide NPPH with preoxidized Cu(cyclam(OH)$_4$)Cl$_2$ complex.

Time of reaction, h	NMR yield of *N*',*N*'-diphenylpropionohydrazide (DPPH), %
0	0
1	13
2	16
3	16
4	17
8	17
24	18

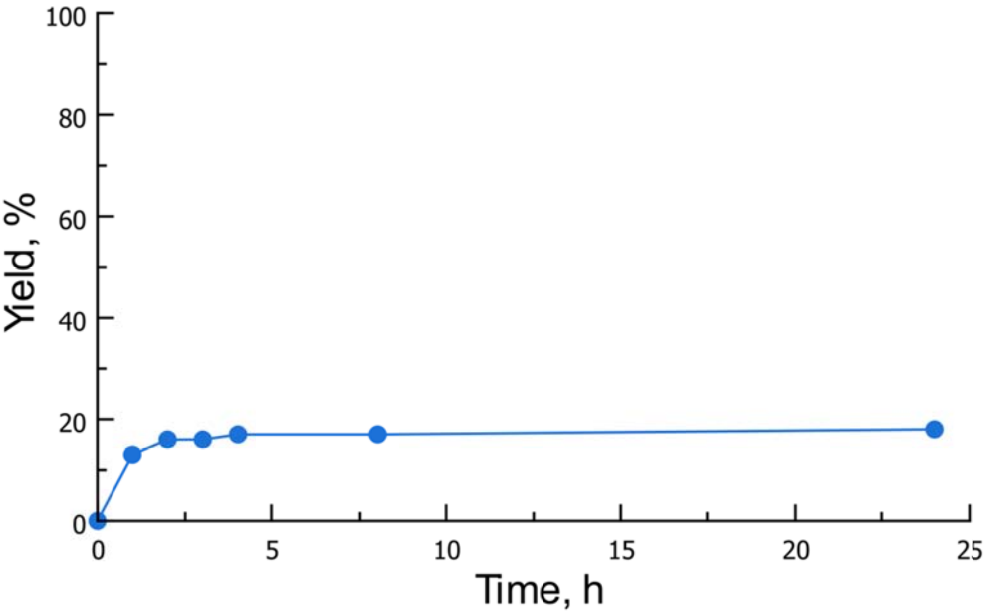

1.9 X-ray crystallographic data and refinement details

For $Mn(cyclam(OH)_4)Cl_2$ X-Ray diffraction data were collected at 100K on a four-circle Rigaku Synergy S diffractometer equipped with a HyPix600HE area-detector (kappa geometry, shutterless ω-scan technique), using graphite monochromatized Cu K_{α}-radiation. The intensity data were integrated and corrected for absorption and decay by the CrysAlisPro program.[16]

For $Cu(cyclam(OH)_4)Cl_2$, $cyclam(OH)_4$•2HBr intensities of reflections were measured with a Bruker Quest CCD.

For $tacn(OBz)_3$, $[Zn(tacn(OH)_3)_2](NO_3)_2$ intensities of reflections were measured with a Bruker AXS D8 QUEST, Photon III detector.

For $[Ni(tacn)_2]Cl_2$, $[Ni_2(\mu\text{-}Cl)_2(tacn)_2Cl_2]$, $[Ni_2(\mu\text{-}Cl)(\mu\text{-}O_2CPh)(tacn(OH)_3)_2Cl_2]$, $cyclam(OBz)_4$, $Mn(cyclam(OH)_4)Br_2\times1.33$ $cyclam(OH)_4$, $Zn(cyclam(OH)_4)Cl_2$, $cyclam(OH)_4$•HCl×MeOH intensities of reflections were measured with a Bruker APEXII DUO CCD diffractometer.

The structures were solved by direct methods using SHELXT[17] and refined on F^2 using SHELXL-2018[18] or Intrinsic Phasing and refined with the XL9 refinement package using Least-Squares minimization in the OLEX2 program.[19] All non-hydrogen atoms were refined with individual anisotropic displacement parameters. Hydrogen atoms of NH and OH groups are located from difference Fourier synthesis while the positions of other hydrogen atoms were calculated, and they all were refined in the isotropic approximation within the riding model. Graphical materials were prepared using Olex2, Mercury and Crystal explorer software. For pictures shown below hydrogen atoms except those of the NH and OH groups are omitted together with minor components of the disordered moieties, non-hydrogen atoms are shown as thermal ellipsoids at 50% probability level.

X-ray crystallographic data and refinement details are given in Supplementary Table 5.

In ORTEP pictures given below (Supplementary Figs. 1−14), hydrogen atoms except those of the NH and OH groups are omitted together with minor components of the disordered moieties, non-hydrogen atoms are shown as thermal ellipsoids at 50% probability level and only the labels of symmetry-independent heteroatoms are given.

Alerts A and B in the cif file of $[Ni(tacn)_2]Cl_2$ (CCDC 2259687) are because the best available crystal was small, of low reflective power and suffered from a severe disorder. Alert B in the cif file of $Ni(cyclam(O^-)(OH)_3)(ClO_4)$•$Ni(cyclam(OH)_4)(ClO_4)_2$•MeOH (CCDC 2271901) is because the compound suffers from a severe disorder.

CCDC numbers:

CCDC 2265473 tacn$(OBz)_3$

CCDC 2257251 cyclam$(OBz)_4$)

CCDC 2265480 cyclam$(OH)_4$•HCl•MeOH

CCDC 2257250 cyclam$(OH)_4$•2HBr

CCDC 2257253 $[Ni_2(\mu\text{-}Cl)(\mu\text{-}O_2CPh)(tacn(OH)_3)_2Cl_2]$

CCDC 2257254 $[Zn(tacn(OH)_3)_2](NO_3)_2$

CCDC 2257248 Cu(cyclam$(OH)_4)Cl_2$

CCDC 2257249 Mn(cyclam$(OH)_4)Br_2$•1.33 cyclam$(OH)_4$

CCDC 2257252 Zn(cyclam$(OH)_4)Cl_2$

CCDC 2265474 Mn(cyclam$(OH)_4)Cl_2$

CCDC 2265481 Ni(cyclam$(OH)_4)(NO_3)_2$

CCDC 2259686 $[Ni_2(\mu\text{-}Cl)_2(tacn)_2Cl_2]$

CCDC 2259687 $[Ni(tacn)_2]Cl_2$

CCDC 2271901 Ni(cyclam$(O^-)(OH)_3)(ClO_4)$•Ni(cyclam$(OH)_4)(ClO_4)_2$•MeOH

Supplementary Table 5. X-ray crystallographic data and refinement details

	**[Ni_2(μ-Cl)(μ-O_2CPh)(*tacn*$(OH)_3)_2Cl_2$]**	**cyclam$(OBz)_4$**	**Cu(cyclam$(OH)_4)Cl_2$**	**Mn(cyclam$(OH)_4)Br_2$ • 1.33 cyclam$(OH)_4$**
CCDC	2257253	2257251	2257248	2257249
Empirical formula	$C_{19}H_{35}Cl_3N_6Ni_2O_8$	$C_{38}H_{40}N_4O_8$	$C_{10}H_{24}Cl_2CuN_4O_4$	$C_{20}H_{48}Br_3Mn_{1.5}N_8O_8$
Formula weight	699.30	680.74	398.77	850.80
T, K	120	120	100	100
Crystal system	Monoclinic	Monoclinic	Orthorhombic	Orthorhombic
Space group	P_21/c	P_21/n	Pnnm	Pnnm
Z	4	2	2	1
a, Å	16.0394(14)	9.8615(18)	8.5435(3)	8.3953(2)
b, Å	10.8976(9)	17.354(3)	8.7986(4)	8.9647(2)
c, Å	16.1448(13)	10.091(2)	10.2326(3)	10.5499(3)
α, °	90	90	90	90
β, °	101.281(2)	99.634(5)	90	90
γ, °	90	90	90	90
V, Å^3	2767.4(4)	1702.6(6)	769.19(5)	794.00(3)
Dcalc (g/cm^3)	1.678	1.328	1.722	1.779
Linear absorption, μ (cm^{-1})	17.04	0.94	17.87	44.34
F(000)	1448	720	414	430
2θmax, °	56	50	54	54
Reflections measured	31291	10017	6601	8291
Independent reflections	6681	2993	887	919
Observed reflections [$I > 2\sigma(I)$]	4842	1751	810	907
Parameters	371	226	53	55
R_1	0.0425	0.0493	0.0257	0.0449
wR_2	0.1155	0.0979	0.0658	0.0983
GOF	1.018	0.962	1.118	1.197
$\Delta\rho$max/ $\Delta\rho$min (e Å^{-3})	0.971/-0.697	0.252/-0.220	0.454/-0.345	0.678/-0.722

Supplementary Table 5. X-ray crystallographic data and refinement details (continued)

	cyclam(OH)$_4$•2HBr	Zn(cyclam(OH)$_4$)Cl$_2$	[Zn(tacn(OH)$_3$)$_2$](NO$_3$)$_2$	tacn(OBz)$_3$
CCDC	2257250	2257252	2257254	2265473
Empirical formula	$C_{10}H_{26}Br_2N_4O_4$	$C_{10}H_{24}Cl_2N_4O_4Zn$	$C_{12}H_{30}N_8O_{12}Zn$	$C_{27}H_{27}N_3O_6$
Formula weight	426.17	400.60	543.81	489.51
T, K	100	100	100	100(2) K
Crystal system	Orthorhombic	Orthorhombic	Orthorhombic	Orthorhombic
Space group	Pnnm	Pnnm	Pccn	Pna$_2$1
Z	2	2	4	4
a, Å	8.3116(3)	8.5912(2)	17.8697(4)	9.9879(2)
b, Å	8.8500(3)	8.8714(2)	7.7553(2)	20.1943(5)
c, Å	10.7625(4)	10.1638(2)	15.4116(4)	12.4880(3)
α, °	90	90	90	90
β, °	90	90	90	90
γ, °	90	90	90	90
V, Å^3	791.66(5)	774.64(3)	2135.81(9)	2518.81(10)
Dcalc (g/cm^3)	1.788	1.717	1.691	1.291
Linear absorption, μ (cm^{-1})	51.38	19.5	12.29	0.92
F(000)	432	416	1136	1032
2θmax, °	50	54	56	56
Reflections measured	6408	8322	41322	51645
Independent reflections	745	895	2579	6068
Observed reflections [$I > 2\sigma(I)$]	716	854	2499	4520
Parameters	49	54	150	325
R_1	0.0456	0.0166	0.0185	0.0487
wR_2	0.1019	0.0440	0.0503	0.0840
GOF	1.342	1.061	1.073	1.077
$\Delta\rho$max/ $\Delta\rho$min (e Å^{-3})	0.999/-1.644	0.353/-0.214	0.392/-0.299	0.200/-0.227

Supplementary Table 5. X-ray crystallographic data and refinement details (continued)

	**Mn(cyclam(OH)$_4$)Cl$_2$**	**cyclam(OH)$_4$•HCl•MeOH**	**[Ni$_2$(μ-Cl)$_2$(tacn)$_2$Cl$_2$]**	**[Ni(tacn)$_2$]Cl$_2$**
CCDC	2265474	2265480	2259686	2259687
Empirical formula	$C_{10}H_{24}Cl_2MnN_4O_4$	$C_{11}H_{29}ClN_4O_5$	$C_{12}H_{30}Cl_4N_6Ni_2$	$C_{12}H_{30}Cl_2N_6Ni$
Formula weight	390.17	332.83	517.64	388.03
T, K	99.98(10)	100.00	120	120
Crystal system	Orthorhombic	Monoclinic	Monoclinic	Trigonal
Space group	Pnnm	P_21/n	P_21/c	P-3
Z	2	4	2	8
a, Å	8.6161(3)	8.7927(4)	7.4881(5)	16.597(6)
b, Å	8.9070(2)	16.2993(6)	7.3807(5)	16.597(6)
c, Å	10.2737(3)	12.2382(5)	17.3661(13)	17.424(10)
α, °	90	90	90	90
β, °	90	108.492(2)	97.451(2)	90
γ, °	90	90	90	120
V, Å^3	788.44(4)	1663.36(12)	951.68(12)	4157(4)
*D*calc (g/cm^3)	1.643	1.329	1.806	1.240
Linear absorption, *μ* (cm^{-1})	101.22	2.56	25.48	11.93
F(000)	406	720	536	1648
2*θ*max, °	154.644	61	50	50
Reflections measured	4653	37007	9105	16346
Independent reflections	884	5080	2526	5472
Observed reflections [$I > 2\sigma(I)$]		3615	2062	1257
Parameters	57	216	109	291
R_1	0.0506	0.0429	0.0337	0.0897
wR_2	0.1394	0.0872	0.0706	0.4188
GOF	1.057	1.038	1.020	0.993
Δ*ρ*max/ Δ*ρ*min (e Å^{-3})	1.028/-0.858	0.386/-0.309	0.478/-0.482	1.685/-1.027

Supplementary Table 5. X-ray crystallographic data and refinement details (continued)

	**Ni(cyclam(OH)$_4$)(NO$_3$)$_2$**	**Ni(cyclam(O$^-$)(OH)$_3$)(ClO$_4$)•** **Ni(cyclam(OH)$_4$)(ClO$_4$)$_2$•**MeOH
CCDC	2265481	2271901
Empirical formula	$C_{10}H_{24}N_6NiO_{10}$	$C_{21}H_{51}Cl_3N_8Ni_2O_{21}$
Formula weight	447.06	975.46
T, K	100.00	100
Crystal system	Monoclinic	Triclinic
Space group	P 1 n 1	P1¯
Z	2	4
a, Å	8.9390(2)	9.3800(5)
b, Å	8.4427(2)	13.2148(8)
c, Å	11.2600(2	30.3250(17)
α, °	90	77.443(3)
β, °	99.2930(10)	89.421(3)
γ, °	90	88.821(3)
V, Å^3	838.63(3) Å	3668.2(4)
Dcalc (g/cm$_3$)	1.770	1.766
Linear absorption, μ (cm^{-1})	12.26	13.40
F(000)	468	2032
2θmax, °	58.32	56
Reflections measured	48550	43643
Independent reflections	4517	17596
Observed reflections [I > 2σ(I)]	4425	14148
Parameters	331	1110
R_1	0.0461	0.1258
wR_2	0.1221	0.3235
GOF	1.033	1.126
$\Delta\rho$max/ $\Delta\rho$min (e Å^{-3})	0.647 and -1.065	1.838/ -1.406

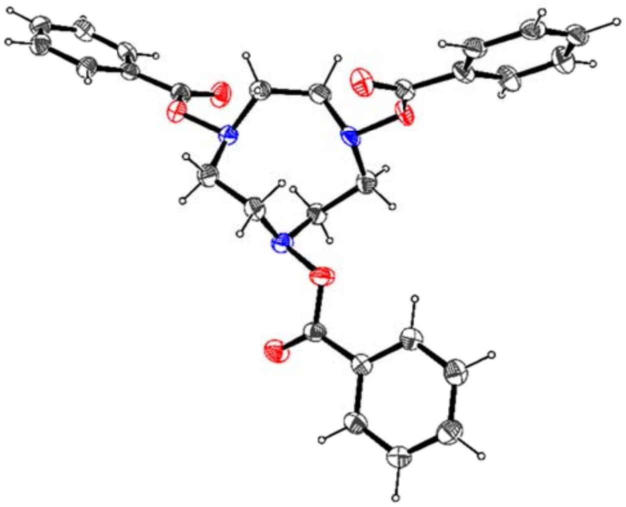

Supplementary Fig. 1. Ortep view of tacn(OBz)$_3$ with 50% probability level of displacement ellipsoids.

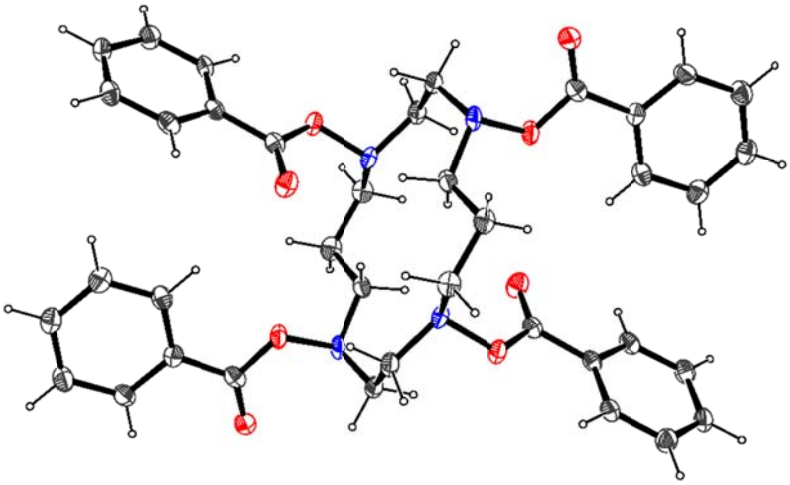

Supplementary Fig. 2. Ortep view of cyclam(OBz)$_4$ with 50% probability level of displacement ellipsoids.

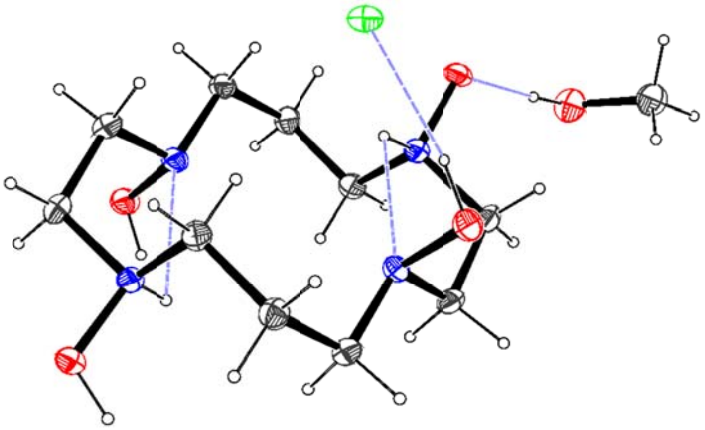

Supplementary Fig. 3. Ortep view of cyclam(OH)$_4$•HCl with 50% probability level of displacement ellipsoids.

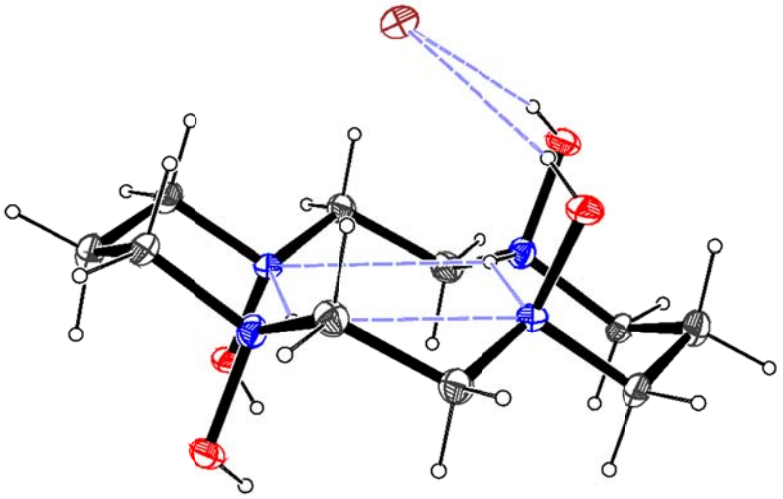

Supplementary Fig. 4. Ortep view of cyclam(OH)$_4$•2HBr with 50% probability level of displacement ellipsoids.

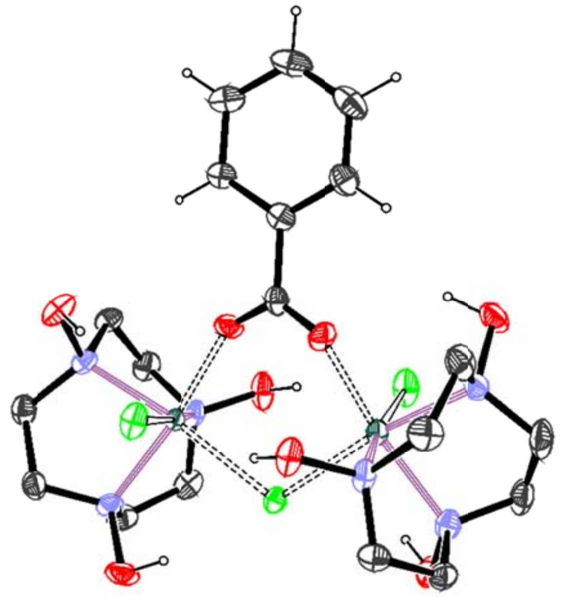

Supplementary Fig. 5. Ortep view of [Ni_2(μ-Cl)(μ-O_2CPh)(tacn$(OH)_3)_2Cl_2$] with 50% probability level of displacement ellipsoids.

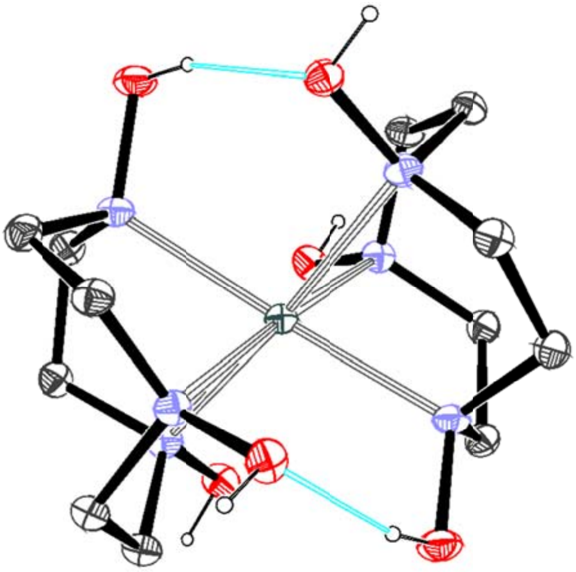

Supplementary Fig. 6. Ortep view of [Zn(tacn$(OH)_3)_2$]Cl_2 with 50% probability level of displacement ellipsoids.

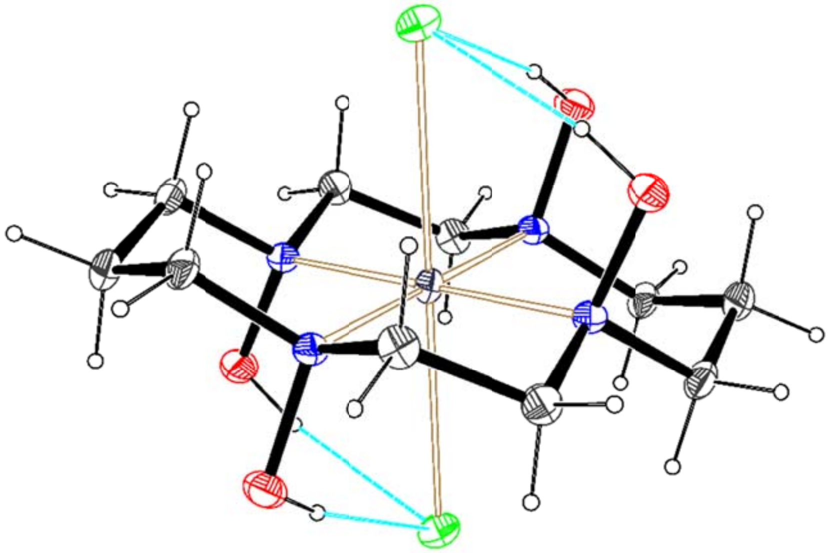

Supplementary Fig. 7. Ortep view of $Cu(cyclam(OH)_4)Cl_2$ with 50% probability level of displacement ellipsoids.

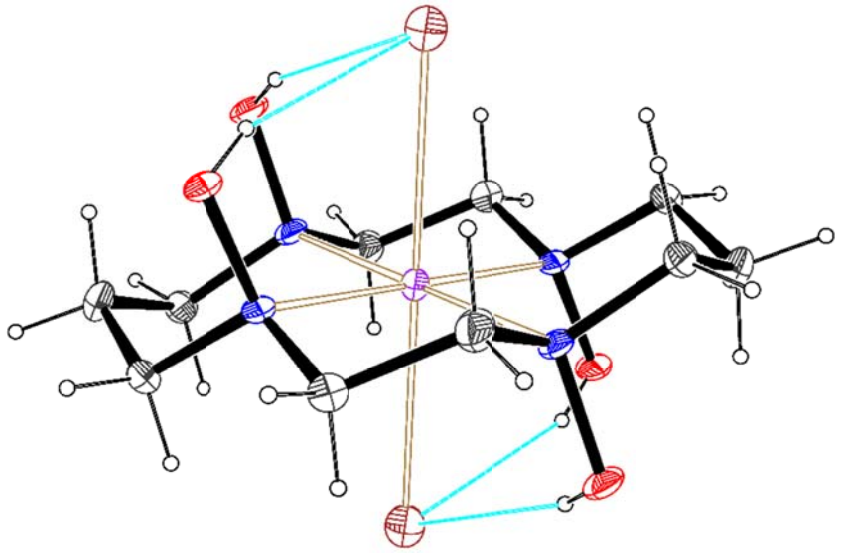

Supplementary Fig. 8. Ortep view of $Mn(cyclam(OH)_4)Br_2$ with 50% probability level of displacement ellipsoids.

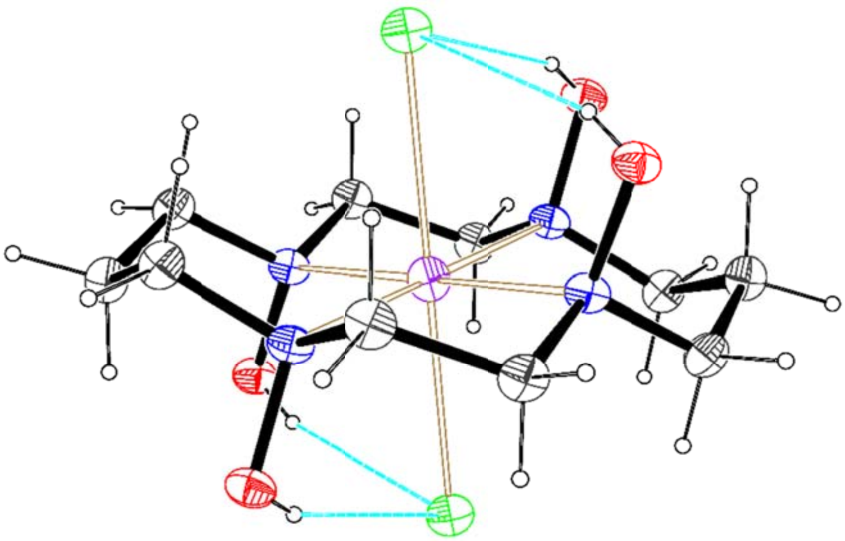

Supplementary Fig. 9. Ortep view of Mn(cyclam(OH)$_4$)Cl$_2$ with 50% probability level of displacement ellipsoids.

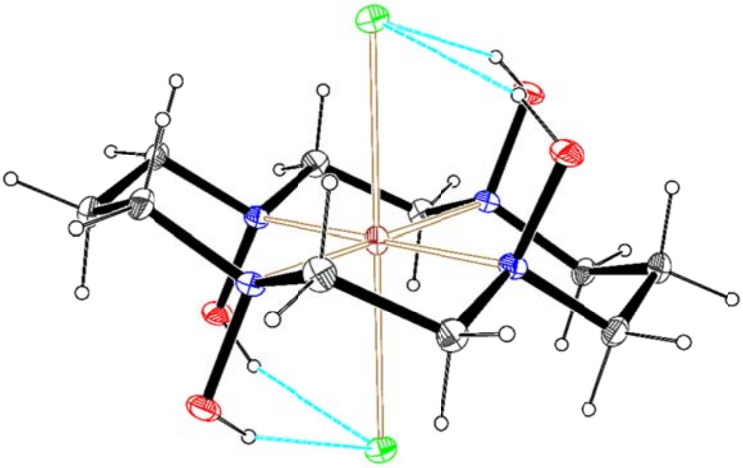

Supplementary Fig. 10. Ortep view of Zn(cyclam(OH)$_4$)Cl$_2$ with 50% probability level of displacement ellipsoids.

a

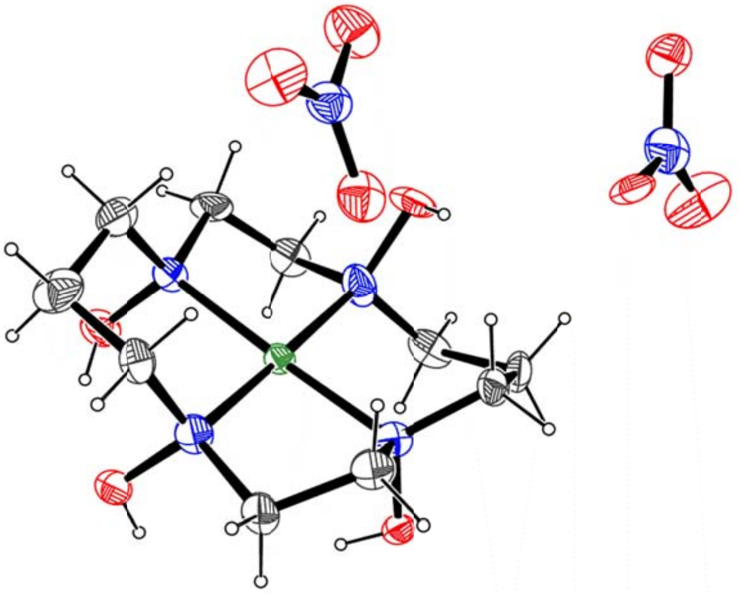

b

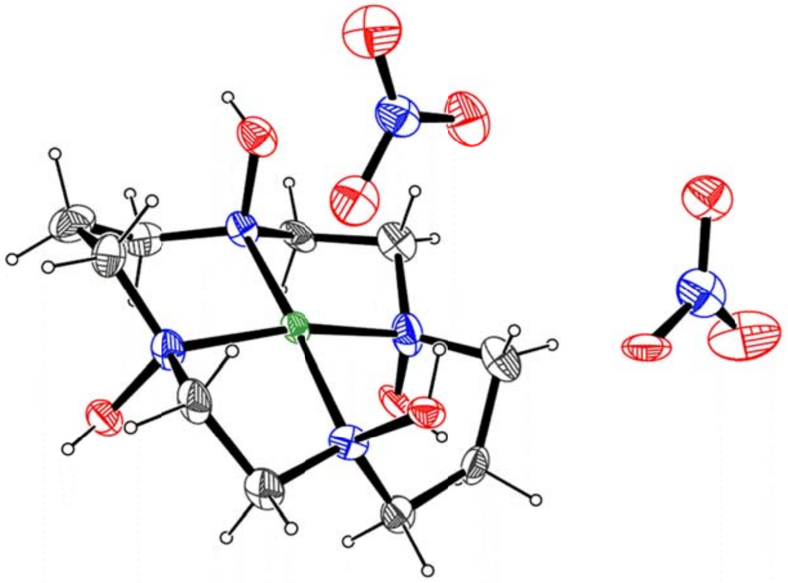

Supplementary Fig. 11. Crystal structure of Ni(cyclam(OH)$_4$(NO$_3$)$_2$. **a** Ortep view of Ni(cyclam(OH)$_4$(NO$_3$)$_2$ (part A) with 50% probability level of displacement ellipsoids. **b** Ortep view of Ni(cyclam(OH)$_4$(NO$_3$)$_2$ (part B) with 50% probability level of displacement ellipsoids.

a

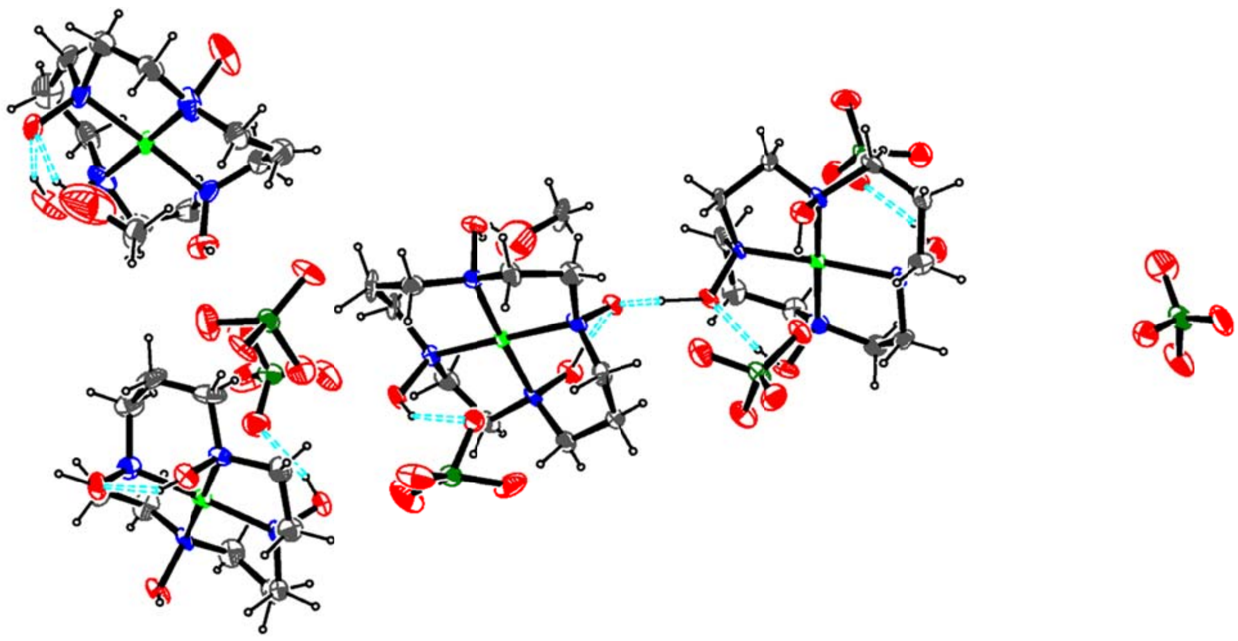

b

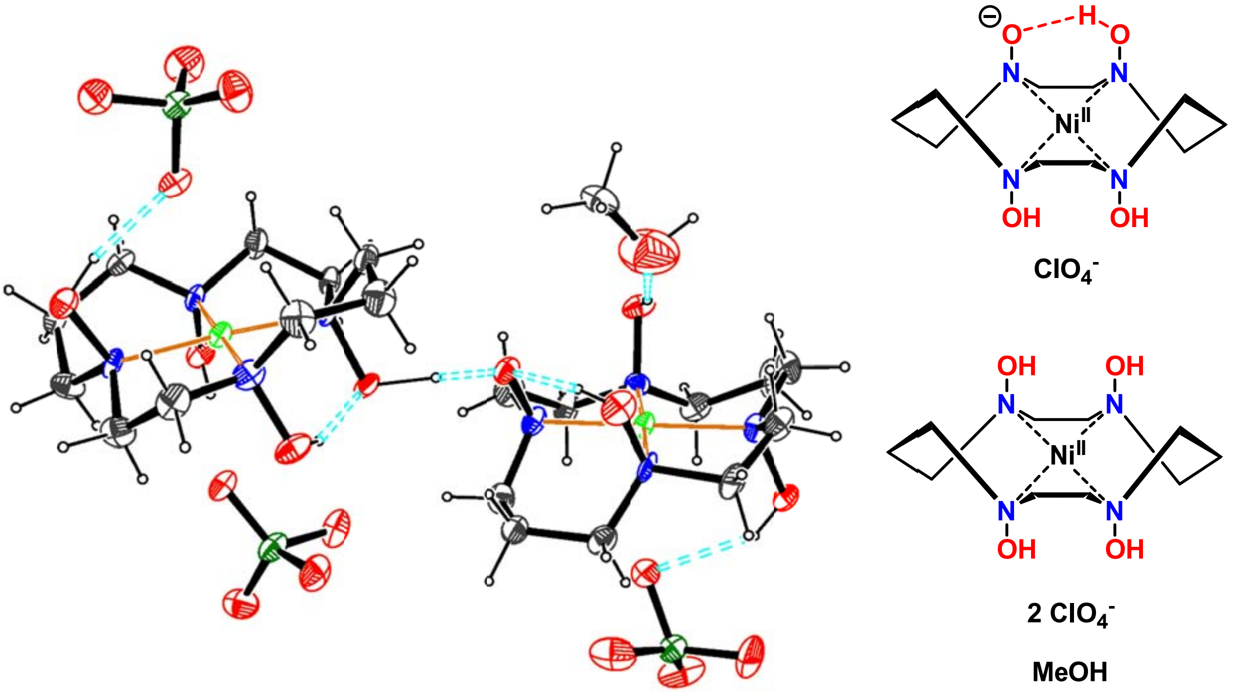

Supplementary Fig. 12. Crystal structure of Ni(cyclam(O$^-$)(OH)$_3$)(ClO$_4$)•Ni(cyclam(OH)$_4$)(ClO$_4$)$_2$•MeOH. **a** Ortep view of Ni(cyclam(O$^-$)(OH)$_3$)(ClO$_4$)•Ni(cyclam(OH)$_4$)(ClO$_4$)$_2$•MeOH with 50% probability level of displacement ellipsoids. **b** Close view on central motif.

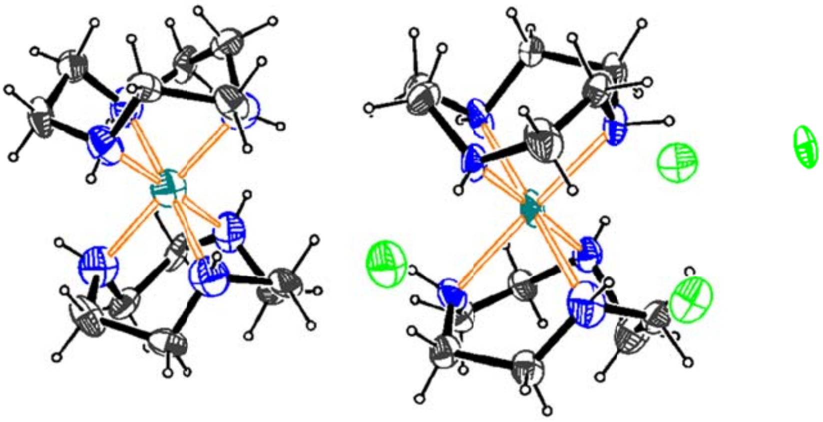

Supplementary Fig. 13. Ortep view of $[Ni(tacn)_2]Cl_2$ with 50% probability level of displacement ellipsoids.

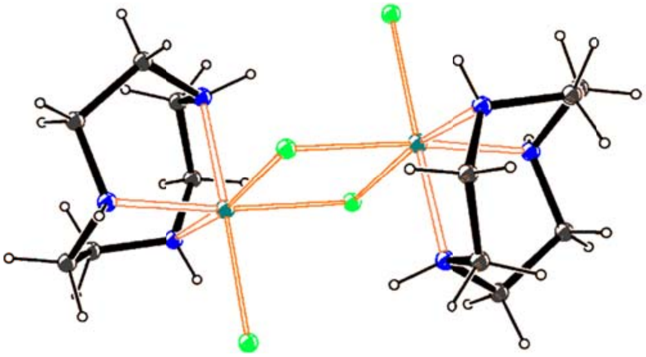

Supplementary Fig. 14. Ortep view of $[Ni_2(\mu\text{-}Cl)_2(tacn)_2Cl_2]$ with 50% probability level of displacement ellipsoids.

1.10 Copies of NMR, FT-IR and UV-Vis spectra

^{1}H NMR, $CDCl_3$, 298K

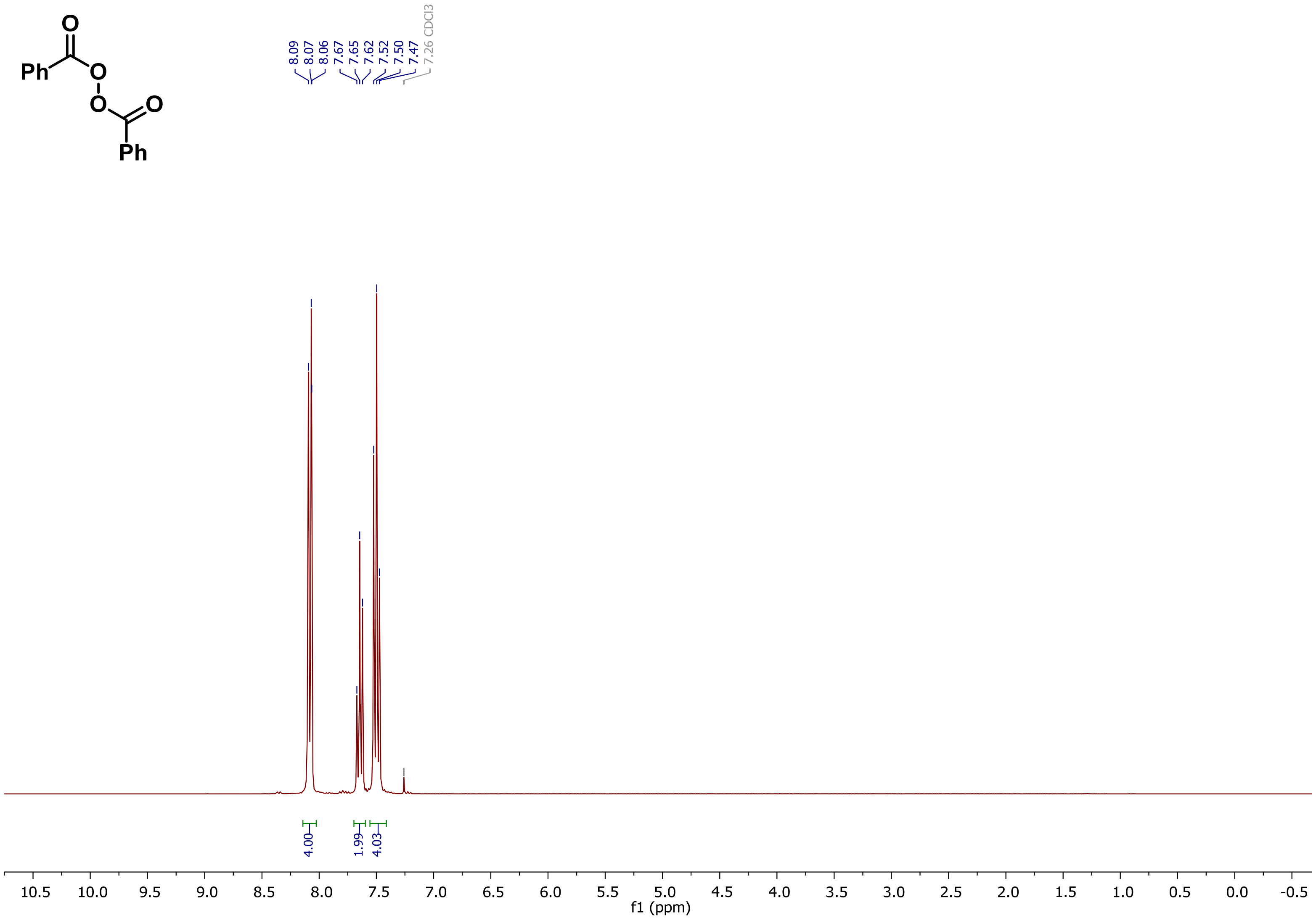

^{13}C NMR, $CDCl_3$, 298K

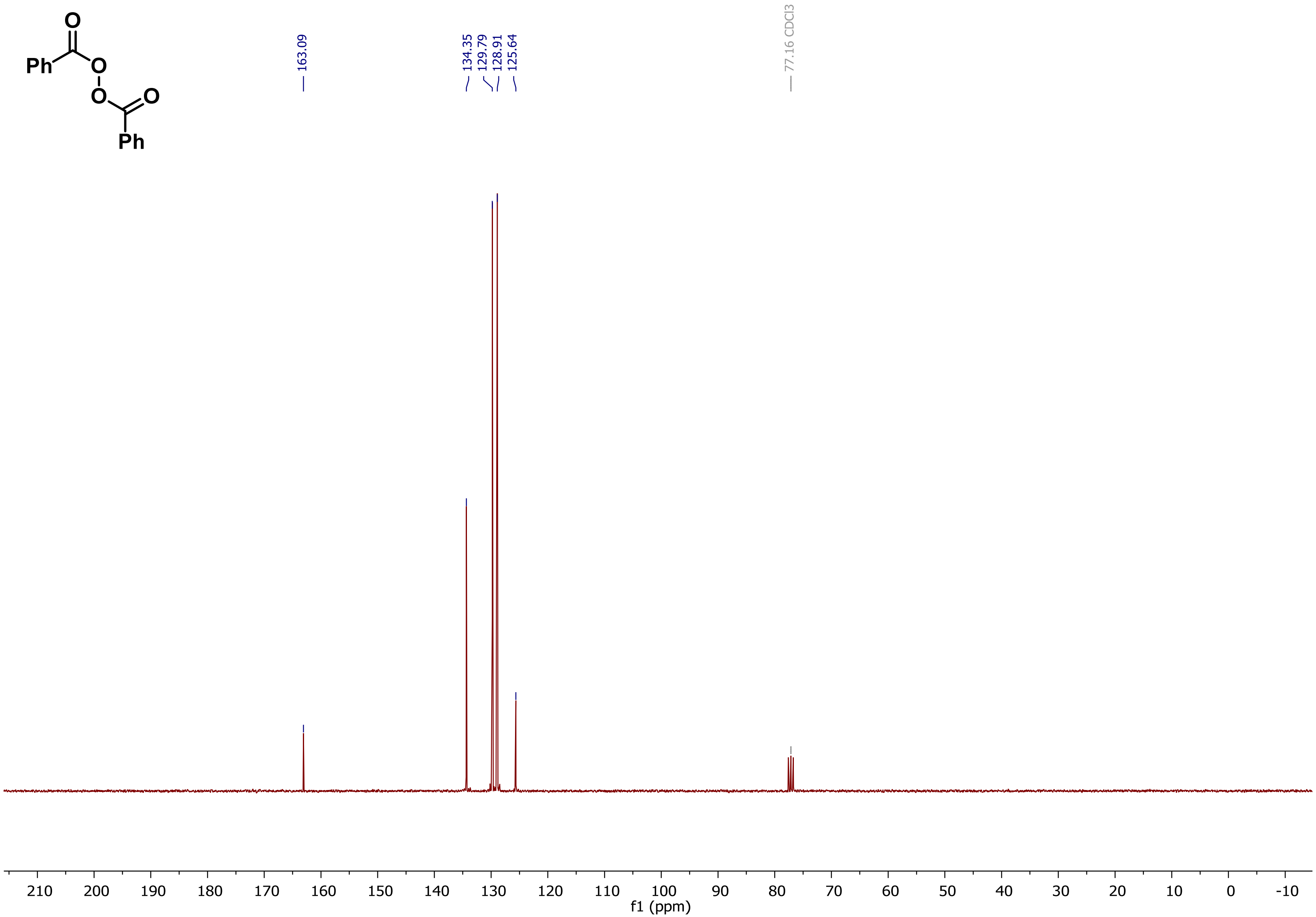

^{1}H NMR, $CDCl_3$, 298K

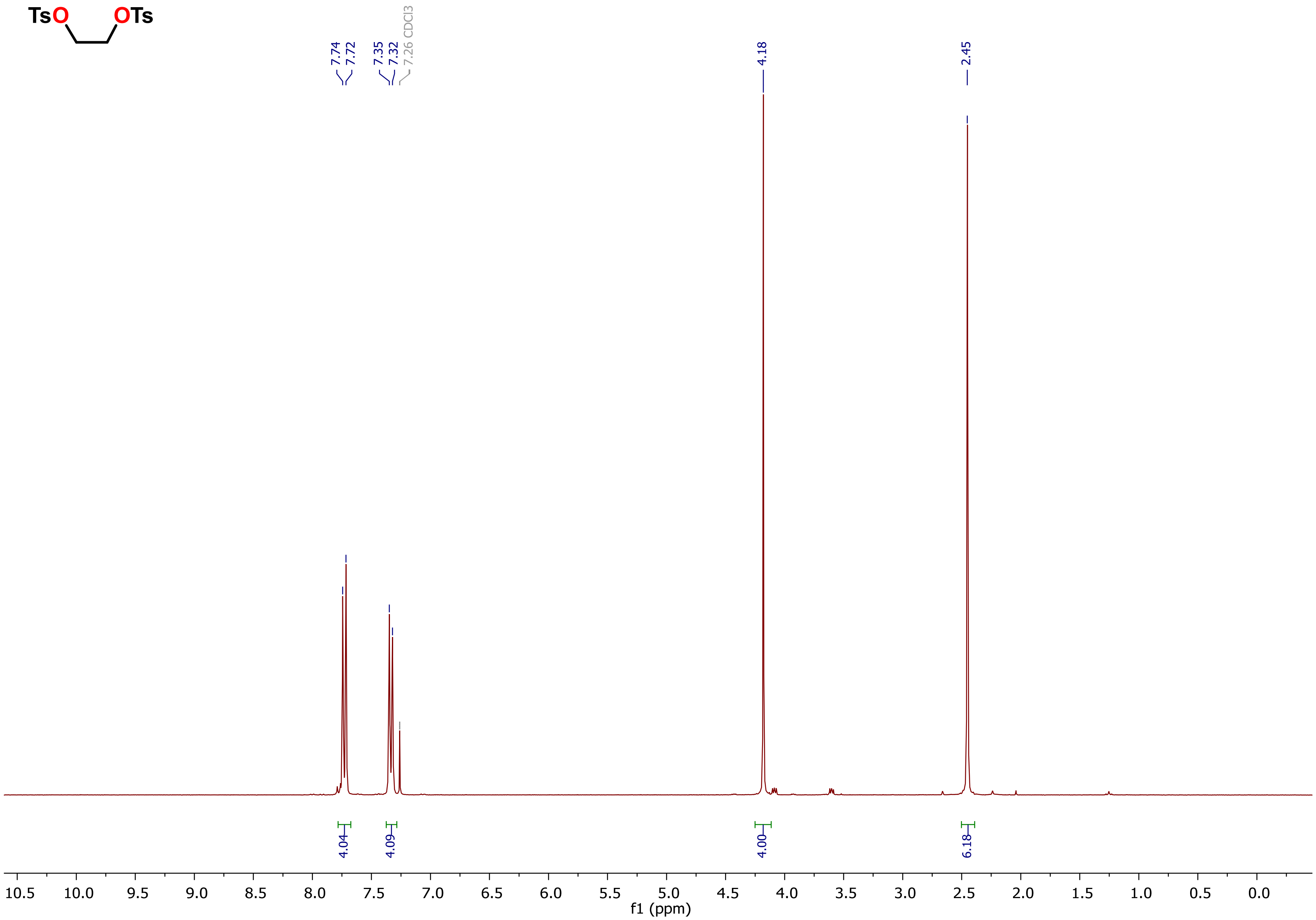

^{13}C NMR, $CDCl_3$, 298K

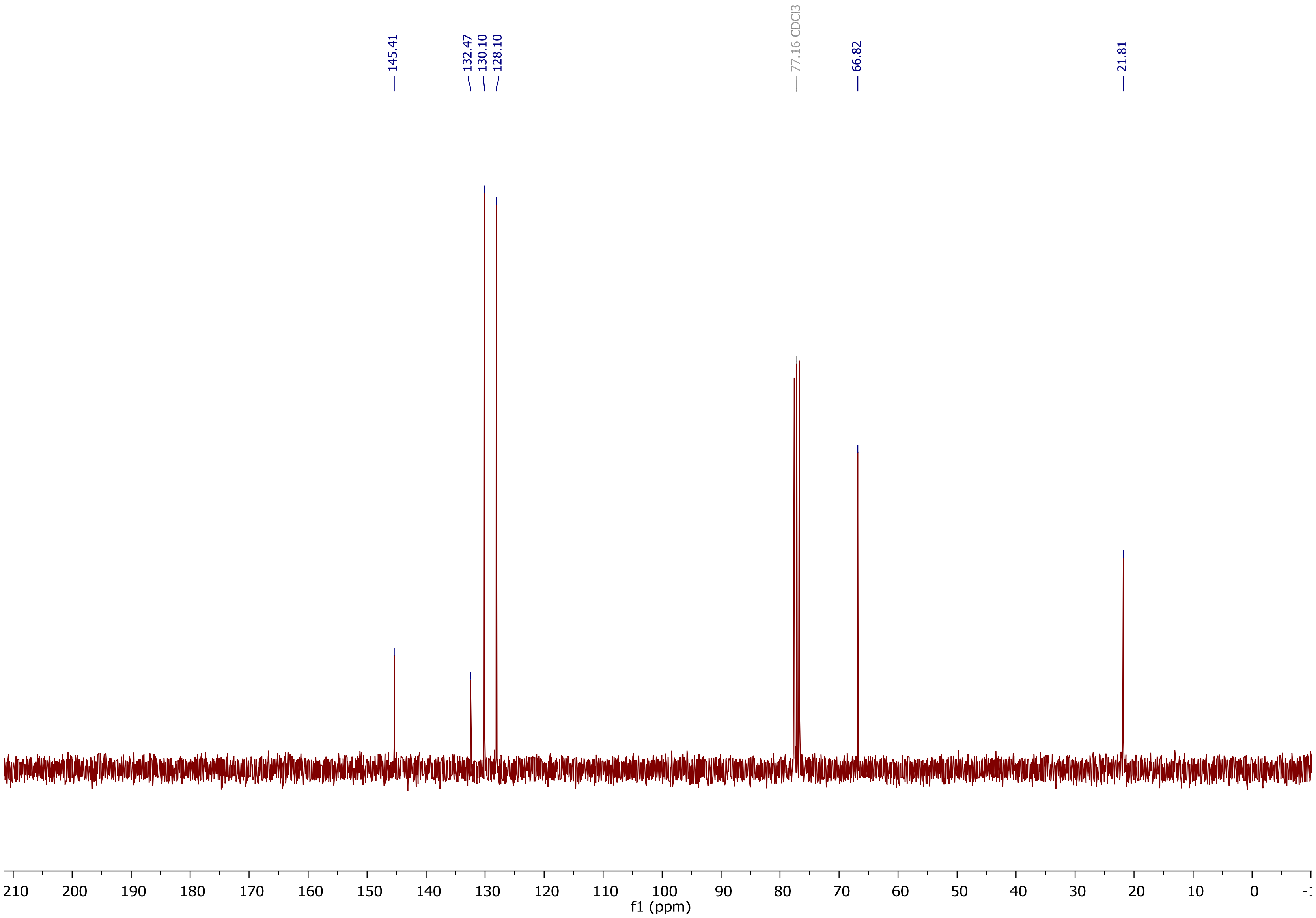

^1H NMR, $CDCl_3$, 298K

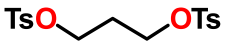

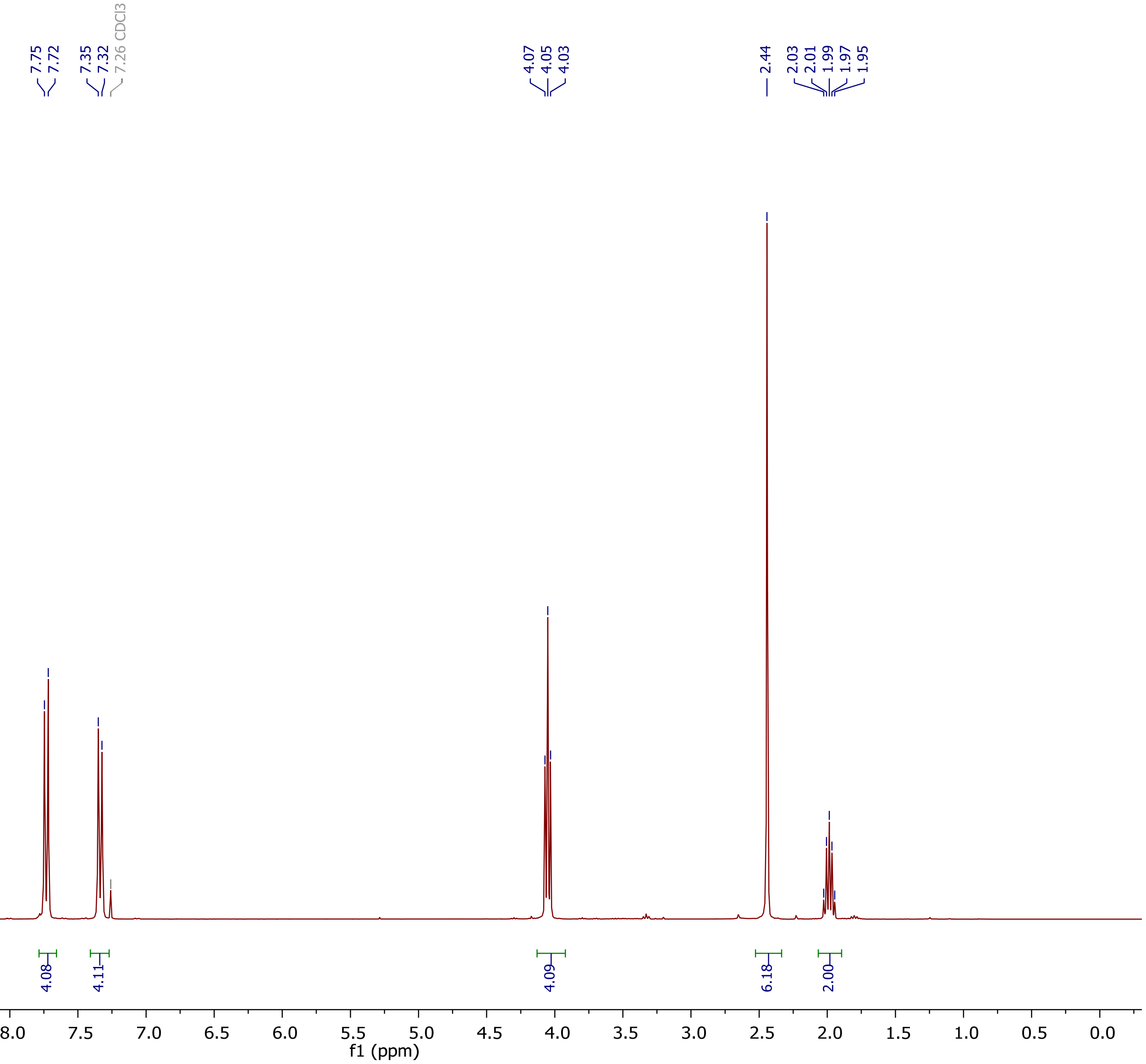

^{13}C NMR, $CDCl_3$, 298K

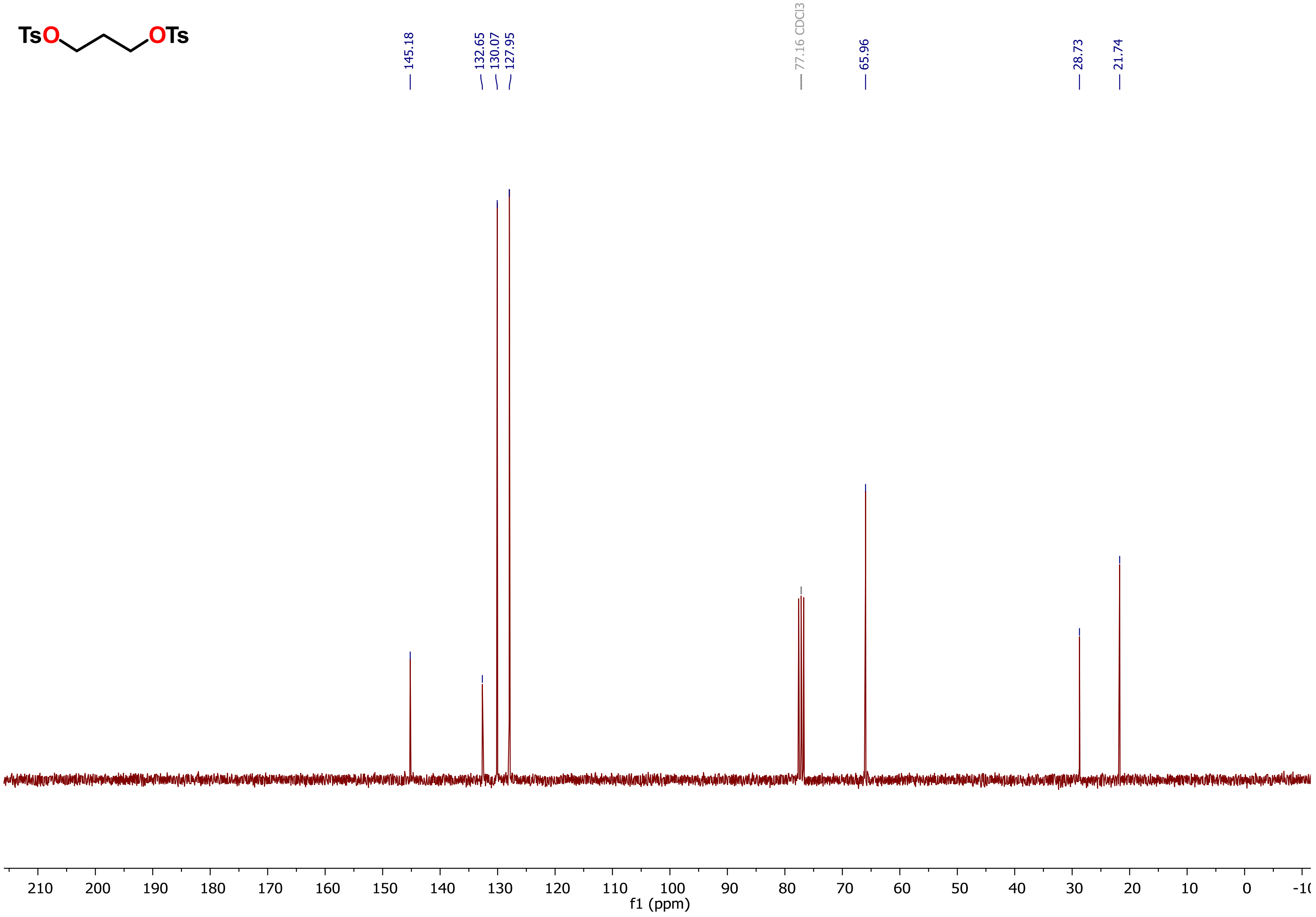

^{1}H NMR, DMSO-d_6, 298K

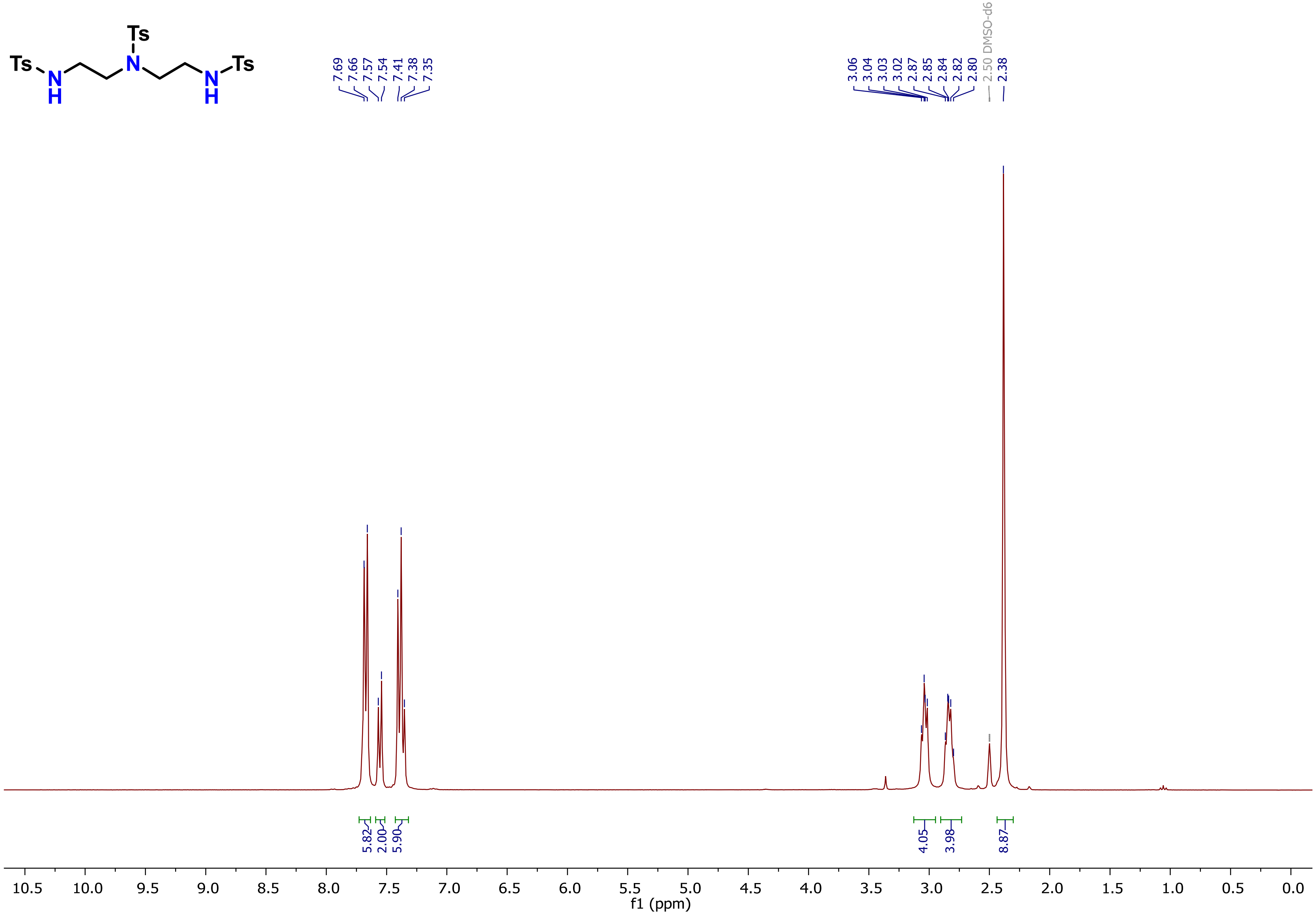

^{13}C NMR, DMSO-d_6, 298K

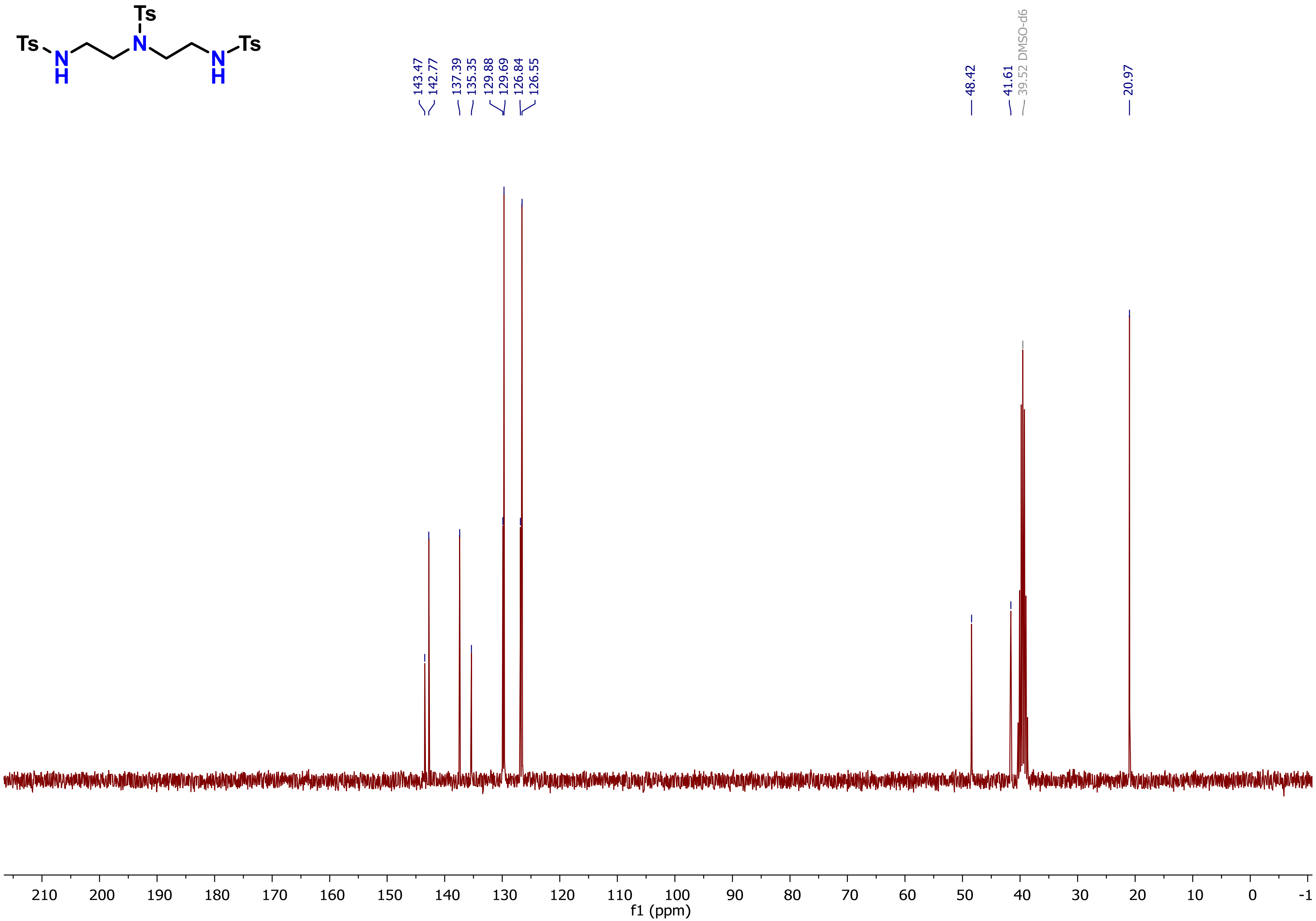

^{1}H-^{13}C HSQC, DMSO-d_6, 298K

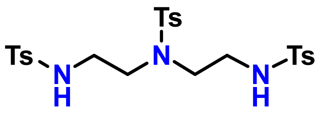

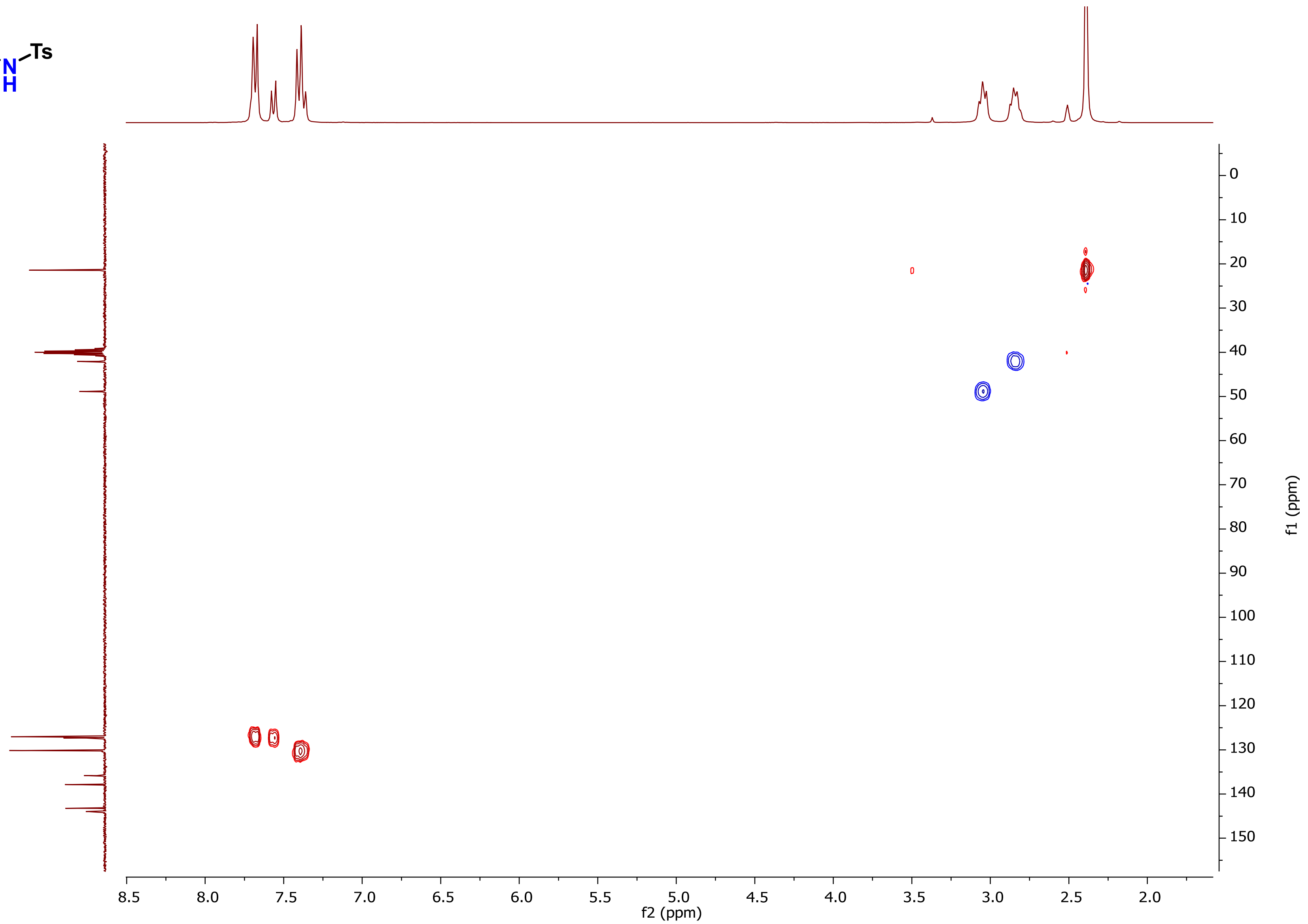

^{1}H NMR, DMSO-d_6, 298K

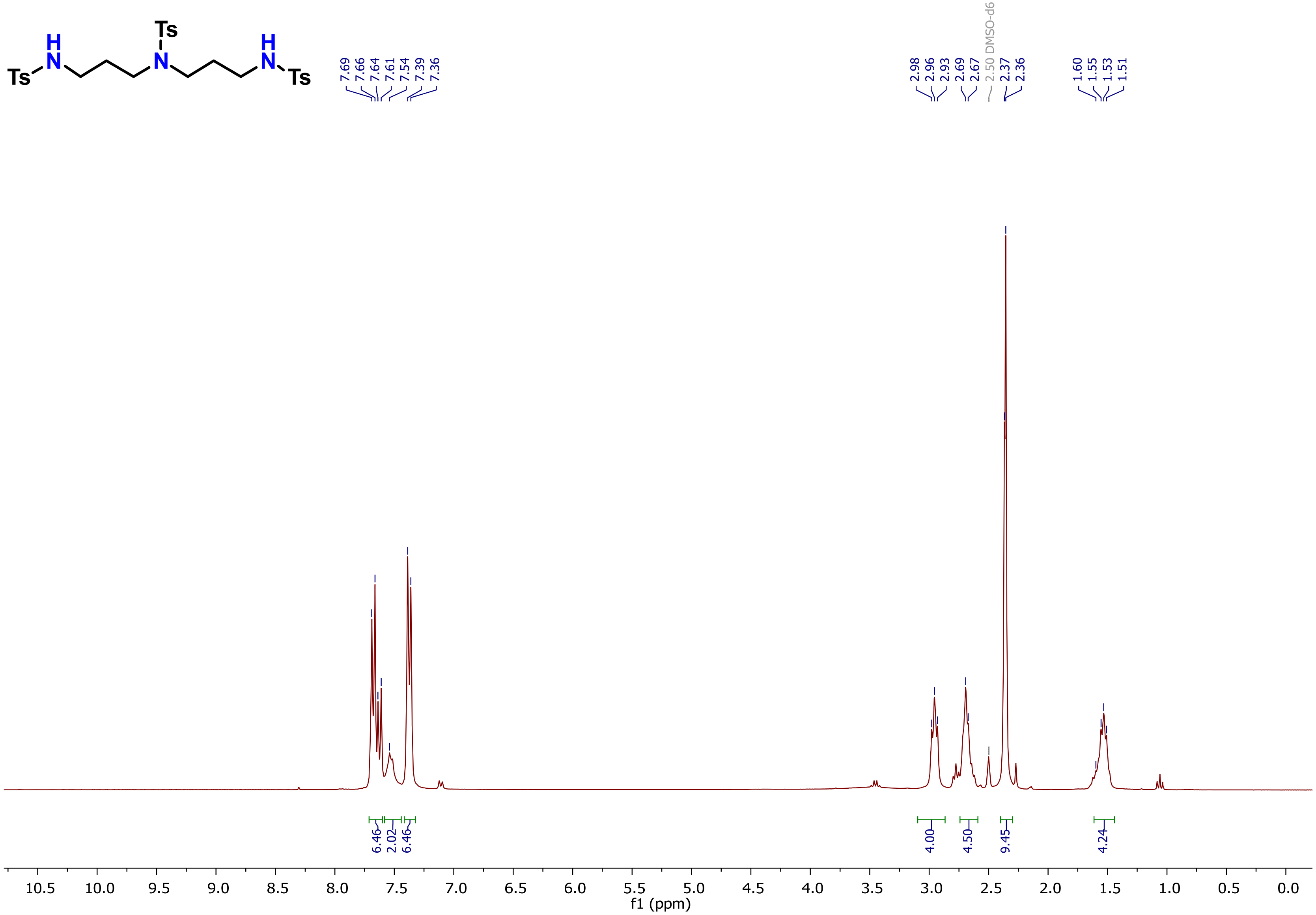

^{13}C NMR, DMSO-d_6, 298K

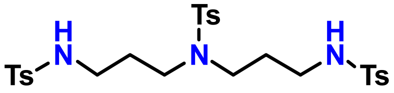

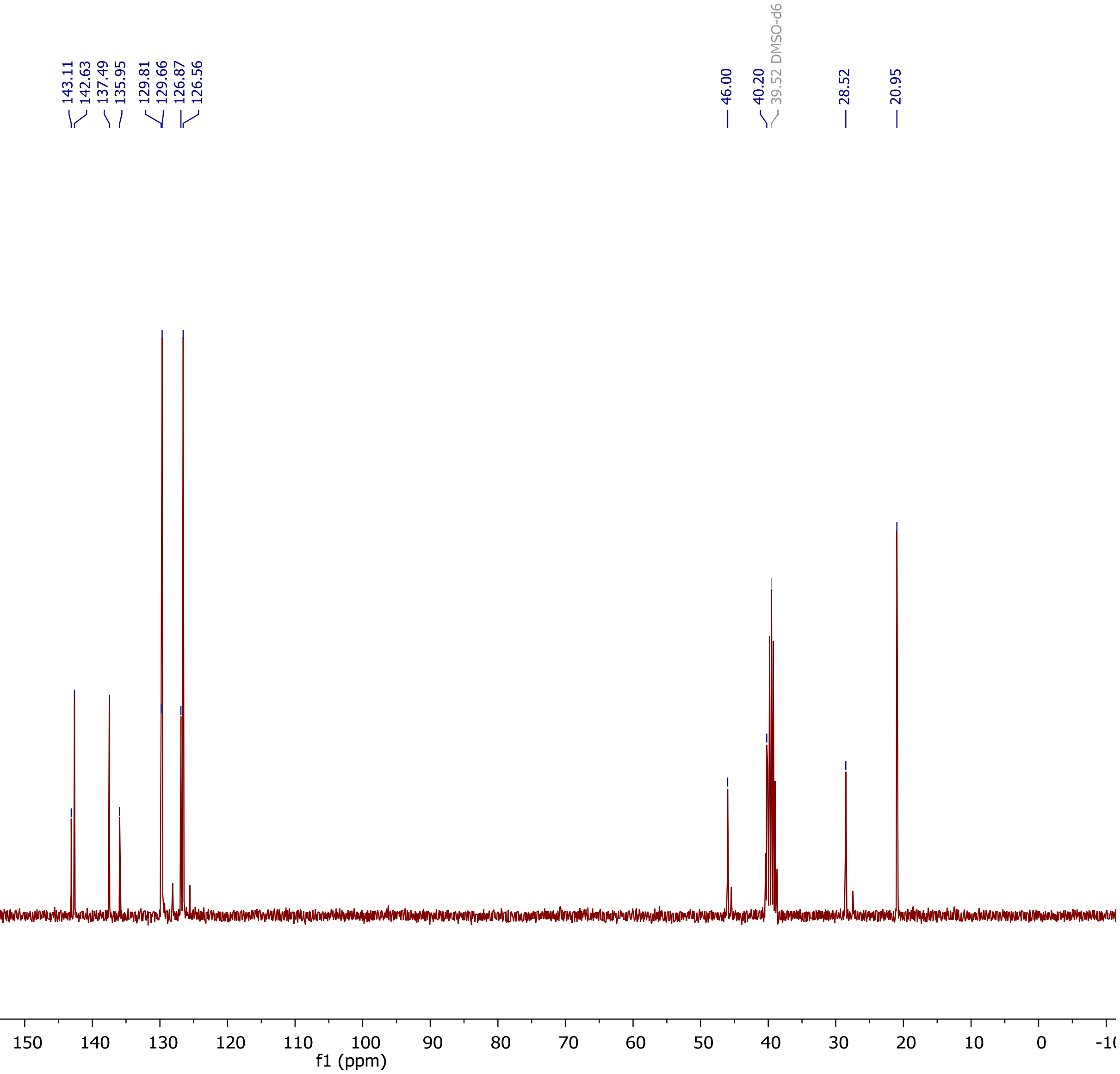

^{1}H-^{13}C HSQC, DMSO-d_6, 298K

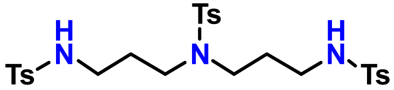

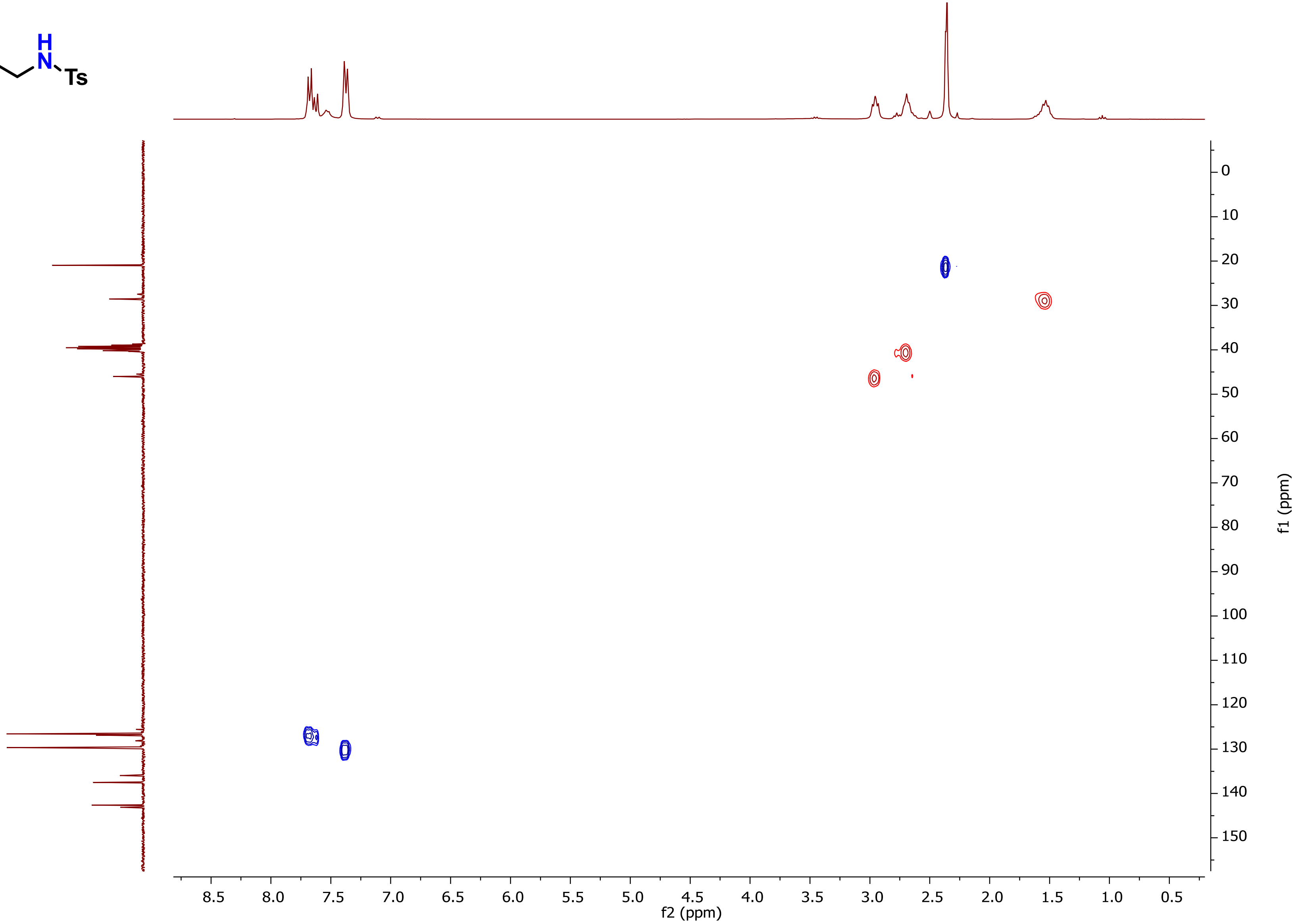

^1H NMR, $CDCl_3$, 298K

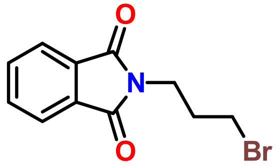

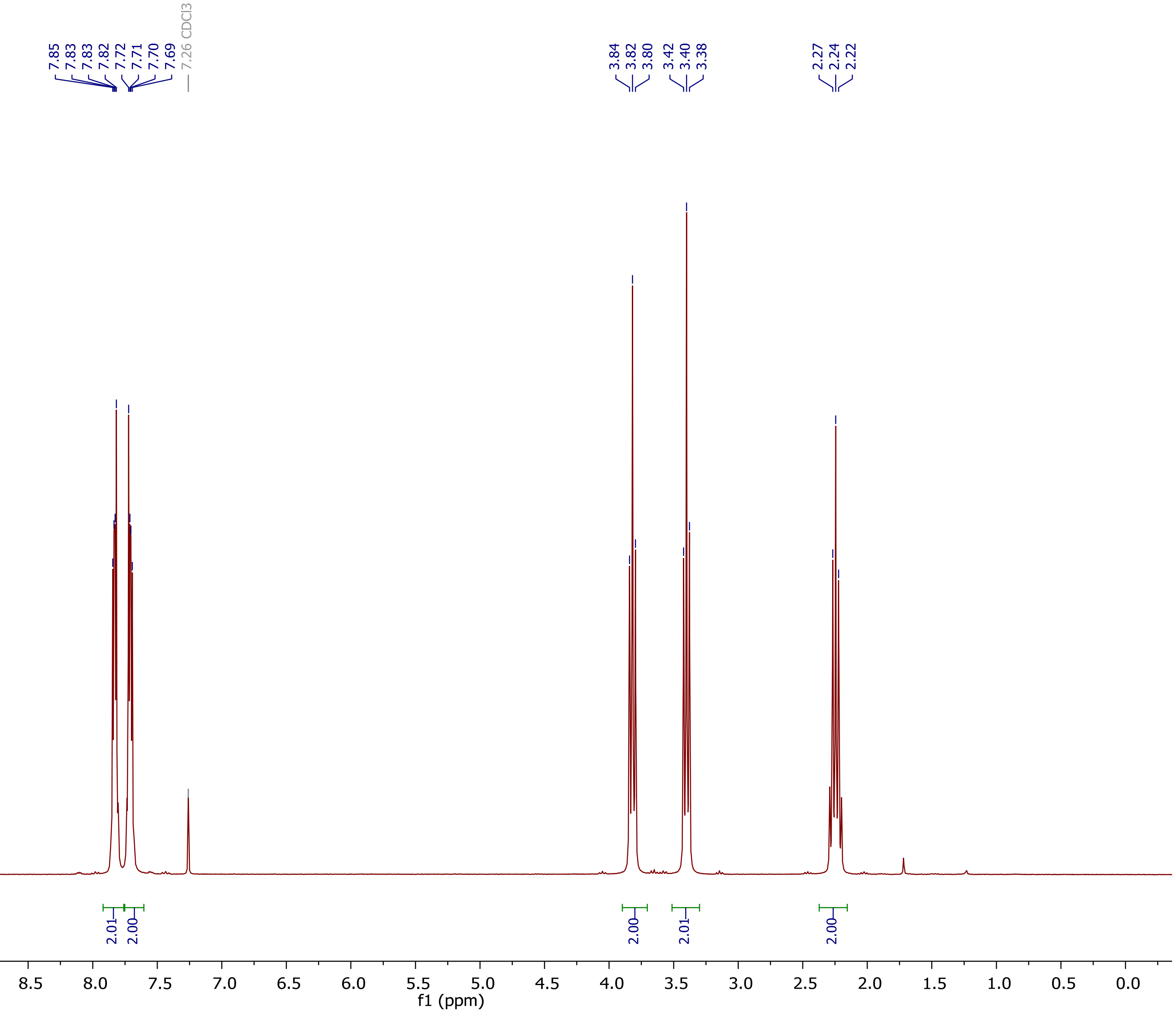

^{13}C NMR, $CDCl_3$, 298K

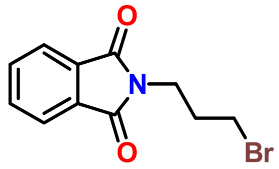

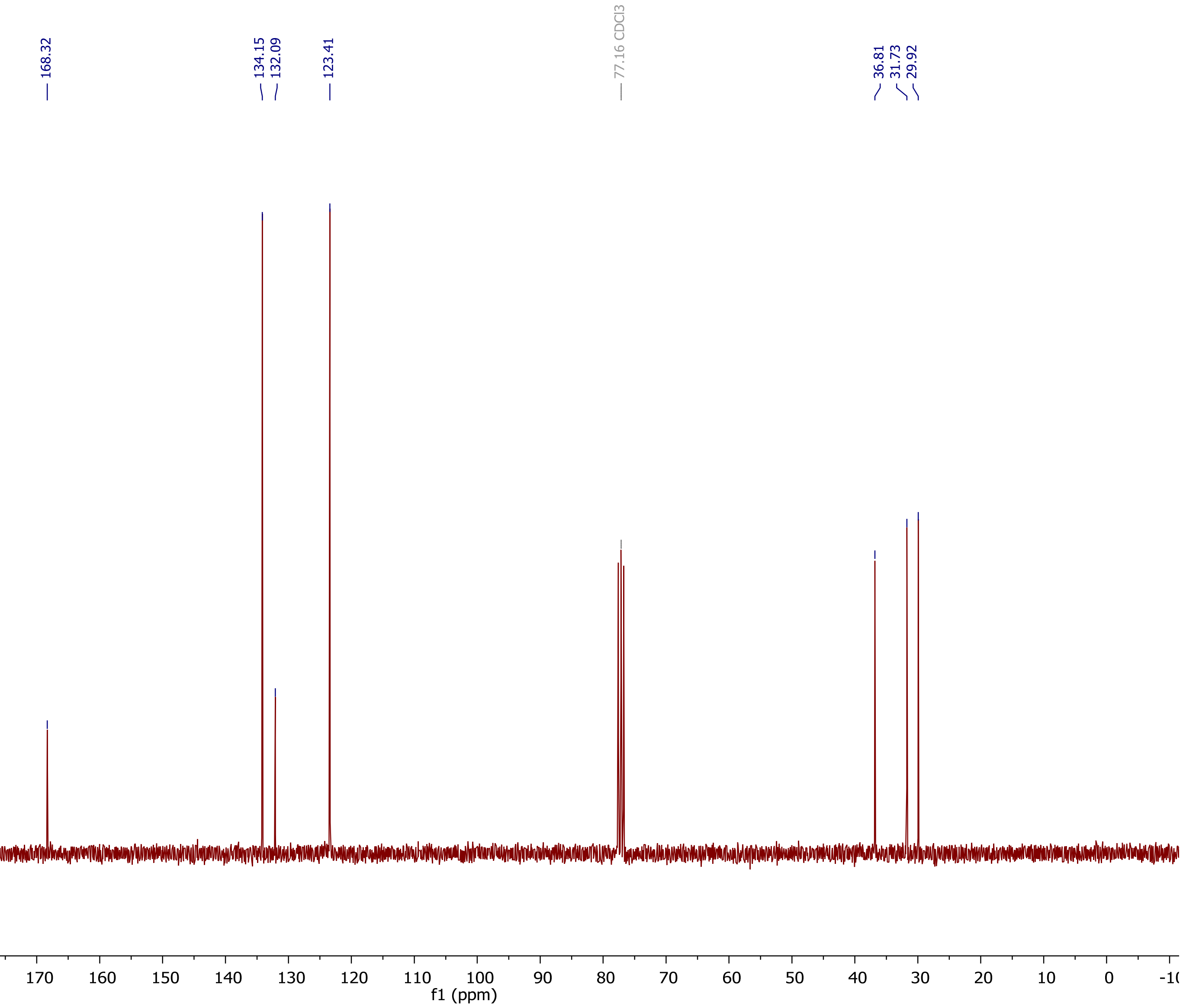

^{1}H-^{13}C HSQC, $CDCl_3$, 298K

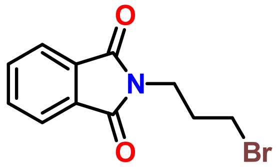

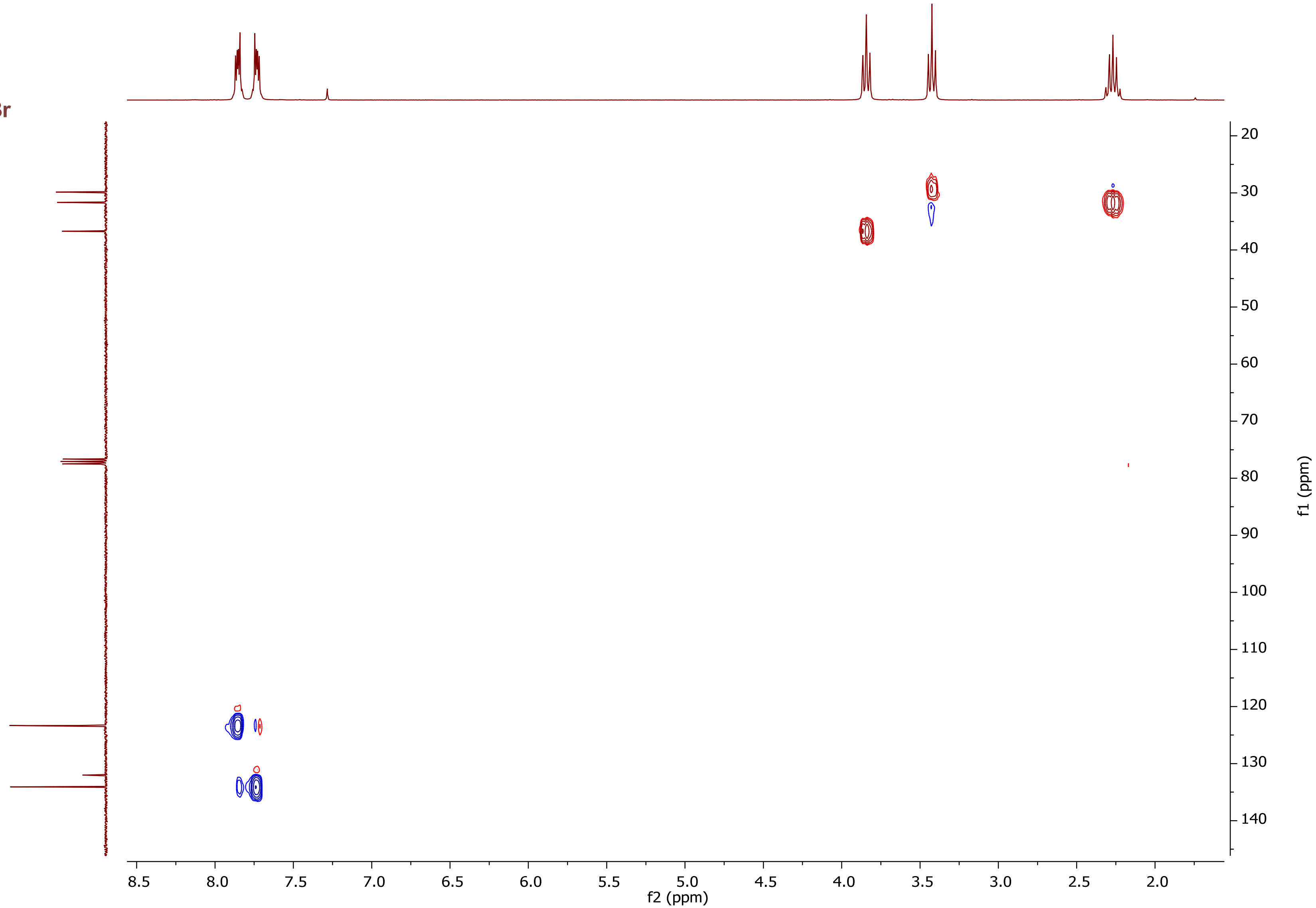

^1H NMR, $CDCl_3$, 298K

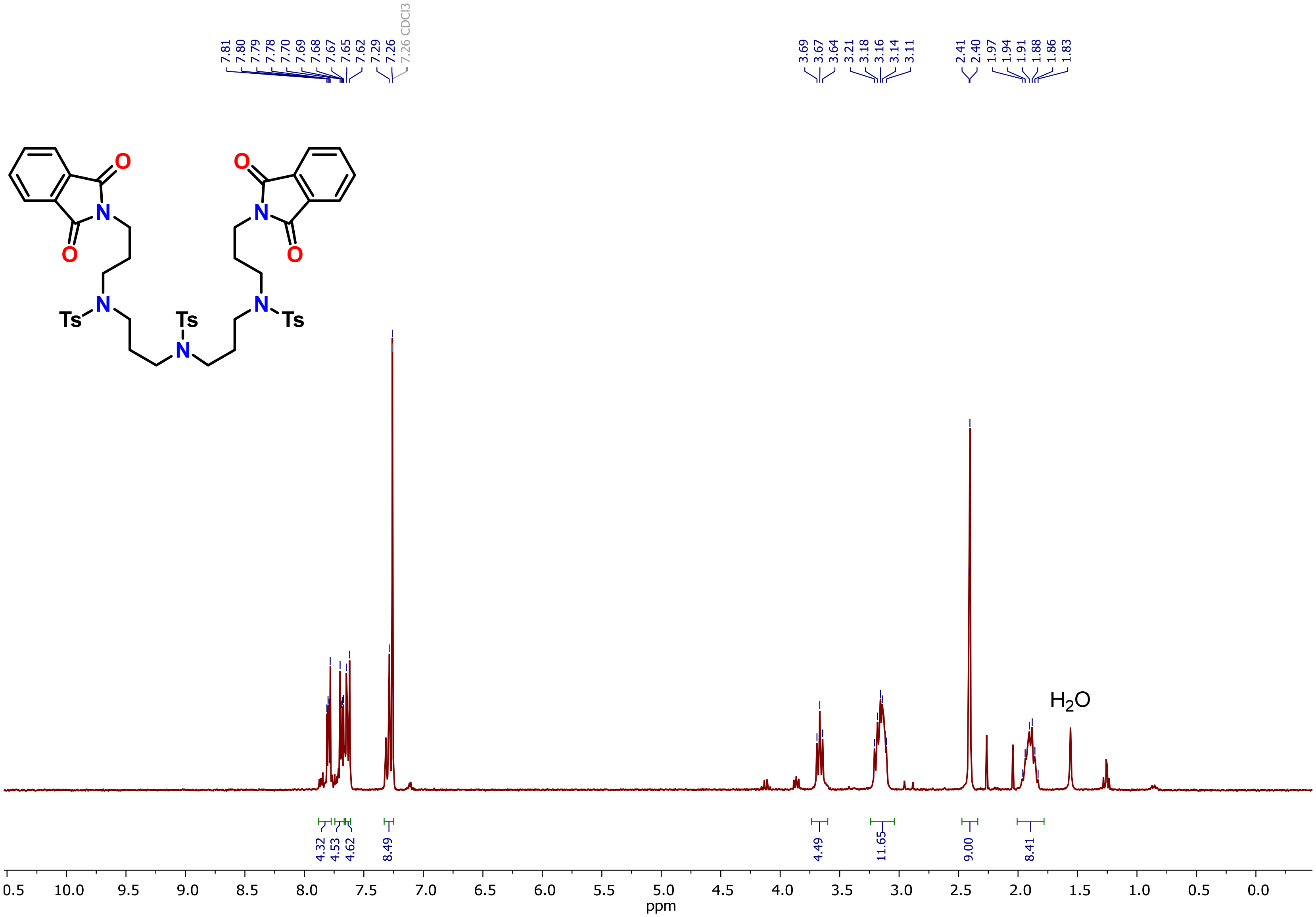

^{13}C NMR, $CDCl_3$, 298K

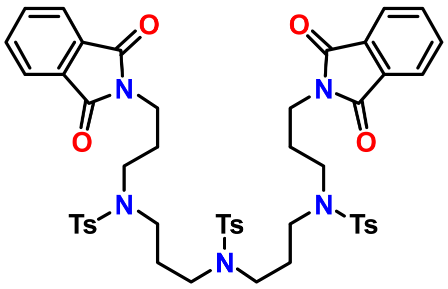

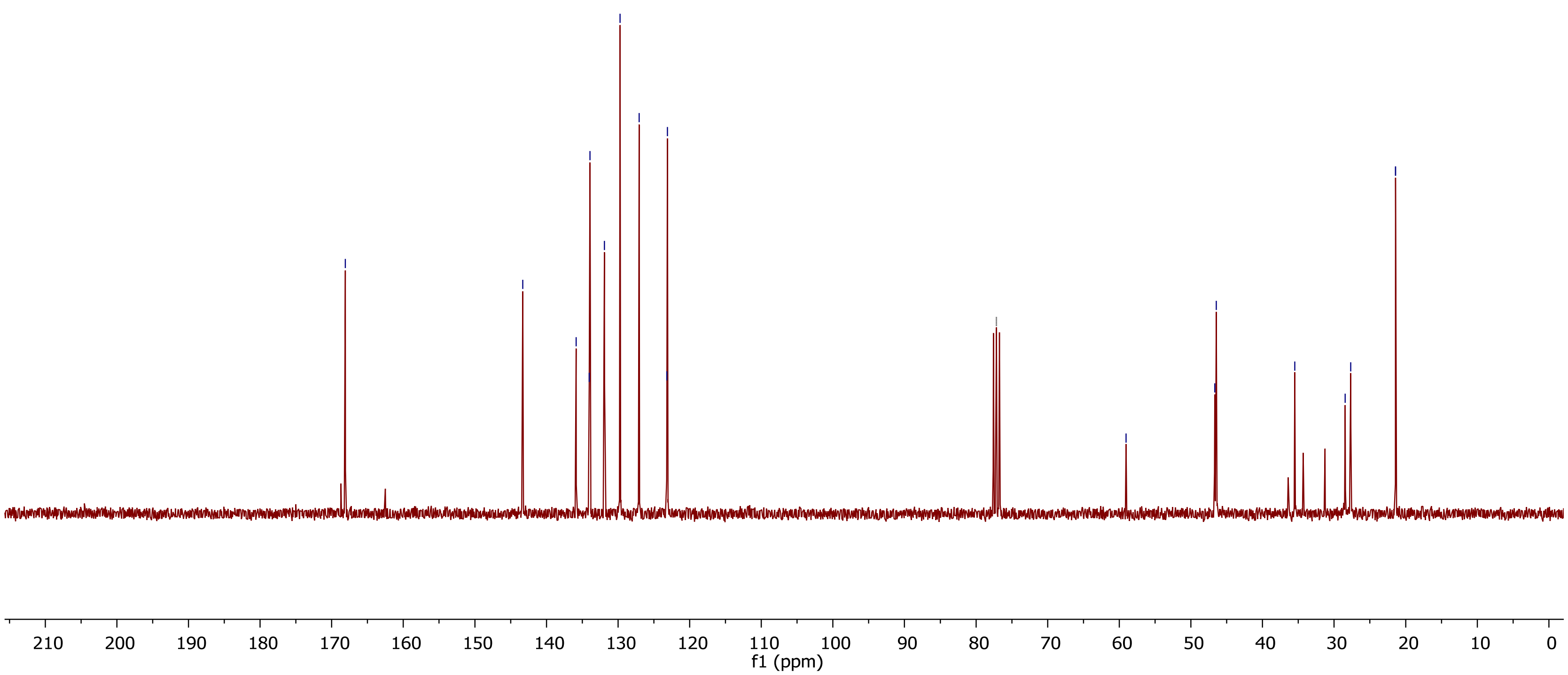

^{1}H-^{13}C HSQC, $CDCl_3$, 298K

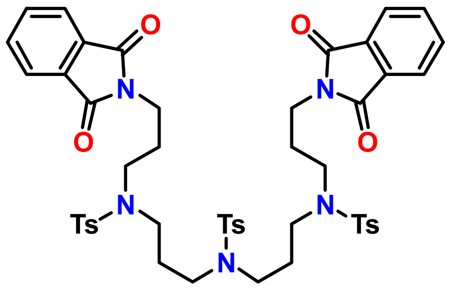

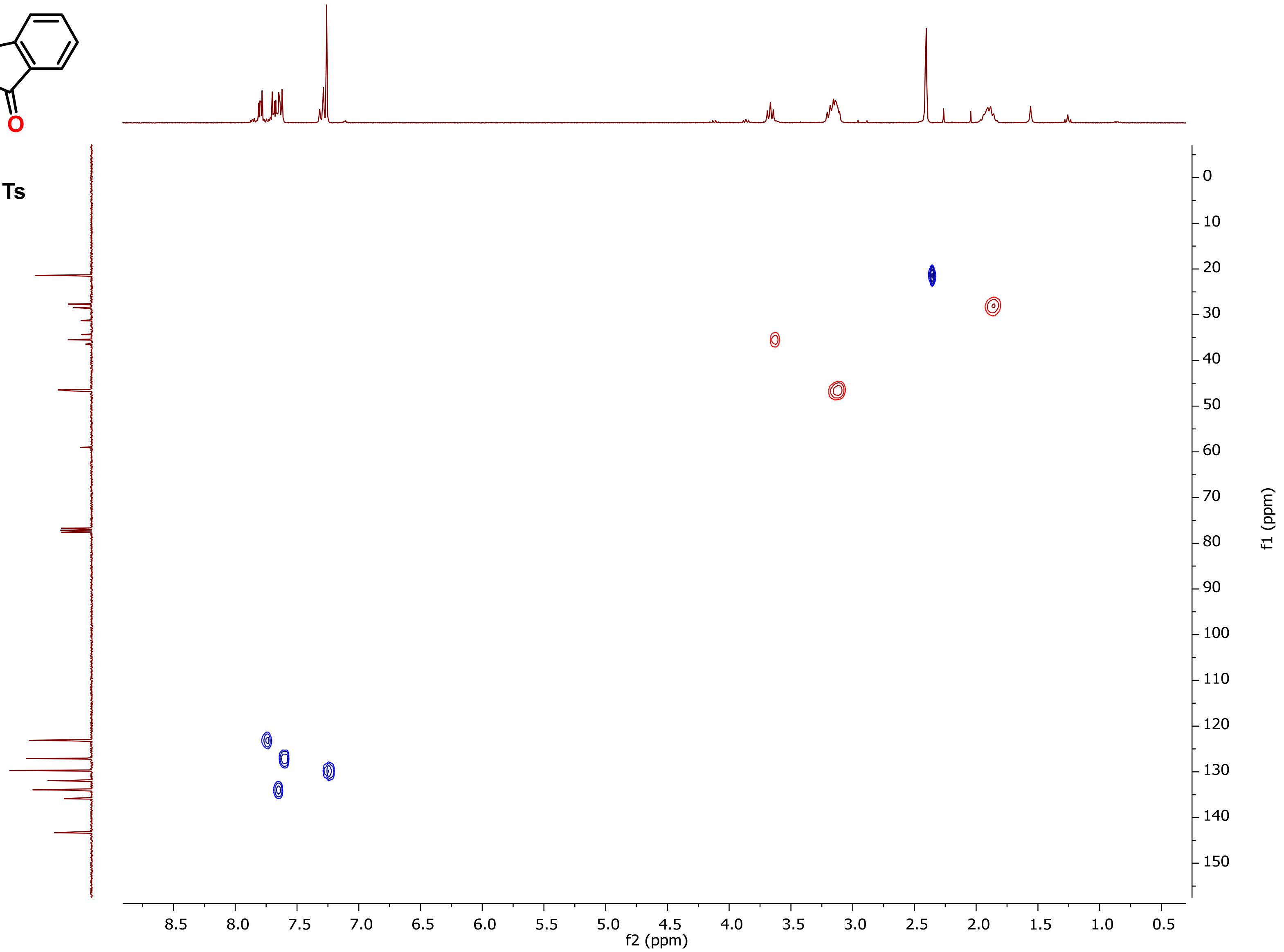

^{1}H NMR, $CDCl_3$, 298K

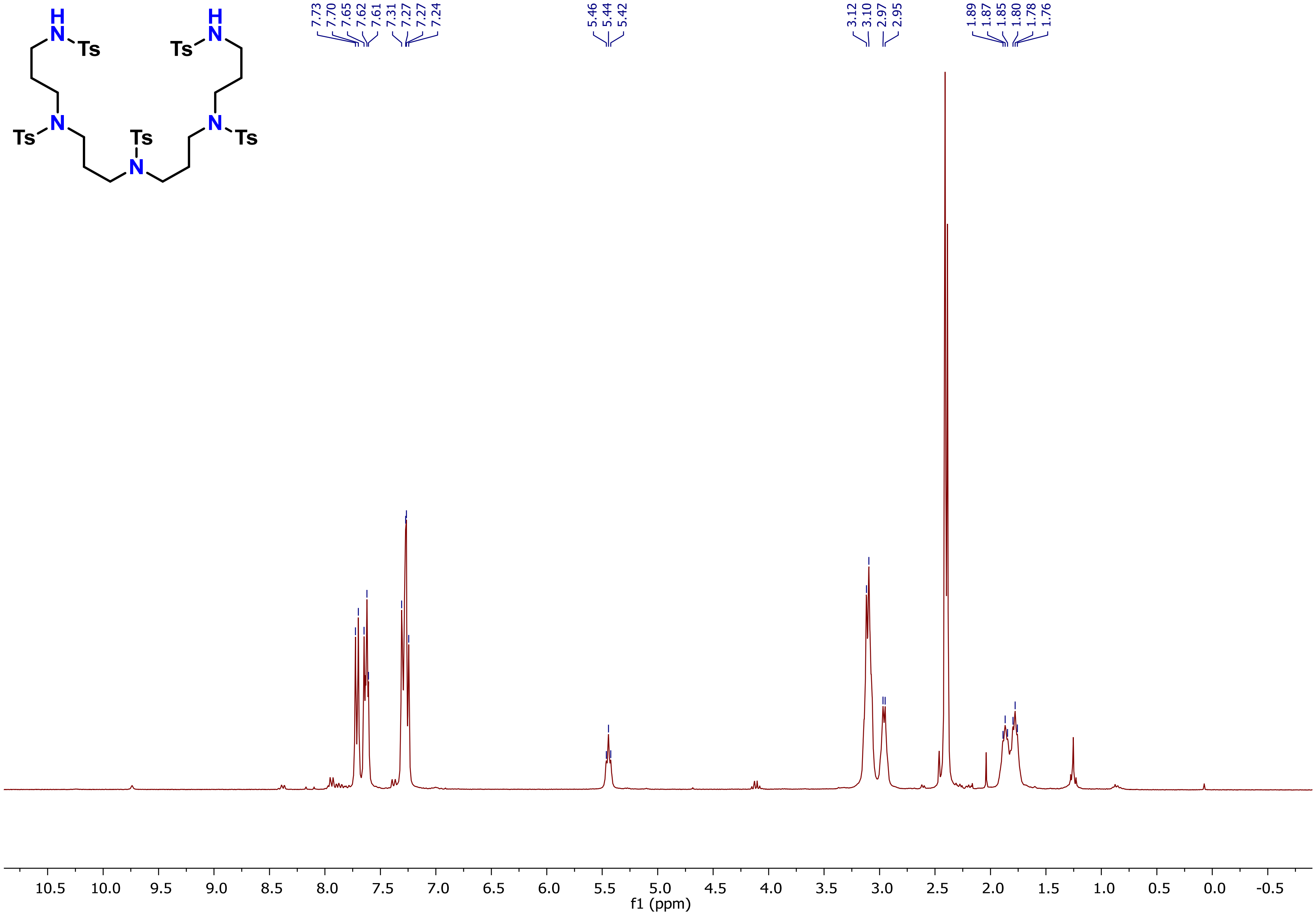

^{13}C NMR, $CDCl_3$, 298K

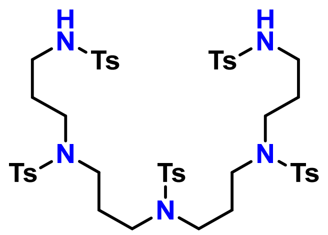

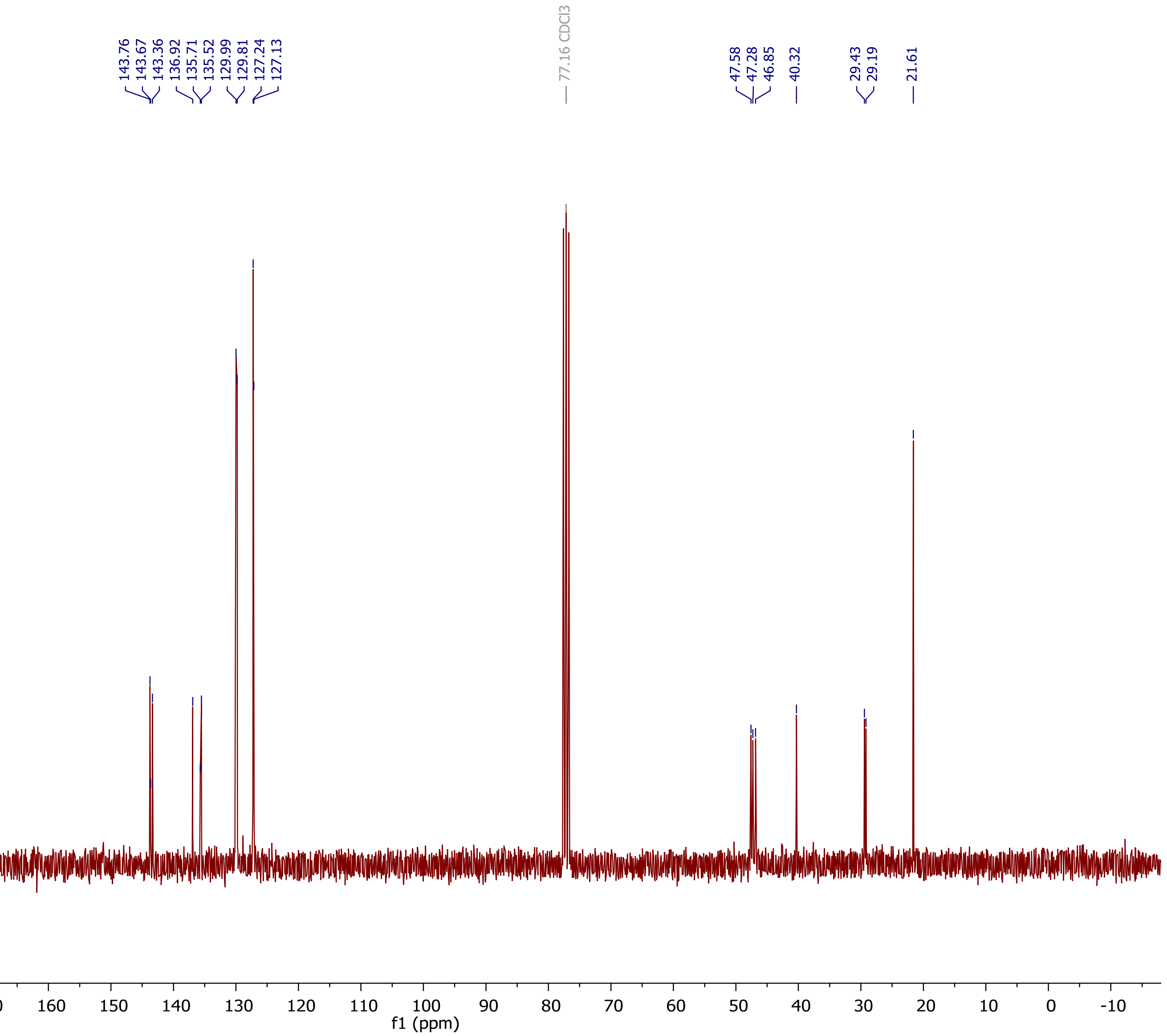

^1H-^{13}C HSQC, $CDCl_3$, 298K

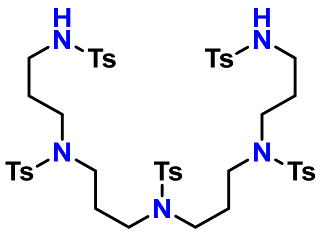

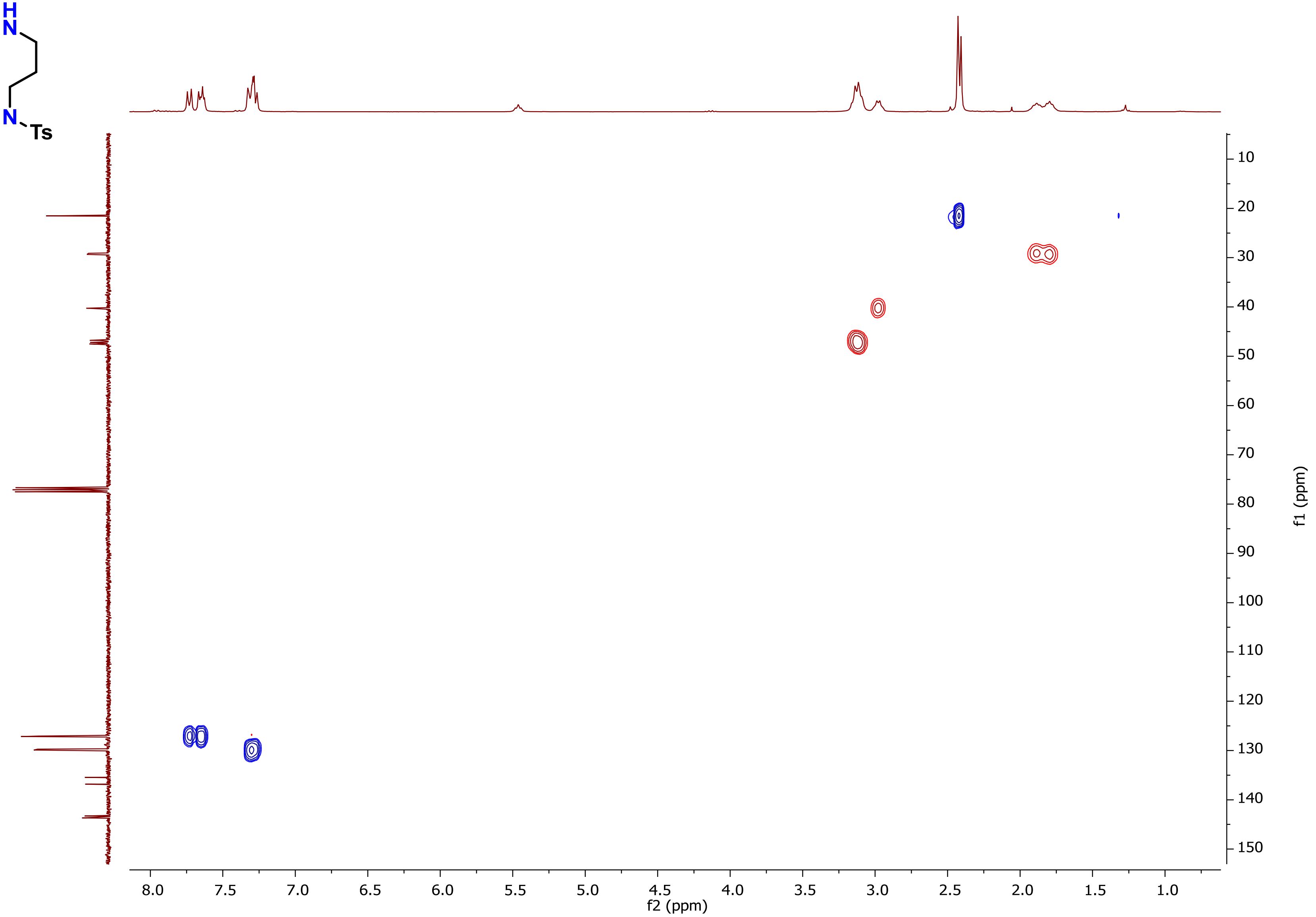

^{1}H NMR, $CDCl_3$, 298K

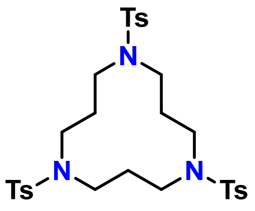

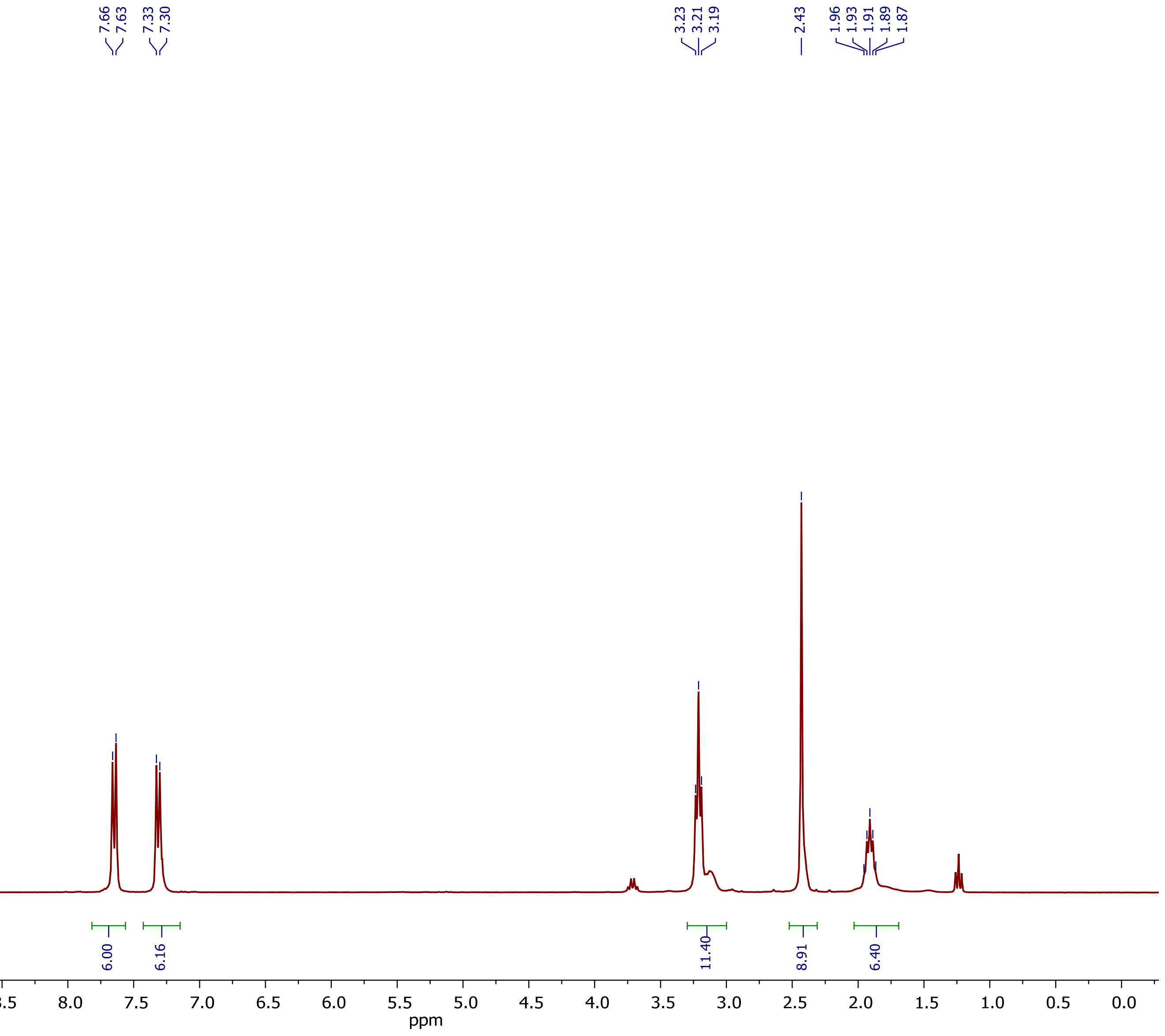

^{13}C NMR, $CDCl_3$, 298K

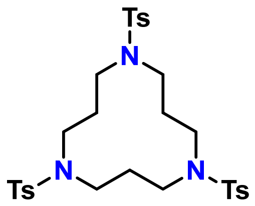

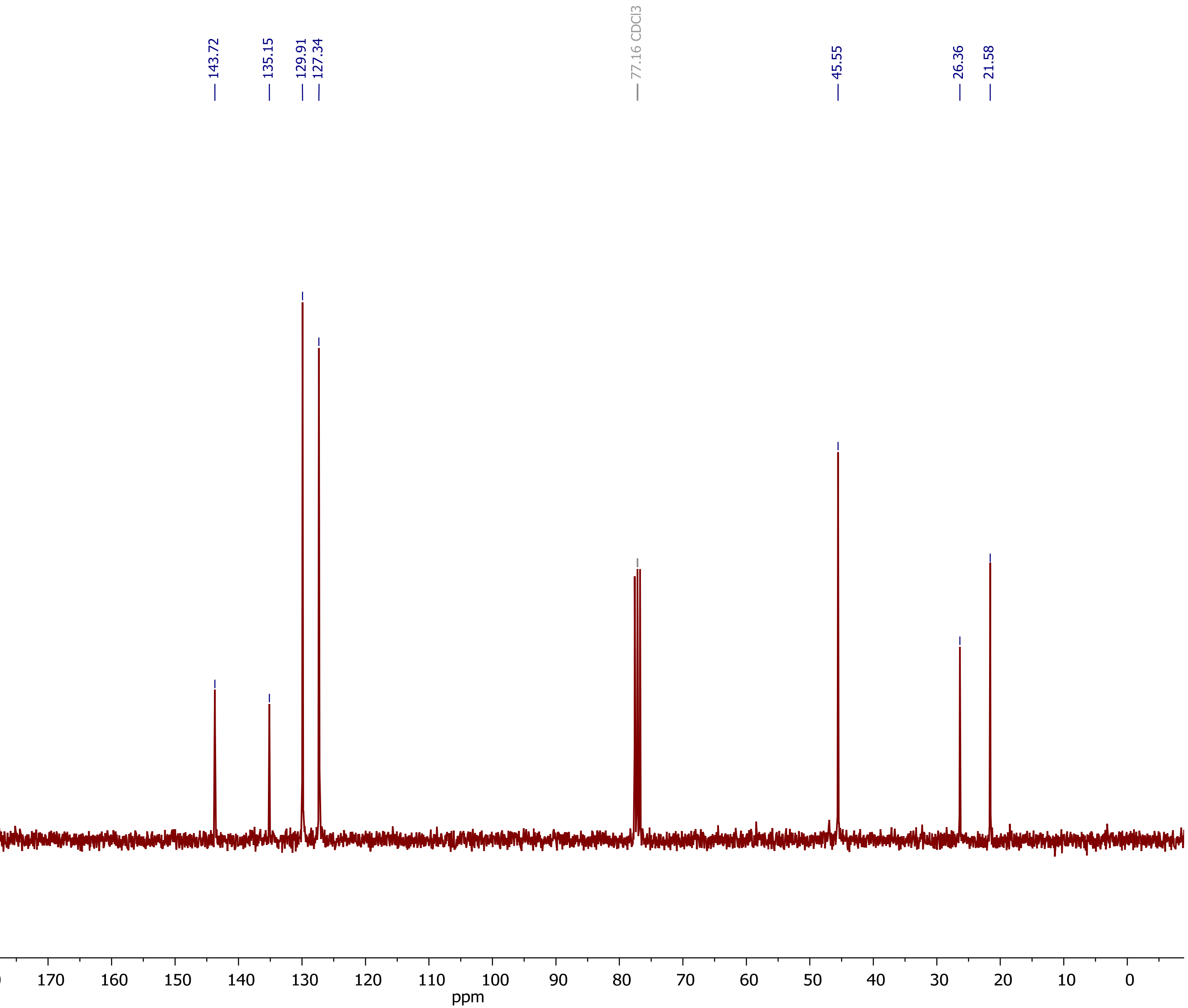

^{1}H NMR, $CDCl_3$, 298K

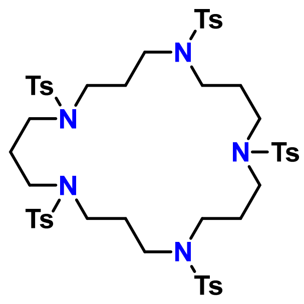

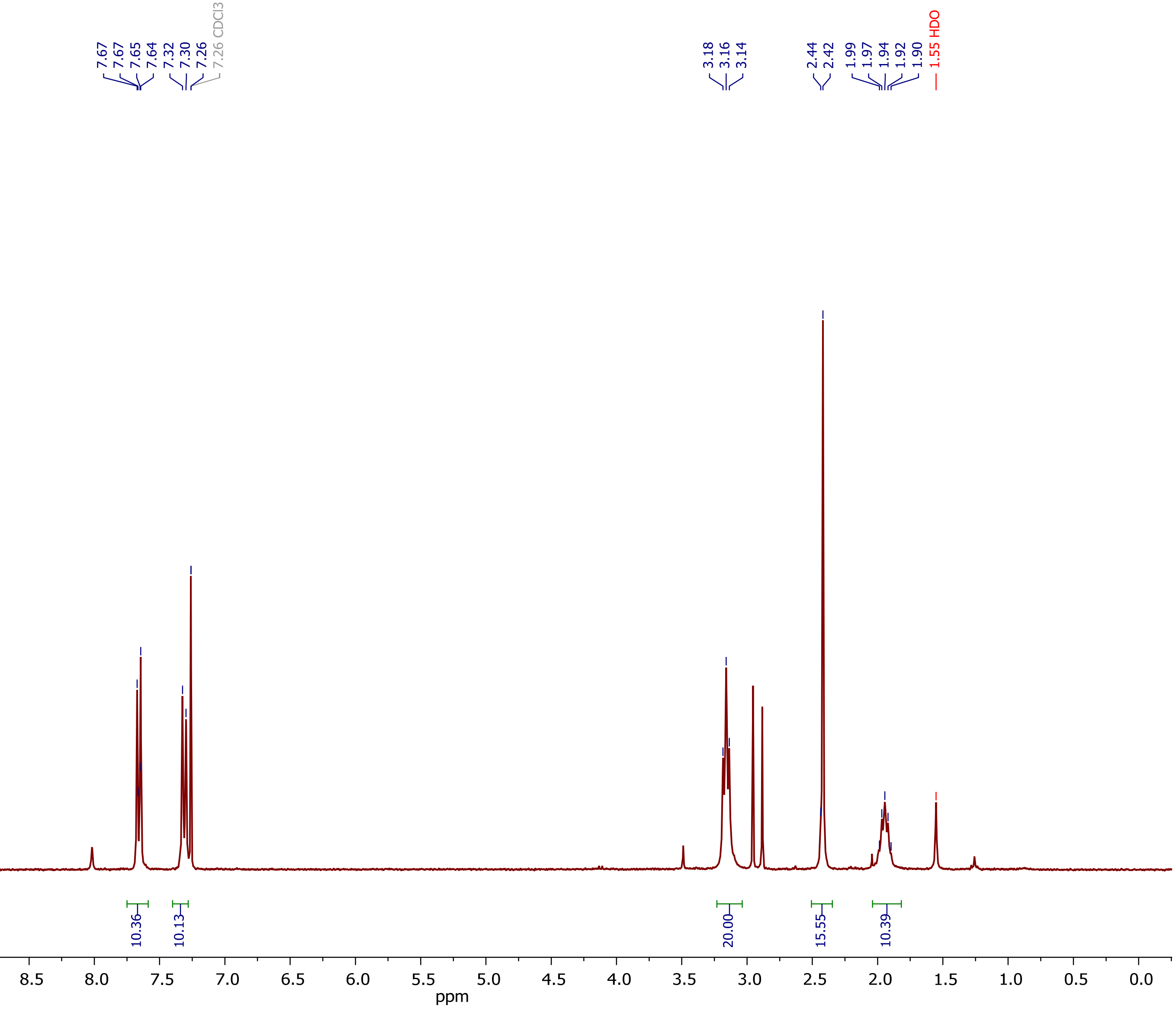

^{1}H NMR, $CDCl_3$, 298K

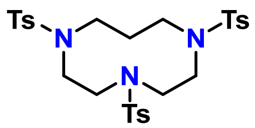

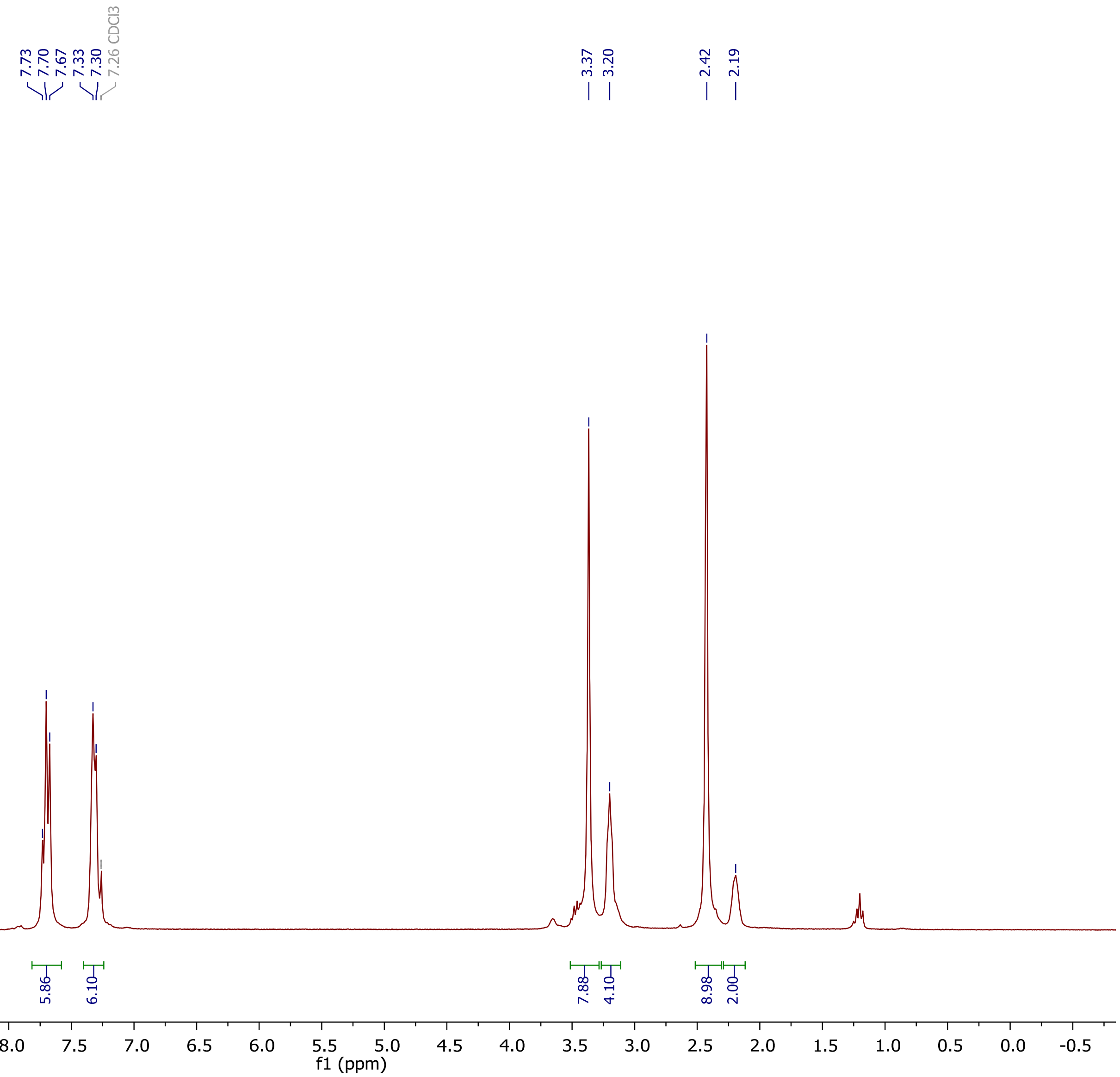

^{13}C NMR, $CDCl_3$, 298K

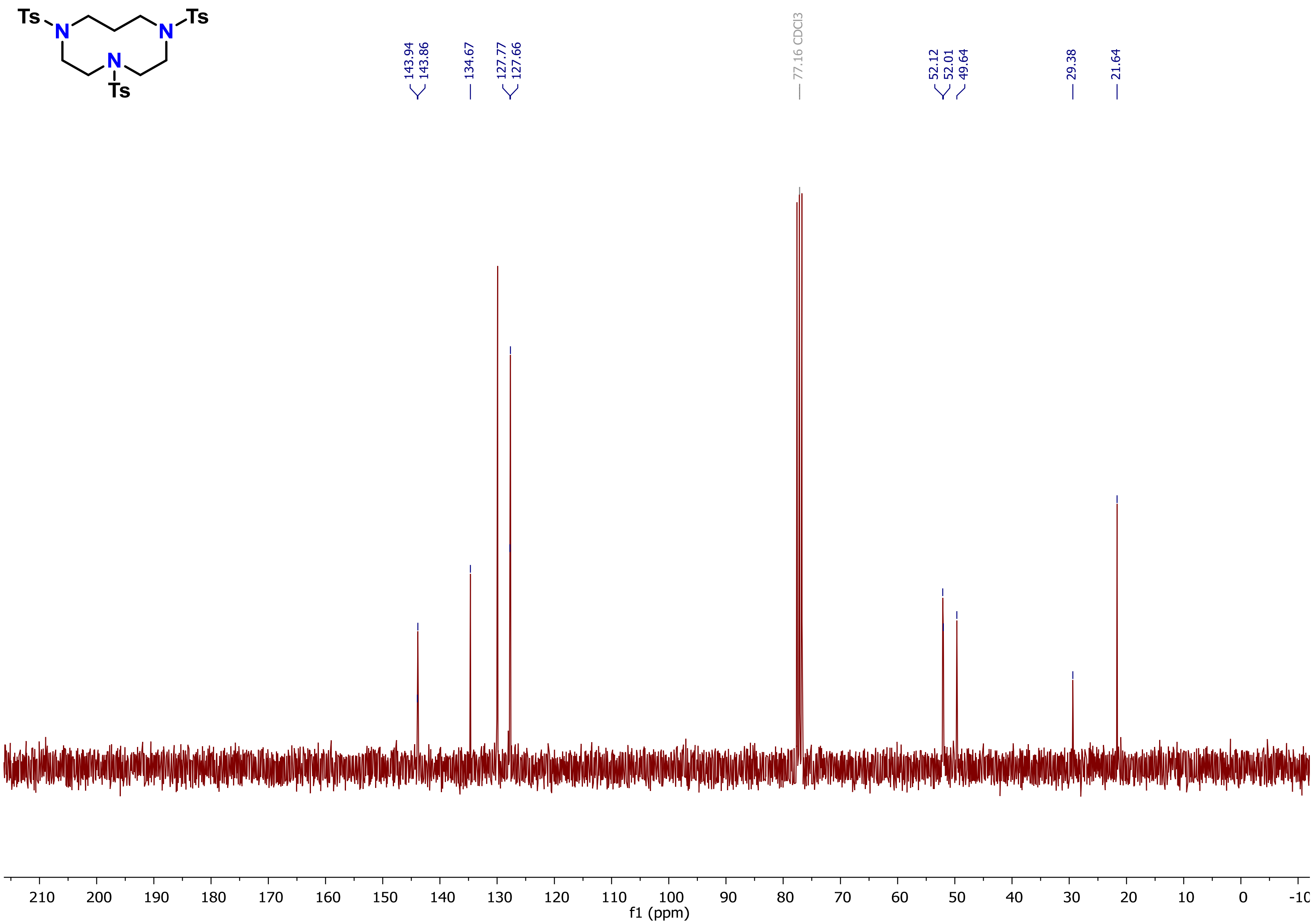

^{1}H-^{13}C HSQC, $CDCl_3$, 298K

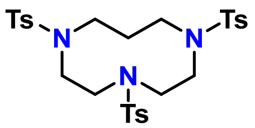

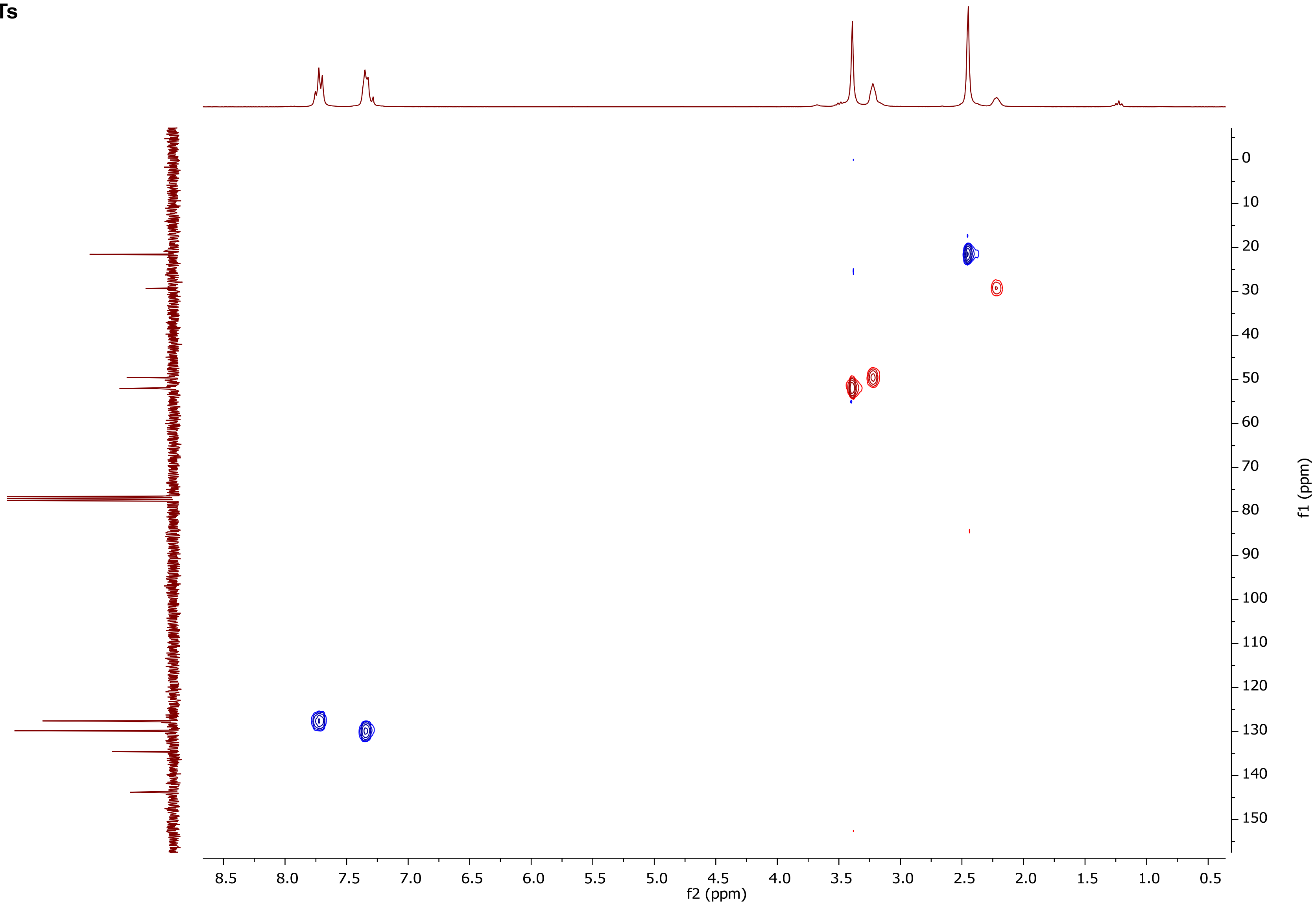

^{1}H NMR, $CDCl_3$, 298K

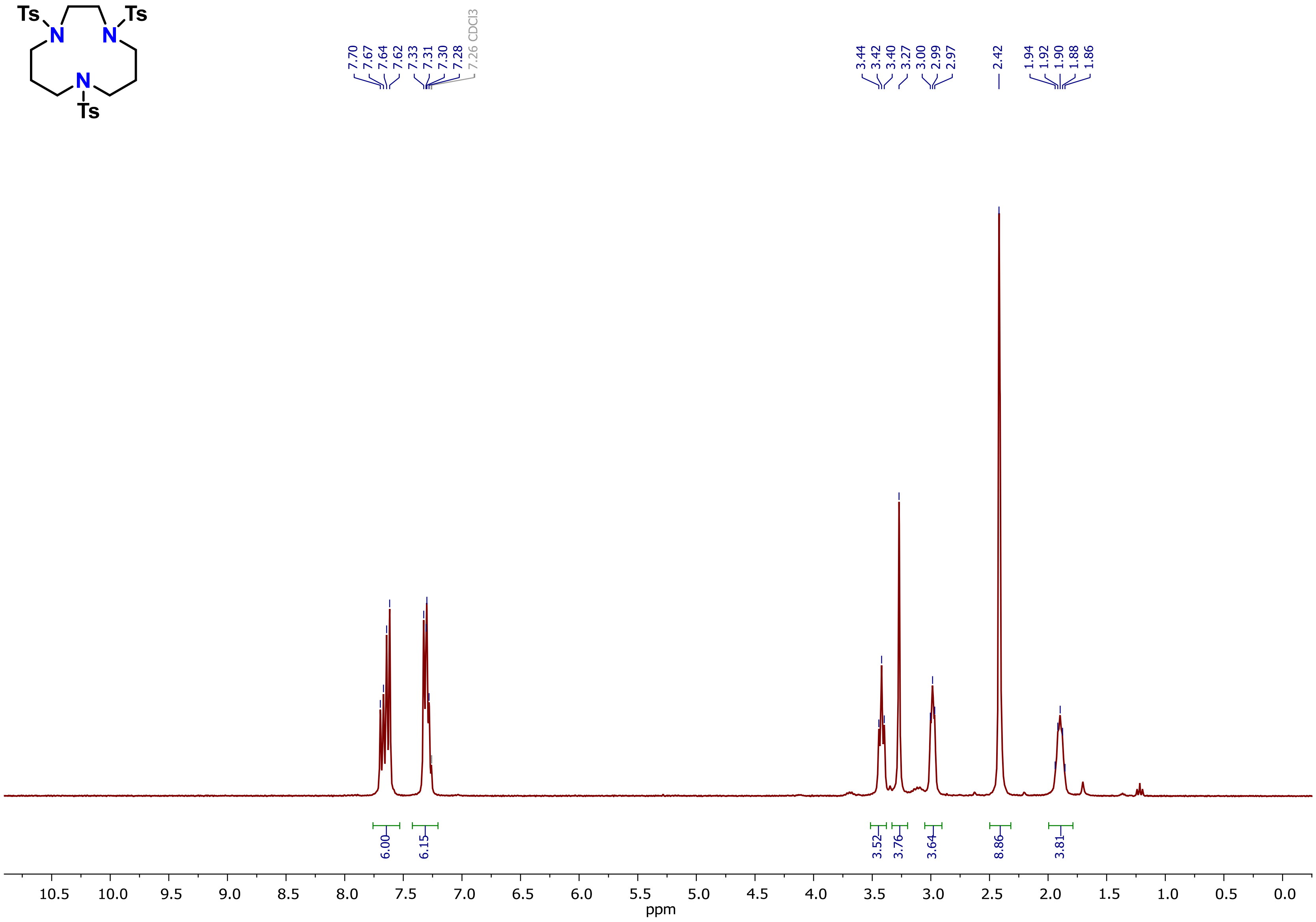

^{13}C NMR, $CDCl_3$, 298K

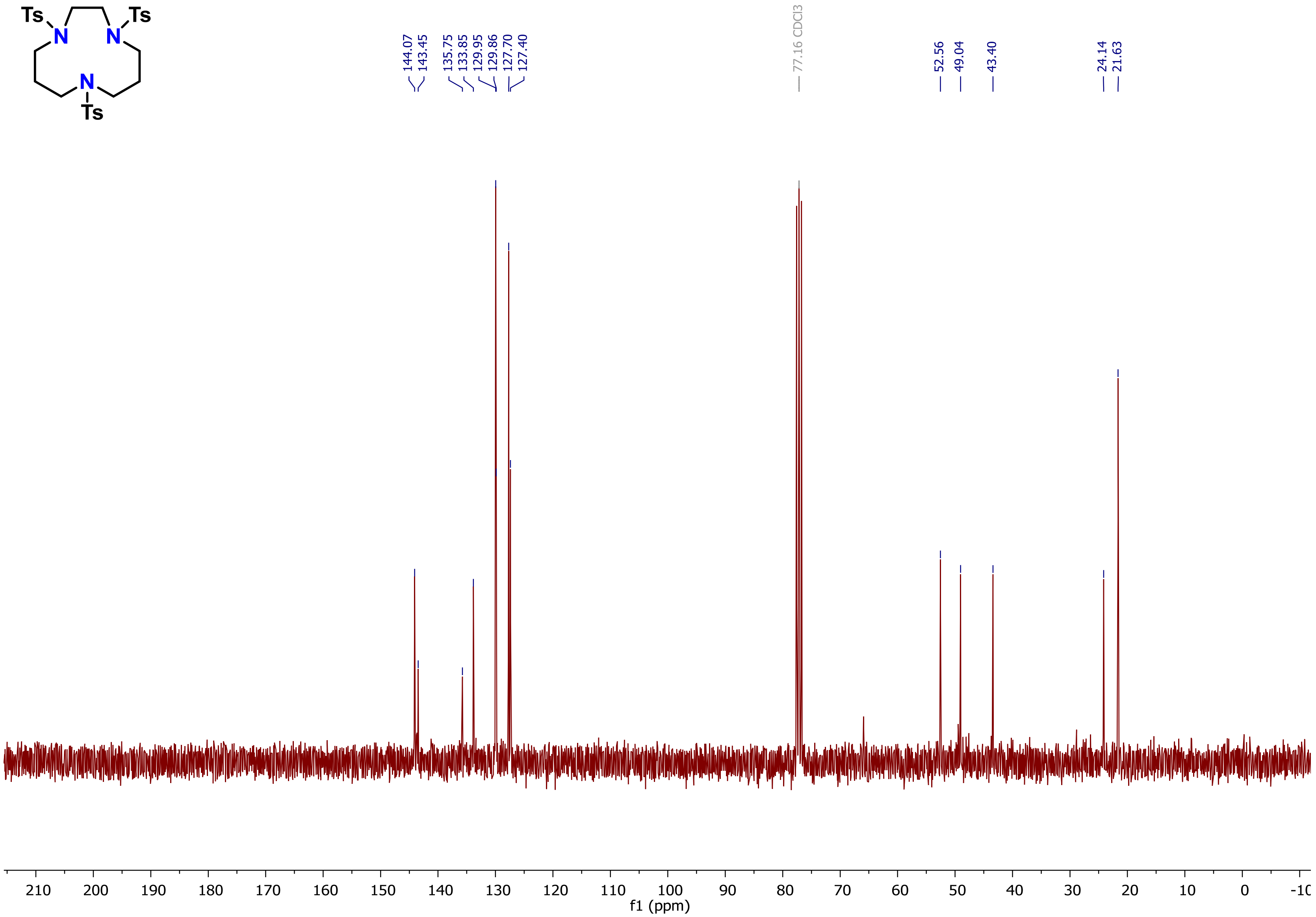

^{1}H-^{13}C HSQC, $CDCl_3$, 298K

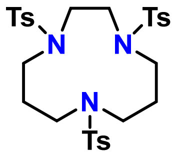

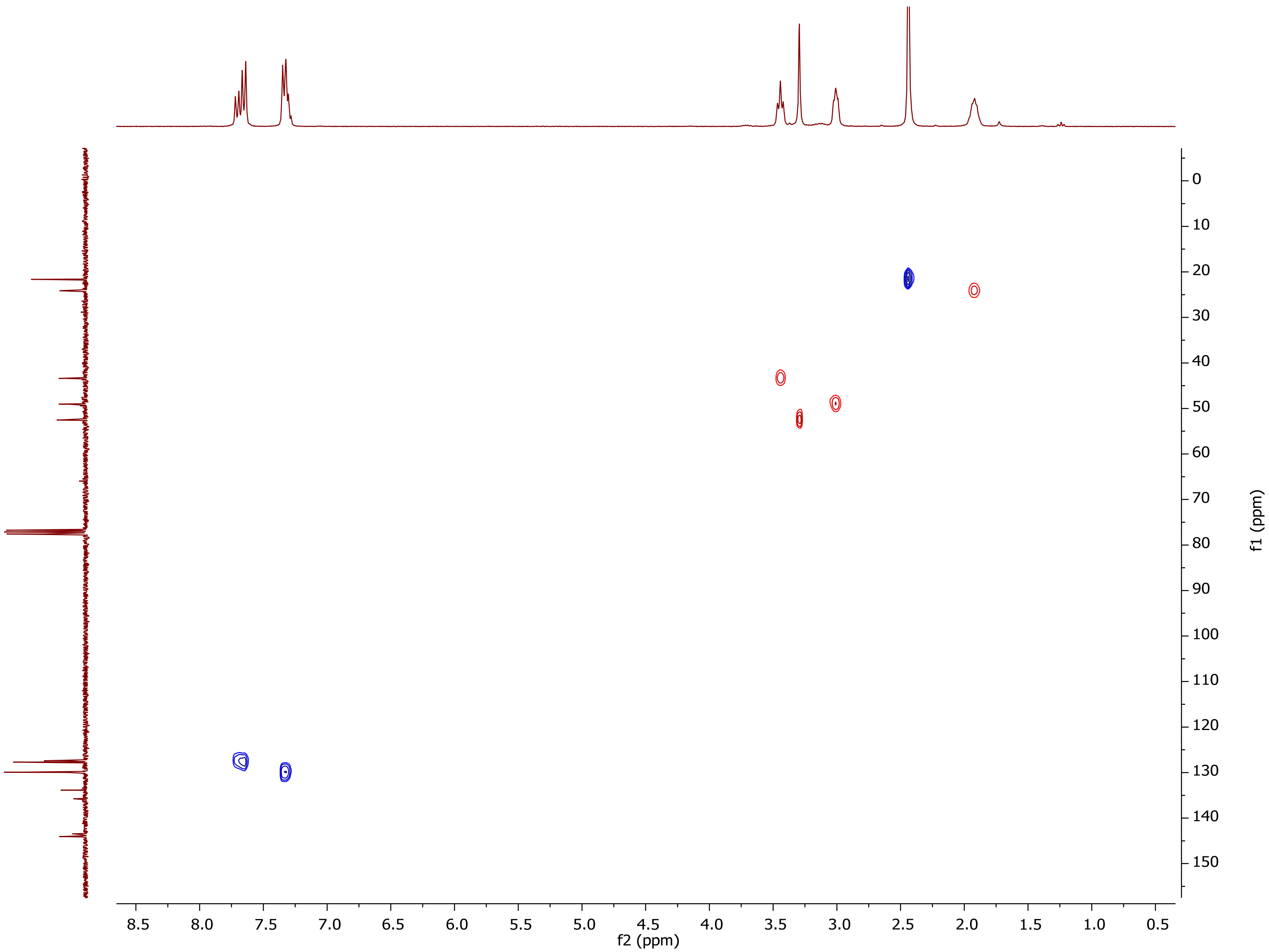

^{1}H NMR, D_2O, 298K

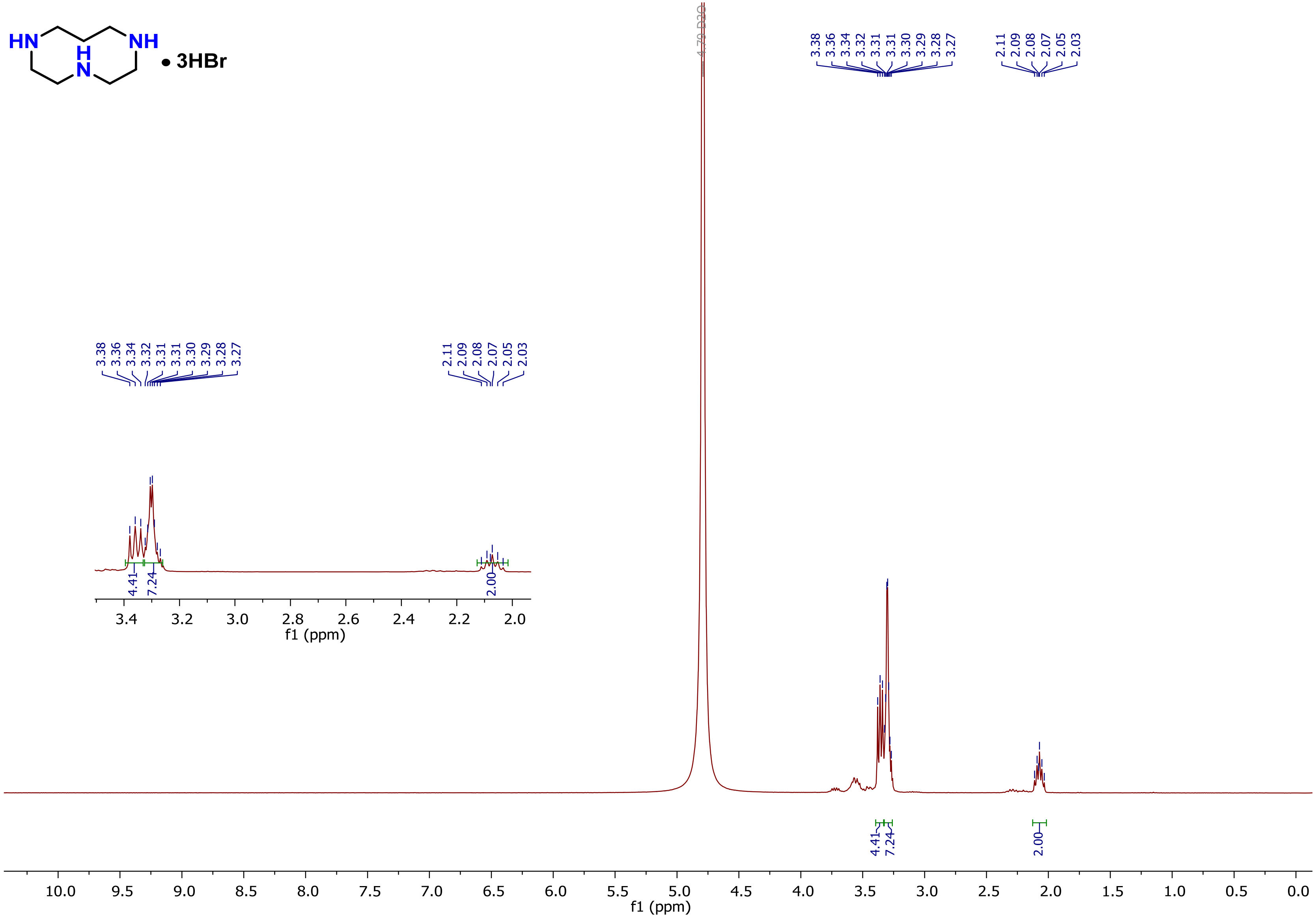

^{13}C NMR, D_2O, 298K

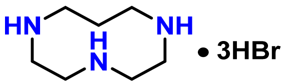

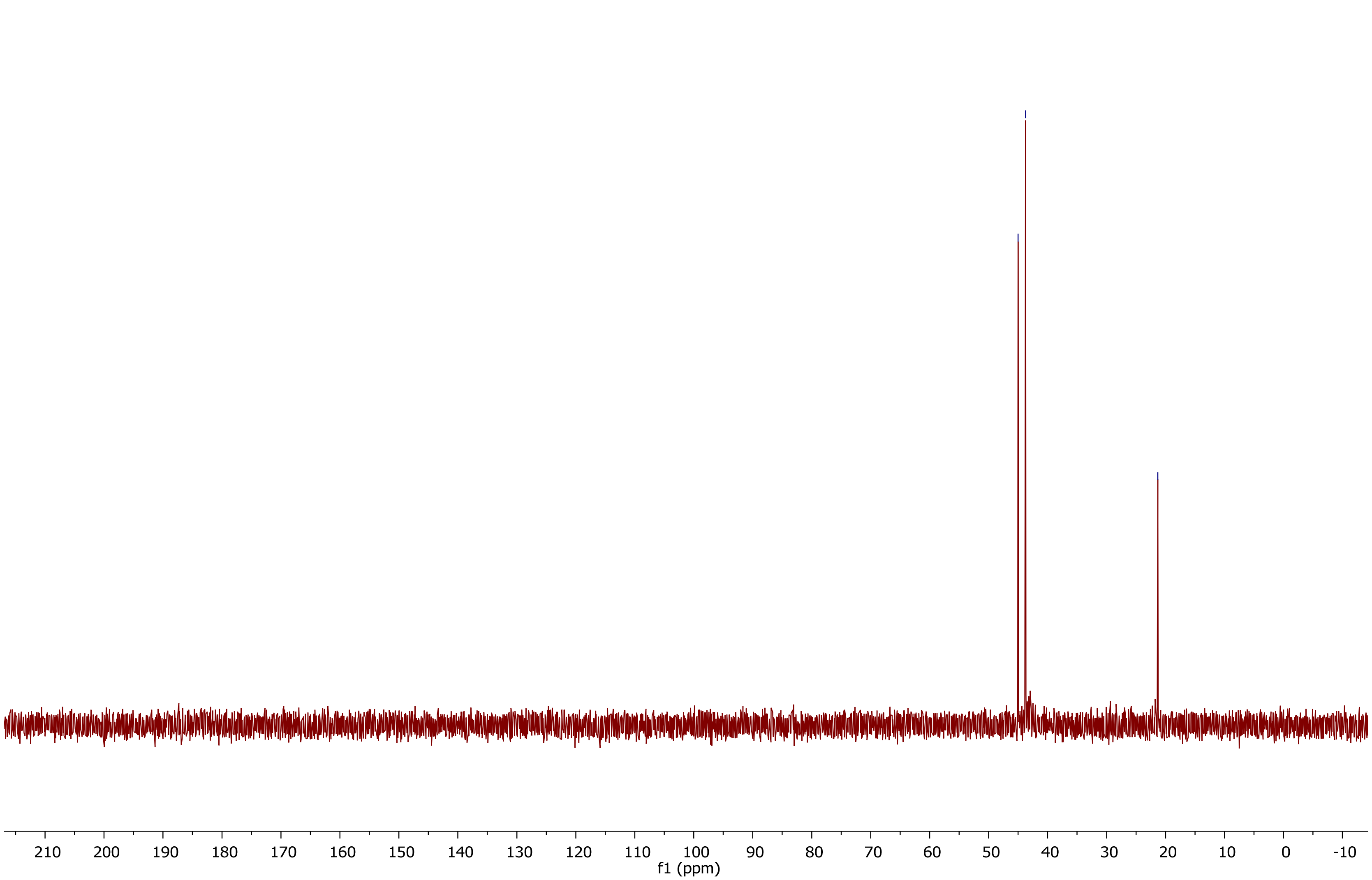

^{1}H-^{13}C HSQC, D_2O, 298K

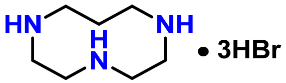

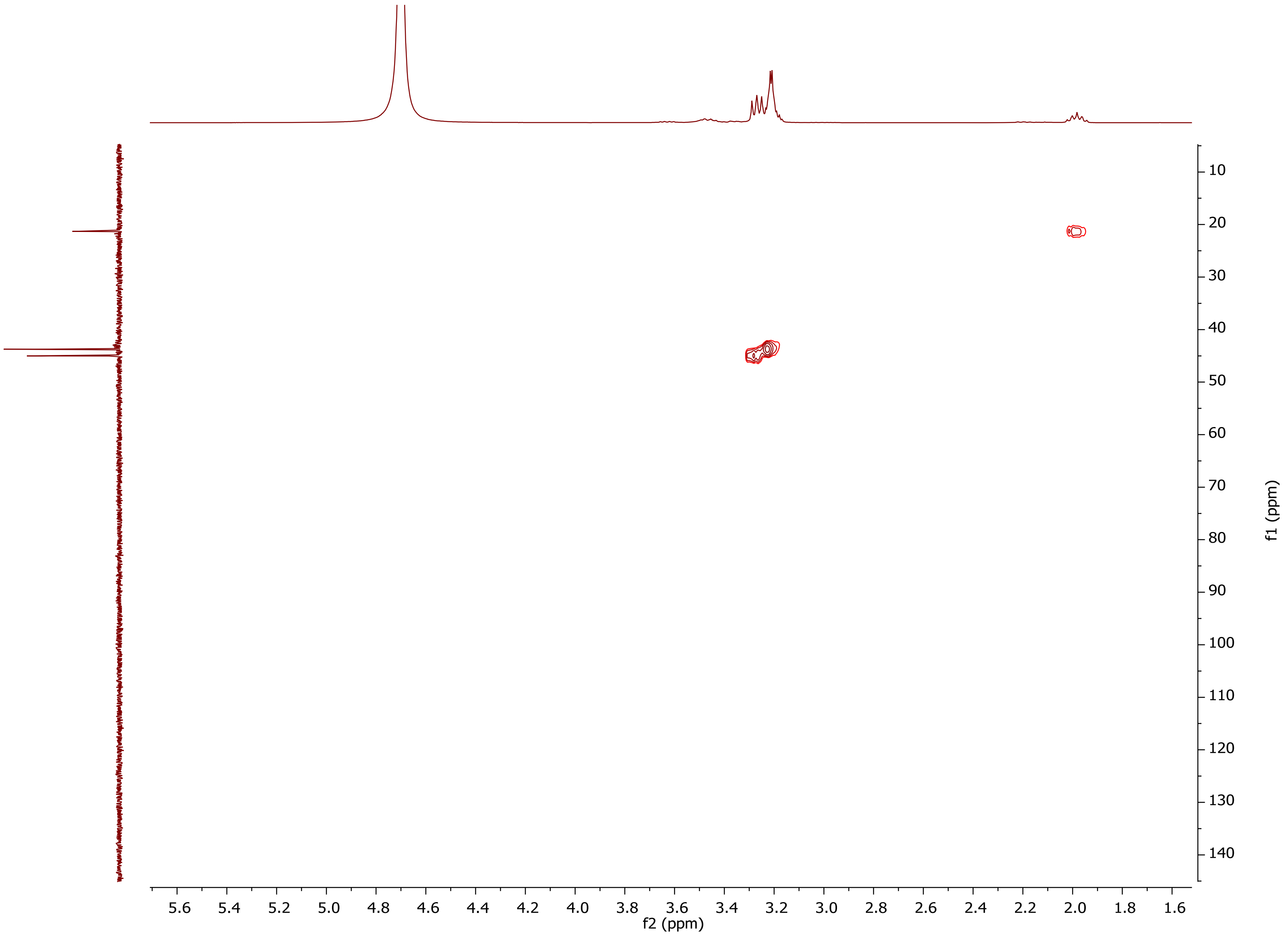

^{1}H NMR, D_2O, 298K

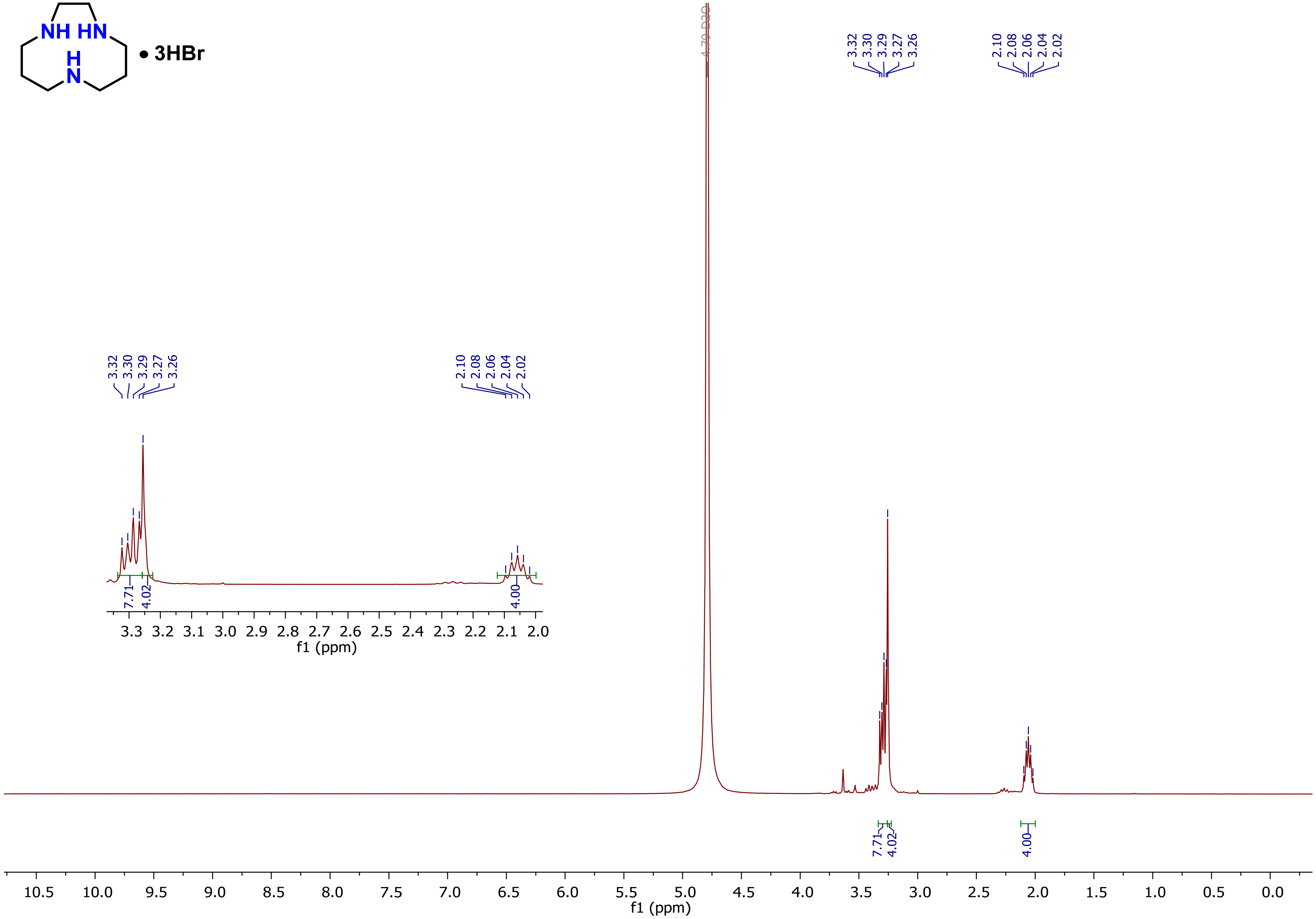

^{13}C NMR, D_2O, 298K

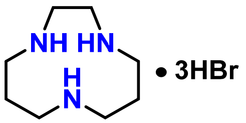

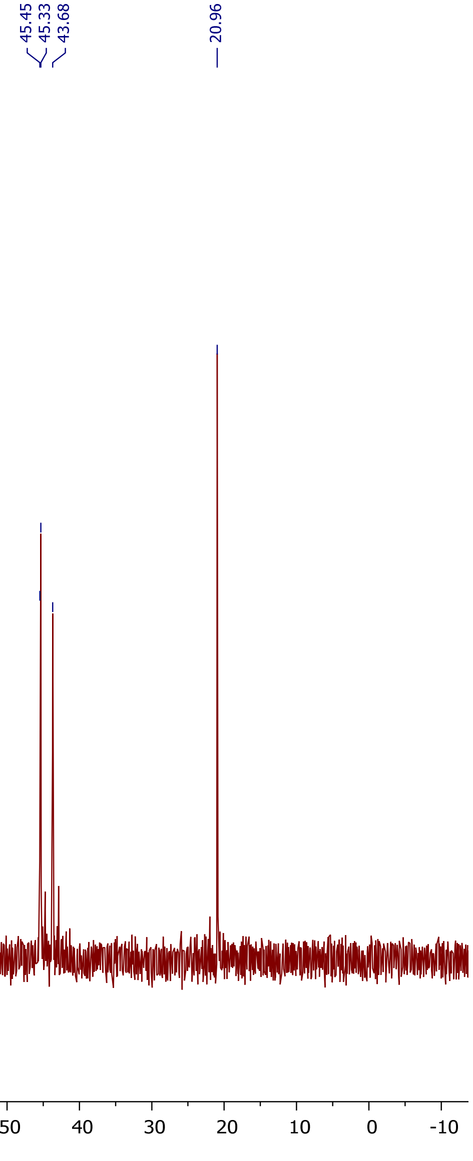

^{1}H-^{13}C HSQC, D_2O, 298K

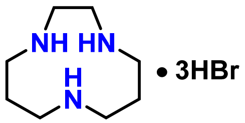

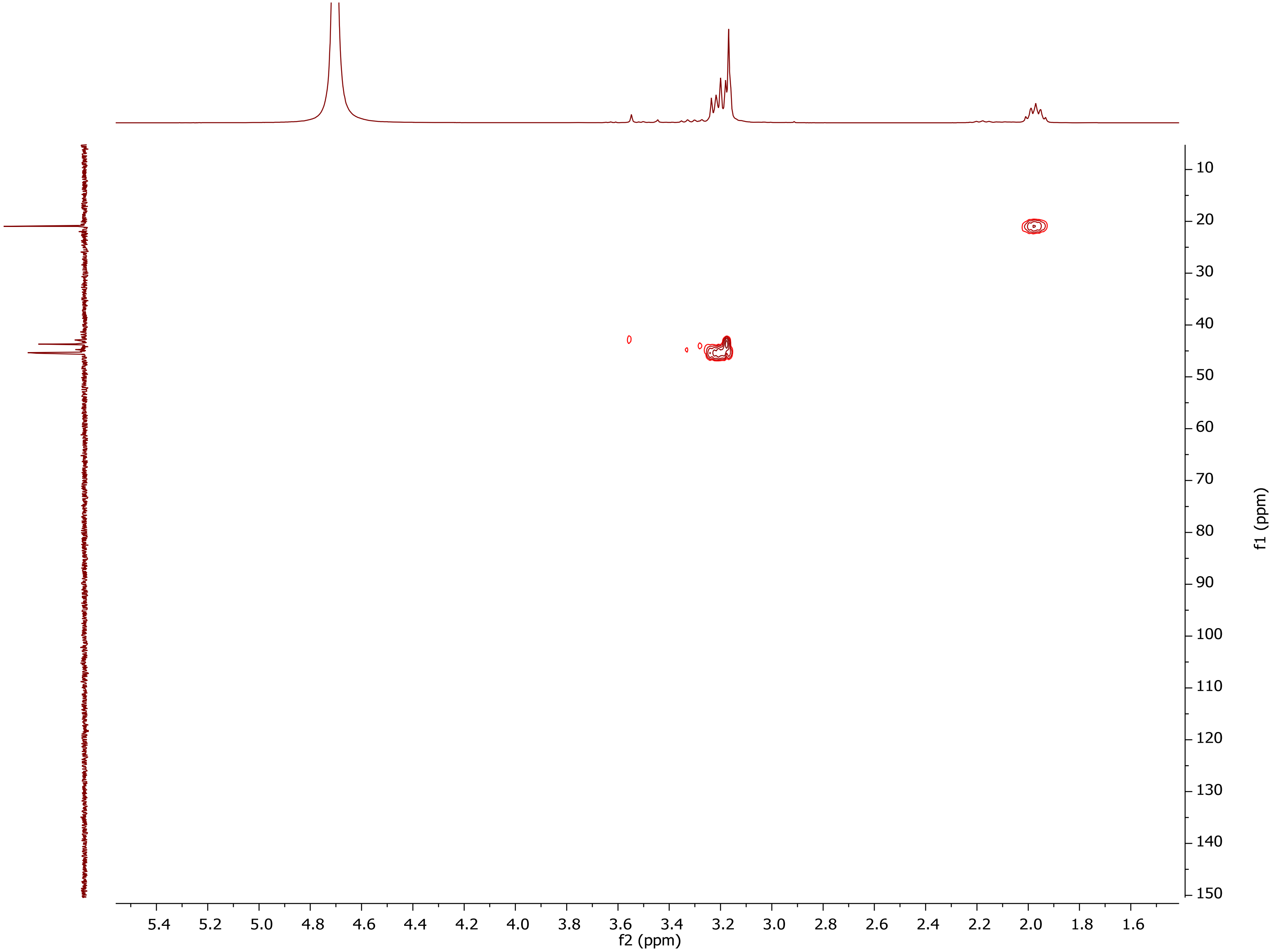

^{1}H NMR, D_2O, 298K

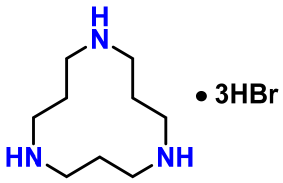

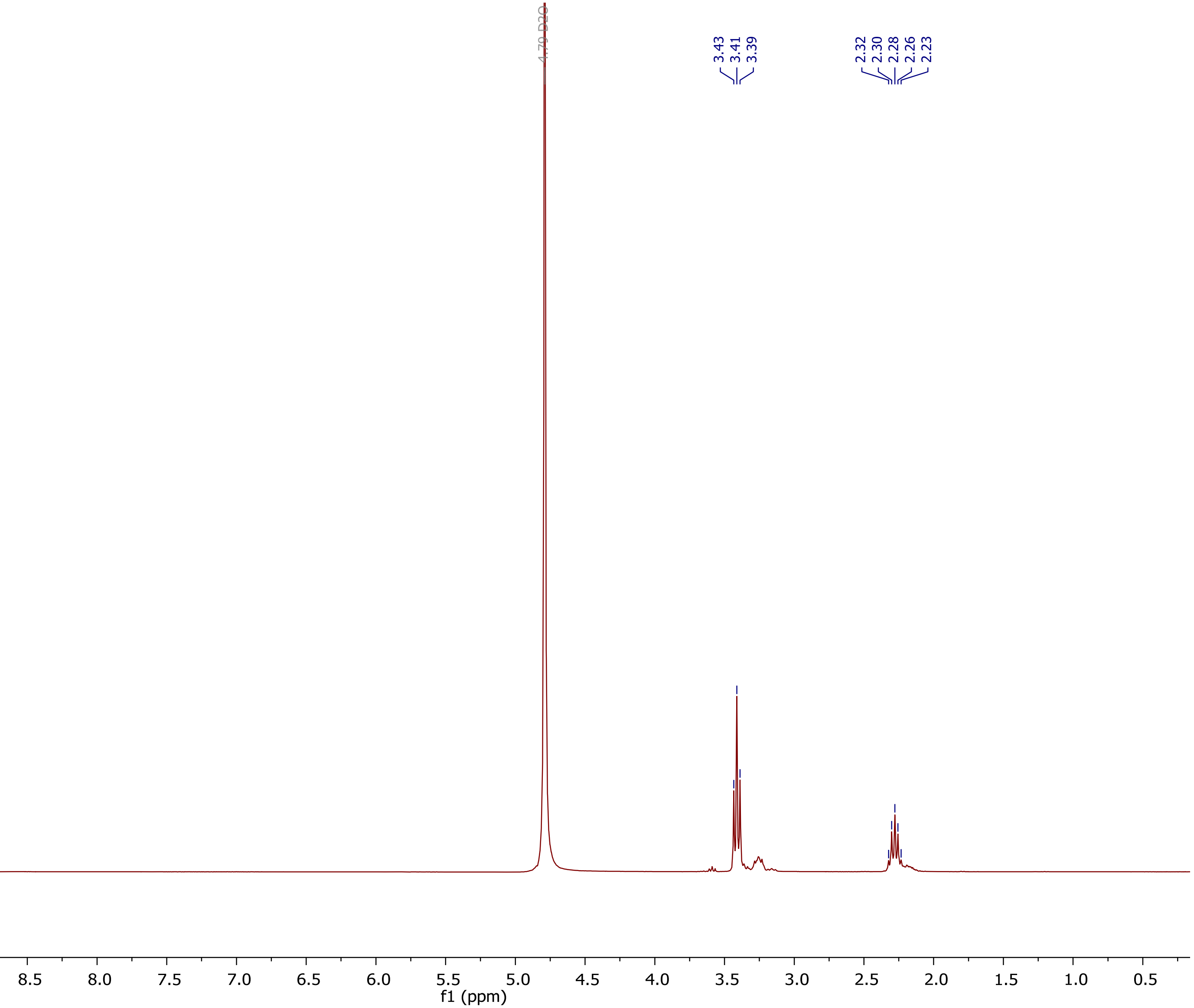

^{13}C NMR, D_2O, 298K

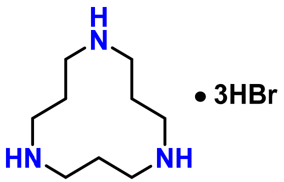

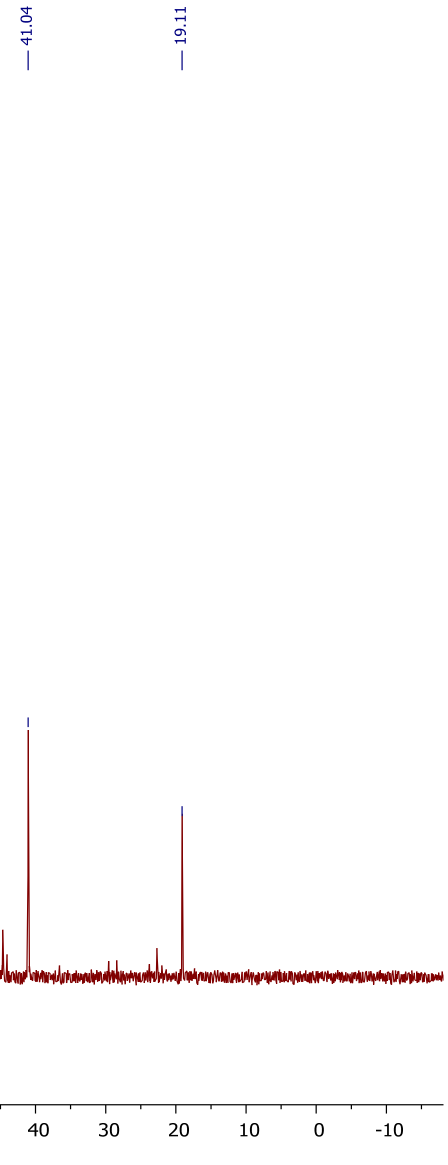

^{1}H-^{13}C HSQC, D_2O, 298K

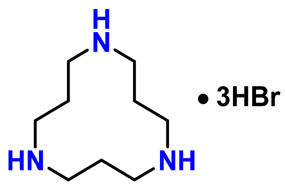

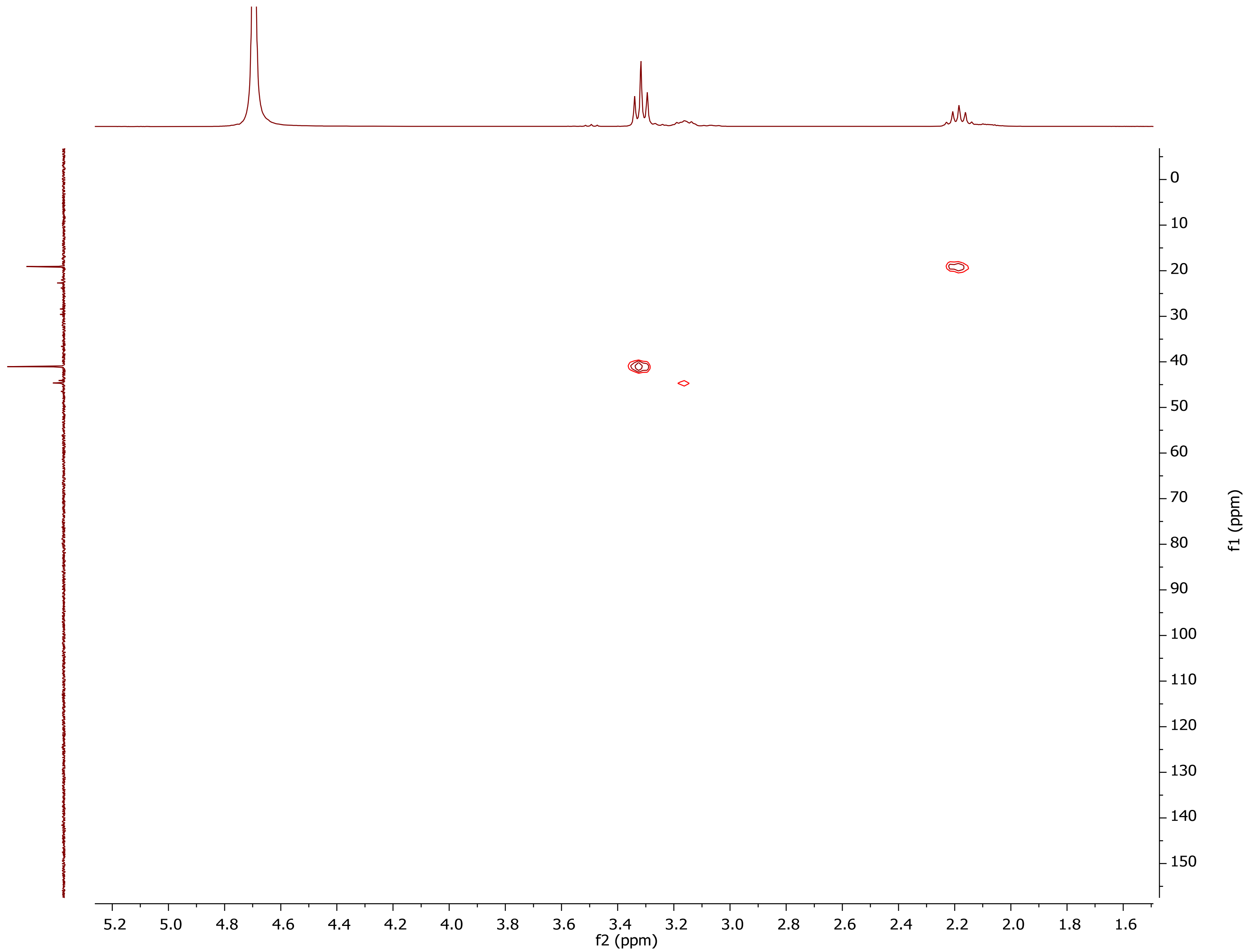

^{1}H NMR, D_2O, 298K

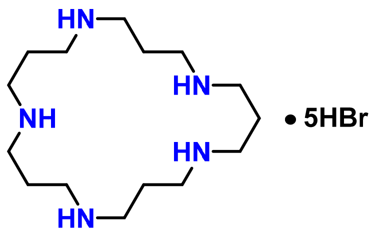

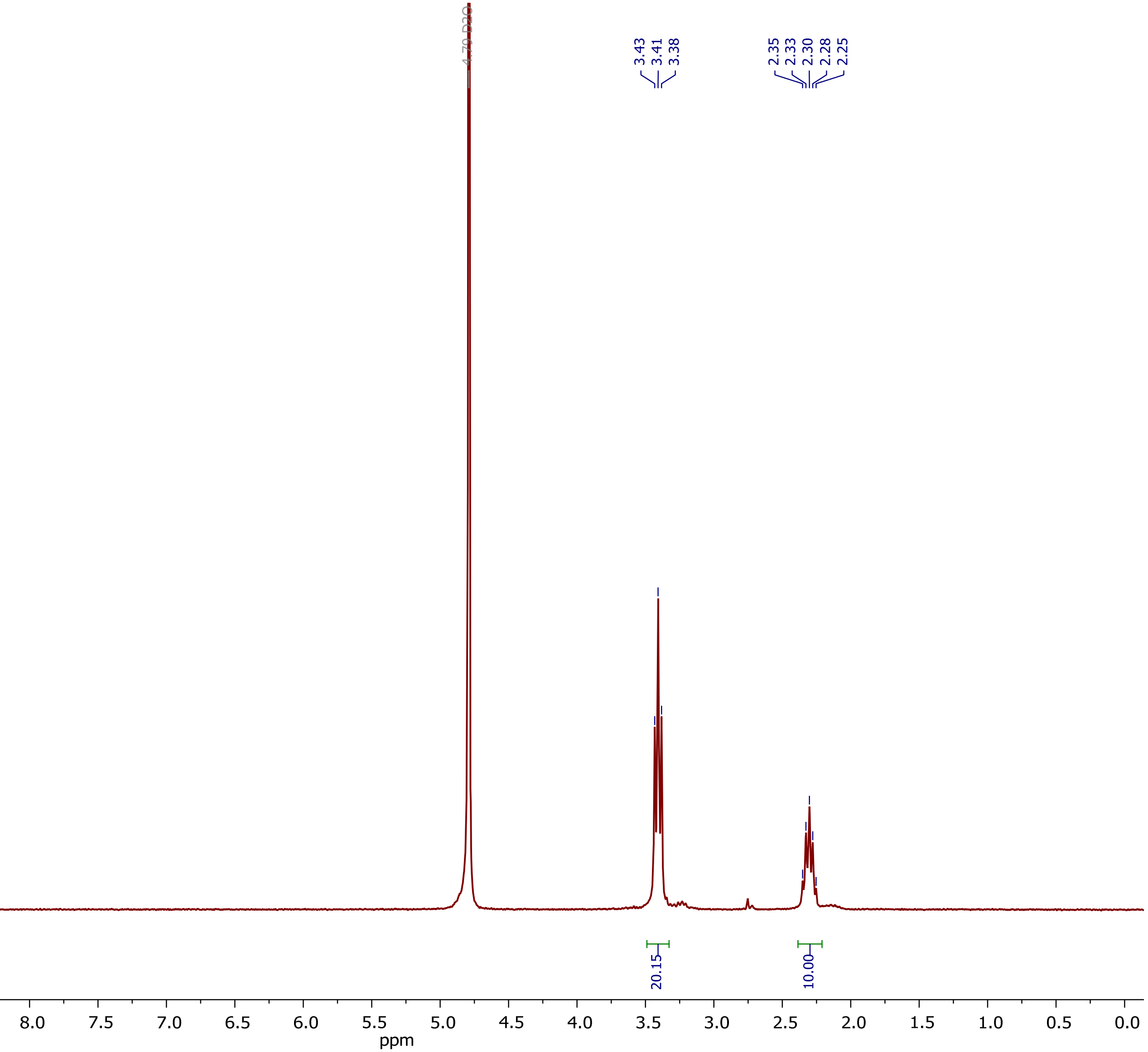

^{1}H NMR, $CDCl_3$, 298K

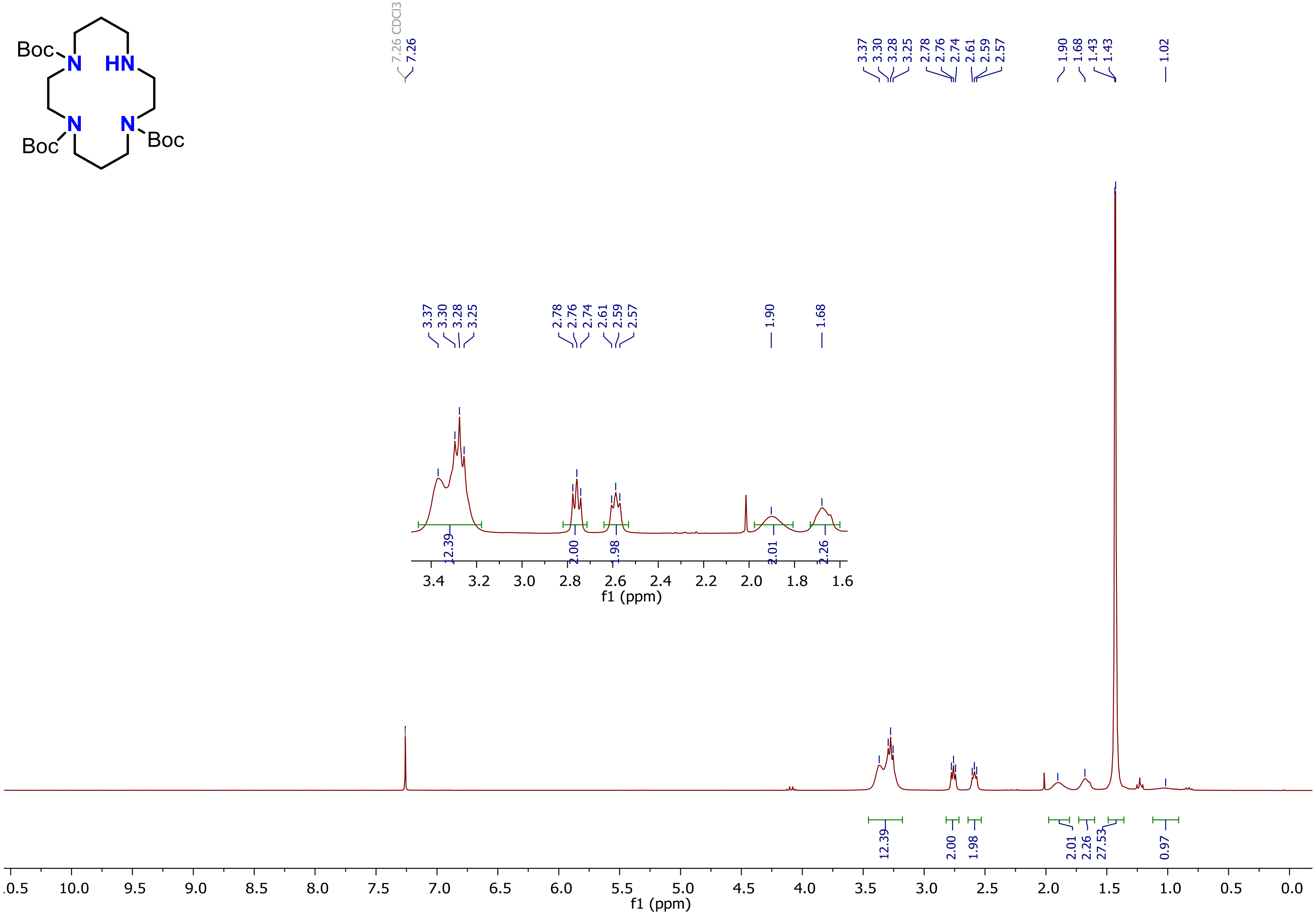

^{13}C NMR, $CDCl_3$, 298K

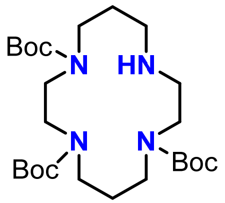

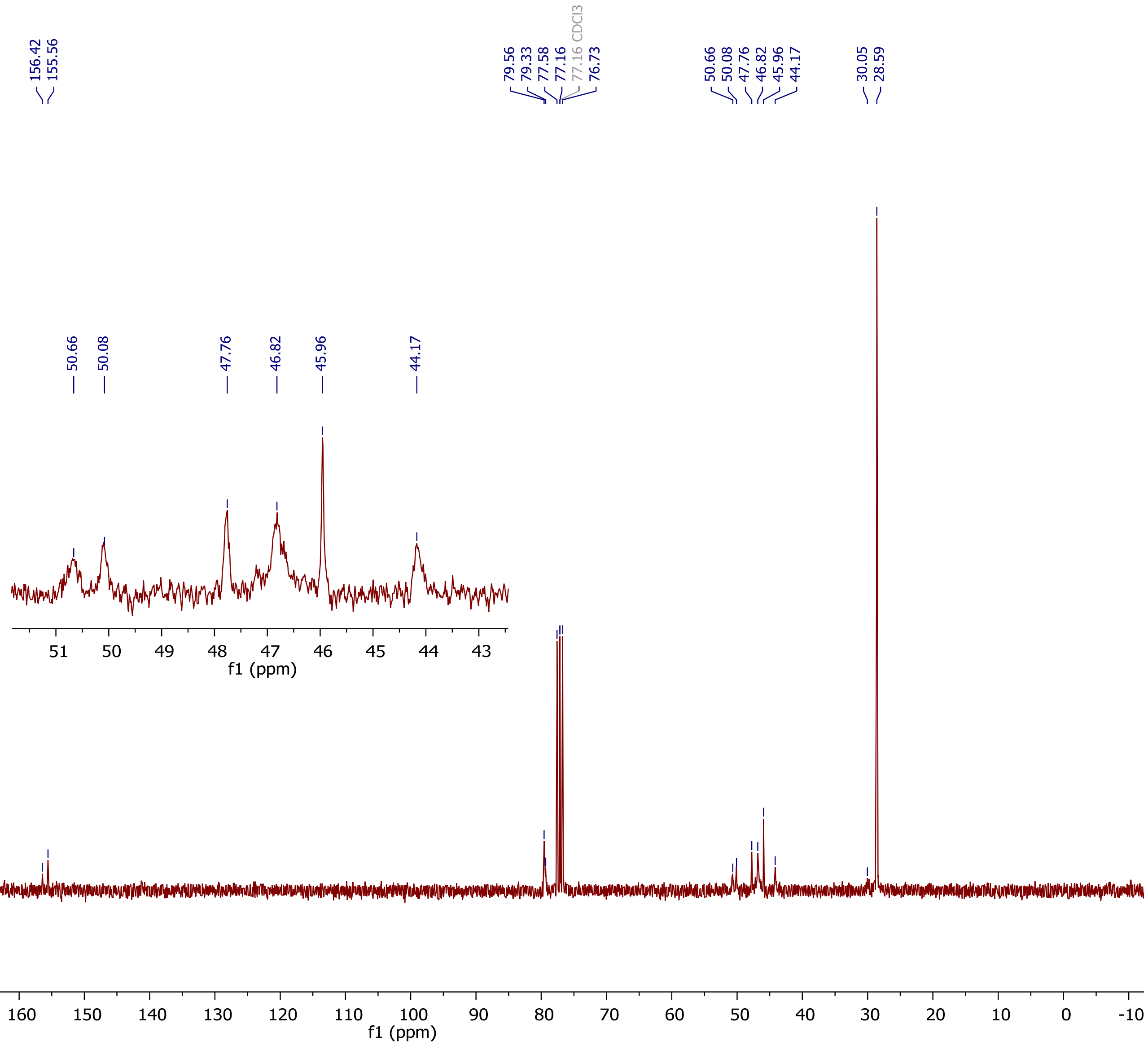

$^{1}H-^{13}C$ HSQC, $CDCl_3$, 298K

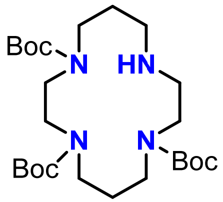

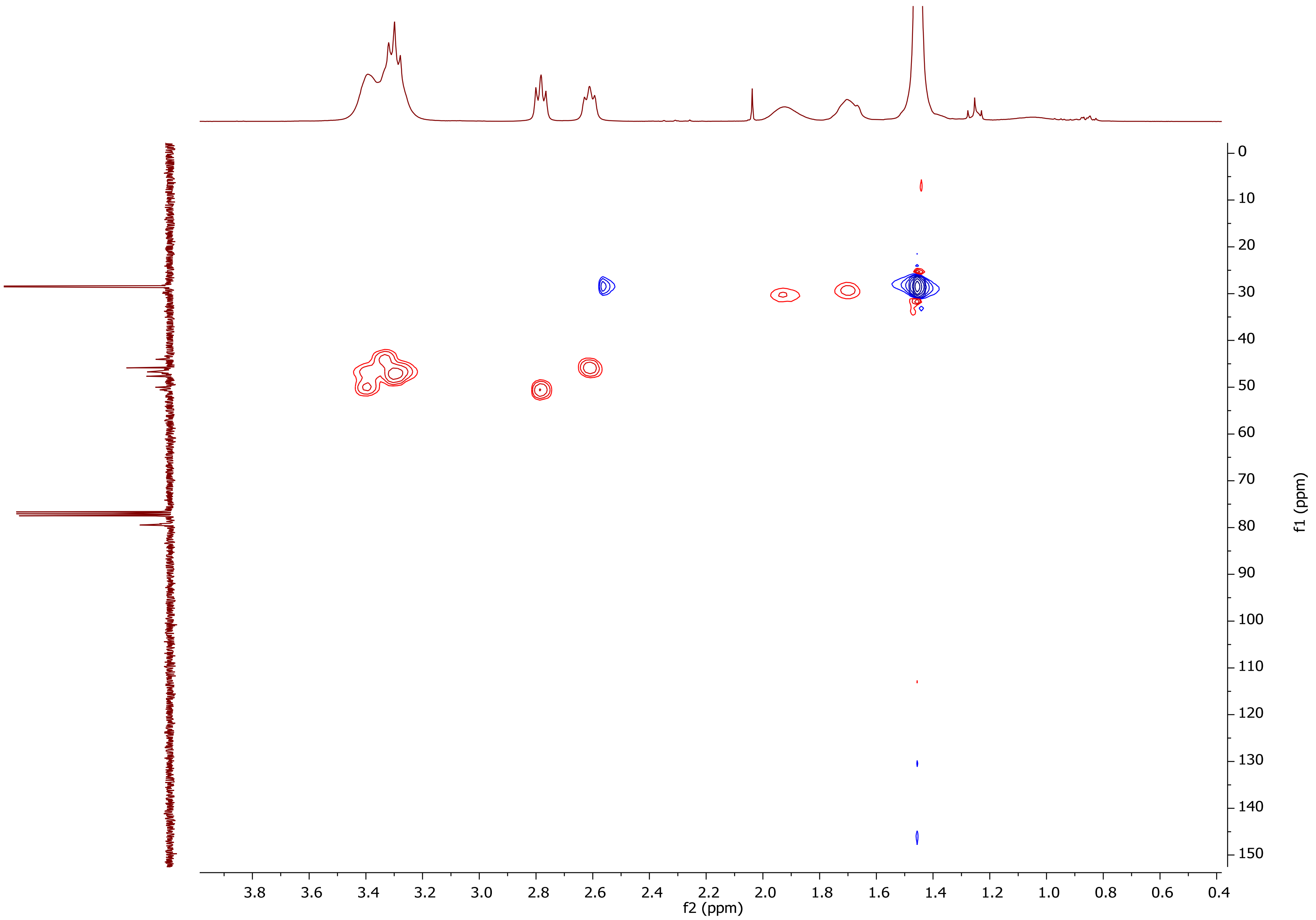

^1H-^1H COSY, $CDCl_3$, 298K

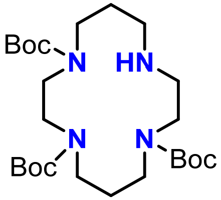

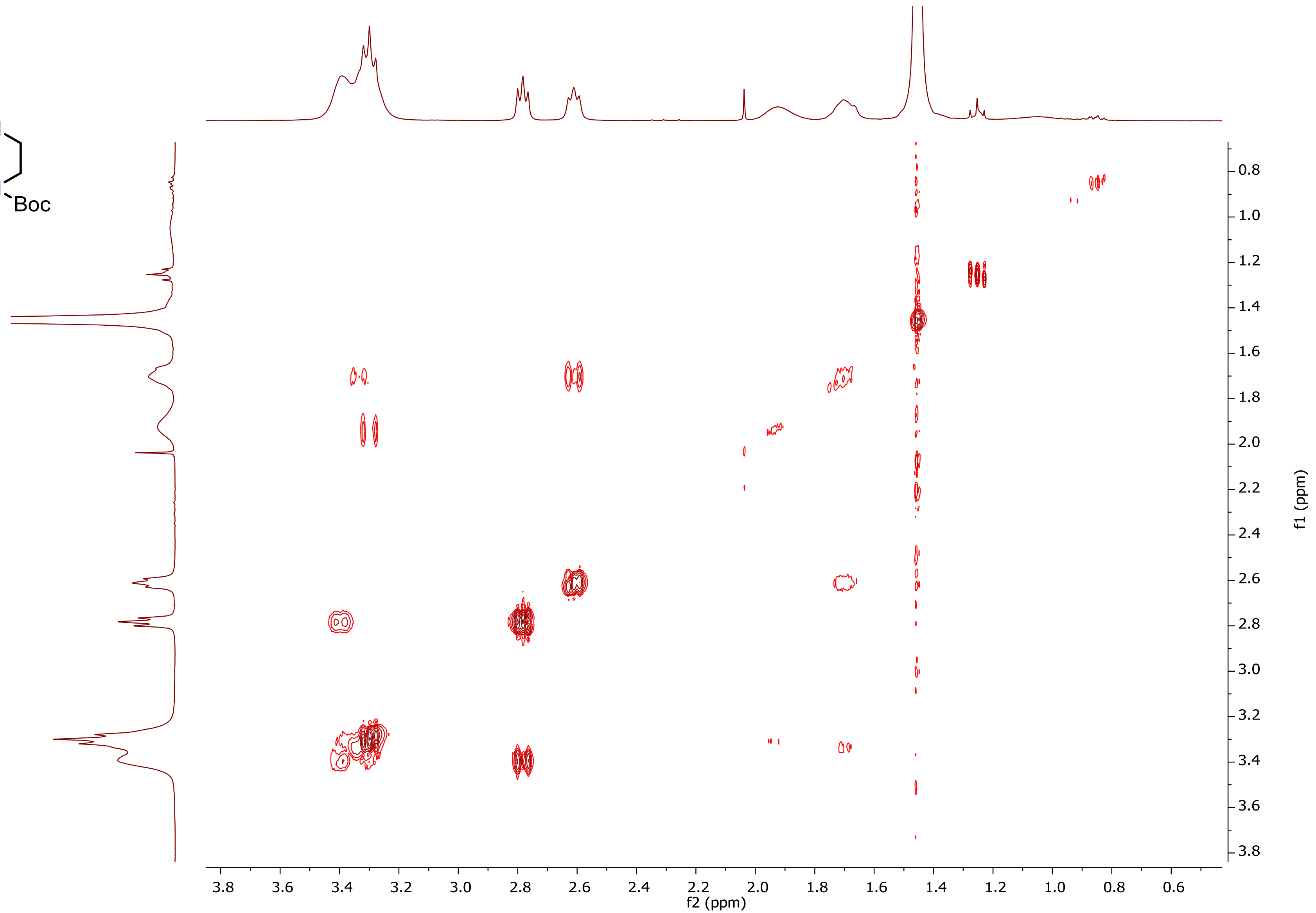

^{1}H NMR, $CDCl_3$, 298K

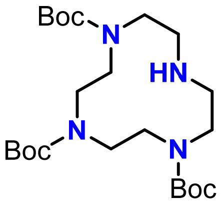

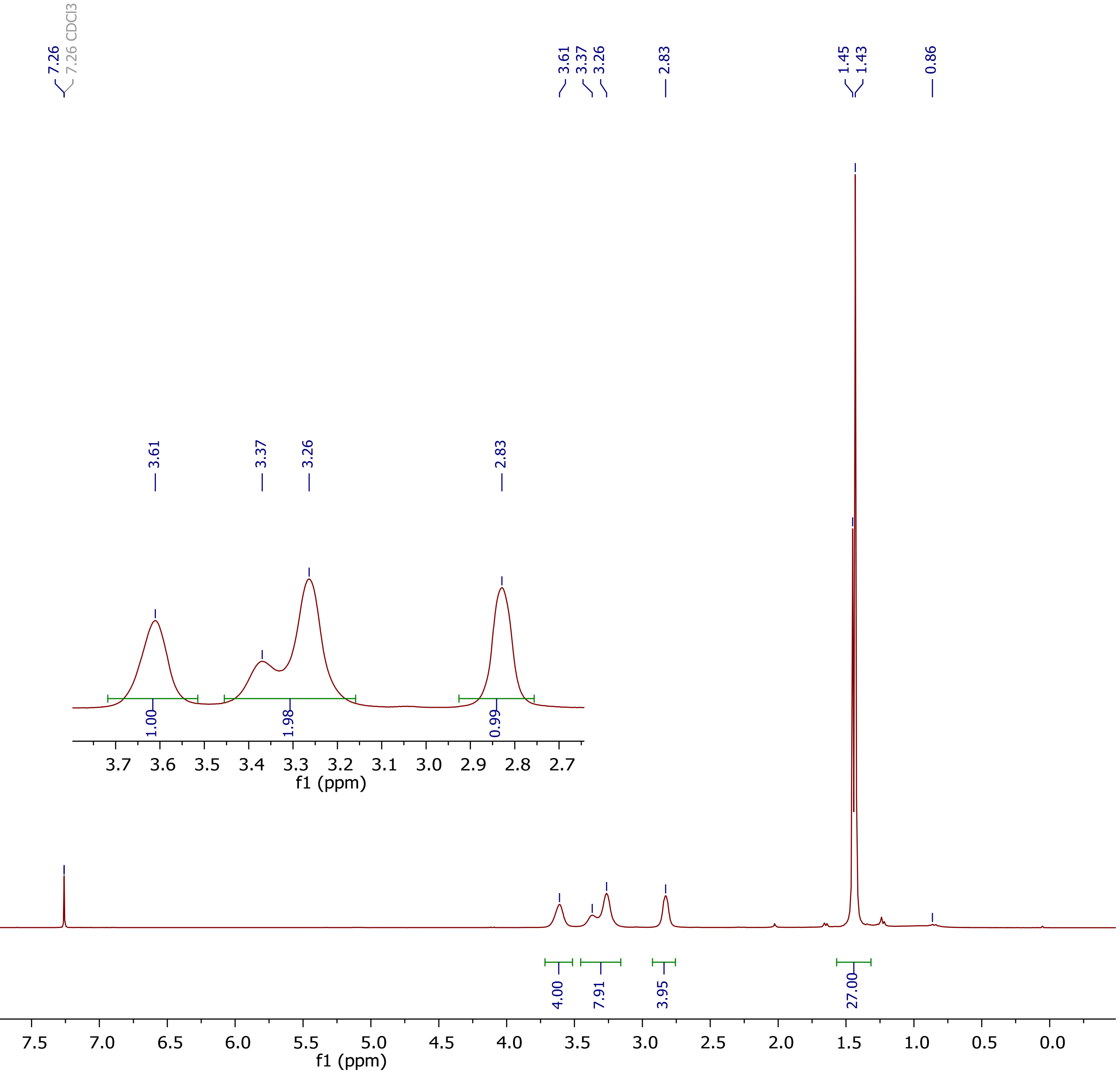

^{13}C NMR, $CDCl_3$, 298K

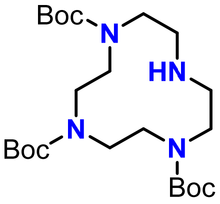

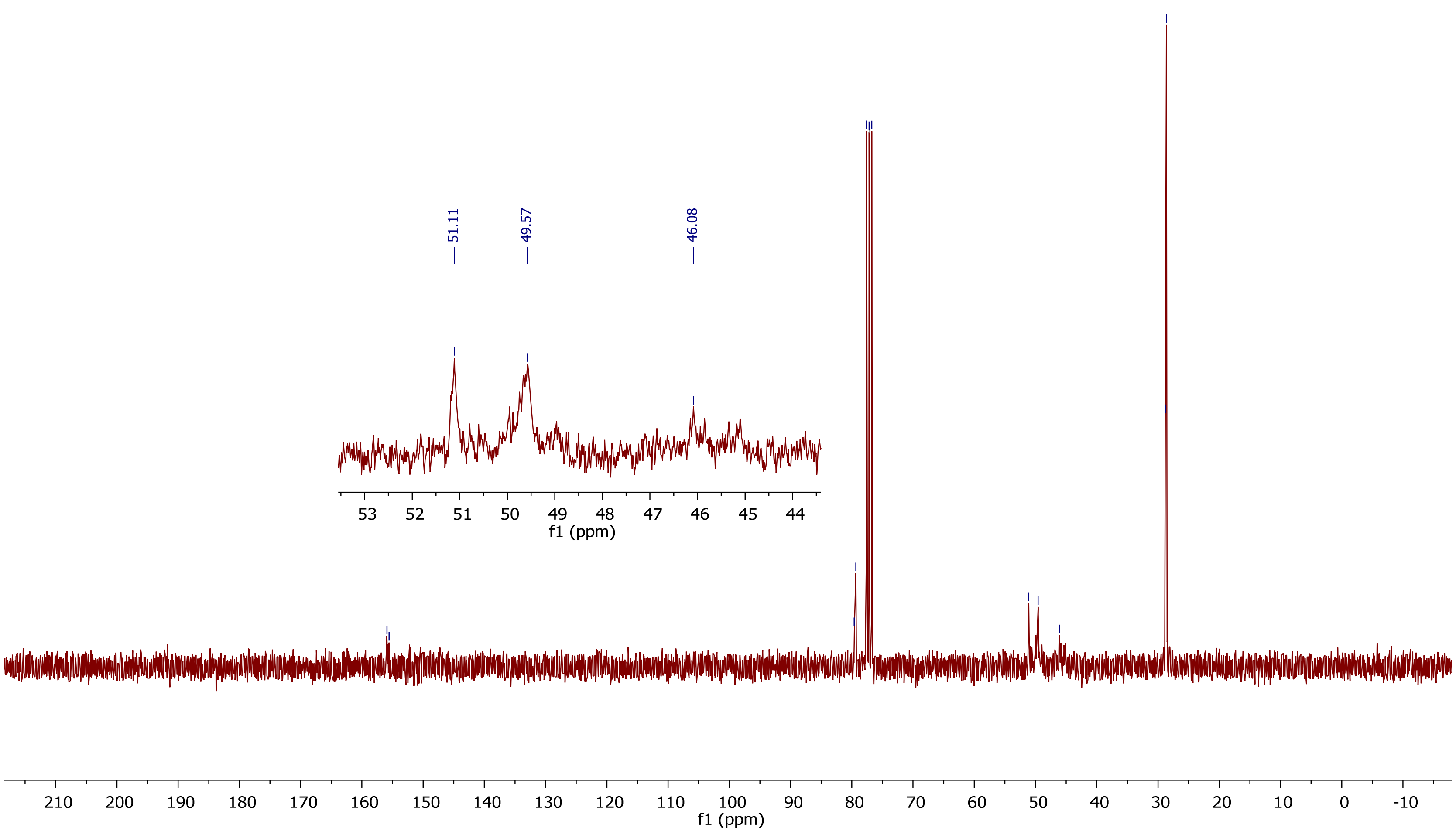

^{1}H-^{13}C HSQC, $CDCl_3$, 298K

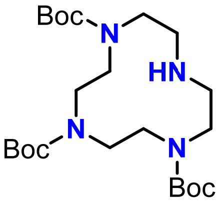

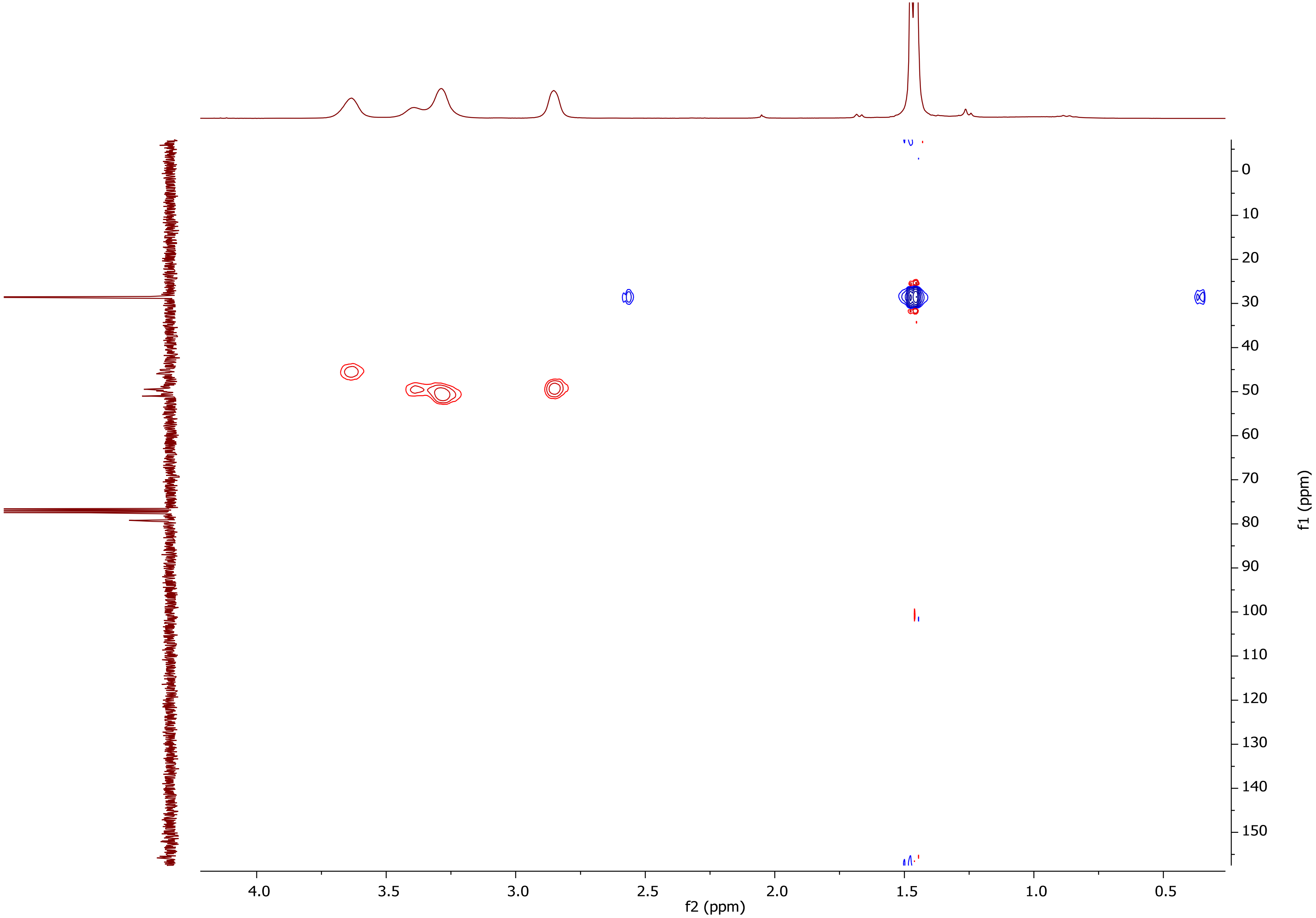

^{1}H-^{1}H COSY, $CDCl_3$, 298K

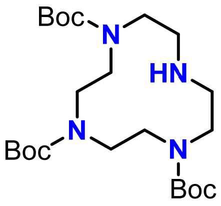

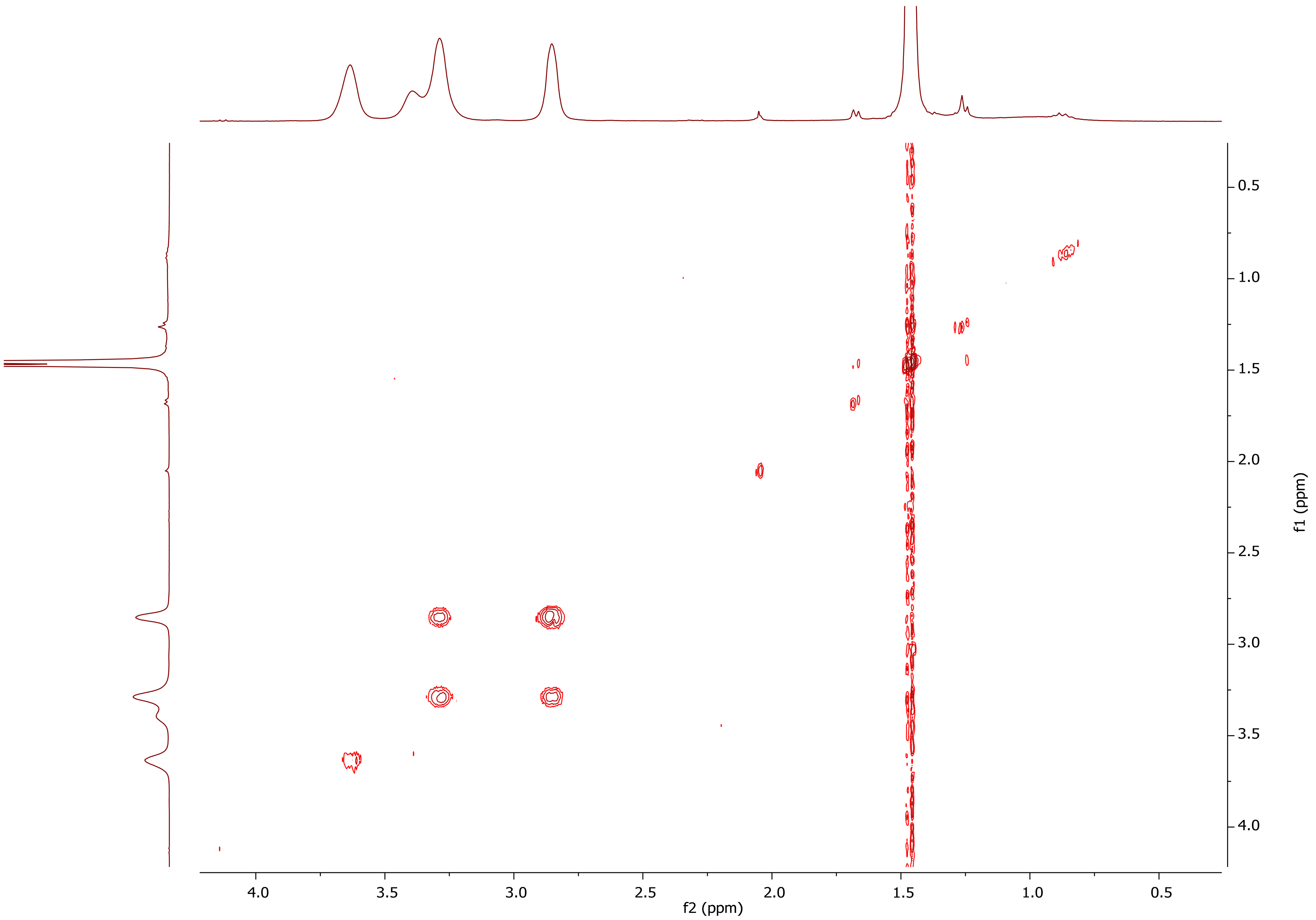

^{1}H NMR, $CDCl_3$, 298K

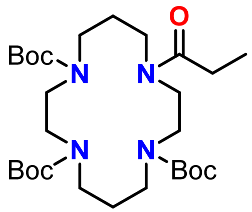

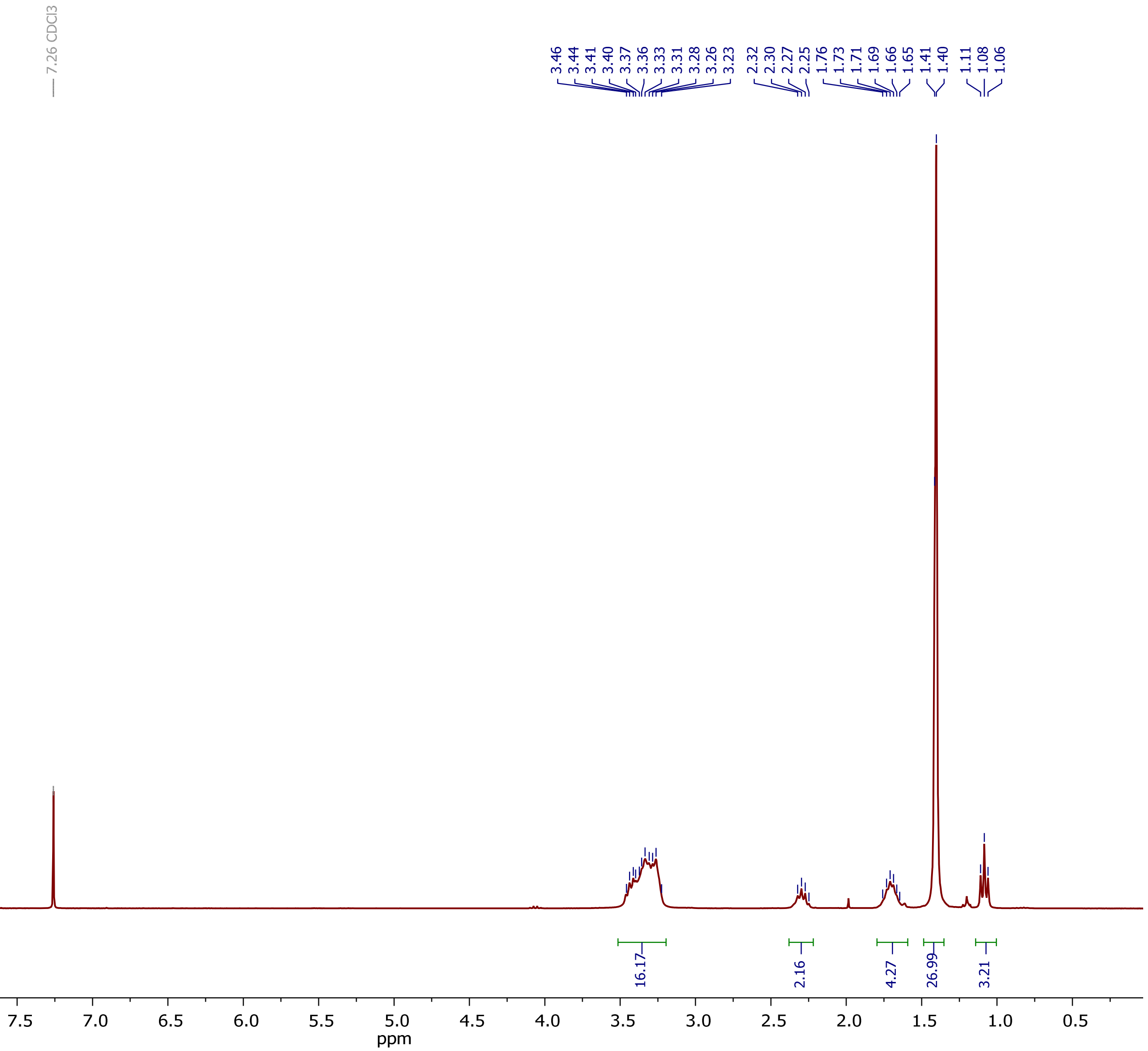

^{13}C NMR, $CDCl_3$, 298K

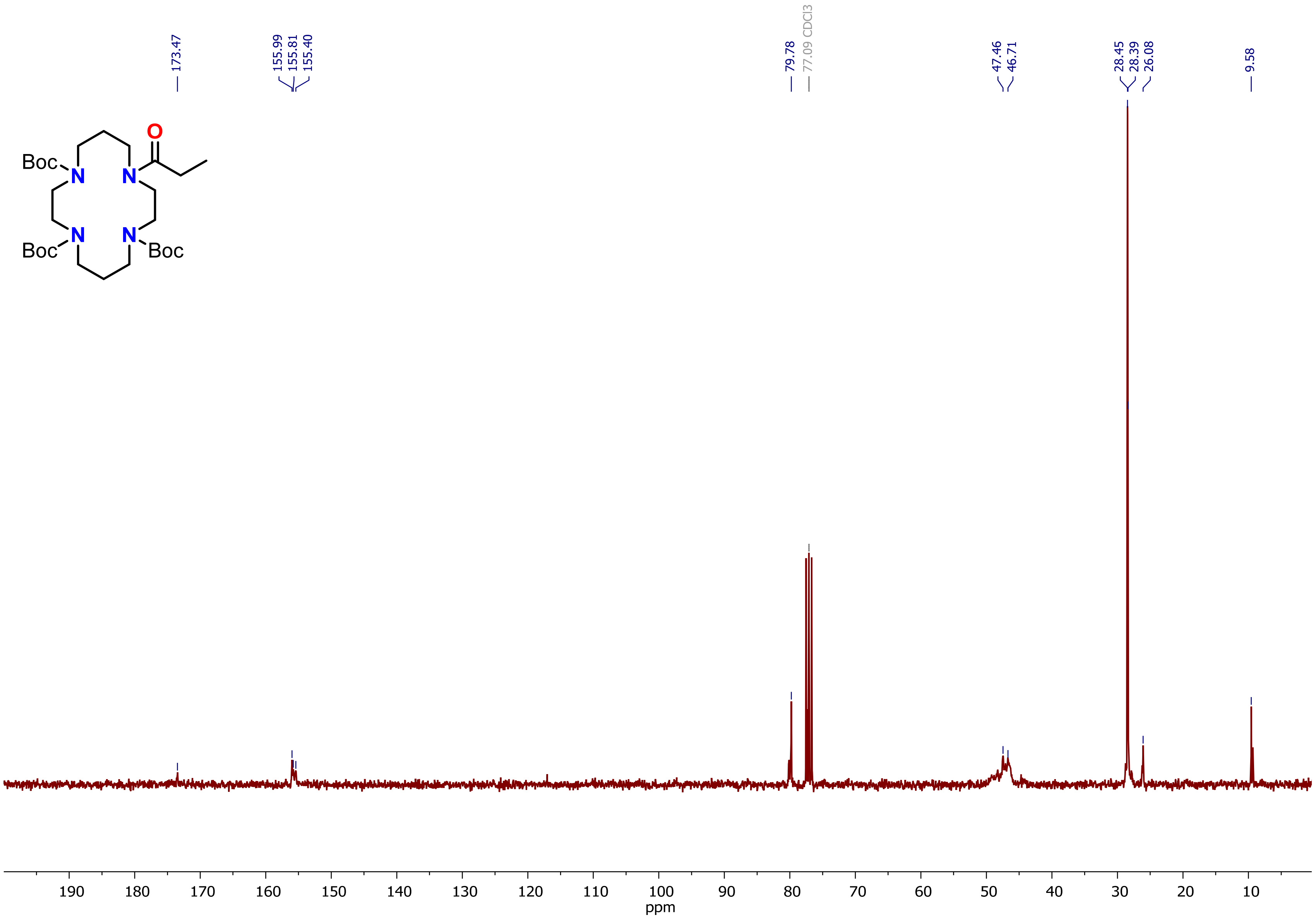

^{1}H-^{13}C HSQC, $CDCl_3$, 298K

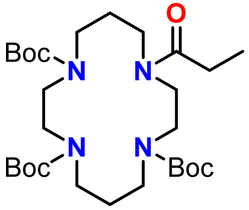

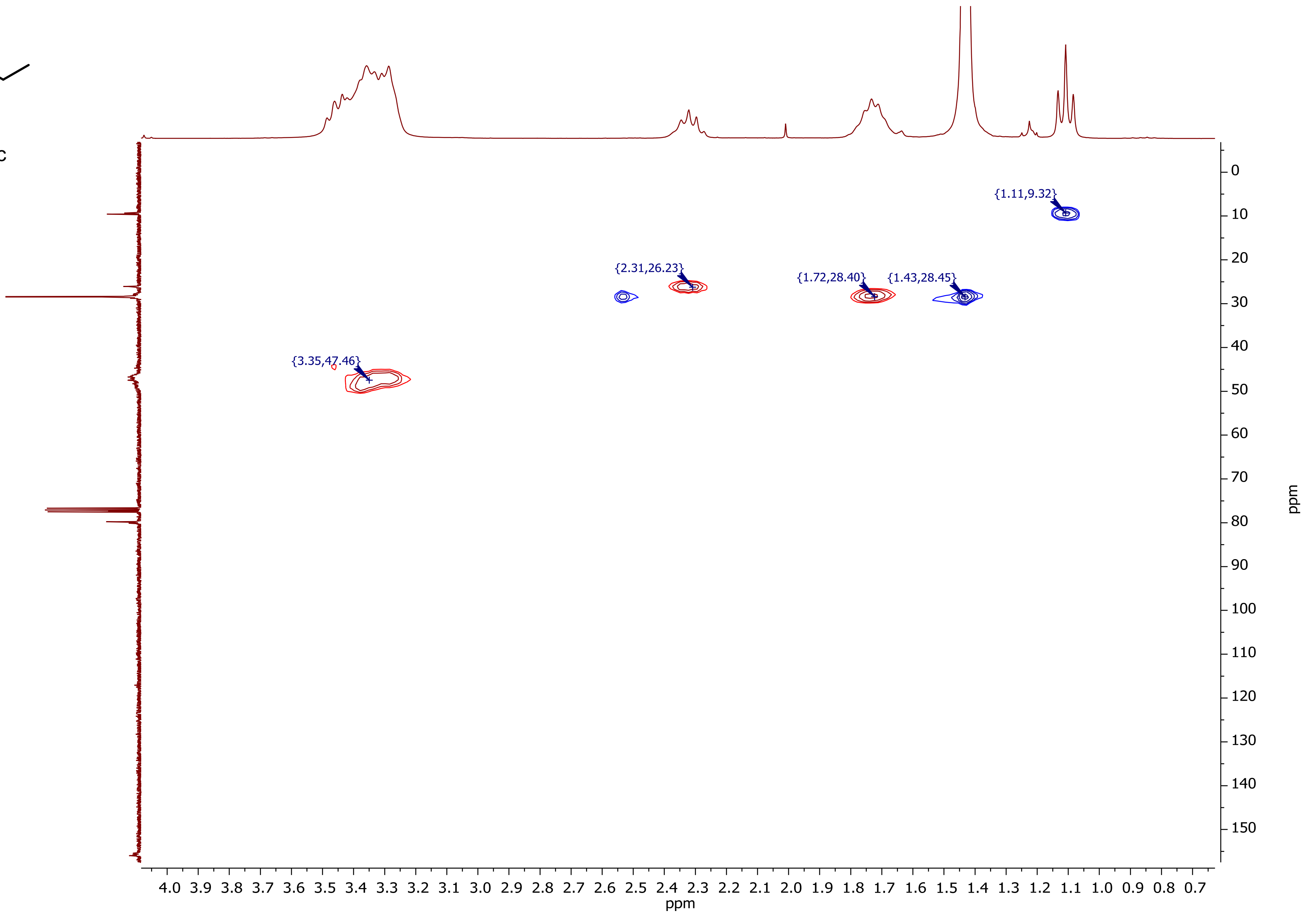

^{1}H NMR, $CDCl_3$, 298K

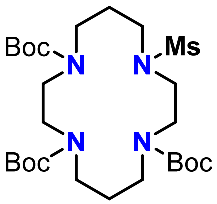

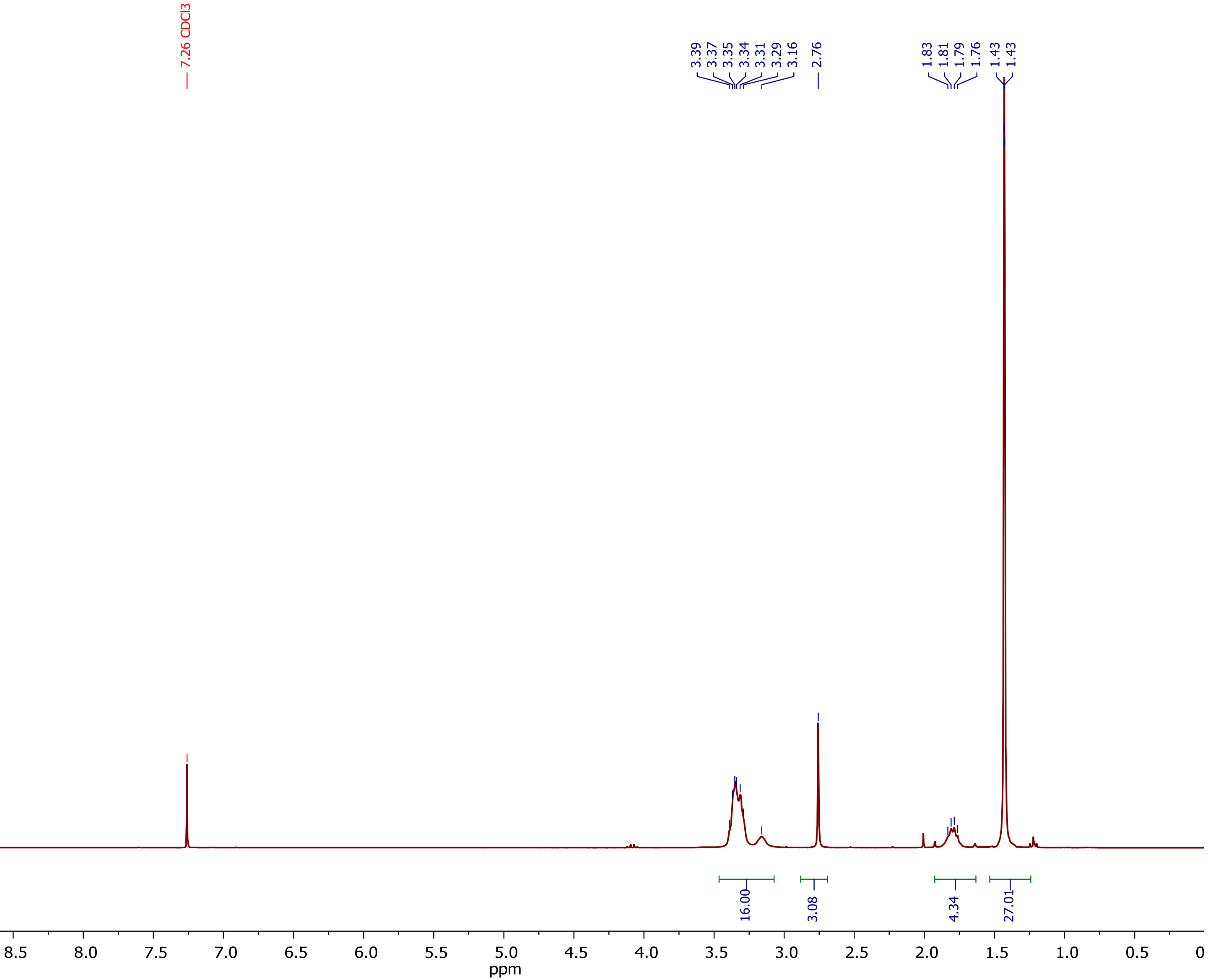

^{13}C NMR, $CDCl_3$, 298K

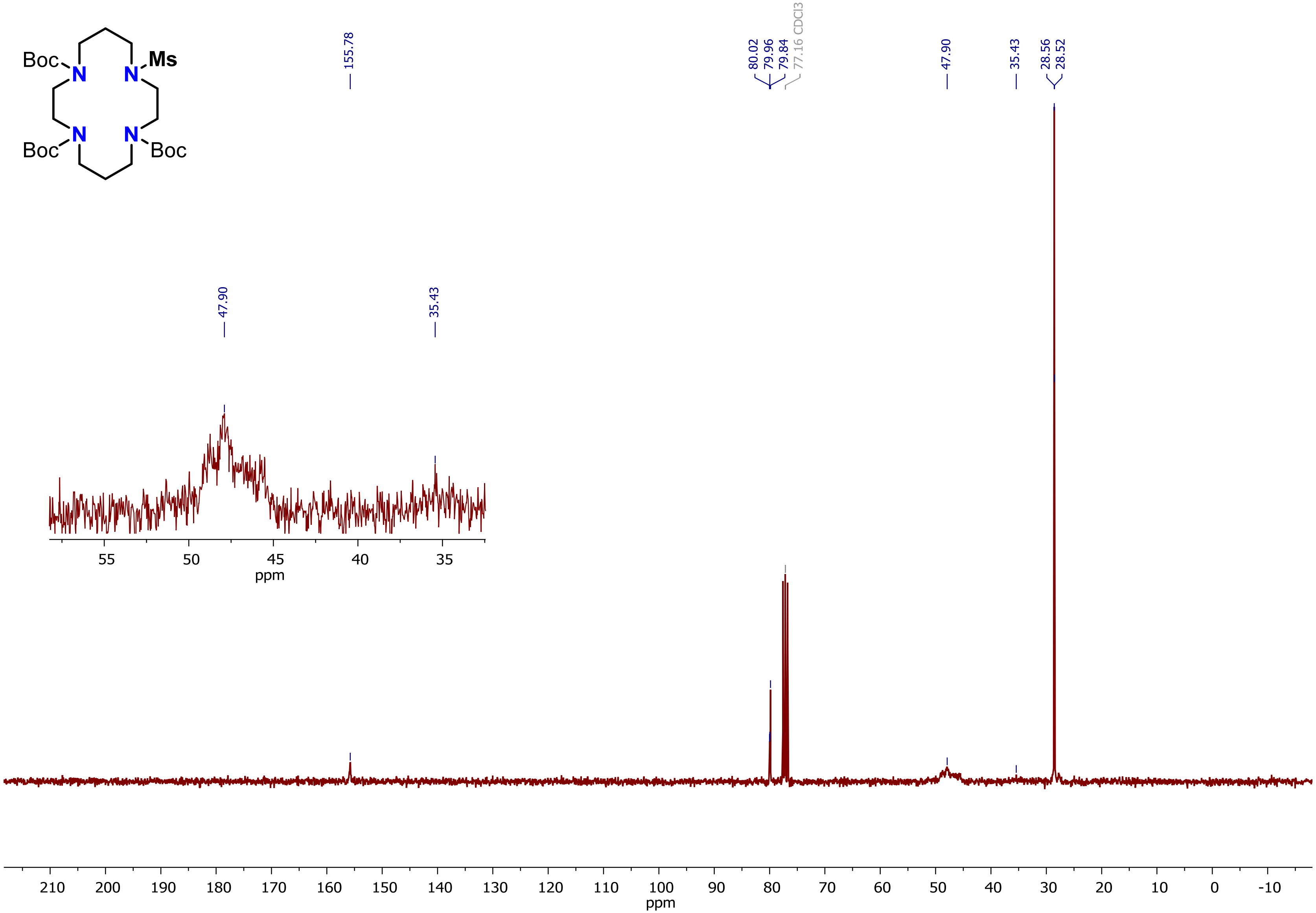

$^1H-^{13}C$ HSQC, $CDCl_3$, 298K

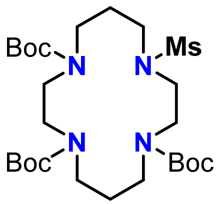

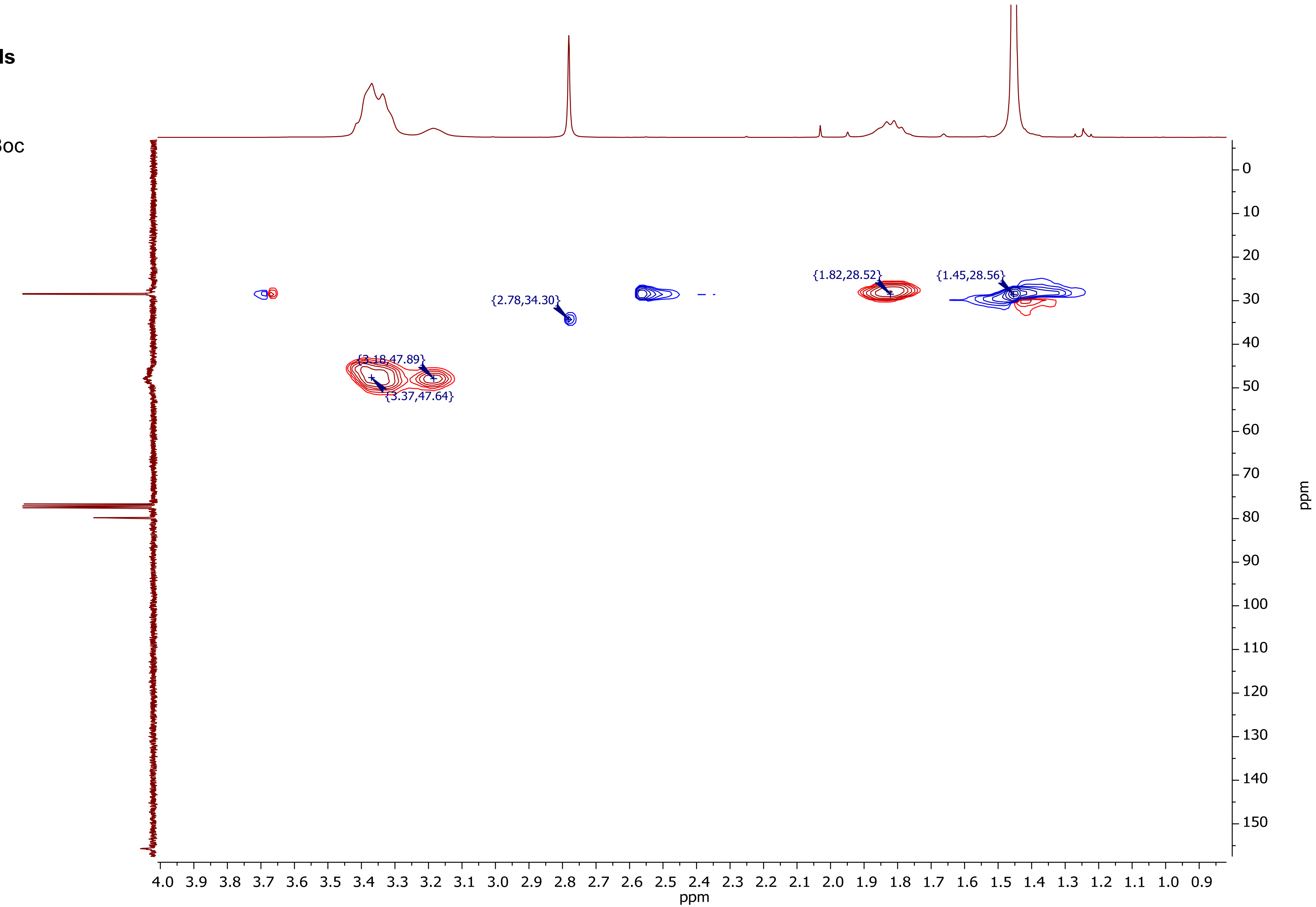

^{1}H NMR, CDCl$_3$, 298K

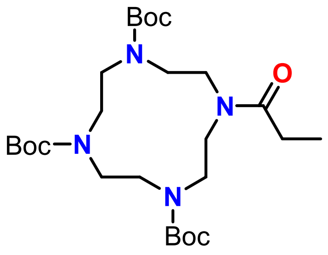

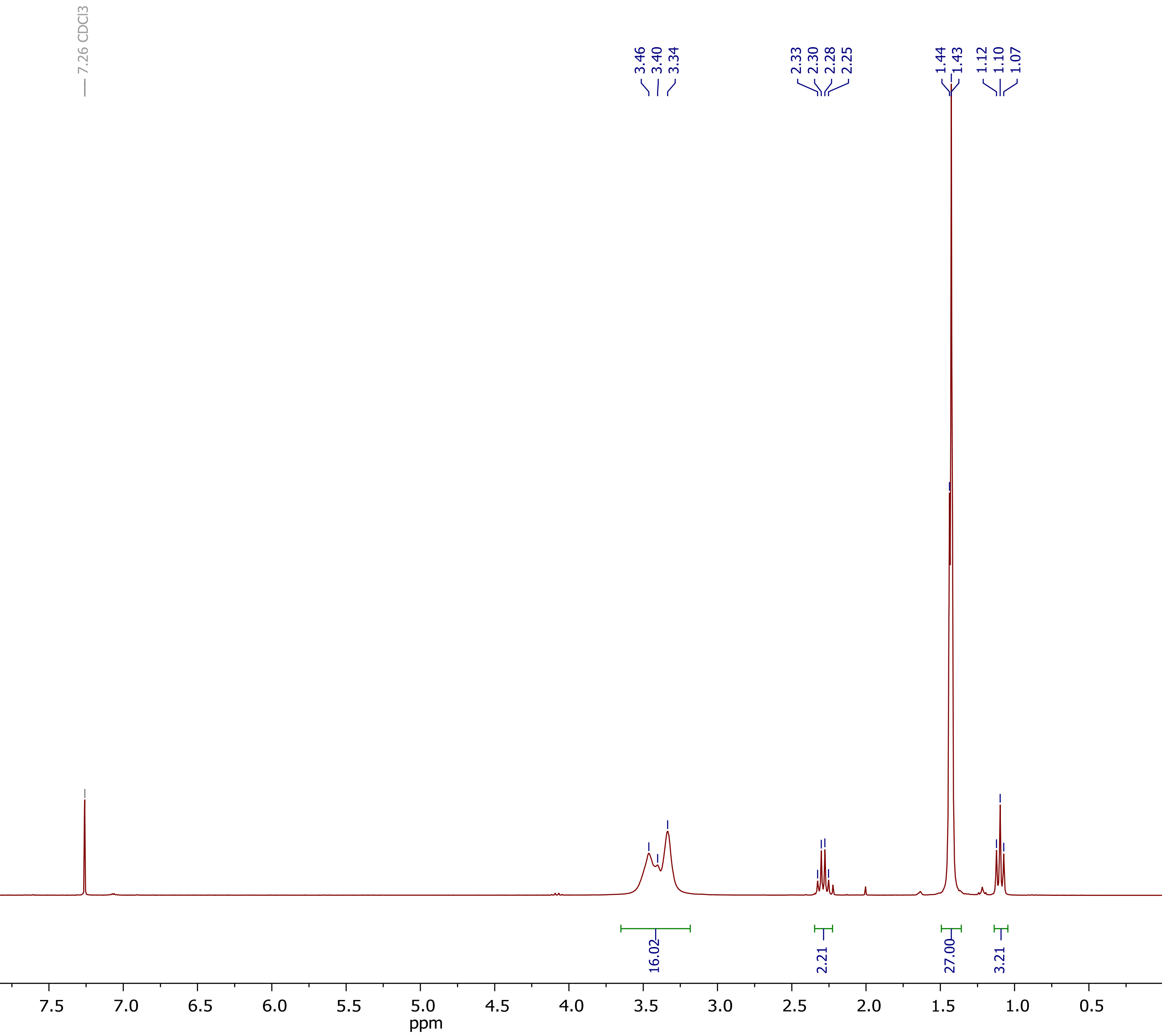

^{13}C NMR, $CDCl_3$, 298K

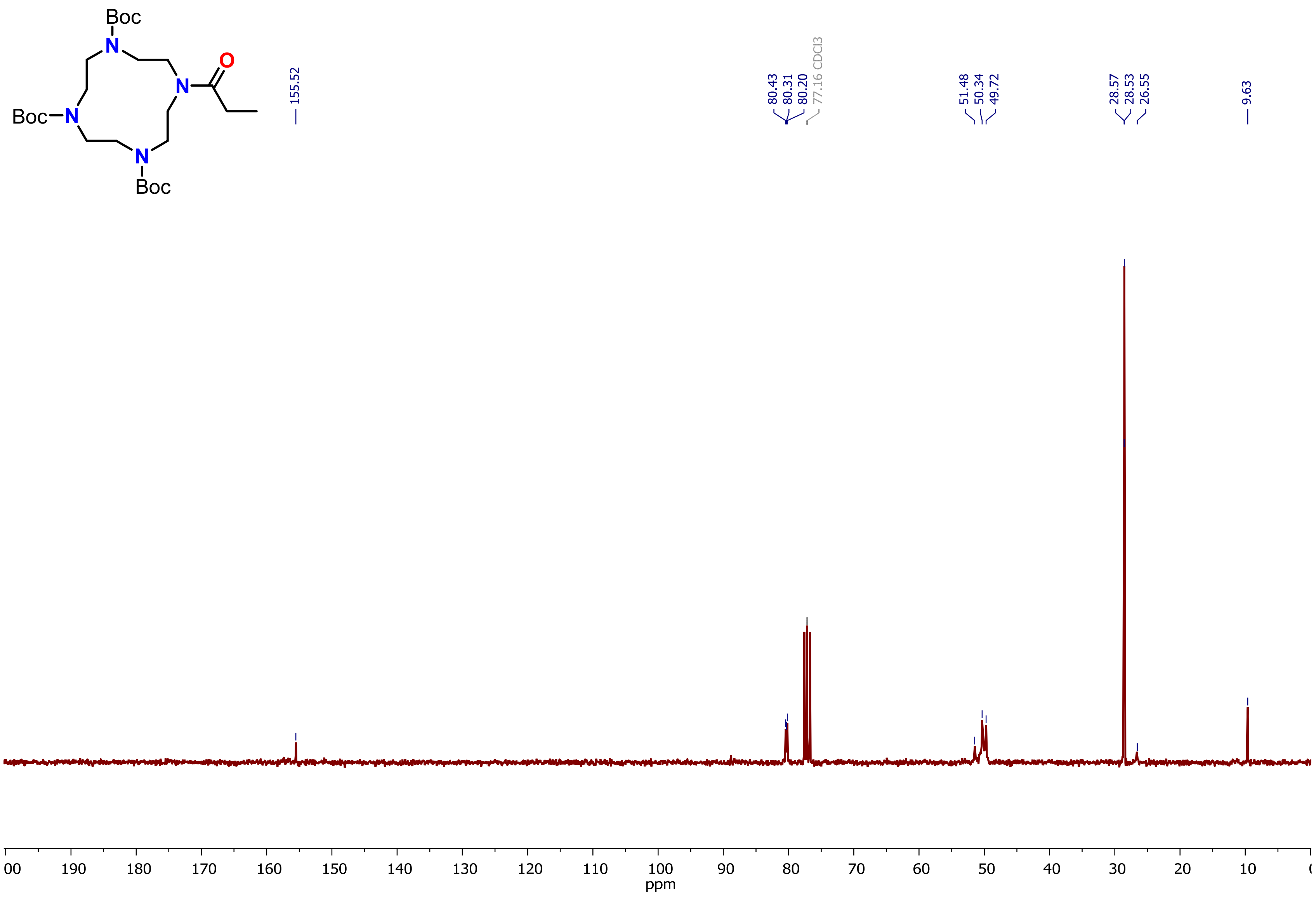

^{1}H-^{13}C HSQC, $CDCl_3$, 298K

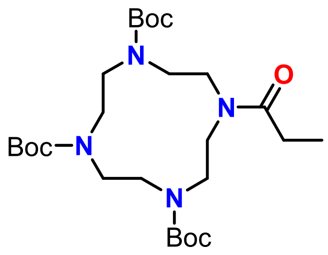

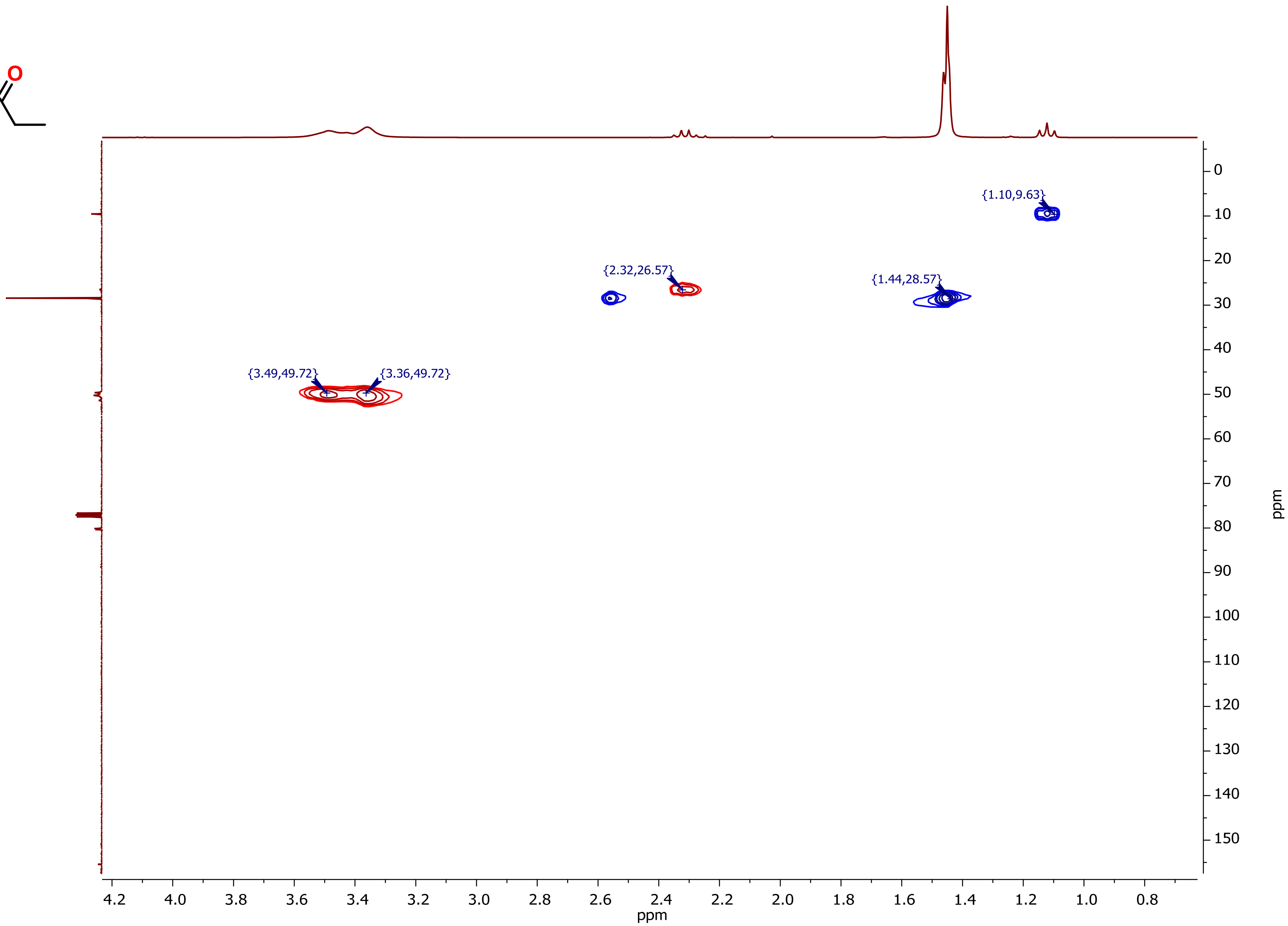

^{1}H NMR, D_2O, 298K

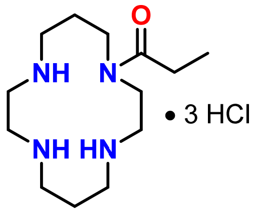

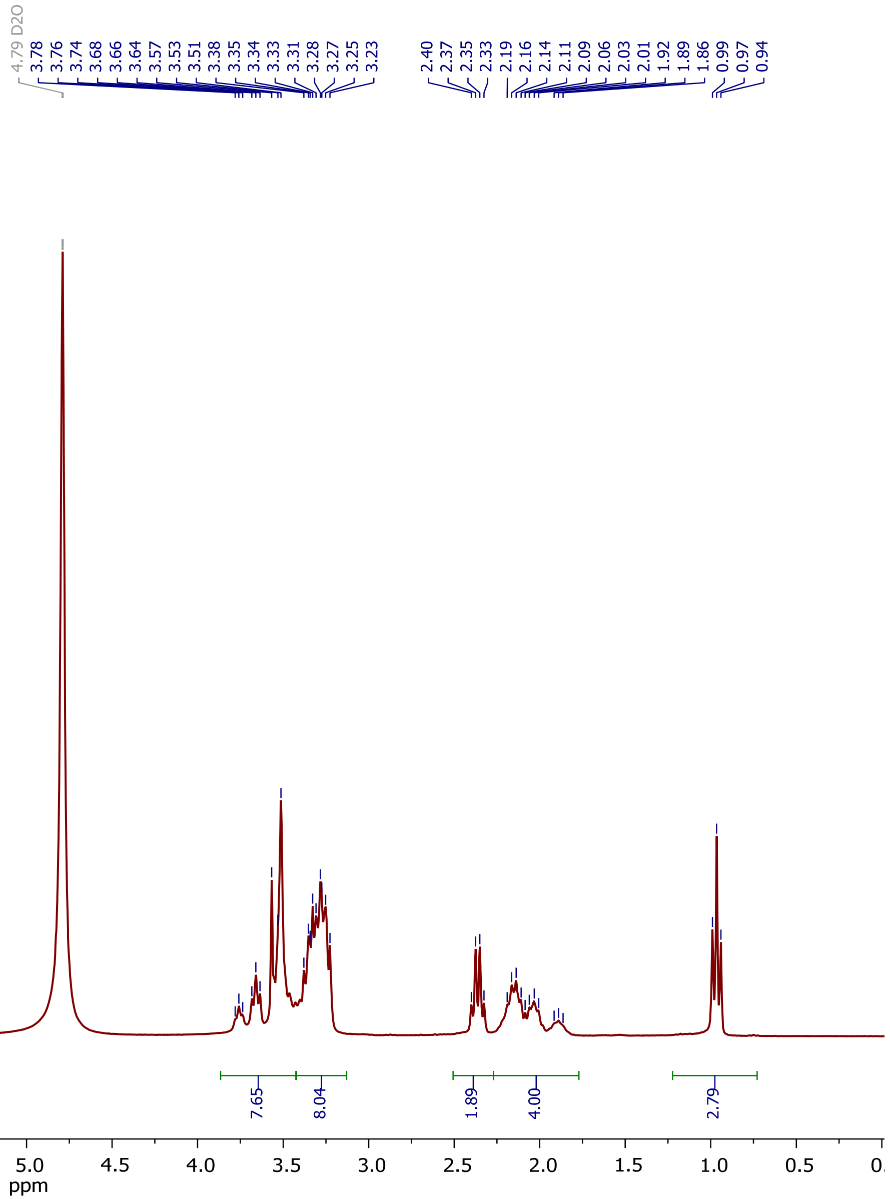

^{13}C NMR, D_2O, 298K

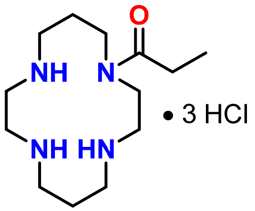

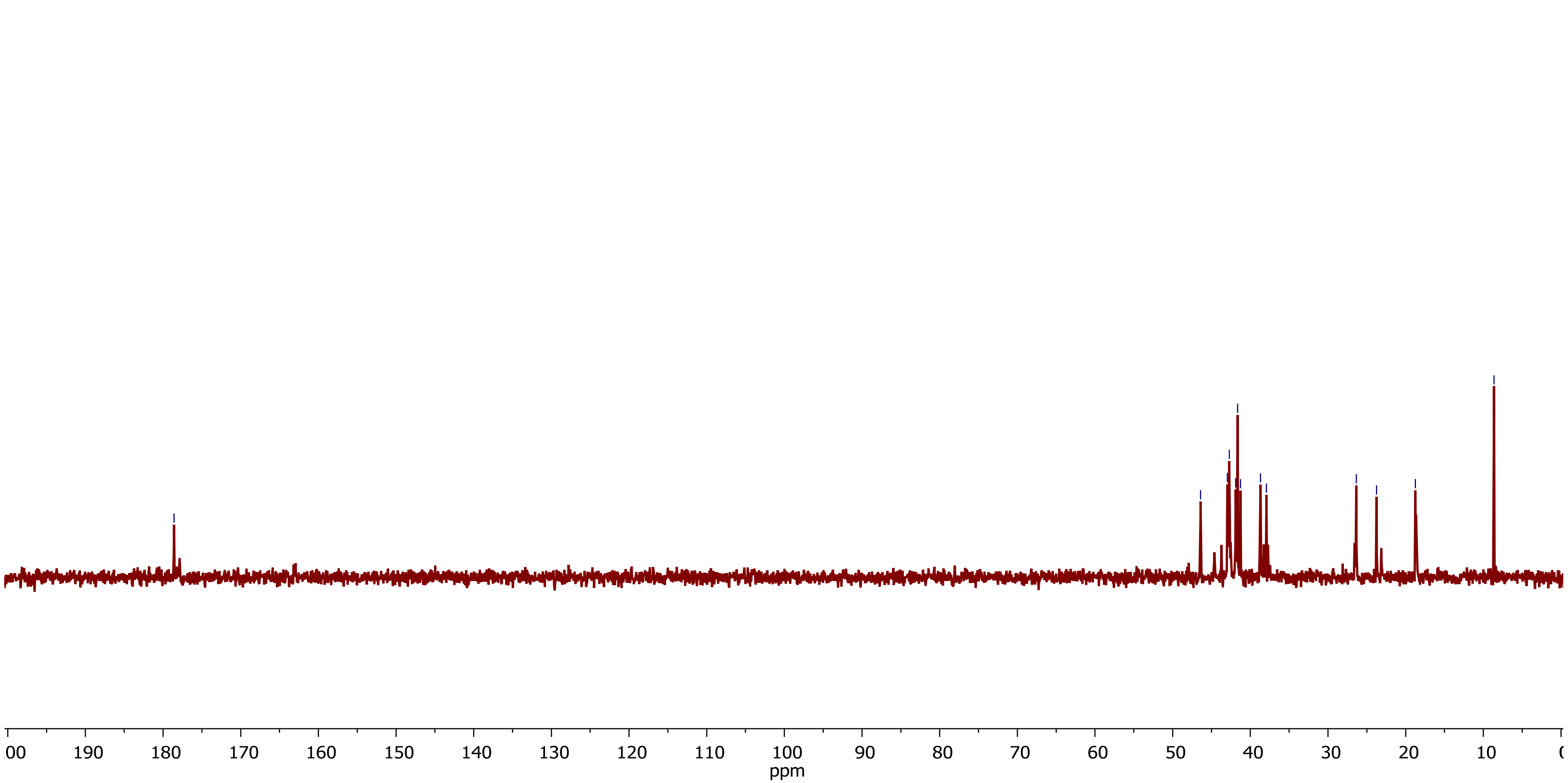

^{1}H-^{13}C HSQC, D_2O, 298K

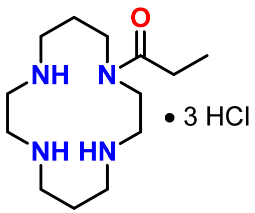

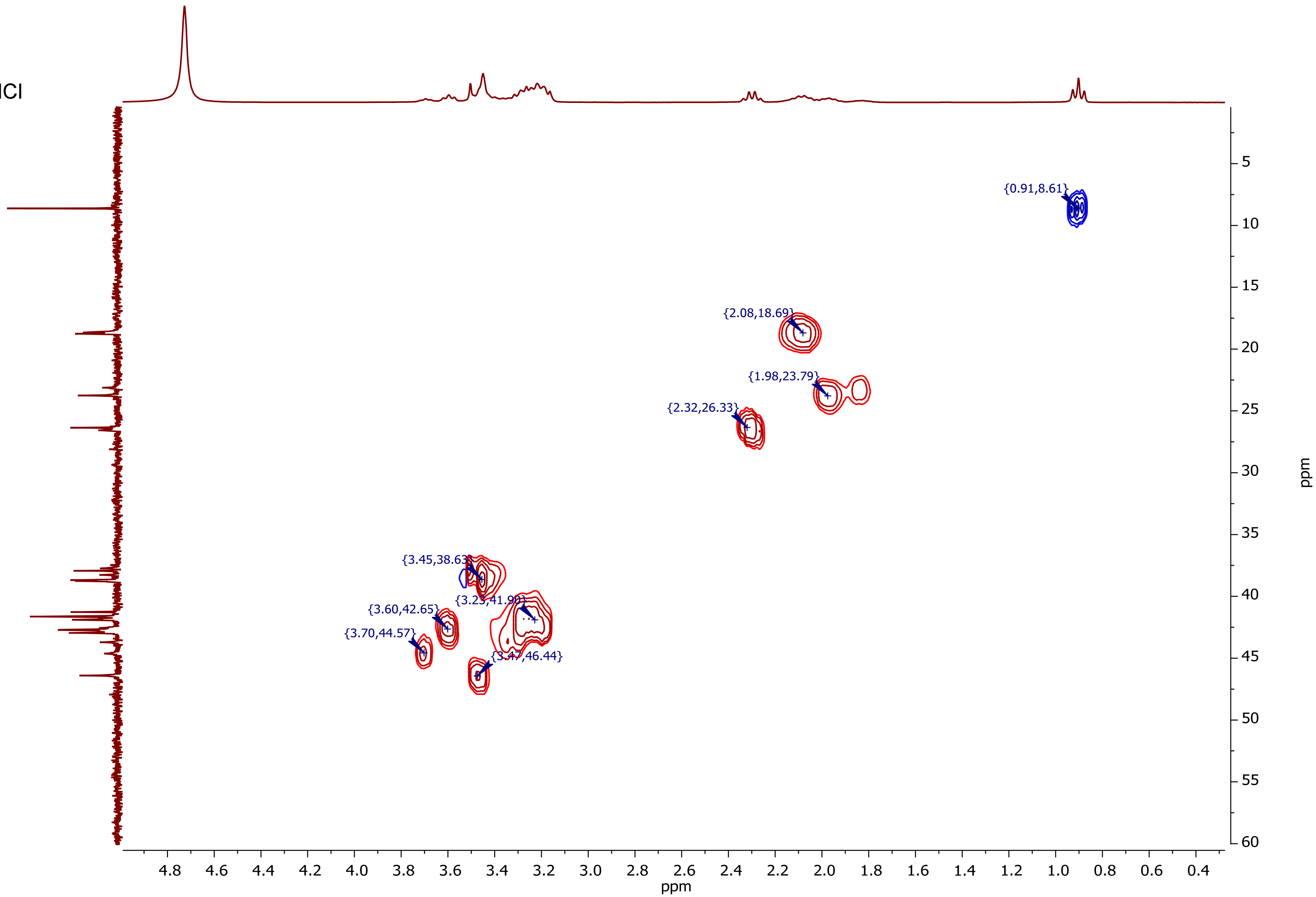

FT-IR, KBr

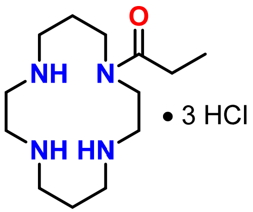
NH
N
O
NH
HN
• 3 HCl

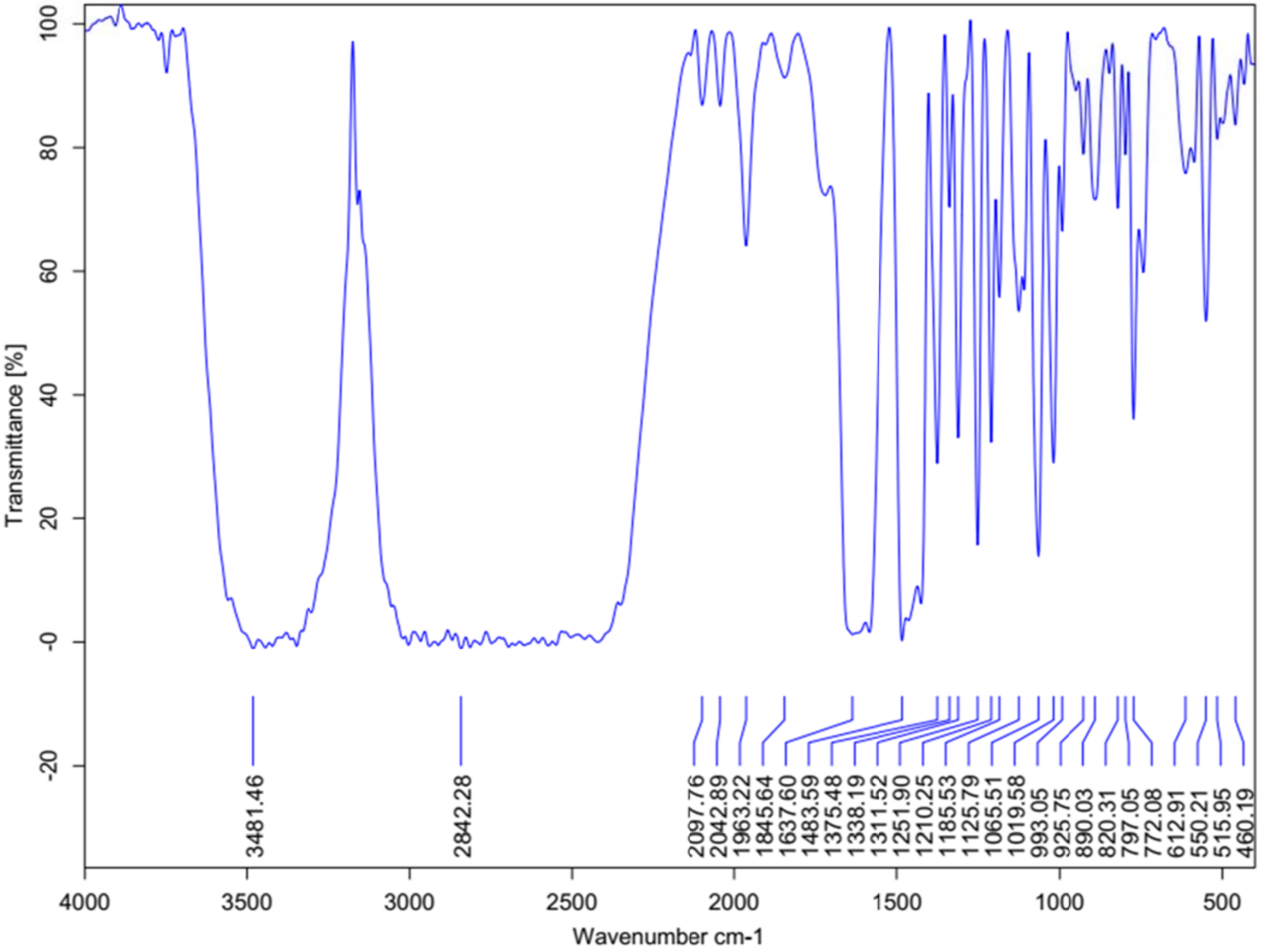
Transmittance [%]
100
80
60
40
20
0
-20
3481.46
2842.28
2097.76
2042.89
1963.22
1845.64
1637.60
1483.59
1375.48
1338.19
1311.52
1251.90
1210.25
1185.53
1125.79
1065.51
1019.58
993.05
925.75
890.03
820.31
797.05
772.08
612.91
550.21
515.95
460.19
4000
3500
3000
2500
2000
1500
1000
500
Wavenumber cm-1

^{1}H NMR, D_2O, 298K

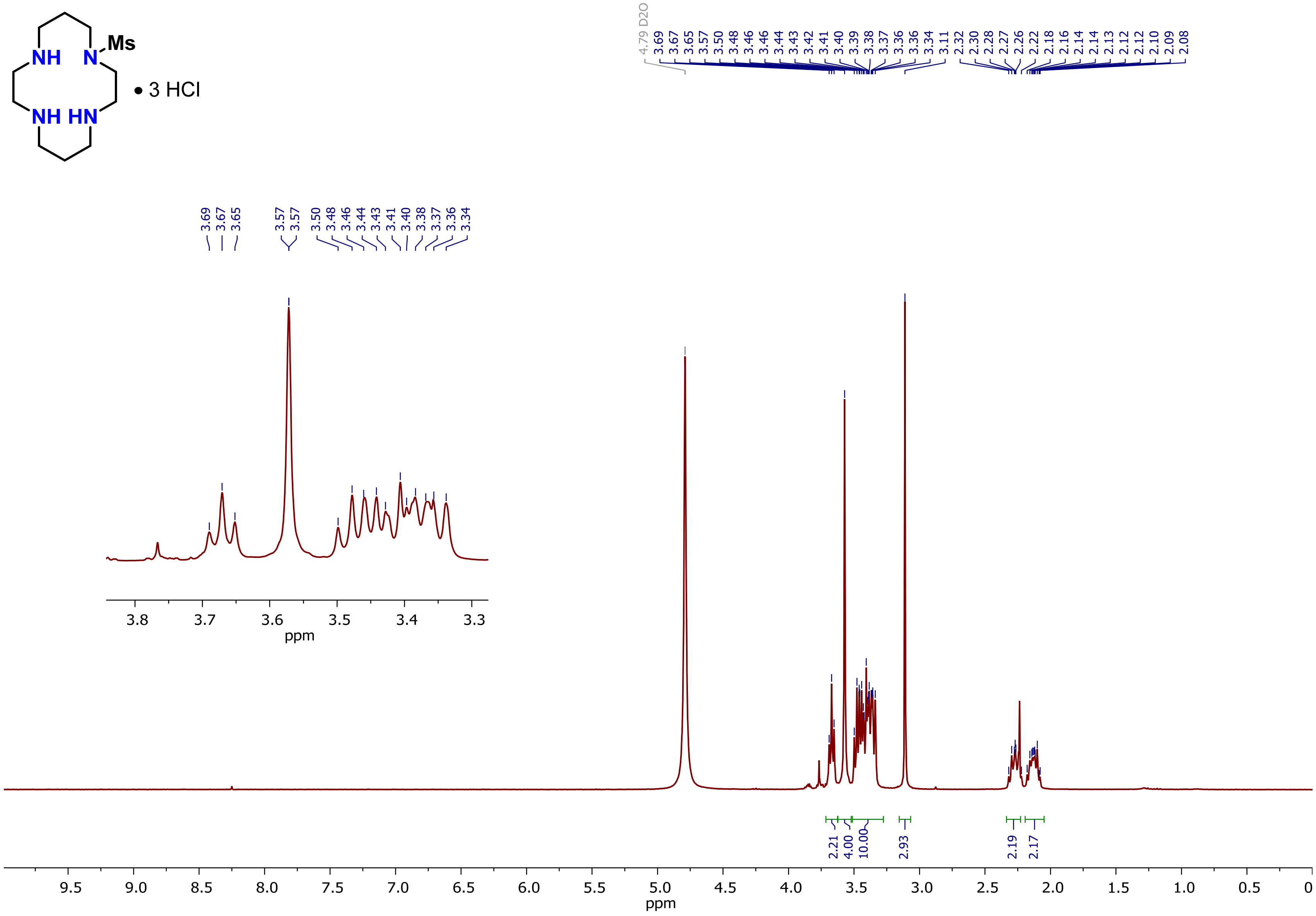

^{13}C NMR, D_2O, 298K

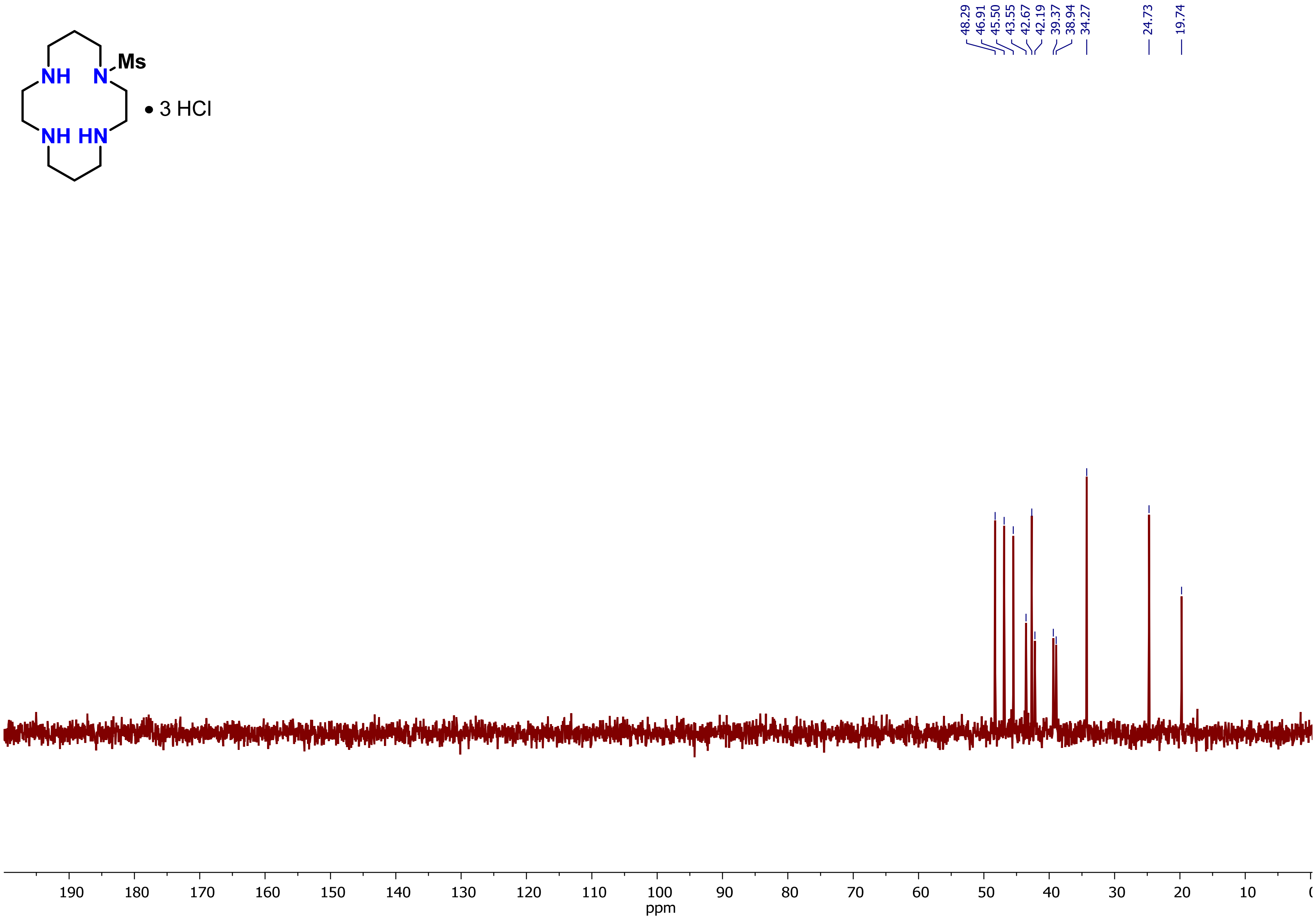

^{1}H-^{13}C HSQC, D_2O, 298K

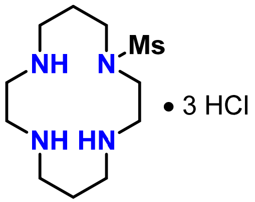

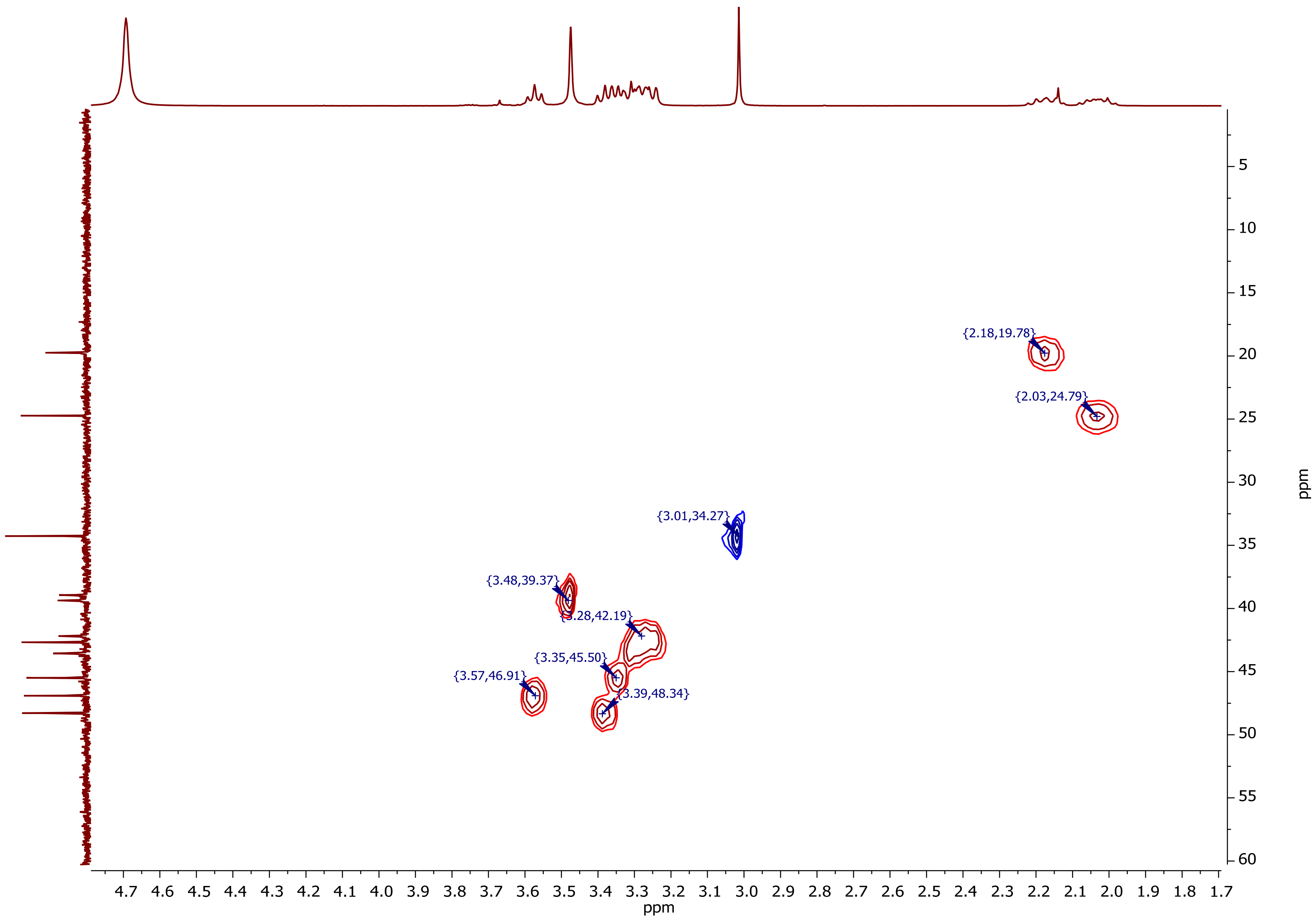

FT-IR, KBr

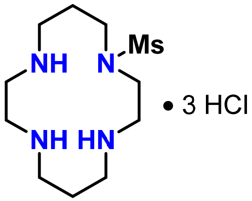

NH
N
Ms
NH
HN
• 3 HCl

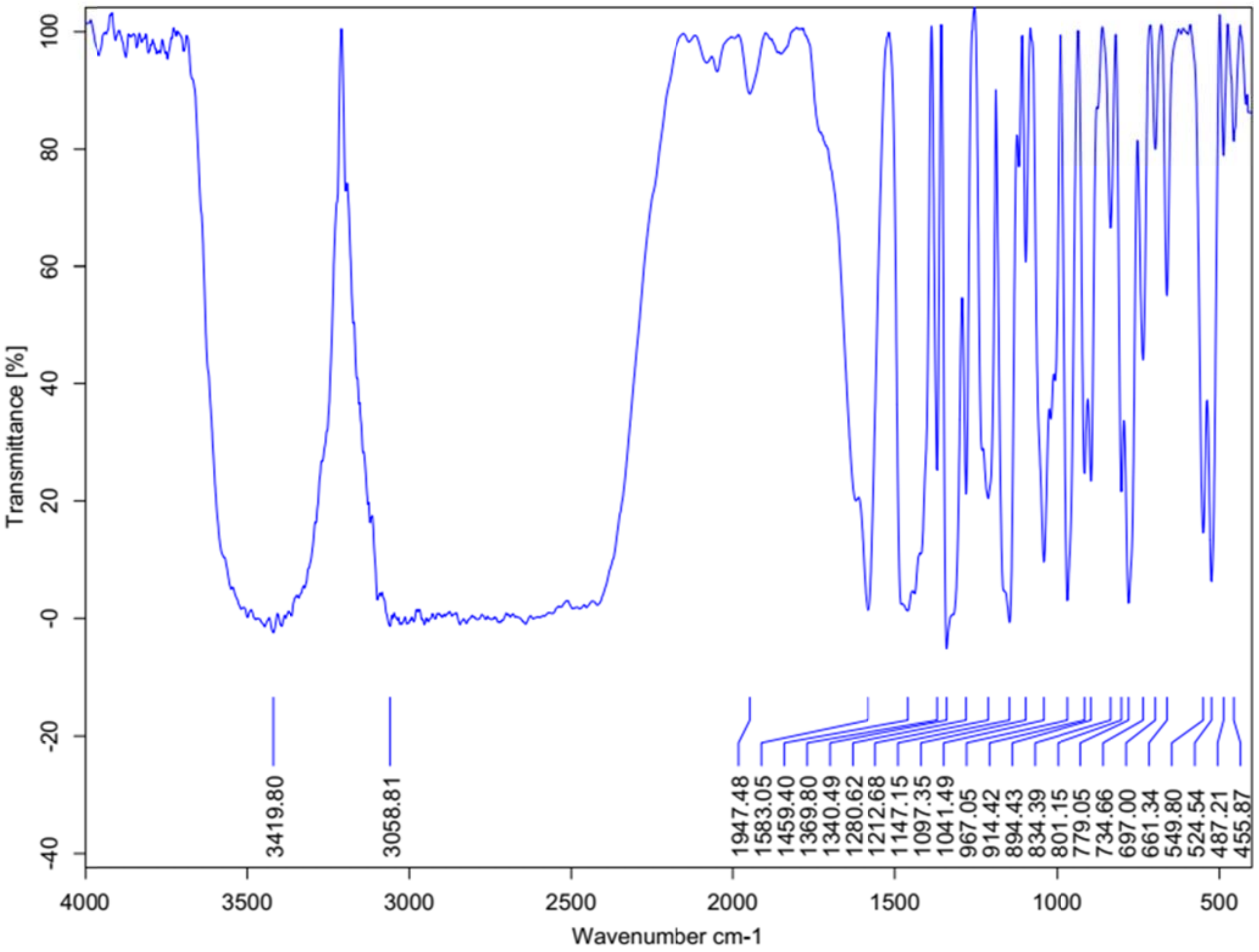

Transmittance [%]
Wavenumber cm-1
3419.80
3058.81
1947.48
1583.05
1459.40
1369.80
1340.49
1280.62
1212.68
1147.15
1097.35
1041.49
967.05
914.42
894.43
834.39
801.15
779.05
734.66
697.00
661.34
549.80
524.54
487.21
455.87

^{1}H NMR, D_2O, 298K

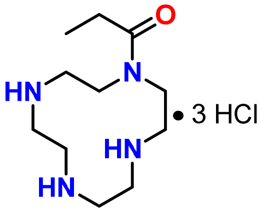

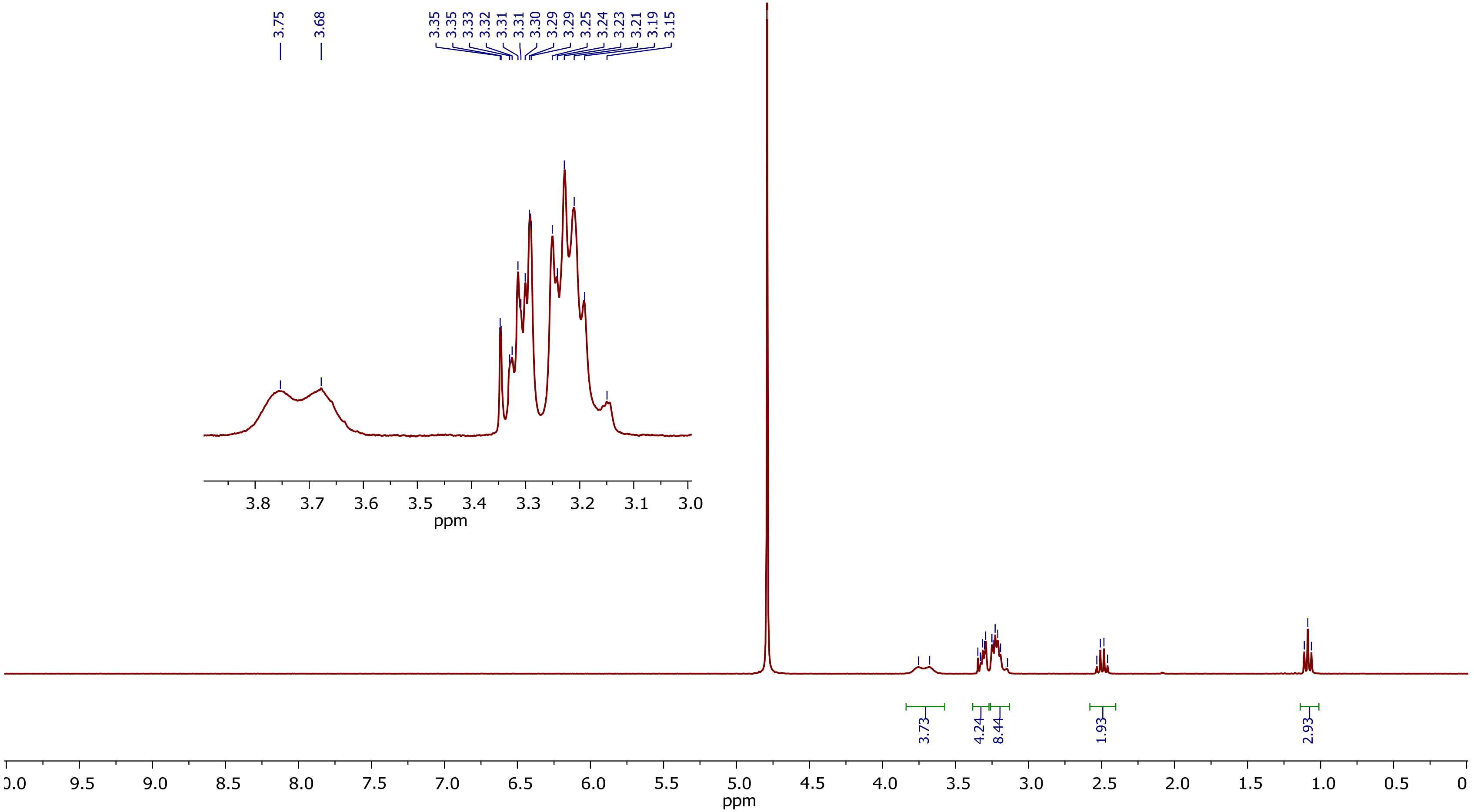

^{13}C NMR, D_2O, 298K

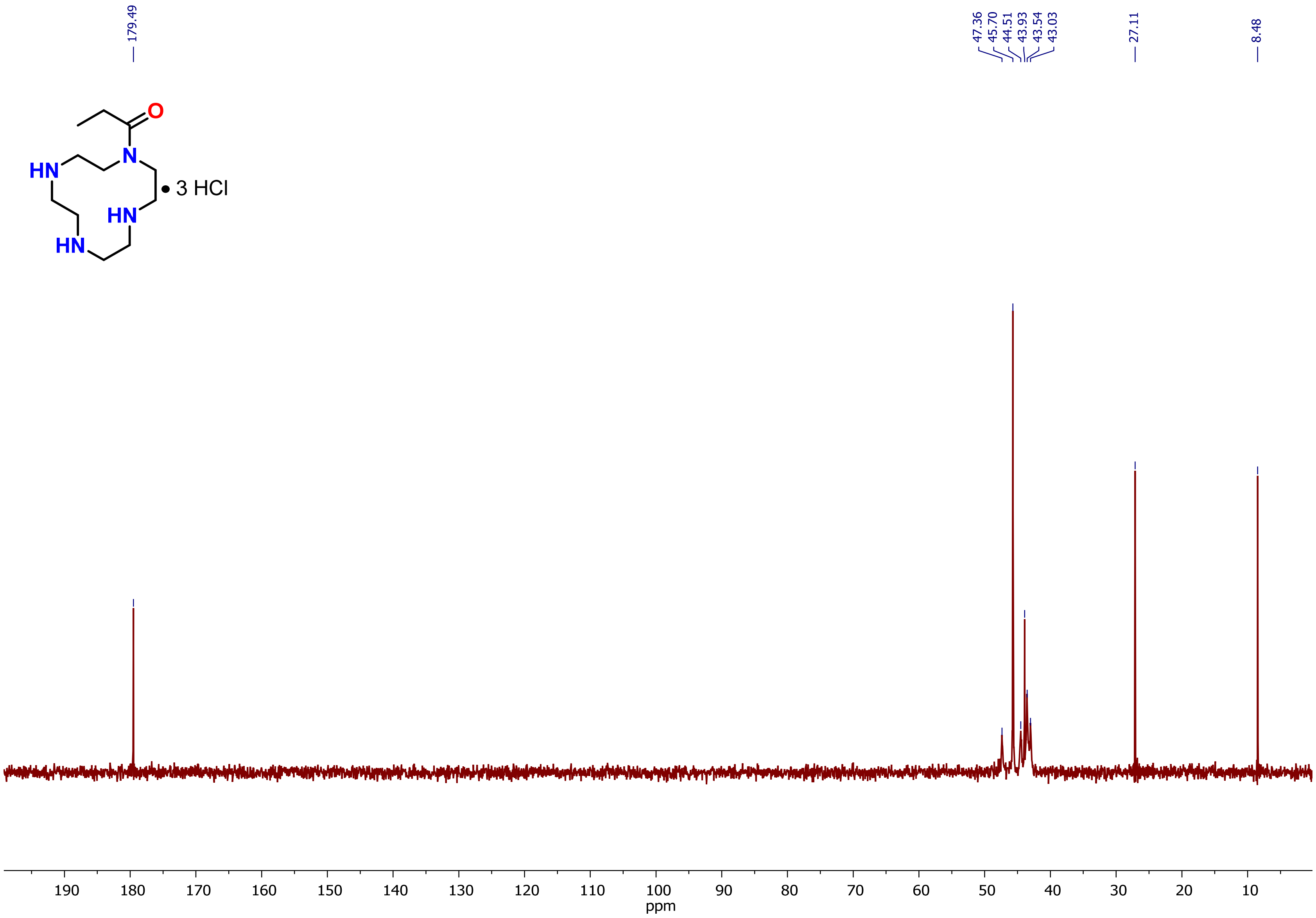

^1H-^{13}C HSQC, D_2O, 298K

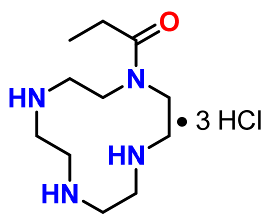

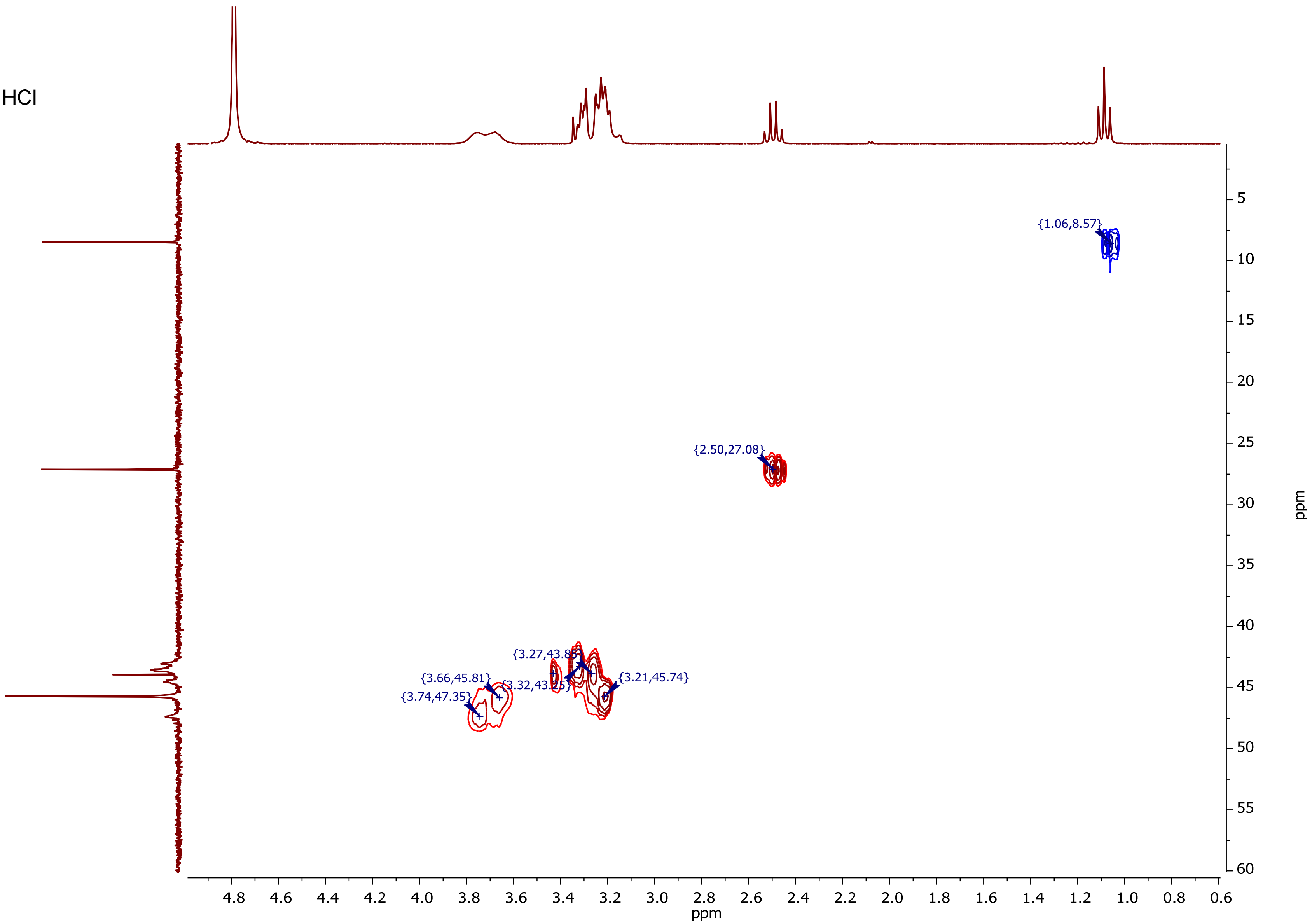

FT-IR, KBr

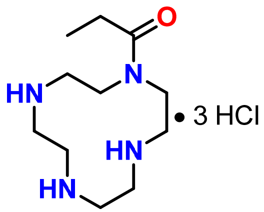

O
N
HN
HN
HN
• 3 HCl

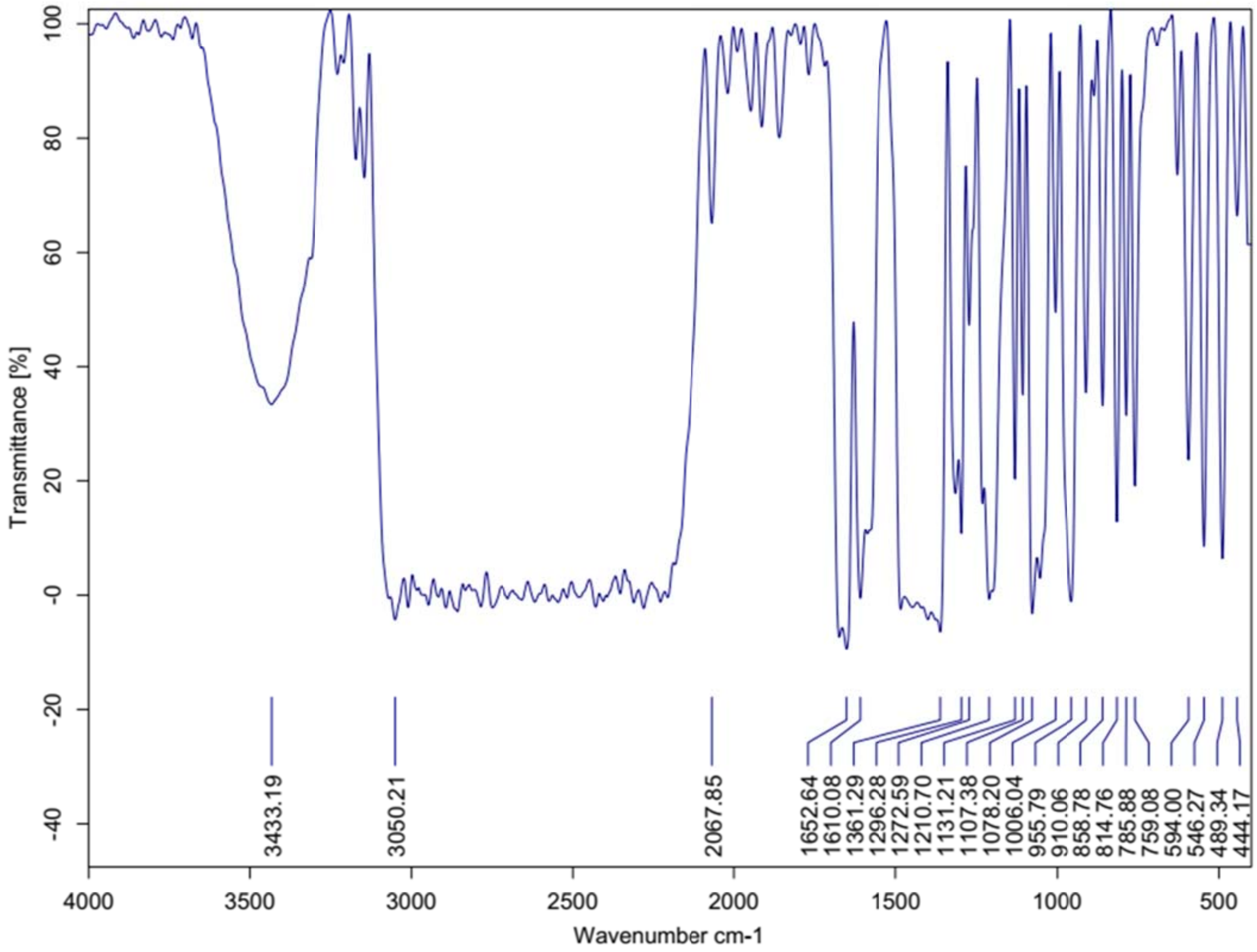

Transmittance [%]
Wavenumber cm-1
3433.19
3050.21
2067.85
1652.64
1610.08
1361.29
1296.28
1272.59
1210.70
1131.21
1107.38
1078.20
1006.04
955.79
910.06
858.78
814.76
785.88
759.08
594.00
546.27
489.34
444.17

^{1}H NMR of tacn(OBz)$_3$, $CDCl_3$, 300K

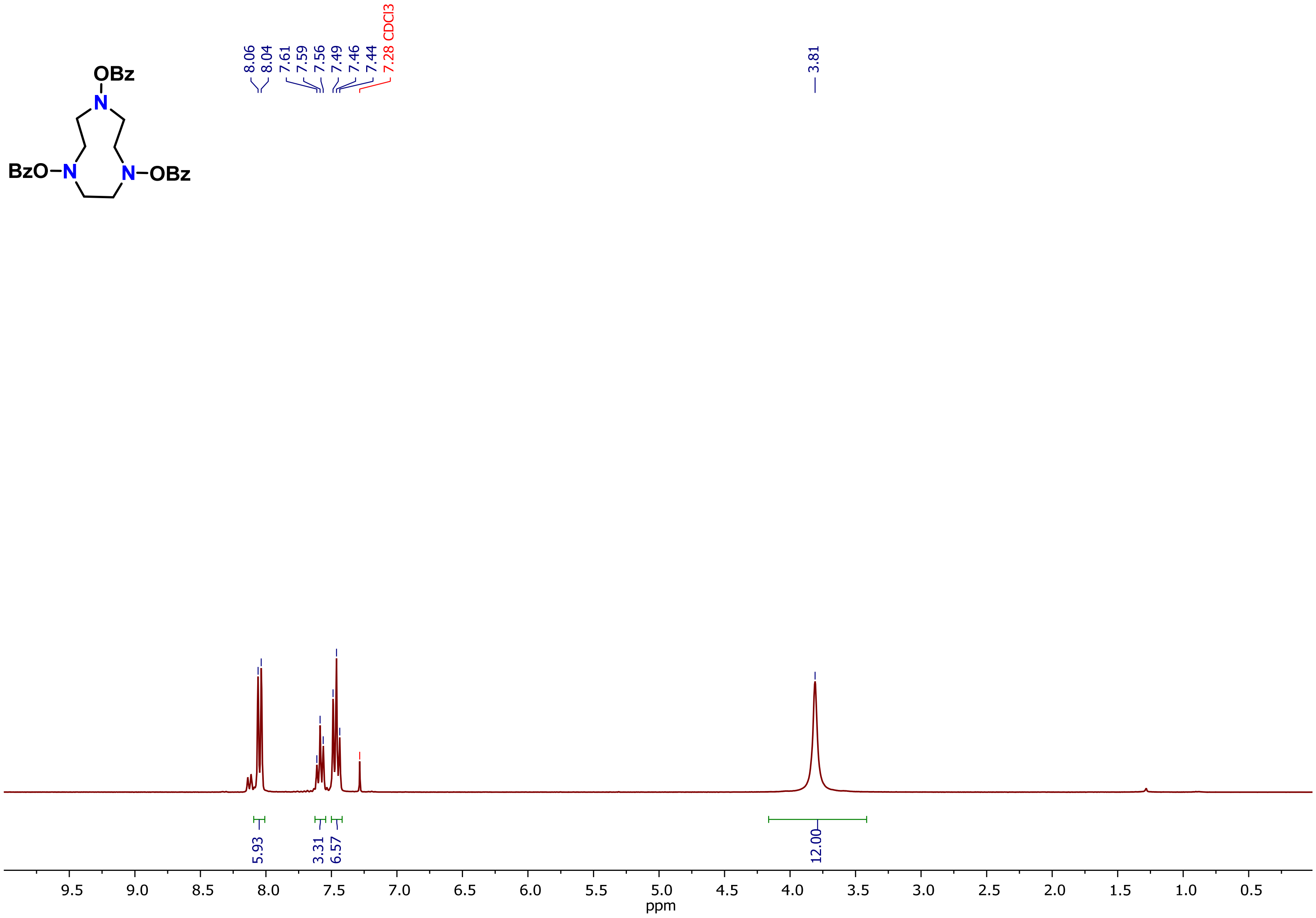

^{13}C NMR of tacn(OBz)$_3$, $CDCl_3$, 300K

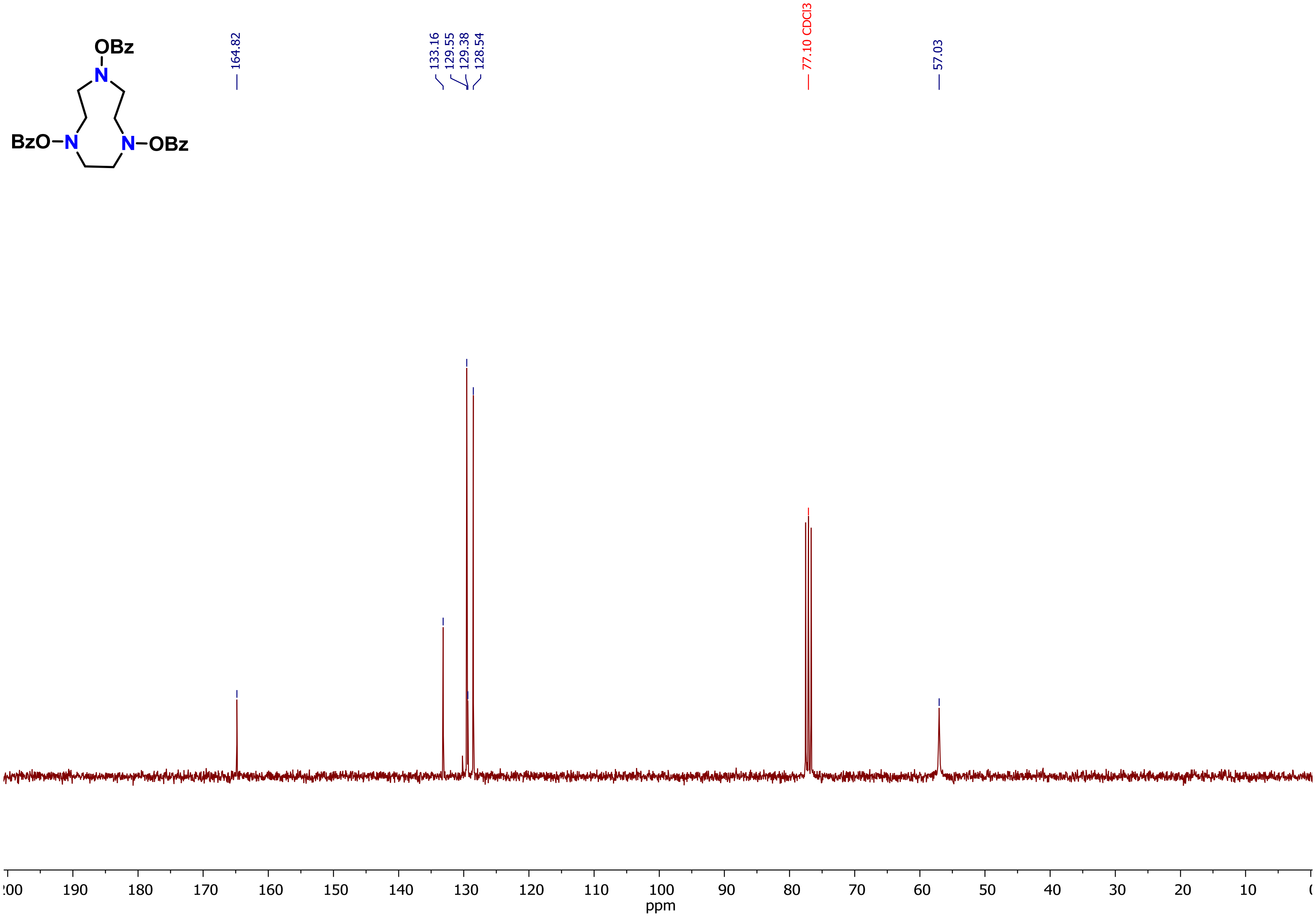

^{13}C DEPT135 of tacn(OBz)$_3$, $CDCl_3$, 300K

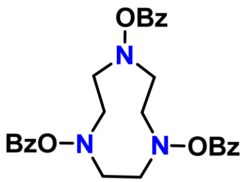

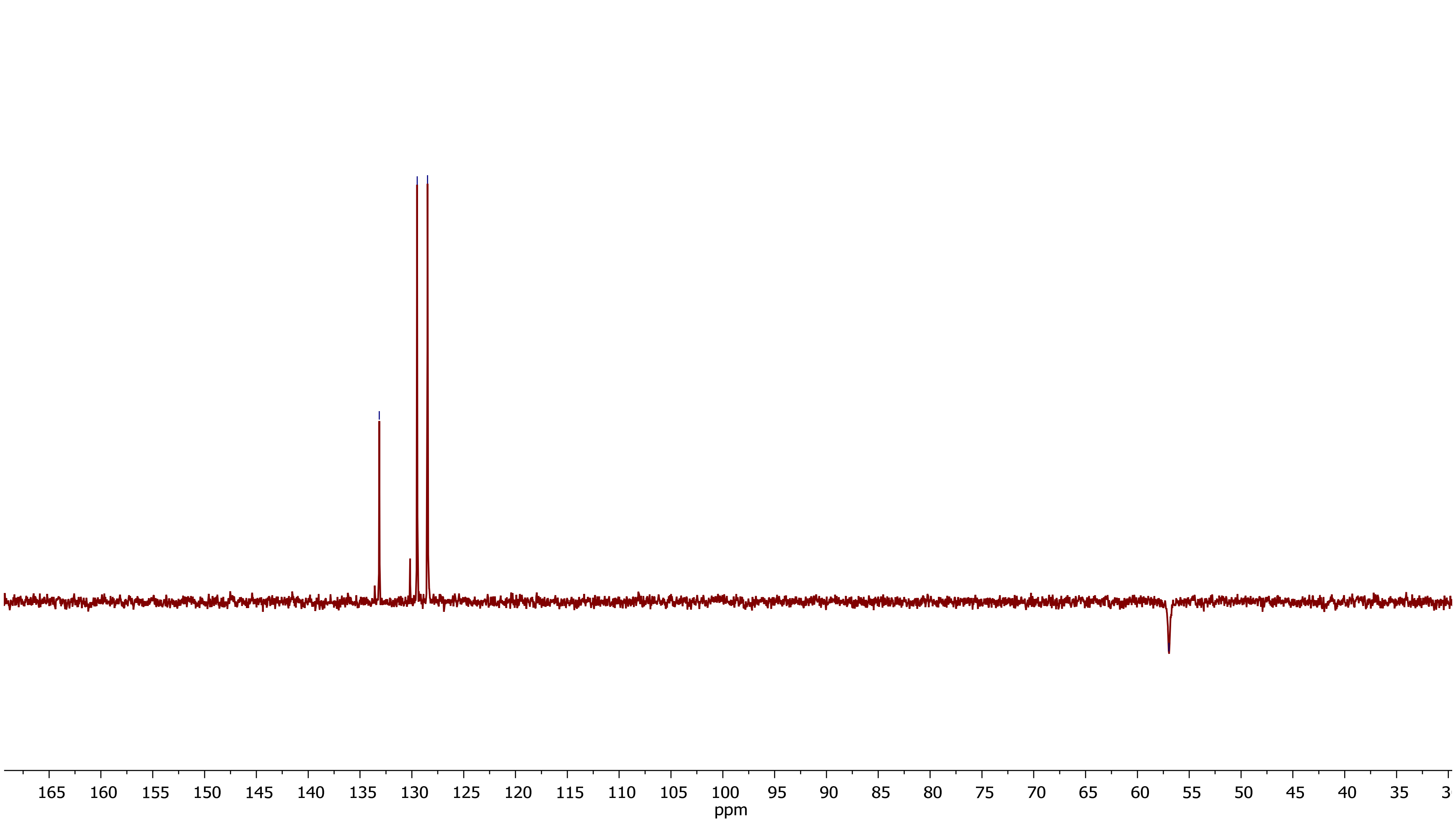

^{1}H NMR of [10]-ane[NOBz]$_3$, $CDCl_3$, 300K

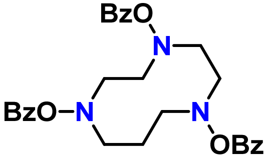

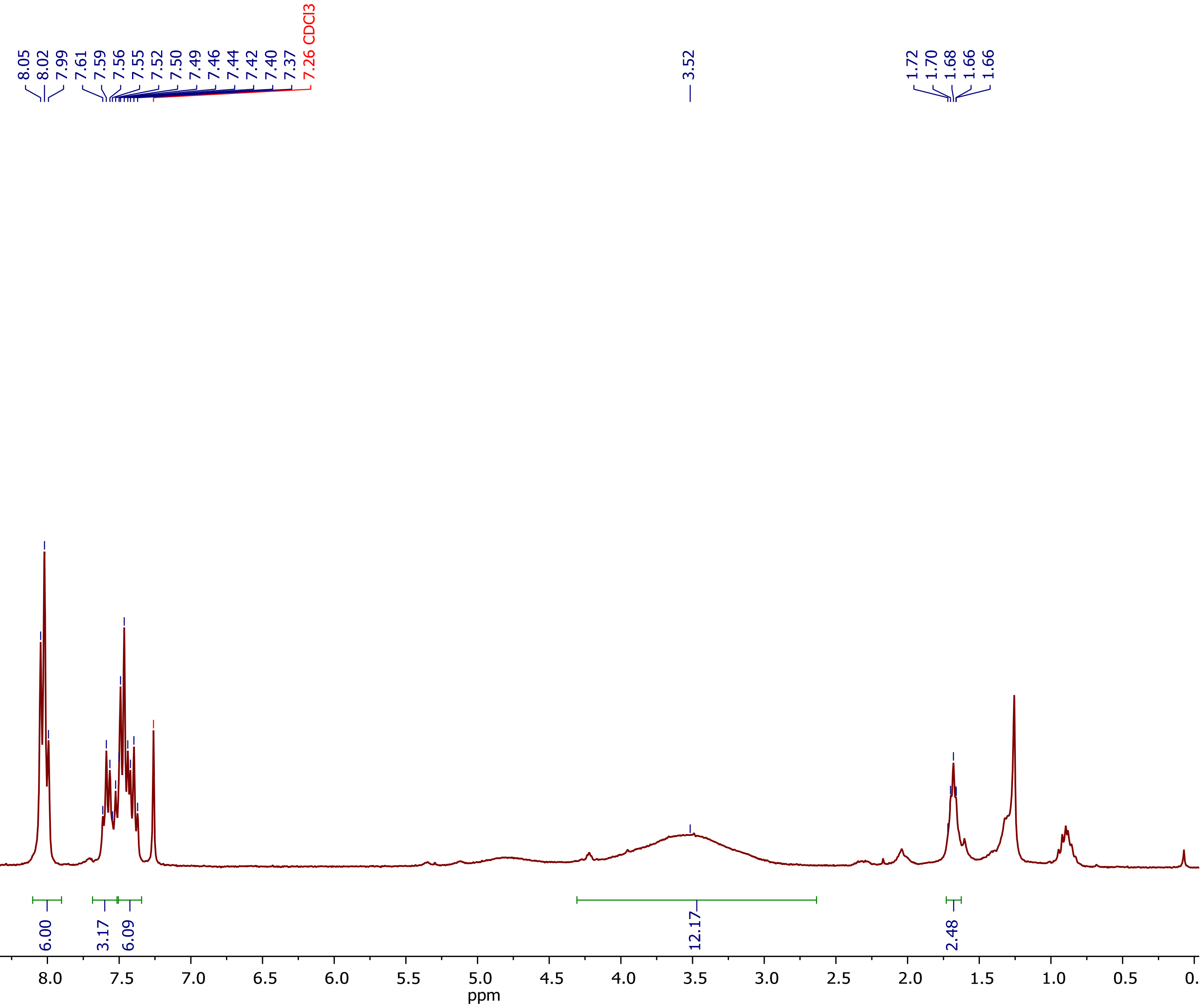

^{13}C NMR of [10]-ane[NOBz]$_3$, $CDCl_3$, 300K

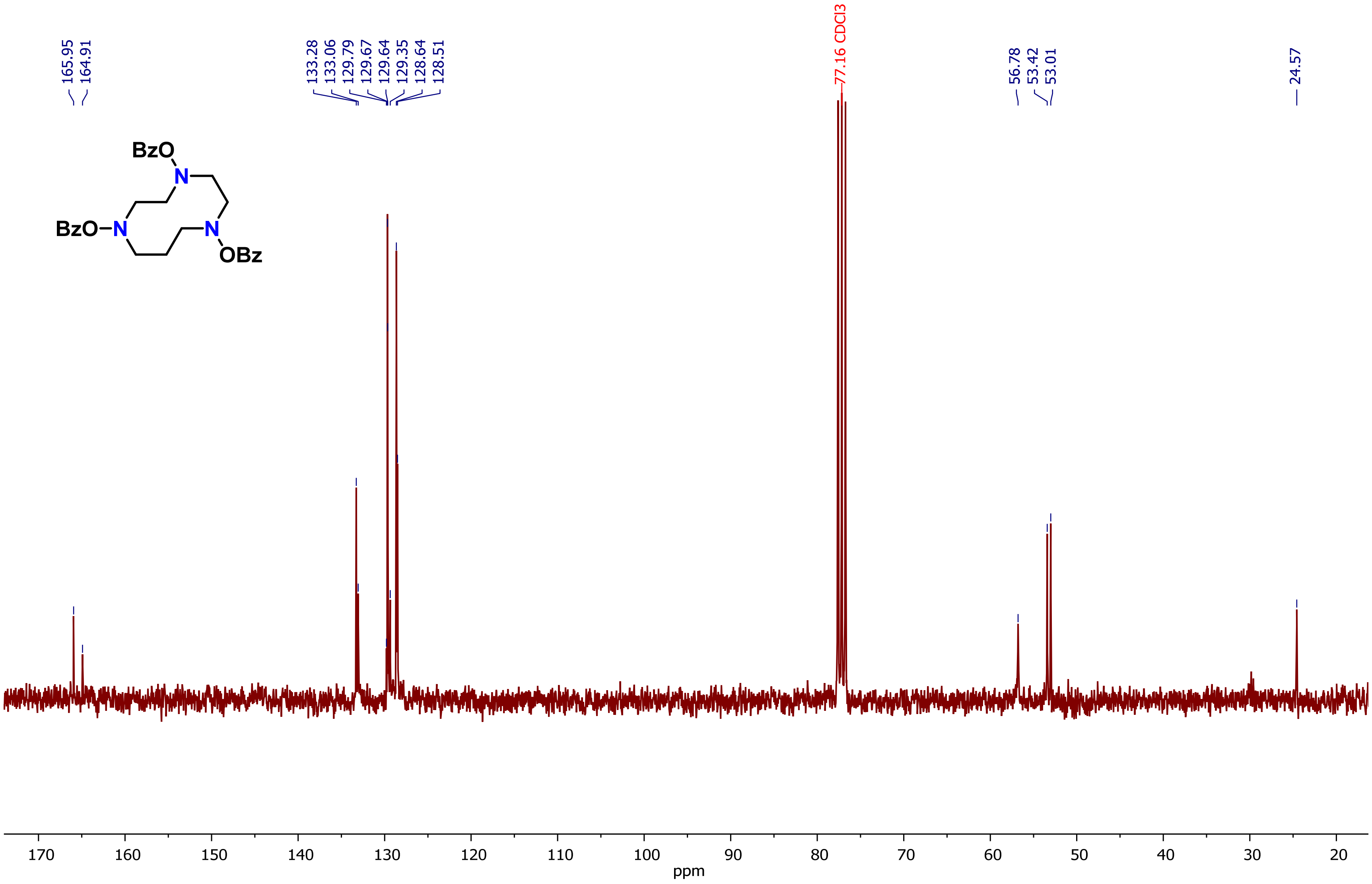

^{1}H NMR of [11]-ane[NOBz]$_3$, $CDCl_3$, 300K

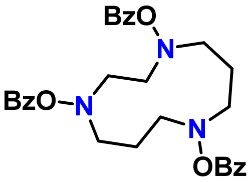

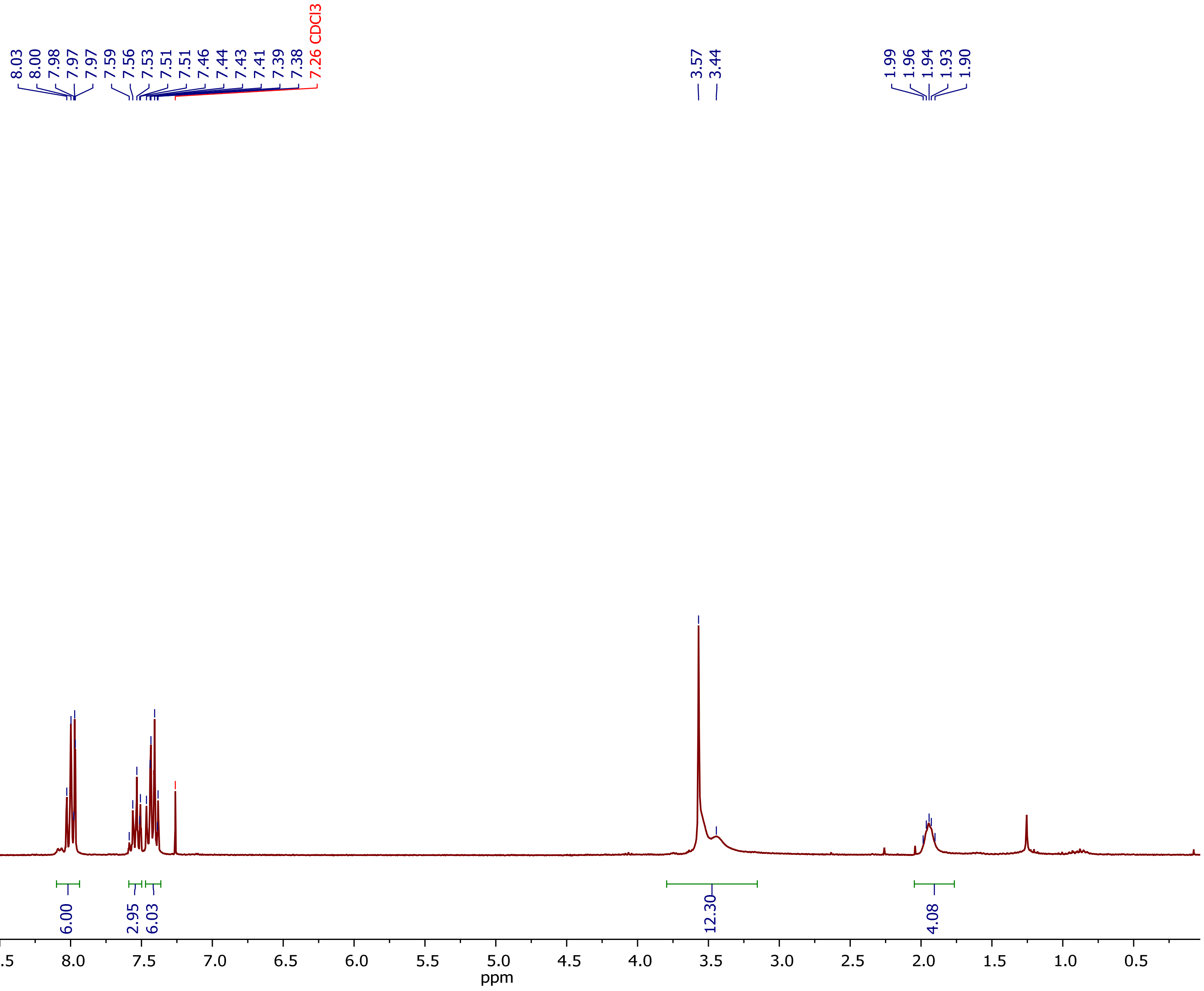

^{13}C NMR of [11]-ane[NOBz]$_3$, $CDCl_3$, 300K

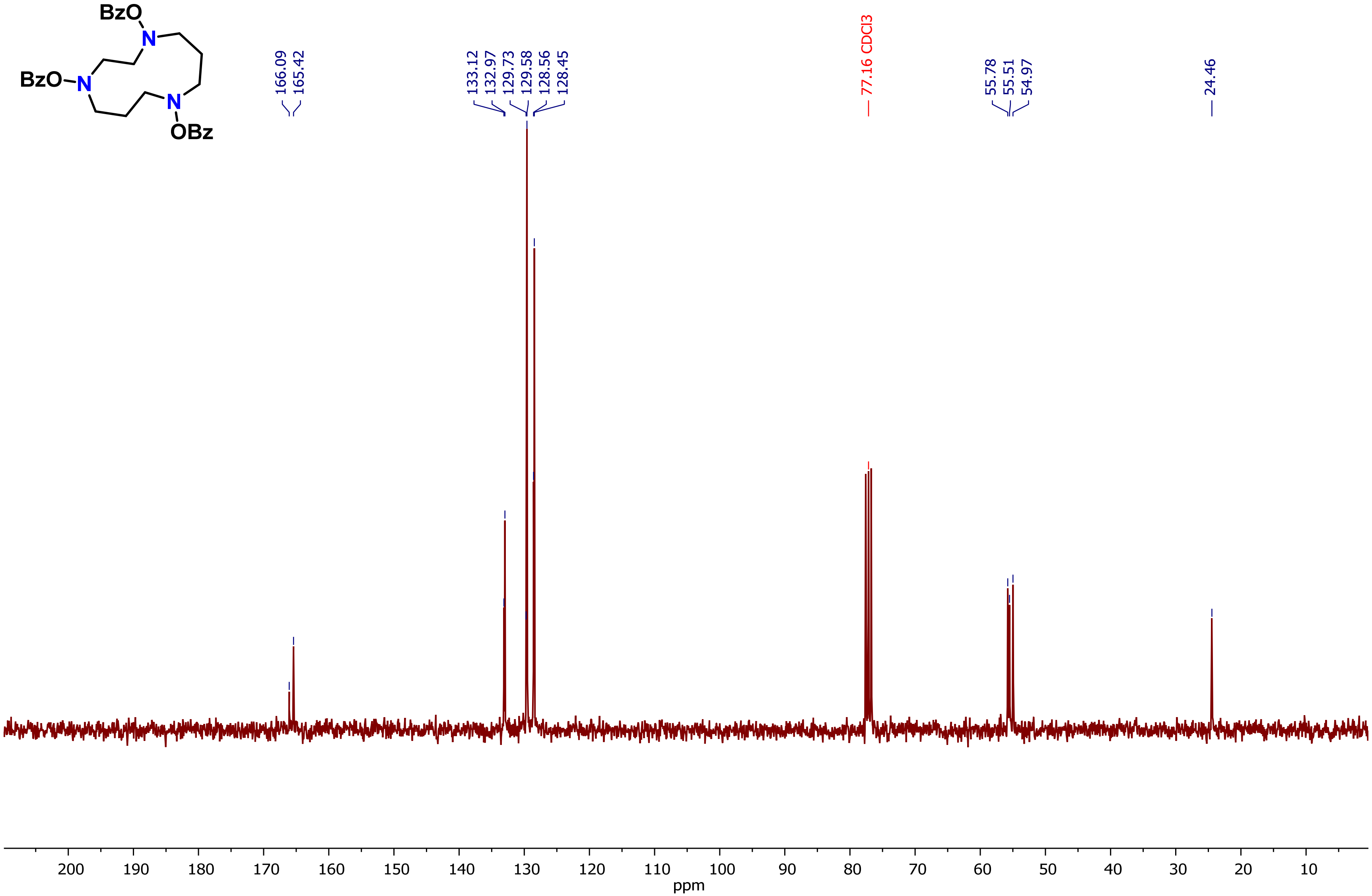

^{1}H NMR of tacd(OBz)$_3$, $CDCl_3$, 300K

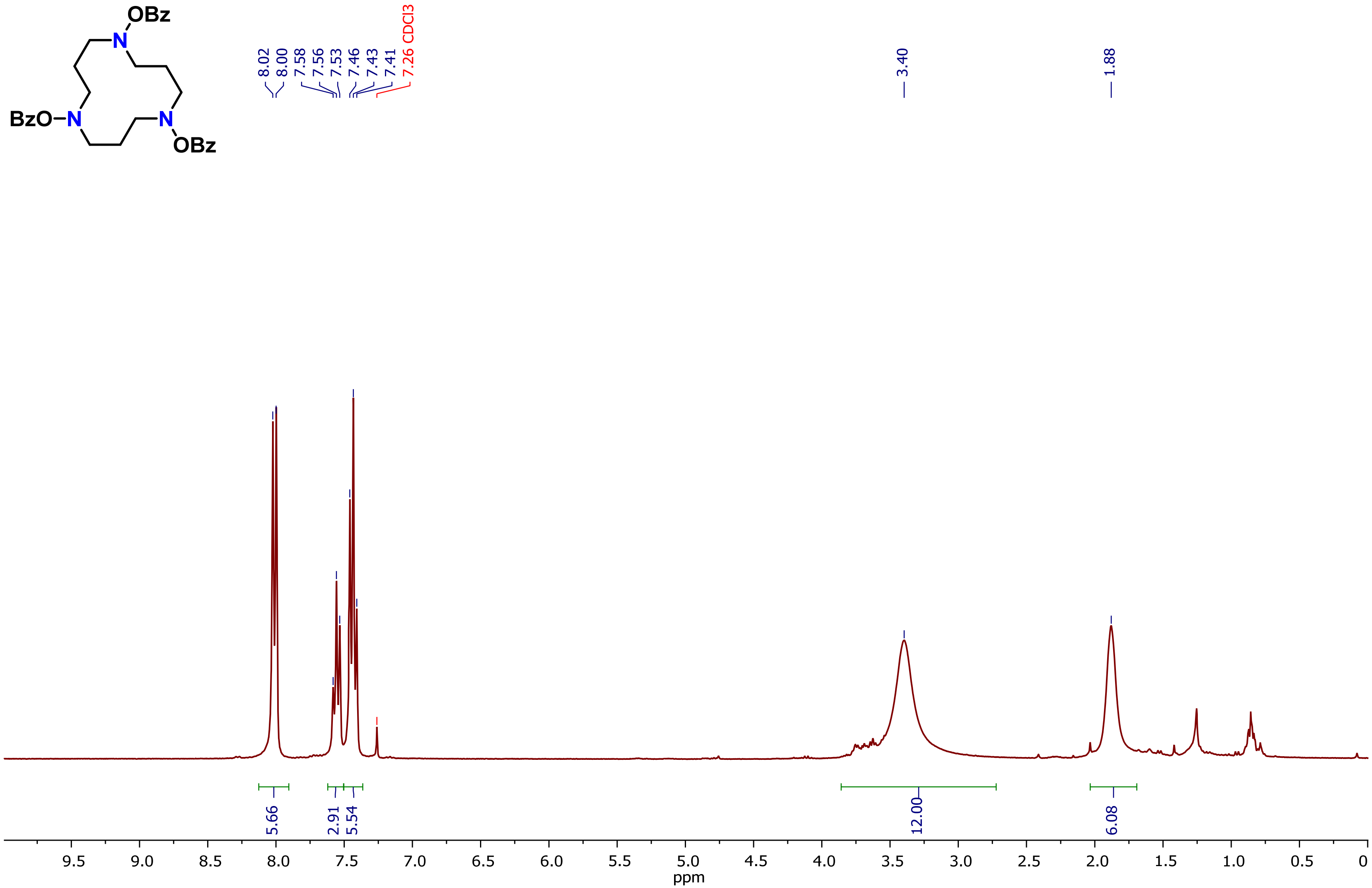

^{13}C NMR of tacd(OBz)$_3$, CDCl$_3$, 300K

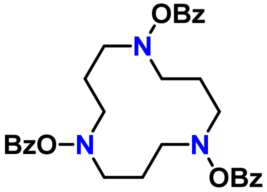

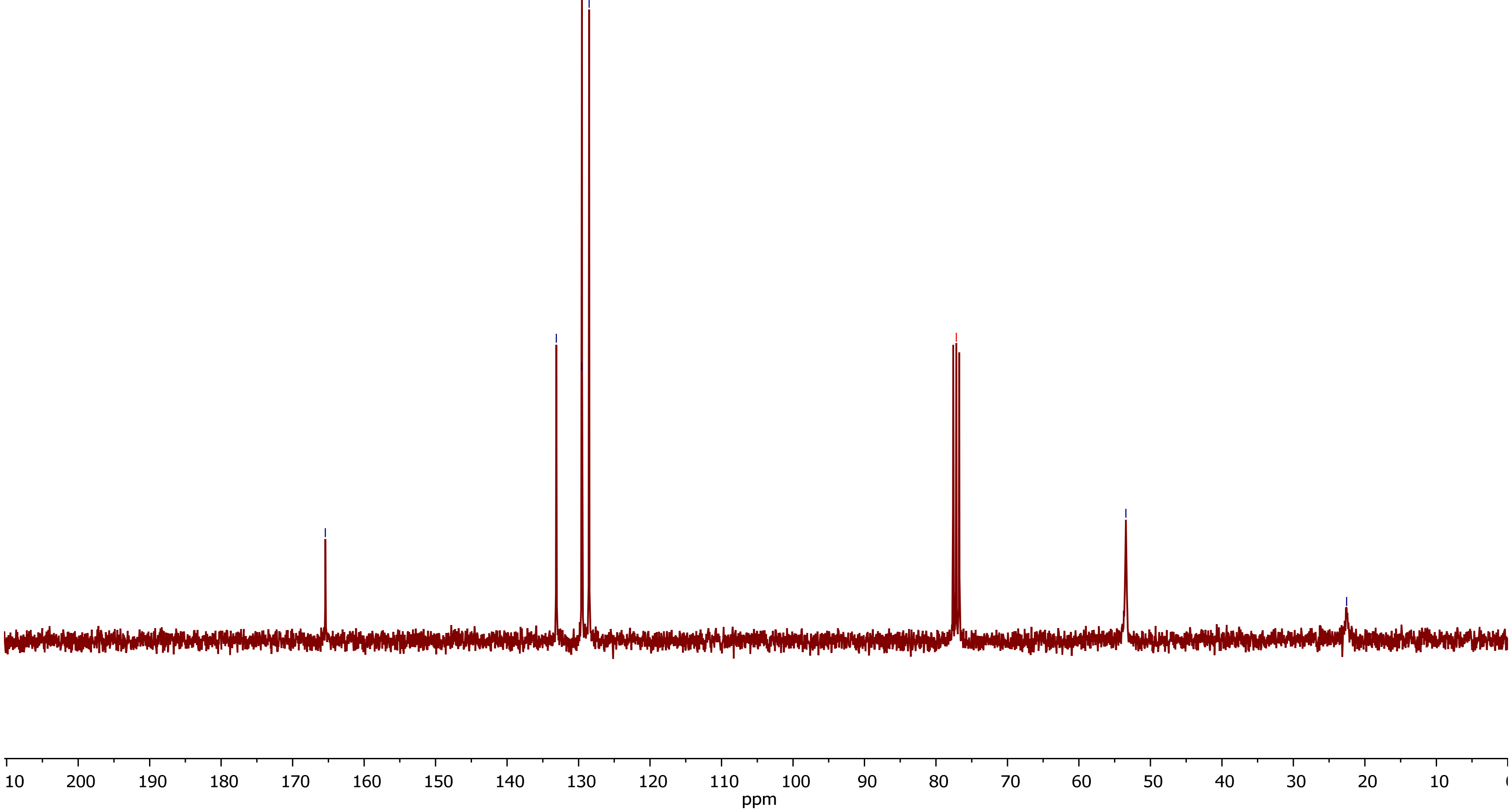

^{1}H NMR of [13]-ane[NOBz]$_3$, $CDCl_3$, 298K

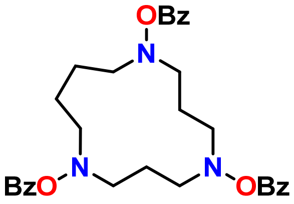

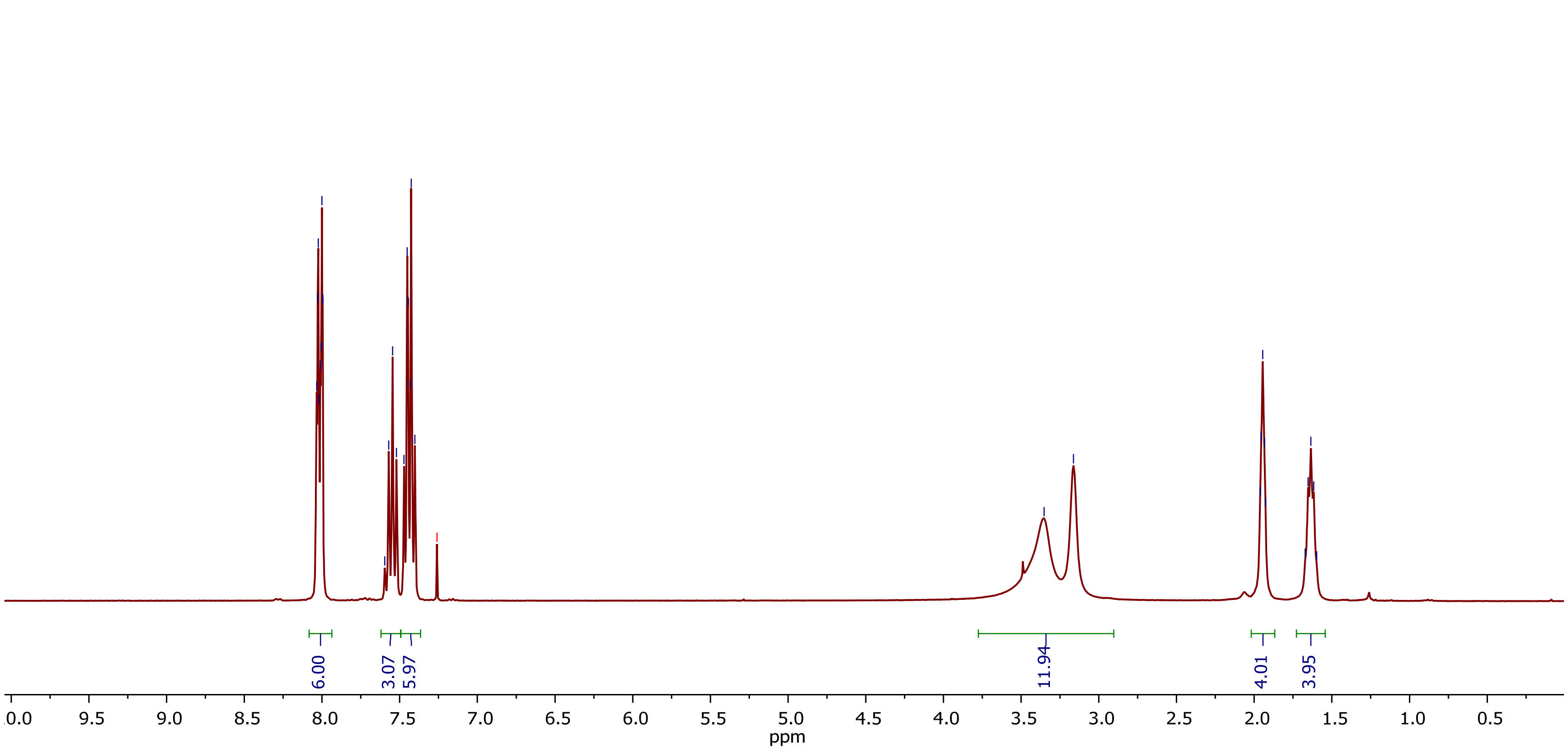

^{13}C NMR of [13]-ane[NOBz]$_3$, $CDCl_3$, 298K

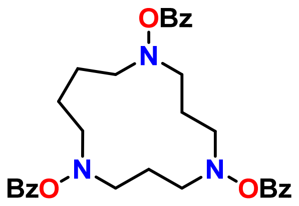

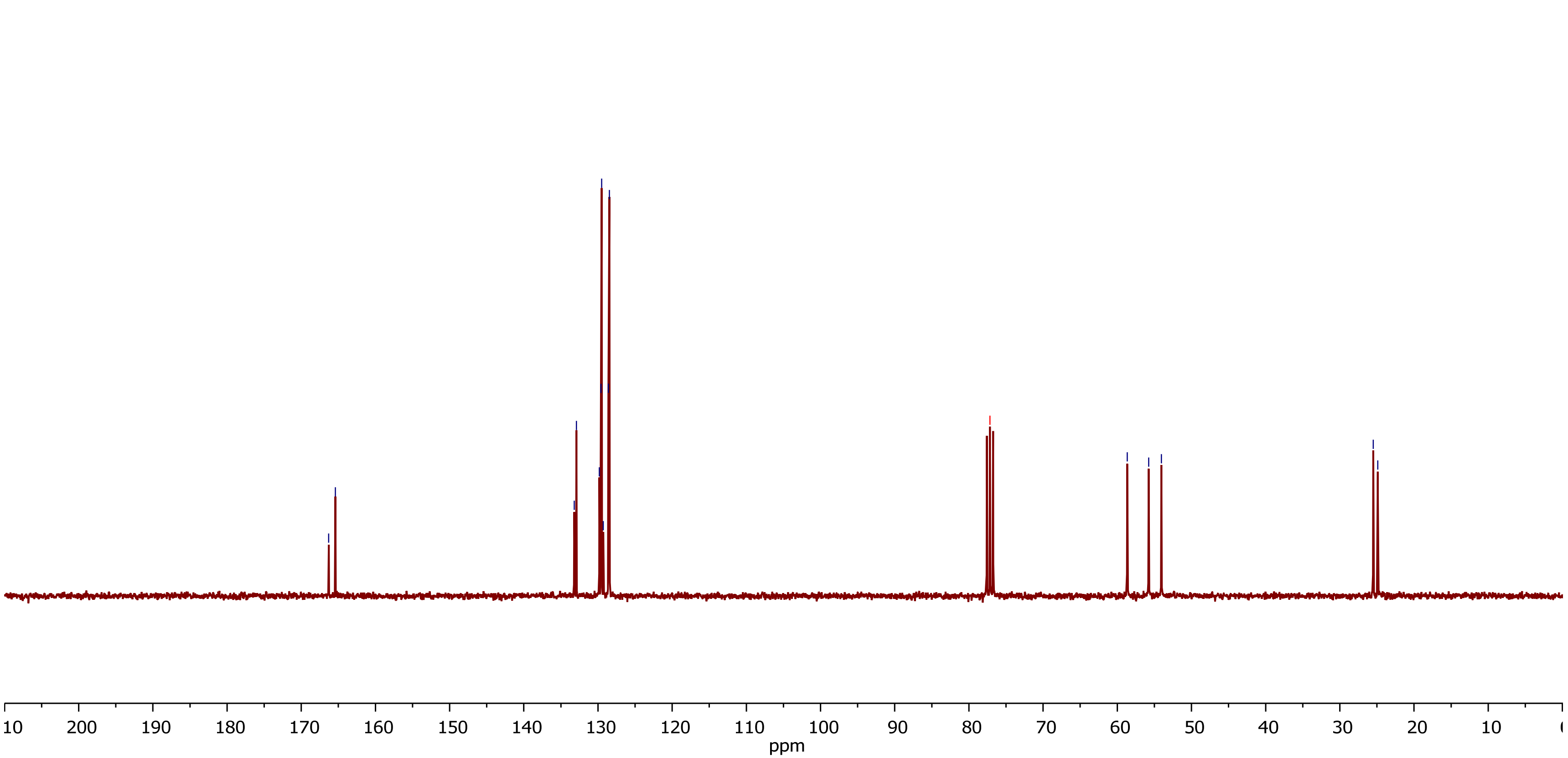

^{1}H-^{13}C HSQC of [13]-ane[NOBz]$_3$, $CDCl_3$, 298K

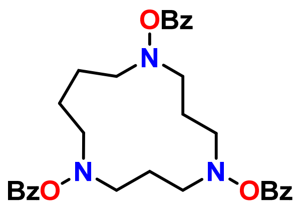

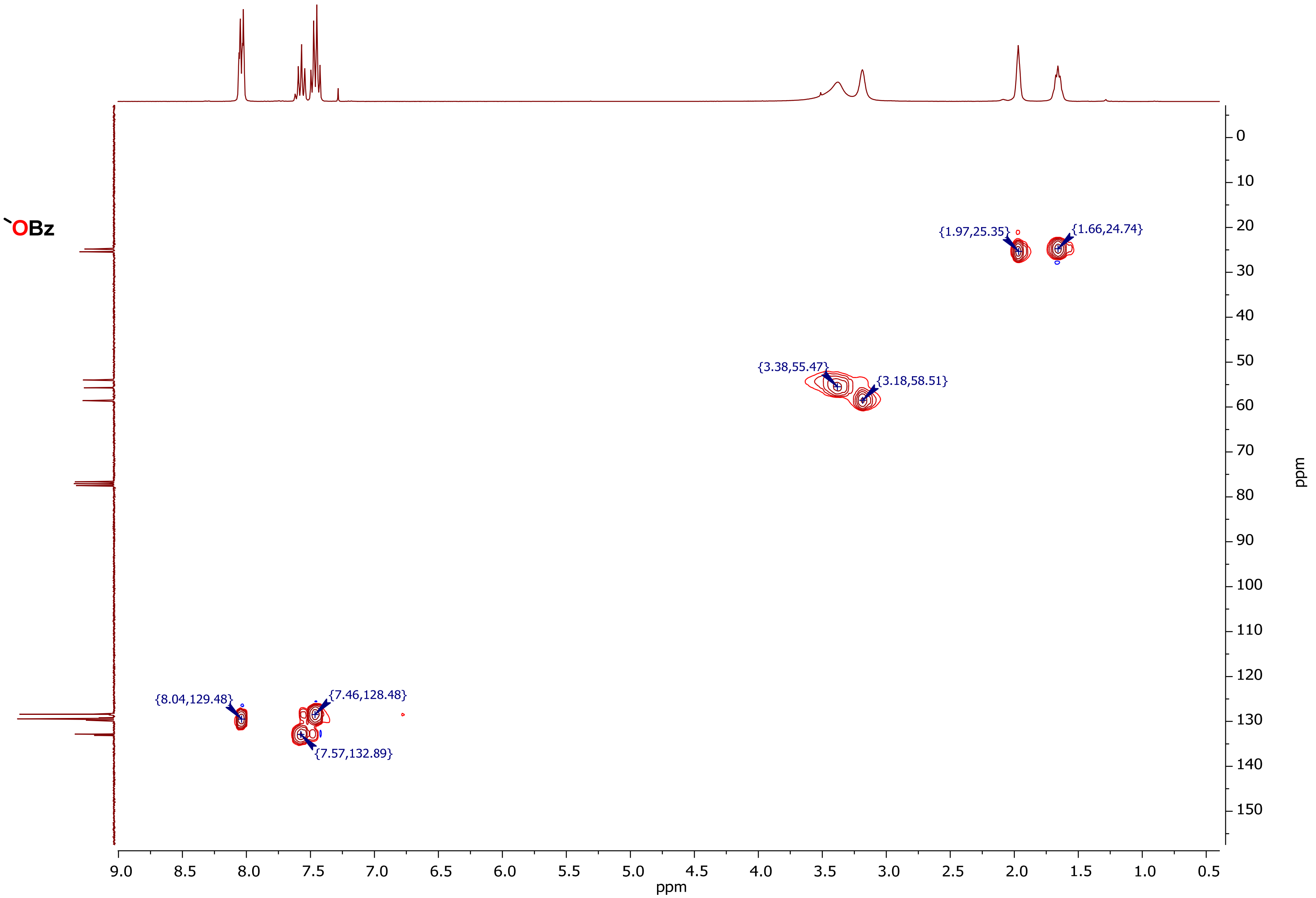

^{1}H NMR of cyclam(OBz)$_4$, $CDCl_3$, 300K

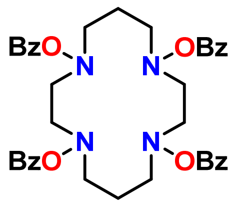

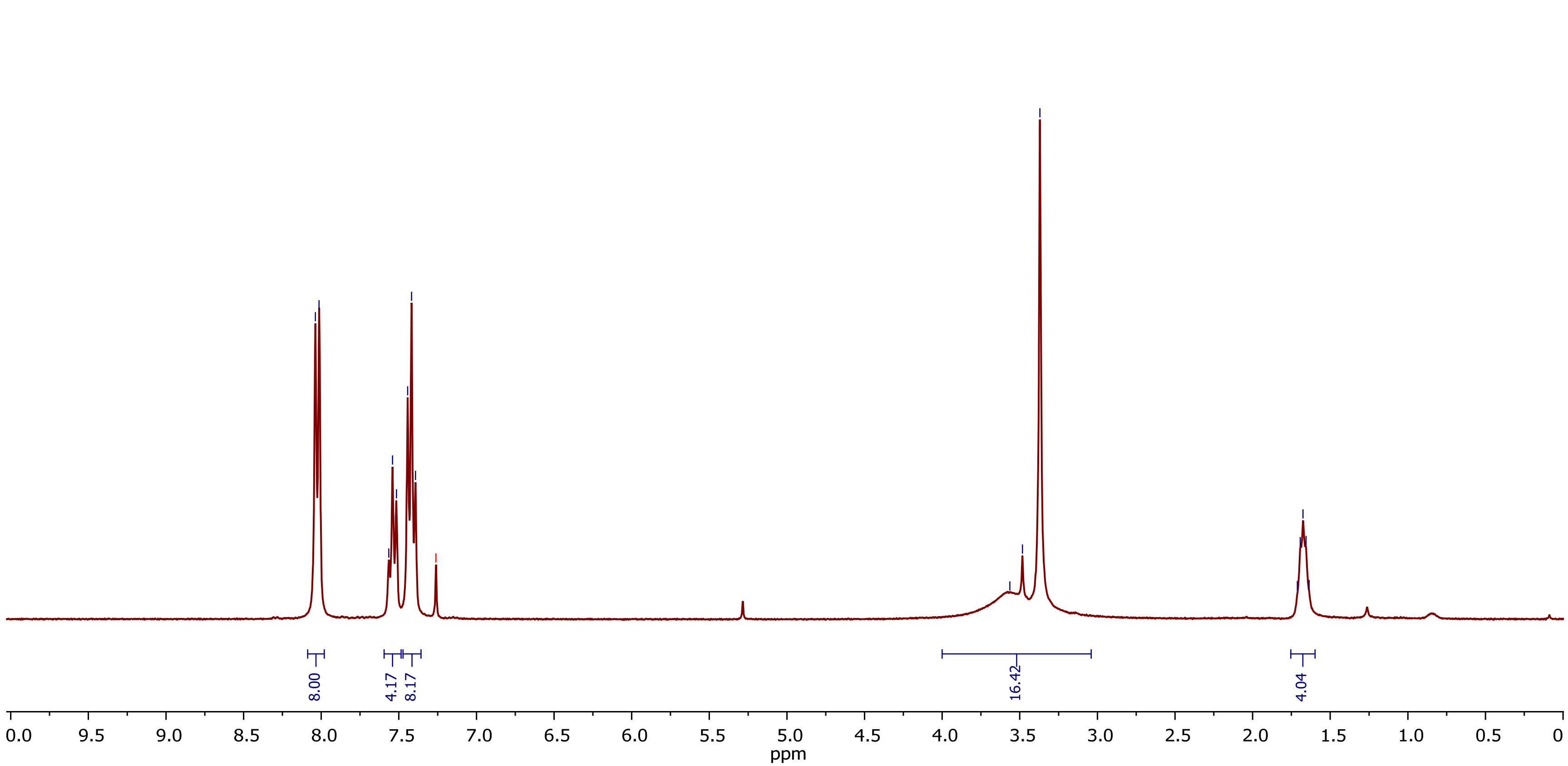

^{13}C NMR of cyclam(OBz)$_4$, $CDCl_3$, 300K

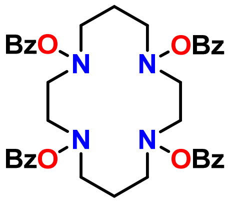

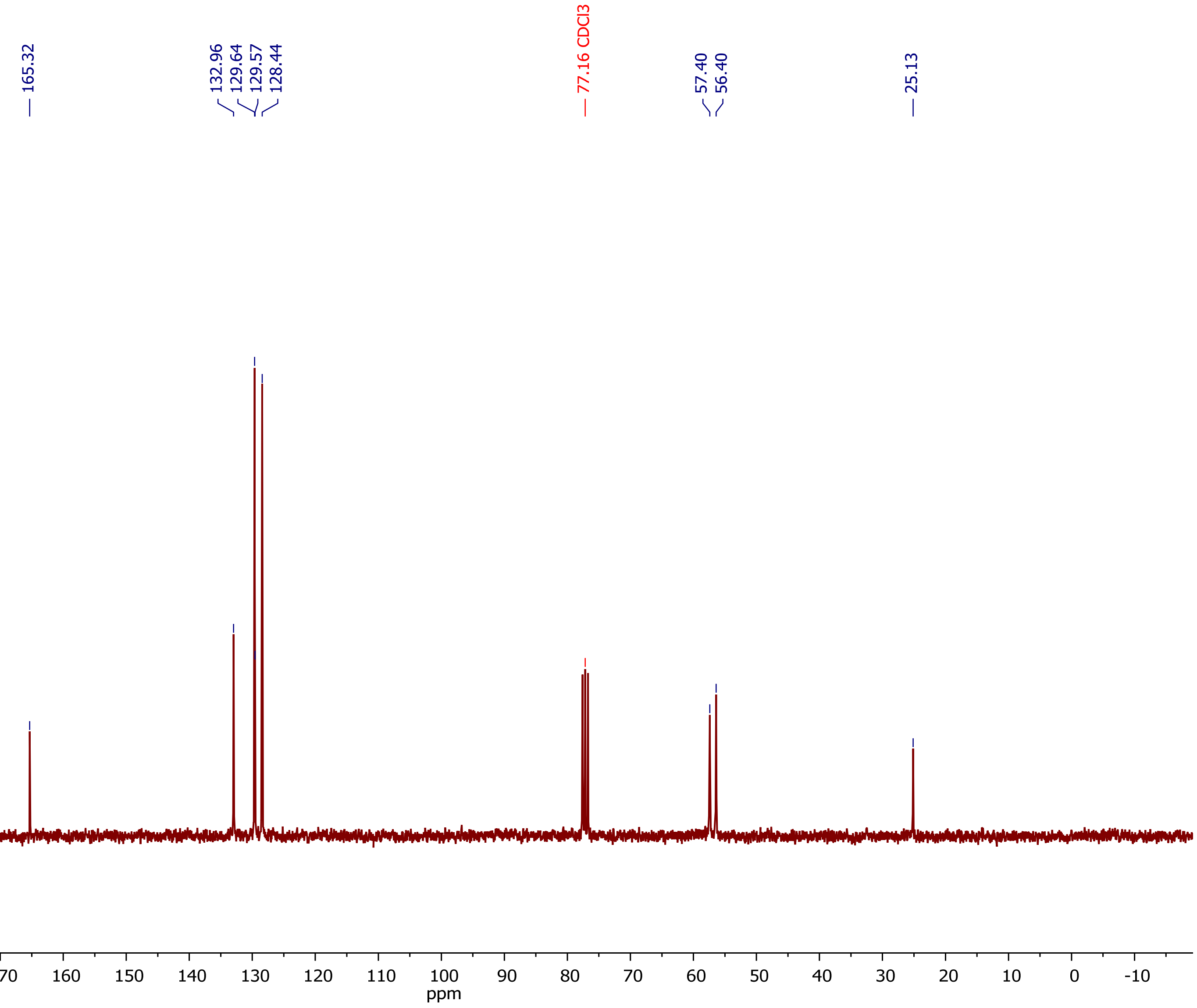

^{13}C DEPT135 NMR of cyclam(OBz)$_4$, $CDCl_3$, 300K

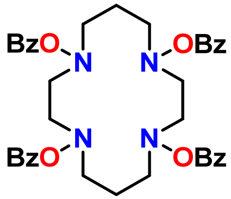

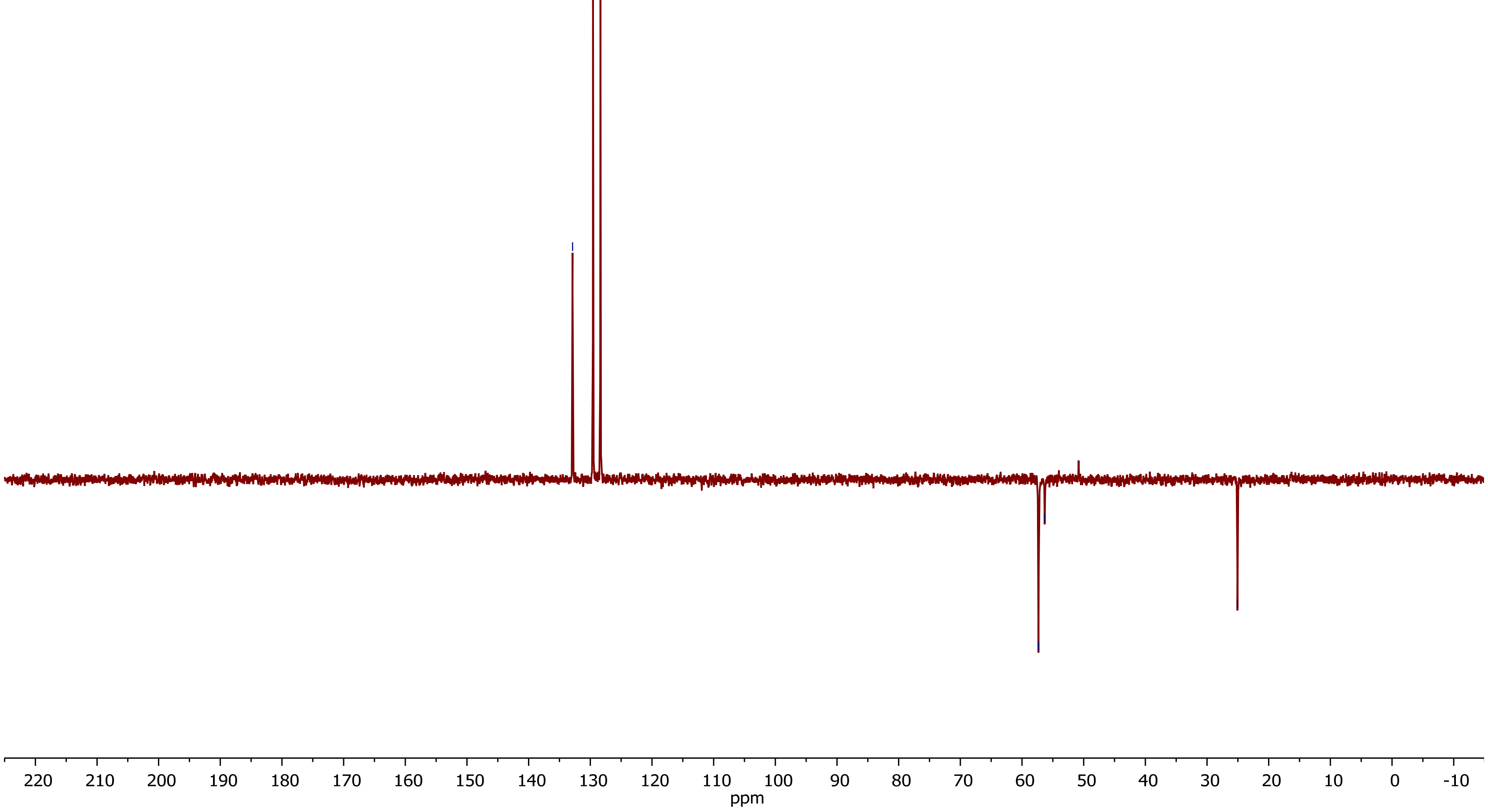

^{1}H-^{13}C HSQC of cyclam(OBz)$_4$, $CDCl_3$, 300K

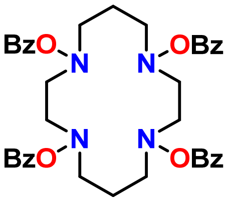

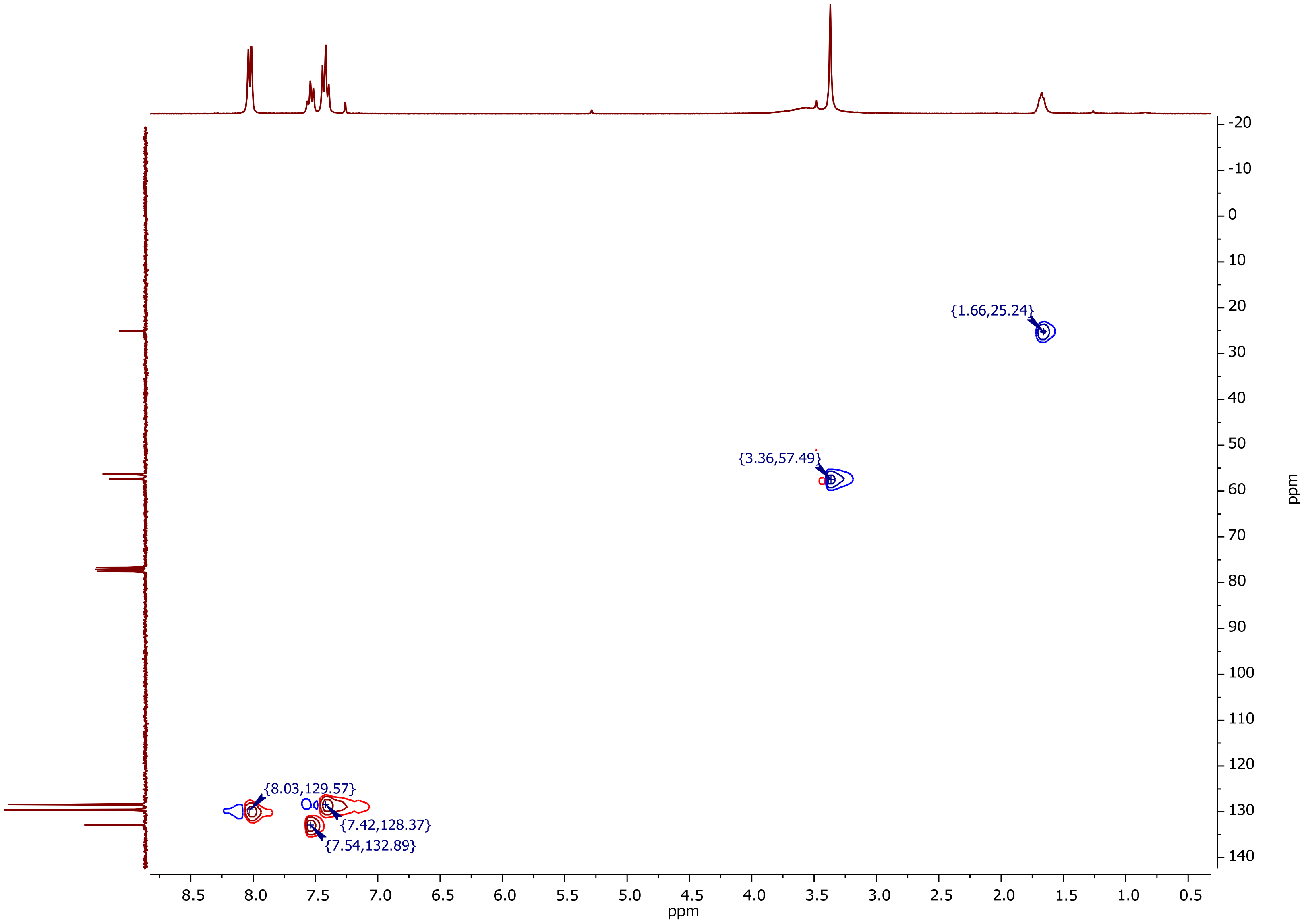

^{1}H NMR of Bz-cyclam(OBz)$_3$, $CDCl_3$, 298K

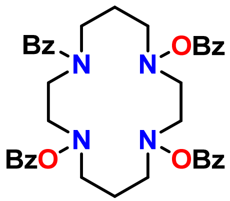

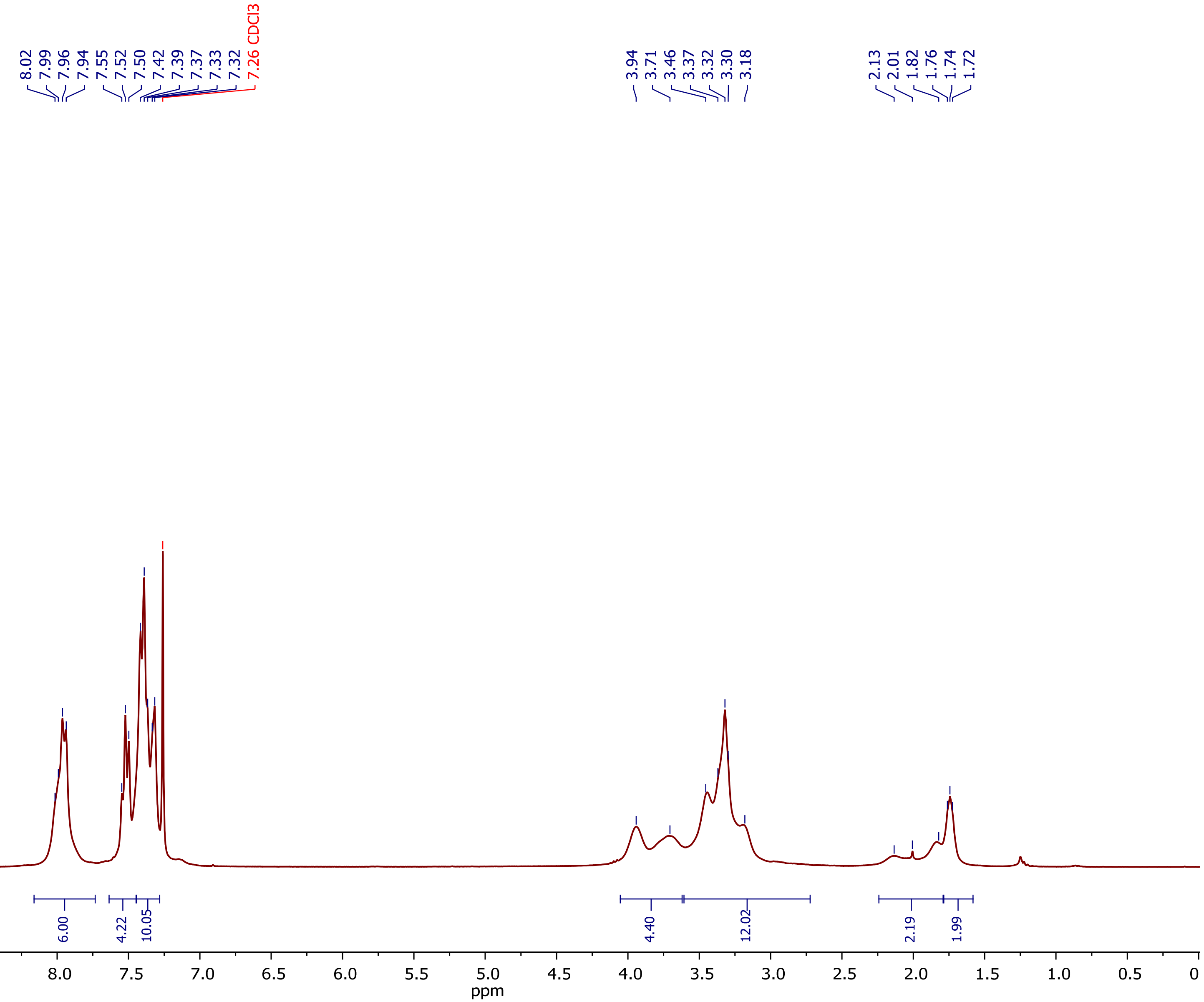

^{13}C NMR of Bz-cyclam(OBz)$_3$, $CDCl_3$, 298K

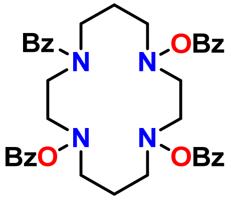

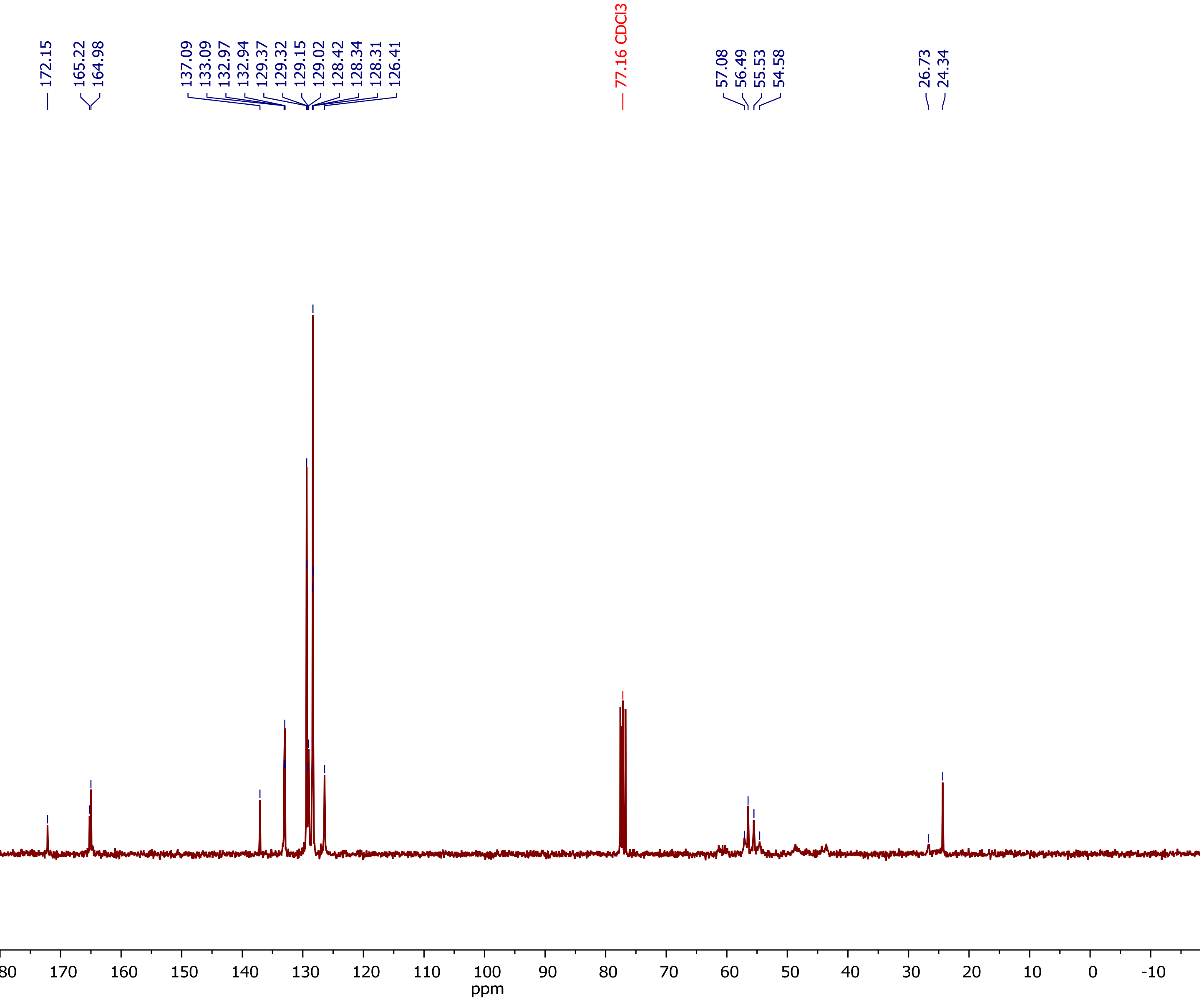

^1H-^{13}C HSQC of Bz-cyclam(OBz)$_3$, $CDCl_3$, 298K

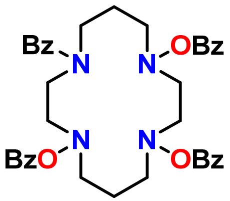

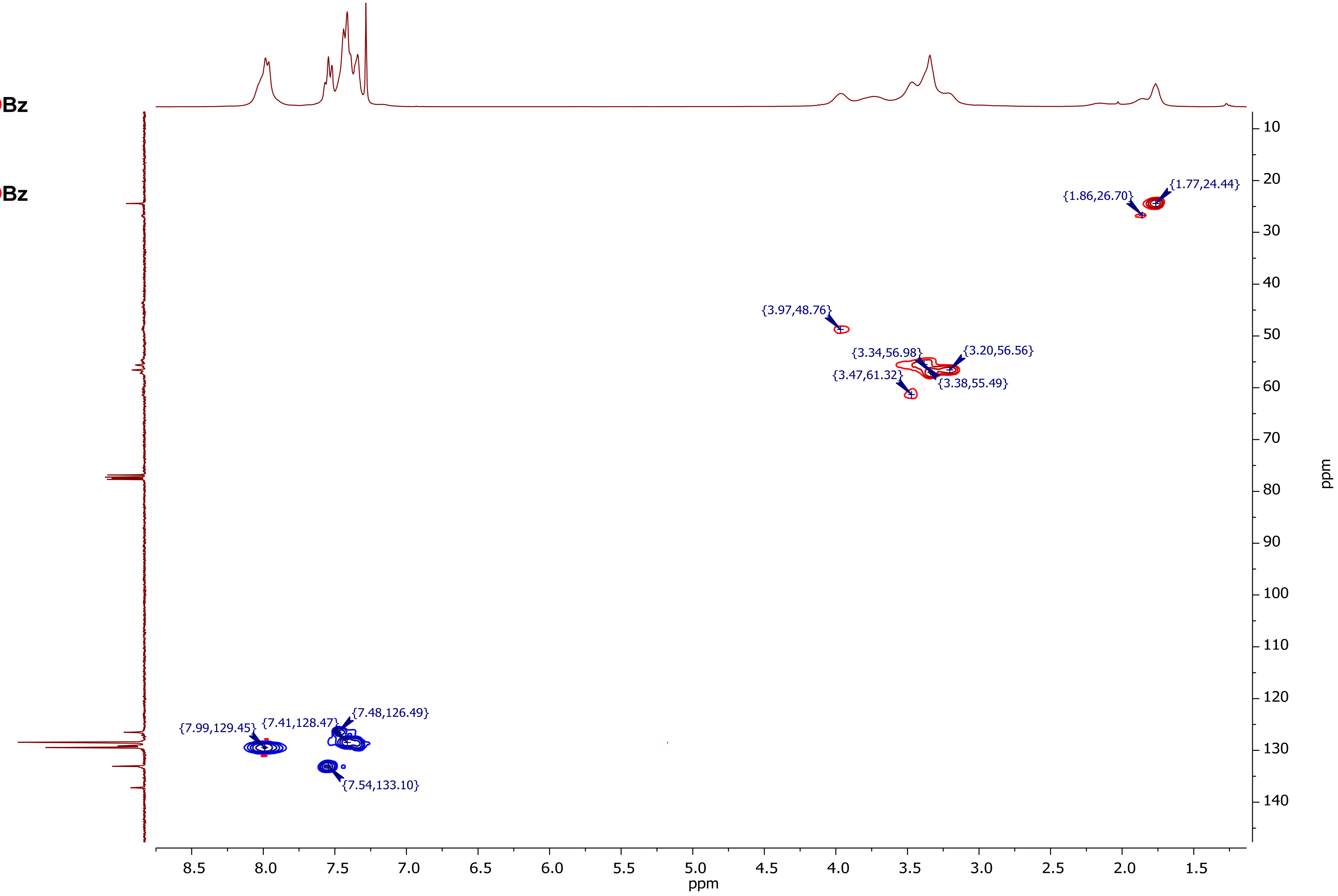

^{1}H NMR of EtC(O)-cyclam(OBz)$_3$, CDCl$_3$, 298K

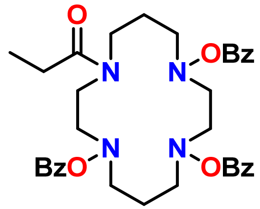

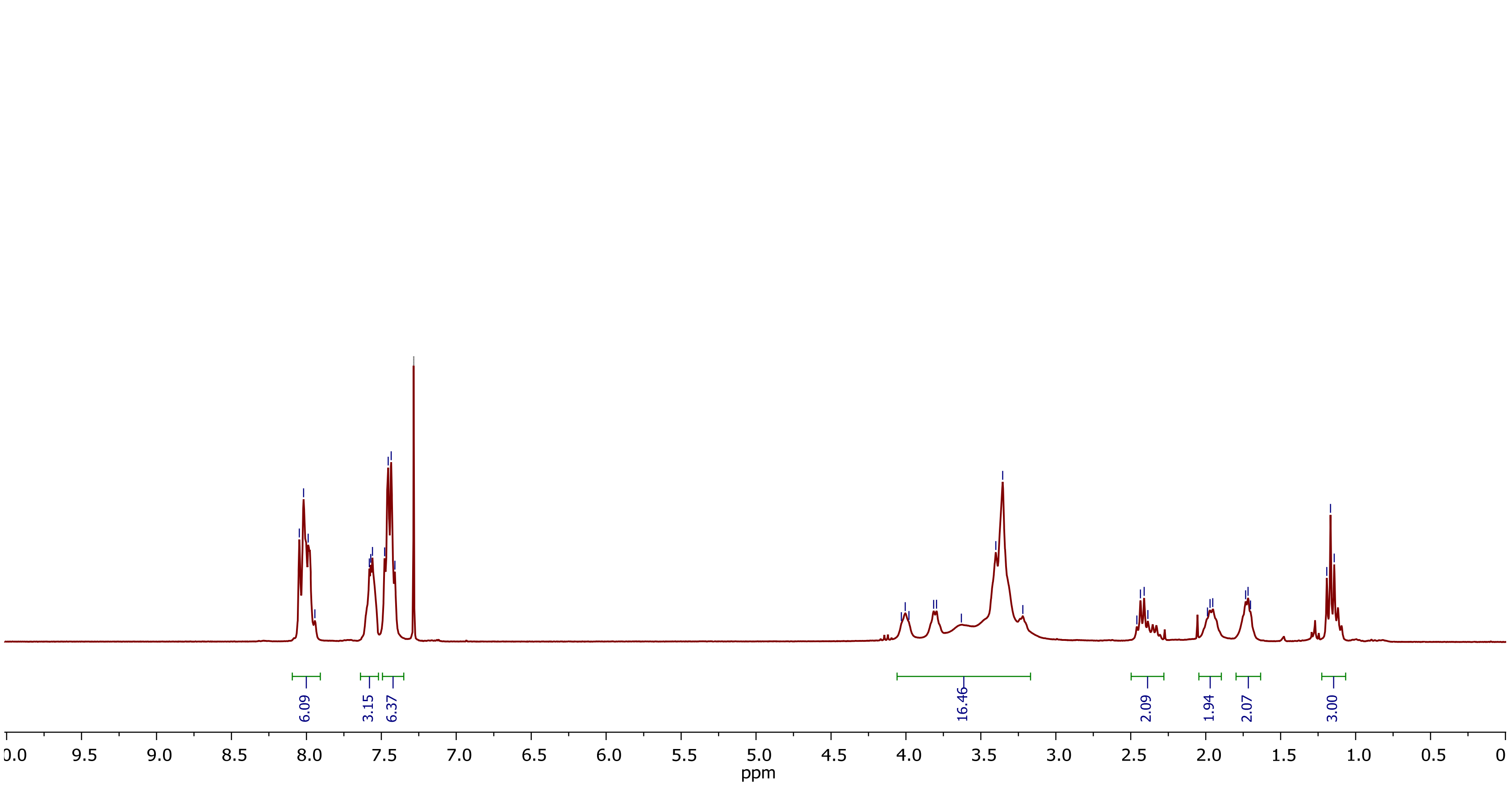

^{13}C NMR of EtC(O)-cyclam(OBz)$_3$, $CDCl_3$, 298K

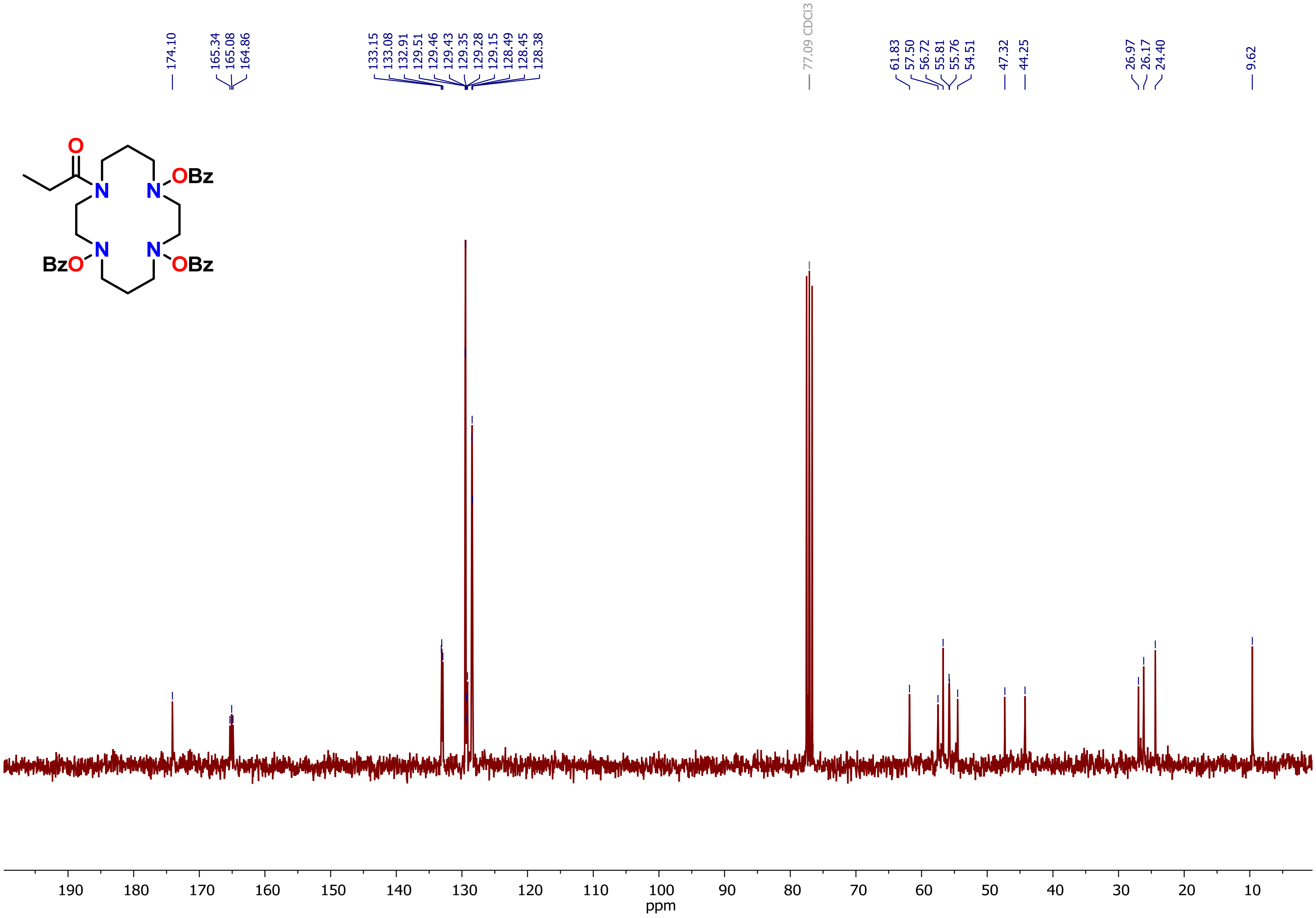

^{1}H-^{13}C HSQC of EtC(O)-cyclam(OBz)$_3$, $CDCl_3$, 298K

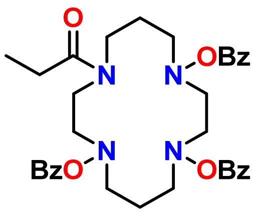

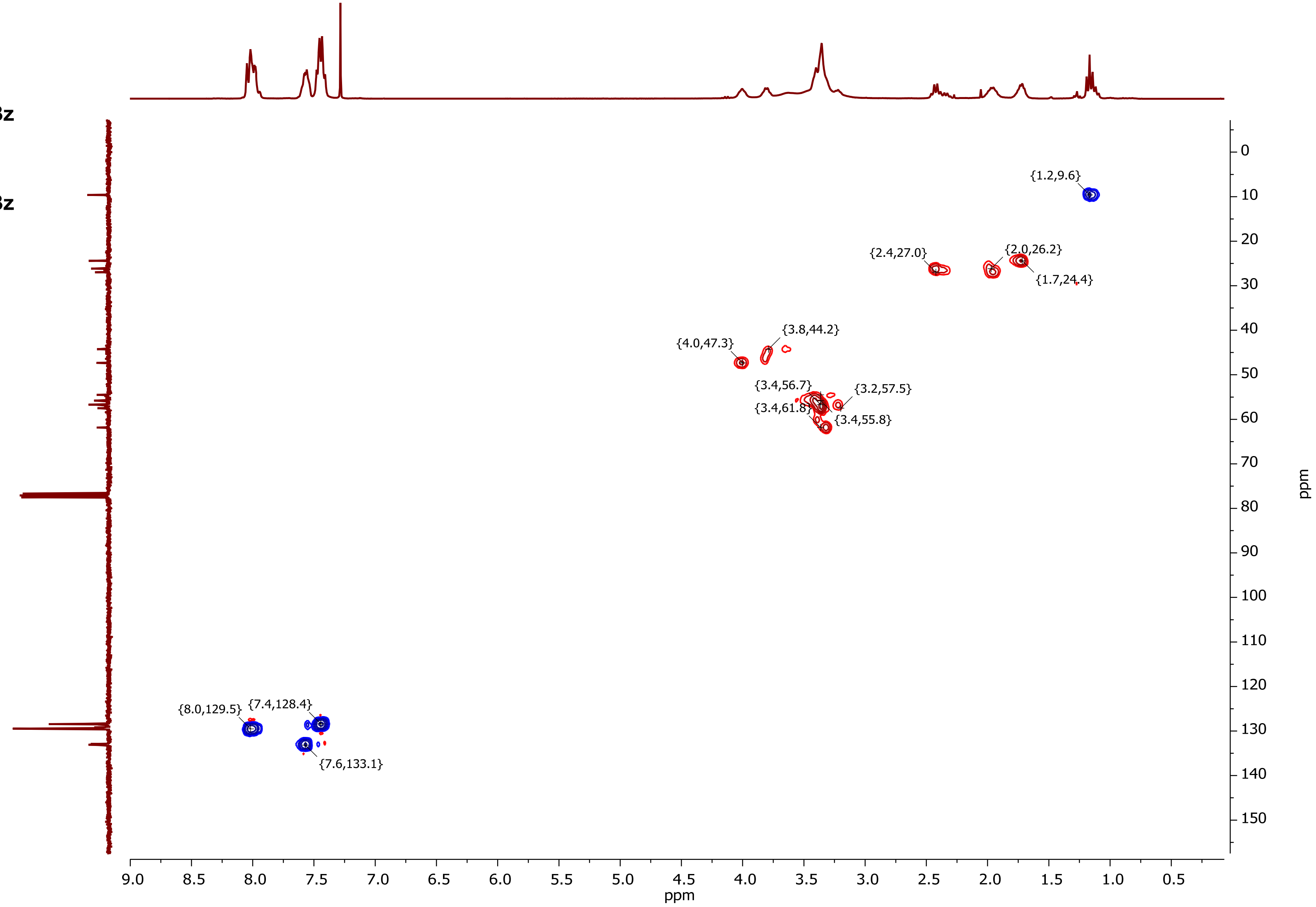

^{1}H NMR of Ms-cyclam(OBz)$_3$, $CDCl_3$, 298K

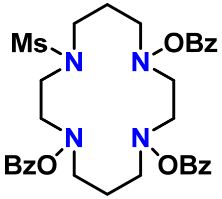

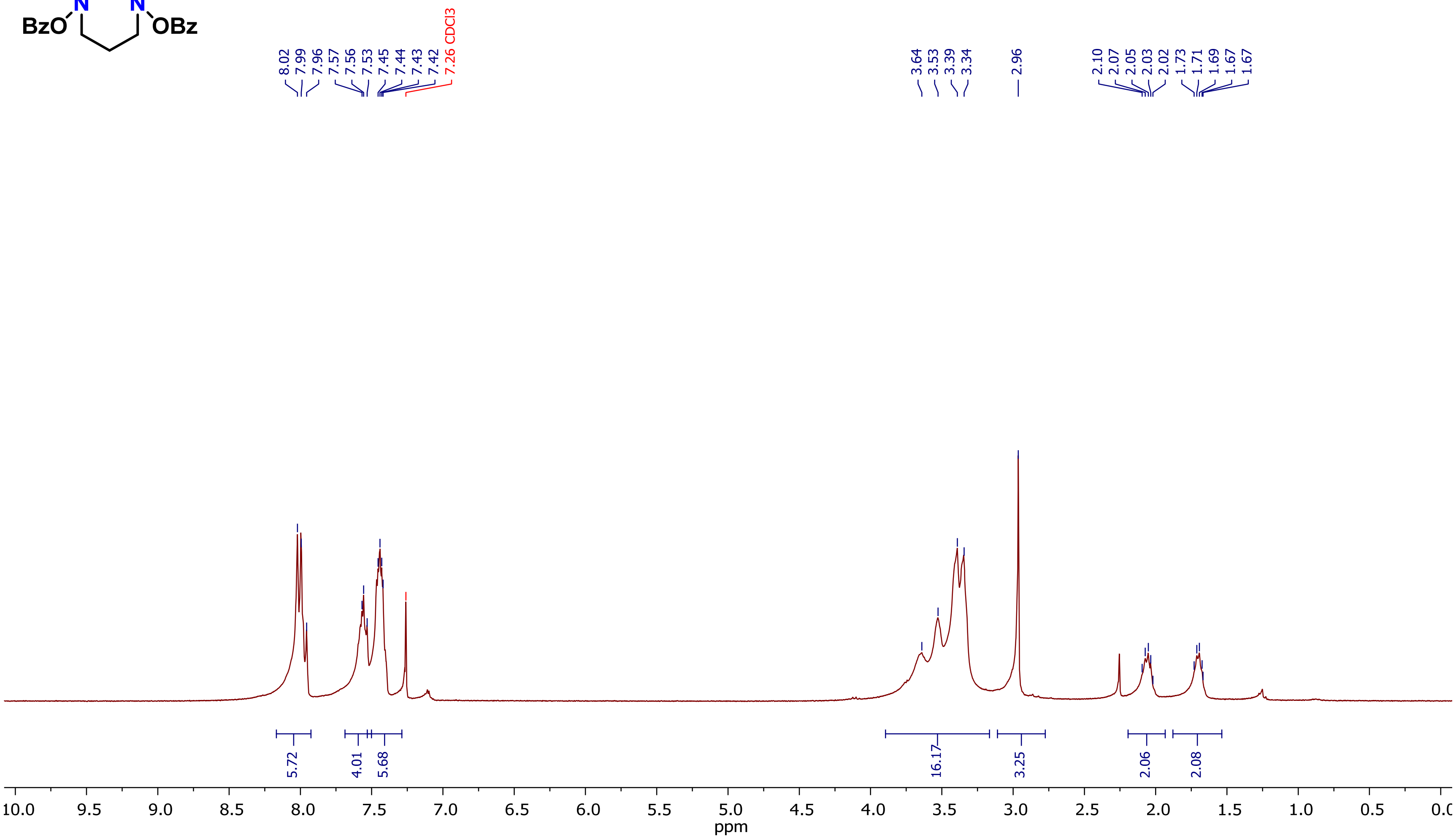

^{13}C NMR of Ms-cyclam(OBz)$_3$, $CDCl_3$, 298K

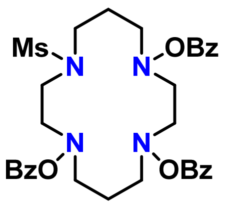

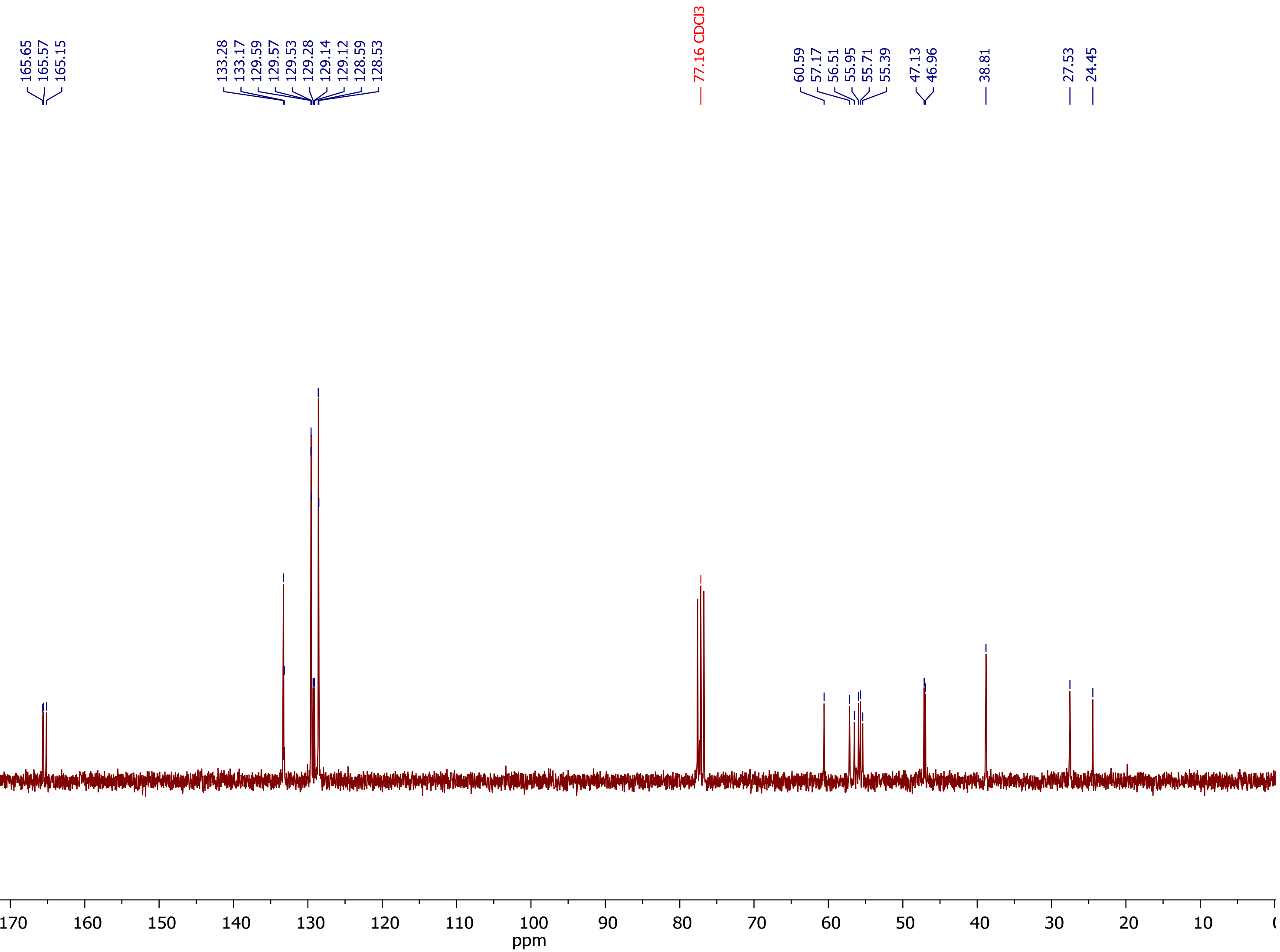

^{1}H-^{13}C HSQC of Ms-cyclam(OBz)$_3$, $CDCl_3$, 298K

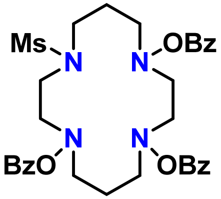

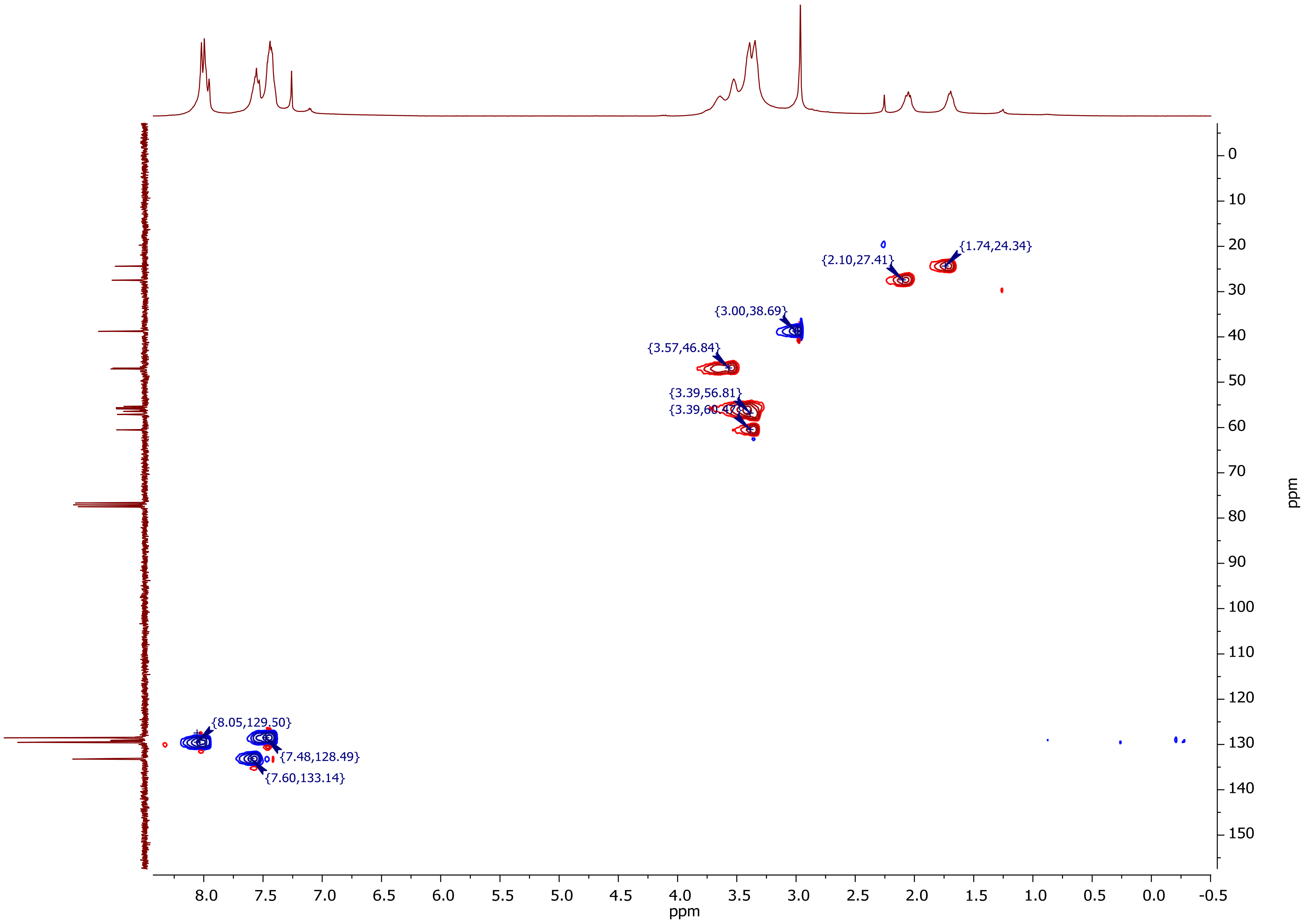

^{1}H NMR of EtC(O)-cyclen(OBz)$_3$, $CDCl_3$, 298K

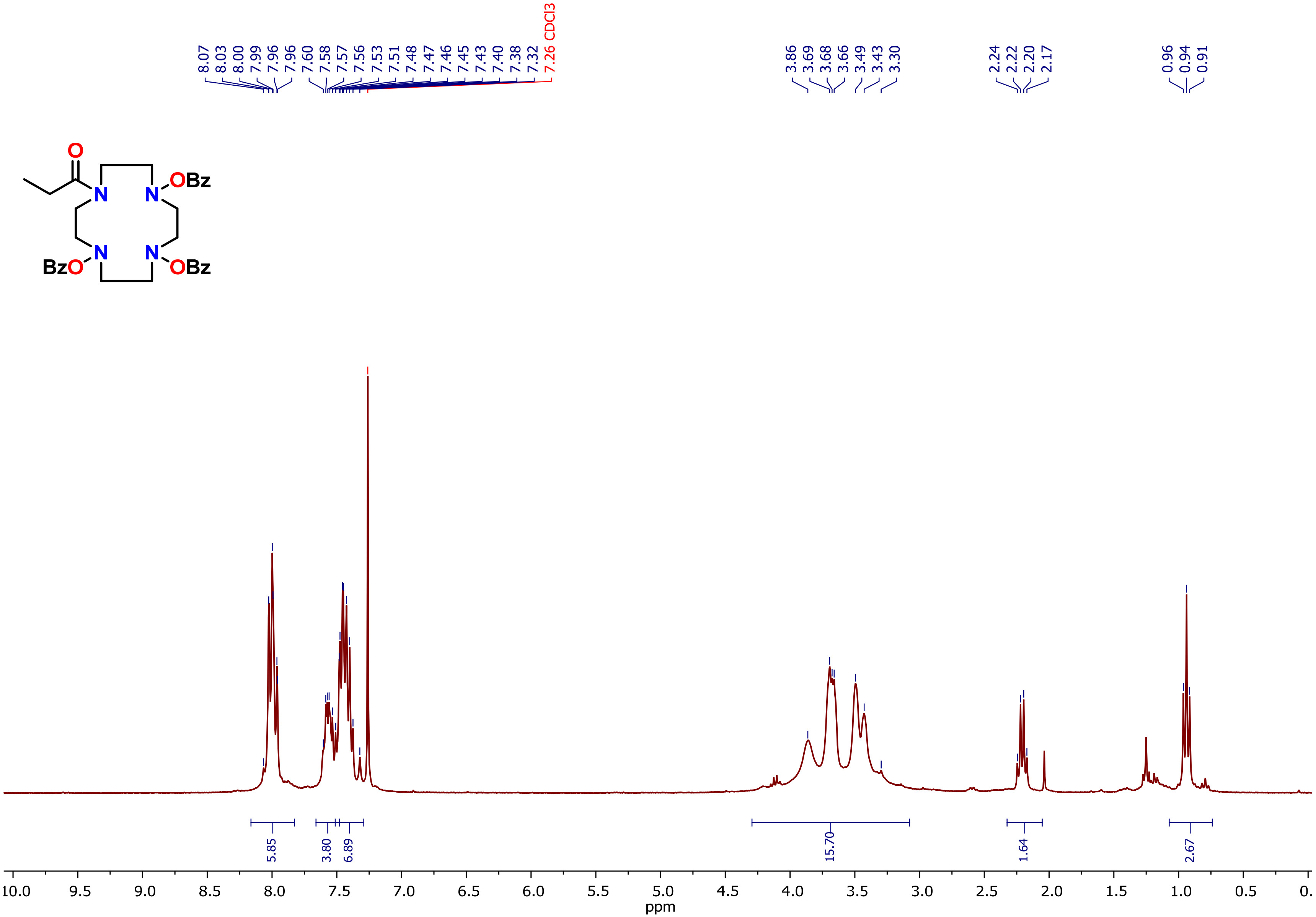

^{13}C NMR of EtC(O)-cyclen(OBz)$_3$, CDCl$_3$, 298K

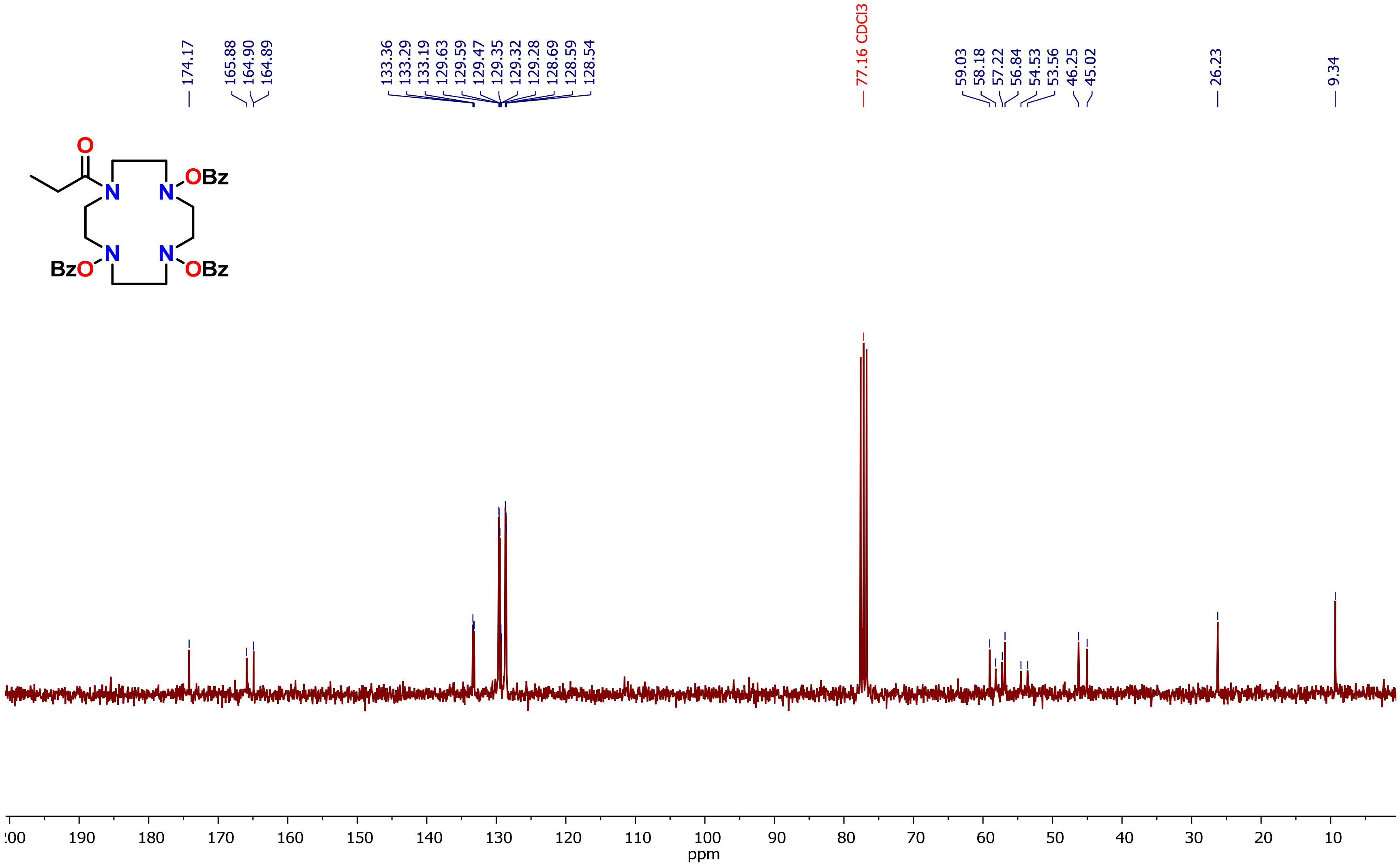

^{1}H-^{13}C HSQC of EtC(O)-cyclen(OBz)$_3$, $CDCl_3$, 298K

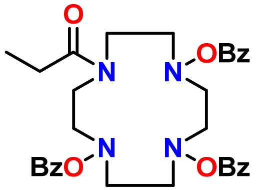

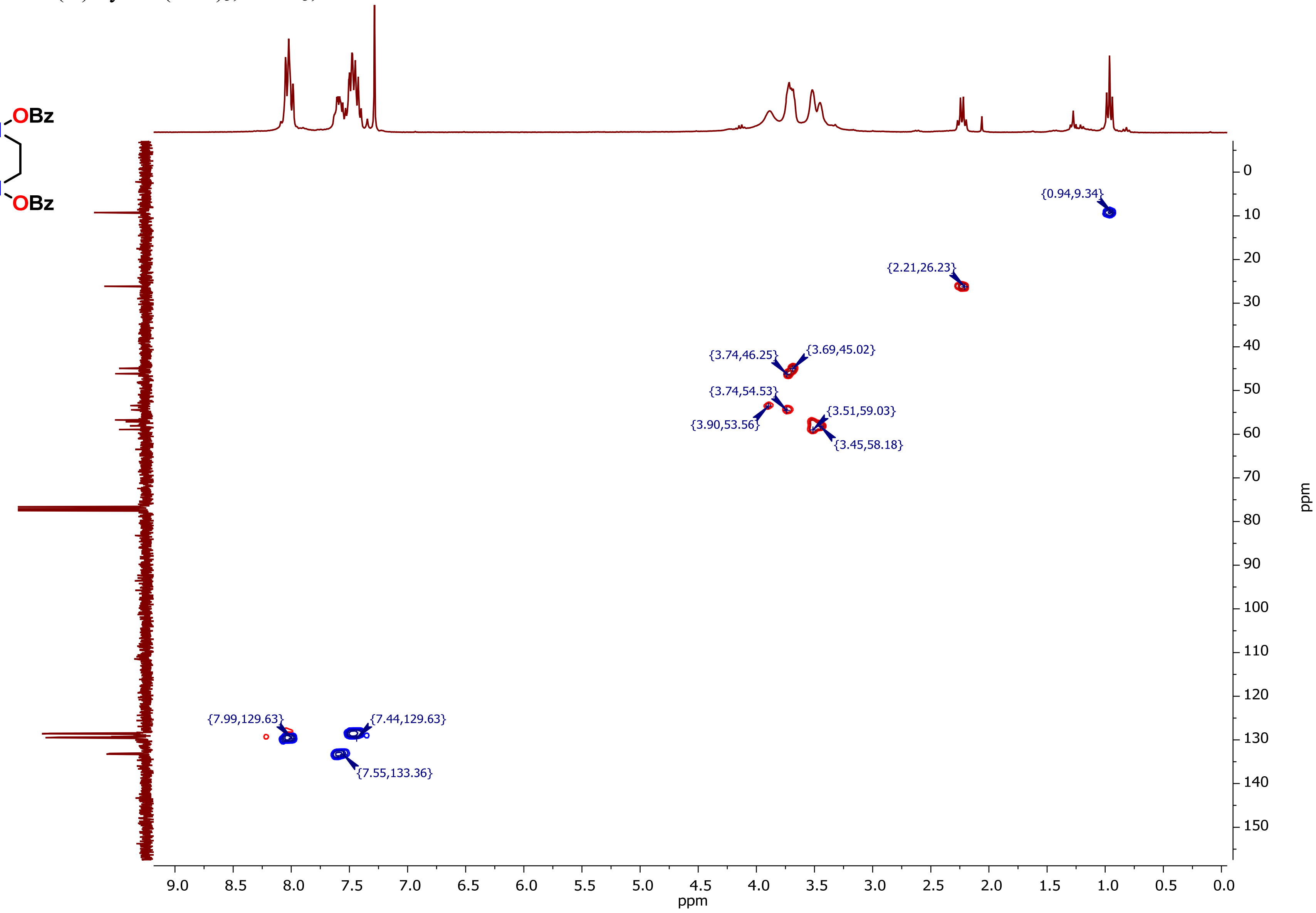

^{1}H NMR of [15]-ane[NOBz]$_4$, $CDCl_3$, 298K

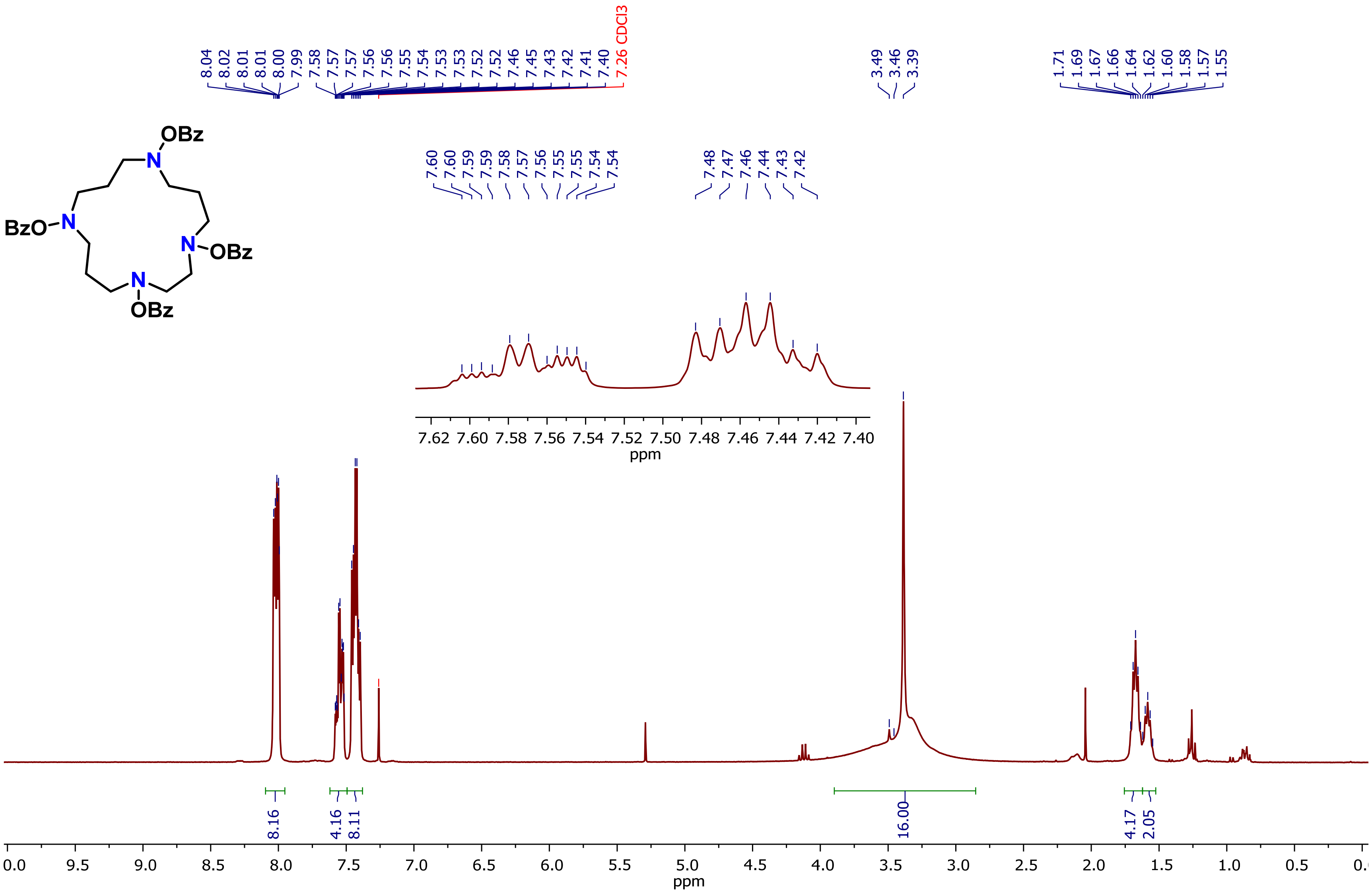

^{13}C NMR of [15]-ane[NOBz]$_4$, $CDCl_3$, 298K

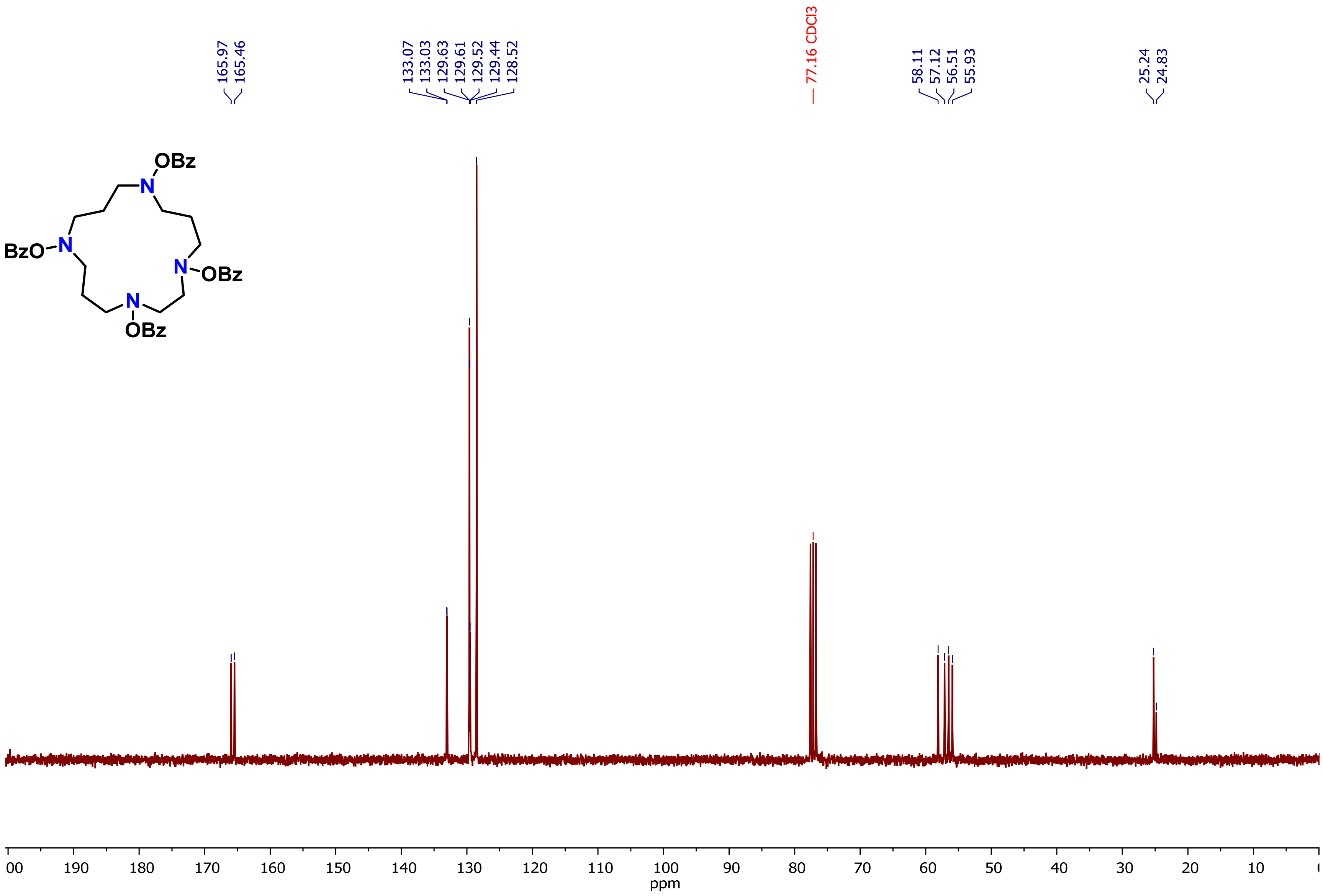

^1H NMR of [20]-ane[NOBz]$_5$, $CDCl_3$, 300K

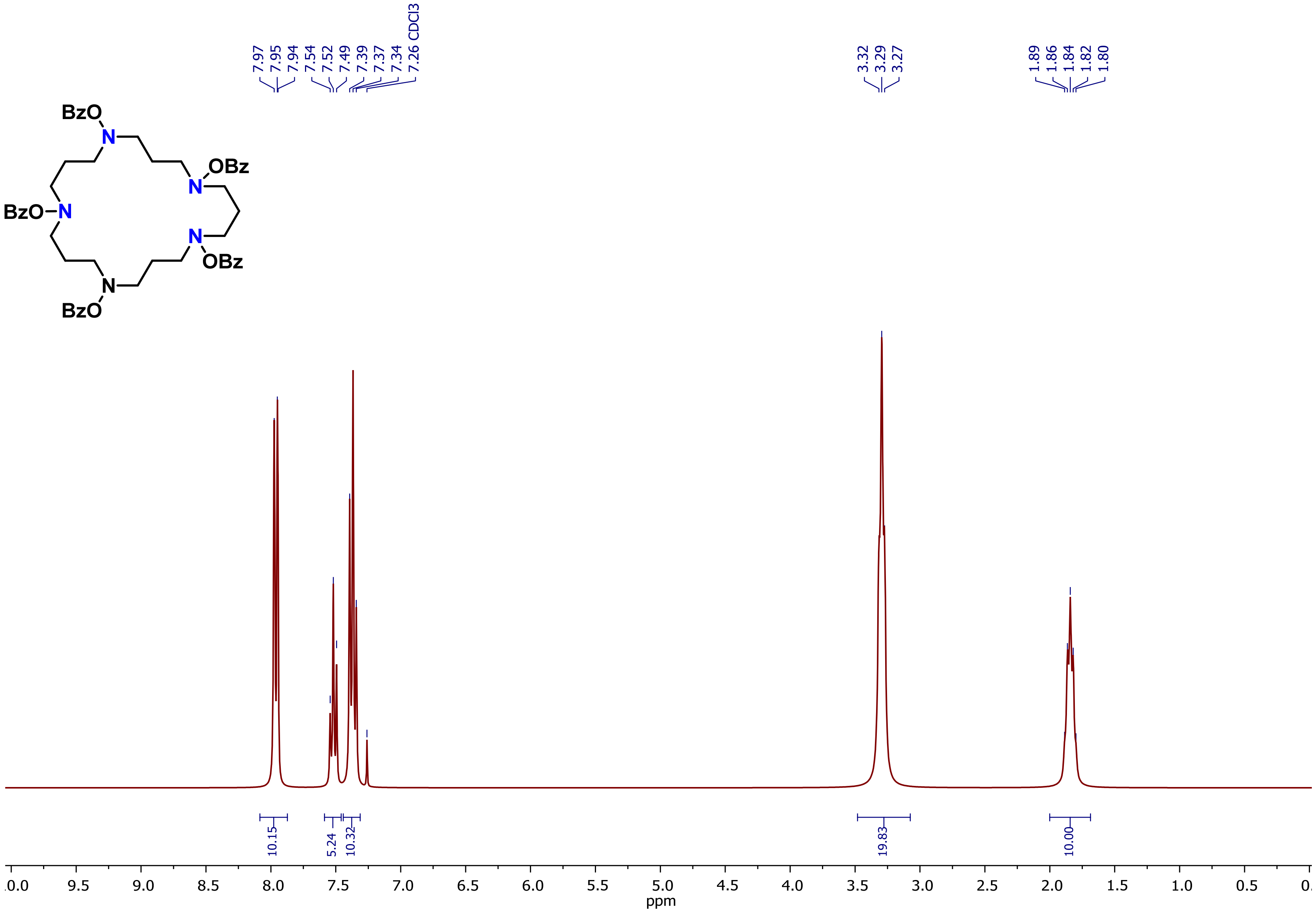

^{13}C NMR of [20]-ane[NOBz]$_5$, $CDCl_3$, 300K

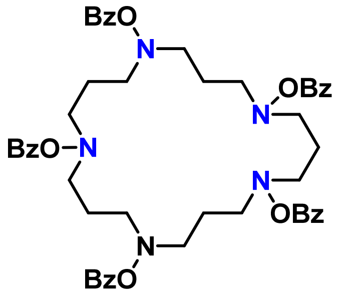

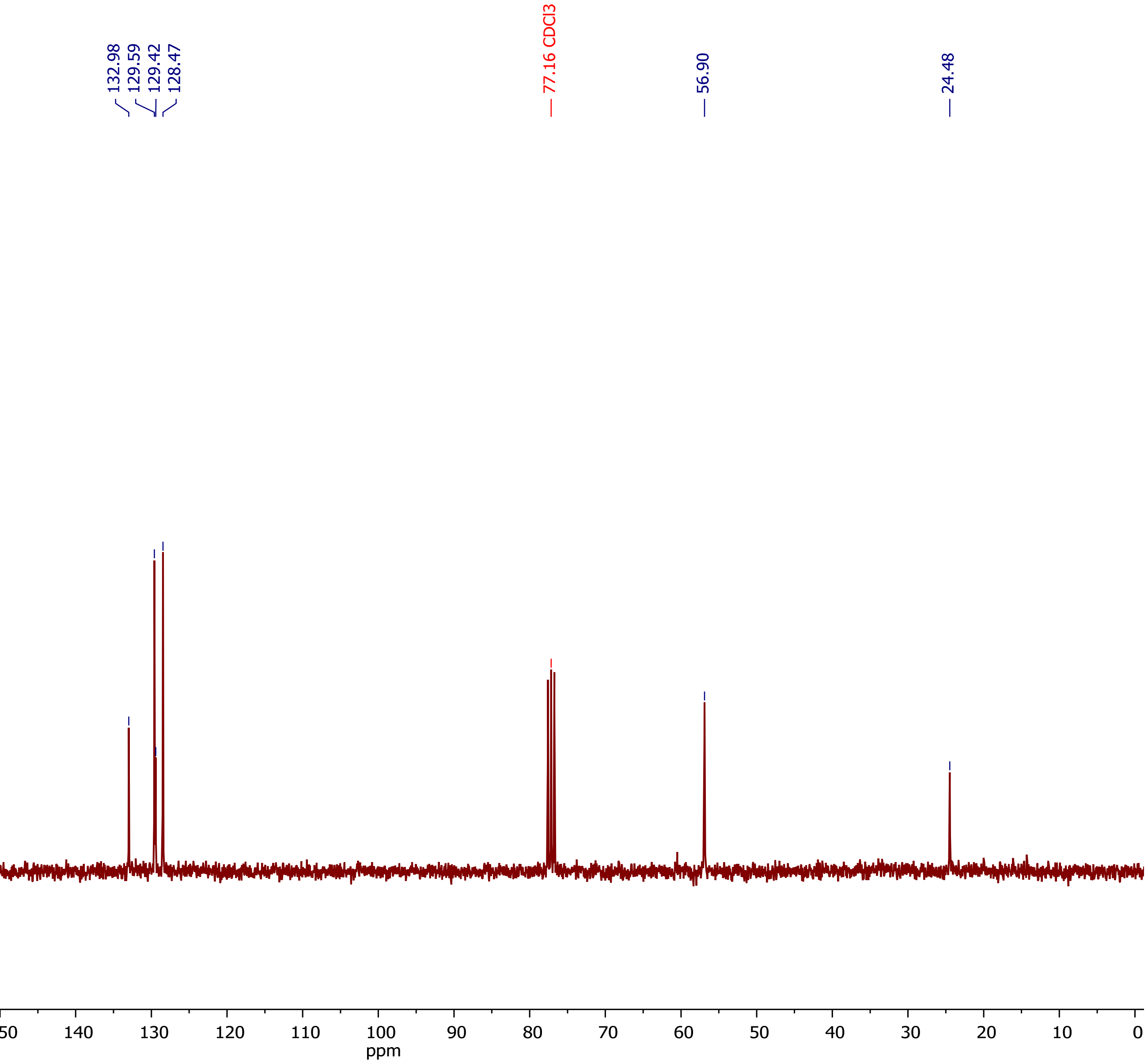

^{1}H NMR of tacn(OH)$_3$, D_2O, 298K

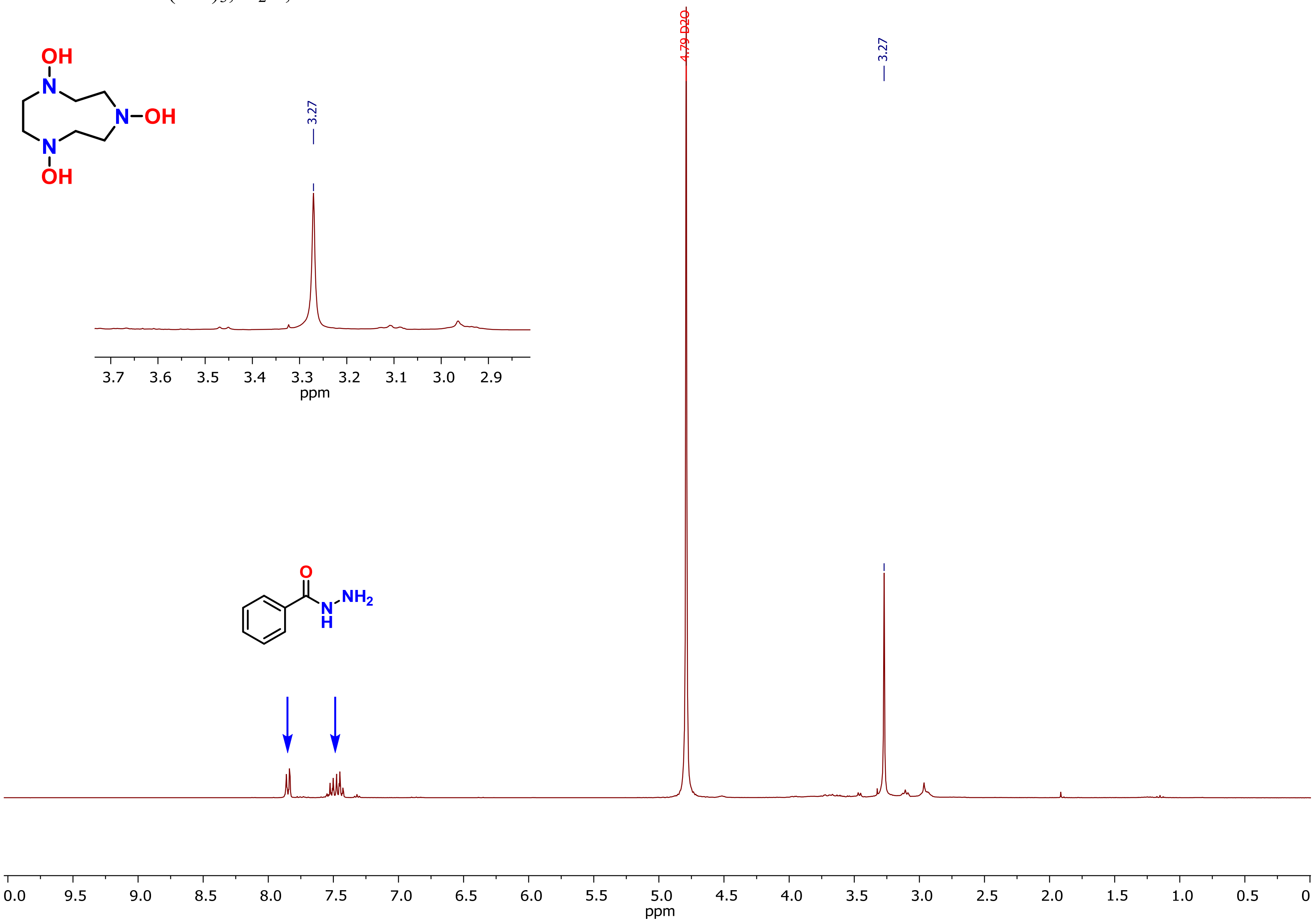

^{13}C NMR of tacn(OH)$_3$, D_2O, 298K

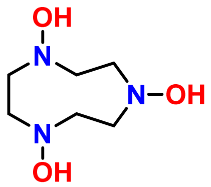

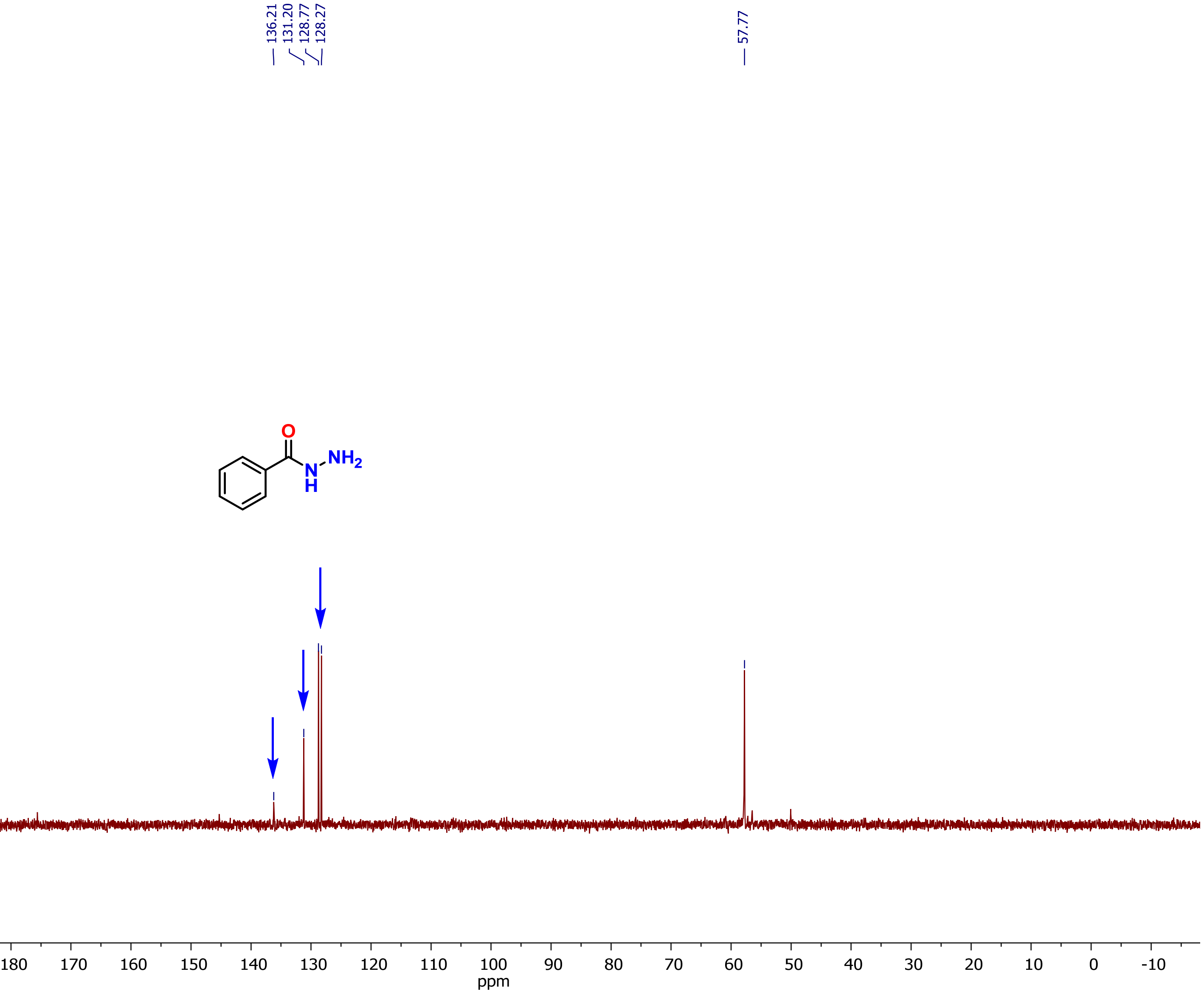

^{1}H-^{13}C HSQC of tacn(OH)$_3$, D_2O, 298K

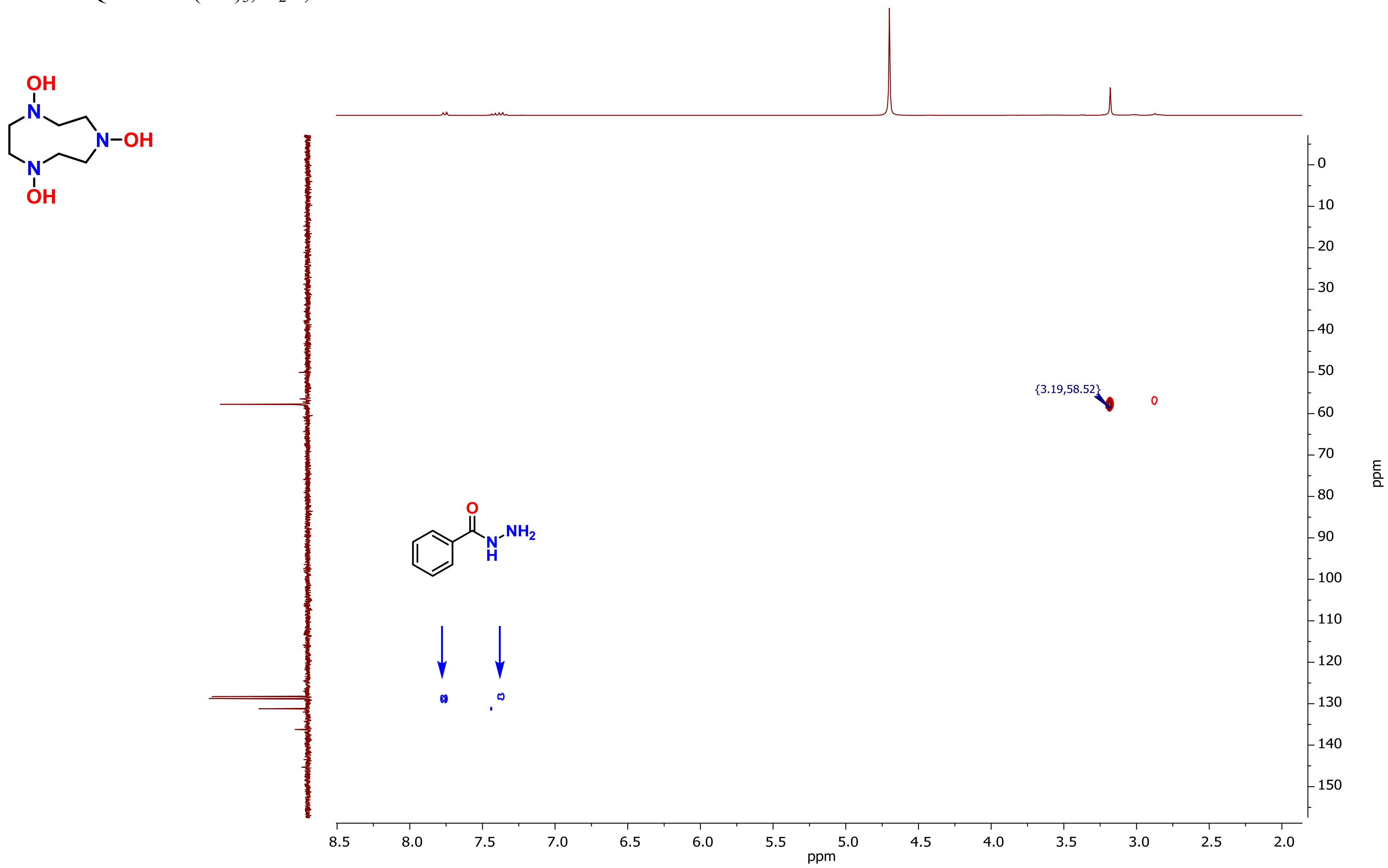

^{1}H NMR of cyclam$(OH)_4$, D_2O, 298K

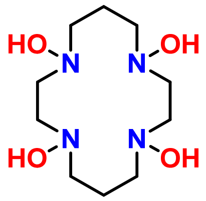

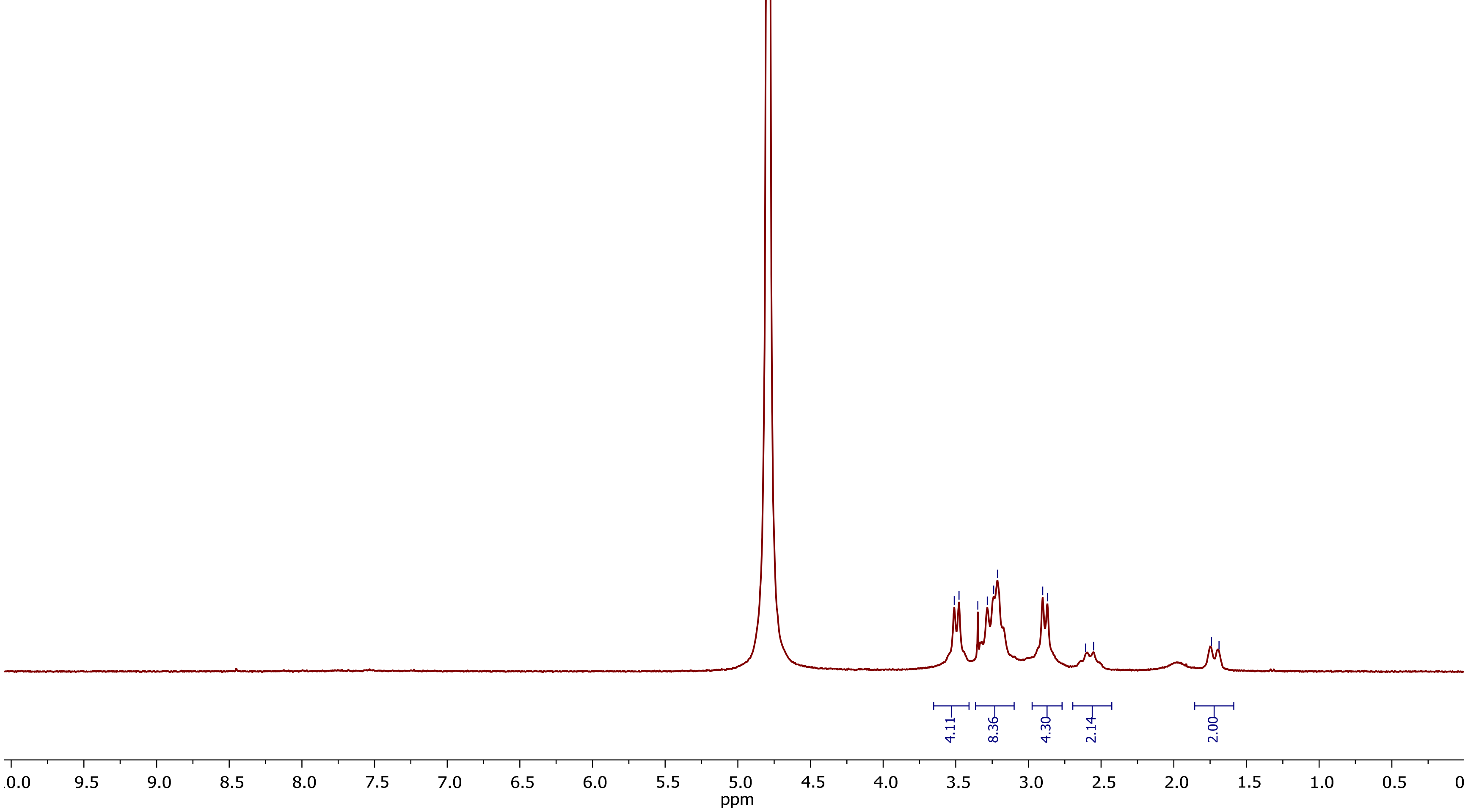

^{13}C NMR of cyclam(OH)$_4$, D_2O, 298K

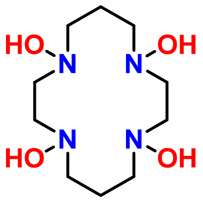

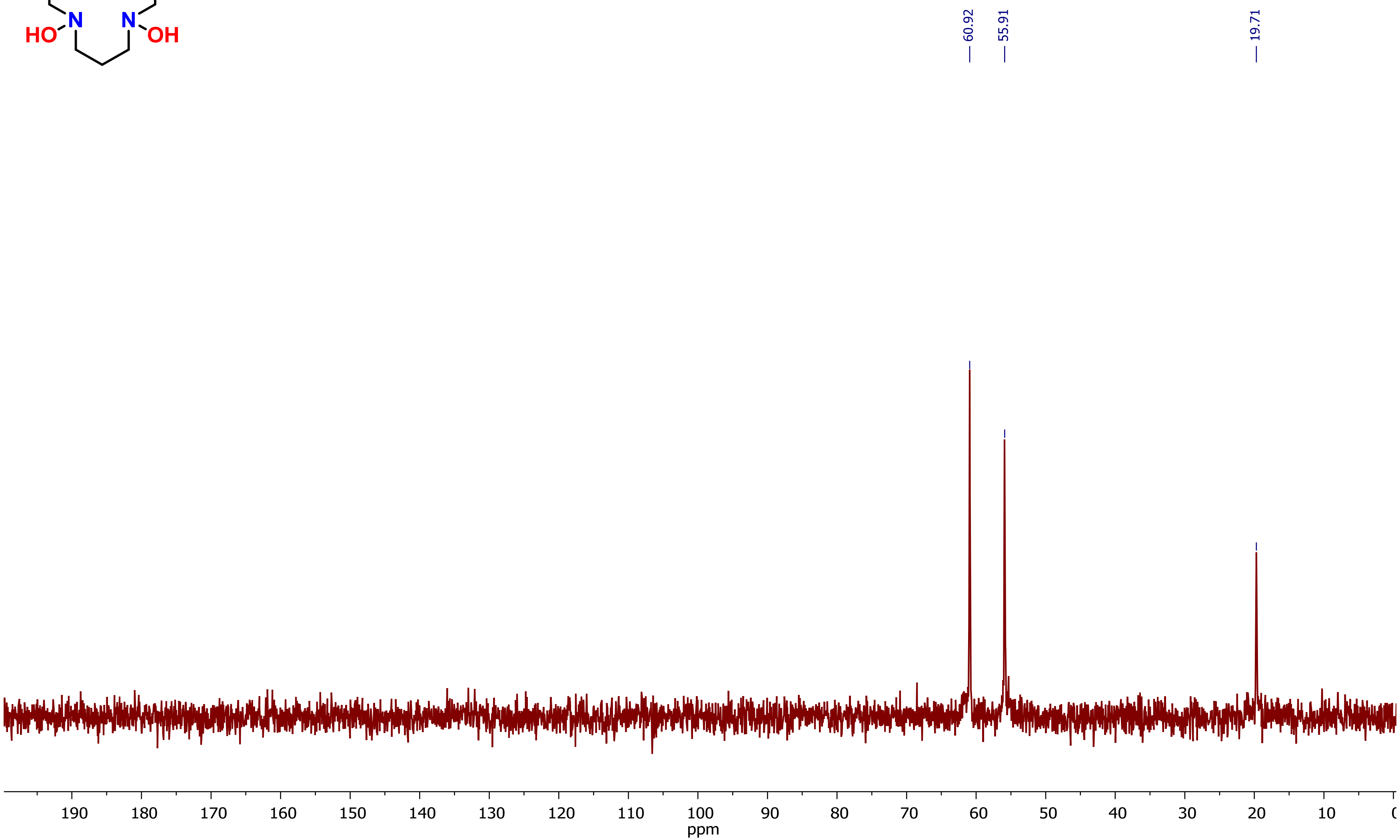

^{1}H-^{1}H COSY of cyclam(OH)$_4$, D_2O, 298K

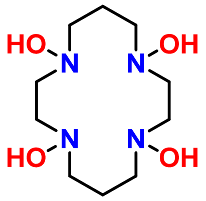

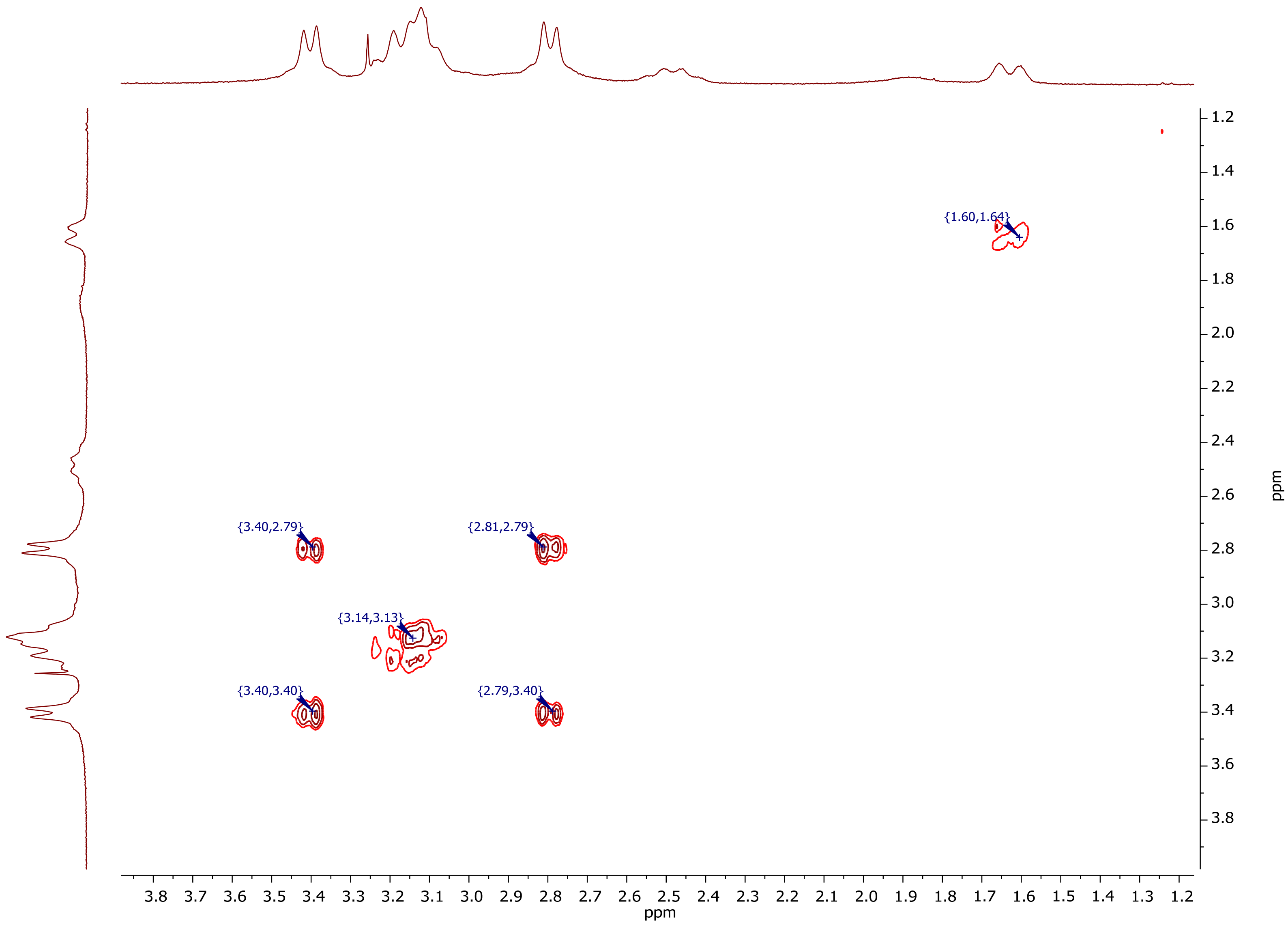

^{1}H-^{13}C HSQC of cyclam(OH)$_4$, D_2O, 298K

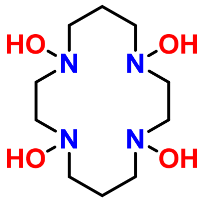

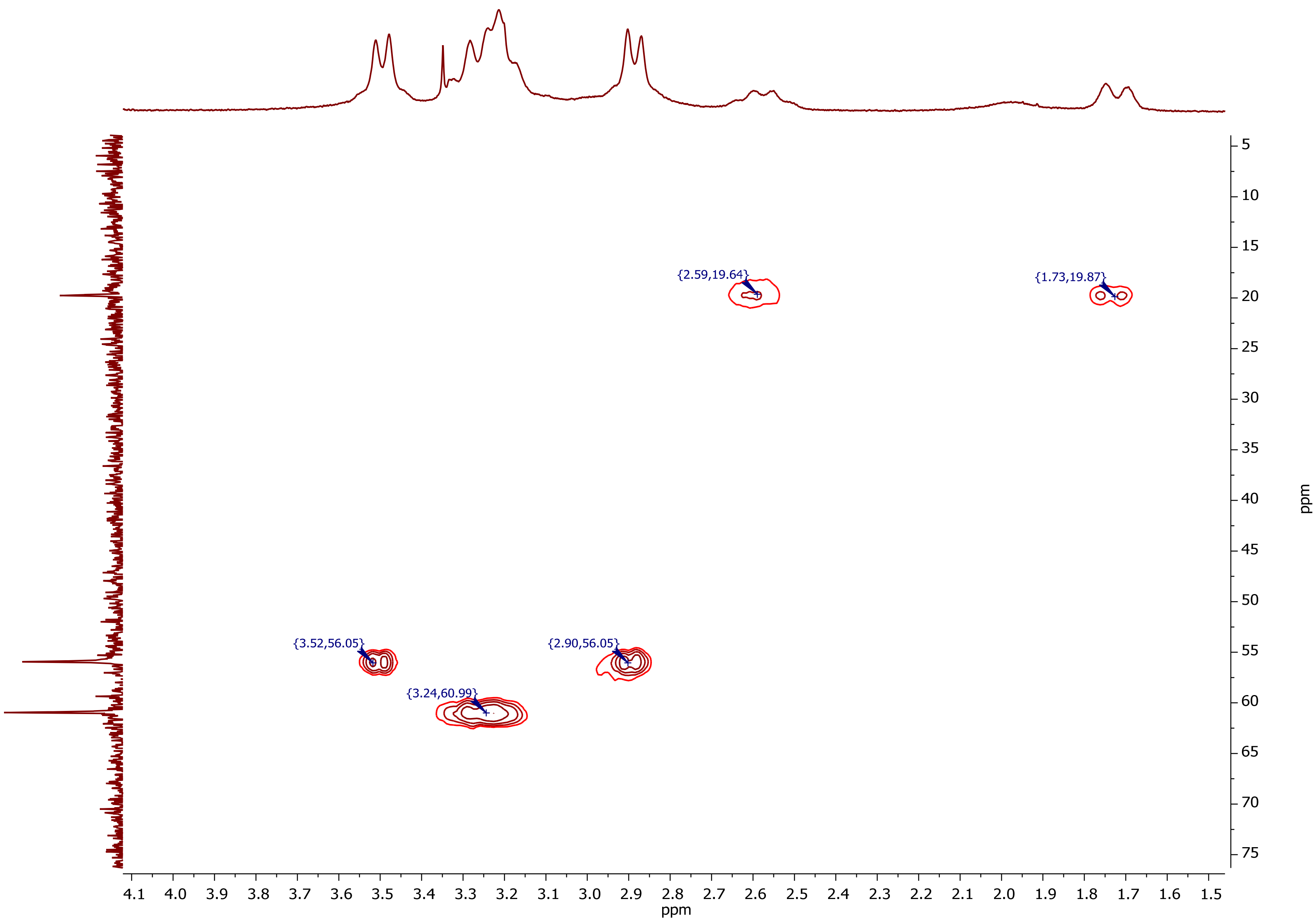

FT-IR of cyclam(OH)$_4$, KBr

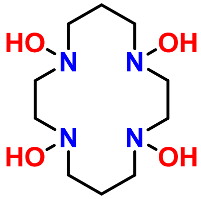

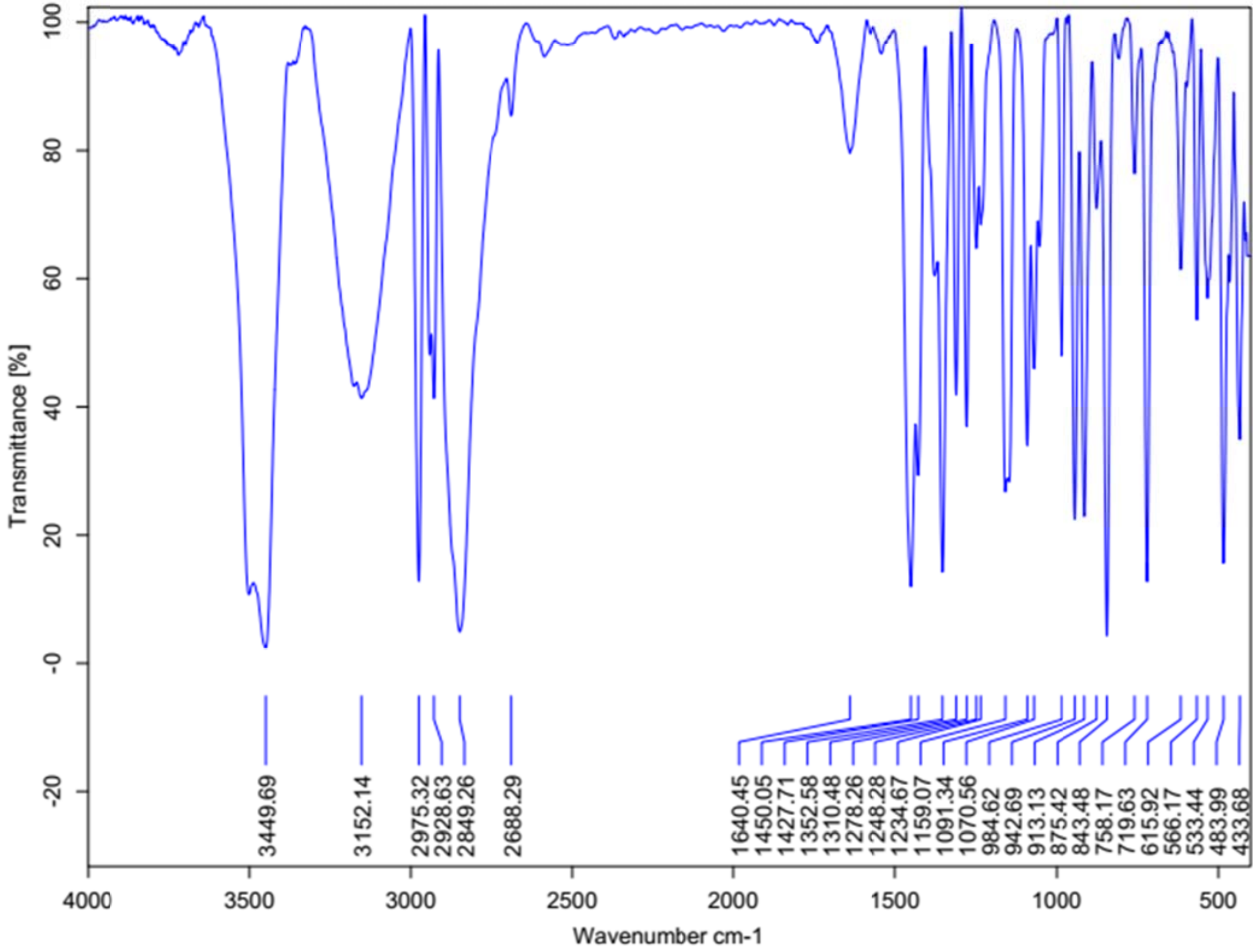

^1H NMR of cyclam(OH)$_4$•HCl, D_2O, 298K

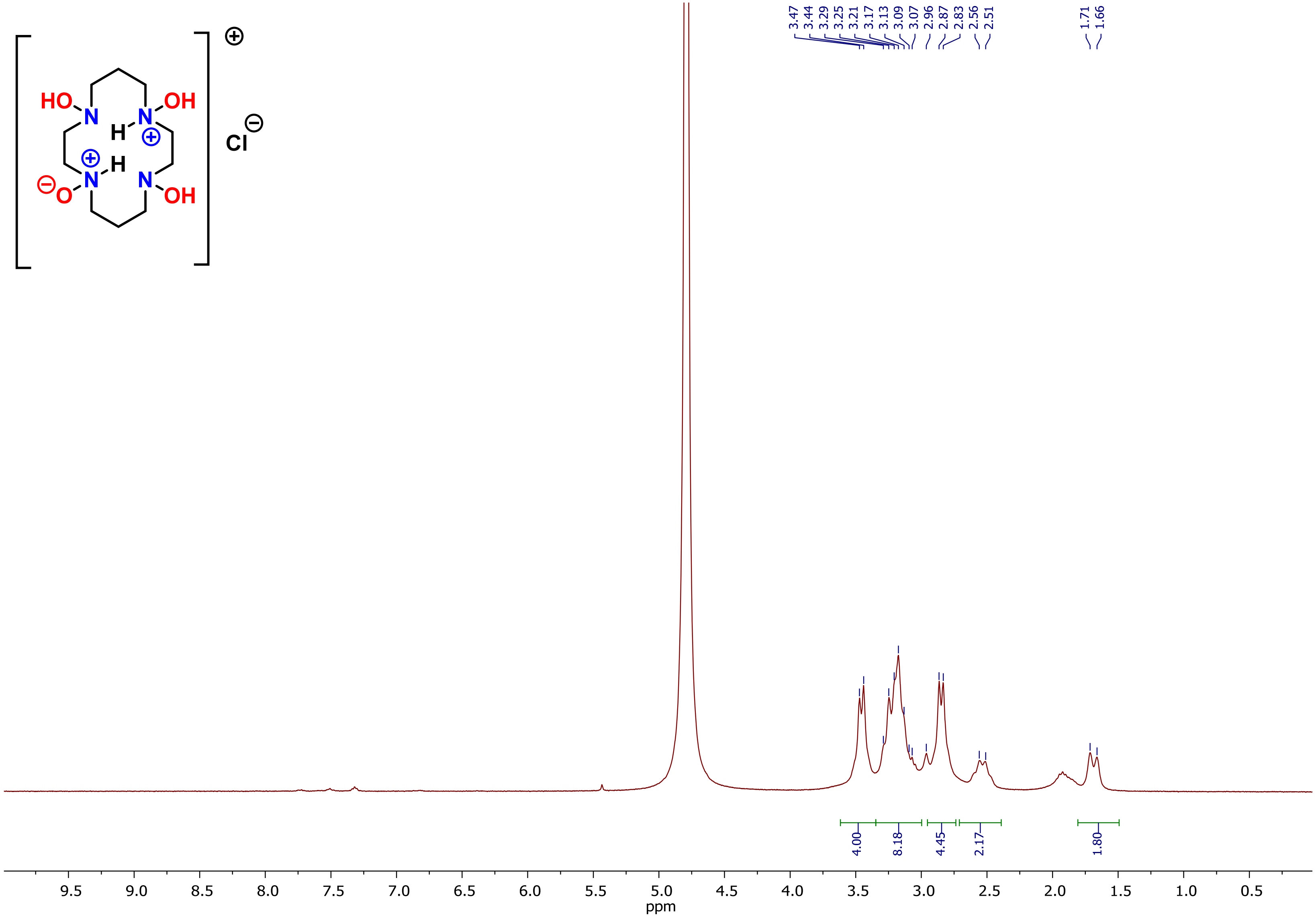

^{1}H-^{13}C HSQC of cyclam(OH)$_4$•HCl, D_2O, 298K

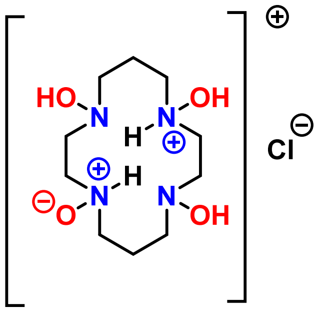

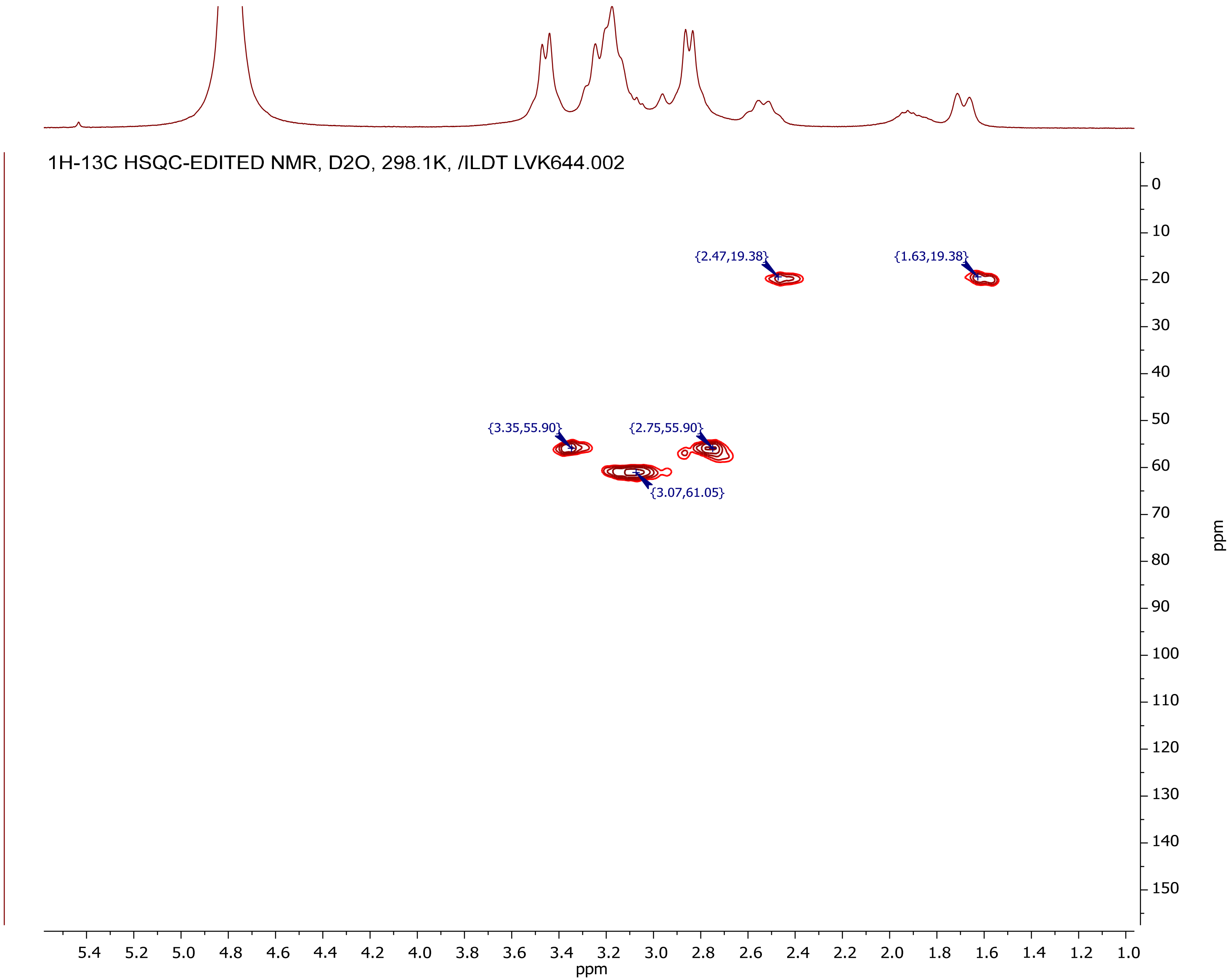

FT-IR of cyclam(OH)$_4$•HCl, KBr

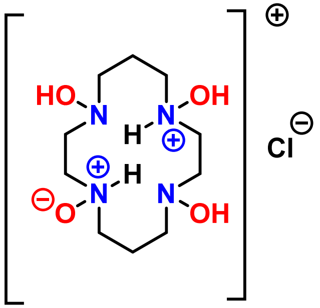

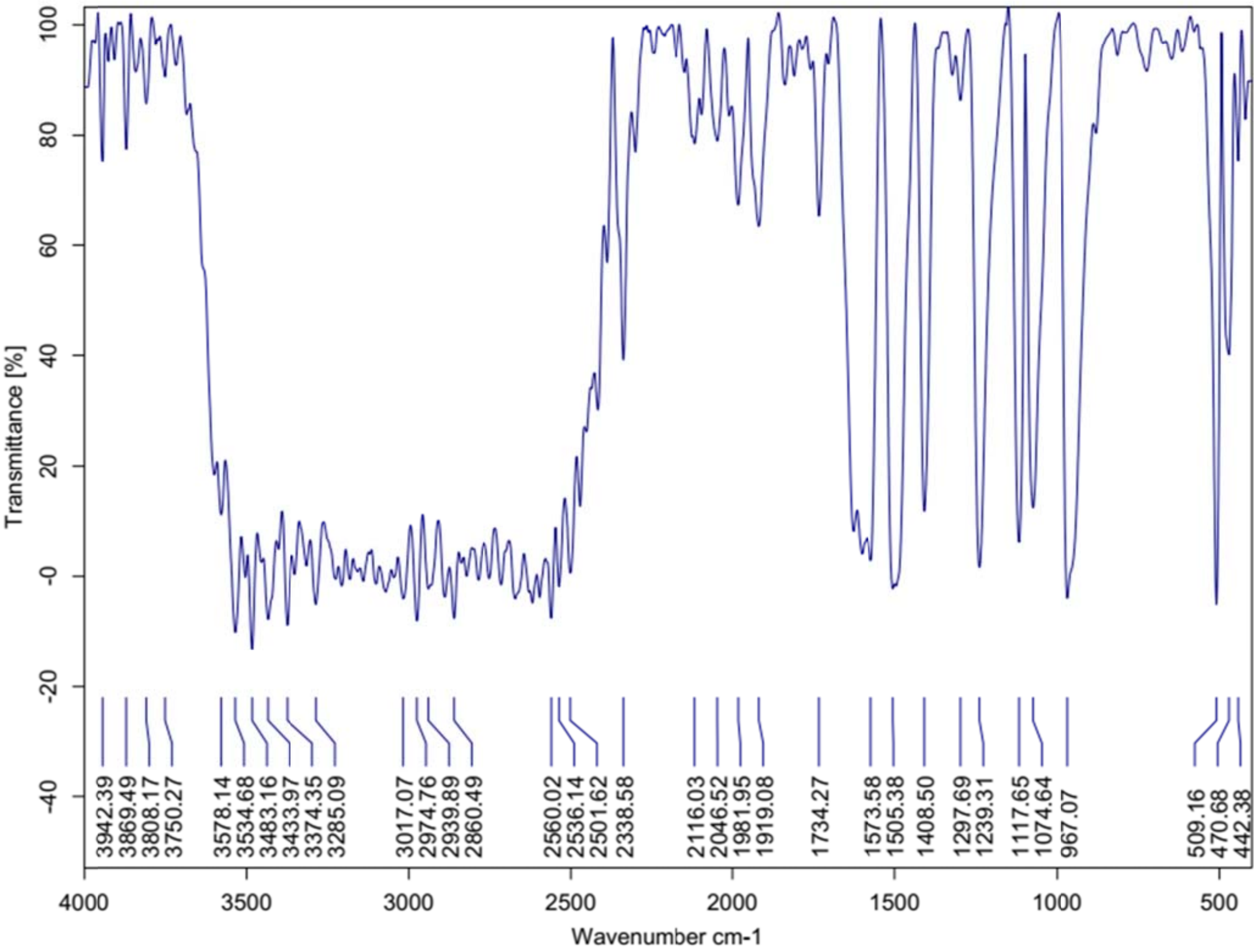

^{1}H NMR of [20]-ane[NOH]$_5$, D_2O, 298K

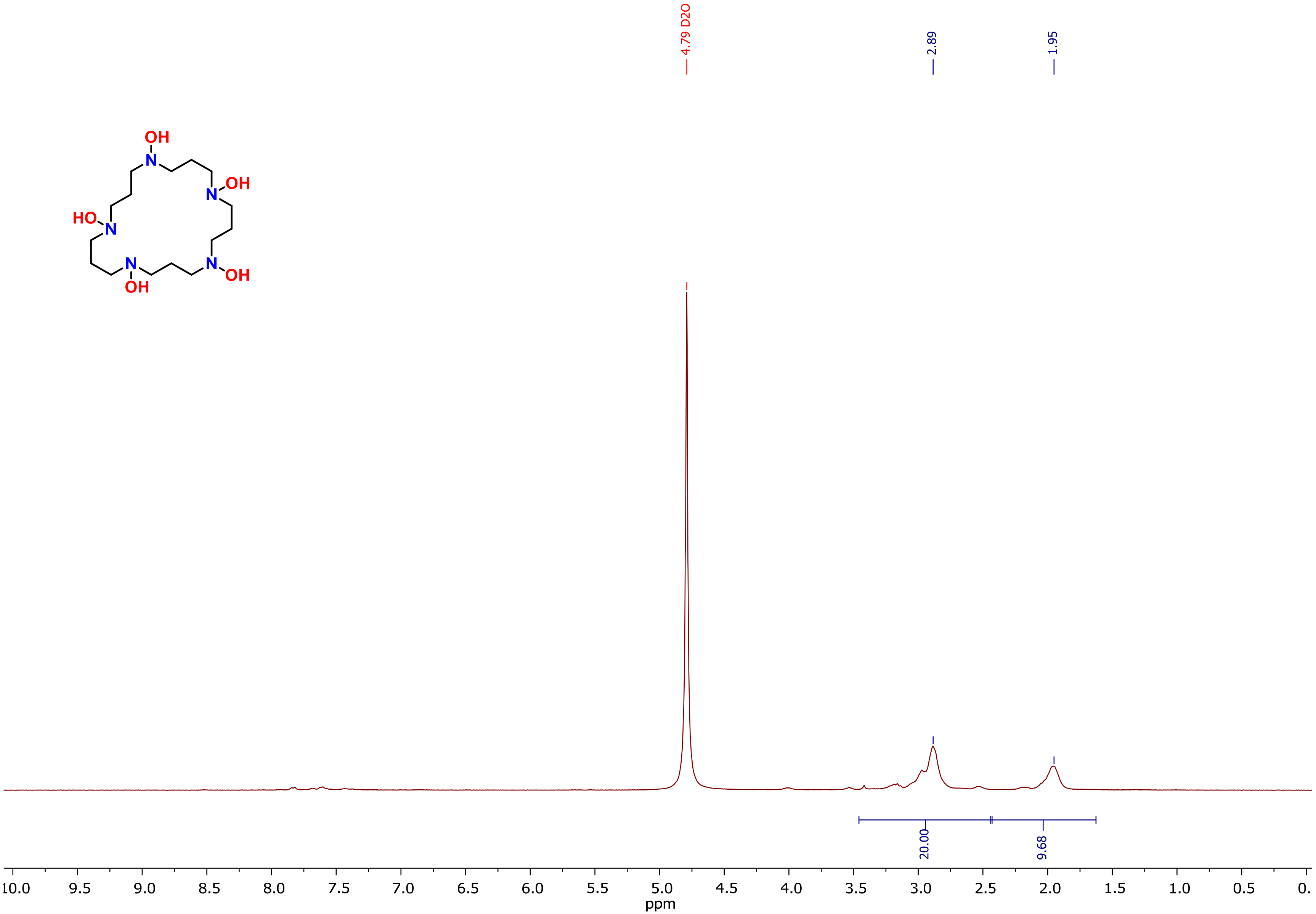

^{13}C NMR of [20]-ane[NOH]$_5$, D_2O, 298K

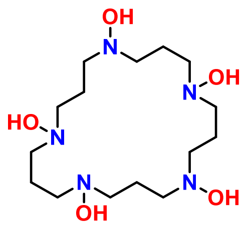

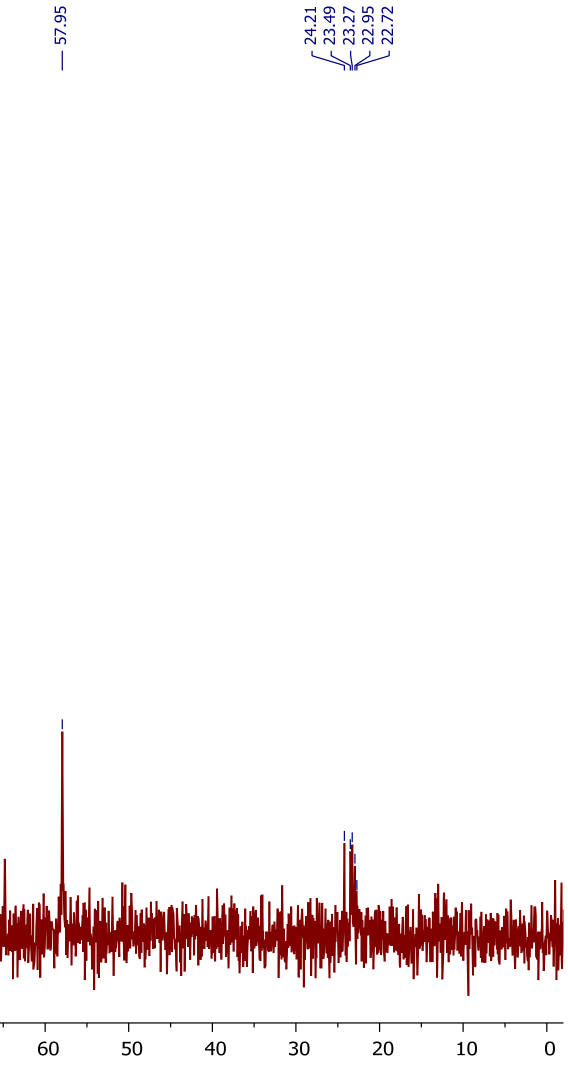

^{1}H-^{13}C HSQC of [20]-ane[NOH]$_5$, D_2O, 298K

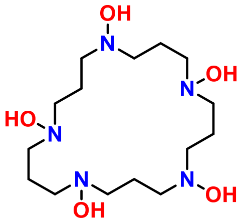

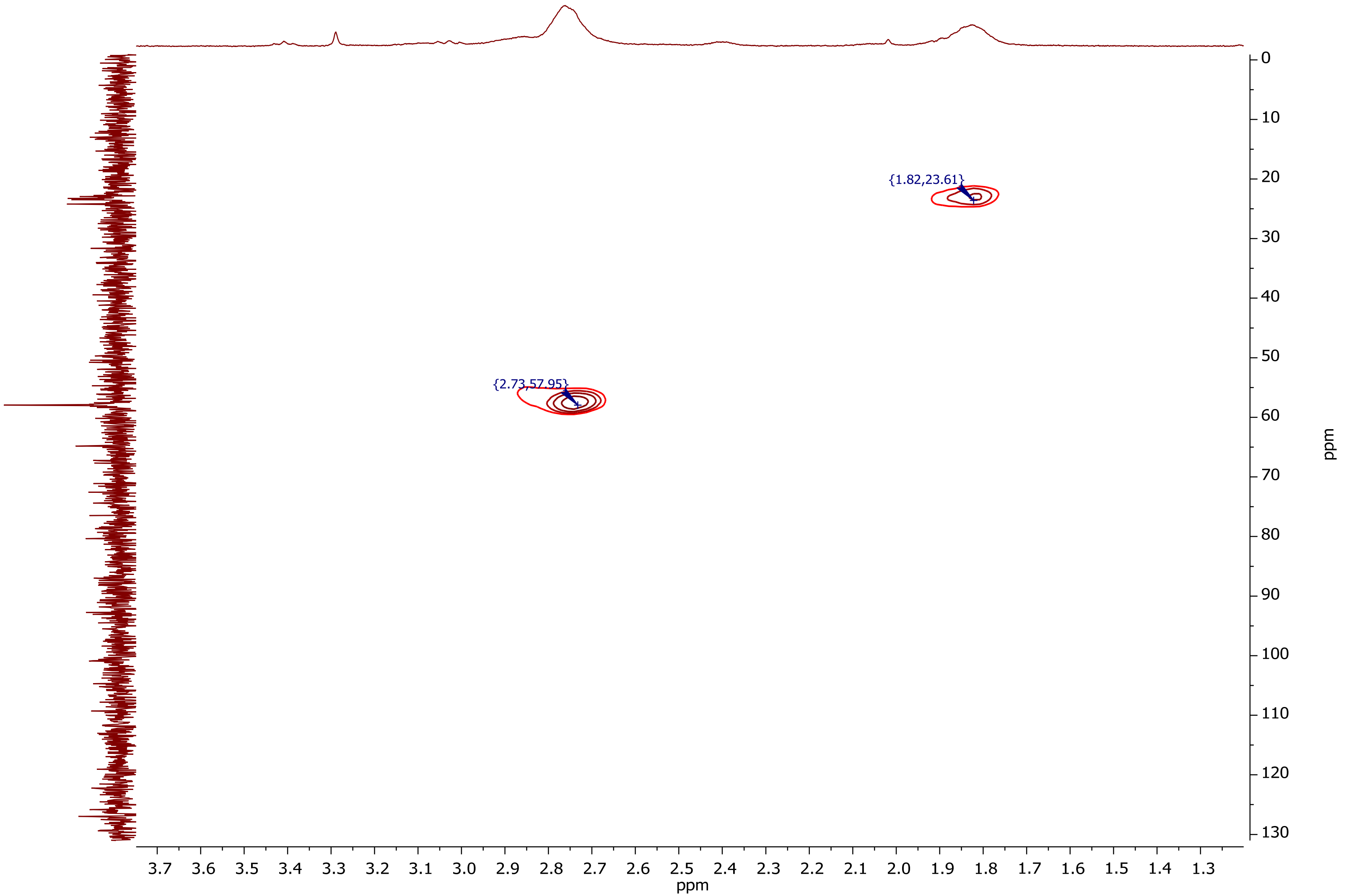

FT-IR of [20]-ane[NOH]$_5$, KBr

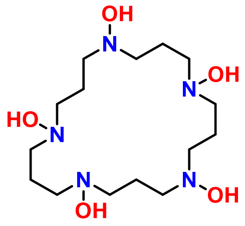

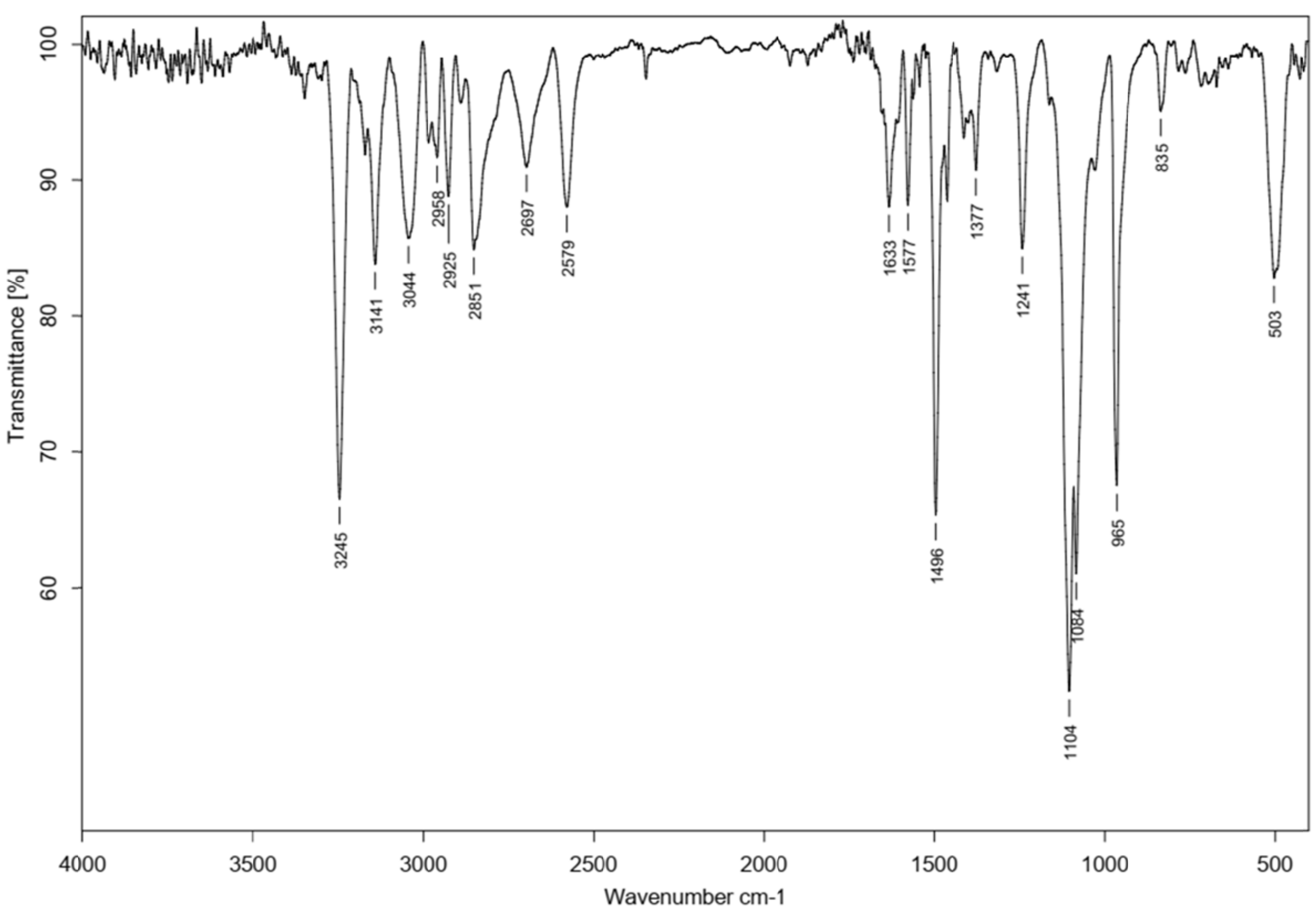

^{1}H NMR of $[Zn(tacn(OH)_3)_2](NO_3)_2$•$2H_2O$•$^1/_6PhCO_2{}^iPr$, D_2O, 298K

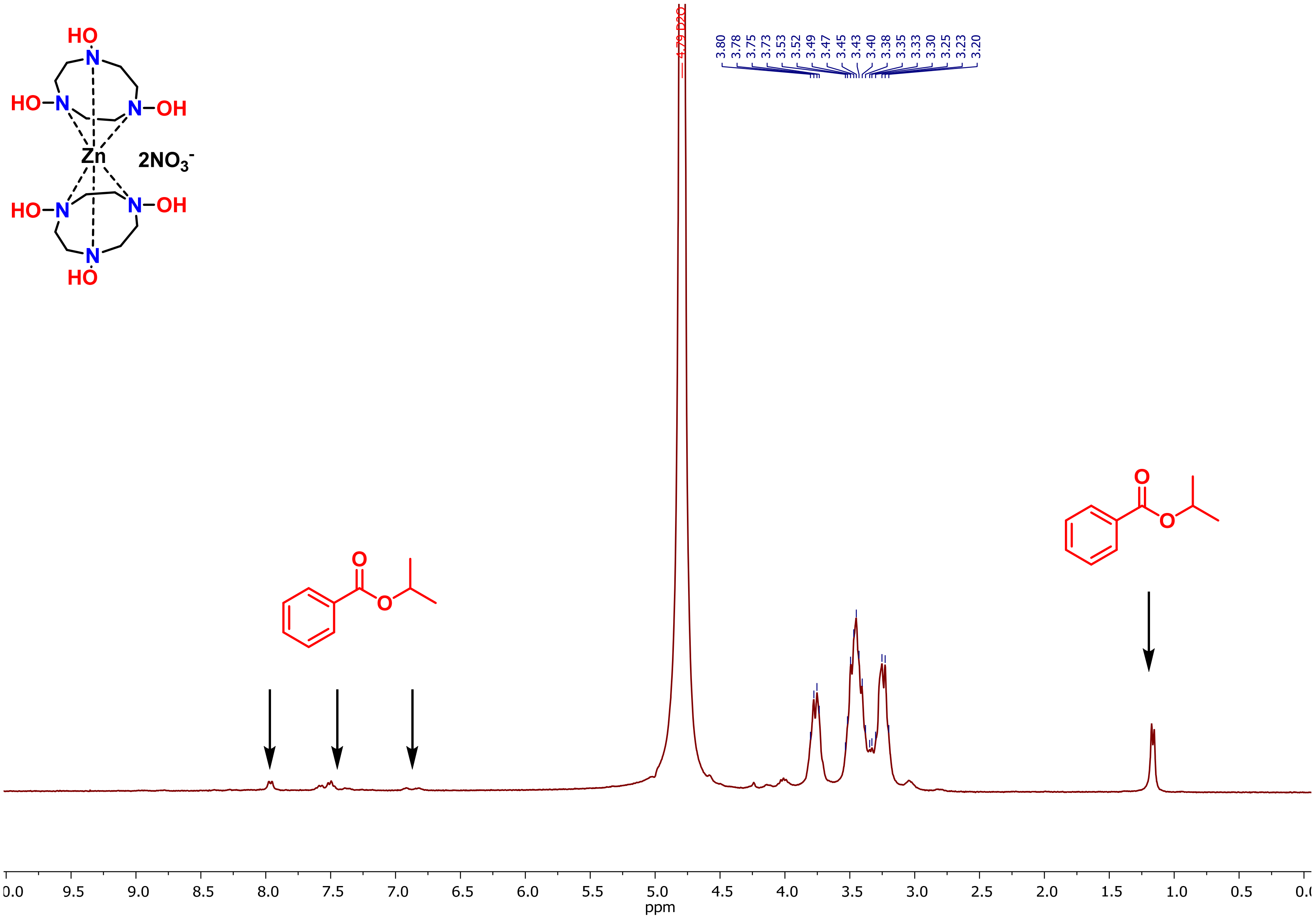

^{13}C NMR of $[Zn(tacn(OH)_3)_2](NO_3)_2 \bullet 2H_2O \bullet {}^1/_6 PhCO_2{}^iPr$, D_2O, 298K

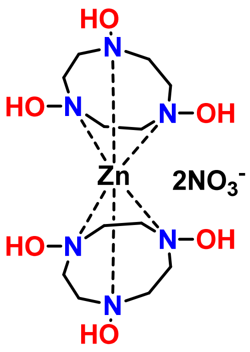

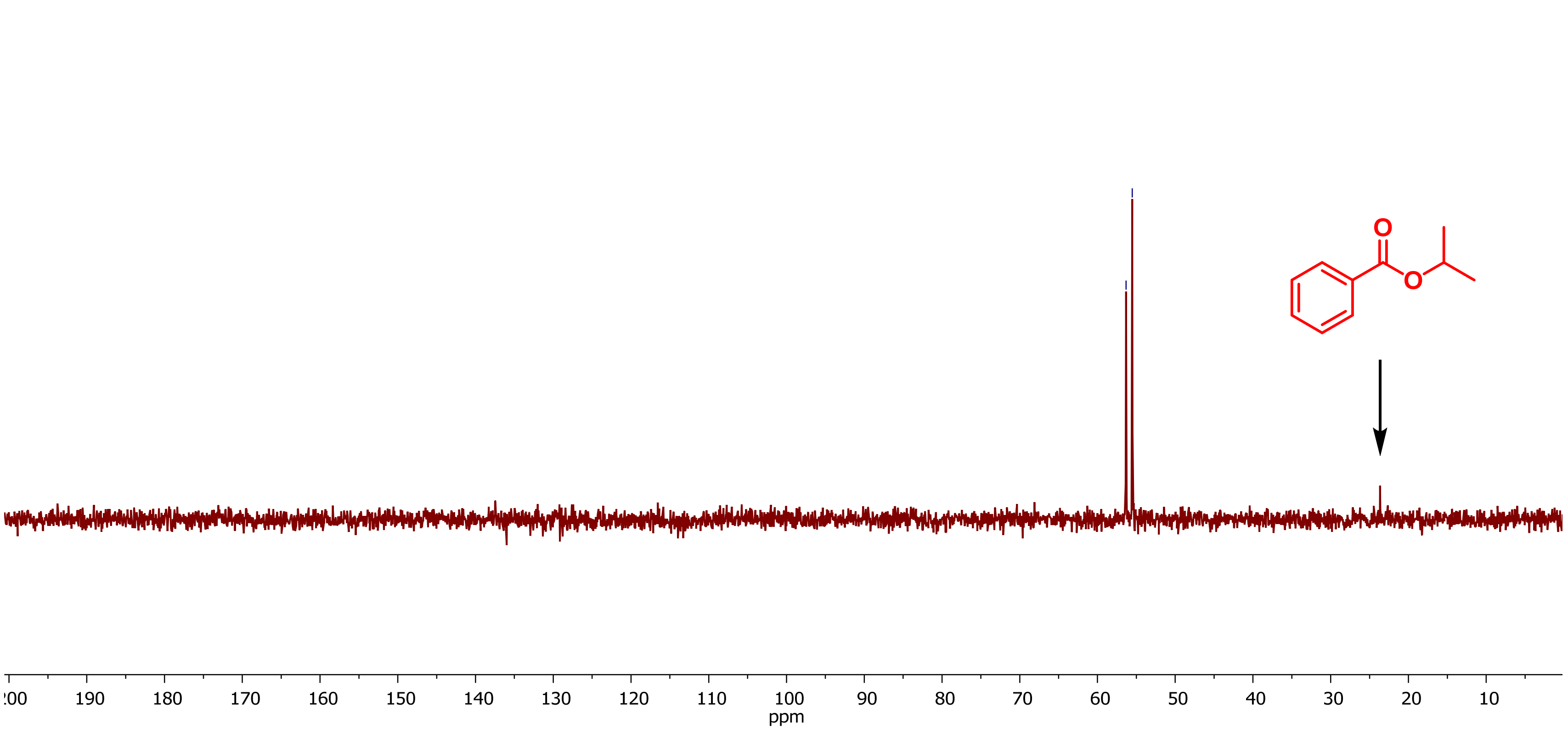

^1H-^{13}C HSQC of $[Zn(tacn(OH)_3)_2](NO_3)_2 \bullet 2H_2O \bullet {}^1/_6 PhCO_2{}^iPr$, D_2O, 298K

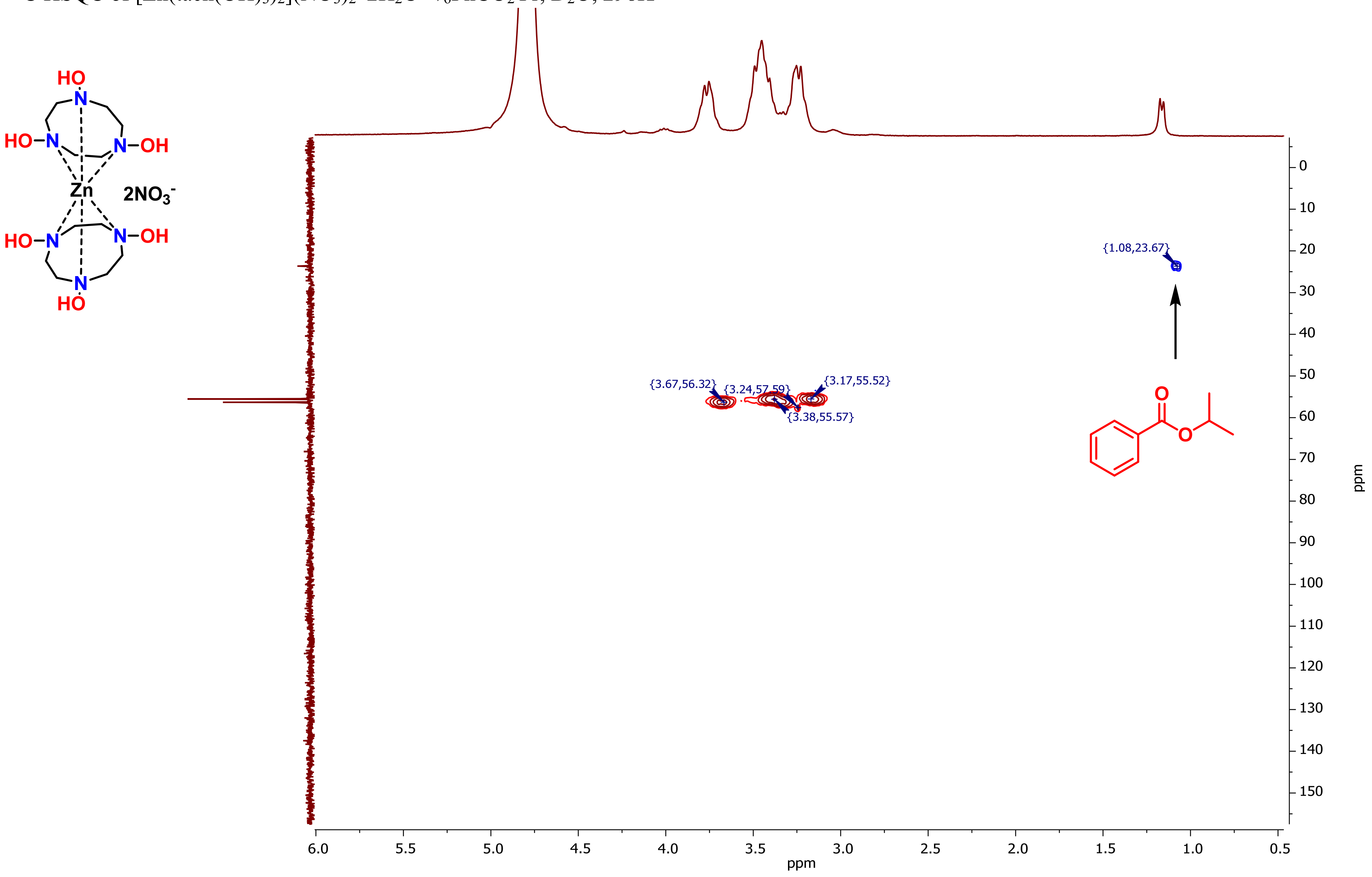

FT-IR of [Zn(tacn(OH)$_3$)$_2$](NO$_3$)$_2$, KBr

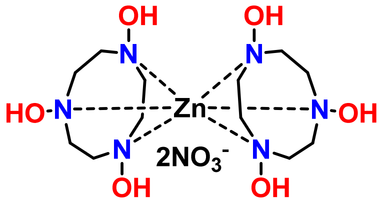

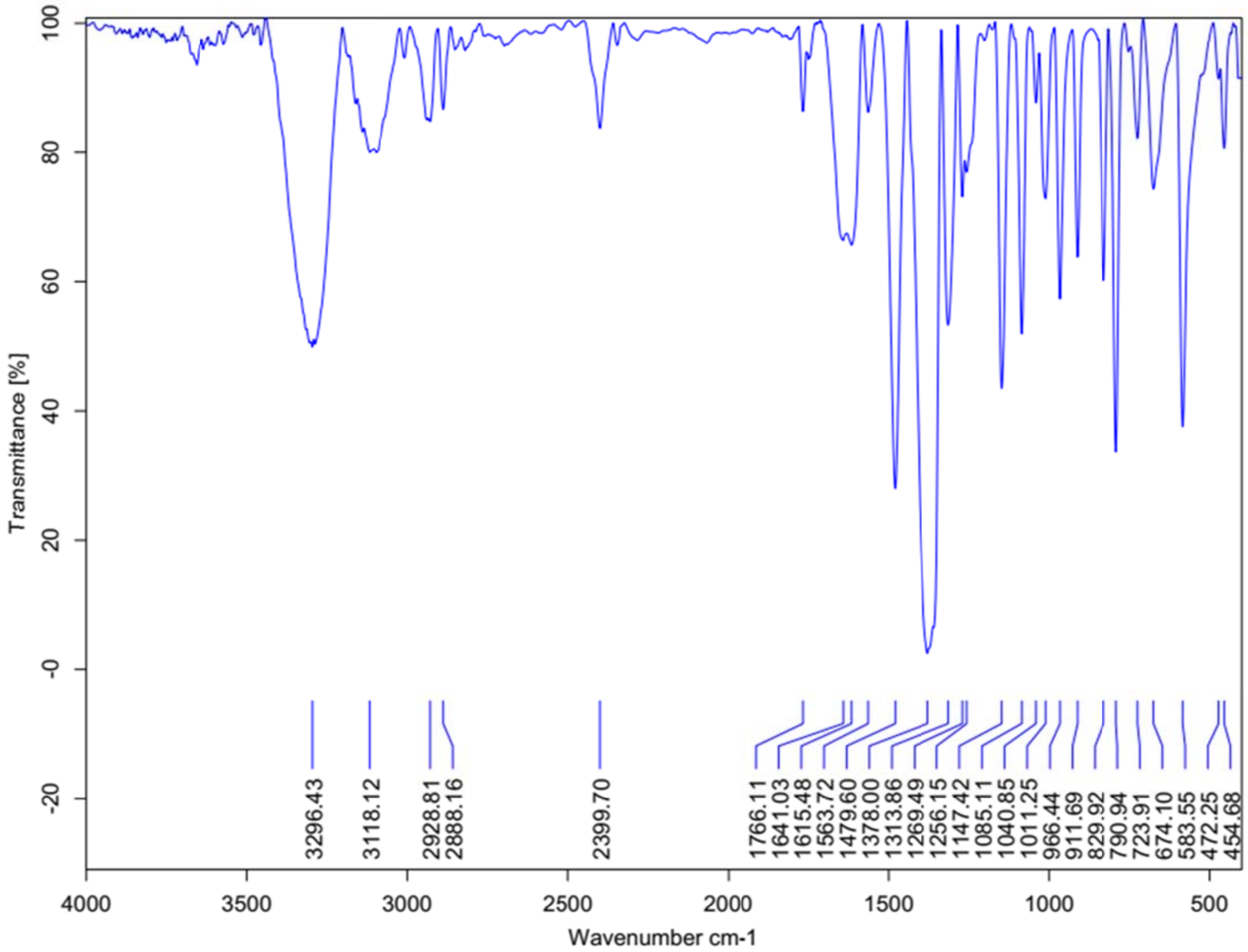

FT-IR of [Ni_2(μ-Cl)(μ-O_2CPh)(tacn$(OH)_3)_2Cl_2$], KBr

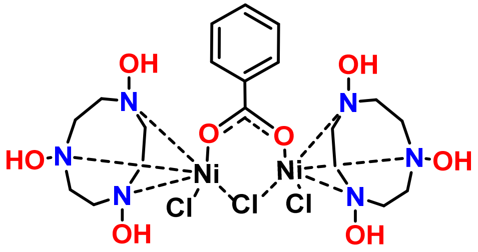

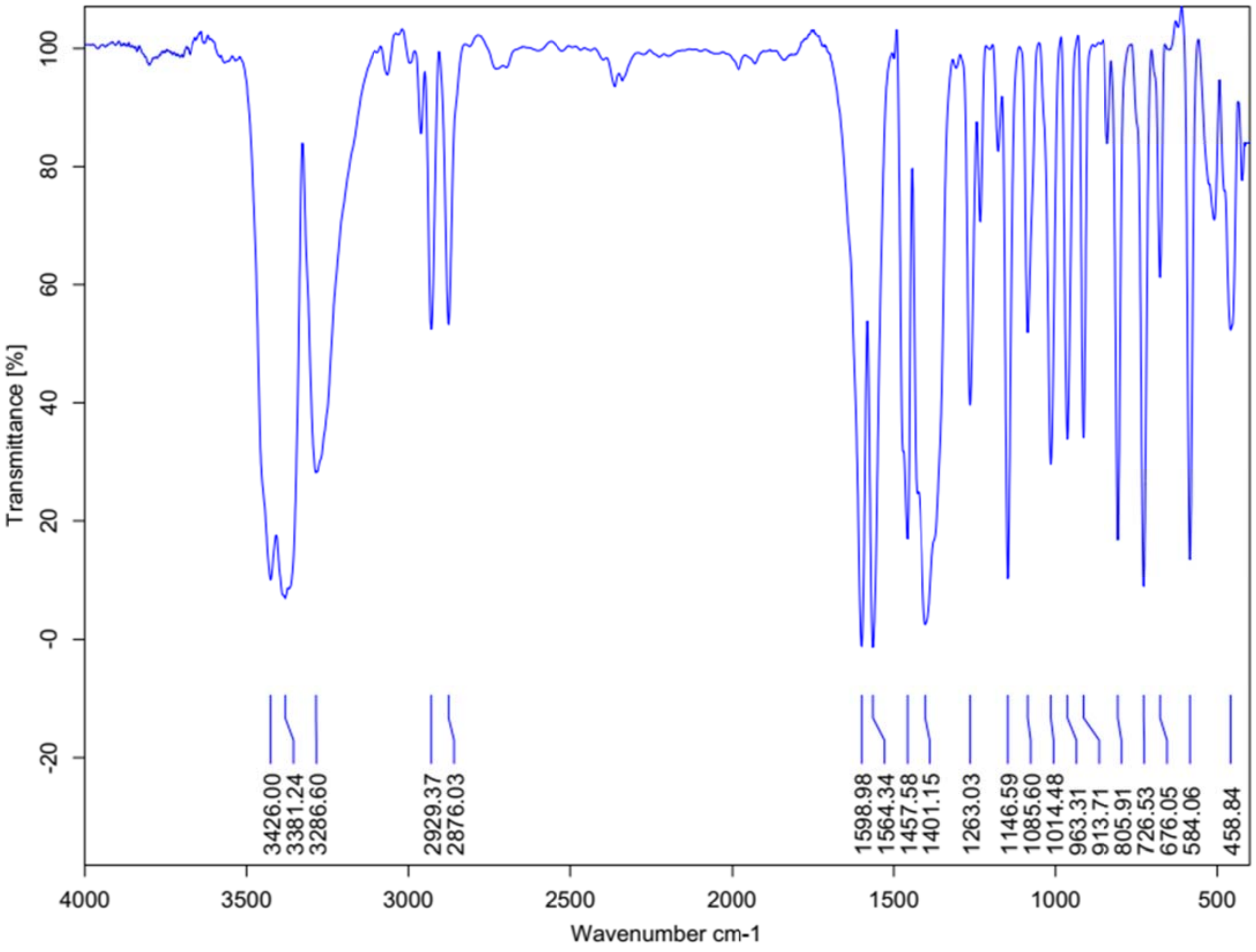

FT-IR of $Cu(cyclam(OH)_4)Cl_2$, KBr

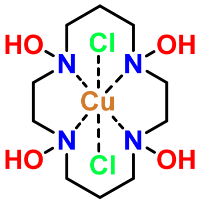

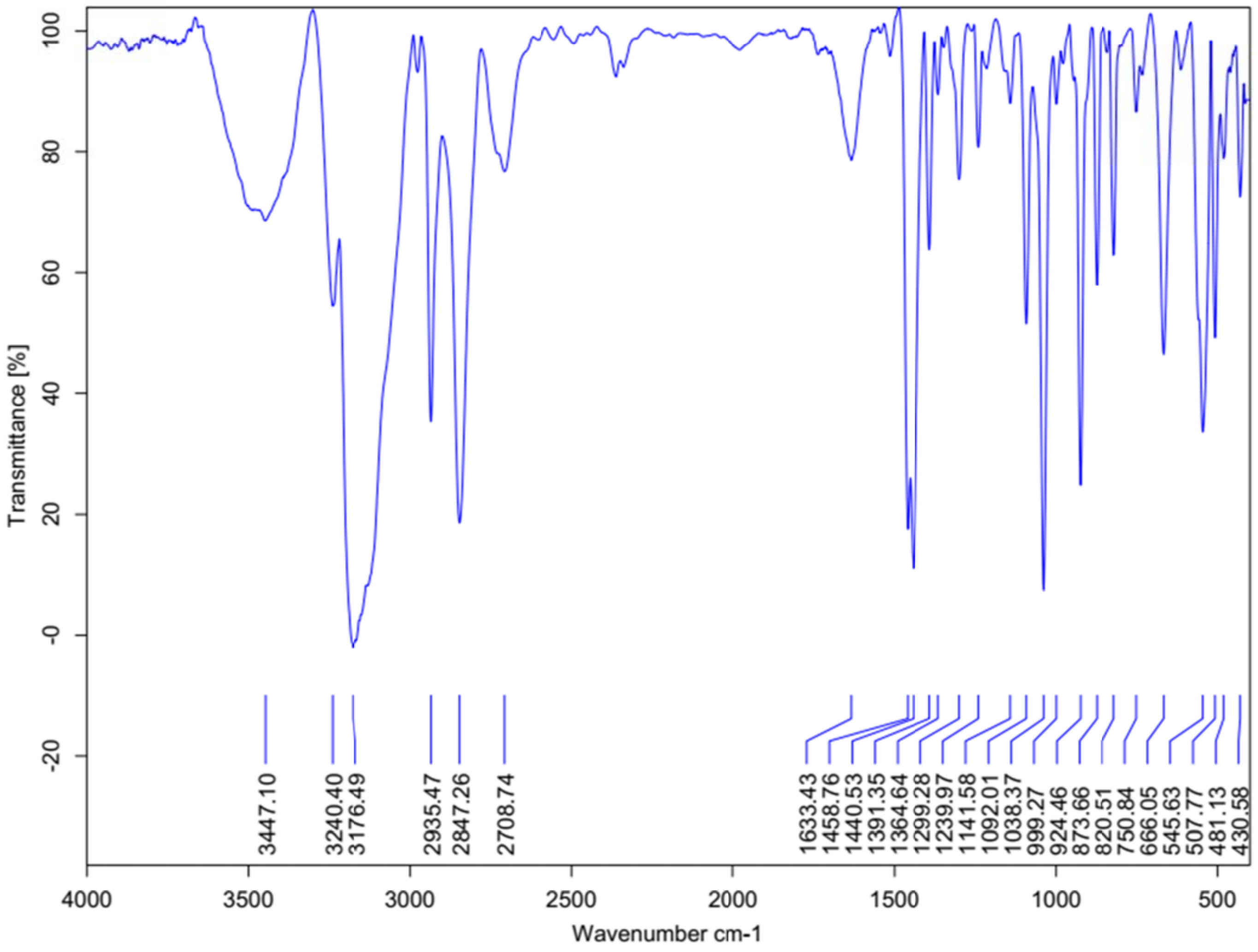

UV-Vis of Cu(cyclam(OH)$_4$)Cl$_2$, H_2O

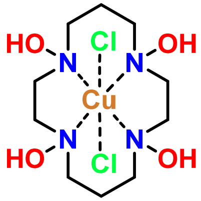

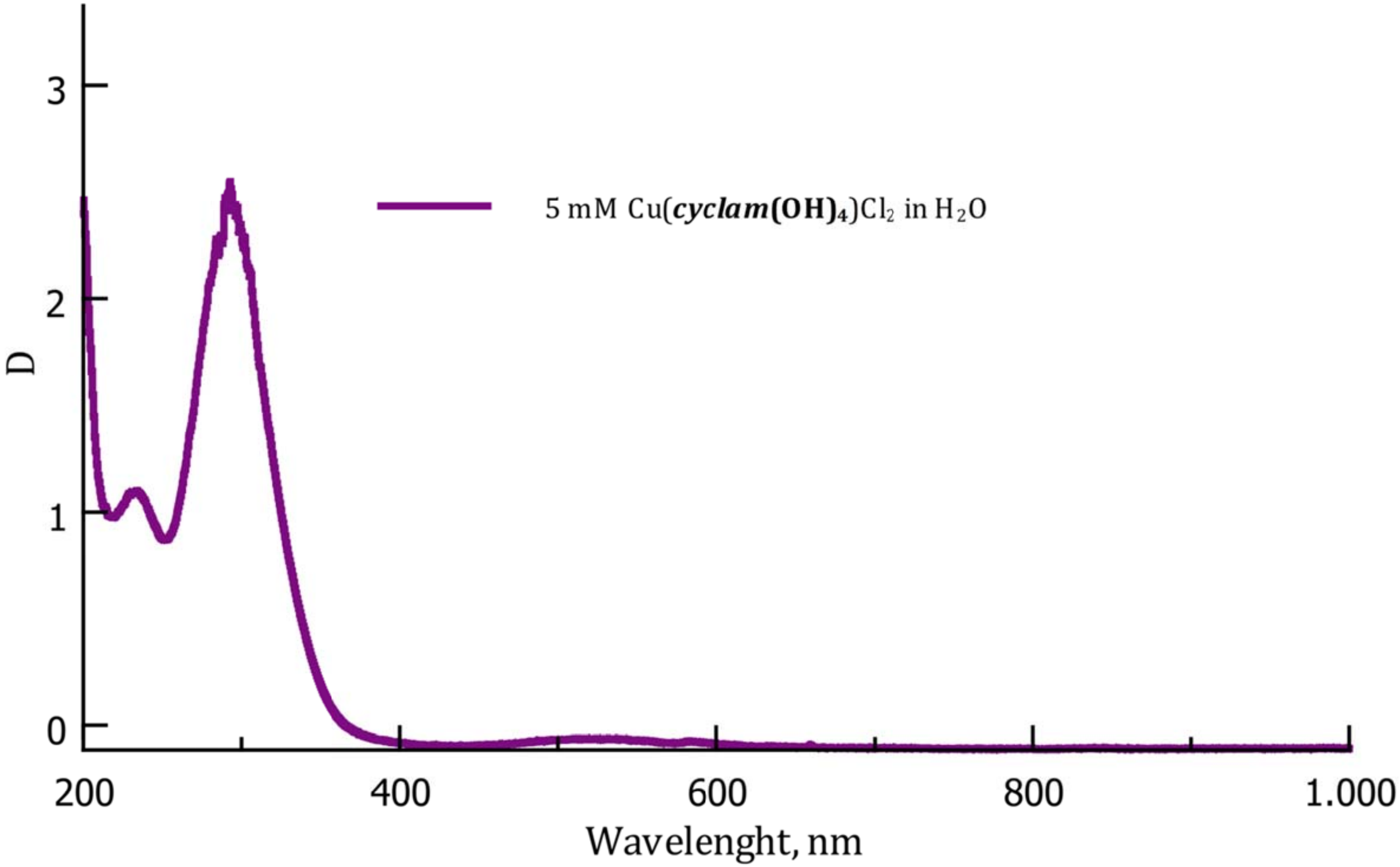

UV-Vis spectra of Cu(cyclam(OH)$_4$)Cl$_2$ in neutral and alkali solutions (H_2O)

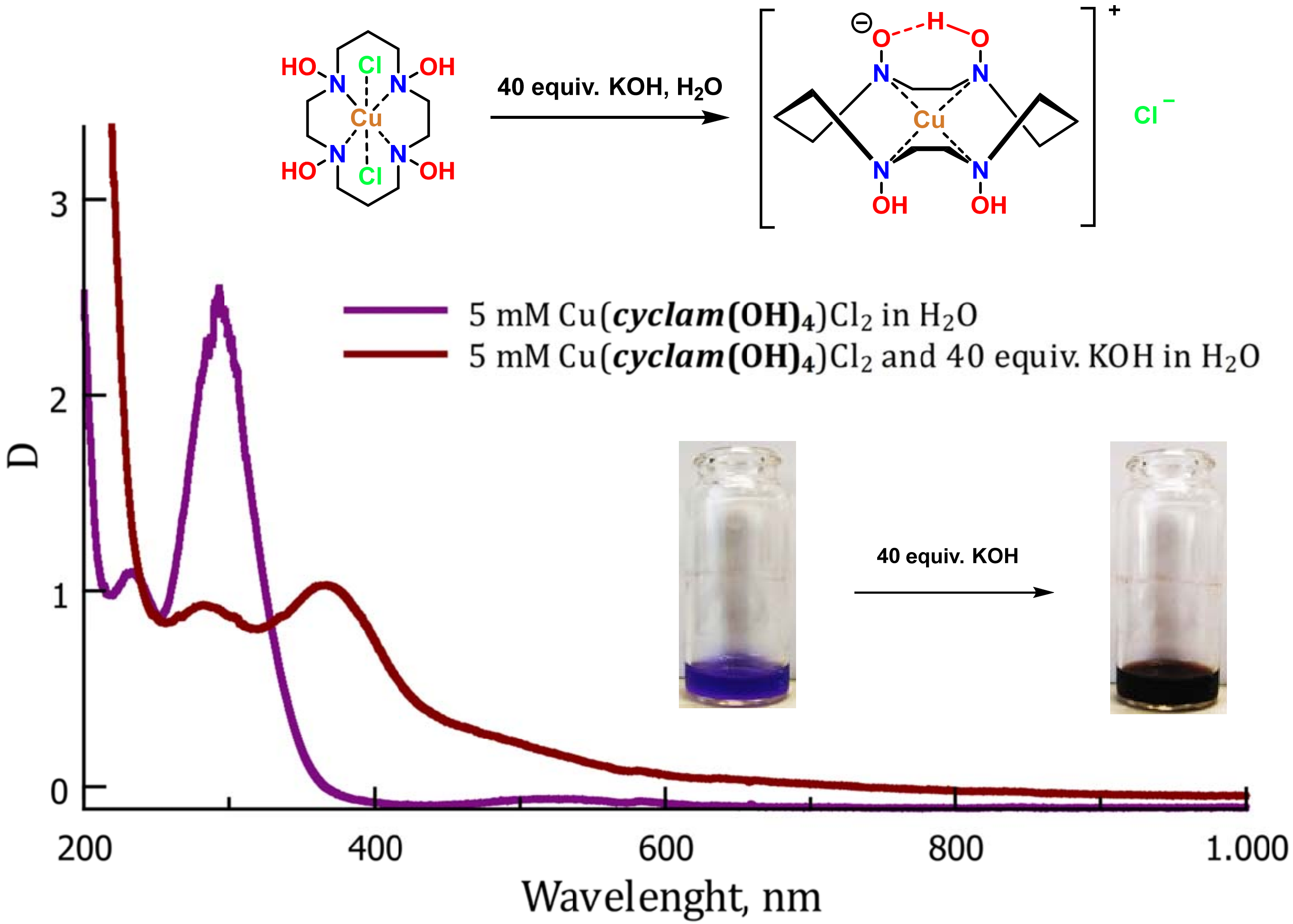

^{1}H NMR of Zn(cyclam(OH)$_4$)Cl_2, D_2O, 315K

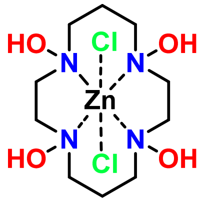

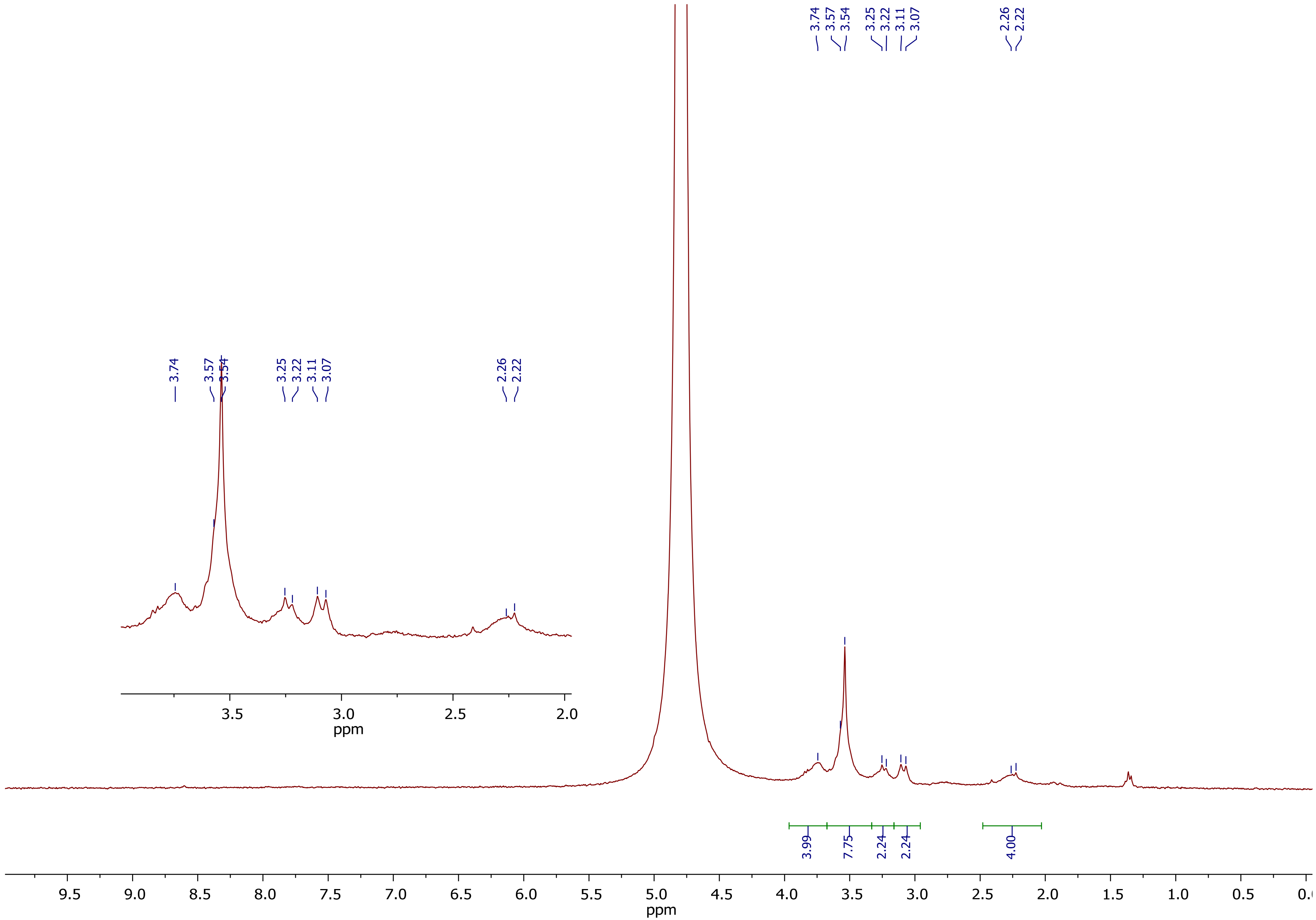

FT-IR of $Zn(cyclam(OH)_4)Cl_2$, KBr

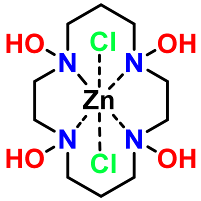

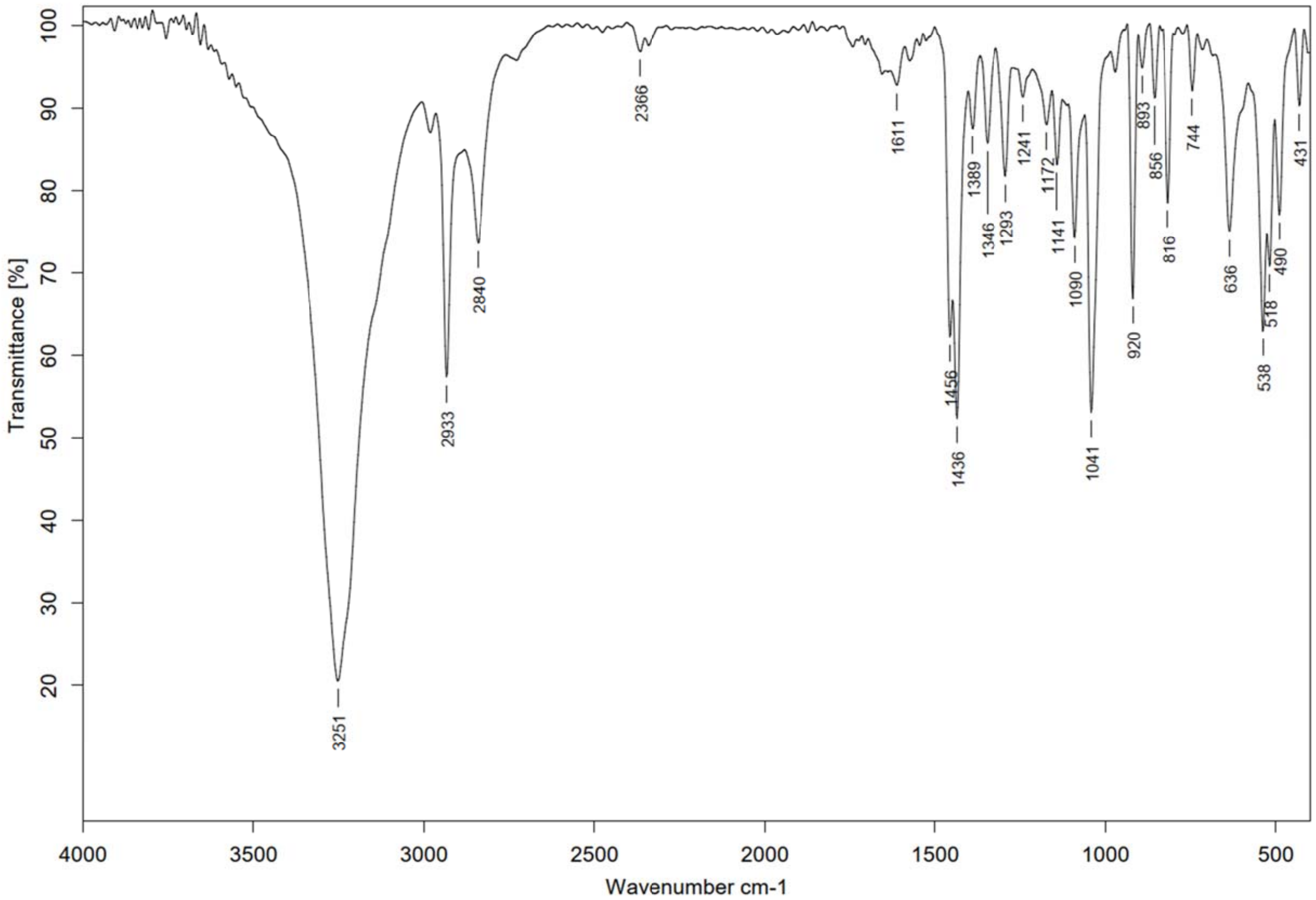

FT-IR of Mn(cyclam(OH)$_4$)Cl$_2$, KBr

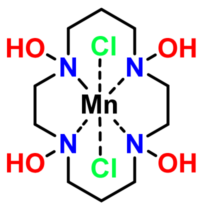

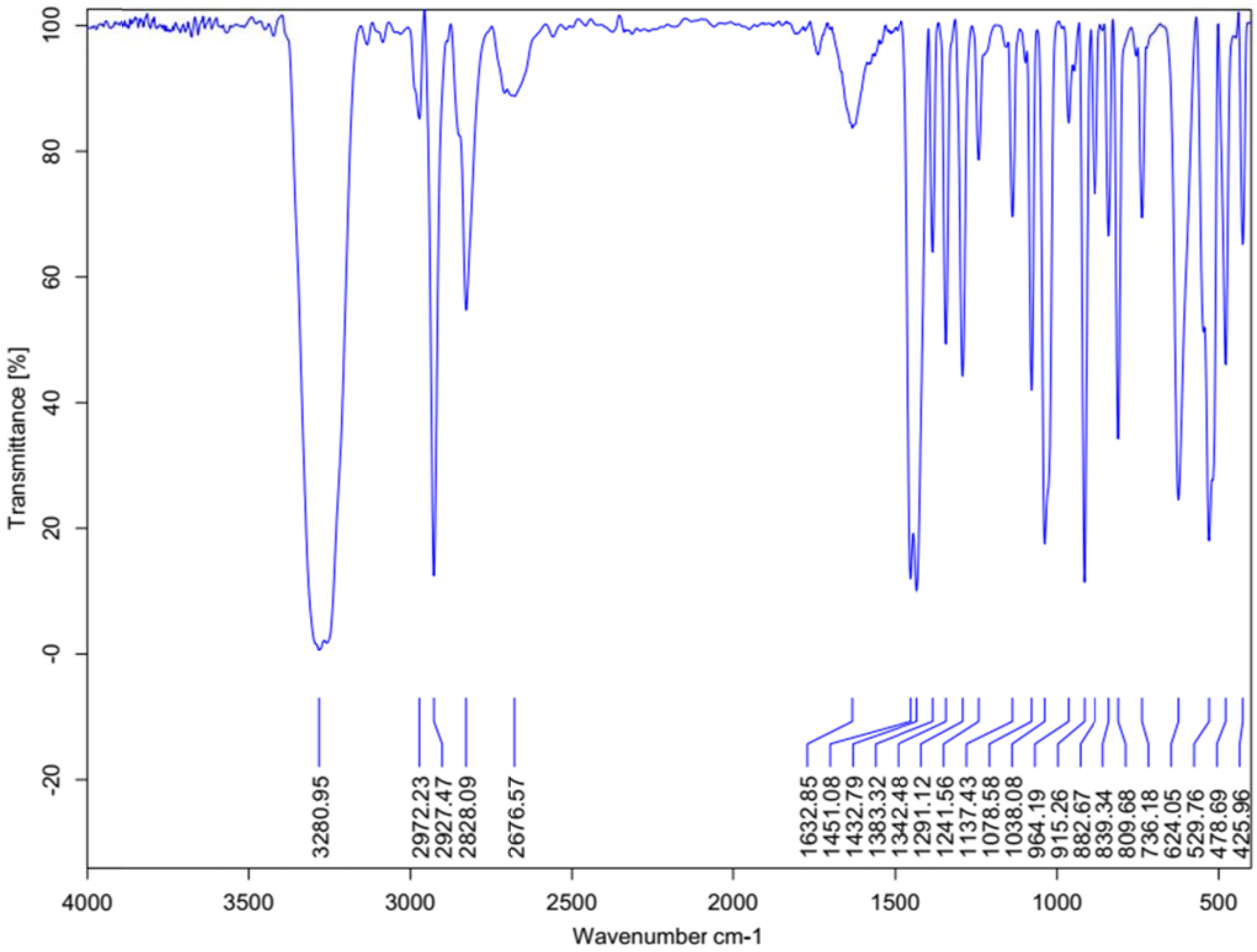

FT-IR of Mn(cyclam(OH)$_4$)Br$_2$•1.33 cyclam(OH)$_4$, KBr

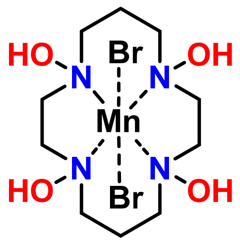

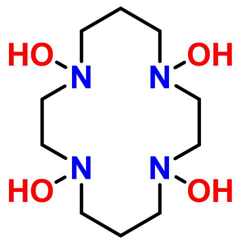

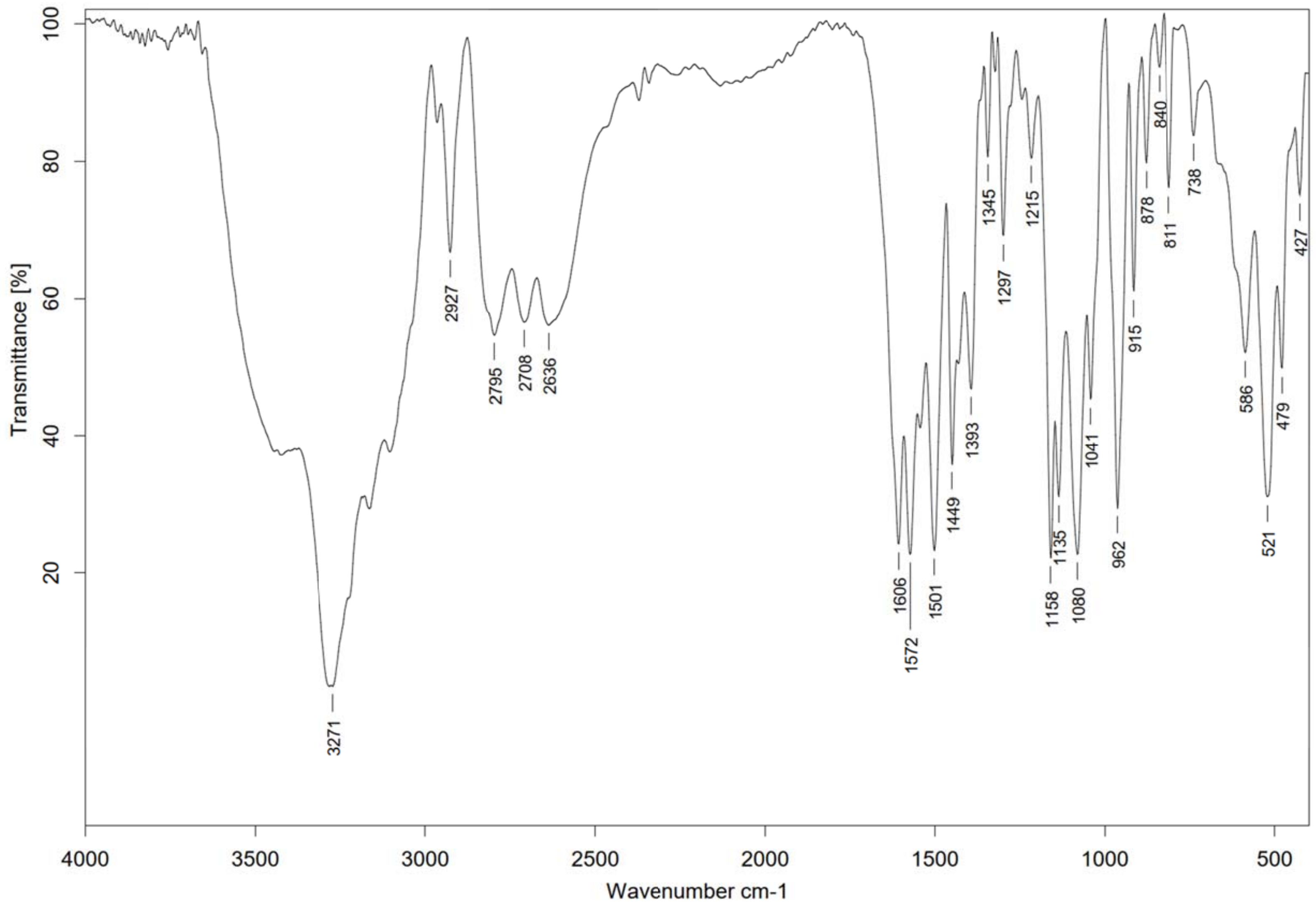

^{1}H NMR of Ni(cyclam(OH)$_4$)(NO$_3$)$_2$, D$_2$O, 298K

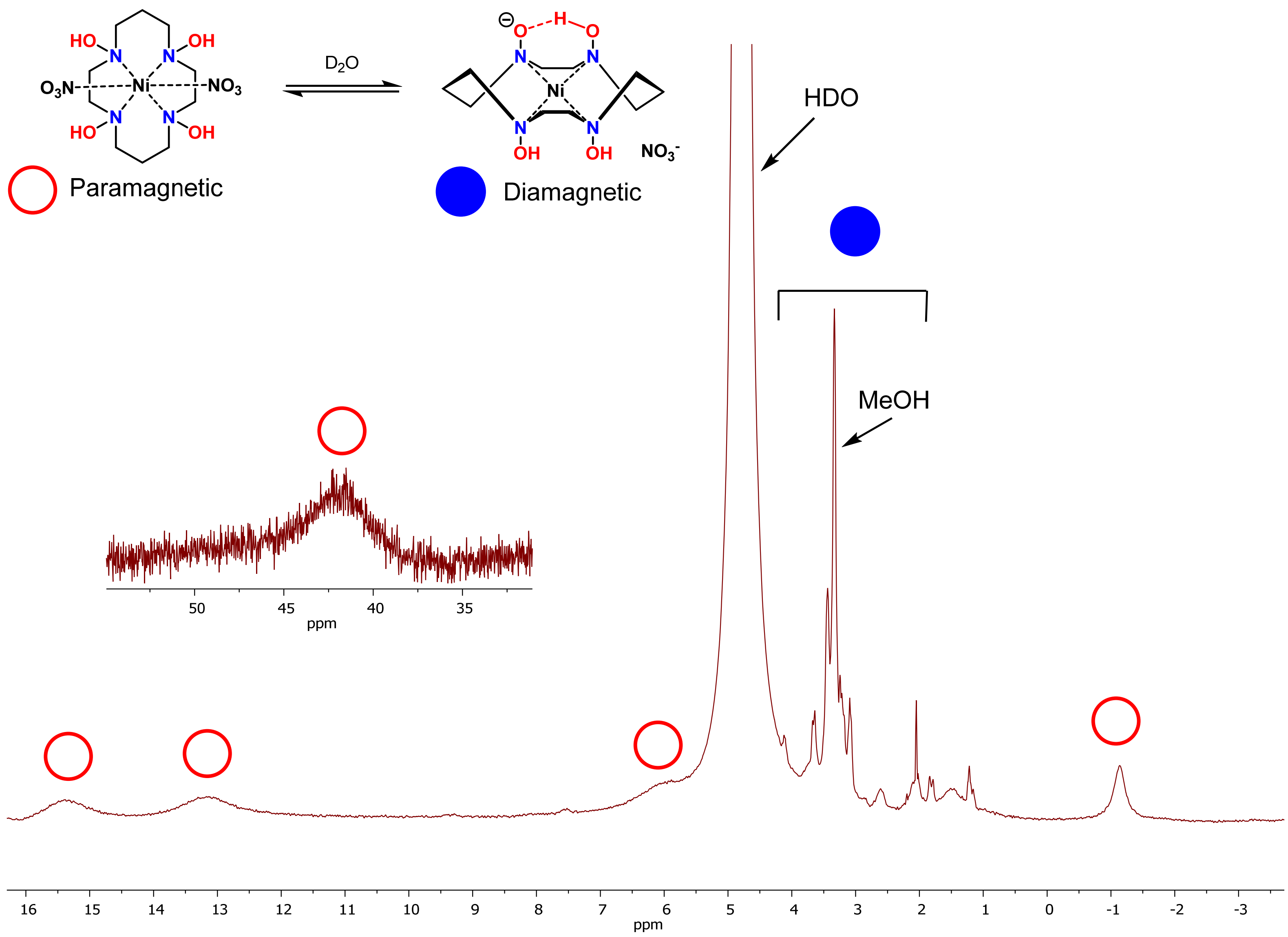

^{1}H NMR of Ni(cyclam(OH)$_4$)(NO$_3$)$_2$ in D_2O with 40 equiv. of KOH (298K)

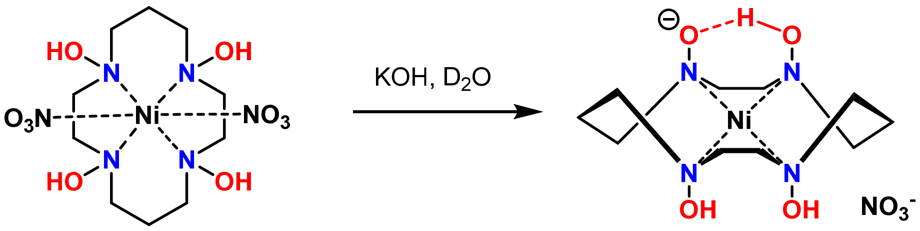

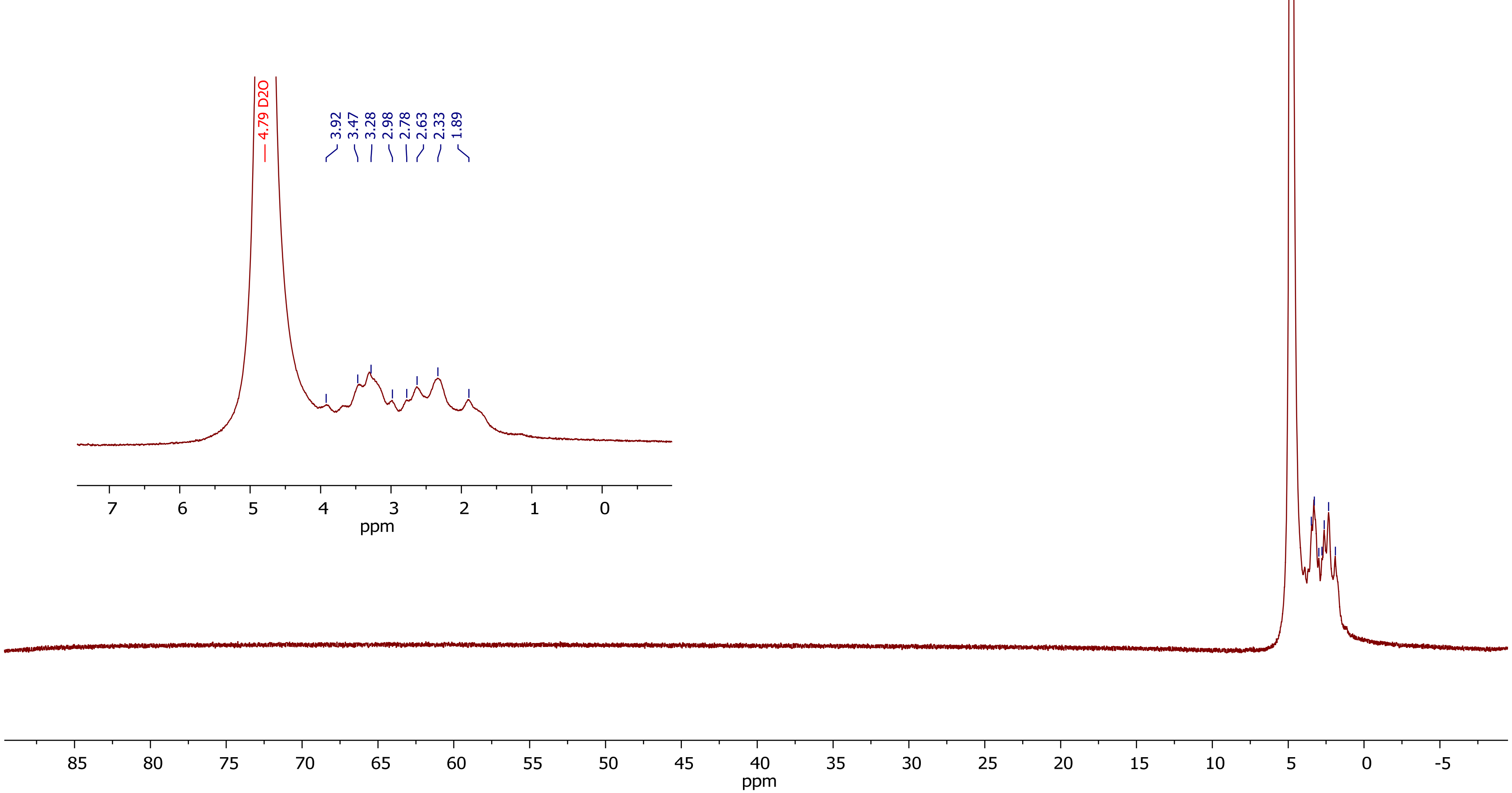

FT-IR of $Ni(cyclam(OH)_4)(NO_3)_2$, KBr

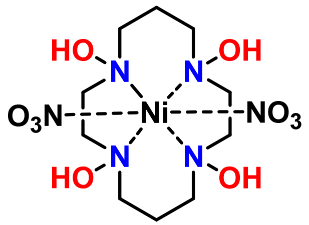

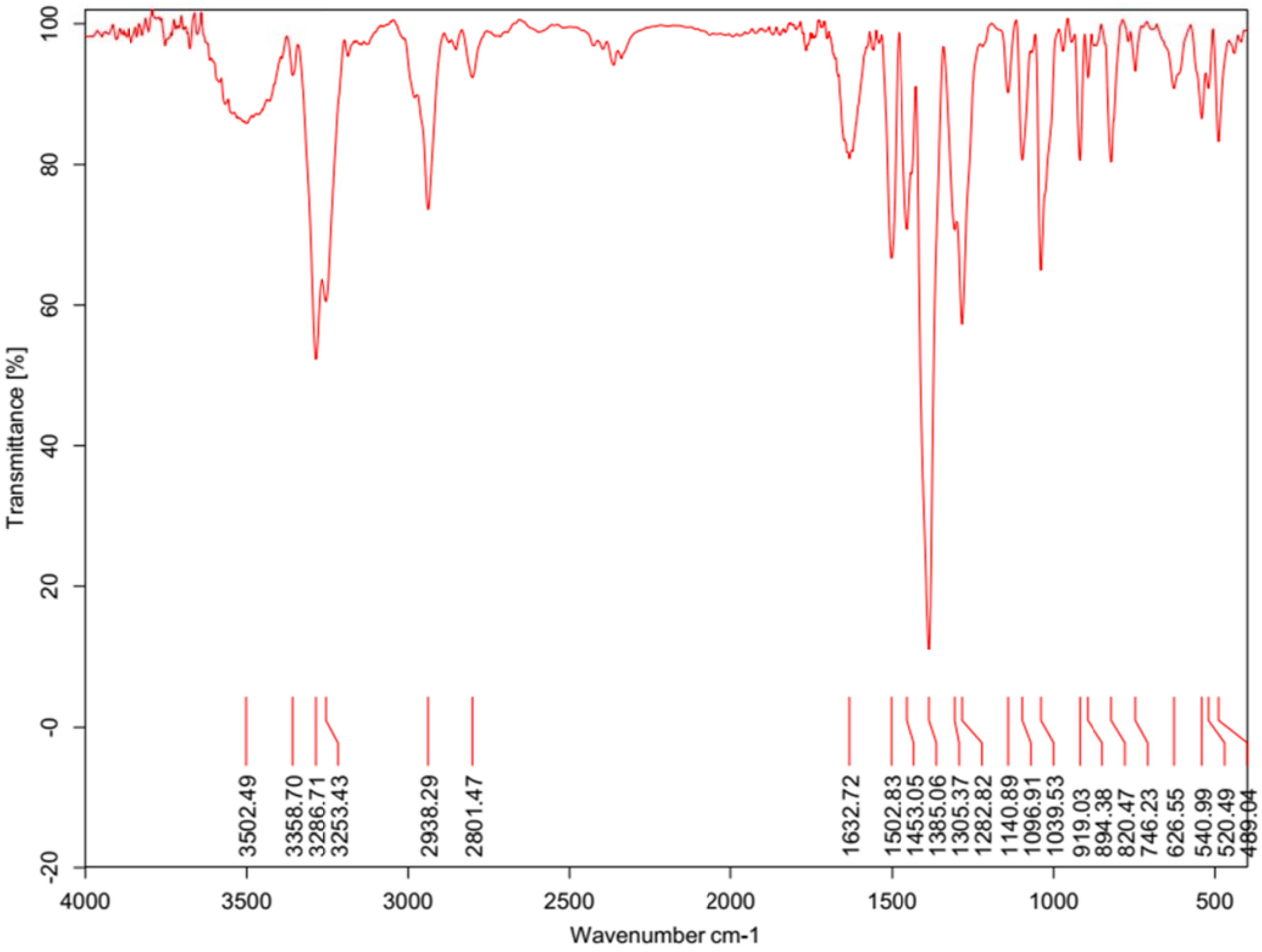

UV-Vis of Ni(cyclam(OH)$_4$)(NO_3)$_2$, H_2O

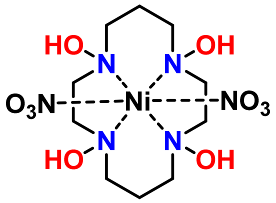

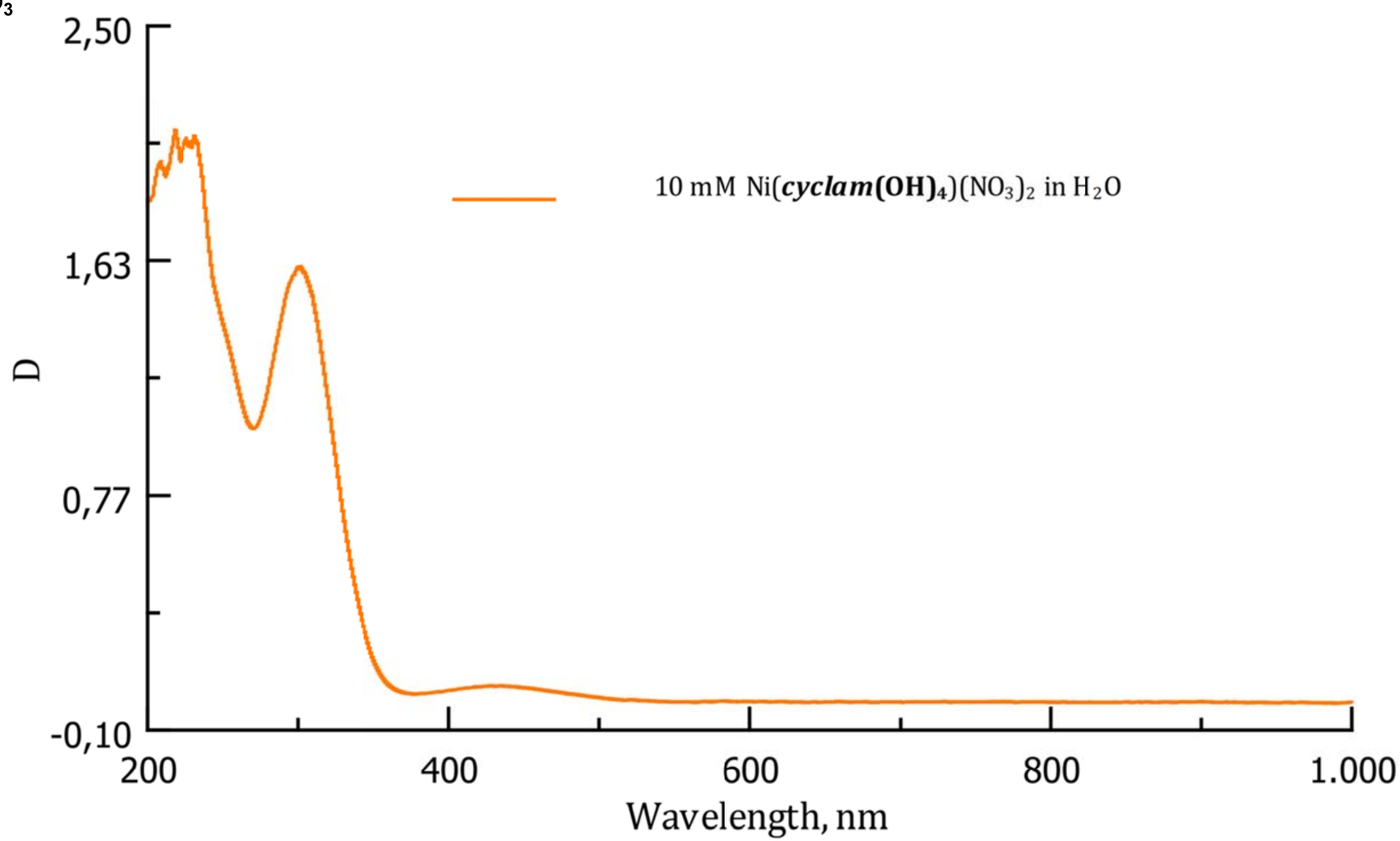

FT-IR of Ni(cyclam(OH)$_4$)(ClO$_4$)$_2$, KBr

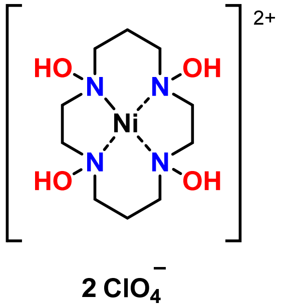

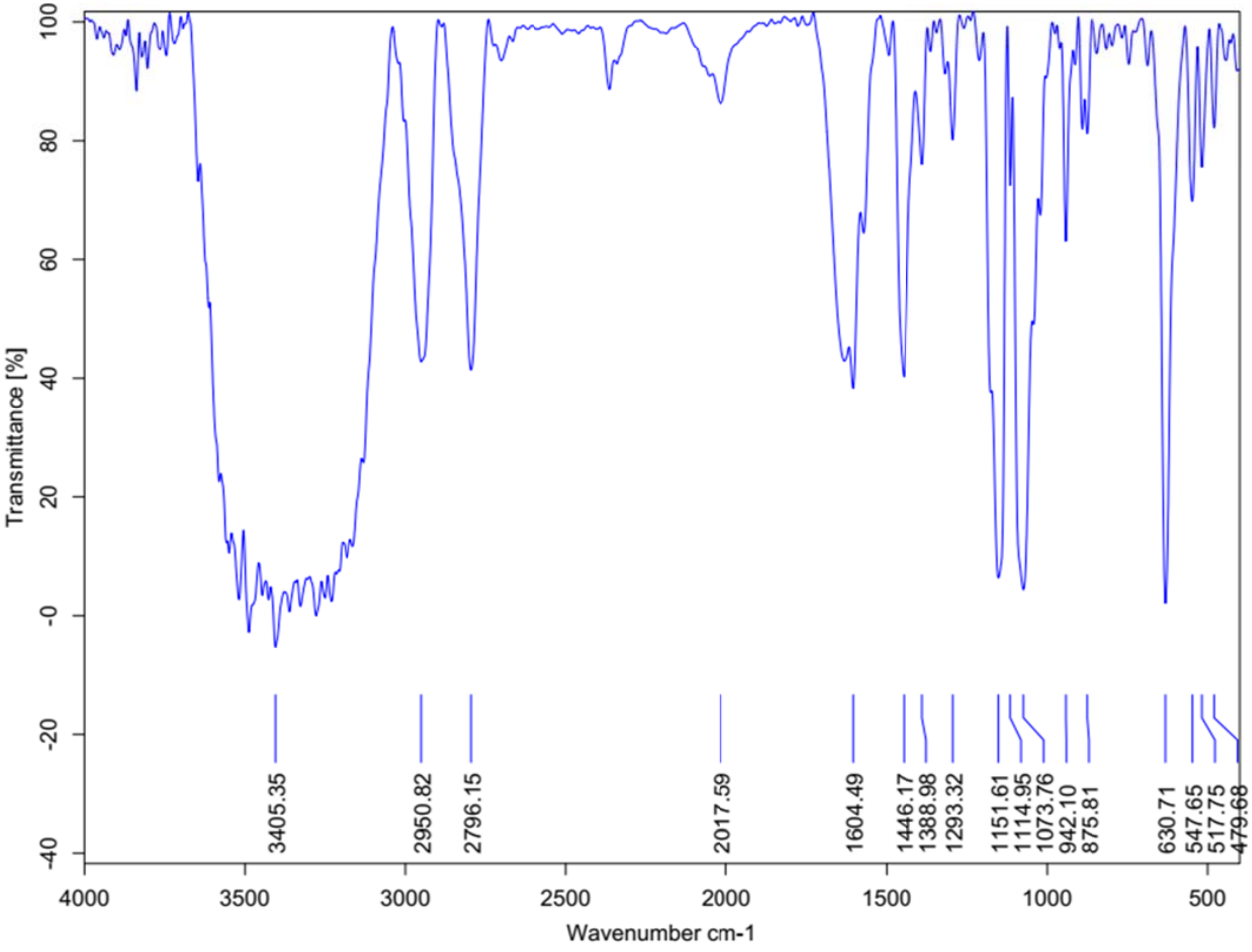

UV-Vis of Ni(cyclam(OH)$_4$)(ClO$_4$)$_2$, H_2O

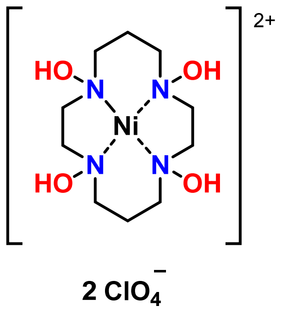

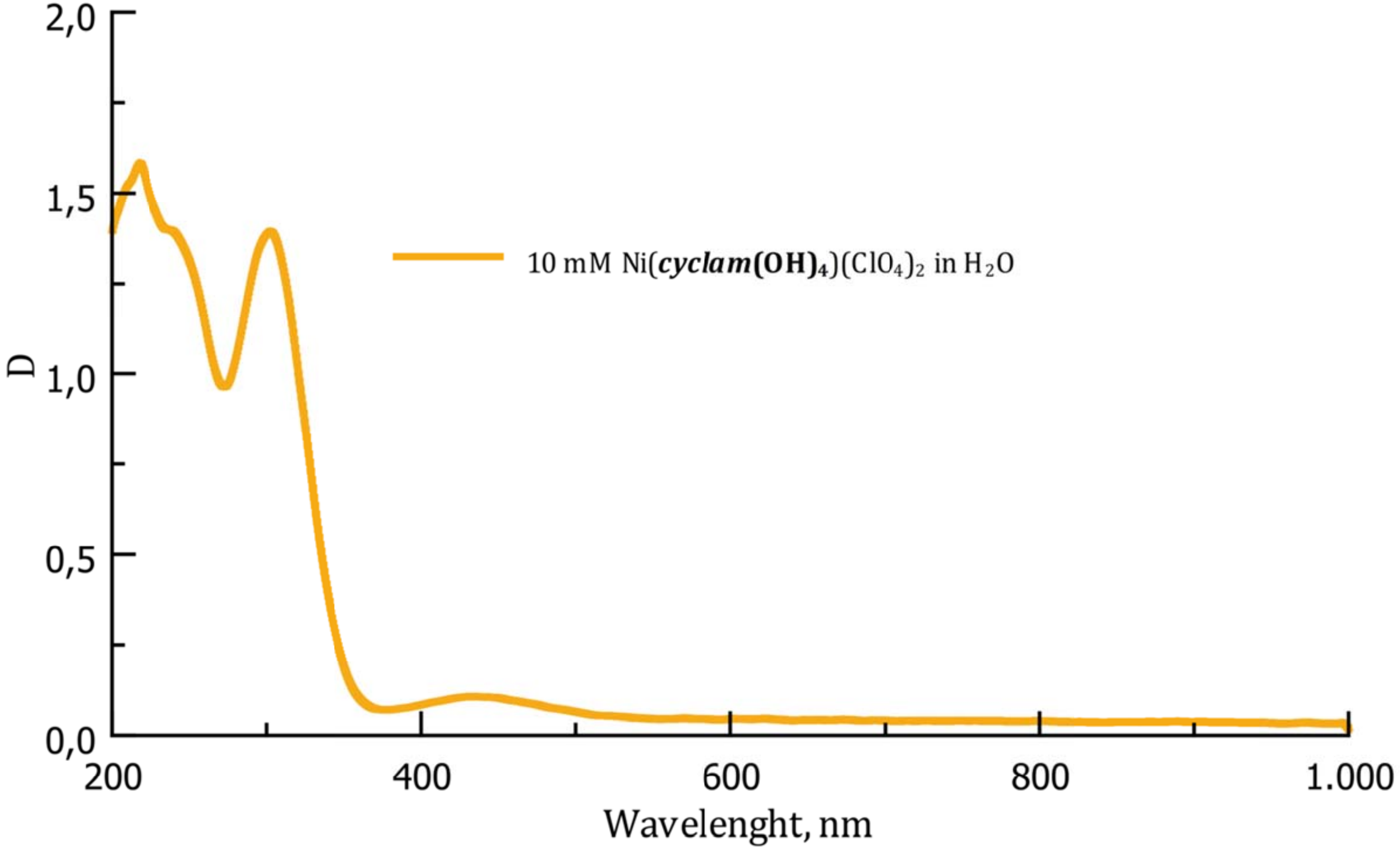

UV-Vis spectra of Ni(cyclam(OH)$_4$)(ClO$_4$)$_2$ in neutral, acidic and alkali solutions (H_2O)

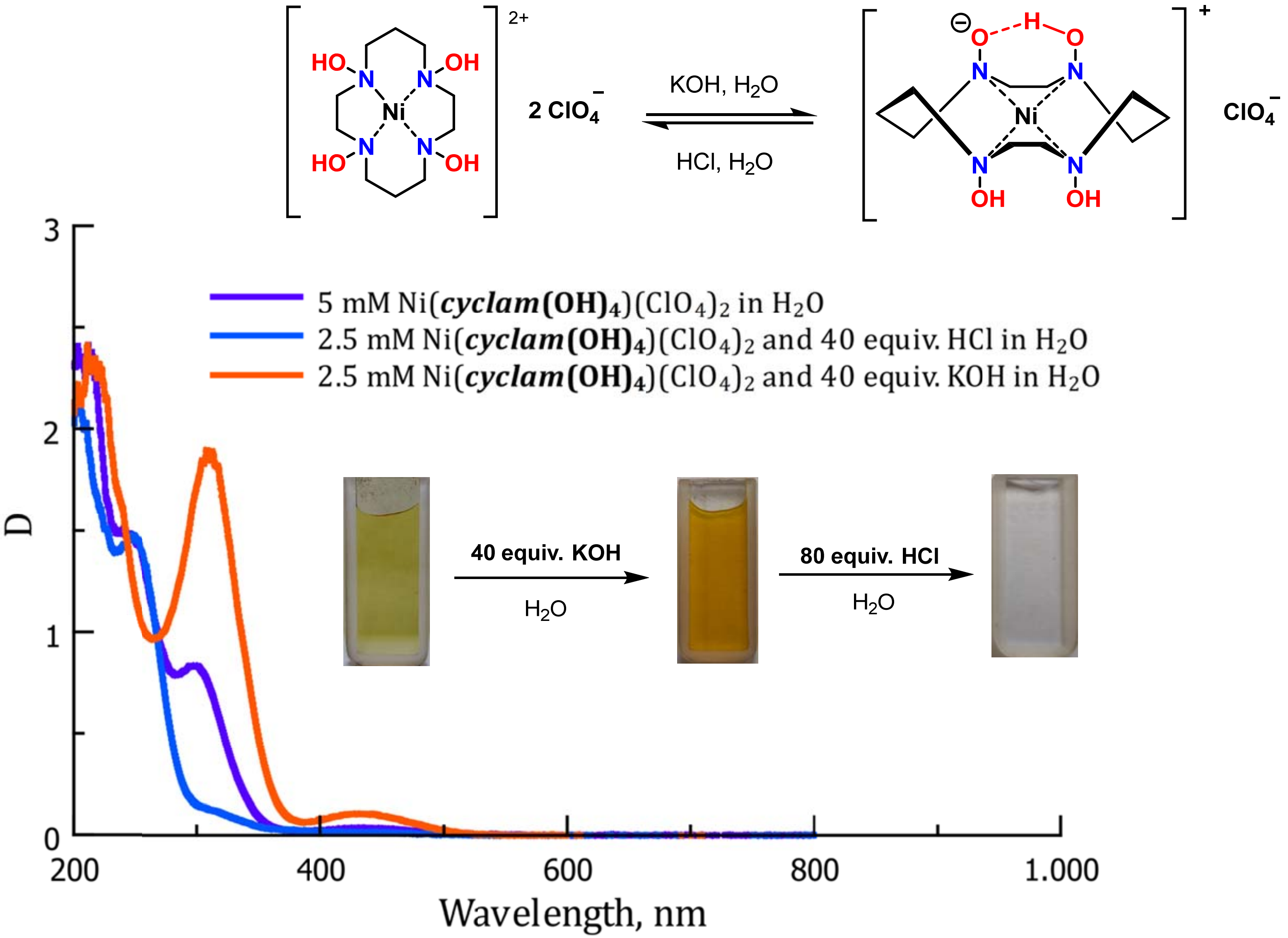

^1H NMR of $Ni(cyclam(O^-)(OH)_3)(NO_3)$•$Ni(cyclam(OH)_4)(NO_3)_2$, D_2O, 298K

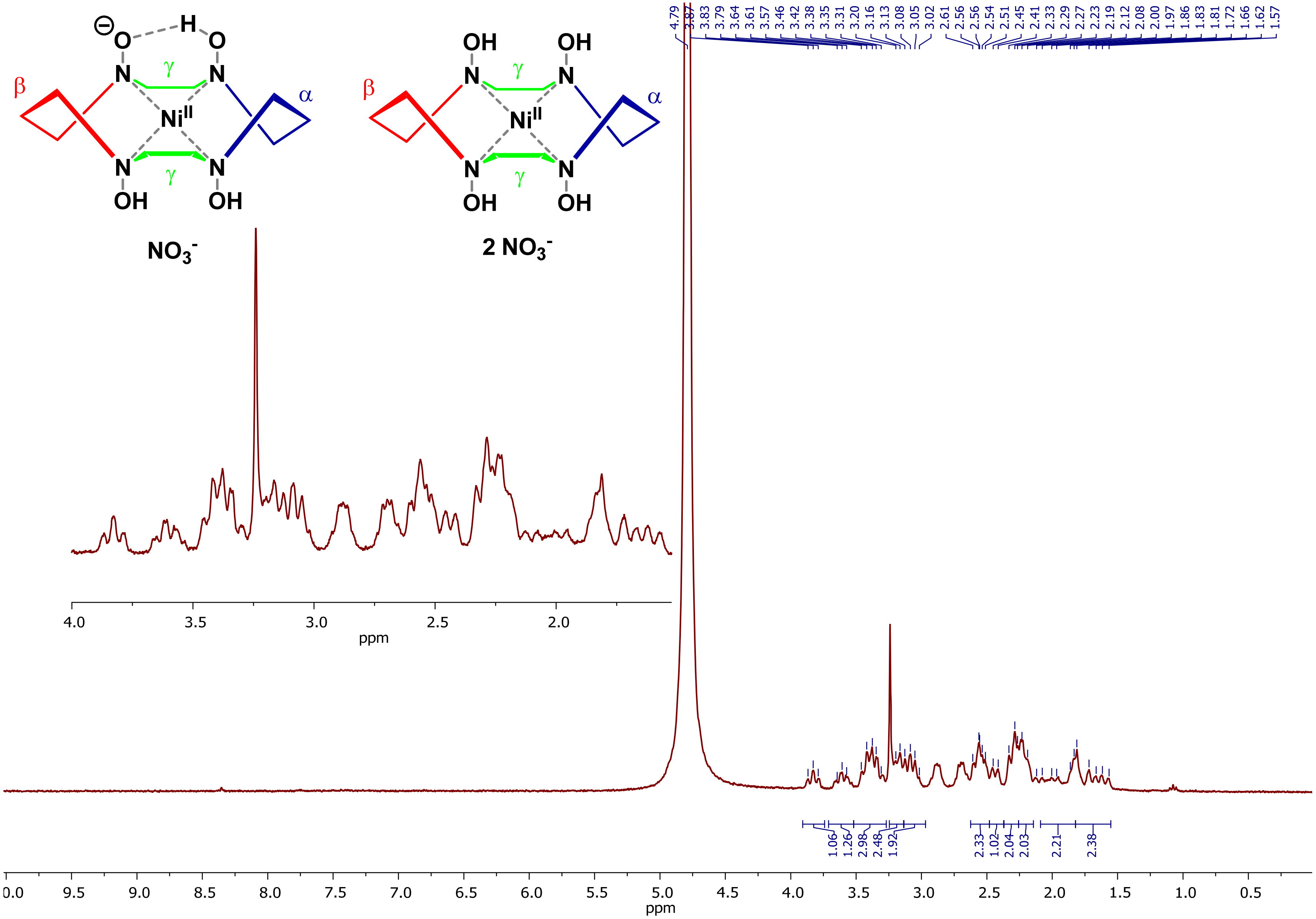

^{13}C NMR of $Ni(cyclam(O^{-})(OH)_3)(NO_3)$•$Ni(cyclam(OH)_4)(NO_3)_2$, D_2O, 298K

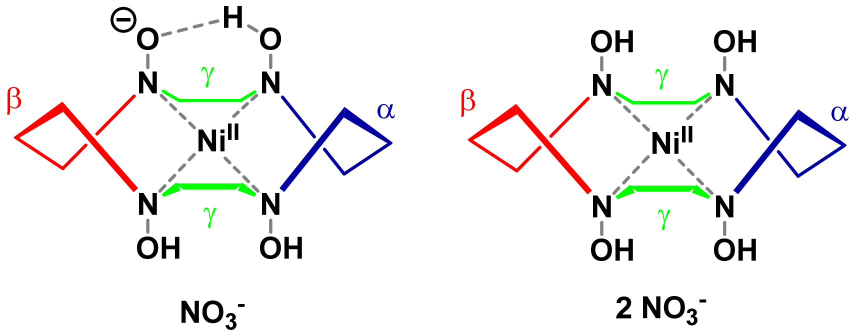

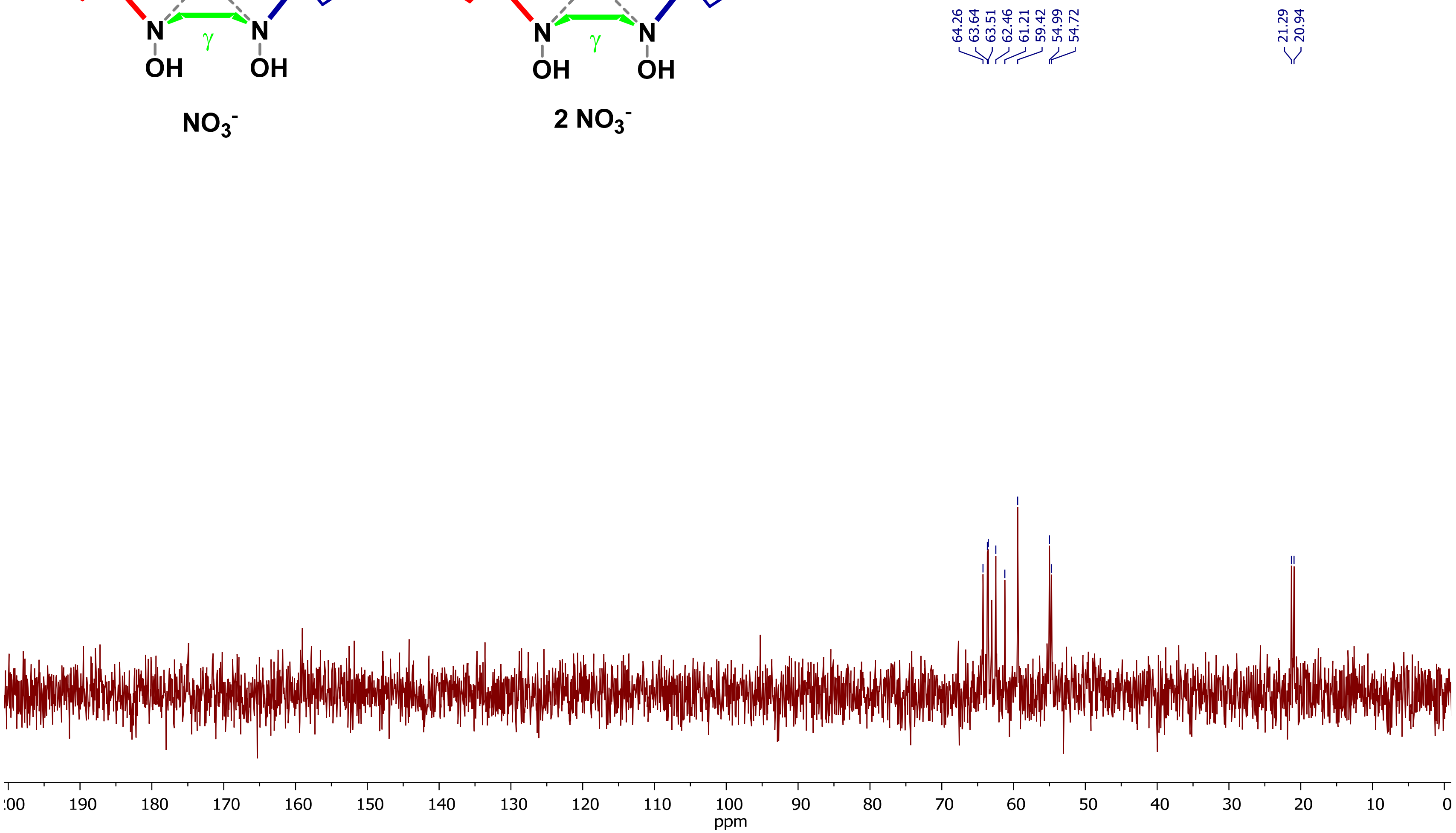

$^{1}H-^{13}C$ HSQC of $Ni(cyclam(O^{-})(OH)_3)(NO_3)\cdot Ni(cyclam(OH)_4)(NO_3)_2$, D_2O, 298K

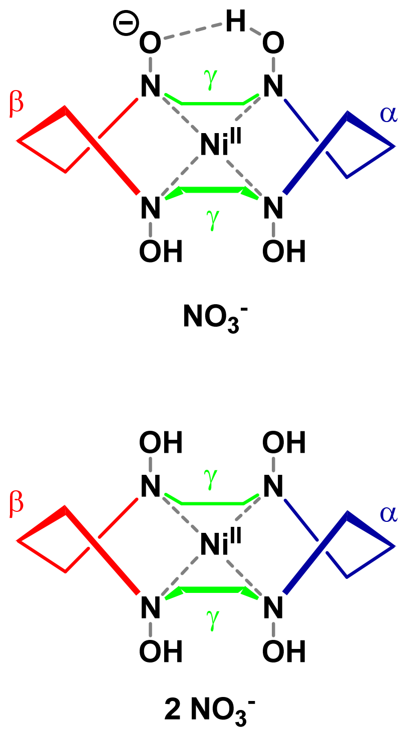

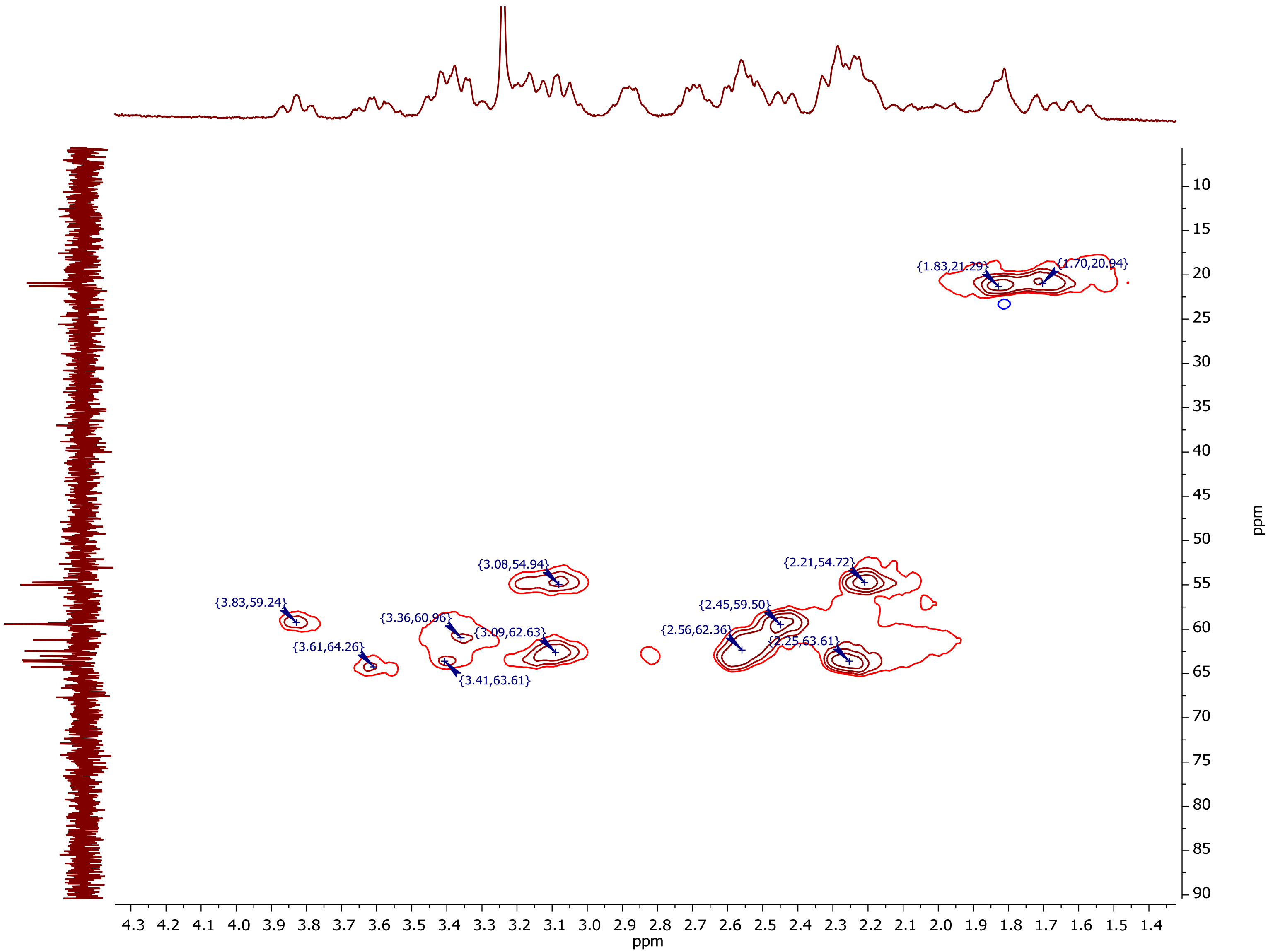

^{1}H-^{1}H COSY of Ni(cyclam(O^-)$(OH)_3$)(NO_3)•Ni(cyclam$(OH)_4$)$(NO_3)_2$, D_2O, 298K

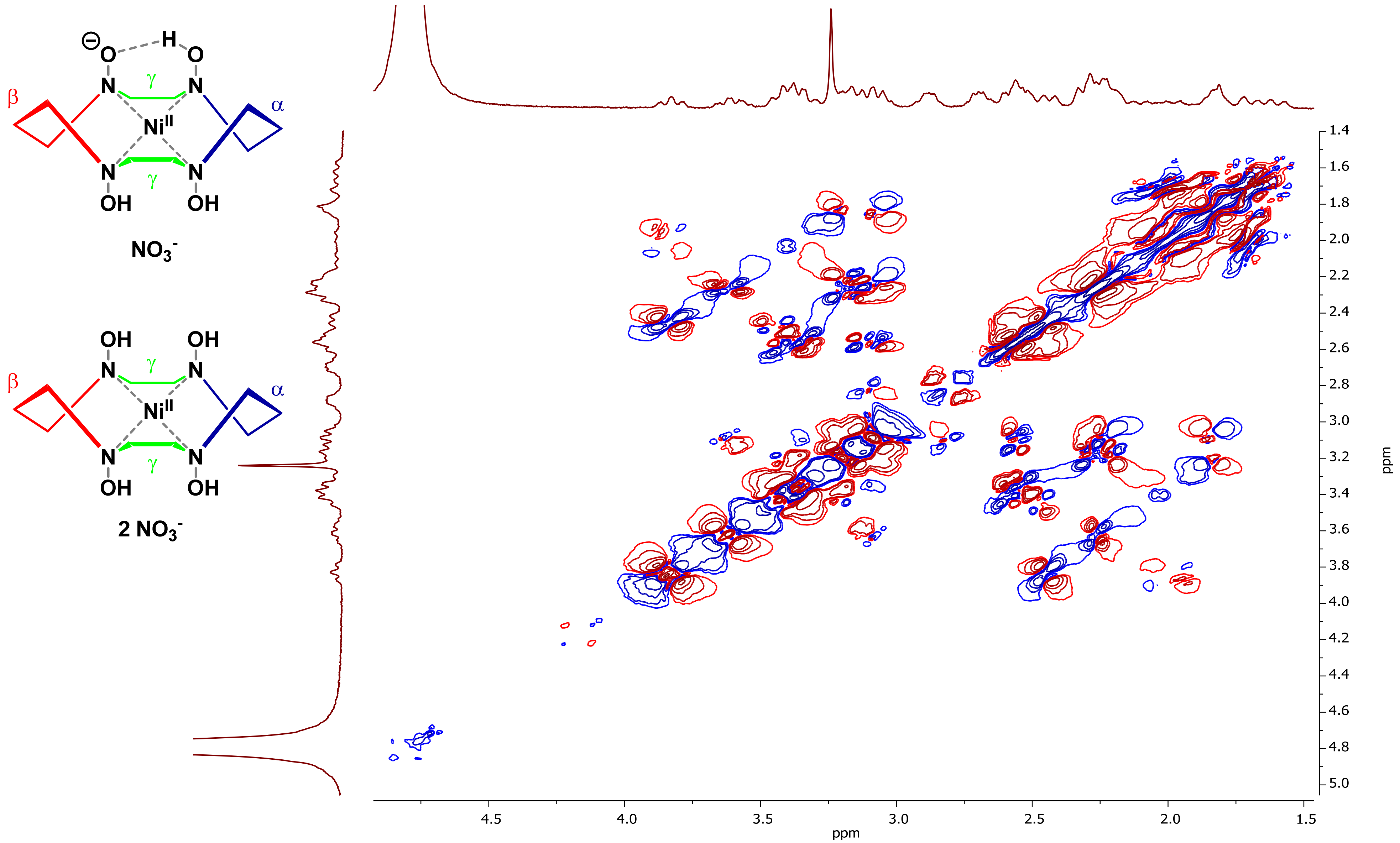

FT-IR of Ni(cyclam(O⁻)(OH)$_3$)(NO$_3$)•Ni(cyclam(OH)$_4$)(NO$_3$)$_2$, KBr

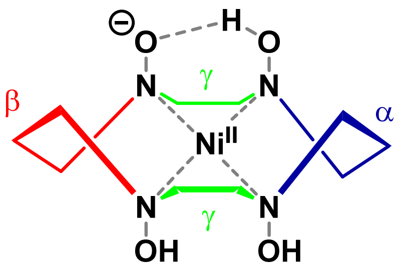

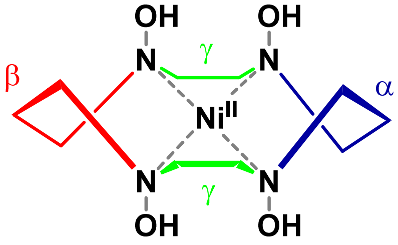

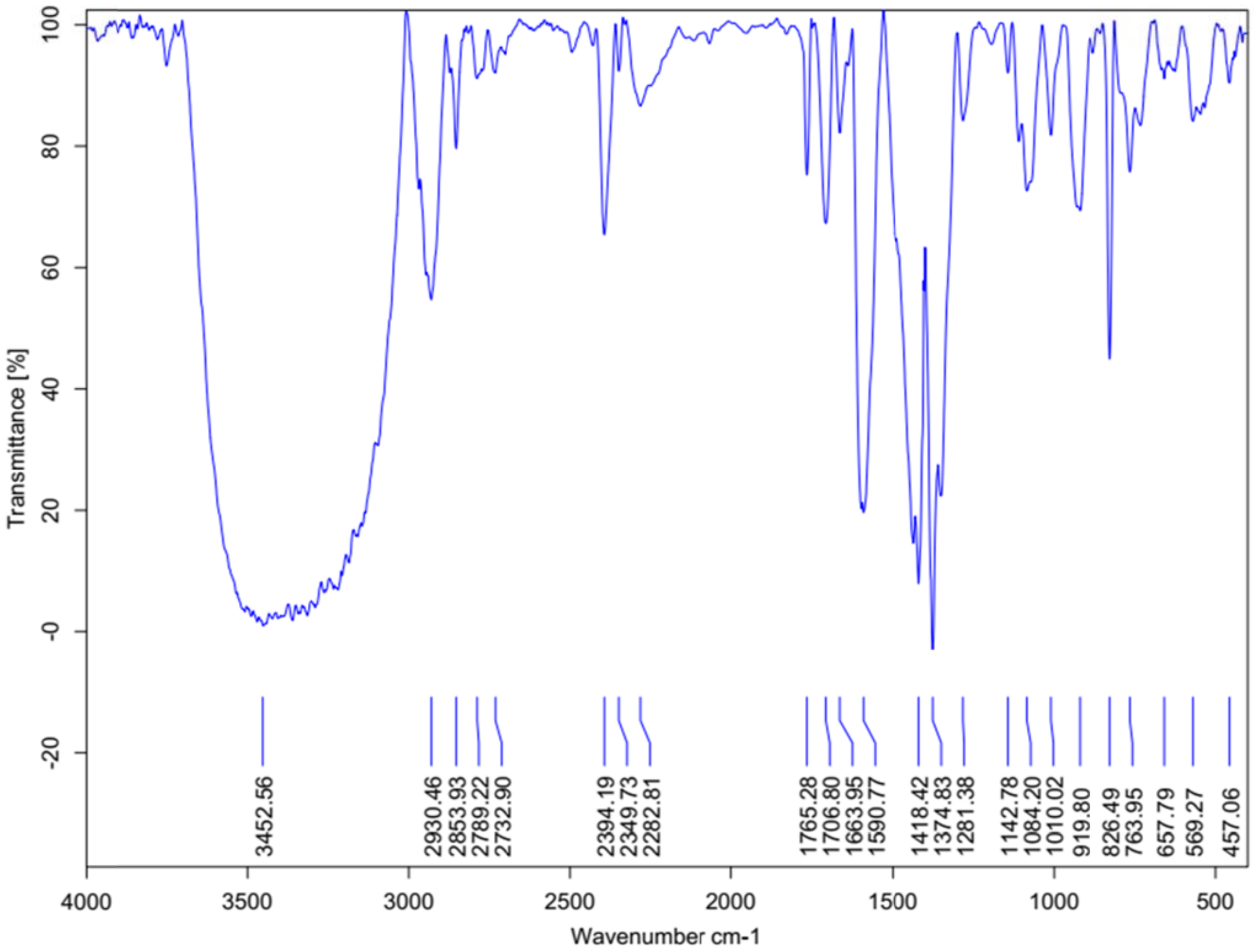

UV-Vis of Ni(cyclam(O^-)$(OH)_3$)(NO_3)•Ni(cyclam$(OH)_4$)$(NO_3)_2$), H_2O (1mM)

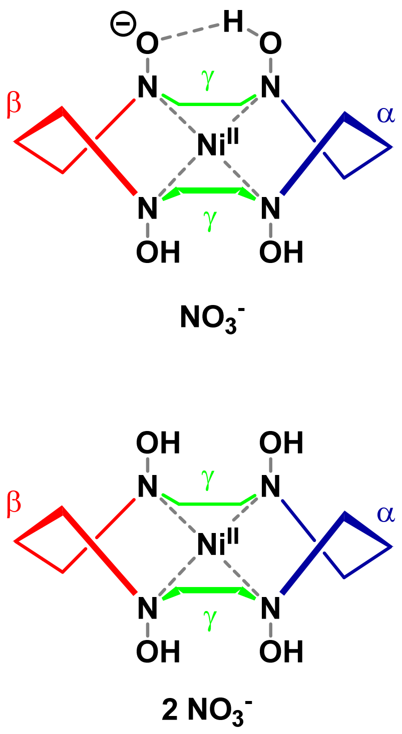

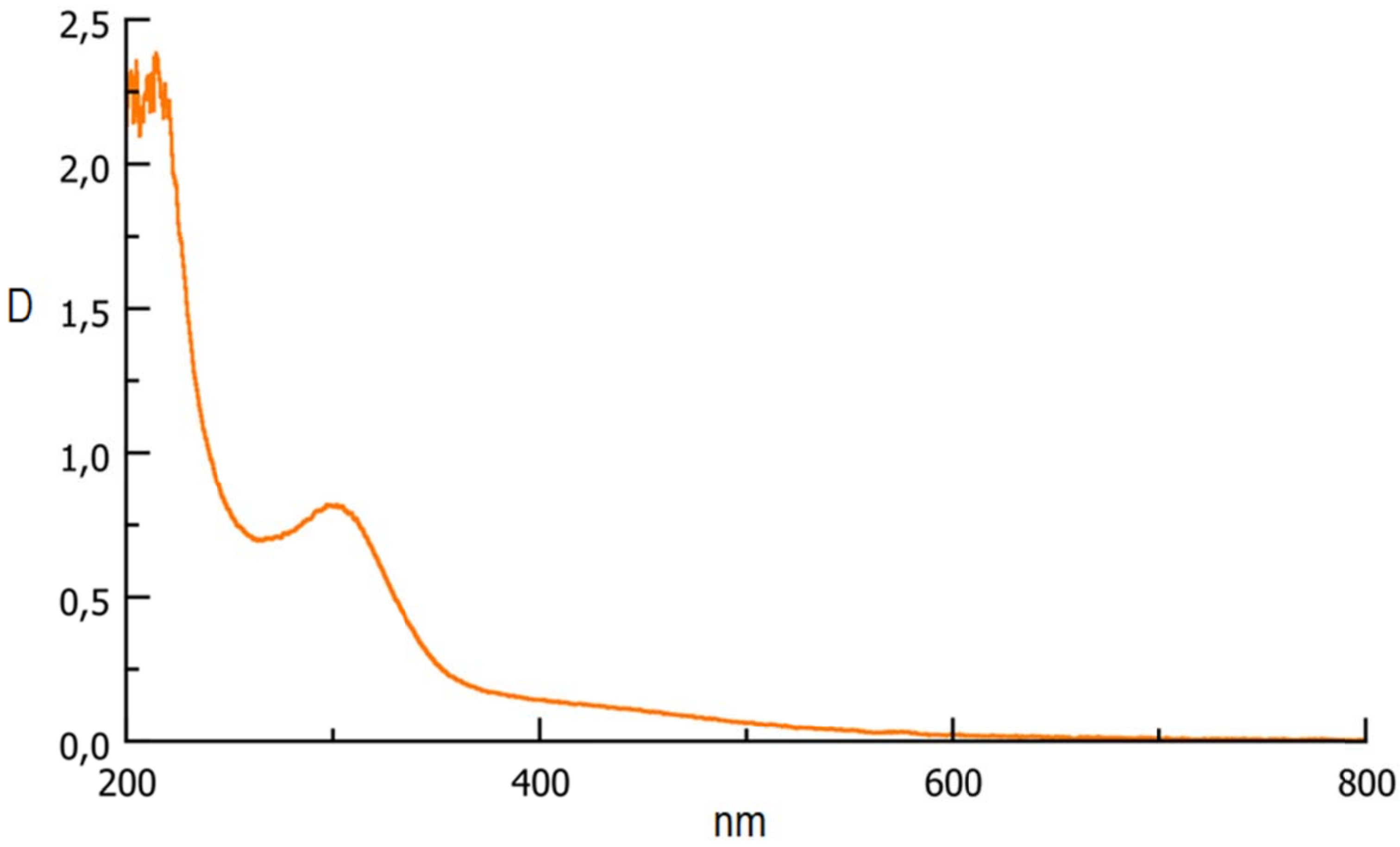

^{1}H NMR, DMSO-d_6, 298K

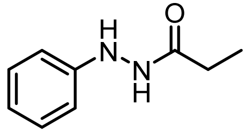

NPPH
2 conformers, 7 : 1

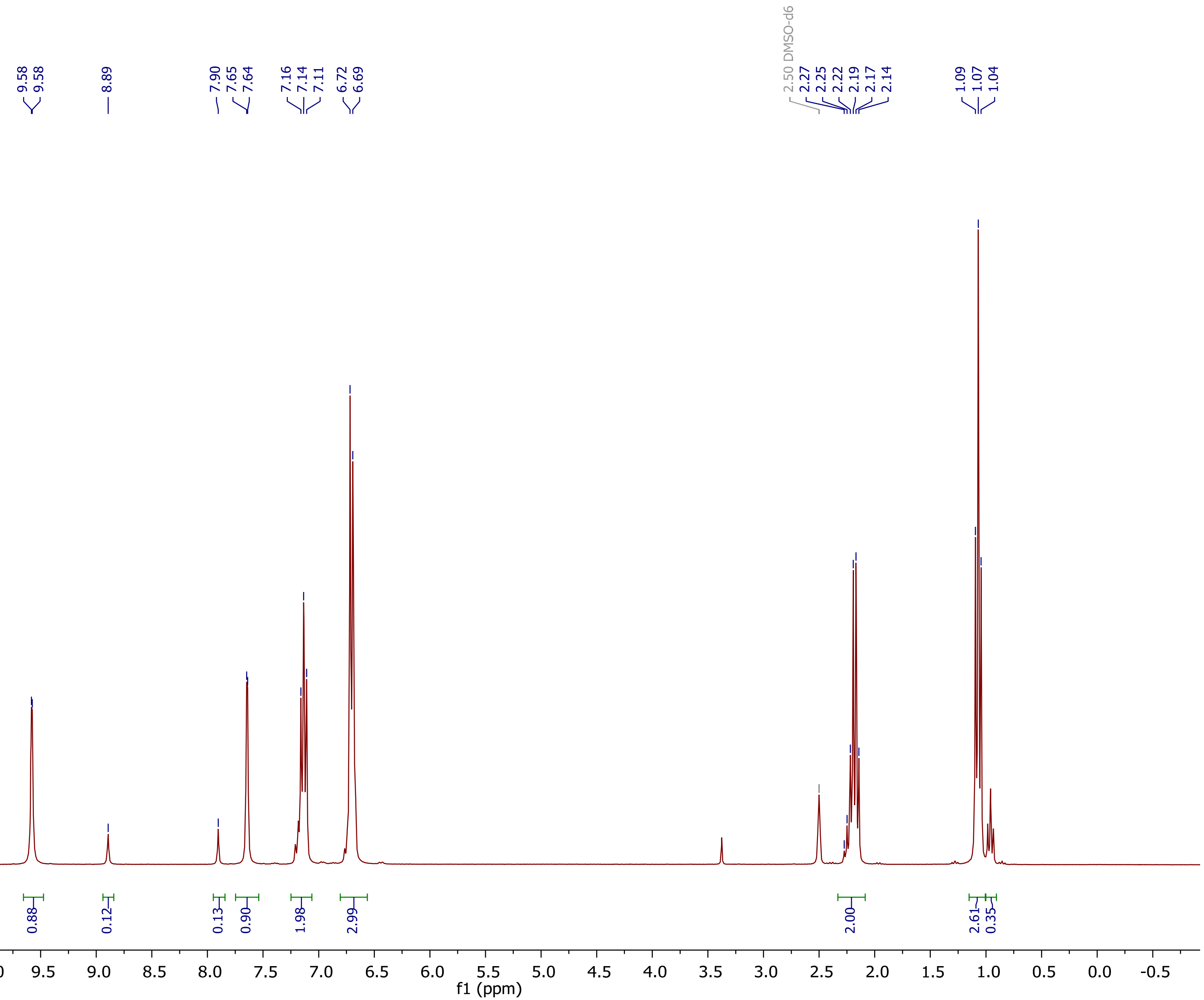

^{13}C NMR, DMSO-d_6, 298K

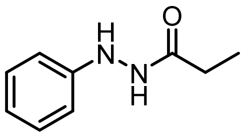

NPPH
2 conformers, 7 : 1

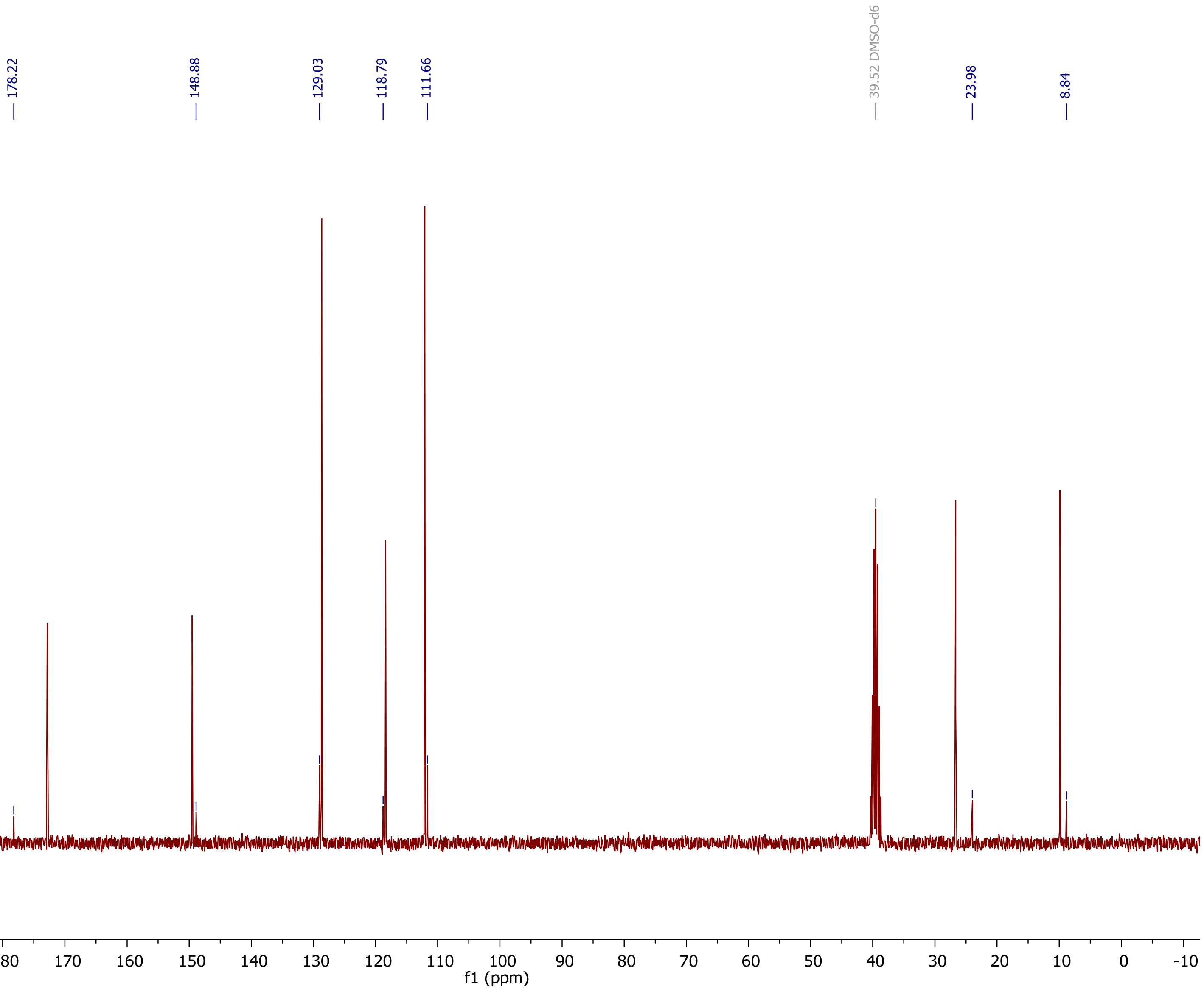

^{1}H-^{13}C HSQC, DMSO-d_6, 298K

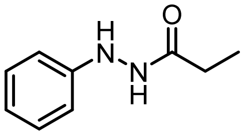

NPPH
2 conformers, 7 : 1

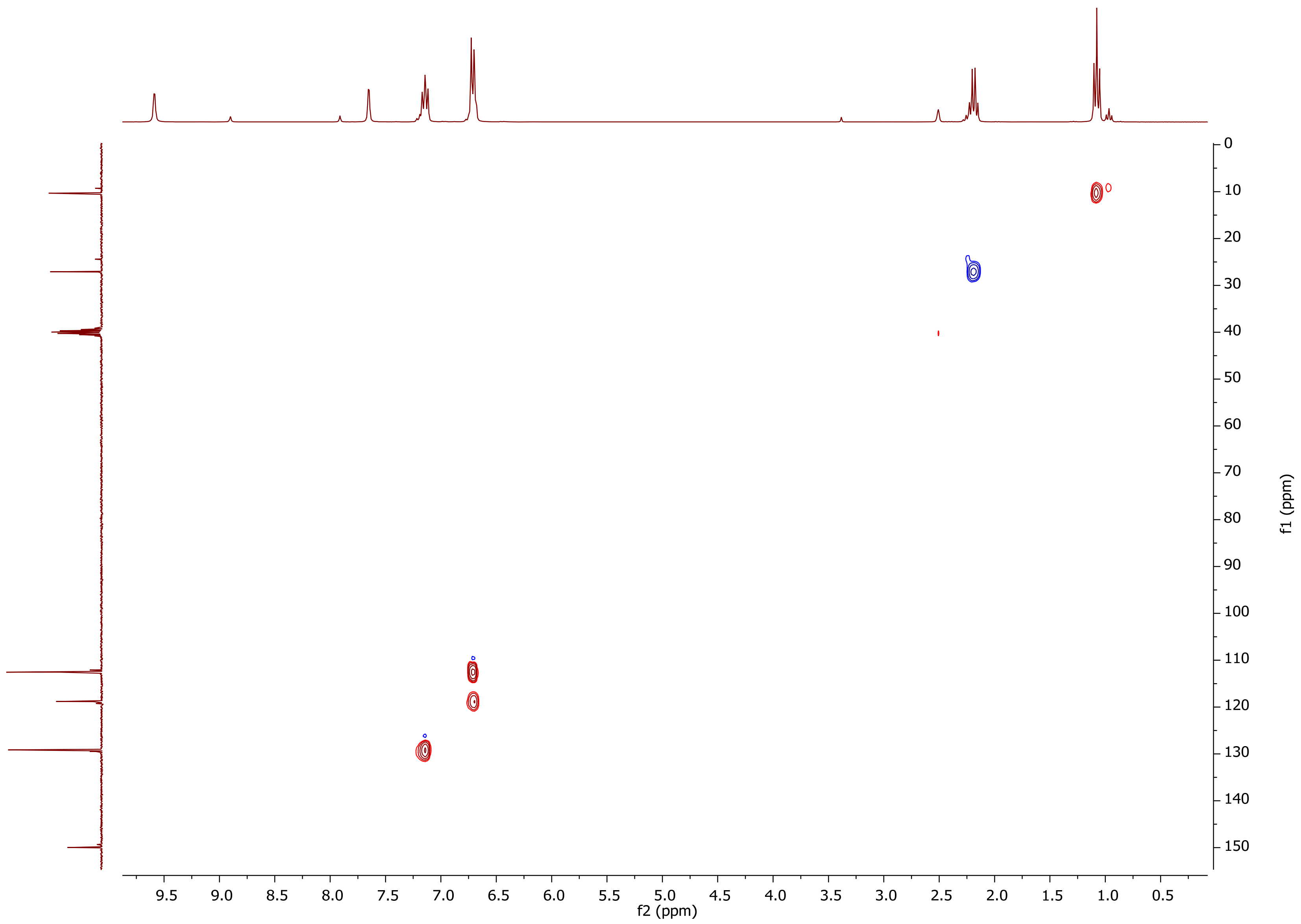

^{1}H NMR, DMSO-d_6, 298K

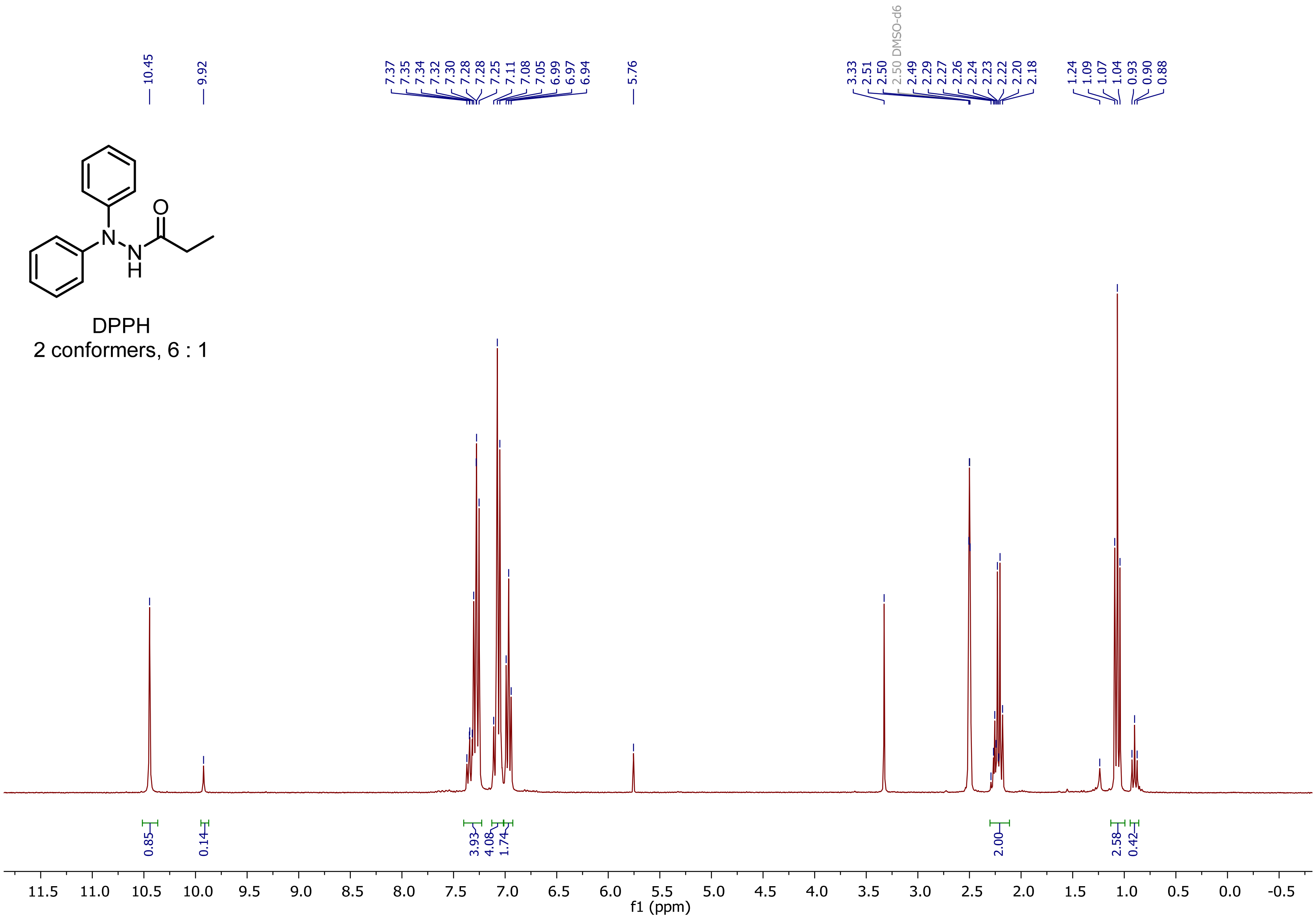

DPPH
2 conformers, 6 : 1

^{1}H NMR, $CDCl_3$, 298K

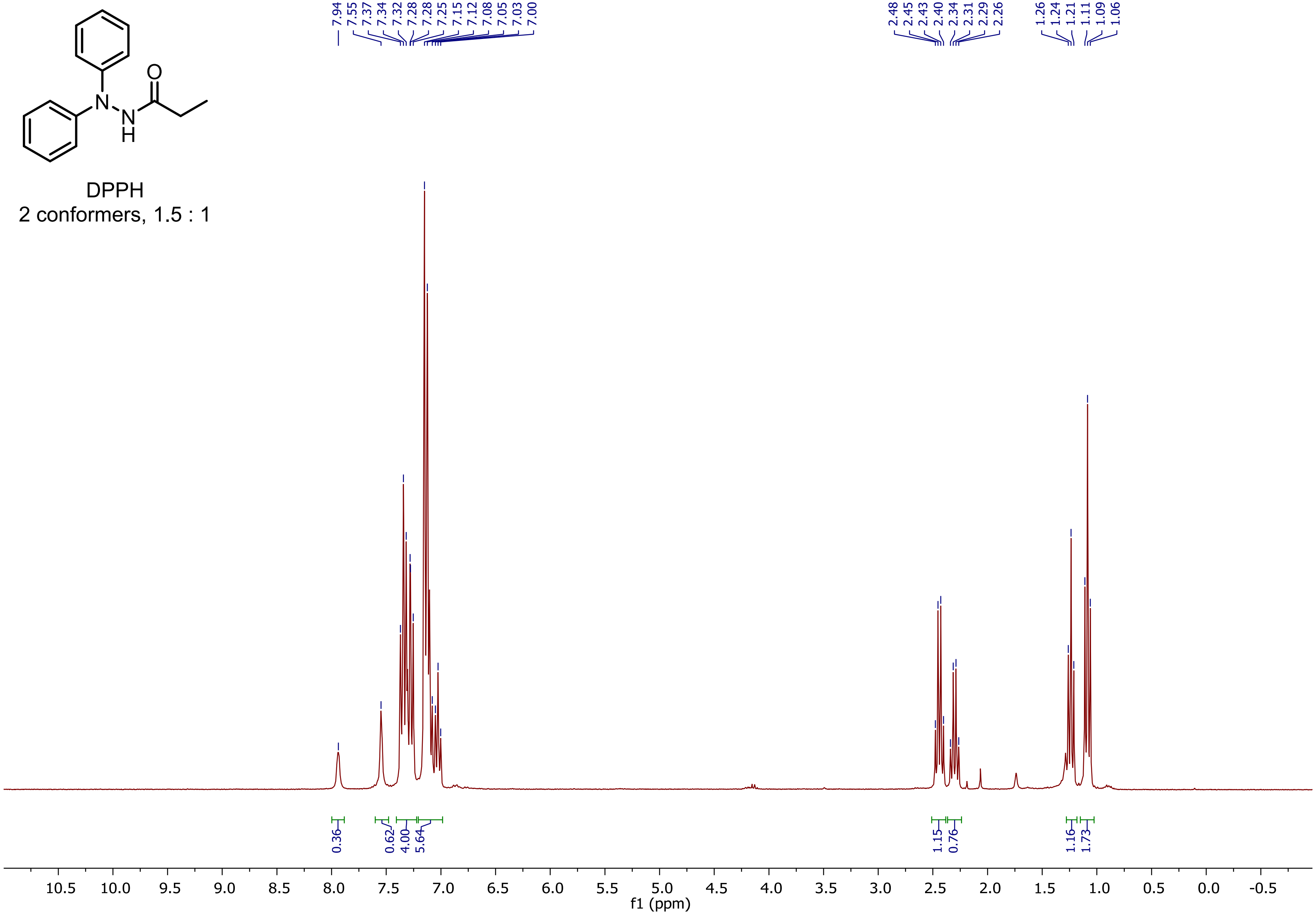

^{13}C NMR, DMSO-d_6, 298K

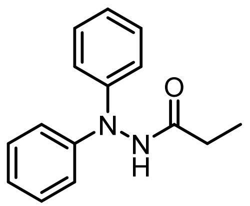

DPPH
2 conformers, 6 : 1

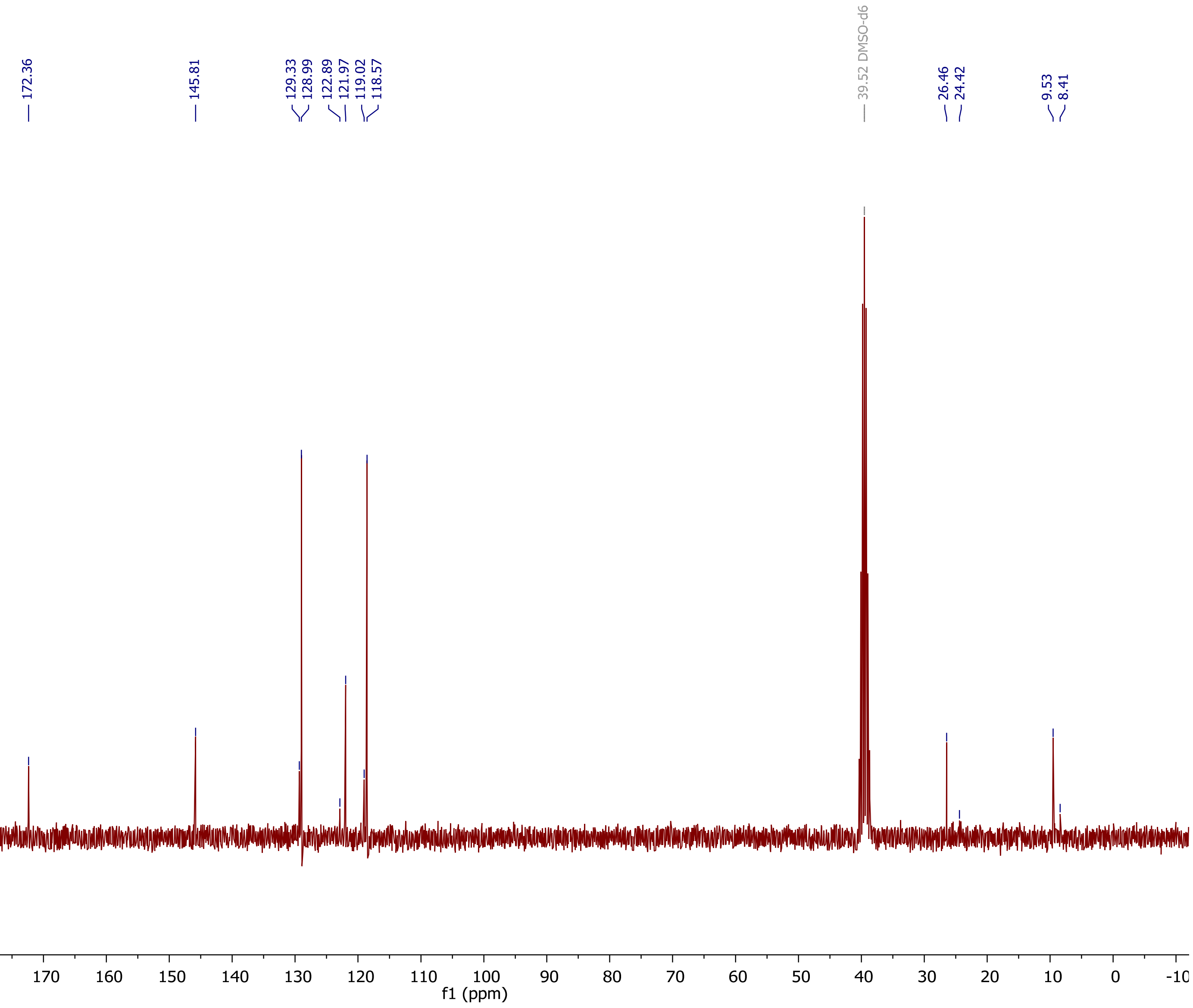

^{1}H-^{13}C HSQC, DMSO-d_6, 298K

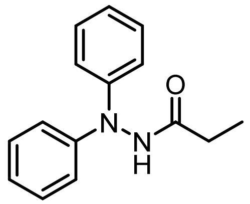

DPPH
2 conformers, 6 : 1

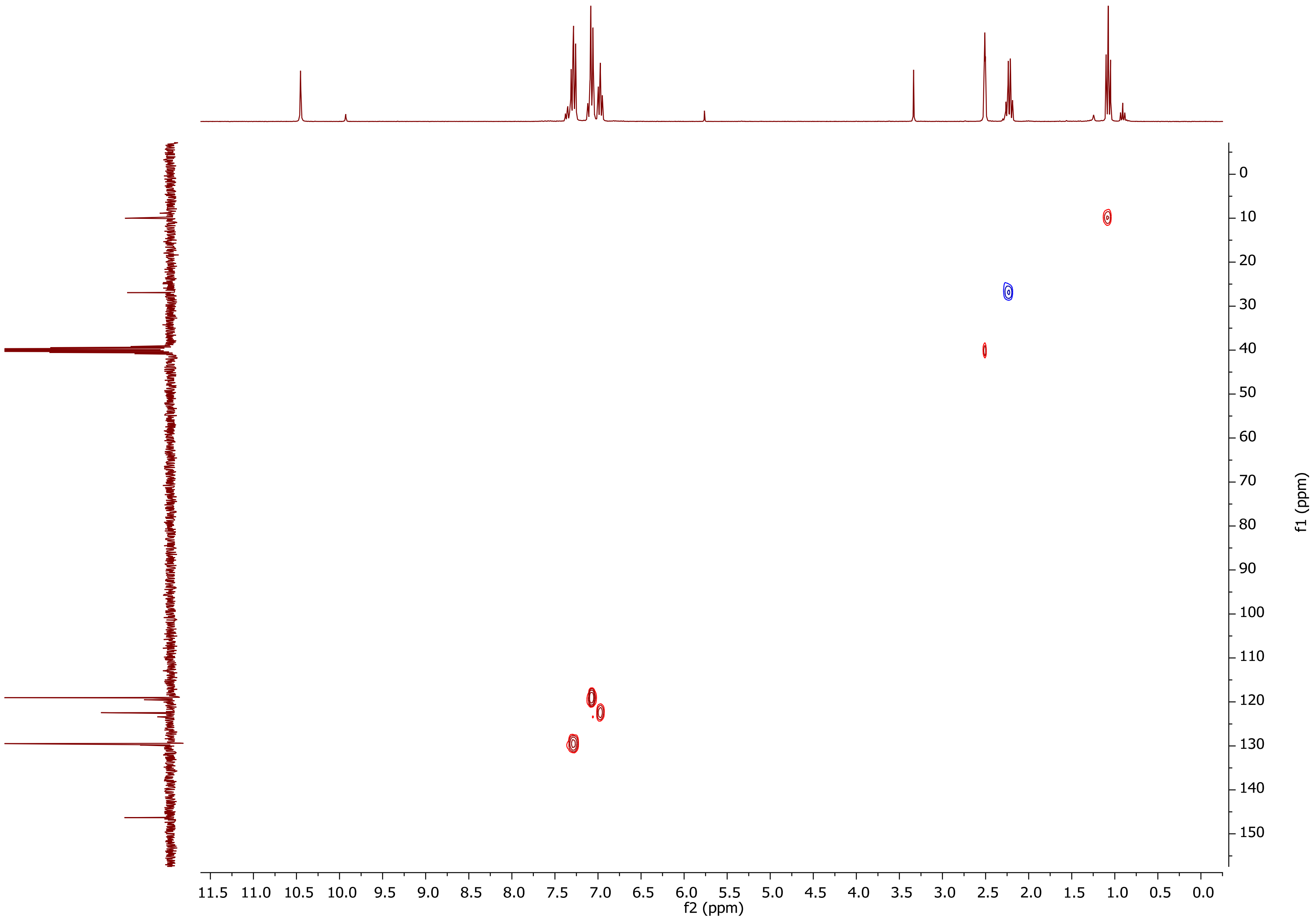

^1H NMR, $CDCl_3$, 298K

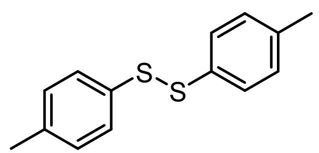

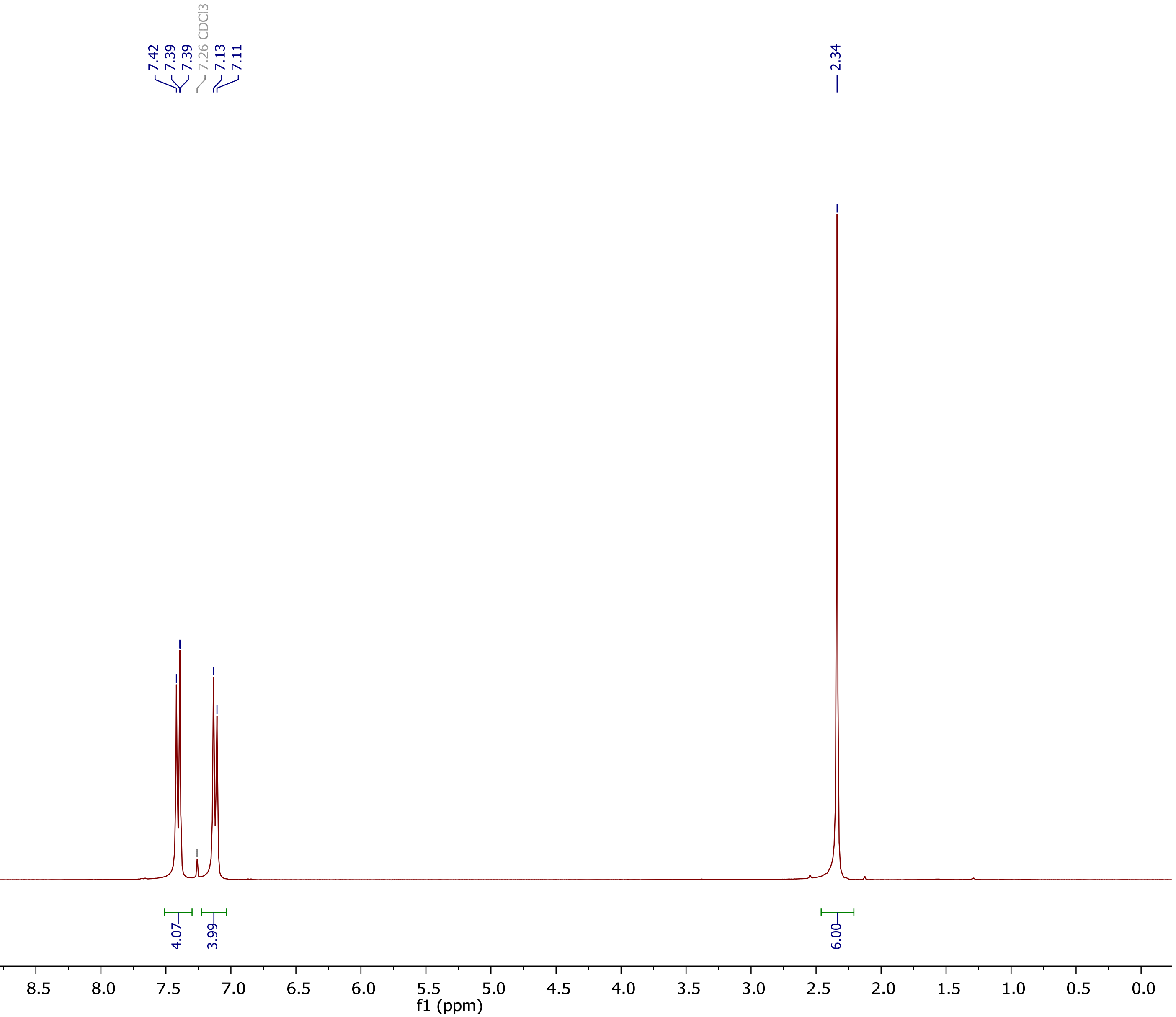

^{13}C NMR, $CDCl_3$, 298K

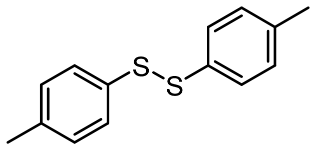

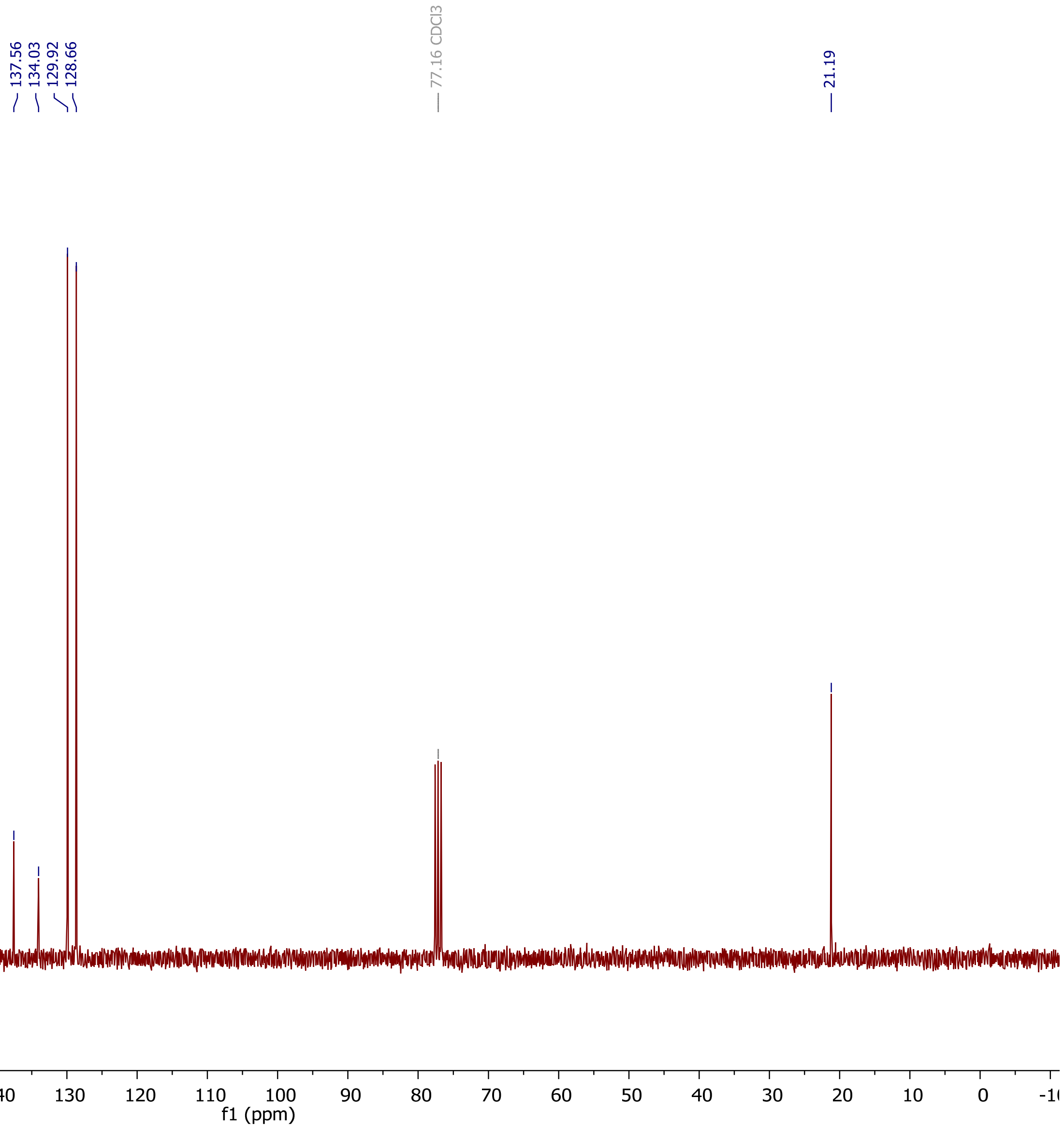

1.11 Cyclic voltammetry analysis

General procedure for CV measurements. Cyclic voltammetry (CV) experiments were performed for dimethylsulfoxide solutions with 0.1 M tetrabutylammonium hexafluorophosphate as a supporting electrolyte using a Metrohm Autolab PGSTAT128N potentiostat with a conventional one-compartment three-electrode cell (5 mL of solution) and 100 mV/s scan rate. The Pt disk electrode, which was used as a working electrode, was thoroughly polished with a 0.05 µm alumina slurry, sonicated for two minutes in deionized water and rinsed before every measurement. The Pt disk counter electrode and a platinum wire reference electrode were used. After ohmic drop compensation with internal software a drift of the reference electrode was dismissed, but for confidence ferrocene was added after the measurements as an internal standard. All the potentials are reported relative to the Pt wire ref. electrode. Fc/Fc^{+} couple was in all cases $E_{1/2}$ = 0.32 V (0.1 V vs Ag/AgCl). The solutions were thoroughly deaerated by passing argon through them before the CV experiments and above these solutions during the measurements.

CV measurements were performed for complexes $M(cyclam(OH)_4)X_2$ prepared as described above. Complexes of cyclam used for CV measurements were prepared according to the following procedure: a solution of corresponding d-metal salt ($Ni(NO_3)_2{\times}6H_2O$, $CuCl_2{\times}2H_2O$ or $ZnCl_2$, 1 equiv.) in MeOH (c = 50 mM) was added to a solution of cyclam (1 equiv.) in MeOH (c = 25 mM). The resulting mixture was kelp for 30 minutes at rt. Then, the volatiles were removed under reduced pressure and the solid residue was dried in a vacuum (ca. 0.5 Torr) until constant weight to give the corresponding complex $M(cyclam)X_2$, which was used without further purification.

The results are shown in Supplementary Figs. 15–25.

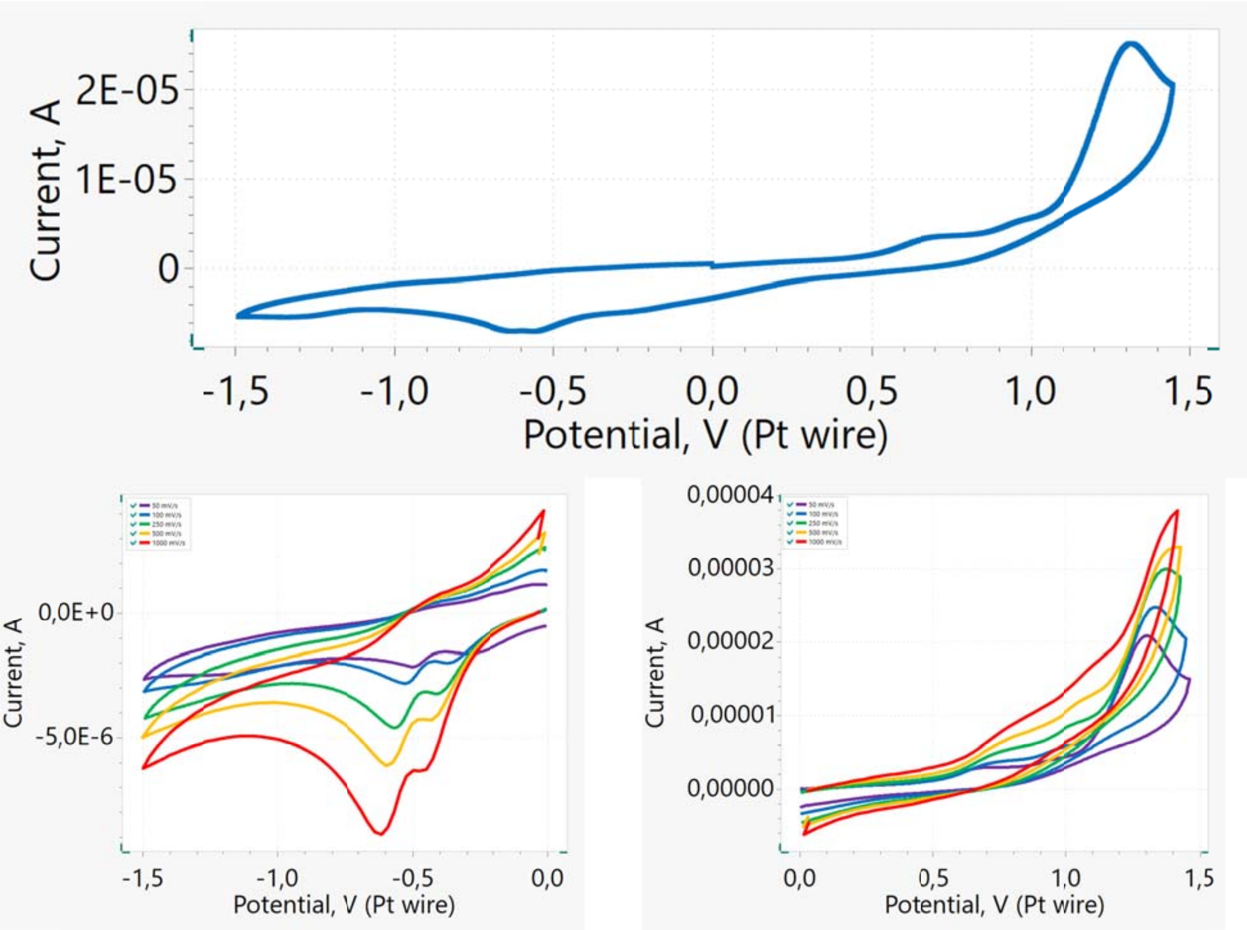

Supplementary Fig. 15. CV for cyclam(OH)$_4$ in 0.05 M TBAPF$_6$ solution in DMSO with Pt disk working and counter electrodes, Pt wire ref. electrode. Common view performed at 100 mV/s scan rate. Colors of lines: red – 1000 mV/s, yellow – 500 mV/s, green – 250 mV/s, blue – 100 mV/s, violet – 50 mV/s.

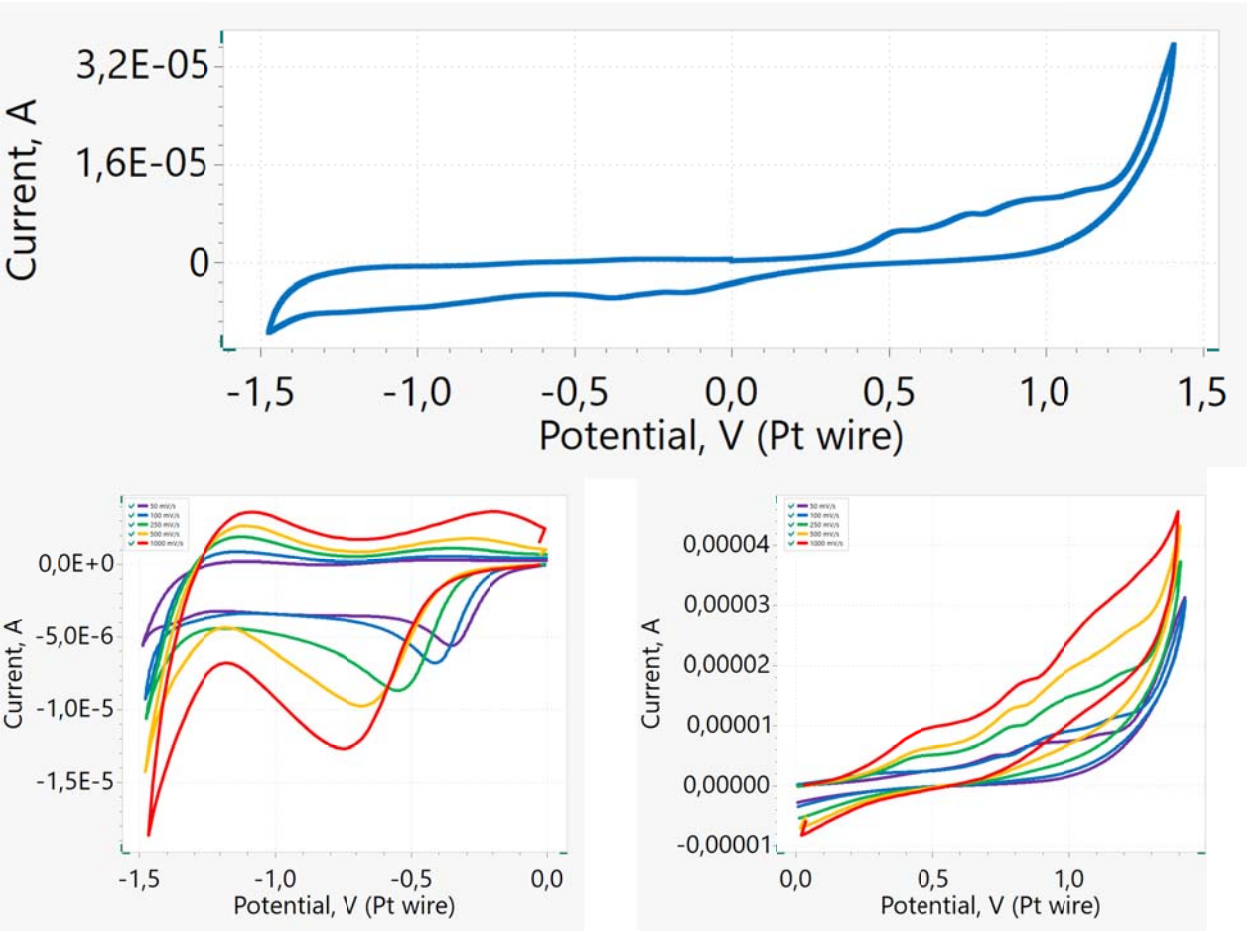

Supplementary Fig. 16. CV for cyclam(OH)$_4$ in 0.05 M TBAPF$_6$ and 4 equiv. of NaHCO$_3$ solution in DMSO with Pt disk working and counter electrodes, Pt wire ref. electrode. Common view performed at 100 mV/s scan rate. Colors of lines: red – 1000 mV/s, yellow – 500 mV/s, green – 250 mV/s, blue – 100 mV/s, violet – 50 mV/s.

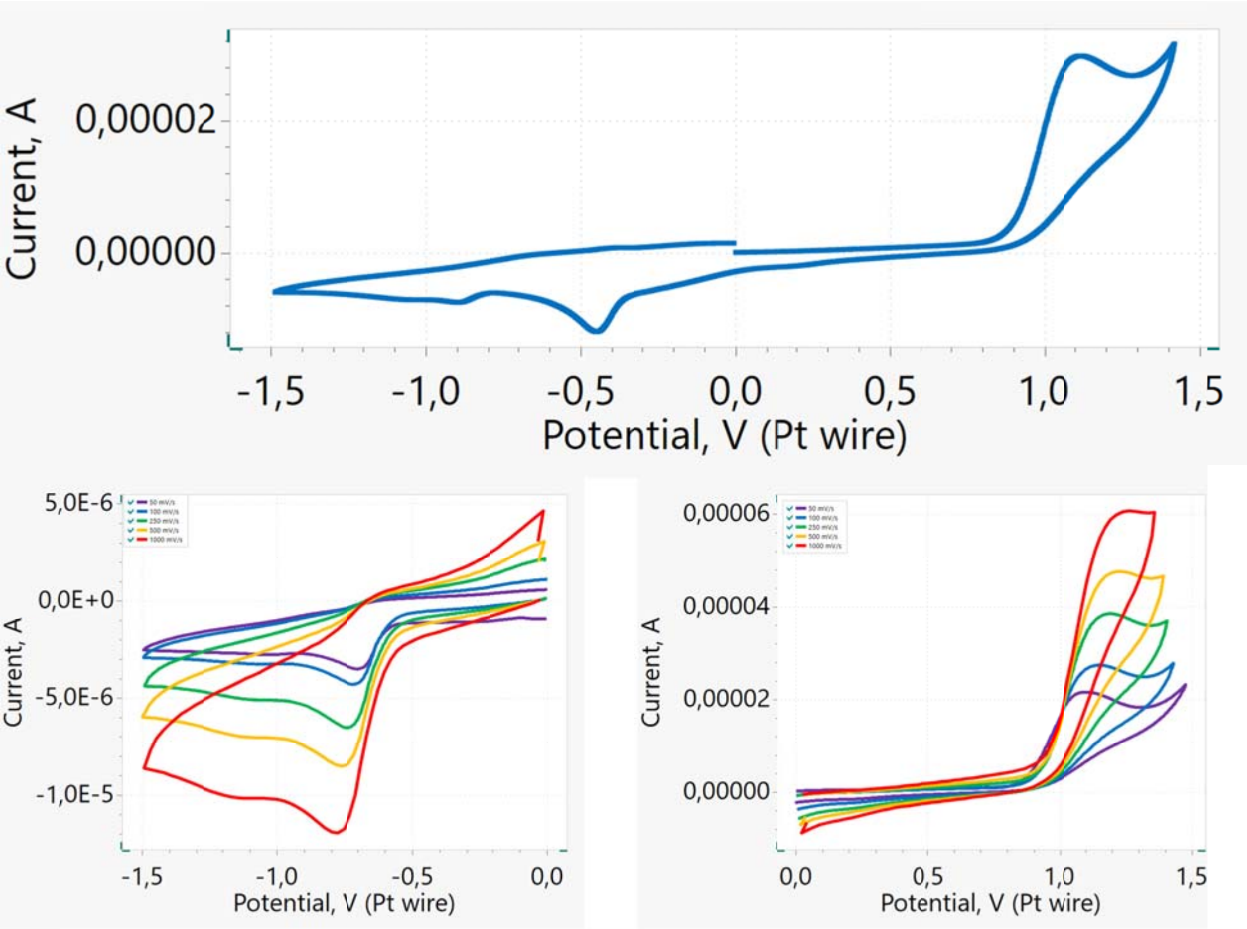

Supplementary Fig. 17. CV for Cu(cyclam)Cl_2 in 0.1 M $TBAPF_6$ solution in DMSO with Pt disk working and counter electrodes, Pt wire ref. electrode. Common view performed at 100 mV/s scan rate. Colors of lines: red – 1000 mV/s, yellow – 500 mV/s, green – 250 mV/s, blue – 100 mV/s, violet – 50 mV/s.

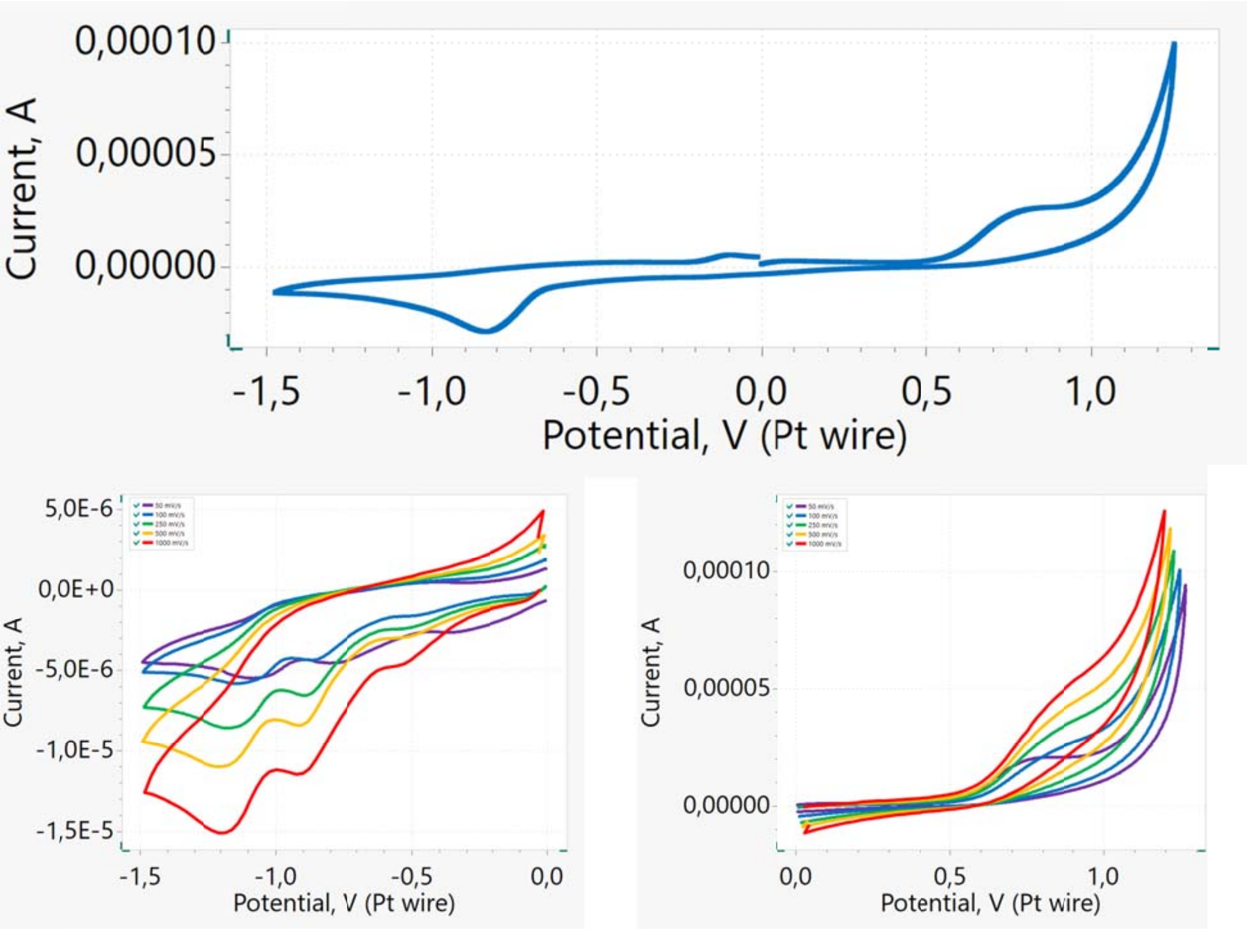

Supplementary Fig. 18. CV for $Cu(cyclam(OH)_4)Cl_2$ in 0.1 M $TBAPF_6$ solution in DMSO with Pt disk working and counter electrodes, Pt wire ref. electrode. Common view performed at 100 mV/s scan rate. Colors of lines: red – 1000 mV/s, yellow – 500 mV/s, green – 250 mV/s, blue – 100 mV/s, violet – 50 mV/s.

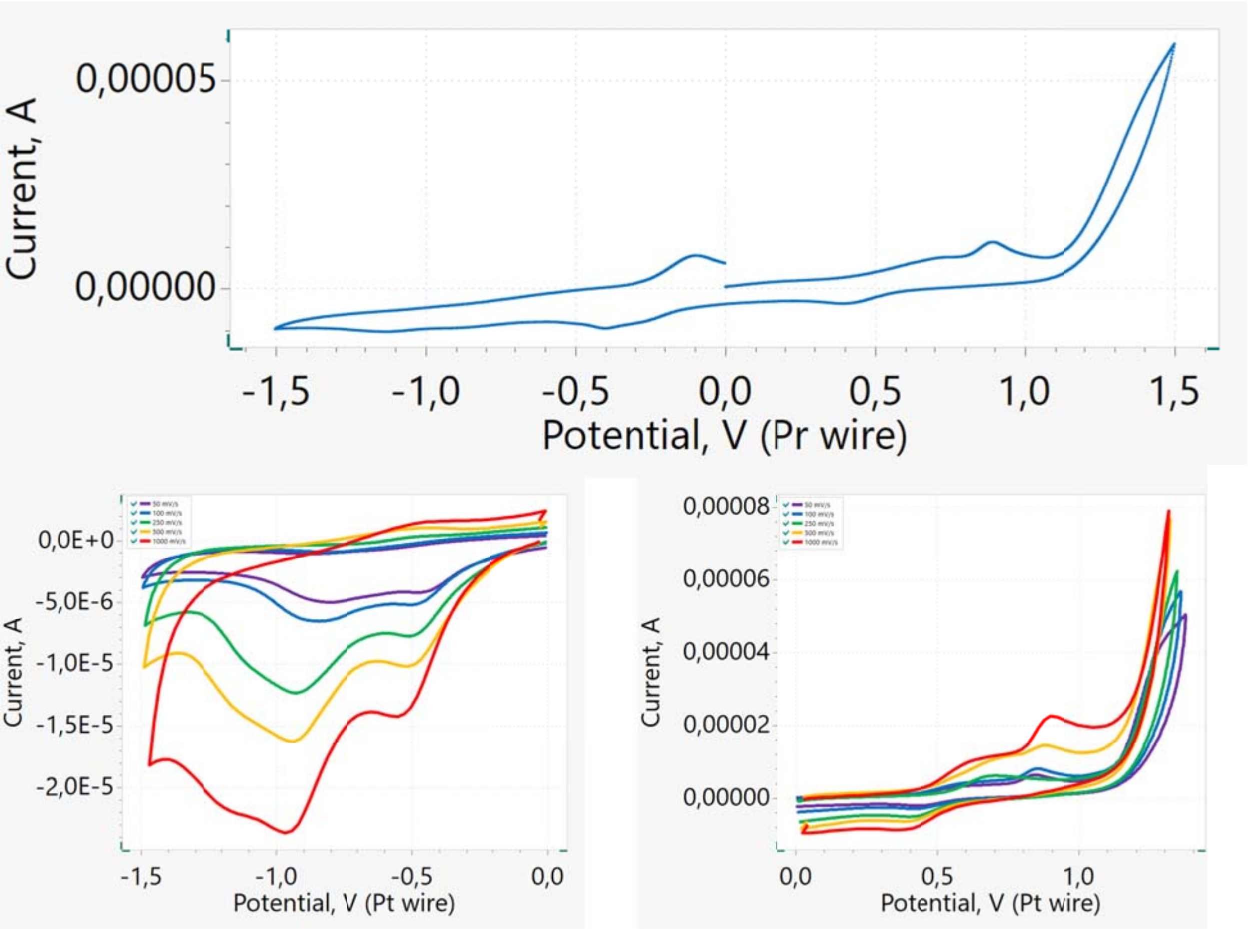

Supplementary Fig. 19. CV for Cu(cyclam(OH)$_4$)Cl$_2$ in 0.1 M TBAPF$_6$ and 4 equiv. of NaHCO$_3$ solution in DMSO with Pt disk working and counter electrodes, Pt wire ref. electrode. Common view performed at 100 mV/s scan rate. Colors of lines: red – 1000 mV/s, yellow – 500 mV/s, green – 250 mV/s, blue – 100 mV/s, violet – 50 mV/s.

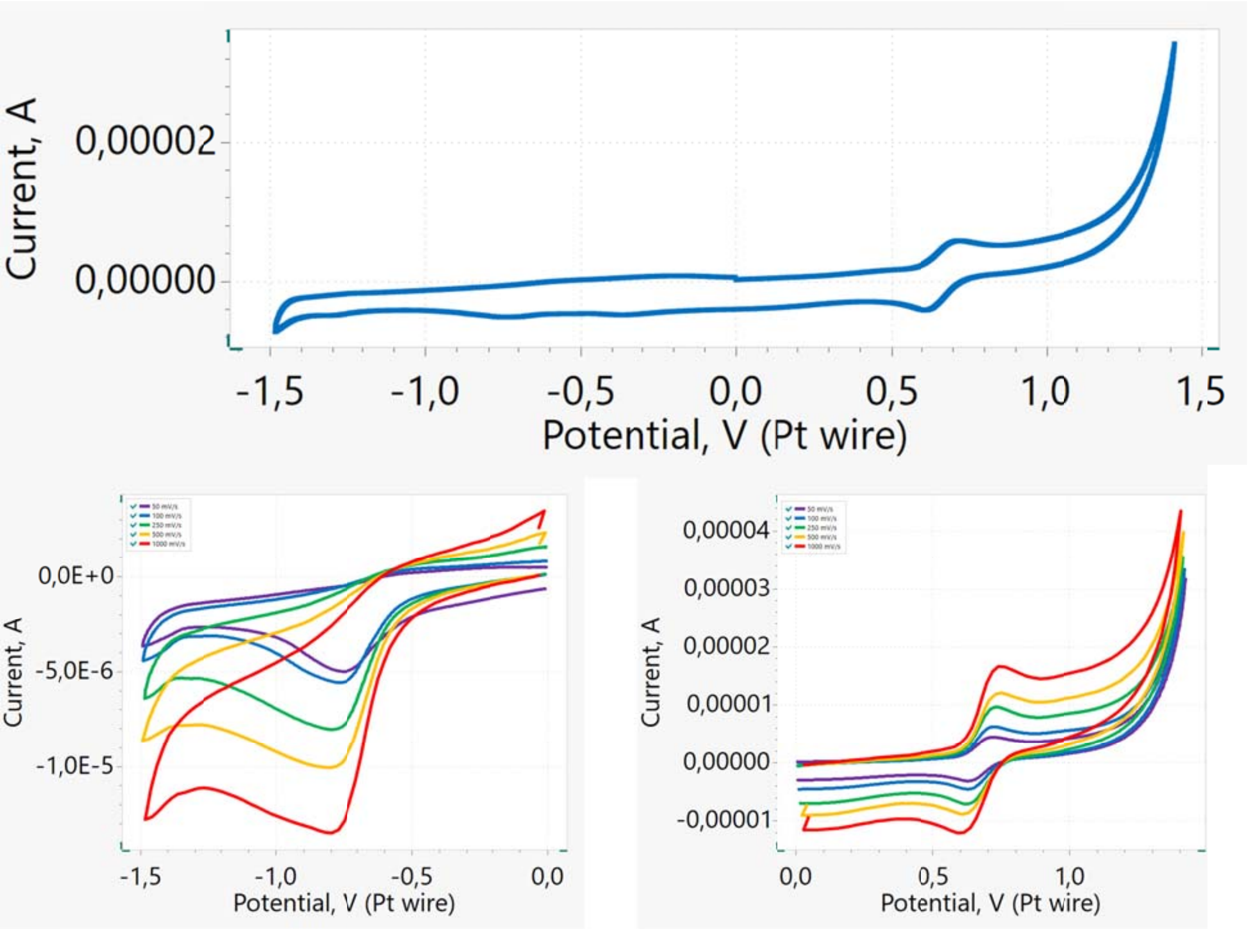

Supplementary Fig. 20. CV for Ni(cyclam)$(ClO_4)_2$ in 0.1 M $TBAPF_6$ solution in DMSO with Pt disk working and counter electrodes, Pt wire ref. electrode. Common view performed at 100 mV/s scan rate. Colors of lines: red – 1000 mV/s, yellow – 500 mV/s, green – 250 mV/s, blue – 100 mV/s, violet – 50 mV/s.

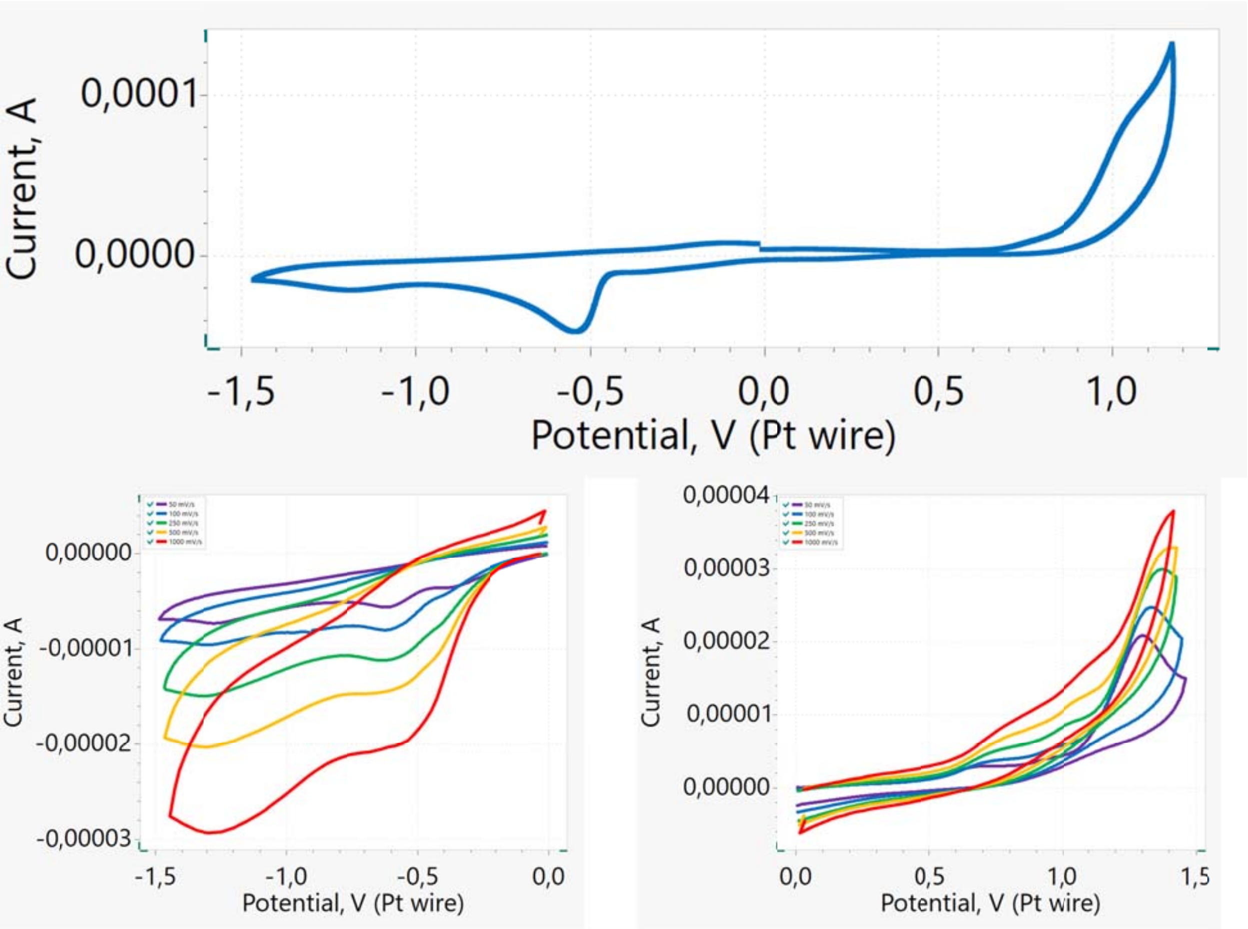

Supplementary Fig. 21. CV for Ni(cyclam(OH)$_4$)(ClO_4)$_2$ in 0.1 M $TBAPF_6$ solution in DMSO with Pt disk working and counter electrodes, Pt wire ref. electrode. Common view performed at 100 mV/s scan rate. Colors of lines: red – 1000 mV/s, yellow – 500 mV/s, green – 250 mV/s, blue – 100 mV/s, violet – 50 mV/s.

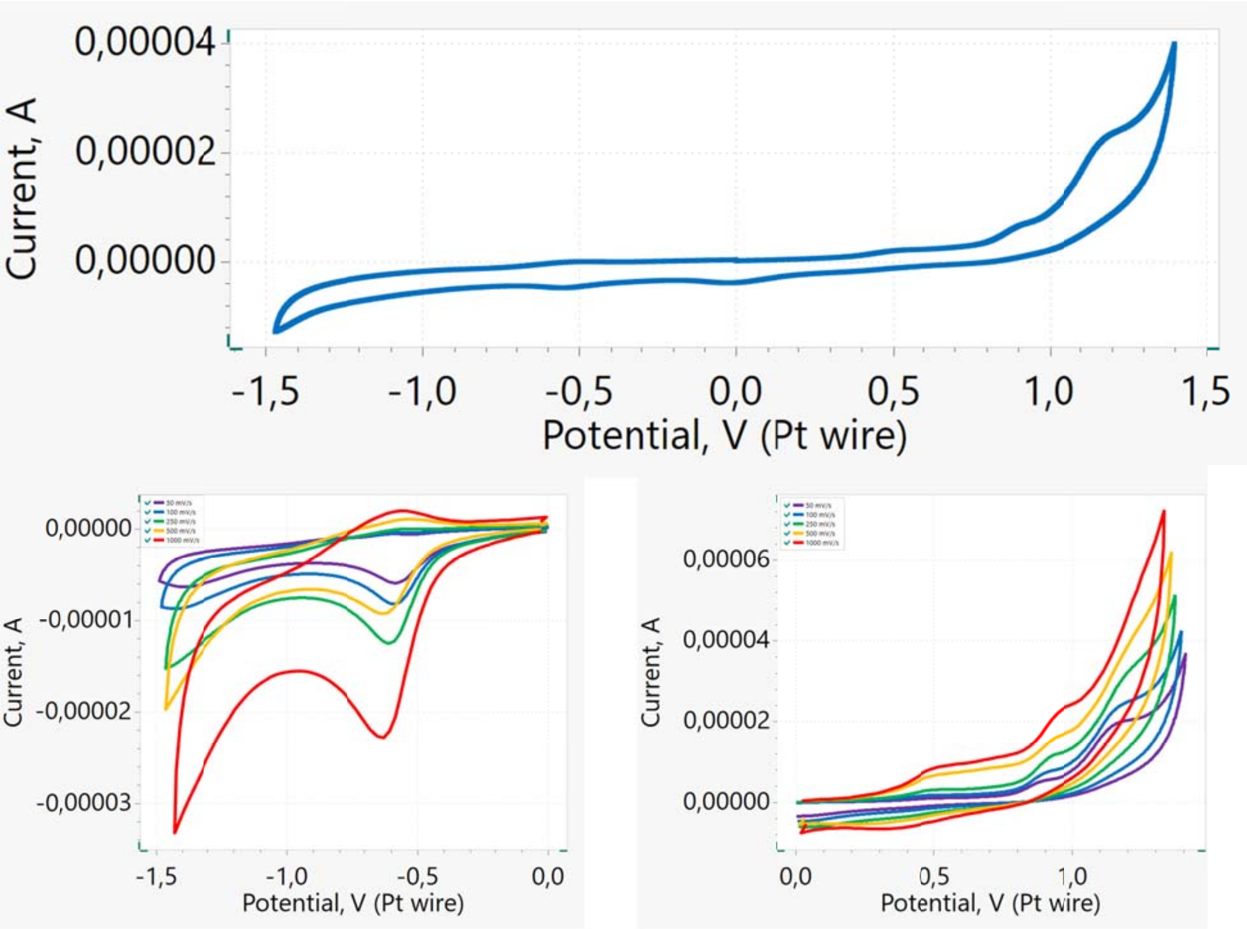

Supplementary Fig. 22. CV for Ni(cyclam(OH)$_4$)(ClO$_4$)$_2$ in 0.1 M TBAPF$_6$ and 4 equiv. of $NaHCO_3$ solution in DMSO with Pt disk working and counter electrodes, Pt wire ref. electrode. Common view performed at 100 mV/s scan rate. Colors of lines: red – 1000 mV/s, yellow – 500 mV/s, green – 250 mV/s, blue – 100 mV/s, violet – 50 mV/s.

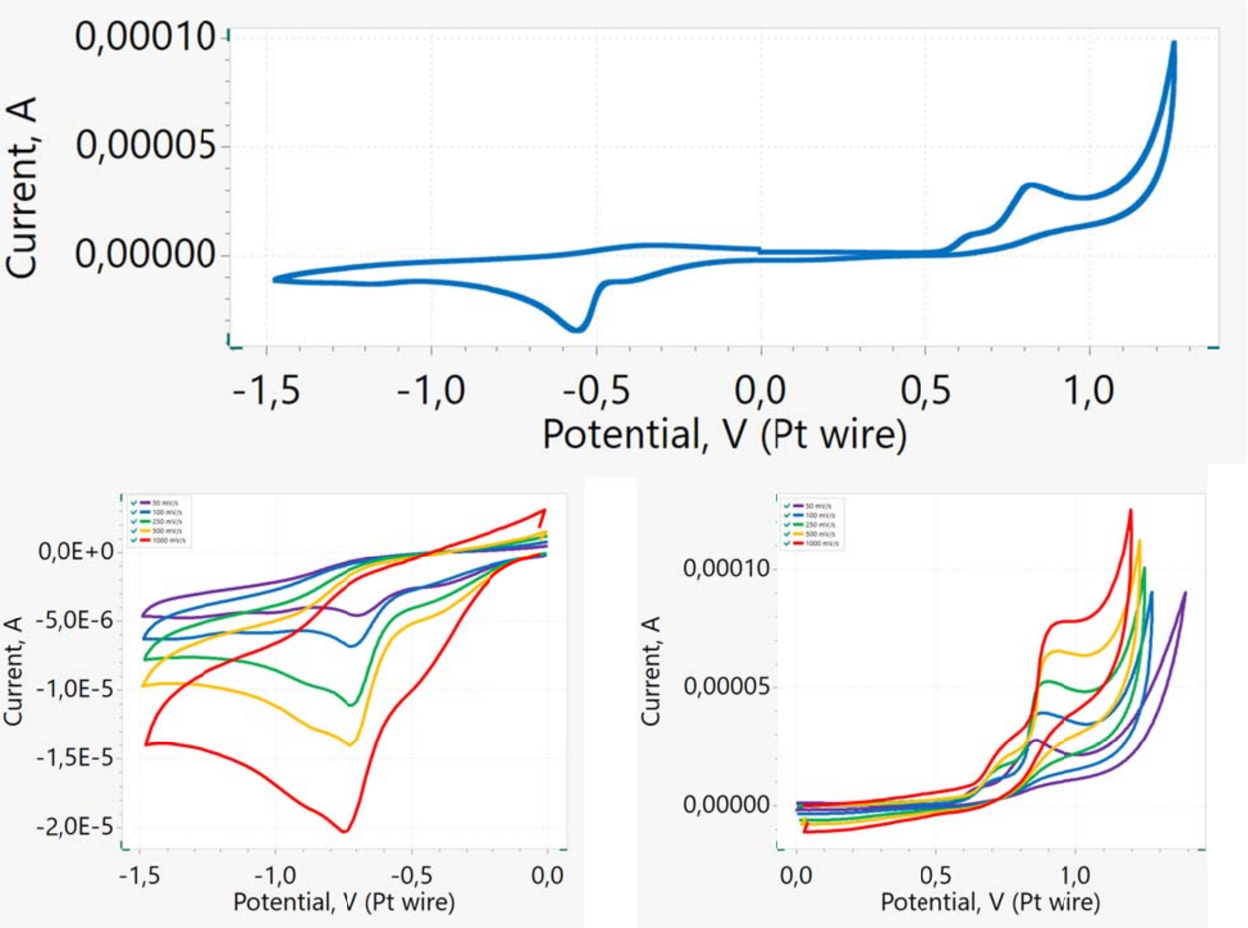

Supplementary Fig. 23. CV for Mn(cyclam(OH)$_4$)Br$_2$ in 0.1 M TBAPF$_6$ solution in DMSO with Pt disk working and counter electrodes, Pt wire ref. electrode. Common view performed at 100 mV/s scan rate. Colors of lines: red – 1000 mV/s, yellow – 500 mV/s, green – 250 mV/s, blue – 100 mV/s, violet – 50 mV/s.

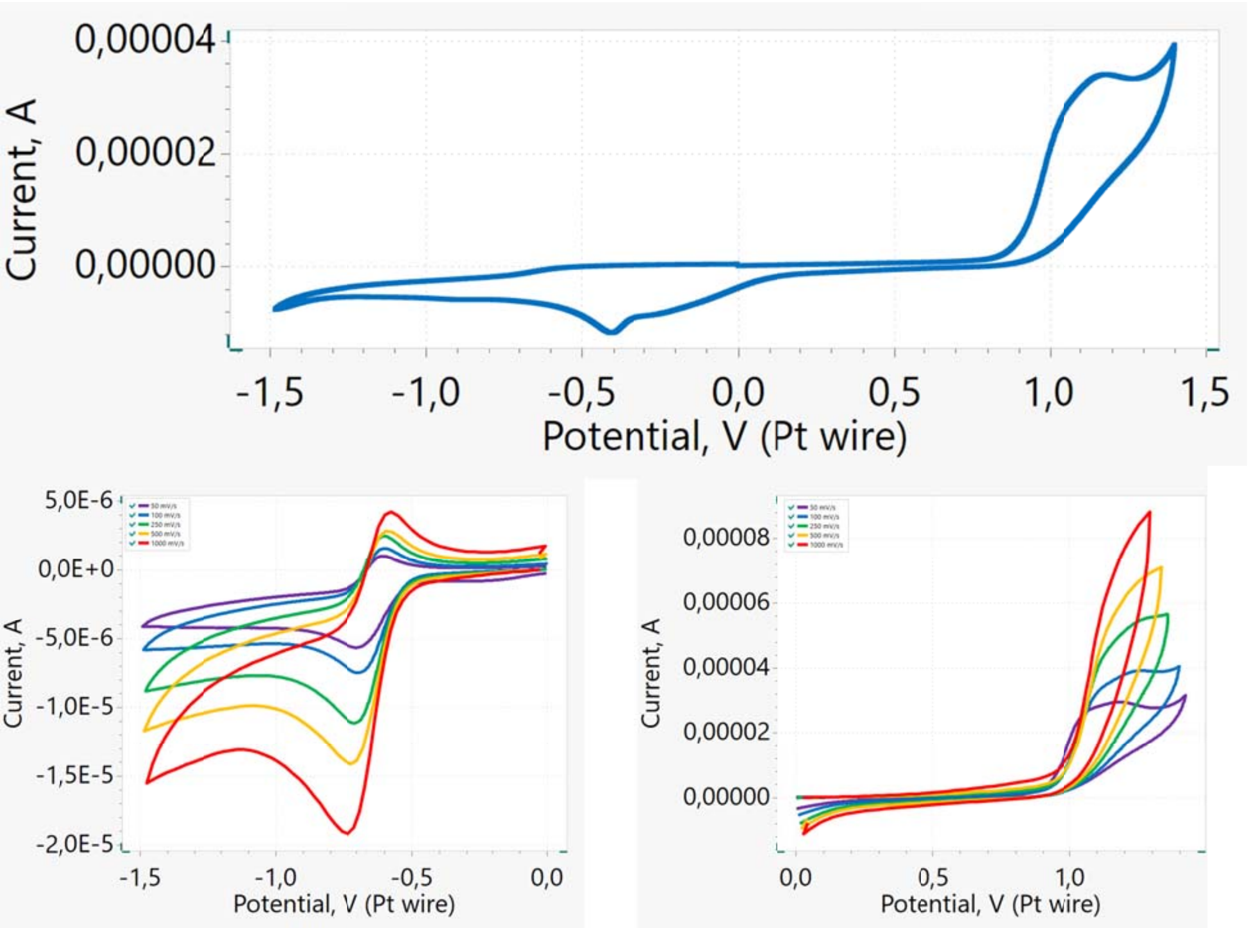

Supplementary Fig. 24. CV for Zn(cyclam)Cl_2 in 0.1 M $TBAPF_6$ solution in DMSO with Pt disk working and counter electrodes, Pt wire ref. electrode. Common view performed at 100 mV/s scan rate. Colors of lines: red – 1000 mV/s, yellow – 500 mV/s, green – 250 mV/s, blue – 100 mV/s, violet – 50 mV/s.

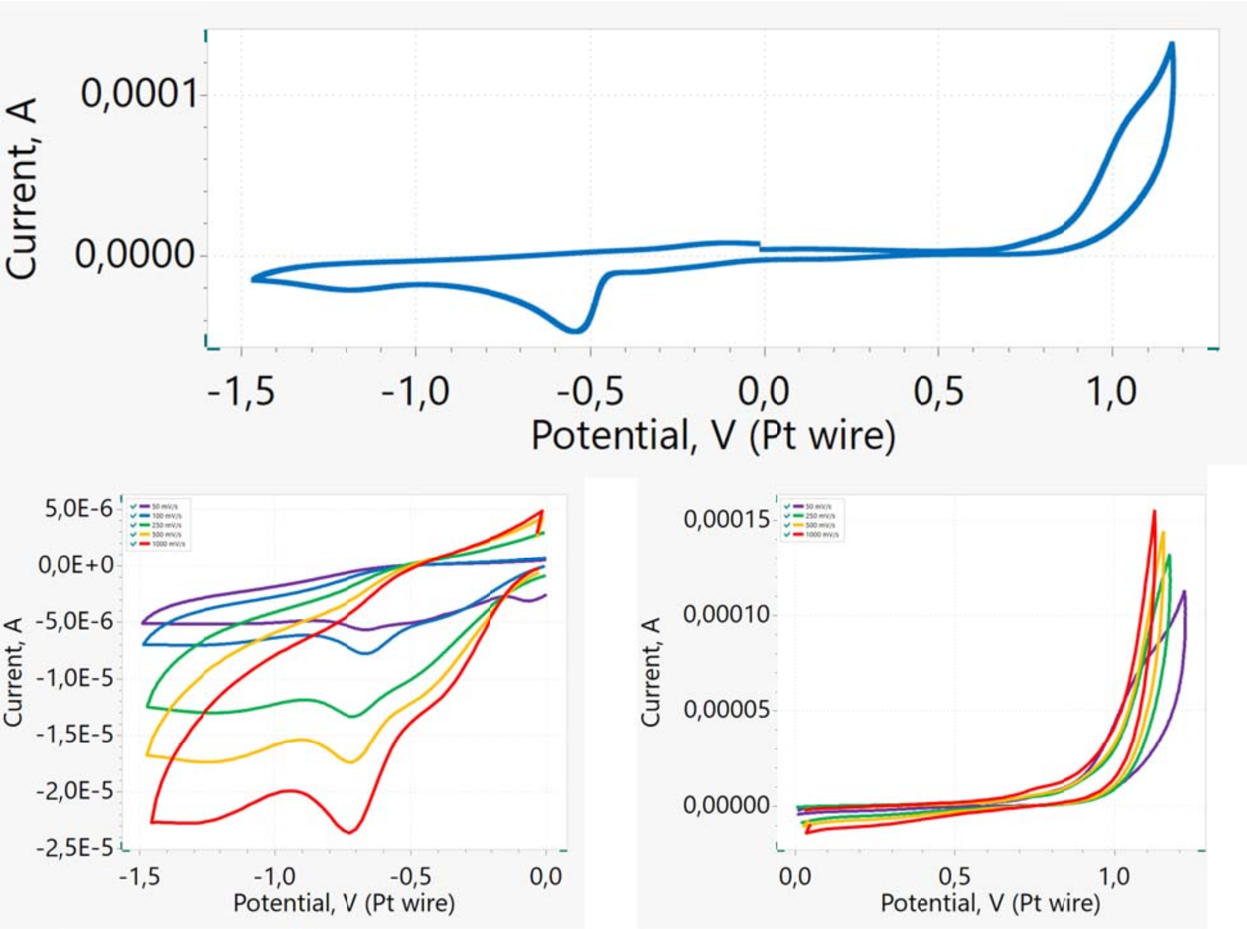

Supplementary Fig. 25. CV for Zn(cyclam(OH)$_4$)Cl$_2$ in 0.1 M TBAPF$_6$ solution in DMSO with Pt disk working and counter electrodes, Pt wire ref. electrode. Common view performed at 100 mV/s scan rate. Colors of lines: red – 1000 mV/s, yellow – 500 mV/s, green – 250 mV/s, blue – 100 mV/s, violet – 50 mV/s.

Electrochemical oxidation of cyclam(OH)$_4$

The electrochemical oxidation was conducted in a 50 mM TBAPF$_6$ solution in DMSO at a constant voltage of 1.5 V against a platinum wire with a total charge of 4 F/mol and 0.02 mmol of cyclam(OH)$_4$ at ambient temperature. The working and counter electrodes were made of glassy carbon and used with an undivided cell. The resulting mixture was evaporated and analyzed using ESI-HRMS.

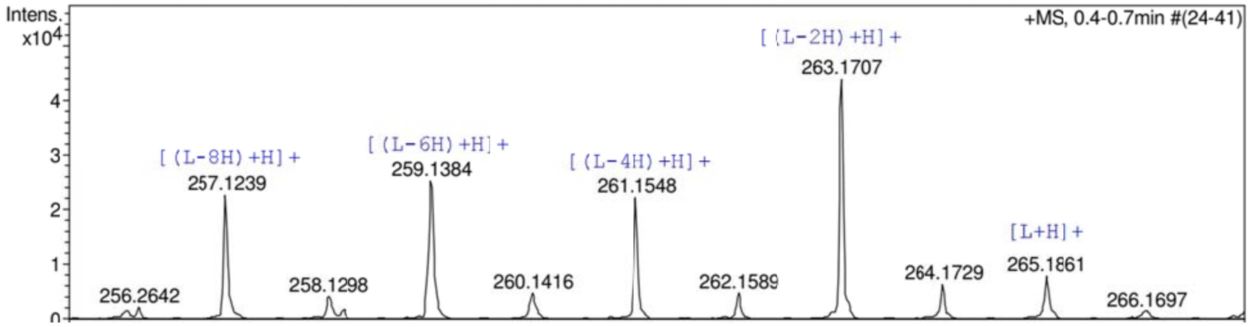

Chemical oxidation of cyclam(OH)$_4$

$AgClO_4$ (1 eq., 12 mg, 57 µmol) was added to a 25 mM methanol solution of cyclam(OH)$_4$ (15 mg, 57 µmol, 2.3 ml). The solution turned dark gray in color and a dark brown precipitate was formed. After 24 hours, the precipitate was centrifuged off and washed twice with a MeOH/water mixture (2:1). The combined solutions were concentrated under reduced pressure and analyzed by ^{1}H (D_2O) and HSQC NMR and ESI-HRMS.

^1H NMR of oxidized cyclam(OH)$_4$ in D_2O

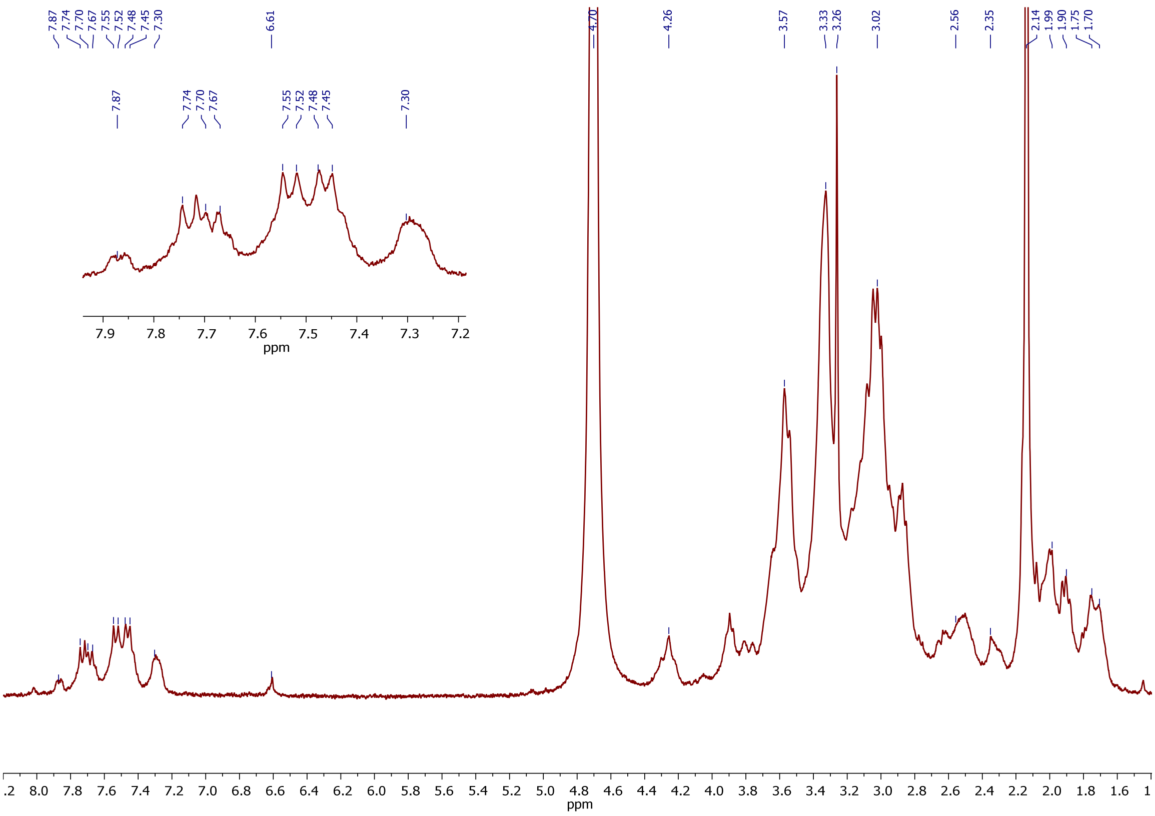

^1H-^{13}C HSQC of oxidized cyclam(OH)$_4$ in D_2O

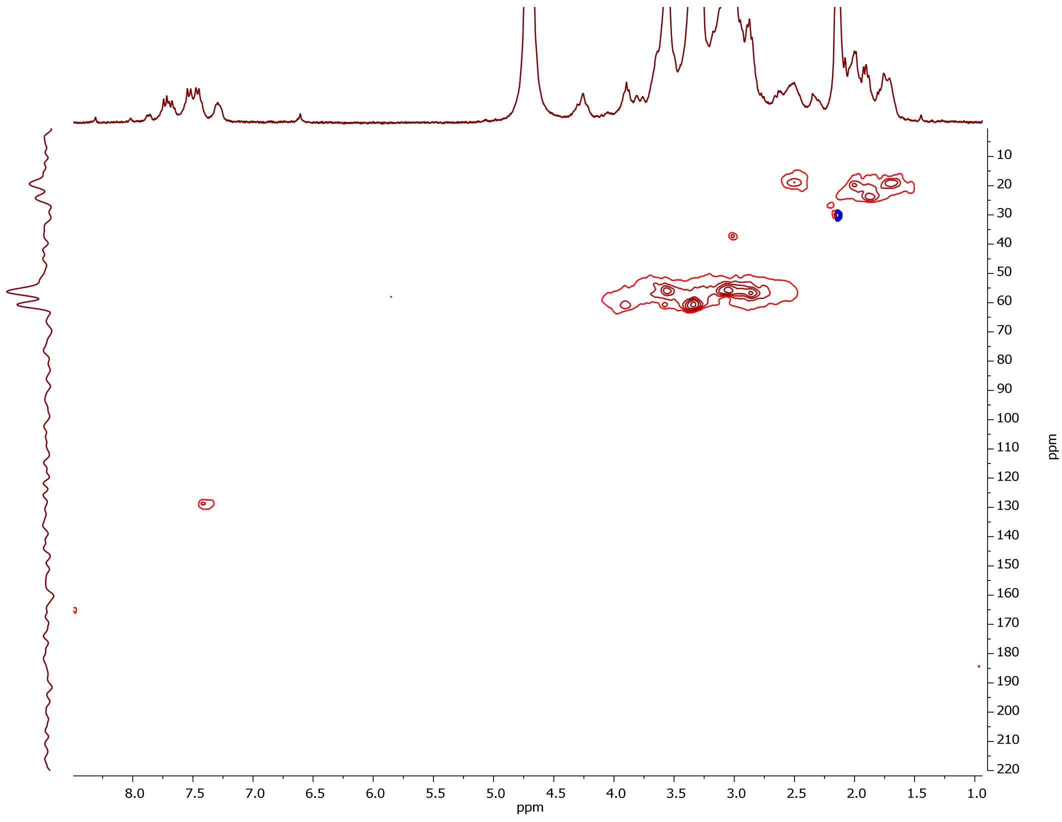

1.12 HRMS studies of metal–cyclam(OH)$_4$ complexes

HRMS of Cu(cyclam(OH)$_4$)Cl$_2$

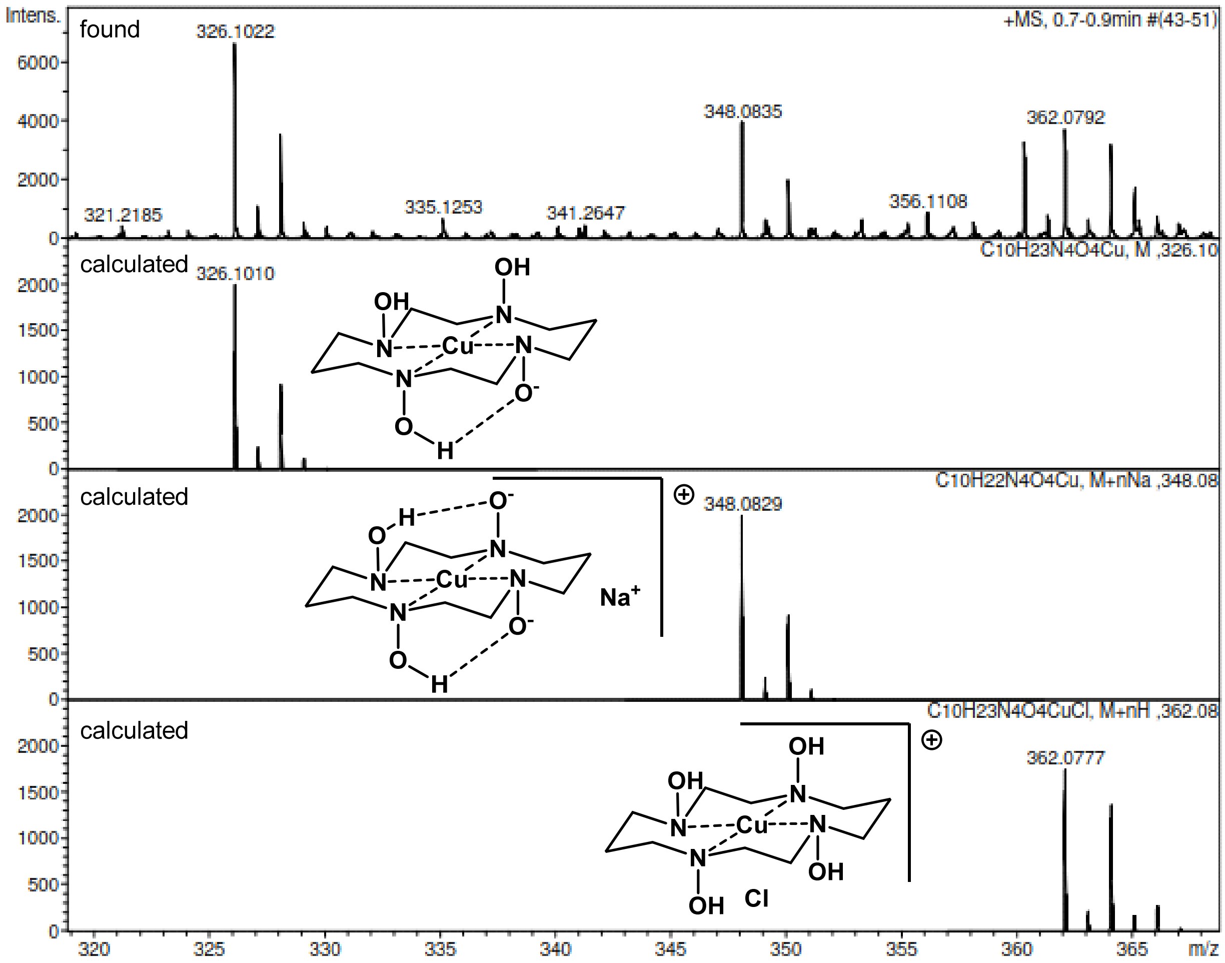

HRMS of Ni(cyclam(OH)$_4$)(NO_3)$_2$

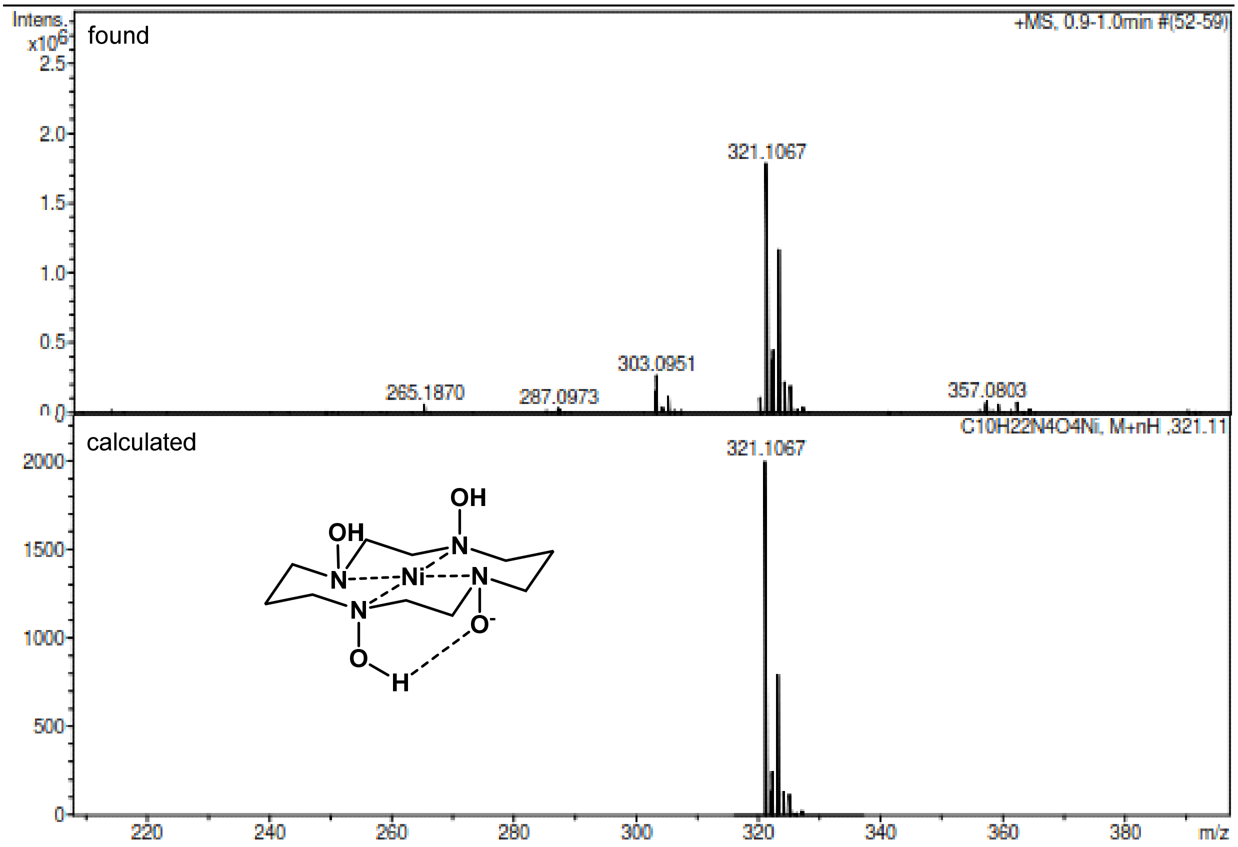

HRMS of Zn(cyclam(OH)$_4$)Cl$_2$

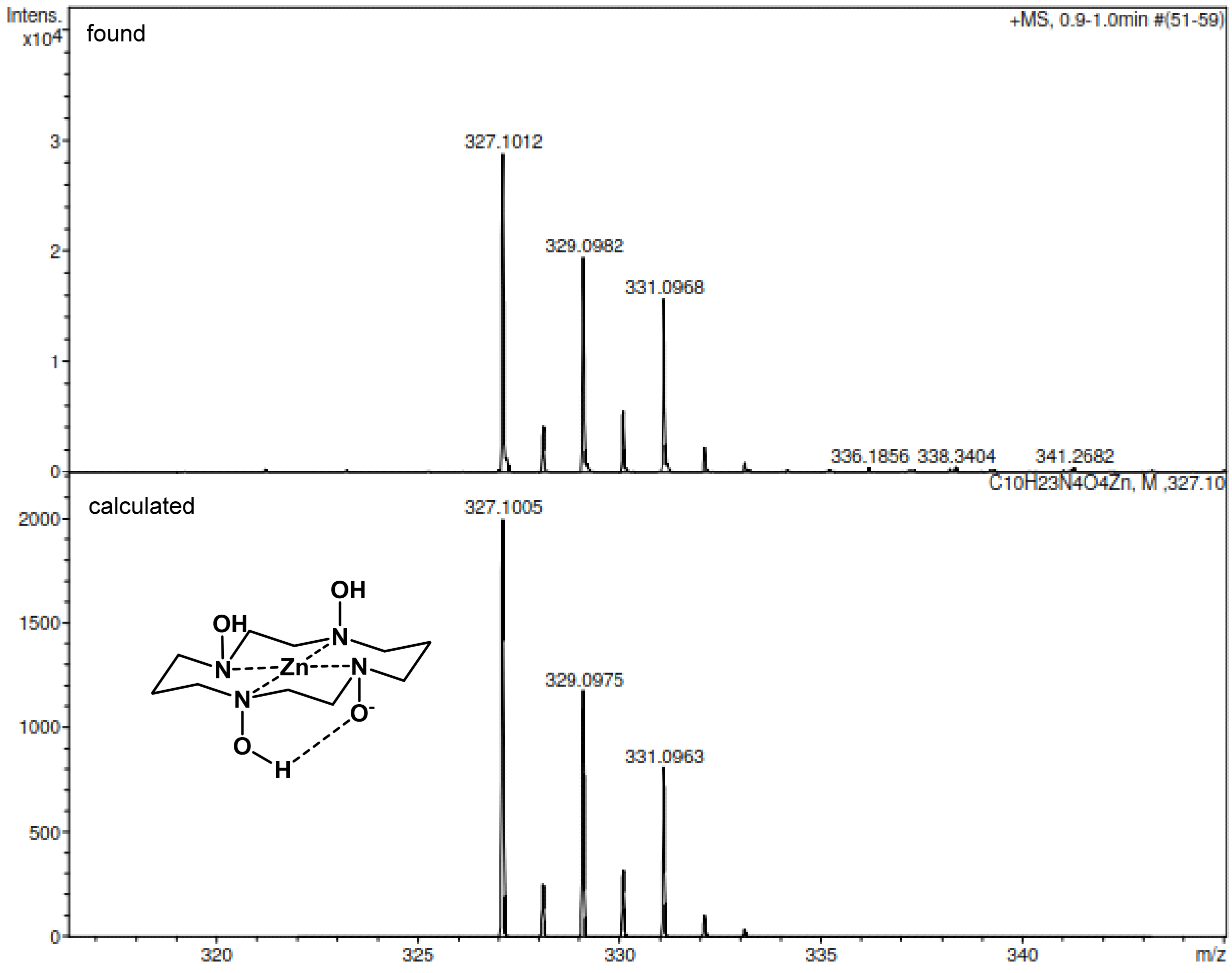

HRMS of Mn(cyclam(OH)$_4$)Cl$_2$

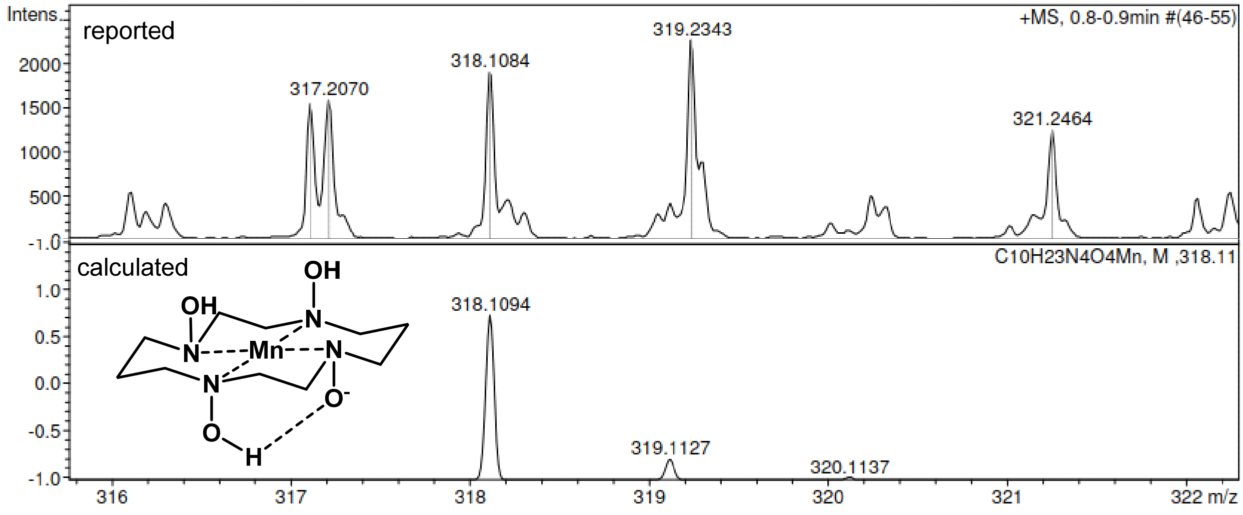

1.13 Competitive complexation of cyclam and cyclam(OH)$_4$ study

Procedure: Stock solutions of cyclam(OH)$_4$, cyclam and $CuCl_2$ were prepared as a 15 mM solutions in H_2O. Before each measurement stock solution of ligand and $CuCl_2$ were mixed in appropriate vessel, diluted with H_2O to a 5 mM concentration and kept for 15 minutes at ambient temperature. In a competitive complexation experiment, equal amounts of both ligands stock solutions were mixed in appropriate vessel, and then the same amount of stock solution of $CuCl_2$ was added. Measurements were performed after keeping the resulting mixtures for 15 minutes at ambient temperature. The results are given in Supplementary Figs 26–28.

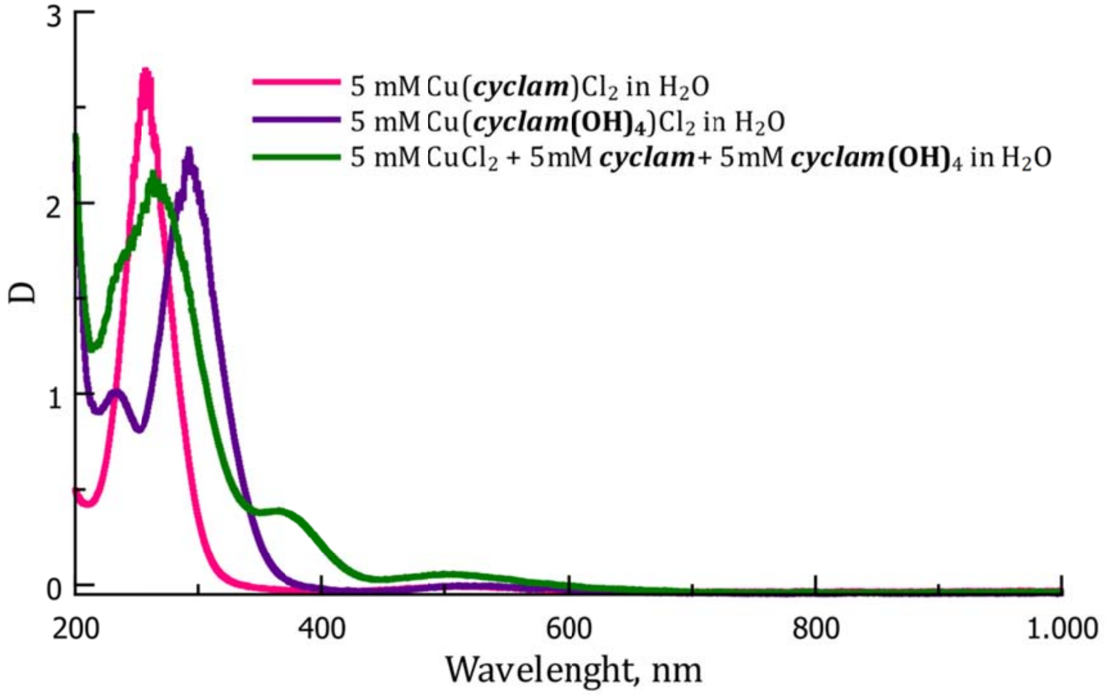

Supplementary Fig. 26. UV-Vis spectra of Cu(cyclam(OH)$_4$)Cl_2, Cu(cyclam)Cl_2 and competitive behavior of ligands relatively to $CuCl_2$ in H_2O at neutral pH.

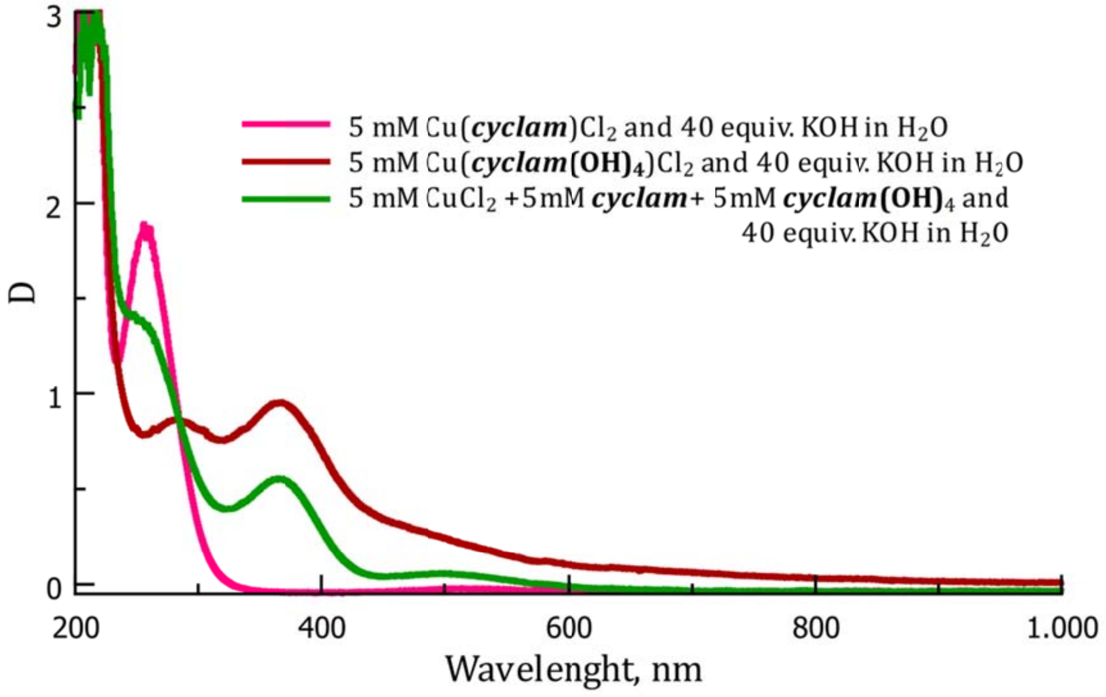

Supplementary Fig. 27. UV-Vis spectra of $Cu(cyclam(OH)_4)Cl_2$, $Cu(cyclam)Cl_2$ and competitive behavior of ligands relatively to $CuCl_2$ in H_2O with addition of KOH.

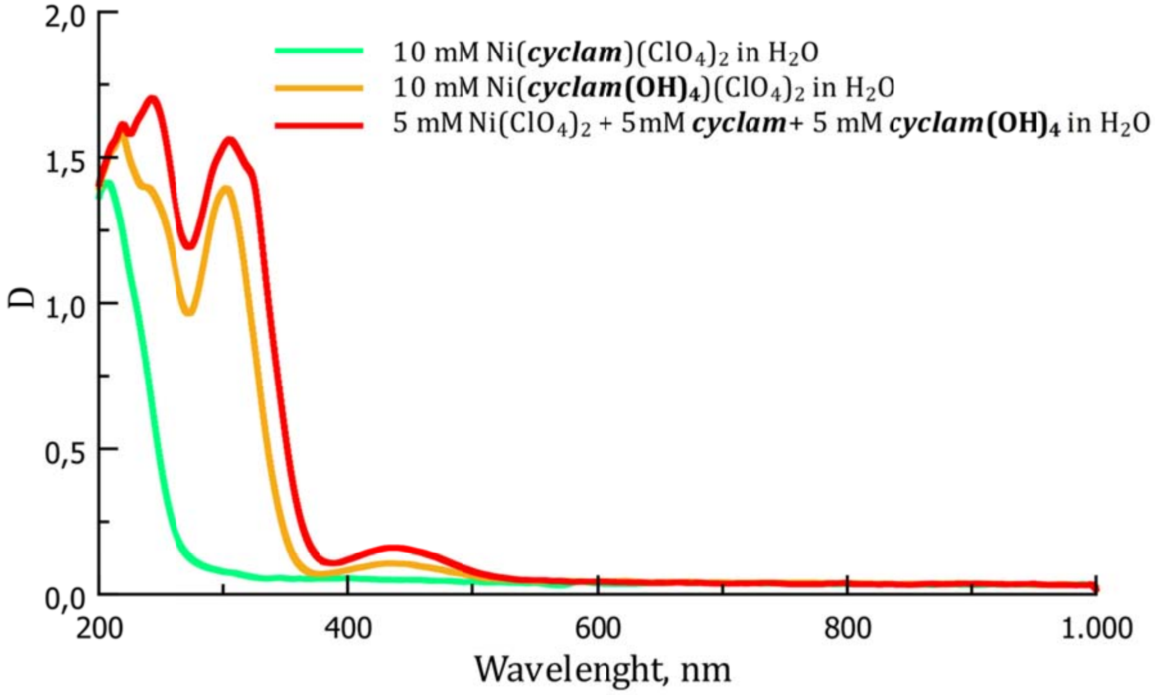

Supplementary Fig. 28. UV-Vis spectra of Ni(cyclam(OH)$_4$)(ClO$_4$)$_2$, Ni(cyclam)(ClO$_4$)$_2$ and competitive behavior of ligands relatively to Ni(ClO$_4$)$_2$ in H_2O at neutral pH.

1.14 Potentiometric pH titration study

Protonation and stability constant were determined by the Irving and Rossotti method[20] using aqueous solutions of all components. 0.002 M of “metal” and ligand, 0.01 M of HCl and 0.1 M of KOH initial concentrations were used in this experiments. The ionic strength of solutions was constant due to performing experiments in 0.01 M KCl solution. Experiments were performed for both cyclam and cyclam$(OH)_4$ ligands. Checker® (HI 98103) from HANNA® instruments was used for pH measurements with calibration related to standard solutions. All measurements were performed three times before convergent results. The titration curves are given in Supplementary Figures 29 and 30.

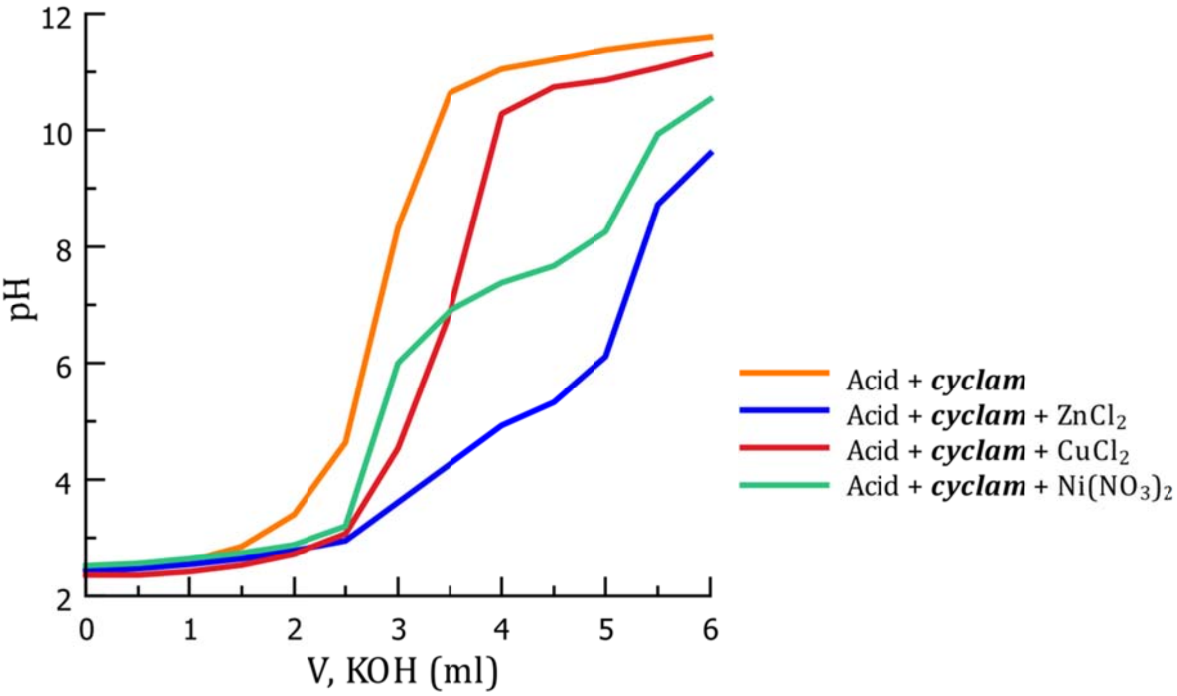

Supplementary Fig. 29. Titration curves for cyclam and its complexes (generated *in situ*).

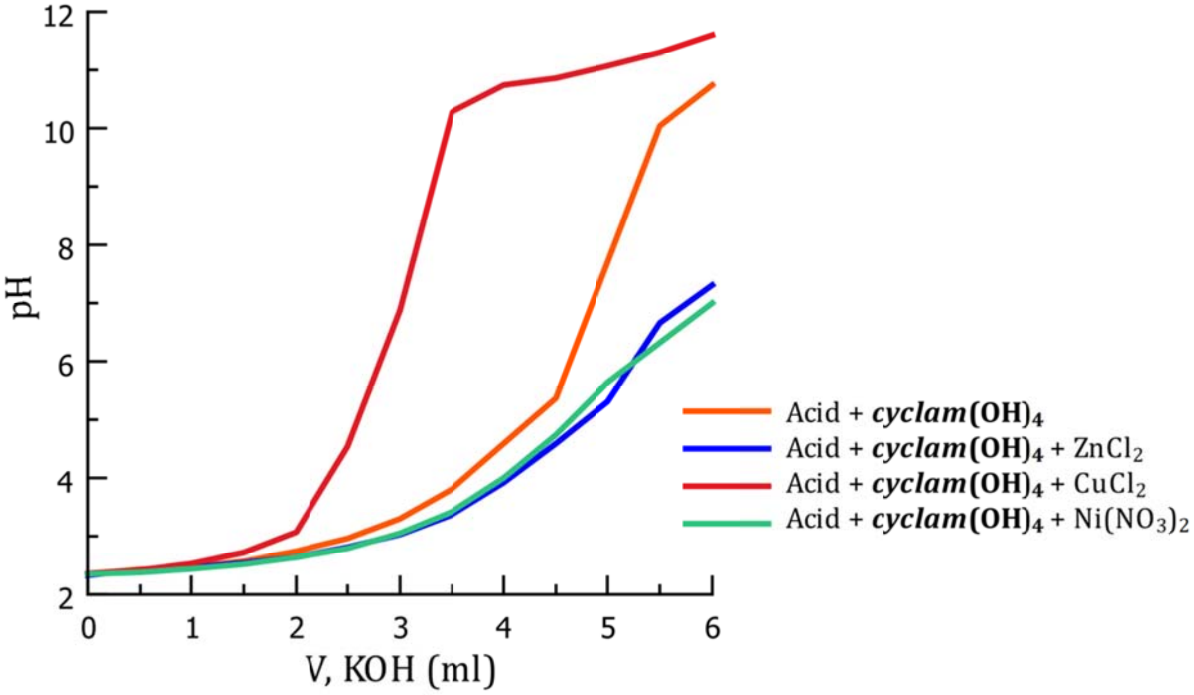

Supplementary Fig. 30. Titration curves for cyclam$(OH)_4$ and its complexes (generated *in situ*).

1.15 Quantum chemical calculations

Geometry optimization and analysis of vibrational frequencies

Quantum chemical calculations were performed with the Gaussian 16 Rev C.01 and the ORCA 4.2.1 programs. Geometry optimization and analysis of vibrational frequencies were performed with ORCA, using TPSSh DFT functional with D3BJ empirical dispersion correction. ZORA-def2-TZVPP, SARC/J basis set on Ni and ZORA-def2-SVP, SARC/J basis set on other atoms was used. Relativistic effects were taken into account by requesting a ZORA relativistic calculation with one center approximation and with inclusion of picture change effects. Cartesian coordinates are given in angstroms; absolute energies for all substances are given in hartrees. All compounds were characterized by only real vibrational frequencies. Also %scf StabpeRform true end keyword was used to check DFT wavefunction stability.

Geometry optimizations of $[Ni(cyclam(O^{-})(OH)_3)]^{+}$ with the TPSSh DFT functional using the ZORA scalar relativistic method identified two low lying stationary points having singlet and triplet multiplicities. The diamagnetic structure (consistent with NMR data) is characterized by a square-planar geometry around nickel, which is coordinated by four nitrogen atoms. In the hypothetical triplet structure, the deprotonated hydroxylamine unit coordinates nickel in a η^2-fashion.

Sample input file:

```
! RKS TPSSh OPT Numfreq ZORA RIJCOSX GRIDX6 D3BJ ZORA-def2-SVP SARC/J TightSCF Grid6 FinalGrid7 SlowConv
%method
IntAcc 7.0
end
%basis
NewGTO 28
"ZORA-def2-TZVPP"
end
NewAuxJGTO 28
"SARC/J"
end
end
%scf MaxIter 2000 end
%geom MaxIter 1000 end
*xyz 1 1
Ni        6.23380000    4.26880000    2.77610000
…
H         2.95810000    5.22160000    1.27270000
*
%rel PictureChange true OneCenter true end
```

[Ni(cyclam(O$^-$)(OH)$_3$)]$^+$ (diamagnetic)

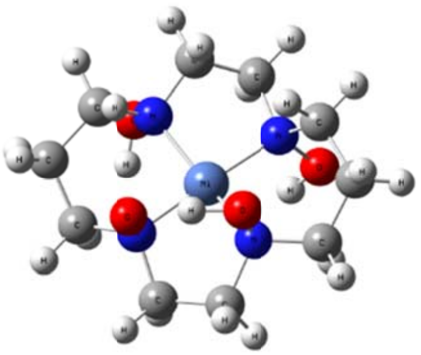

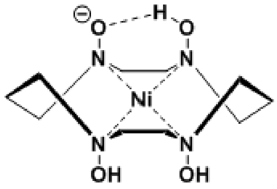

Charge 1; multiplicity 1

Cartesian atomic coordinates are provided in the Source data achieve.

DFT TPSSh; ZORA-def2-TZVPP, SARC/J (on Ni); ZORA-def2-SVP, SARC/J (on other atoms), gas phase

Total electronic energy=	-2442.398291 E_0
Zero-point energy (unscaled) =	0.36239790
Total thermal correction =	0.01833202
Total Enthalpy=	-2442.016617
Final Gibbs free energy=	-2442.078601

Number of imaginary vibrational frequencies = 0

[Ni(cyclam(O$^-$)(OH)$_3$)]$^+$ (paramagnetic)

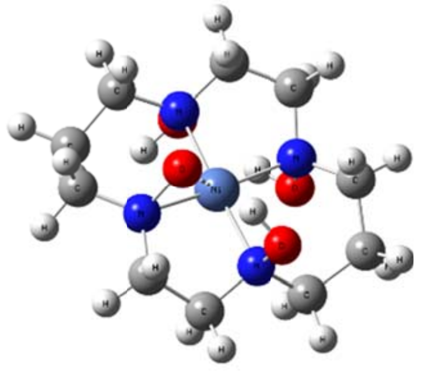

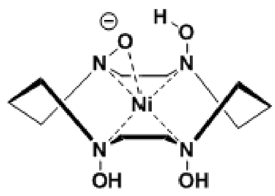

Charge 1; multiplicity 3

Cartesian atomic coordinates are provided in the Source data achieve.

DFT TPSSh; ZORA-def2-TZVPP, SARC/J (on Ni); ZORA-def2-SVP, SARC/J (on other atoms), gas phase

Total electronic energy= -2442.398408 E_0

Zero-point energy (unscaled) = 0.36302316

Total thermal correction = 0.01862641

Total Enthalpy= -2442.015814

Final Gibbs free energy= -2442.079511

Number of imaginary vibrational frequencies = 0

[Ni(cyclam(OH)$_4$)(H$_2$O)$_2$]$^{2+}$ (paramagnetic)

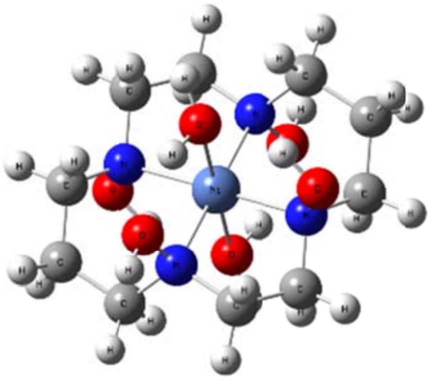

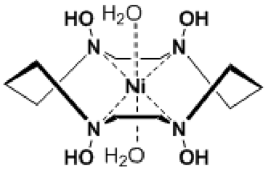

Charge 2; multiplicity 3

Cartesian atomic coordinates are provided in the Source data achieve.

DFT TPSSh; ZORA-def2-TZVPP, SARC/J (on Ni); ZORA-def2-SVP, SARC/J (on other atoms), gas phase
Total electronic energy= -2595.636261 E_0
Zero-point energy (unscaled) = 0.42787555
Total thermal correction = 0.02304767
Total Enthalpy= -2595.184393
Final Gibbs free energy= -2595.255512
Number of imaginary vibrational frequencies = 0

[Ni(cyclam(OH)$_4$)]$^{2+}$ (diamagnetic)

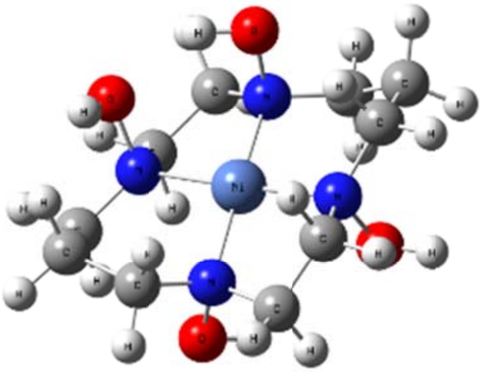

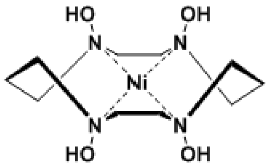

Charge 2; multiplicity 1

Cartesian atomic coordinates are provided in the Source data achieve.

DFT TPSSh; ZORA-def2-TZVPP, SARC/J (on Ni); ZORA-def2-SVP, SARC/J (on other atoms), gas phase
Total electronic energy= -2442.675511 E_0
Zero-point energy (unscaled) = 0.37669153
Total thermal correction = 0.01881440
Total Enthalpy= -2442.279061
Final Gibbs free energy= -2442.341816
Number of imaginary vibrational frequencies = 0

Calculation of d-d transitions with SA-CASSCF(10,9)

UV-Vis absorption spectra of diamagnetic cation $[Ni(cyclam(O^-)(OH)_3)]^+$ and diamagnetic dication $[Ni(cyclam(OH)_4)]^{2+}$ were calculated using QD-SC-NEVPT2 SA-CASSCF(10,9) for d-d transitions. The most intense calculated d-d transition originates as a single electron excitation from d_{xy} to $d_{x^2-y^2}$ orbital (Supplementary Figures 31 and 32, Tables 6 and 7).

Calculations were performed with ORCA. OO-RI-MP2 natural orbitals based on stable reference UHF wavefunction were used as starting orbitals for SA-CASSCF(10,9) calculation. RI approximation with nofrozencore option and def2-TZVPP def2-TZVPP/C basis sets was used throughout the calculations. In SA-CASSCF(10,9) calculation, first ten triplet states and first seven singlet states were averaged. Electron correlation correction to the CASSCF energy was computed using QD-SC-NEVPT2 theory (Nakano formulism). All calculations were performed as single point, based on ZORA optimized geometry (see above).

Input file for generation of MP2 natural orbitals:

```
! UHF OO-RI-MP2 def2-TZVPP def2-TZVPP/C SP SmallPrint TightSCF Grid6 FinalGrid7
SlowConv  MOREAD nofrozencore
%method
IntAcc 7.0
end
%moinp "650.gbw"
%mp2 natorbs true
end
%scf MaxIter 2000 end
*xyz 1 1
28      0.000000000     0.000000000     0.000000000
…
1       3.517880000    -0.354839000    -1.358348000
*
```

SA-CAS SCF(10,9) input file:

```
! def2-TZVPP def2-TZVPP/C SmallPrint moread nofrozencore
%moinp "18000.gbw"
%casscf
trafostep ri
nel 10
norb 9
mult 3,1
nroots 10,7
Maxiter 10000
PTMethod SC_NEVPT2
PTSettings
NThresh 1e-14
d4tpre 0
```

```
d3tpre 0
qdtype 1
end
end
*xyz 1 1
28      0.000000000      0.000000000      0.000000000
…
1       3.517880000     -0.354839000     -1.358348000
*
```

[Ni(cyclam(O$^-$)(OH)$_3$)]$^+$ (diamagnetic)

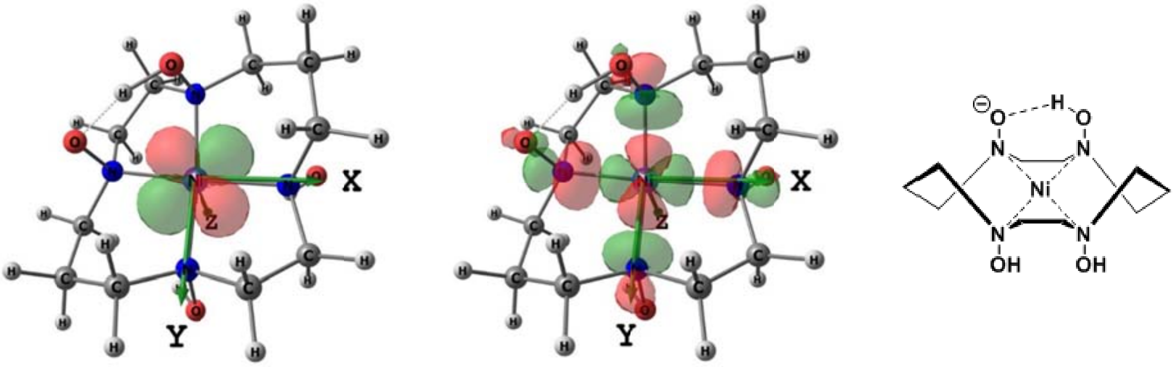

Supplementary Fig. 31. HOMO (left) and LUMO (right) SA-CASSCF natural orbitals of diamagnetic cation [Ni(cyclam(O$^-$)(OH)$_3$)]$^+$.

Supplementary Table 6. Characteristics of the most intense calculated d-d transition of UV-Vis absorption spectrum.

States	Wavelength (nm)	fosc
0(1) -> 1(1) 1	454.9	0.000141391

[Ni(*cyclam*(OH)$_4$)]$^{2+}$ (diamagnetic)

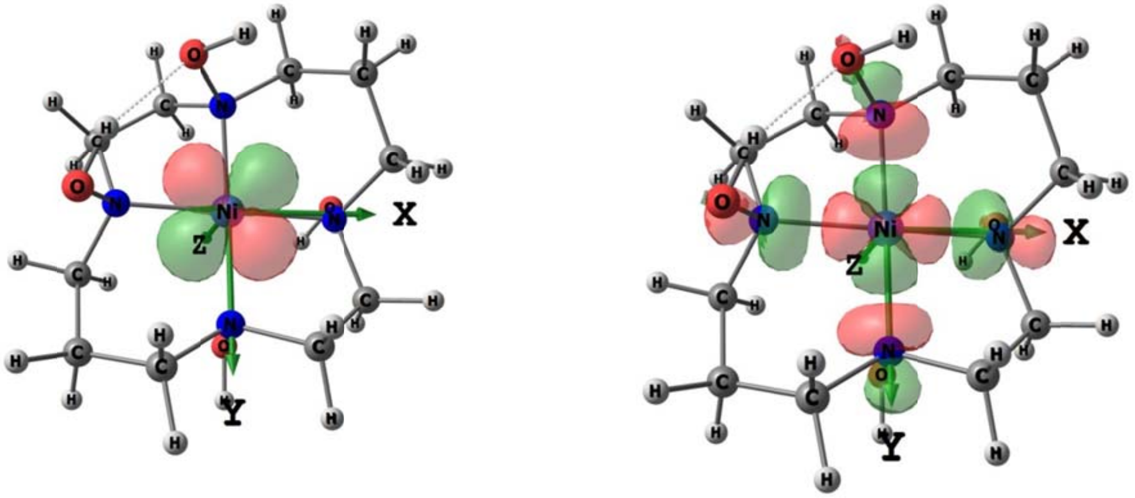

Supplementary Fig. 32. HOMO (left) and LUMO (right) SA-CASSCF natural orbitals of square planar cation [Ni(cyclam(OH)$_4$)]$^{2+}$.

Supplementary Table 7. Characteristics of the most intense calculated d-d transition of UV-Vis absorption spectrum.

States	Wavelength (nm)	fosc
0(1) -> 1(1) 1	462.9	0.000047223

Calculation of LMCT excited states with TD-DFT

UV-Vis absorption spectrum of $[Ni(cyclam(O^{-})(OH)_3)]^+$ (Supplementary Figure 33) were calculated with Gaussian, using TD-DFT with ωB97X-D functional and Def2TZVPP basis set on all atoms. Single point calculation was performed on ZORA optimized geometry (see above) to obtain 20 lowest excited states of diamagnetic cation $[Ni(cyclam(O^{-})(OH)_3)]^+$. Wavefunction stability, using *stable* keyword, was also checked. Then NPA atomic charges (two sets: one - for ground state, the other for fifth excited state, Supplementary Table 8), natural transition orbitals (Supplementary Figure 34) and electron difference density (between ground state and fifth excited state, Supplementary Figure 35) were calculated. Same computation protocol was used for diamagnetic dication $[Ni(cyclam(OH)_4)]^{2+}$ (Supplementary Figures 36–40, Tables 9 and 10).

Keywords for TD-DFT input file are:

%Chk=e8_03_xs

TD(NStates=20) wB_97XD Def_2TZVPP nosymm test

Keywords for NPA charges input file (example for fifth excited state) are:

%OldChk=e8_03_xs

%Chk=e8_03_xs5

wB97XD Def2TZVPP TD(NStates=20,Read,Root=5) Density=Current Pop=NPA Geom=AllCheck Guess=Read nosymm test

Keywords for natural transition orbitals are:

%OldChk=e8_03_xs

%chk=e8_03_nto_5

#ChkBasis guess=(only,read) pop=(savento) geom=allcheck density=(check,transition=5) nosymm test

[Ni(*cyclam(O$^-$)*(OH)$_3$)]$^+$ (diamagnetic)

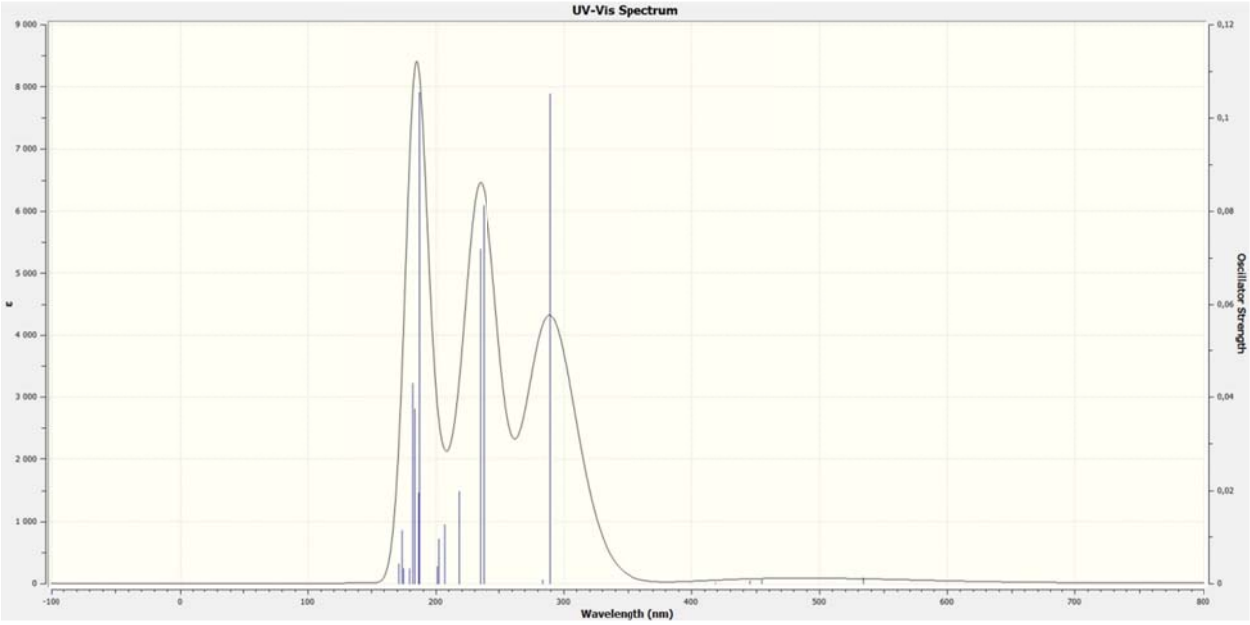

Supplementary Fig. 33. Calculated TD-DFT UV-Vis absorption spectrum of diamagnetic cation **[Ni(*cyclam(O$^-$)*(OH)$_3$)]$^+$**.

Raw data for first 20 singlet excited states of diamagnetic cation [Ni(cyclam(O$^-$)(OH)$_3$)]$^+$ in a vertical approximation.

Excitation energies and oscillator strengths:

Excited State 1: Singlet-?Sym 2.3213 eV 534.11 nm f=0.0013 <S**2>=0.000
77 -> 86 0.11275
82 -> 86 0.35451
83 -> 86 0.50914
85 -> 86 -0.26765
Total Energy, E(TD-HF/TD-DFT) = -2422.84304060
Excited State 2: Singlet-?Sym 2.7299 eV 454.17 nm f=0.0008 <S**2>=0.000
80 -> 86 0.49292
81 -> 86 -0.41311
82 -> 86 -0.18436
85 -> 86 -0.14906
Excited State 3: Singlet-?Sym 2.7850 eV 445.18 nm f=0.0005 <S**2>=0.000
77 -> 86 0.10555
80 -> 86 0.43154
81 -> 86 0.52778
Excited State 4: Singlet-?Sym 2.9638 eV 418.33 nm f=0.0001 <S**2>=0.000
73 -> 86 -0.10675
74 -> 86 0.20906
76 -> 86 0.12729
77 -> 86 0.12491
78 -> 86 0.52058
79 -> 86 0.33587

Excited State 5: Singlet-?Sym 4.2820 eV 289.55 nm f=0.1052 <S2>=0.000**
80 -> 86 0.20055
82 -> 86 0.12238
83 -> 86 0.17679
85 -> 86 0.62143
Excited State 6: Singlet-?Sym 4.3660 eV 283.97 nm f=0.0008 <S**2>=0.000
84 -> 86 0.69075
Excited State 7: Singlet-?Sym 5.2313 eV 237.00 nm f=0.0811 <S**2>=0.000
82 -> 86 -0.32619
82 -> 87 -0.15121
83 -> 86 0.30529
83 -> 87 -0.18357
85 -> 87 0.45348
Excited State 8: Singlet-?Sym 5.2937 eV 234.21 nm f=0.0718 <S**2>=0.000
82 -> 86 0.40939
83 -> 86 -0.29328
83 -> 87 -0.16029
85 -> 87 0.42169
Excited State 9: Singlet-?Sym 5.6901 eV 217.89 nm f=0.0196 <S**2>=0.000
80 -> 87 -0.10771
82 -> 87 0.20958
83 -> 87 0.55766
85 -> 87 0.27568
Excited State 10: Singlet-?Sym 6.0057 eV 206.44 nm f=0.0126 <S**2>=0.000
81 -> 87 0.54483
82 -> 87 0.21379
84 -> 87 0.33396
Excited State 11: Singlet-?Sym 6.1343 eV 202.12 nm f=0.0095 <S**2>=0.000
80 -> 87 0.55540
81 -> 87 -0.15524
83 -> 87 0.12370
84 -> 87 0.30124
Excited State 12: Singlet-?Sym 6.1645 eV 201.13 nm f=0.0037 <S**2>=0.000
80 -> 87 -0.34751
81 -> 87 -0.27801
84 -> 87 0.49981
Excited State 13: Singlet-?Sym 6.6187 eV 187.32 nm f=0.1054 <S**2>=0.000
77 -> 86 -0.23320
78 -> 86 -0.17403
79 -> 86 0.39089
82 -> 86 0.10320
85 -> 88 0.34675
85 -> 89 -0.23124
Excited State 14: Singlet-?Sym 6.6440 eV 186.61 nm f=0.0193 <S**2>=0.000
77 -> 86 0.34835
79 -> 86 -0.28137
85 -> 88 0.41529
85 -> 89 -0.20247
85 -> 91 -0.11752
Excited State 15: Singlet-?Sym 6.7495 eV 183.69 nm f=0.0375 <S**2>=0.000
77 -> 86 0.18115
78 -> 86 -0.12889

```
     79 -> 86         0.16764
     81 -> 87        -0.20164
     82 -> 87         0.49379
     83 -> 87        -0.23854
 Excited State  16:      Singlet-?Sym    6.8086 eV  182.10 nm  f=0.0430  <S**2>=0.000
     77 -> 86         0.44370
     78 -> 86        -0.23415
     79 -> 86         0.21389
     79 -> 87        -0.10562
     81 -> 86        -0.12057
     82 -> 87        -0.24876
     85 -> 88        -0.16392
 Excited State  17:      Singlet-?Sym    6.9002 eV  179.68 nm  f=0.0032  <S**2>=0.000
     74 -> 87         0.13513
     77 -> 87         0.14930
     78 -> 86        -0.11815
     78 -> 87         0.46792
     79 -> 87         0.38899
 Excited State  18:      Singlet-?Sym    7.0959 eV  174.73 nm  f=0.0033  <S**2>=0.000
     72 -> 86         0.10027
     74 -> 86         0.49283
     75 -> 86         0.13673
     76 -> 86         0.34348
     78 -> 86        -0.21676
     82 -> 86         0.11759
 Excited State  19:      Singlet-?Sym    7.1335 eV  173.81 nm  f=0.0113  <S**2>=0.000
     76 -> 86        -0.13659
     82 -> 89        -0.11611
     82 -> 90        -0.11436
     83 -> 88         0.23064
     83 -> 89        -0.29924
     85 -> 88         0.16166
     85 -> 89         0.20166
     85 -> 90         0.31857
     85 -> 92        -0.11243
     85 -> 93         0.10154
 Excited State  20:      Singlet-?Sym    7.2326 eV  171.42 nm  f=0.0042  <S**2>=0.000
     74 -> 86         0.13477
     76 -> 86        -0.12661
     78 -> 87        -0.25879
     79 -> 87         0.24823
     82 -> 88         0.14436
     83 -> 88         0.32648
     83 -> 89        -0.13847
     85 -> 90        -0.22805
     85 -> 92         0.15253
```

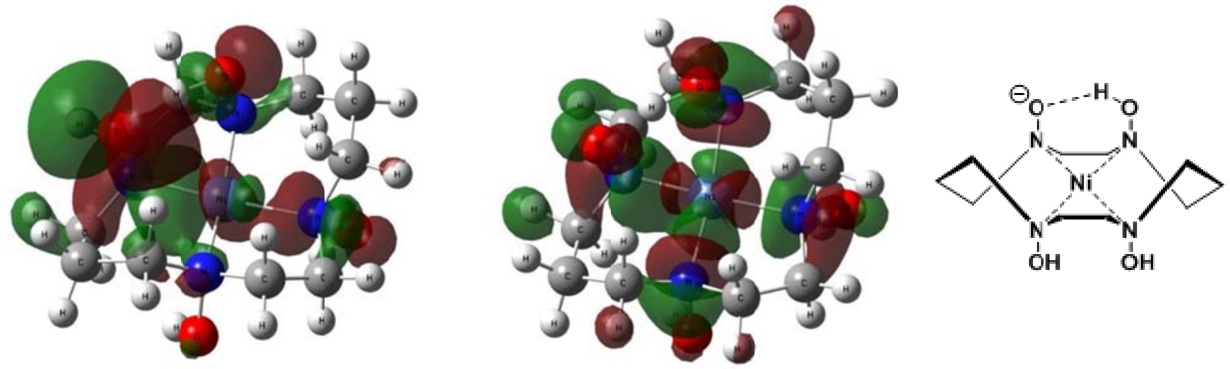

Supplementary Fig. 34. HOTO (left) and LUTO (right) TD-DFT natural transition orbitals of diamagnetic cation $[Ni(cyclam(O^{-})(OH)_3)]^{+}$.

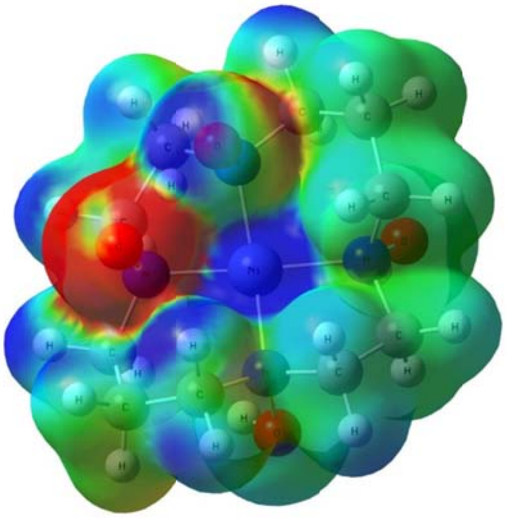

Supplementary Fig. 35. Isosurface of ground state electron density mapped using the value of the difference density (CI - SCF) for diamagnetic cation $[Ni(cyclam(O^{-})(OH)_3)]^{+}$. Isovalue = 0.01. Blue region - electron density in the fifth excited state is larger than it is in the ground state, red region – smaller.

Supplementary Table 8. Calculated NPA charges for diamagnetic cation $[Ni(cyclam(O^{-})(OH)_3)]^{+}$.

№	Atom	NPA charge		Selected Δ
		**Ground state**	**Excited state 5**	
1	Ni	0.56985	0.41831	-0.15
2	N	-0.13899	-0.15195	
3	N	-0.14428	-0.12125	
4	N	-0.13039	-0.16294	
5	N	-0.11444	-0.19087	-0.08
6	C	-0.21724	-0.21843	
7	H	0.23145	0.21632	
8	H	0.17980	0.19698	
9	C	-0.43077	-0.43293	
10	O	-0.54841	-0.55630	
11	C	-0.24676	-0.24438	
12	C	-0.23415	-0.22850	
13	O	-0.53911	-0.54224	
14	H	0.47291	0.47575	
15	C	-0.24677	-0.24669	
16	C	-0.22765	-0.22835	
17	H	0.47274	0.46954	
18	H	0.23095	0.22495	
19	H	0.24293	0.23381	
20	C	-0.25032	-0.25642	
21	O	-0.57834	-0.56314	
22	C	-0.21893	-0.21866	
23	C	-0.42421	-0.42317	
24	H	0.23751	0.23681	
25	H	0.24643	0.24168	
26	C	-0.24067	-0.23868	
27	H	0.23441	0.21847	
28	H	0.20650	0.22447	
29	H	0.22401	0.22880	
30	H	0.24312	0.23627	
31	H	0.24023	0.23962	
32	H	0.23199	0.22678	
33	H	0.22135	0.21713	
34	H	0.24893	0.23145	
35	H	0.23075	0.23706	
36	H	0.25455	0.24554	
37	O	-0.66626	-0.34276	+0.32
38	H	0.48252	0.46763	
39	H	0.22604	0.21186	
40	H	0.21663	0.22010	
41	H	0.23265	0.22992	
42	H	0.21945	0.21844	

[Ni(cyclam(OH)$_4$)]$^{2+}$ (diamagnetic)

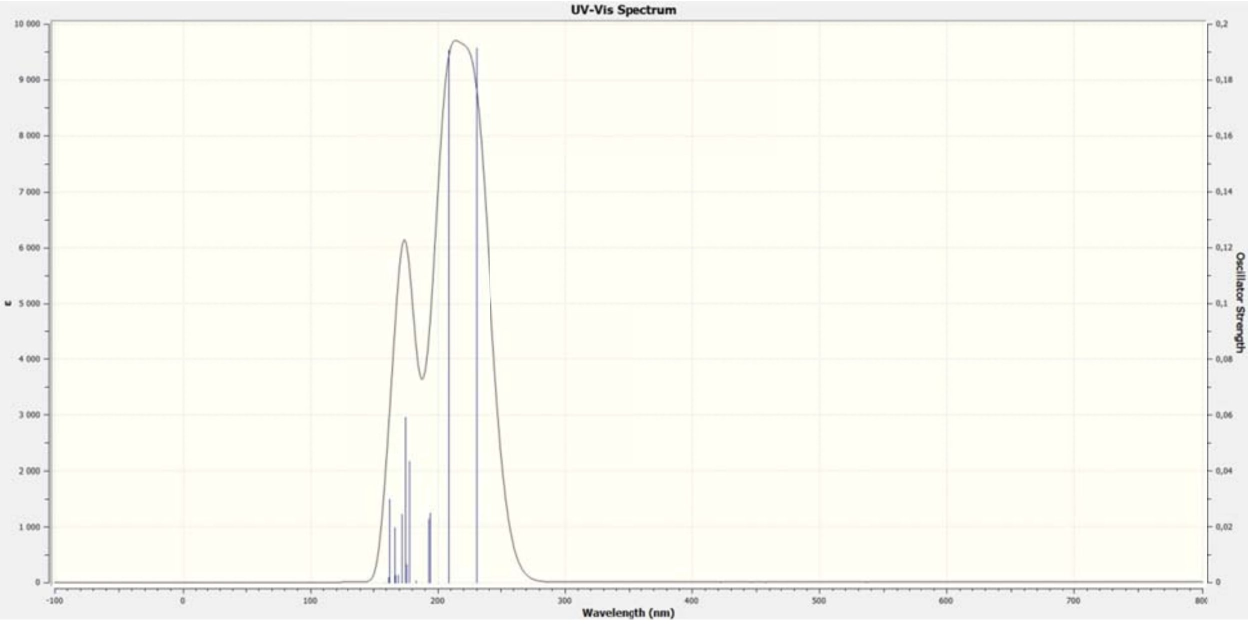

Supplementary Fig. 36. Calculated TD-DFT UV-Vis absorption spectrum of diamagnetic dication [Ni(cyclam(OH)$_4$)]$^{2+}$.

Raw data for first 20 singlet excited states of [Ni(cyclam(OH)$_4$)]$^{2+}$ in a vertical approximation.

Excited State 1: Singlet-?Sym 2.3103 eV 536.65 nm f=0.0001 <S**2>=0.000
72 -> 86 -0.10028
78 -> 86 0.11166
80 -> 86 0.13786
84 -> 86 0.21261
85 -> 86 0.63098
85 <- 86 -0.10284
Excited State 2: Singlet-?Sym 2.7139 eV 456.85 nm f=0.0001 <S**2>=0.000
80 -> 86 0.17514
82 -> 86 0.27585
83 -> 86 0.59265
84 -> 86 -0.12636
Excited State 3: Singlet-?Sym 2.7774 eV 446.41 nm f=0.0001 <S**2>=0.000
81 -> 86 0.67843
82 -> 86 -0.10269
84 -> 86 -0.10704
Excited State 4: Singlet-?Sym 2.9389 eV 421.87 nm f=0.0000 <S**2>=0.000
72 -> 86 0.17771
73 -> 86 0.24616
76 -> 86 0.10667
77 -> 86 -0.22897
78 -> 86 0.39596
79 -> 86 0.39311
Excited State 5: Singlet-?Sym 5.3943 eV 229.84 nm f=0.1916 <S2>=0.000**

83 -> 86 0.10911
84 -> 86 0.63375
85 -> 86 -0.23237
Excited State 6: Singlet-?Sym 5.9488 eV 208.42 nm f=0.1908 <S2>=0.000**
78 -> 86 0.11287
81 -> 86 0.11052
82 -> 86 0.57518
83 -> 86 -0.31364
85 -> 86 -0.11583
Excited State 7: Singlet-?Sym 6.3894 eV 194.05 nm f=0.0248 <S**2>=0.000
72 -> 86 -0.10194
78 -> 86 0.13876
79 -> 86 -0.11682
80 -> 86 0.43452
82 -> 86 -0.16341
83 -> 86 -0.10006
85 -> 86 -0.14746
85 -> 87 -0.38849
Excited State 8: Singlet-?Sym 6.4244 eV 192.99 nm f=0.0226 <S**2>=0.000
78 -> 86 0.10046
80 -> 86 0.30058
82 -> 86 -0.13012
84 -> 87 0.14508
85 -> 87 0.53064
85 -> 88 -0.10397
Excited State 9: Singlet-?Sym 6.7690 eV 183.17 nm f=0.0007 <S**2>=0.000
75 -> 86 0.11161
77 -> 86 0.29779
78 -> 86 -0.30126
79 -> 86 0.48584
80 -> 86 0.22179
Excited State 10: Singlet-?Sym 6.9616 eV 178.10 nm f=0.0433 <S**2>=0.000
80 -> 87 0.10993
81 -> 87 0.10395
82 -> 87 0.16973
83 -> 87 0.44409
84 -> 87 -0.42808
Excited State 11: Singlet-?Sym 7.0558 eV 175.72 nm f=0.0067 <S**2>=0.000
72 -> 86 0.20934
74 -> 86 -0.11874
75 -> 86 0.21784
76 -> 86 0.10289
77 -> 86 -0.16699
78 -> 86 -0.15272
79 -> 86 -0.12953
80 -> 86 0.12393
81 -> 87 0.37232
82 -> 87 -0.14563

83 -> 87 -0.19294
85 -> 88 -0.18369
Excited State 12: Singlet-?Sym 7.0833 eV 175.04 nm f=0.0592 <S**2>=0.000
72 -> 86 -0.18638
74 -> 86 0.12064
75 -> 86 -0.16331
77 -> 86 0.18588
78 -> 86 0.14471
79 -> 86 0.10214
81 -> 87 0.37465
82 -> 87 -0.18024
83 -> 87 -0.16888
84 -> 87 -0.19216
84 -> 88 0.10180
85 -> 88 0.20041
Excited State 13: Singlet-?Sym 7.1922 eV 172.39 nm f=0.0034 <S**2>=0.000
76 -> 86 0.23914
81 -> 87 0.25917
83 -> 87 0.28650
84 -> 87 0.34937
85 -> 88 0.21400
85 -> 90 -0.11309
Excited State 14: Singlet-?Sym 7.2000 eV 172.20 nm f=0.0242 <S**2>=0.000
73 -> 86 0.15813
75 -> 86 0.12874
76 -> 86 0.37883
77 -> 86 0.12795
79 -> 86 -0.10584
81 -> 87 -0.25755
83 -> 87 -0.16400
84 -> 87 -0.14471
84 -> 88 0.13472
85 -> 88 0.27889
Excited State 15: Singlet-?Sym 7.3291 eV 169.17 nm f=0.0029 <S**2>=0.000
70 -> 86 0.10108
72 -> 86 -0.14946
76 -> 86 0.30050
77 -> 86 0.10927
80 -> 87 -0.10567
81 -> 87 0.10013
82 -> 87 0.31776
82 -> 89 0.10951
83 -> 87 -0.12966
84 -> 89 -0.11789
85 -> 88 -0.19911
85 -> 89 -0.18382
85 -> 90 -0.12156
Excited State 16: Singlet-?Sym 7.4256 eV 166.97 nm f=0.0025 <S**2>=0.000

70 -> 86 -0.11175
72 -> 86 0.33797
73 -> 86 0.22066
76 -> 86 -0.27354
77 -> 86 0.28621
79 -> 86 -0.13047
82 -> 87 0.17316
85 -> 88 0.11986
85 -> 89 -0.10196
85 -> 90 -0.12835
Excited State 17: Singlet-?Sym 7.4505 eV 166.41 nm f=0.0196 <S**2>=0.000
72 -> 86 0.11360
73 -> 86 -0.27662
75 -> 86 0.25242
77 -> 86 0.33831
78 -> 86 0.30949
80 -> 86 -0.13067
82 -> 87 -0.11775
85 -> 88 -0.16654
85 -> 90 0.11555
Excited State 18: Singlet-?Sym 7.6176 eV 162.76 nm f=0.0296 <S**2>=0.000
70 -> 86 0.11717
73 -> 86 0.35575
74 -> 86 -0.14967
75 -> 86 -0.32765
77 -> 86 0.19139
82 -> 87 -0.15069
85 -> 88 -0.22509
85 -> 90 0.10987
Excited State 19: Singlet-?Sym 7.6621 eV 161.82 nm f=0.0020 <S**2>=0.000
78 -> 87 0.15422
80 -> 87 0.39207
80 -> 88 -0.10143
80 -> 89 -0.11468
84 -> 88 0.13037
84 -> 89 0.16885
84 -> 90 0.12480
84 -> 92 0.18975
84 -> 95 -0.12675
85 -> 88 -0.14484
85 -> 90 -0.13240
Excited State 20: Singlet-?Sym 7.7750 eV 159.46 nm f=0.0001 <S**2>=0.000
72 -> 87 0.12531
73 -> 87 0.17662
77 -> 87 -0.18705
78 -> 87 0.38526
79 -> 87 0.44909

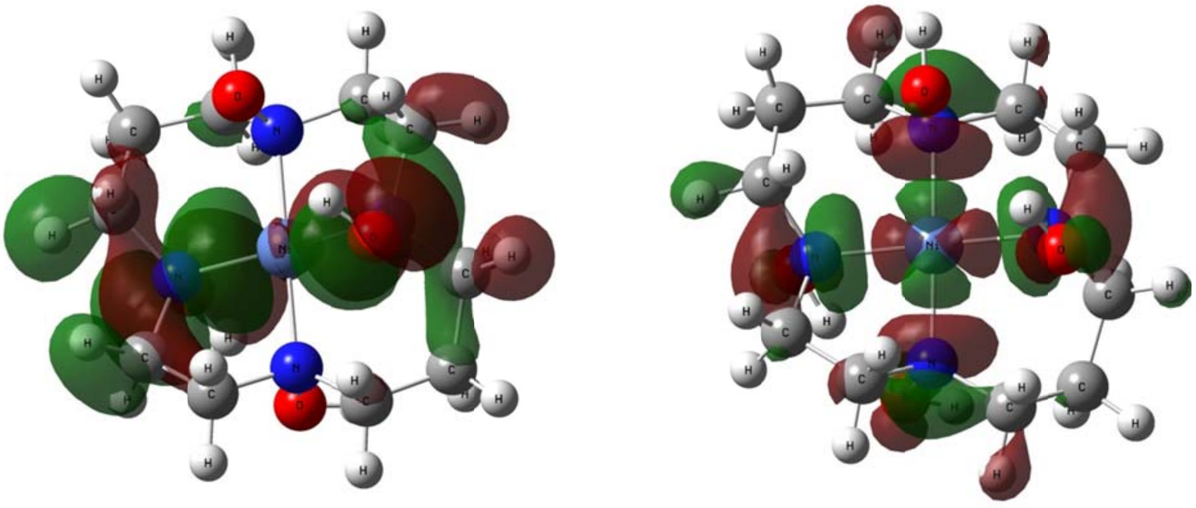

Supplementary Fig. 37. HOTO (left) and LUTO (right) TD-DFT natural transition orbitals of diamagnetic dication $[Ni(cyclam(OH)_4)]^{2+}$ (ground to fifth excited state).

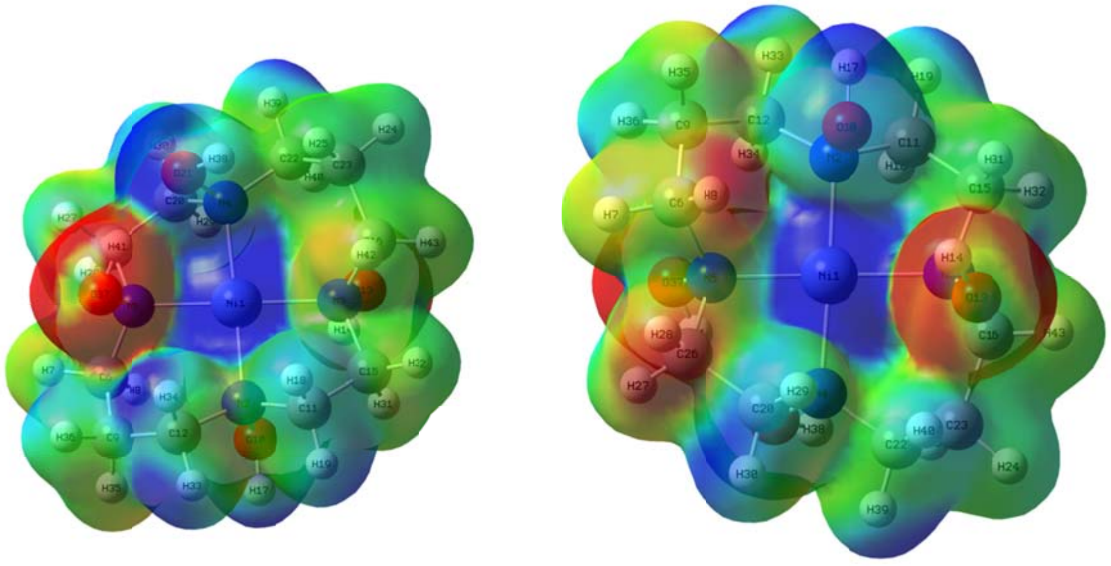

Supplementary Fig. 38. Isosurface of ground state electron density mapped using the value of the difference density (CI - SCF) for diamagnetic dication $[Ni(cyclam(OH)_4)]^{2+}$. Isovalue = 0.01. Blue region - electron density in the fifth excited state is larger than it is in the ground state, red region – smaller.

Supplementary Table 9. Calculated NPA charges for diamagnetic dication $[Ni(cyclam(OH)_4)]^{2+}$.

№	Atom	NPA charge		Selected Δ
		**Ground state**	**Excited state 5**	
1	Ni	0.66005	0.42073	-0.24
2	N	-0.12352	-0.15744	
3	N	-0.13190	-0.08294	+0.05
4	N	-0.12007	-0.15464	
5	N	-0.12936	-0.09045	
6	C	-0.22749	-0.23348	
7	H	0.24324	0.24956	
8	H	0.23193	0.24816	
9	C	-0.43704	-0.43799	
10	O	-0.54765	-0.55893	
11	C	-0.25476	-0.25408	
12	C	-0.24218	-0.23735	
13	O	-0.52929	-0.45567	+0.07
14	H	0.48713	0.49595	
15	C	-0.25354	-0.25232	
16	C	-0.23566	-0.23916	
17	H	0.48185	0.47367	
18	H	0.23908	0.23554	
19	H	0.24397	0.23603	
20	C	-0.24639	-0.24935	
21	O	-0.56136	-0.59076	
22	C	-0.24955	-0.24668	
23	C	-0.44376	-0.44016	
24	H	0.27913	0.27539	
25	H	0.21174	0.21261	
26	C	-0.25967	-0.25274	
27	H	0.25653	0.25099	
28	H	0.25090	0.26113	
29	H	0.23888	0.23887	
30	H	0.25428	0.24990	
31	H	0.24734	0.24544	
32	H	0.25287	0.25264	
33	H	0.22920	0.22114	
34	H	0.24532	0.23442	
35	H	0.24630	0.25143	
36	H	0.26238	0.25716	
37	O	-0.53456	-0.35155	+ 0.18
38	H	0.49280	0.49237	
39	H	0.24454	0.23466	
40	H	0.25846	0.25134	
41	H	0.49477	0.51366	
42	H	0.22915	0.23308	
43	H	0.24592	0.24884	

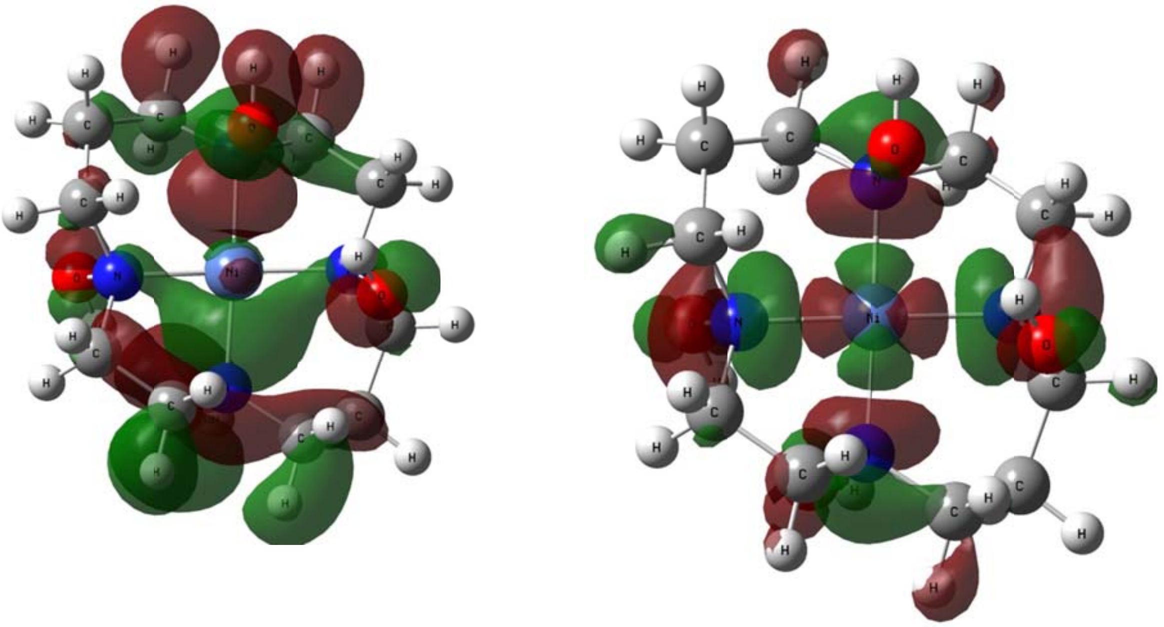

Supplementary Fig. 39. HOTO (left) and LUTO (right) TD-DFT natural transition orbitals of diamagnetic dication $[Ni(cyclam(OH)_4)]^{2+}$ (ground to sixth excited state).

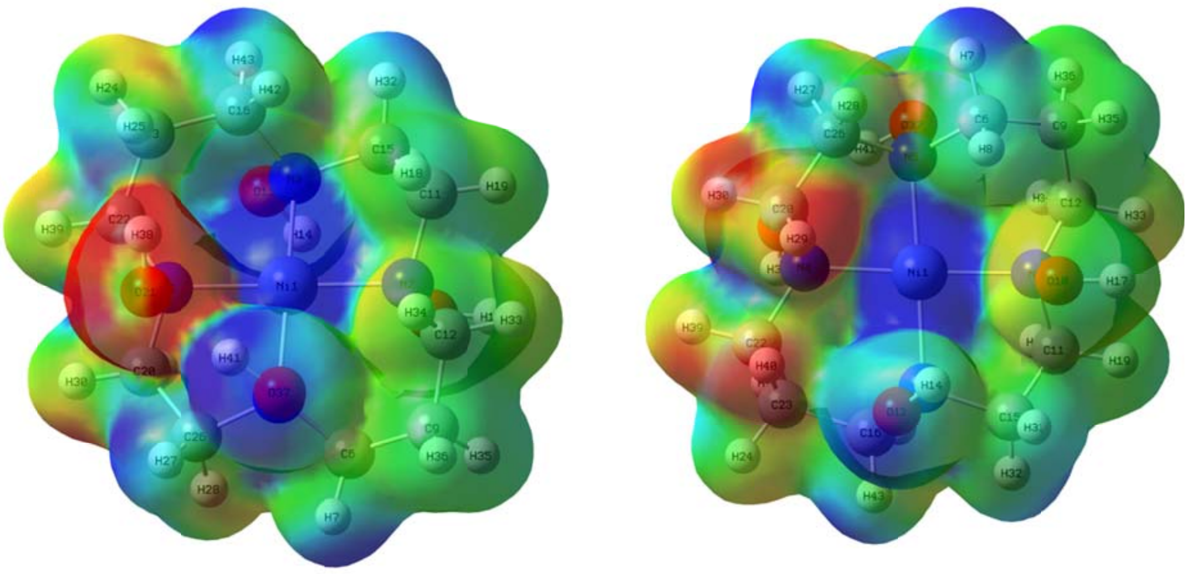

Supplementary Fig. 40. Isosurface of ground state electron density mapped using the value of the difference density (CI - SCF) for diamagnetic dication $[Ni(cyclam(OH)_4)]^{2+}$. Isovalue = 0.01. Blue region - electron density in the sixth excited state is larger than it is in the ground state, red region – smaller.

Supplementary Table 10. Calculated NPA charges for diamagnetic dication $[Ni(cyclam(OH)_4)]^{2+}$.

№	Atom	NPA charge		Selected Δ
		Ground state	Excited state 6	
1	Ni	0.66005	0.40988	-0.25
2	N	-0.12352	-0.06878	+0.05
3	N	-0.13190	-0.16562	
4	N	-0.12007	-0.02710	+0.09
5	N	-0.12936	-0.17623	-0.05
6	C	-0.22749	-0.22385	
7	H	0.24324	0.23176	
8	H	0.23193	0.22979	
9	C	-0.43704	-0.43519	
10	O	-0.54765	-0.53379	
11	C	-0.25476	-0.25288	
12	C	-0.24218	-0.24291	
13	O	-0.52929	-0.53811	
14	H	0.48713	0.48805	
15	C	-0.25354	-0.25495	
16	C	-0.23566	-0.23658	
17	H	0.48185	0.48336	
18	H	0.23908	0.23868	
19	H	0.24397	0.24375	
20	C	-0.24639	-0.25404	
21	O	-0.56136	-0.38882	+0.17
22	C	-0.24955	-0.25260	
23	C	-0.44376	-0.44268	
24	H	0.27913	0.28354	
25	H	0.21174	0.20696	
26	C	-0.25967	-0.26308	
27	H	0.25653	0.25060	
28	H	0.25090	0.25337	
29	H	0.23888	0.25210	
30	H	0.25428	0.26073	
31	H	0.24734	0.24369	
32	H	0.25287	0.24501	
33	H	0.22920	0.23971	
34	H	0.24532	0.25073	
35	H	0.24630	0.24474	
36	H	0.26238	0.26086	
37	O	-0.53456	-0.54047	
38	H	0.49280	0.50641	
39	H	0.24454	0.25115	
40	H	0.25846	0.27431	
41	H	0.49477	0.47799	
42	H	0.22915	0.22882	
43	H	0.24592	0.24052	

2. Supplementary references

1. Fulmer GR*, et al.* NMR Chemical Shifts of Trace Impurities: Common Laboratory Solvents, Organics, and Gases in Deuterated Solvents Relevant to the Organometallic Chemist. *Organometallics* **29**, 2176-2179 (2010).

2. Frisch, M. J.; Trucks, G. W.; Schlegel, H. B.; Scuseria, G. E.; Robb, M. A.; Cheeseman, J. R.; Scalmani, G.; Barone, V.; Petersson, G. A.; Nakatsuji, H.; Li, X.; Caricato, M.; Marenich, A. V.; Bloino, J.; Janesko, B. G.; Gomperts, R.; Mennucci, B.; Hratchian, H. P.; Ortiz, J. V.; Izmaylov, A. F.; Sonnenberg, J. L.; Williams; Ding, F.; Lipparini, F.; Egidi, F.; Goings, J.; Peng, B.; Petrone, A.; Henderson, T.; Ranasinghe, D.; Zakrzewski, V. G.; Gao, J.; Rega, N.; Zheng, G.; Liang, W.; Hada, M.; Ehara, M.; Toyota, K.; Fukuda, R.; Hasegawa, J.; Ishida, M.; Nakajima, T.; Honda, Y.; Kitao, O.; Nakai, H.; Vreven, T.; Throssell, K.; Montgomery Jr., J. A.; Peralta, J. E.; Ogliaro, F.; Bearpark, M. J.; Heyd, J. J.; Brothers, E. N.; Kudin, K. N.; Staroverov, V. N.; Keith, T. A.; Kobayashi, R.; Normand, J.; Raghavachari, K.; Rendell, A. P.; Burant, J. C.; Iyengar, S. S.; Tomasi, J.; Cossi, M.; Millam, J. M.; Klene, M.; Adamo, C.; Cammi, R.; Ochterski, J. W.; Martin, R. L.; Morokuma, K.; Farkas, O.; Foresman, J. B.; Fox, D. J. Gaussian 16 Rev. C.01, Wallingford, CT, 2016.

3. Neese F. Software update: the ORCA program system, version 4.0. *WIREs Computational Molecular Science* **8**, e1327 (2018).

4. Searle G, Geue R. Improved Richman-Atkins syntheses of cyclic polyamines particularly 1,4,7-Triazacyclononane (tacn) and 1,4,7-Triazacyclodecane (tacd), with the aid of cation-exchange in purification and isolation. *Aust J Chem* **37**, 959-970 (1984).

5. Bencini A, Burguete MI, Garcia-Espana E, Luis SV, Miravet JF, Soriano C. An efficient synthesis of polyaza[n]paracyclophanes. *J Org Chem* **58**, 4749-4753 (1993).

6. Bazzicalupi C*, et al.* Exploring the Binding Ability of Phenanthroline-Based Polyammonium Receptors for Anions: Hints for Design of Selective Chemosensors for Nucleotides. *J Org Chem* **74**, 7349-7363 (2009).

7. Bell TW, Choi H-J, Harte W, Drew MGB. Syntheses, Conformations, and Basicities of Bicyclic Triamines. *J Am Chem Soc* **125**, 12196-12210 (2003).

8. Osvath P, Curtis N, Weatherburn D. Copper(II) and Nickel(II) Complexes of Pentaaza Macrocyclic Ligands. *Aust J Chem* **40**, 347-360 (1987).

9. Hiroyuki K, Tamotsu Y. Syntheses of Some Medium Sized Cyclic Triamines and Their Cobalt(III) Complexes. *Bull Chem Soc Jpn* **45**, 481-484 (1972).

10. Brandès S, Gros C, Denat F, Pullumbi P, Guilard R. New facile and convenient synthesis of bispolyazamacrocycles using Boc protection. Determination of geometric parameters of dinuclear copper (II) complexes using ESR spectroscopy and molecular mechanics calculations. *Bull Soc Chim Fr* **1**, 65-73 (1996).

11. Her B, Jones A, Wollack JW. A Three-Step Synthesis of Benzoyl Peroxide. *J Chem Educ* **91**, 1491-1494 (2014).

12. Semakin AN, Nelyubina YV, Ioffe SL, Sukhorukov AY. 2,4,9-Triazaadamantanes with “Clickable” Groups: Synthesis, Structure and Applications as Tripodal Platforms. *Eur J Org Chem* **2020**, 6723-6735 (2020).

13. Hisler K, Commeureuc AGJ, Zhou S-z, Murphy JA. Synthesis of indoles via alkylidenation of acyl hydrazides. *Tetrahedron Lett* **50**, 3290-3293 (2009).

14. Yu J, Wang S, Wen J, Wang J, Li J-H. A Novel Approach to N′,N′-Diarylhydrazides from N′-Arylhydrazides. *Synlett* **26**, 1121-1123 (2015).

15. Golovanov IS*, et al.* Iron(iv) complexes with tetraazaadamantane-based ligands: synthesis, structure, applications in dioxygen activation and labeling of biomolecules. *Dalton Transactions* **51**, 4284-4296 (2022).

16. CrysAlisPro. Version 1.171.41.106a. Rigaku Oxford Diffraction, 2021.

17. Sheldrick G. SHELXT - Integrated space-group and crystal-structure determination. *Acta Cryst* **71**, 3-8 (2015).

18. Sheldrick G. Crystal structure refinement with SHELXL. *Acta Cryst* **71**, 3-8 (2015).

19. Dolomanov OV, Bourhis LJ, Gildea RJ, Howard JAK, Puschmann H. OLEX2: a complete structure solution, refinement and analysis program. *J Appl Crystallogr* **42**, 339-341 (2009).

20. Mellor DP, Maley L. Order of Stability of Metal Complexes. *Nature* **161**, 436-437 (1948).
